# Halogenation Reactions of Alkyl Alcohols Employing Methyl Grignard Reagents

**DOI:** 10.1021/acs.joc.2c01590

**Published:** 2022-09-01

**Authors:** Nadia Hirbawi, Patricia C. Lin, Elizabeth R. Jarvo

**Affiliations:** Department of Chemistry, University of California, Irvine, California 92697-2025, United States

## Abstract

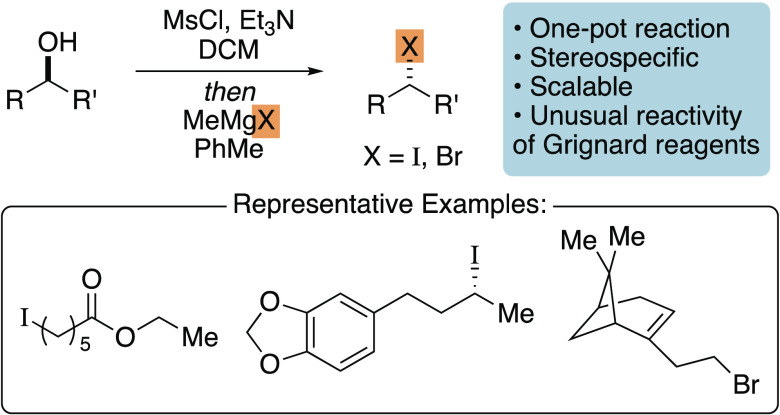

Grignard reagents are commonly used as carbanion equivalents. Herein, we report an example of Grignard reagents acting as halide nucleophiles to form alkyl iodides and bromides. We establish that Grignard reagents can convert alkyl mesylates into alkyl halides, as well as be employed in a one-pot halogenation reaction starting from alcohols, which proceed through mesylate intermediates. The halogenation reaction is confirmed to occur by an S_N_2 pathway with inversion of configuration and is demonstrated to be efficient on multi-gram scale.

## Introduction

**Scheme 1:**
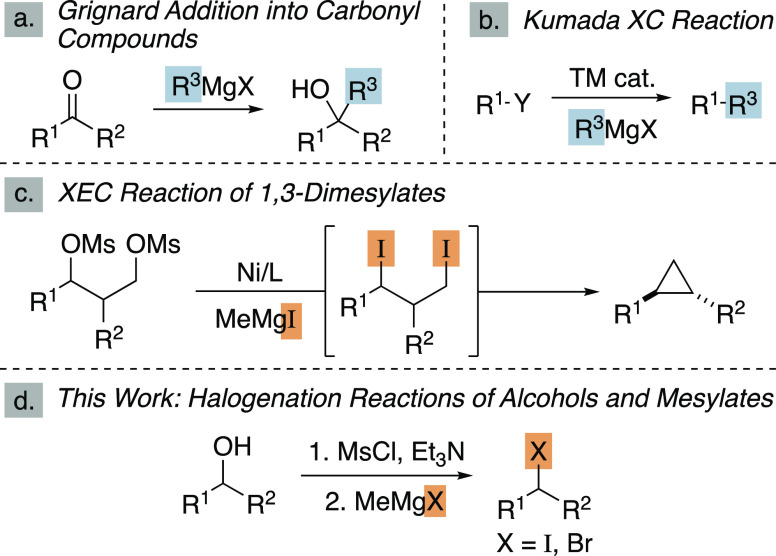
Grignard Reagents as Carbanion Equivalents and Halide Nucleophiles R^1^, R^3^ = Ar, alkyl; R^2^ = Ar, alkyl, H.

Since their discovery by Victor Grignard at the turn of the 20th century, alkylmagnesium halides have become ubiquitous organometallic reagents, typically serving as carbanion equivalents.^1,2^ For example, Grignard reagents readily react with carbonyl moieties to afford secondary and tertiary alcohols (Scheme 1a).^3^ Grignard reagents also participate in cross-coupling (XC) reactions, once again serving as carbanion equivalents (Scheme 1b).^4,5^ Based on the structure of the Grignard reagent and the electronegativity difference of the Mg–X bond, it is plausible that Grignard reagents could also serve as halide nucleophiles. For example, subjecting epoxides to Grignard reagents can result in the formation of chlorohydrins.^6,7^ Recently, in the context of development of a cross-electrophile coupling (XEC) reaction of mesylates, our laboratory has demonstrated that, in addition to the anticipated role of reducing the nickel catalyst, methylmagnesium iodide also serves as a nucleophilic iodide source (Scheme 1c).^8^

Alkyl halides are versatile reagents in synthetic chemistry, most commonly employed in the alkylation of enolates and as starting materials for XC and XEC reactions, as well as the synthesis of Wittig and alkylmetal reagents.^9−13^ Therefore, we sought to develop new halogenation reactions that start from alkyl alcohols. Numerous methods have been established to transform alcohols into alkyl halides, in general, by coupling alcohol activation with a nucleophilic halide source.^14,15^ For example, the Appel reaction, which converts alcohols to iodides with PPh_3_ and I_2_, is a robust method employed by synthetic organic chemists.^16^ In this manuscript, we report a halogenation reaction with methylmagnesium iodide and bromide for the rapid synthesis of alkyl iodides and bromides (Scheme 1d). These reactions showcase an unusual reactivity mode of Grignard reagents and are stereospecific and scalable.

## Results and Discussion

We began by optimizing the iodination reaction of alkyl mesylates utilizing mesylate **1** as a model substrate (Table 1). This secondary mesylate was chosen due to the low volatility of the corresponding iodide, allowing for the ease of isolation and analysis of conversion. By performing the iodination reaction with MeMgI at room temperature for 1 h,^8^ iodide **2** was observed in an 81% yield (entry 1). Excitingly, this reaction demonstrates minimal amounts of elimination products. In an effort to minimize the formation of alkenes, we performed the reaction at 0 °C and observed decreased yields of alkenes **3** with no effect on the yield of iodide **2** (entry 2). In an attempt to further reduce the formation of the elimination products, the reaction temperature was lowered to −78 °C. Although we observed a decreased formation of the elimination products, the conversion of mesylate **1** also decreased (entry 3). However, the yield of iodide **2** was not affected, which would prove useful for substrates with functional groups that are sensitive to Grignard reagents (vide infra). We hypothesized that at 0 °C, shortening the reaction time to 5 min could potentially minimize the formation of elimination products while maintaining the high conversion of mesylate. We were pleased to see that this afforded iodide **2** in a 94% yield with a minimal amount of the elimination product **3** (entry 4). While freshly prepared MeMgI provided the highest rates, employing commercially available MeMgI also provided good results (entries 5 and 6).

**Table 1:**
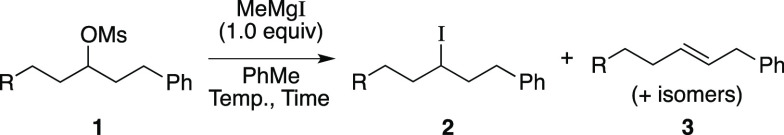
Optimization of Iodination Reaction^a^

entry	temp. (°C)	time	yield **2** (%)^b^	yield **3** (%)^b^	RSM **1** (%)^b^
1	25	1 h	81	13	<5
2	0	1 h	84	8	<5
3	–78	1 h	81	5	11
**4**	**0**	**5 m**	**92 (94)**^c^	**5(5)**^c^	**<5**
***Using Commercial MeMgI***:					
5	0	5 m	75	6	11
6	0	1 h	80 (78)^c^	7(7)^c^	5
***Using MgI***_***2***_***Instead of MeMgI:***					
7	25	5 m	27	<5	69
8^d^	25	5 m	69	6	20
9^d^	0	5 m	6	<1	81
***Using PhMgI***					
10^e^	0	5 m	54^c^	24^c^	<1

a R = (*p*-MeO)C_6_H_4_.

b Determined by ^1^H NMR based on comparison to PhTMS as an internal standard.

c Isolated yield.

d 30 μL of Et_2_O added.

e Ph-substituted product was isolated in a 14% yield.

We set out to distinguish whether the Grignard reagent itself, or MgI_2_, formed in situ via competitive Wurtz coupling during Grignard formation and the Schlenk equilibrium, serves as the iodide source for this reaction.^15,17,18^ To investigate the source of iodide, we subjected mesylate **1** to reactions with MgI_2_. We observed a decrease in yield compared to the standard reaction conditions employing the Grignard reagent (c.f. entries 4 and 7). Because the Grignard reagent is prepared in Et_2_O, and the low solubility of MgI_2_ in PhMe could account for the lower conversion of mesylate **1** to iodide **2** in entry 7, we performed the reaction with the addition of 30 μL of Et_2_O.^19^ Addition of Et_2_O did indeed increase the yield of iodide **2** (entry 8); however, the rate remained significantly slower than when MeMeI was employed. At 0 °C, the reaction with MgI_2_ and Et_2_O only afforded the desired iodide **2** in a 6% yield, with 81% of mesylate **1** observed (entry 9); in comparison, at the same temperature and reaction times, reactions employing the commercial and freshly prepared Grignard reagent provided 75% and 92% yields, respectively (entries 4 and 5). These results are consistent with a faster iodination reaction when MeMgI is used.^20^ We investigated if other Grignard reagents could be employed in the reaction to afford the alkyl iodide product from mesylate **1**. Employing PhMgI instead of MeMgI resulted in a decrease in yield, with increased elimination and S_N_2 substitution (entry 10).

**Scheme 2:**
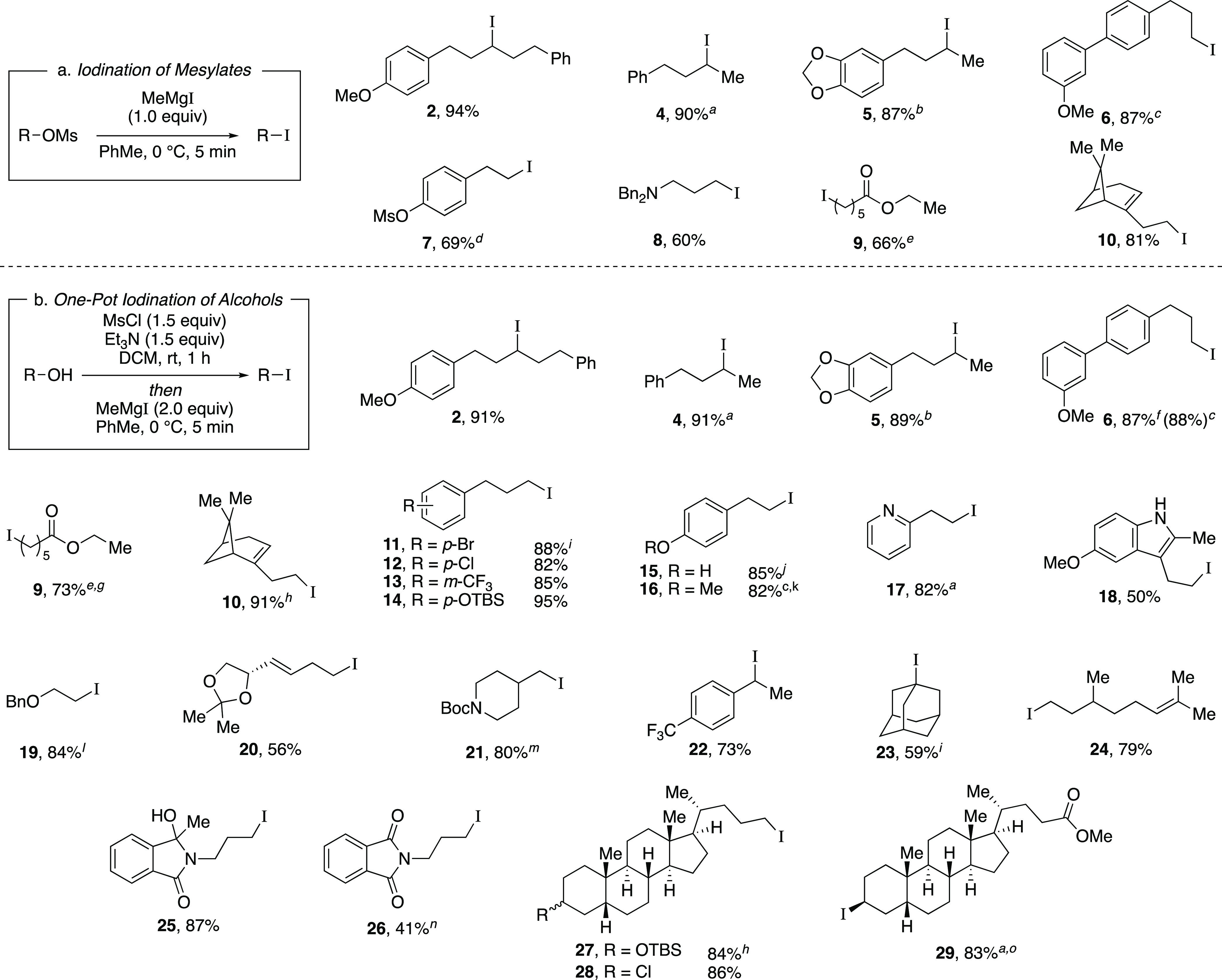
Iodination Reaction of Mesylates and One-Pot Reaction of Alcohols to Form Alkyl Iodides 1 h iodination reaction. ^b^30 min iodination reaction. ^c^Employing commercial MeMgI for 1 h. ^d^5.0 equiv MeMgI. ^e^–78 °C iodination reaction for 3 h. ^f^5 min mesylation. ^g^1.0 equiv MsCl, 1.0 equiv Et_3_N, 1.0 equiv MeMgI. ^h^3.0 equiv MeMgI. ^i^1 mmol scale. ^j^2.5 equiv MsCl, 2.5 equiv Et_3_N, 5 min mesylation, 2.5 equiv MeMgI, reaction followed by 1.0 equiv MeMgCl at rt for 1 h. ^k^30. mmol (4.6 g) scale. ^l^6 h mesylation, 2.5 equiv MeMgI, 2 h iodination reaction. ^m^4 h iodination reaction. ^n^–78 °C iodination reaction. ^o^0.4 mmol scale.

With optimized reaction conditions in hand, we evaluated the functional group compatibility of the iodination reaction (Scheme 2a). Both secondary and primary alkyl mesylates provided the corresponding alkyl iodides **4**–**6** in excellent yields. As one would predict from an S_N_2 mechanism, when a substrate bearing both an aryl mesylate and alkyl mesylate was subjected to the standard reaction conditions, iodination occurred at the alkyl mesylate to afford iodide **7**. Next, we explored functional groups that are typically sensitive to nucleophilic Grignard reagents. Both a dibenzylated amine and an ester provided the desired iodides **8** and **9**, respectively, when modified reaction conditions were utilized. Finally, the terpenol (*R*)-nopol cleanly underwent the iodination reaction to afford **10** in an 81% yield, comparing favorably to literature methods for the preparation of this compound.^21^

Next, we envisioned a one-pot protocol where the alcohol could be directly transformed into the iodide by in situ mesylation followed by iodination with MeMgI (Scheme 2b).^22^ A variety of secondary and primary alcohols underwent the one-pot reaction to give corresponding iodides in very good yields. We were pleased to observe that electron-donating groups (**6**, **11**–**12, 14–16**), an electron-withdrawing group (**13**), and heteroaryl groups (**17–18**) were tolerated to provide the desired alkyl iodides in great yields. Excitingly, we observed that common protecting groups—silyl ether, benzyl ether, acetal, and carbamate —were also tolerated in the one-pot reaction (**14**, **19**–**21**). We observed that 2-(4-hydroxyphenyl) ethanol provided the phenol-substituted alkyl iodide **15** in an 85% yield, via mesylation, iodination, and in situ deprotection of the phenol.^23^ Next, we investigated a benzylic alcohol substrate and observed the desired iodide **22** in moderate yields with a small amount of benzylic chloride as a byproduct. We anticipate that this benzylic chloride forms under the mesylation reaction conditions.^24^ Tertiary alcohols proved difficult for this reaction, resulting in the formation of high yields of elimination products; however, under the one-pot reaction conditions, iodide **23** could be obtained from 1-adamantanol in a 59% yield with a minor amount of alkyl chloride. Another terpenol, citronellol, was subjected to the one-pot iodination reaction to yield iodide **24** in moderate yield. Additionally, we evaluated a series of substrates bearing functional groups that are known to be sensitive to Grignard reagents. For a substrate bearing a phthalimide, under the standard conditions at 0 °C, the Grignard reagent reacted with both phthalimide and alkyl mesylate functionalities to afford **25**. We were pleased to see that at −78 °C, selectivity slightly favored the reaction of the alkyl mesylate to afford **26**, which could be separated from undesired **25** and mesylate that were each observed in a <20% yield. Even more encouraging, an ester-containing substrate underwent the one-pot mesylation and iodination reaction at low temperature to provide **9** in a 73% yield. Finally, derivatives of lithocholic acid containing a secondary silyl ether, a secondary chloride, and a pendant ester all provided the desired product in good yields (**27**–**29**). In comparison to literature methods reported for the synthesis of the 23 known iodides in Scheme 2, the majority (15) of these reactions provided similar yields (within ∼10%) to those previously reported. Therefore, this reaction provides a new set of conditions for the formation of alkyl iodides, with the advantage that the reactions are rapid at low temperatures.

**Scheme 3:**
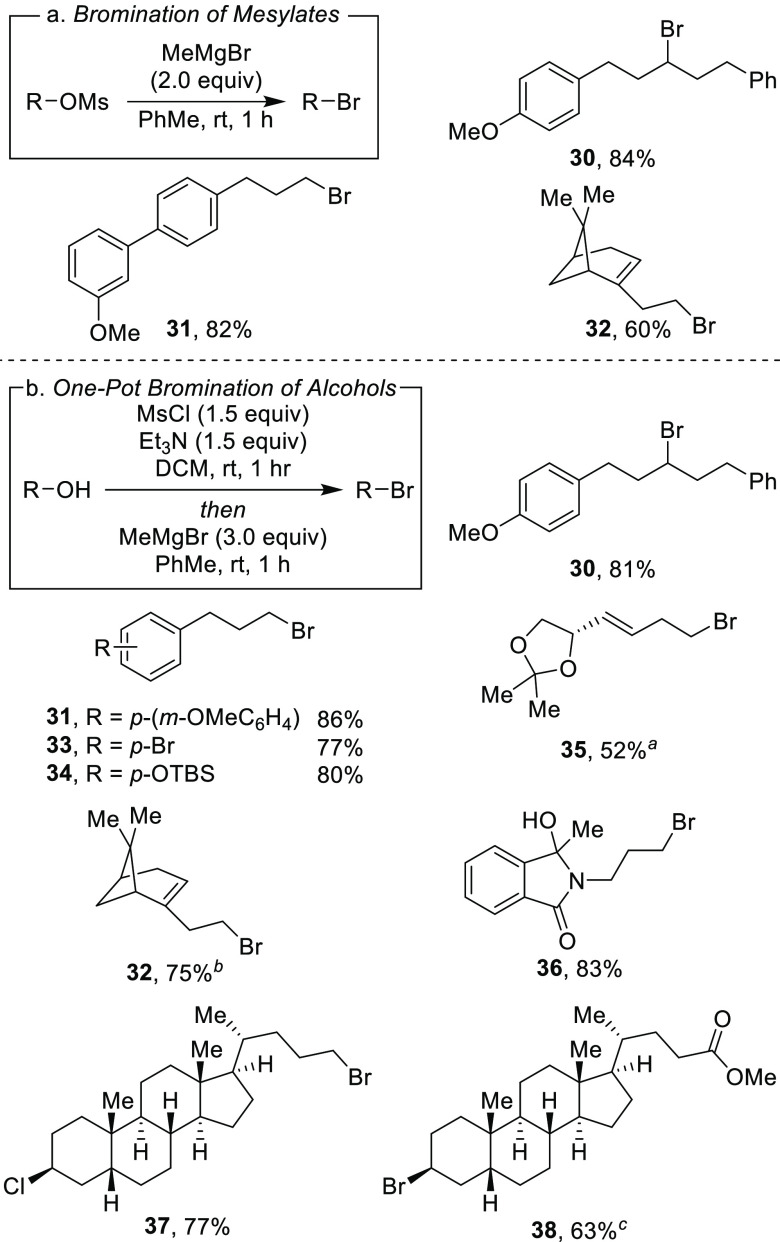
Bromination Reaction of Mesylates and One-Pot Reaction of Alcohols to Form Alkyl Bromides 0 °C bromination reaction. 4.0 equiv MeMgBr. 30 min mesylation, 2.0 equiv MeMgBr, 2 h bromination reaction at 0 °C.

With the success of the iodination reaction, we aimed to synthesize alkyl bromides through similar two-step and one-pot procedures (Scheme 3).^25^ We were pleased to see that employing MeMgBr in place of MeMgI afforded the desired alkyl bromides. Both secondary and primary bromides with pendant aryl substituents (**30–31, 33**–**34**) were synthesized in great yields. Similar to the iodination reaction, silyl ether and acetal protecting groups were tolerated in the reaction (**34**–**35**). An alkyl bromide (**32**) derived from chiral terpenol (*R*)-nopol was obtained in a 75% yield. A lithocholic acid derivative with a secondary alkyl chloride was also well tolerated to afford **37** in this one-pot reaction. Finally, an ester-containing lithocholic acid derivative provided secondary bromide **38** in moderate yield.

**Scheme 4:**
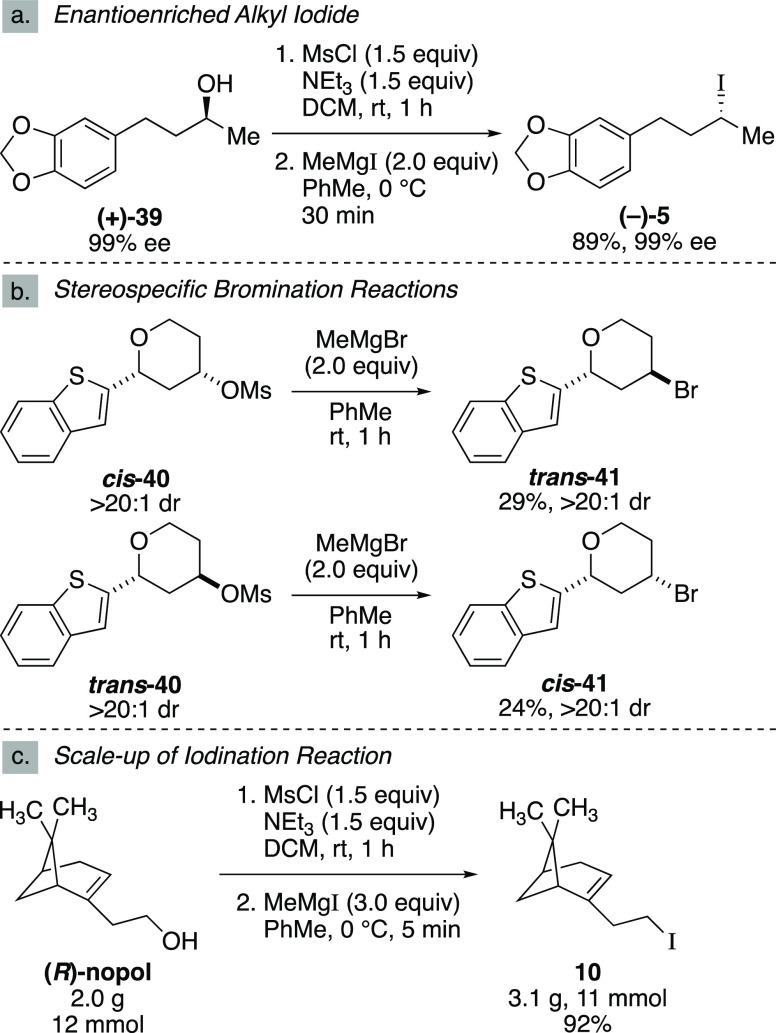
Stereospecificity and Scalability

Next, we turned our attention to the stereochemical outcome of the halogenation reaction. We hypothesized that a stereospecific S_N_2 reaction was operative and would proceed cleanly with inversion. To confirm this hypothesis, we prepared enantioenriched alcohol **39** via a lipase-catalyzed kinetic resolution^26^ and subjected it to the one-pot mesylation and iodination reaction (Scheme 4a). The reaction afforded enantioenriched alkyl iodide **5** in greater than 99% ee. To determine the stereochemical course of the reaction, we synthesized diastereomeric aryl-substituted 4-hydroxy tetrahydropyrans and investigated the outcome of the halogenation reaction. We subjected both *cis*- and *trans*-substituted tetrahydropyrans **40** to the bromination reaction (Scheme 4b). We observed that *cis*-**40** afforded *trans*-**41**, and similarly *trans*-**40** provided *cis*-**41**, both in >20:1 dr. These results demonstrated that the halogenation reaction proceeds with inversion. These results also corroborated that the substitution occurs with high stereochemical fidelity. Finally, we investigated the scalability of the reaction (Scheme 4c). When we performed the one-pot mesylation and iodination reaction of (*R*)-nopol on a two-gram scale, the desired alkyl iodide **10** was afforded in a 92% yield. Therefore, this reaction provides a reliable method for the preparative-scale synthesis of alkyl iodides and bromides.

## Conclusions

In summary, we have developed a halogenation reaction to transform alkyl alcohols into alkyl iodides and bromides that employs Grignard reagents as nucleophilic halide sources. The reaction is compatible with substrates containing various functional groups, including some that are typically sensitive to Grignard reagents. Through various stereochemical experiments, we have demonstrated that the halogenation reaction occurs through a stereospecific S_N_2 reaction, proceeding with inversion. This reaction was also effective on the gram scale, which supports its synthetic utility. Most importantly, this work clearly establishes that methyl Grignard reagents can act as halide nucleophiles as well as carbanion equivalents.

## Experimental Section

### General Procedures

All reactions were carried out under an atmosphere of N_2_ when noted. All glassware was oven- or flame-dried prior to use. Tetrahydrofuran (THF), diethyl ether (Et_2_O), dichloromethane (DCM), dimethylformamide (DMF), and toluene (PhMe) were degassed with Ar and then passed through two 4 × 36 inch columns of anhydrous neutral A-2 alumina (8 × 14 mesh; LaRoche Chemicals; activated under a flow of argon at 350 °C for 12 h) to remove H_2_O.^27^ All other solvents utilized were purchased “anhydrous” commercially or purified as described. ^1^H NMR spectra were recorded on Bruker DRX-400 (400 MHz ^1^H, 100 MHz ^13^C, 376.5 MHz ^19^F), CRYO-500 (500 MHz ^1^H, 125.8 MHz ^13^C), or AVANCE-600 (600 MHz ^1^H, 150 MHz ^13^C, 564.7 MHz ^19^F) spectrometers. Proton chemical shifts are reported in ppm (δ) relative to internal tetramethylsilane (TMS, δ 0.00). Data is reported as follows: chemical shift (multiplicity [singlet (s), broad singlet (br s), doublet (d), doublet of doublets (dd), doublet of doublet of doublets (ddd), doublet of doublet of doublet of doublets (dddd), triplet of doublets (td), doublet of triplet of doublets (dtd), quartet of doublets (qd), triplet (t), doublet of triplets (dt), doublet of doublet of triplets (ddt), triplet of triplets (tt), quartet (q), triplet of quartets (tq), quintet (quint), sextet (sext), apparent singlet (as), apparent doublet (ad), apparent triplet (at), apparent quartet (aq), apparent quintet (aquint), apparent septet (asept), multiplet (m)], coupling constants [Hz], integration). Carbon chemical shifts are reported in ppm (δ) relative to TMS with the respective solvent resonance as the internal standard (CDCl_3_, δ 77.16 ppm). Fluorine chemical shifts are reported in ppm (δ) relative to the absolute frequency of 0.00 ppm in the proton spectrum. NMR data were collected at 25 °C. Structural assignments were made with additional information from gCOSY experiments. Infrared (IR) spectra were obtained on a Thermo Scientific Nicolet iS5 spectrometer with an iD5 ATR tip (neat) and are reported in terms of the frequency of absorption (cm^–1^). Analytical thin-layer chromatography (TLC) was performed using Silica Gel 60 F254 precoated plates (0.25 mm thickness). Visualization was accomplished by irradiation with a UV lamp and/or staining with cerium ammonium molybdate (CAM), phosphomolybdic acid (PMA), or potassium permanganate (KMnO_4_) solutions. Flash chromatography was performed using a SiliaFlash P60 (40–63 μm, 60 Å) from SiliCycle. Melting points (m.p.) were obtained using a MelTemp melting point apparatus and are uncorrected. Optical rotations were measured on a Rudolph Research Analytical Autopol III Automatic Polarimeter. SFC determinations of enantiopurity were determined by chiral SFC analysis and performed on Agilent Technologies HPLC (1260 series) system AD Chiralpak columns (100 bar, 50 °C, 254 nm). High-resolution mass spectrometry was performed by the University of California, Irvine Mass Spectrometry Center.

### Reagents

Methylmagnesium iodide was titrated with iodine prior to use.^28^ All other chemicals were purchased commercially and used as received unless otherwise noted.

### General Procedures for the Synthesis of Iodides and Bromides

#### Method A: Mesylation

A flame-dried round-bottom flask equipped with a stir bar was charged with alcohol (1.0 equiv) and DCM (0.20 M in alcohol) under N_2_. Et_3_N (1.5 equiv) and DMAP (0–0.2 equiv) were added, and the reaction mixture was allowed to stir for 5 min. Then, MsCl (1.5 equiv) was added, and the reaction mixture was allowed to stir at rt for 1–16 h. Once complete by TLC, sat. NaHCO_3_ (5 mL) was added, and the reaction mixture was extracted with DCM (3 × 10 mL). The combined organic layers were washed with brine, dried over Na_2_SO_4_, and concentrated in vacuo.

#### Preparation of MeMgI

Under a N_2_ atmosphere, a three-neck round-bottom flask equipped with a stir bar, a reflux condenser, and a Schlenk filtration apparatus was charged with magnesium turnings (4.3 g, 180 mmol, 1.5 equiv). The flask and magnesium turnings were placed under vacuum and flame-dried and then back-filled with N_2_. A crystal of iodine (ca. 2 mg) was added to the flask, followed by anhydrous Et_2_O (30 mL). Freshly distilled iodomethane (7.5 mL, 120 mmol, 1.0 equiv) was added dropwise until the reaction initiated, and then the reaction mixture was cooled to 0 °C and the remaining iodomethane was added slowly over 30 min to maintain a gentle reflux. The mixture was stirred for 2 h at rt and then filtered through the fritted Schlenk filter into a pear-shaped flask under a N_2_ atmosphere. The pear-shaped flask was capped with a septum, sealed with parafilm, and stored either in the glovebox under a N_2_ atmosphere for up to 8 weeks or in a −20 °C freezer for up to 4 weeks. The resulting methyl Grignard reagent was typically between 2.9 and 3.1 M, as titrated by Knochel’s method.^28^

#### Method B: Iodination Reaction of Mesylates

Under a N_2_ atmosphere, a flame-dried round-bottom flask equipped with a stir bar was charged with mesylate substrate (1.0 equiv) and PhMe (0.10 M in mesylate). The reaction mixture was cooled to 0 °C, and then MeMgI (1.0 equiv, 2.4–3.2 M in Et_2_O) was added dropwise. The reaction mixture was allowed to stir for 5 min. If commercial MeMgI was employed, then the reaction mixture was allowed to stir for 1 h instead. The reaction mixture was warmed to rt for 5 min. MeOH was added dropwise to quench the reaction, and then the mixture was filtered through a plug of silica gel eluting with Et_2_O and concentrated in vacuo. The reaction mixture was purified by column chromatography. For the optimization reactions, phenyltrimethylsilane (PhTMS; 8.6 μL, 50 μmol) was added before purification and the yield was determined by ^1^H NMR based on comparison to PhTMS as the internal standard.

#### Method C: One-Pot Reaction of Alcohols to Form Iodides

A flame-dried round-bottom flask equipped with a stir bar was charged with alcohol (1.0 equiv) and DCM (0.20 M in alcohol) under N_2_. Et_3_N (1.5 equiv) was added, and the reaction mixture was allowed to stir for 5 min. Then, MsCl (1.5 equiv) was added, and the reaction mixture was allowed to stir at rt for 1 h. PhMe (0.20 M in alcohol) was added, the reaction mixture was cooled to 0 °C, and then MeMgI (2.0 equiv, 2.4–3.2 M in Et_2_O) was added dropwise. The reaction mixture was allowed to stir at 0 °C for 5 min. If commercial MeMgI was employed, the reaction mixture was allowed to stir for 1 h. Then, the reaction mixture was warmed to rt for 5 min. MeOH was added dropwise to quench the reaction, and then the mixture was filtered through a plug of silica gel eluting with Et_2_O and concentrated in vacuo. The reaction mixture was purified by column chromatography. If the reaction scale was 0.40 mmol or greater, then an extraction workup was carried out, instead of the silica gel plug, as follows. After warming up the reaction for 5 min, sat. aqueous NH_4_Cl was added dropwise. The layers were separated, and then the aqueous layer was extracted with DCM (×3). The organic layers were combined, dried over Na_2_SO_4_, and concentrated in vacuo. For the optimization reactions, phenyltrimethylsilane (PhTMS; 8.6 μL, 50. μmol) was added before purification and the yield was determined by ^1^H NMR based on comparison to PhTMS as the internal standard.

#### Method D: Bromination Reaction of Mesylates

Under a N_2_ atmosphere, a flame-dried round-bottom flask equipped with a stir bar was charged with mesylate substrate (1.0 equiv) and PhMe (0.10 M in mesylate). Then, commercial MeMgBr (2.0 equiv, 2.7–3.0 M in Et_2_O) was added dropwise, and the reaction mixture was allowed to stir at rt for 1 h. MeOH was added dropwise to quench the reaction, and then the mixture was filtered through a plug of silica gel eluting with Et_2_O and concentrated in vacuo. The reaction mixture was purified by column chromatography. For the optimization reactions, phenyltrimethylsilane (PhTMS; 8.6 μL, 50. μmol) was added before purification, and the yield was determined by ^1^H NMR based on comparison to PhTMS as the internal standard.

#### Method E: One-Pot Reaction of Alcohols to Form Bromides

A flame-dried round-bottom flask equipped with a stir bar was charged with alcohol (1.0 equiv) and DCM (0.20 M in alcohol) under N_2_. Et_3_N (1.5 equiv) was added, and the reaction mixture was allowed to stir for 5 min. Then, MsCl (1.5 equiv) was added, and the reaction mixture was allowed to stir at rt for 1 h. PhMe (0.20 M in alcohol) was added, and then commercial MeMgBr (3.0 equiv, 2.7–3.0 M in Et_2_O) was added dropwise. The reaction mixture was allowed to stir for 1 h at rt. MeOH was added dropwise to quench the reaction, and then the mixture was filtered through a plug of silica gel eluting with Et_2_O and concentrated in vacuo. The reaction mixture was purified by column chromatography.

#### Characterization Data for Alcohols, Mesylates, Iodides, and Bromides

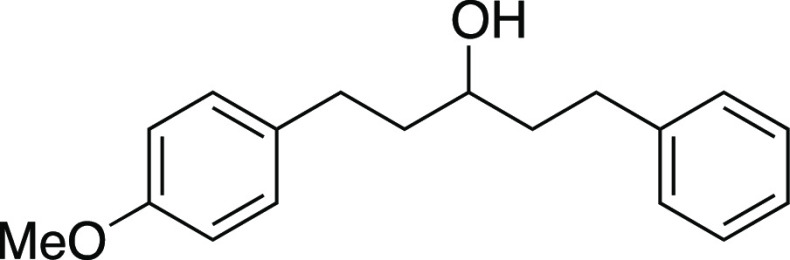



Alcohol **42** was prepared according to the following procedure. A flame-dried round-bottom flask with a stir bar was charged with Grignard **SI-3** (5.3 mL, 8.3 mmol, 1.1 equiv, 1.6 M in Et_2_O) and cooled to 0 °C. A solution of aldehyde **SI-2** (1.2 g, 7.5 mmol, 1.0 equiv) in anhydrous THF (38 mL, 0.20 M in substrate) was added dropwise. The reaction mixture was allowed to stir at rt for at least 2 h. The reaction was quenched with sat. aqueous NH_4_Cl (10 mL), and the mixture was extracted with Et_2_O (3 × 20 mL). The combined organic layers were washed with brine, dried over Na_2_SO_4_, and concentrated in vacuo. The residue was purified by column chromatography (0–25% EtOAc/hexanes) to afford the title compound as a white solid (1.5 g, 5.4 mmol, 72% yield). **m.p.** 77–78 °C; **TLC R**_**f**_ = 0.5 (25% EtOAc/hexanes, CAM stain); ^**1**^**H NMR** (400 MHz, CDCl_3_) δ 7.31–7.26 (m, 2H), 7.26–7.16 (m, 3H), 7.10 (d, *J* = 8.6 Hz, 2H), 6.83 (d, *J* = 8.6 Hz, 2H), 3.89 (s, 3H), 3.66 (m, 1H), 2.83–2.57 (m, 4H), 1.88–1.69 (m, 4H), 1.34 (d, *J* = 5.2 Hz, 1H). Analytical data is consistent with literature values.^29^
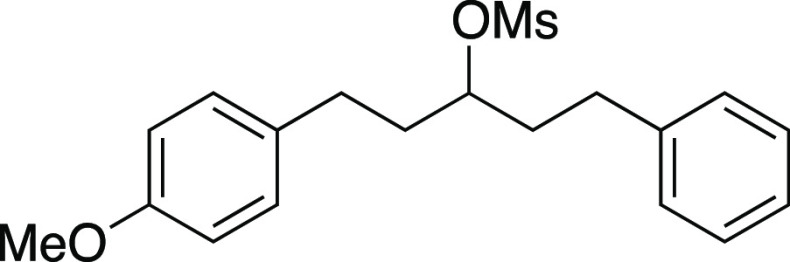


Mesylate **1** was prepared according to Method A. The following amounts of reagents were used: alcohol **42** (0.27 g, 1.0 mmol, 1.0 equiv), MsCl (0.12 mL, 1.5 mmol, 1.5 equiv), triethylamine (0.21 mL, 1.5 mmol, 1.5 equiv), and DCM (5.0 mL, 0.20 M in substrate). The reaction mixture was allowed to stir overnight. The residue was purified by column chromatography (0–25% EtOAc/hexanes) to afford the title compound as a colorless oil (0.32 g, 0.93 mmol, 93% yield). **TLC R**_**f**_ = 0.6 (25% EtOAc/hexanes, CAM stain); ^**1**^**H NMR** (400 MHz, CDCl_3_) δ 7.29 (t, *J* = 7.4 Hz, 2H), 7.23–7.15 (m, 3H), 7.09 (d, *J* = 8.6 Hz, 2H), 6.83 (d, *J* = 8.6 Hz, 2H), 4.80 (quint, *J* = 6.0 Hz, 1H), 3.78 (s, 3H), 2.98 (s, 3H), 2.81–2.60 (m, 4H), 2.15–1.95 (m, 4H); ^**13**^**C{**^**1**^**H} NMR** (125.8 MHz, CDCl_3_) δ 158.1, 140.9, 132.8, 129.4 (2C), 128.7 (2C), 128.4 (2C), 126.3, 114.0 (2C), 82.6, 33.4, 38.8, 36.5, 36.3, 31.3, 30.4; **IR** (neat) 2935, 1611, 1512, 1330, 1244, 1169, 1033, 897, 700 cm^–1^; **HRMS** (TOF MS ES+) *m*/*z*: [M + Na]^+^ calcd for C_19_H_24_O_4_SNa, 371.1293; found, 371.1276.
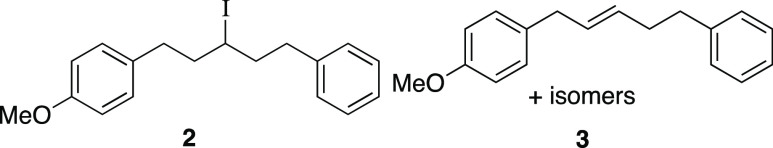


##### From Mesylate

Iodide **2** was prepared according to Method B. The following amounts of reagents were used: mesylate **1** (33 mg, 94 μmol, 1.0 equiv), MeMgI (32 μL, 94 μmol, 1.0 equiv, 2.9 M in Et_2_O), and PhMe (0.94 mL, 0.10 M in substrate). The residue was purified by column chromatography (0–5% EtOAc/hexanes) to afford the title compound as a yellow oil (33 mg, 88 μmol, 94% yield) containing alkenes **3** (1 mg, 5 μmol, 5% yield). Refer to iodide **2** below for analytical data.

##### From Alcohol

Iodide **2** was prepared according to Method C. The following amounts of reagents were used: alcohol **42** (30. mg, 0.11 mmol, 1.0 equiv), MsCl (13 μL, 0.17 mmol, 1.5 equiv), Et_3_N (23 μL, 0.17 mmol, 1.5 equiv), DCM (0.55 mL, 0.20 M in substrate), MeMgI (76 μL, 0.22 mmol, 2.0 equiv, 2.9 M in Et_2_O), and PhMe (0.55 mL, 0.20 M in substrate). The residue was purified by column chromatography (0–5% EtOAc/hexanes) to afford the title compound as a yellow oil (38 mg, 0.10 mmol, 91% yield) containing alkene **3** (2 mg, 7 μmol, 6% yield). To remove the alkenes, a modified Sharpless asymmetric dihydroxylation was performed.^30^ To a flame-dried 20 mL vial was added AD-mix-β (0.15 g, 1.4 g/mmol). Then, t-BuOH (1.0 mL) and H_2_O (1.0 mL) were added via a syringe and the vial was capped. The vial was cooled to 0 °C, and then the mixture of iodide **2** and alkenes **3** was added dropwise as a solution in t-BuOH (1.0 mL) and H_2_O (1.0 mL) via a syringe. The mixture was allowed to stir at 0 °C for 24 h. To quench, Na_2_SO_3_ (30. mg) was added and the mixture was allowed to warm to rt and stir for 30 min. Then, the mixture was transferred to a separatory funnel, and the organic layer was extracted with EtOAc (×3). The combined organic layers were washed with brine, dried over Na_2_SO_4_, filtered, and concentrated in vacuo. The residue was purified by column chromatography (0–5% EtOAc/hexanes) to afford the title compound as a yellow oil (33 mg, 86 μmol, 78% yield over two steps). **TLC R**_**f**_ = 0.4 (5% EtOAc/hexanes, CAM stain); ^**1**^**H NMR** (400 MHz, CDCl_3_) δ 7.27 (t, *J* = 7.4 Hz, 2H), 7.22–7.14 (m, 3H), 7.08 (d, *J* = 8.5 Hz, 2H), 6.81 (d, *J* = 8.5 Hz, 2H), 4.00 (asept, *J* = 4.5 Hz, 1H), 3.77 (s, 3H), 2.84 (dddd, *J* = 23.3, 14.0, 9.3, 4.5 Hz, 2H), 2.67 (dddd, *J* = 20.6, 14.0, 9.0, 7.0 Hz, 2H), 2.18 (dtd, *J* = 23.5, 9.1, 5.2 Hz, 2H), 2.05–1.90 (m, 2H); ^**13**^**C{**^**1**^**H} NMR** (125.8 MHz, CDCl_3_) δ 158.1, 140.9, 132.9, 129.5 (2C), 128.60 (2C), 128.57 (2C), 126.2, 114.0 (2C), 55.3, 42.6, 42.4, 38.3, 35.7, 34.7; **IR** (neat) 2922, 1611, 1511, 1453, 1245, 1177, 1036, 825, 747, 699 cm^–1^; **HRMS** (TOF MS CI+) *m*/*z*: [M]^+^ calcd for C_18_H_21_IO, 380.0637; found, 380.0627.
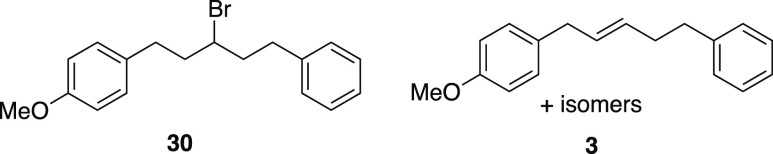


##### From Mesylate

Bromide **30** was prepared according to Method D. The following amounts of reagents were used: mesylate **1** (35 mg, 0.10 mmol, 1.0 equiv), MeMgBr (67 μL, 0.20 mmol, 2.0 equiv, 3.0 M in Et_2_O), and PhMe (1.0 mL, 0.10 M in substrate). The residue was purified by column chromatography (0–5% EtOAc/hexanes) to afford the title compound as a pale-yellow oil (28 mg, 84 μmol, 84% yield) containing alkenes **3** (2.3 mg, 9.1 μmol, 9.0% yield). To remove the alkenes, a modified Sharpless asymmetric dihydroxylation was performed.^30^ To a flame-dried 20 mL vial was added AD-mix-β (0.14 g, 1.4 g/mmol). Then, *t*-BuOH (1.0 mL) and H_2_O (1.0 mL) were added via a syringe and the vial was capped. The vial was cooled to 0 °C, and then the mixture of bromide **30** and alkenes **3** was added dropwise as a solution in *t*-BuOH (1.0 mL) and H_2_O (1.0 mL) via a syringe. The mixture was allowed to stir at 0 °C for 24 h. To quench, Na_2_SO_3_ (30. mg) was added and the mixture was allowed to warm to rt and stir for 30 min. Then, the mixture was transferred to a separatory funnel, and the organic layer was extracted with EtOAc (×3). The combined organic layers were washed with brine, dried over Na_2_SO_4_, filtered, and concentrated in vacuo. The residue was purified by column chromatography (0–5% EtOAc/hexanes) to afford the title compound as a yellow oil (29 mg, 87 μmol, 87% yield over two steps). **TLC R**_**f**_ = 0.3 (5% EtOAc/hexanes, CAM stain); ^**1**^**H NMR** (400 MHz, CDCl_3_) δ 7.27 (t, *J* = 7.5 Hz, 2H), 7.21–7.13 (m, 3H), 7.08 (d, *J* = 8.5 Hz, 2H), 6.81 (d, *J* = 8.5 Hz, 2H), 3.94 (asept, *J* = 4.4 Hz, 1H), 3.78 (s, 3H), 2.85 (dddd, *J* = 30.2, 14.0, 9.0, 5.1 Hz, 2H), 2.76–2.63 (m, 2H), 2.20–2.00 (m, 4H); ^**13**^**C{**^**1**^**H} NMR** (125.8 MHz, CDCl_3_) δ 158.0, 141.1, 133.0, 129.5 (2C), 128.59 (2C), 128.56 (2C), 126.2, 114.0 (2C), 56.8, 55.3, 41.2, 41.0, 33.8, 32.9; **IR** (neat) 2927, 1611, 1512, 1454, 1246, 1177, 1036, 825, 748, 700 cm^–1^; **HRMS** (TOF MS CI+) *m*/*z*: [M]^+^ calcd for C_18_H_21_BrO, 332.0776; found, 332.0767.

##### From Alcohol

Bromide **30** was prepared according to Method E. The following amounts of reagents were used: alcohol **42** (27 mg, 0.10 mmol, 1.0 equiv), MsCl (12 μL, 0.15 mmol, 1.5 equiv), Et_3_N (21 μL, 0.15 mmol, 1.5 equiv), DCM (0.50 mL, 0.20 M in substrate), MeMgBr (0.11 mL, 0.30 mmol, 3.0 equiv, 2.7 M in Et_2_O), and PhMe (0.50 mL, 0.20 M in substrate). The residue was purified by column chromatography (0–5% EtOAc/hexanes) to afford the title compound as a pale-yellow oil (31 mg, 81 μmol, 81% yield) containing alkene **3** (2.8 mg, 11 μmol, 11% yield). Refer to bromide **30** above for analytical data.
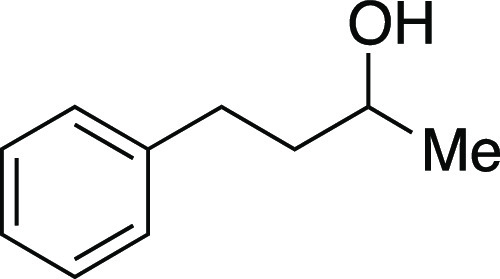


Alcohol **43** was prepared according to the following procedure. Open to air, a round-bottom flask with a stir bar was charged with 4-phenyl-2-butanone (0.75 mL, 5.0 mmol, 1.0 equiv), NaBH_4_ (0.38 g, 10. mmol, 2.0 equiv), and MeOH (25 mL, 0.20 M in substrate). The reaction mixture was stirred for 2 h. After completion, the reaction mixture was concentrated in vacuo and then dissolved in DCM. H_2_O was added, and the aqueous layer was extracted with DCM. The combined organic layers were dried and concentrated in vacuo. The residue was purified by flash column chromatography (20% EtOAc/hexanes) to afford the title compound as a colorless oil (710 mg, 4.8 mmol, 95%). **TLC R**_**f**_ = 0.5 (25% EtOAc/hexanes, KMnO_4_ stain); ^**1**^**H NMR** (400 MHz, CDCl_3_) δ 7.30–7.27 (m, 2H), 7.21–7.17 (m, 3H), 3.85–3.81 (m, 1H), 2.80–2.64 (m, 2H), 1.81–1.74 (m, 2H), 1.32 (br s, 1H), 1.23 (d, *J* = 6.2 Hz, 3H). Analytical data is consistent with literature values.^31^
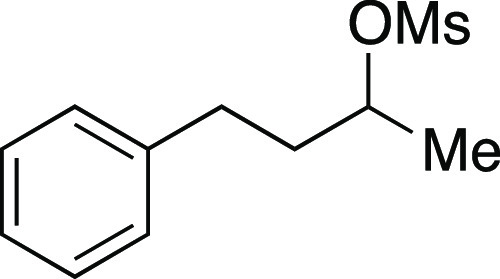


Mesylate **44** was prepared according to Method A. The following amounts of reagents were used: alcohol **43** (300 mg, 2.0 mmol, 1.0 equiv), MsCl (0.23 mL, 3.0 mmol, 1.5 equiv), Et_3_N (0.42 mL, 3.0 mmol, 1.5 equiv), DMAP (24 mg, 0.20 mmol, 0.10 equiv), and DCM (10. mL, 0.20 M in substrate). The reaction mixture was allowed to stir overnight. The residue was purified by flash column chromatography (0–20% EtOAc/hexanes) to afford the title compound as a pale-yellow oil (420 mg, 1.8 mmol, 92%). **TLC R**_**f**_ = 0.4 (20% EtOAc/hexanes, CAM stain); ^**1**^**H NMR** (400 MHz, CDCl_3_) δ 7.27–7.23 (m, 2H), 7.17–7.14 (m, 3H), 4.76 (tq, *J* = 6.4, 6.1 Hz, 1H), 2.88 (s, 3H), 2.75–2.60 (m, 2H), 2.03–1.94 (m, 1H), 1.90–1.81 (m, 1H), 1.39 (d, *J* = 6.3 Hz, 3H); ^**13**^**C{**^**1**^**H} NMR** (100 MHz, CDCl_3_) δ 140.6, 128.3 (2C), 128.1 (2C), 125.9, 79.3, 38.2, 37.9, 31.1, 20.9; **HRMS** (TOF MS ES+) *m*/*z*: [M + Na]^+^ calculated for C_11_H_16_O_3_SNa, 251.0718, found 251.0724. Analytical data is consistent with literature values.^32^
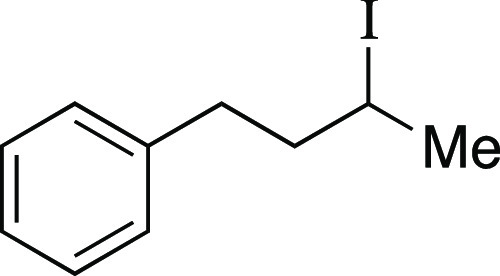


##### From Mesylate

Iodide **4** was prepared according to a modified Method B. The following amounts of reagents were used: mesylate **44** (29 mg, 0.13 mmol, 1.0 equiv), MeMgI (44 μL, 0.13 mmol, 1.0 equiv, 2.9 M in Et_2_O), and PhMe (1.3 mL, 0.10 M in substrate). The reaction mixture was allowed to stir at 0 °C for 1 h. The residue was purified by flash column chromatography (0–10% Et_2_O/hexanes, CAM stain) to afford the title compound as a colorless oil (30. mg, 0.12 mmol, 90%). **TLC R**_**f**_ = 0.7 (5% EtOAc/hexanes, CAM stain); ^**1**^**H NMR** (400 MHz, CDCl_3_) δ 7.30–7.24 (m, 2H), 7.21–7.18 (m, 3H), 4.15–4.07 (m, 1H), 2.88–2.81 (m, 1H), 2.73–2.65 (m, 1H), 2.19–2.10 (m, 1H), 1.94 (d, *J* = 6.8 Hz, 3H), 1.92–1.83 (m, 1H). Analytical data is consistent with literature values.^33^

##### From Alcohol

Iodide **4** was prepared according to a modified Method C. The following amounts of reagents were used: alcohol **43** (18 mg, 0.12 mmol, 1.0 equiv), MsCl (14 μL, 0.18 mmol, 1.5 equiv), Et_3_N (26 μL, 0.18 mmol, 1.5 equiv), DCM (0.62 mL, 0.20 M in substrate), followed by MeMgI (84 μL, 0.25 mmol, 2.0 equiv, 2.9 M in Et_2_O) and PhMe (0.62 mL, 0.20 M in substrate). Upon the addition of MeMgI, the reaction mixture was allowed to stir at 0 °C for 1 h. The residue was purified by flash column chromatography (0–10% Et_2_O/hexanes) to afford the title compound as a colorless oil (29 mg, 0.11 mmol, 91%). Refer to iodide **4** above for analytical data.
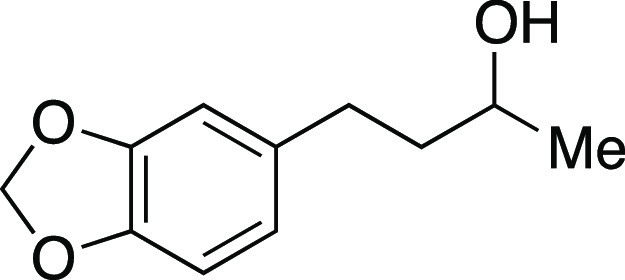


Alcohol **39** was prepared according to the following procedure. A flame-dried round-bottom flask with a stir bar was charged with aldehyde **SI-4** (0.78 g, 4.4 mmol, 1.0 equiv) and anhydrous THF (25 mL, 0.20 M in substrate) and cooled to 0 °C. Then, MeMgCl (2.2 mL, 6.6 mmol, 1.5 equiv) was added dropwise. The reaction mixture was allowed to stir at rt overnight. The reaction was quenched with sat. aqueous NH_4_Cl (10 mL), and the mixture was extracted with Et_2_O (3 ×20 mL). The combined organic layers were washed with brine, dried over Na_2_SO_4_, and concentrated in vacuo. The residue was purified by flash column chromatography (0–30% EtOAc/hexanes) to afford the title compound as a pale-yellow oil (0.81 g, 4.2 mmol, 95%). **TLC R**_**f**_ = 0.4 (30% EtOAc/hexanes); ^**1**^**H NMR** (400 MHz, CDCl_3_) δ 6.72 (d, *J* = 7.9 Hz, 1H), 6.69 (s, 1H), 6.65 (d, *J* = 7.9 Hz, 1H), 5.91 (s, 2H), 3.81 (br s, 1H), 2.71–2.56 (m, 2H), 1.75–1.69 (m, 2H), 1.32 (br s, 1H), 1.22 (d, *J* = 6.2 Hz, 3H); **SFC analysis** (Chiralcel AD, 1% IPA, 2.0 mL/min, 230 nm) indicated 0% ee: t_R_ (minor enantiomer) = 37.0 min, t_R_ (major enantiomer) = 39.5 min. Analytical data is consistent with literature values.^34^
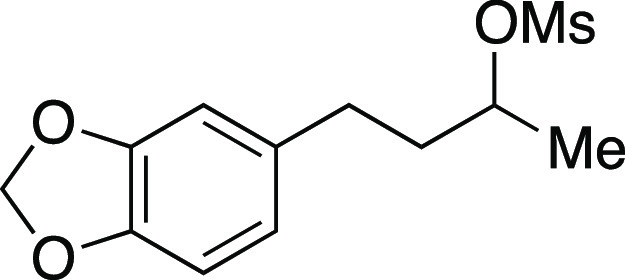


Mesylate **45** was prepared according to Method A. The following amounts of reagents were used: alcohol **39** (0.14 g, 0.70 mmol, 1.0 equiv), MsCl (80. μL, 1.1 mmol, 1.5 equiv), Et_3_N (0.15 mL, 1.1 mmol, 1.5 equiv), and DCM (3.5 mL, 0.20 M in substrate). The reaction mixture was allowed to stir overnight. The residue was purified by flash column chromatography (0–30% EtOAc/hexanes) to afford the title compound as a pale-yellow oil (0.18 g, 0.67 mmol, 96%). **TLC R**_**f**_ = 0.4 (30% EtOAc/hexanes); ^**1**^**H NMR** (400 MHz, CDCl_3_) δ 6.71 (d, *J* = 7.8 Hz, 1H), 6.67 (s, 1H), 6.63 (d, *J* = 7.8 Hz, 1H), 5.89 (s, 2H), 4.83–4.77 (m, 1H), 2.98 (s, 3H), 2.69–2.56 (m, 2H), 2.02–1.94 (m, 1H), 1.90–1.83 (m, 1H), 1.43 (d, *J* = 6.2 Hz, 3H); ^**13**^**C{**^**1**^**H} NMR** (100 MHz, CDCl_3_) δ 147.6, 145.8, 134.5, 121.0, 108.7, 108.2, 100.8, 79.3, 38.6, 38.5, 31.0, 21.1; **HRMS** (TOF MS ES+) *m*/*z*: [M + Na]^+^ calculated for C_12_H_16_O_5_SNa 295.0616, found 295.0605. Analytical data is consistent with literature values.^32^
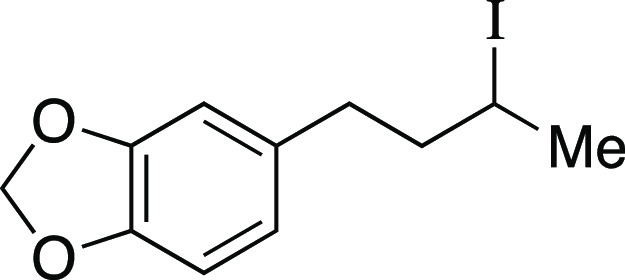


##### From Mesylate

Iodide **5** was prepared according to a modified Method B. The following amounts of reagents were used: mesylate **45** (20. mg, 73 μmol, 1.0 equiv), MeMgI (25 μL, 0.10 mmol, 1.0 equiv, 2.9 M in Et_2_O), and PhMe (0.73 mL, 0.10 M in substrate). The reaction mixture was allowed to stir at 0 °C for 30 min. The residue was purified by flash column chromatography (0–10% Et_2_O/hexanes) to afford the title compound as a pale-yellow oil (19 mg, 63 μmol, 87%). **TLC R**_**f**_ = 0.5 (10% EtOAc/hexanes); ^**1**^**H NMR** (600 MHz, CDCl_3_) δ 6.73 (d, *J* = 7.9 Hz, 1H), 6.69 (s, 1H), 6.66 (d, *J* = 7.9 Hz, 1H), 5.92 (s, 2H), 4.12–4.06 (m, 1H), 2.78–2.73 (m, 1H), 2.64–2.59 (m, 1H), 2.13–2.07 (m, 1H), 1.94 (d, *J* = 6.8 Hz, 3H), 1.85–1.79 (m, 1H); **SFC analysis** (Chiralcel AD, 1% IPA, 2.0 mL/min, 230 nm) indicated 0% ee: t_R_ (major enantiomer) = 8.5 min, t_R_ (minor enantiomer) = 9.4 min. Analytical data is consistent with literature values.^35^

##### From Alcohol

Iodide **5** was prepared according to a modified Method C. The following amounts of reagents were used: alcohol **39** (22 mg, 0.11 mmol, 1.0 equiv), MsCl (13 μL, 0.17 mmol, 1.5 equiv), Et_3_N (23 μL, 0.17 mmol, 1.5 equiv), DCM (0.56 mL, 0.20 M in substrate), followed by MeMgI (75 μL, 0.22 mmol, 2.0 equiv, 2.9 M in Et_2_O) and PhMe (0.56 mL, 0.20 M in substrate). Upon the addition of MeMgI, the reaction mixture was allowed to stir at 0 °C for 30 min. The residue was purified by flash column chromatography (0–10% Et_2_O/hexanes) to afford the title compound as a pale-yellow oil (30. mg, 99 μmol, 89%). Refer to iodide **5** above for analytical data. **SFC analysis** (Chiralcel AD, 1% IPA, 2.0 mL/min, 230 nm) indicated 0% ee: t_R_ (major enantiomer) = 9.1 min, t_R_ (minor enantiomer) = 10.1 min.
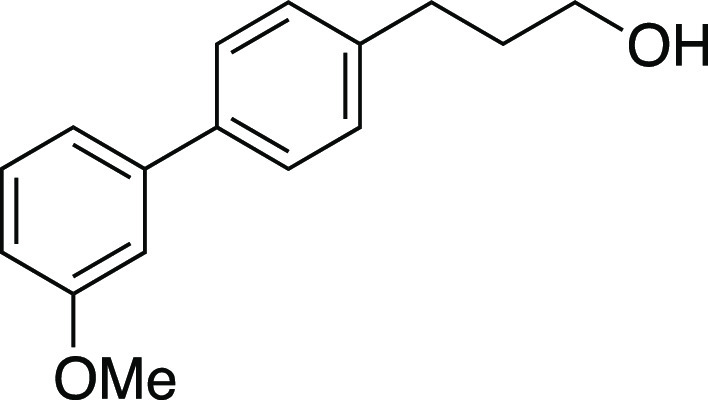


Alcohol **46** was prepared according to a procedure reported by Nagano.^36^ A two-neck round-bottom flask was equipped with a reflux condenser and a stir bar. Aryl bromide **53** (0.43 g, 2.0 mmol, 1.0 equiv), Pd(PPh_3_)_4_ (69 mg, 60. μmol, 0.030 equiv, 3.0 mol %), 3-methoxyphenyl boronic acid (0.36 g, 2.4 mmol, 1.2 equiv), K_2_CO_3_ (2.8 g, 20. mmol, 10. equiv), 1,4-dioxane (16 mL, 0.13 M in substrate), and H_2_O (4.0 mL, 0.50 M in substrate) were added under N_2_. The reaction mixture was allowed to stir at reflux in an oil bath overnight. Once complete, the flask was cooled to rt and H_2_O (10 mL) was added. The reaction mixture was then extracted with EtOAc (3 ×20 mL). The combined organic layers were washed with brine, dried over Na_2_SO_4_, and concentrated in vacuo. The residue was purified by column chromatography (0–30% EtOAc/hexanes) to afford the title compound as a white solid (0.40 g, 1.7 mmol, 83% yield). **m.p.** 41–44 °C; **TLC R**_**f**_ = 0.3 (25% EtOAc/hexanes, CAM stain); ^**1**^**H NMR** (400 MHz, CDCl_3_) δ 7.52 (d, *J* = 8.2 Hz, 2H), 7.34 (t, *J* = 7.9 Hz, 1H), 7.30–7.25 (m, 2H), 7.17 (ad, *J* = 7.7 Hz, 1H), 7.11 (at, *J* = 2.0 Hz, 1H), 6.91–6.85 (m, 1H), 3.86 (s, 3H), 3.72 (aq, *J* = 6.0 Hz, 2H), 2.76 (at, *J* = 7.7 Hz, 2H), 1.94 (aquint, *J* = 7.5 Hz, 2H), 1.27 (t, *J* = 5.2 Hz, 1H); ^**13**^**C{**^**1**^**H} NMR** (125.8 MHz, CDCl_3_) δ 160.0, 142.6, 141.2, 138.8, 129.8, 128.9 (2C), 127.2 (2C), 119.6, 112.8, 112.5, 62.3, 55.3, 34.2, 31.8; **IR** (neat) 3356, 2938, 1600, 1584, 1564, 1481, 1450, 1435, 1403, 1314, 1295, 1219, 1170, 1052, 1032, 1015, 835, 778, 696 cm^–1^; **HRMS** (TOF MS CI+) *m*/*z*: [M]^+^ calcd for C_16_H_18_O_2_, 242.1307; found, 242.1297. Analytical data is consistent with literature values.^8^
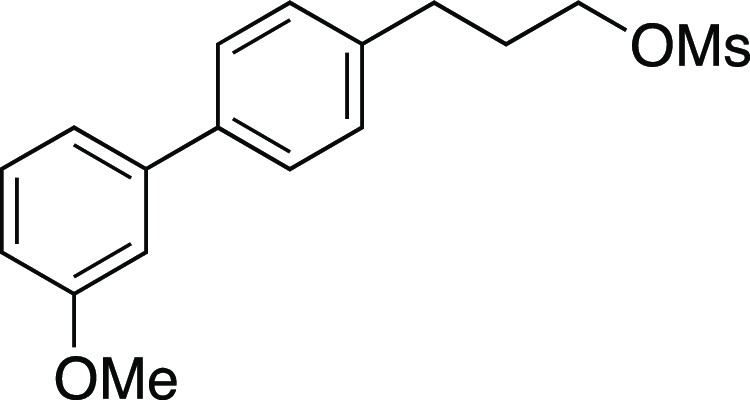


Mesylate **47** was prepared according to Method A. The following amounts of reagents were used: alcohol **46** (0.16 g, 0.65 mmol, 1.0 equiv), MsCl (76 μL, 0.98 mmol, 1.5 equiv), Et_3_N (0.14 mL, 0.98 mmol, 1.5 equiv), and DCM (3.3 mL, 0.20 M in substrate). The reaction mixture was allowed to stir overnight. The residue was purified by column chromatography (0–30% EtOAc/hexanes) to afford the title compound as a white solid (0.19 g, 0.61 mmol, 93% yield). **m.p.** 60–62 °C; **TLC R**_**f**_ = 0.3 (25% EtOAc/hexanes, CAM stain); ^**1**^**H NMR** (400 MHz, CDCl_3_) δ 7.53 (d, *J* = 8.1 Hz, 2H), 7.35 (t, *J* = 7.9 Hz, 1H), 7.28–7.23 (m, 2H), 7.16 (ad, *J* = 7.6 Hz, 1H), 7.11 (t, *J* = 2.0 Hz, 1H), 6.89 (dd, *J* = 8.5, 2.3 Hz, 1H), 4.26 (t, *J* = 6.3 Hz, 2H), 3.86 (s, 3H), 3.01 (s, 3H), 2.80 (t, *J* = 7.5 Hz, 2H), 2.12 (aquint, *J* = 6.9 Hz, 2H); ^**13**^**C{**^**1**^**H} NMR** (125.8 MHz, CDCl_3_) δ 160.0, 142.4, 139.6, 139.2, 129.8, 128.9 (2C), 127.4 (2C), 119.6, 112.8, 112.6, 69.1, 55.4, 37.5, 31.8, 30.7; **IR** (neat) 2939, 1600, 1481, 1351, 1295, 1220, 1172, 1052, 1030, 972, 926, 833, 781, 697 cm^–1^; **HRMS** (TOF MS ES+) *m*/*z*: [M + Na]^+^ calcd for C_17_H_20_O_4_SNa, 343.0980; found, 343.0987.
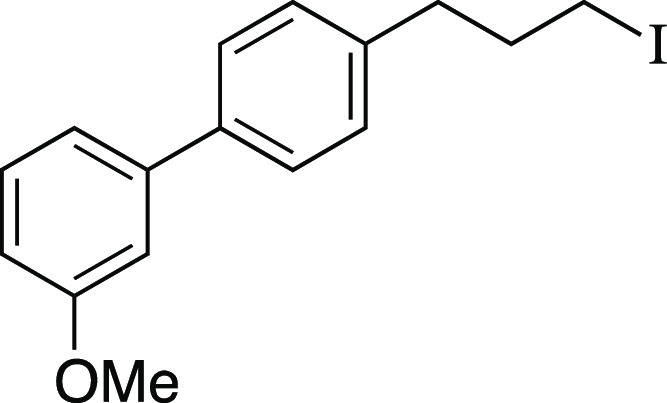


##### From Mesylate

Iodide **6** was prepared according to a modified Method B. The following amounts of reagents were used: mesylate **47** (32 mg, 0.10 mmol, 1.0 equiv), MeMgI (41 μL, 0.10 mmol, 1.0 equiv, 2.4 M in Et_2_O), and PhMe (1.0 mL, 0.10 M in substrate). Commercial MeMgI was used. The reaction mixture was allowed to stir at 0 °C for 1 h. The residue was purified by column chromatography (0–5% EtOAc/hexanes) to afford the title compound as a yellow oil (31 mg, 87 μmol, 87% yield). **TLC R**_**f**_ = 0.5 (5% EtOAc/hexanes, CAM stain); ^**1**^**H NMR** (400 MHz, CDCl_3_) δ 7.51 (ad, *J* = 8.1 Hz, 2H), 7.34 (t, *J* = 8.0 Hz, 1H), 7.25 (ad, *J* = 8.1 Hz, 2H), 7.16 (ad, *J* = 7.7 Hz, 1H), 7.11 (at, *J* = 2.0 Hz, 1H), 6.88 (dd, *J* = 8.2, 2.5 Hz, 1H), 3.85 (s, 3H), 3.19 (t, *J* = 6.8 Hz, 2H), 2.76 (t, *J* = 7.4 Hz, 2H), 2.16 (quint, *J* = 7.1 Hz, 2H); ^**13**^**C{**^**1**^**H} NMR** (125.8 MHz, CDCl_3_) δ 160.1, 142.6, 139.8, 139.2, 129.9, 129.1 (2C), 127.4 (2C), 119.7, 112.9, 112.7, 55.4, 36.0, 35.0, 6.4; **IR** (neat) 2936, 2832, 1599, 1583, 1563, 1518, 1480, 1402, 1295, 1213, 1168, 1052, 1031, 1015, 861, 822, 776, 695 cm^–1^; **HRMS** (TOF MS CI+) *m*/*z*: [M]^+^ calcd for C_16_H_17_IO, 352.0324; found, 352.0340.

##### From Alcohol Using MeMgI Prepared from Freshly Distilled MeI

Iodide **6** was prepared according to a modified Method C. The following amounts of reagents were used: alcohol **46** (24 mg, 0.10 mmol, 1.0 equiv), MsCl (12 μL, 0.15 mmol, 1.5 equiv), Et_3_N (21 μL, 0.15 mmol, 1.5 equiv), DCM (0.50 mL, 0.20 M in substrate), MeMgI (69 μL, 0.20 mmol, 2.0 equiv, 2.9 M in Et_2_O), and PhMe (0.50 mL, 0.20 M in substrate). Upon the addition of MsCl, the reaction mixture was allowed to stir at rt for 5 min. The residue was purified by column chromatography (0–5% EtOAc/hexanes) to afford the title compound as a yellow oil (31 mg, 87 μmol, 87% yield). Refer to iodide **6** above for analytical data.

##### From Alcohol Using Commercially Available MeMgI

Iodide **6** was prepared according to a modified Method C. The following amounts of reagents were used: alcohol **46** (24 mg, 0.10 mmol, 1.0 equiv), MsCl (12 μL, 0.15 mmol, 1.5 equiv), Et_3_N (21 μL, 0.15 mmol, 1.5 equiv), DCM (0.50 mL, 0.20 M in substrate), MeMgI (82 μL, 0.20 mmol, 2.0 equiv, 2.4 M in Et_2_O), and PhMe (0.50 mL, 0.20 M in substrate). Commercial MeMgI was used. Upon the addition of MeMgI, the reaction mixture was allowed to stir at 0 °C for 1 h. The residue was purified by column chromatography (0–5% EtOAc/hexanes) to afford the title compound as a yellow oil (31 mg, 88 μmol, 88% yield). Refer to iodide **6** above for analytical data.
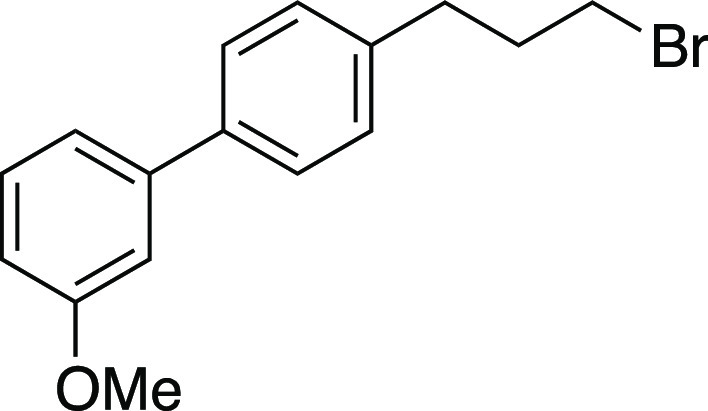


##### From Mesylate

Bromide **31** was prepared according to Method D. The following amounts of reagents were used: mesylate **47** (32 mg, 0.10 mmol, 1.0 equiv), MeMgBr (67 μL, 0.20 mmol, 2.0 equiv, 3.0 M in Et_2_O), and PhMe (1.0 mL, 0.10 M in substrate). The residue was purified by column chromatography (0–5% EtOAc/hexanes) to afford the title compound as a pale-yellow oil (25 mg, 82 μmol, 82% yield). **TLC R**_**f**_ = 0.3 (5% EtOAc/hexanes, CAM stain); ^**1**^**H NMR** (400 MHz, CDCl_3_) δ 7.52 (ad, *J* = 8.1 Hz, 2H), 7.34 (t, *J* = 7.9 Hz, 1H), 7.28–7.22 (m, 2H), 7.16 (ad, *J* = 7.9 Hz, 1H), 7.11 (at, *J* = 2.0 Hz, 1H), 6.88 (dd, *J* = 8.3, 2.5 Hz, 1H), 3.85 (s, 3H), 3.42 (t, *J* = 6.6 Hz, 2H), 2.81 (t, *J* = 7.4 Hz, 2H), 2.20 (aquint, *J* = 7.0 Hz, 2H); ^**13**^**C{**^**1**^**H} NMR** (125.8 MHz, CDCl_3_) δ 160.0, 142.5, 139.9, 139.1, 129.8, 129.0 (2C), 127.3 (2C), 119.6, 112.8, 112.6, 55.4, 34.2, 33.7, 33.2; **IR** (neat) 2937, 1600, 1584, 1564, 1518, 1480, 1435, 1403, 1295, 1220, 1170, 1053, 1032, 1016, 866, 829, 777, 696 cm^–1^; **HRMS** (TOF MS CI+) *m*/*z*: [M]^+^ calcd for C_16_H_17_BrO, 304.0463; found, 304.0478.

##### From Alcohol

Bromide **31** was prepared according to Method E. The following amounts of reagents were used: alcohol **46** (24 mg, 0.10 mmol, 1.0 equiv), MsCl (12 μL, 0.15 mmol, 1.5 equiv), Et_3_N (21 μL, 0.15 mmol, 1.5 equiv), DCM (0.50 mL, 0.20 M in substrate), MeMgBr (0.11 mL, 0.30 mmol, 3.0 equiv, 2.7 M in Et_2_O), and PhMe (0.50 mL, 0.20 M in substrate). The residue was purified by column chromatography (0–5% EtOAc/hexanes) to afford the title compound as a pale-yellow oil (26 mg, 86 μmol, 86% yield). Refer to bromide **31** above for analytical data.
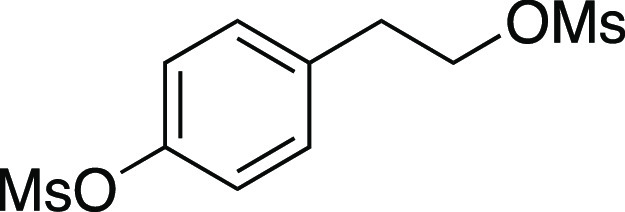


Mesylate **48** was prepared according to a modified Method A. The following amounts of reagents were used: 2-(4-hydroxyphenyl) ethanol (0.14 g, 1.0 mmol, 1.0 equiv), MsCl (0.19 mL, 2.5 mmol, 2.5 equiv), Et_3_N (0.35 mL, 2.5 mmol, 2.5 equiv), and DCM (5.0 mL, 0.20 M in substrate). The reaction mixture was allowed to stir overnight. The residue was purified by column chromatography (0–50% EtOAc/hexanes) to afford the title compound as a yellow oil (0.25 g, 0.86 mmol, 86% yield). **TLC R**_**f**_ = 0.3 (50% EtOAc/hexanes, KMnO_4_ stain); ^**1**^**H NMR** (500 MHz, CDCl_3_) δ 7.30 (d, *J* = 8.6 Hz, 2H), 7.27–7.24 (m, 2H), 4.42 (t, *J* = 6.8 Hz, 2H), 3.15 (s, 3H), 3.08 (t, *J* = 6.8 Hz, 2H), 2.91 (s, 3H). Analytical data is consistent with literature values.^37^
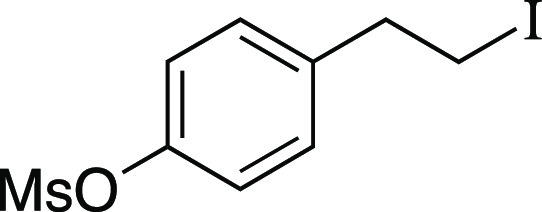


Iodide **7** was prepared according to a modified Method B. The following amounts of reagents were used: mesylate **48** (30. mg, 0.10 mmol, 1.0 equiv), MeMgI (0.17 mL, 0.50 mmol, 5.0 equiv, 3.0 M in Et_2_O), and PhMe (1.0 mL, 0.10 M in substrate). The residue was purified by column chromatography (0–25% EtOAc/hexanes) to afford the title compound as a white solid (23 mg, 69 μmol, 69% yield). **m.p.** 74–76 °C; **TLC R**_**f**_ = 0.2 (20% EtOAc/hexanes, CAM stain); ^**1**^**H NMR** (500 MHz, CDCl_3_) δ 7.27–7.23 (m, 4H), 3.34 (t, *J* = 7.6 Hz, 2H), 3.19 (t, *J* = 7.6 Hz, 2H), 3.14 (s, 3H); ^**13**^**C{**^**1**^**H} NMR** (125.8 MHz, CDCl_3_) δ 148.0, 140.0, 130.0 (2C), 122.3 (2C), 39.5, 37.4, 4.9; **IR** (neat) 2921, 2851, 1499, 1362, 1331, 1193, 1170, 1143, 1020, 976, 870, 844, 821, 780, 731, 701, 599, 564 cm^–1^; **HRMS** (TOF MS ES+) *m*/*z*: [M + Na]^+^ calcd for C_9_H_11_IO_3_SNa, 348.9371; found, 348.9382.
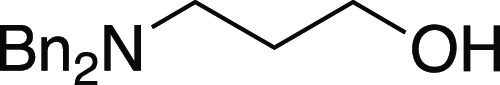


Alcohol **49** was prepared following a procedure reported by Porzi.^38^ To a flame-dried round-bottom flask equipped with a stir bar were added 3-amino-1-propanol (0.23 mL, 3.0 mmol, 1.0 equiv), K_2_CO_3_ (870 mg, 6.3 mmol, 2.1 equiv), and acetone (6.0 mL, 0.50 M in substrate). The reaction mixture was allowed to stir for 5 min at rt. Benzyl bromide (0.75 mL, 6.3 mmol, 2.1 equiv) was then added dropwise via a syringe, and the reaction mixture was allowed to stir for 16 h at rt. The reaction was filtered over a pad of celite while flushing with DCM and then concentrated in vacuo. The residue was purified by flash column chromatography (0–30% EtOAc/hexanes) to afford the title compound as a yellow oil (0.60 g, 2.4 mmol, 79%). **TLC R**_**f**_ = 0.3 (20% EtOAc/hexanes); ^**1**^**H NMR** (400 MHz, CDCl_3_) δ 7.30–7.24 (m, 8H), 7.21–7.17 (m, 2H), 4.42 (br s, 1H), 3.58 (t, *J* = 5.5 Hz, 2H), 3.50 (s, 4H), 2.55 (t, *J* = 6.0 Hz, 2H), 1.68 (tt, *J* = 8.7, 5.8 Hz, 2H). Analytical data is consistent with literature values.^38^
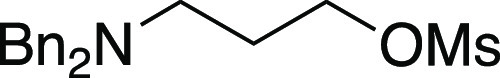


Mesylate **50** was prepared according to Method A. The following amounts of reagents were used: alcohol **49** (0.26 g, 1.0 mmol, 1.0 equiv), MsCl (0.12 mL, 1.5 mmol, 1.5 equiv), Et_3_N (0.21 mL, 1.5 mmol, 1.5 equiv), and DCM (5.0 mL, 0.20 M in substrate). The reaction mixture was allowed to stir overnight. The residue was purified by flash column chromatography (0–30% EtOAc/hexanes) to afford the title compound as a pale-yellow oil (0.19 g, 0.57 mmol, 57%). **TLC R**_**f**_ = 0.3 (30% EtOAc/hexanes); ^**1**^**H NMR** (400 MHz, CDCl_3_) δ 7.33–7.27 (m, 8H), 7.23–7.20 (m, 2H), 4.19 (t, *J* = 6.4 Hz, 2H), 3.53 (s, 4H), 2.76 (s, 3H), 2.52 (t, *J* = 6.6 Hz, 2H), 1.85 (tt, *J* = 9.7, 6.5 Hz, 2H). Analytical data is consistent with literature values.^39^
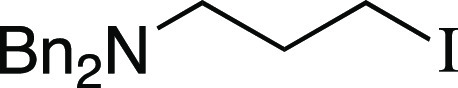


Iodide **8** was prepared according to Method B. The following amounts of reagents were used: mesylate **50** (34 mg, 0.10 mmol, 1.0 equiv), MeMgI (34 μL, 0.10 mmol, 1.0 equiv, 3.0 M in Et_2_O), and PhMe (1.0 mL, 0.10 M in substrate). The residue was purified by flash column chromatography (0–10% Et_2_O/hexanes) to afford the title compound as a yellow oil (23 mg, 62 μmol, 60%). **TLC R**_**f**_ = 0.5 (10% EtOAc/hexanes, CAM stain); ^**1**^**H NMR** (400 MHz, CDCl_3_) δ 7.34–7.29 (m, 8H), 7.24–7.21 (m, 2H), 3.55 (s, 4H), 3.15 (t, *J* = 7.0 Hz, 2H), 2.51 (t, *J* = 6.5 Hz, 2H), 2.02–1.96 (m, 2H). Analytical data is consistent with literature values.^38^
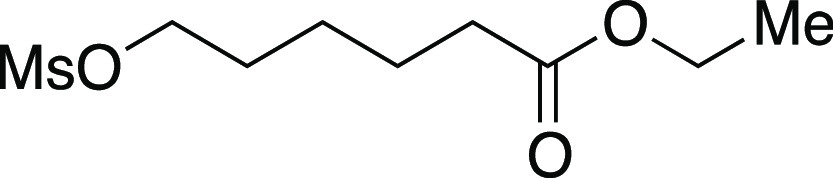


Mesylate **51** was prepared according to Method A. The following amounts of reagents were used: 6-hydroxy-hexanoic acid ethyl ester (0.33 mL, 2.0 mmol, 1.0 equiv), MsCl (0.23 mL, 3.0 mmol, 1.5 equiv), Et_3_N (0.42 mL, 3.0 mmol, 1.5 equiv), and DCM (10. mL, 0.20 M in substrate). The reaction mixture was allowed to stir overnight. The residue was purified by flash column chromatography (0–25% EtOAc/hexanes) to afford the title compound as a pale-yellow oil (0.46 g, 1.9 mmol, 96%). **TLC R**_**f**_ = 0.2 (25% EtOAc/hexanes, PMA stain); ^**1**^**H NMR** (400 MHz, CDCl_3_) δ 4.23 (t, *J* = 6.5 Hz, 2H), 4.13 (q, *J* = 7.1 Hz, 2H), 3.00 (s, 3H), 2.32 (t, *J* = 7.4 Hz, 2H), 1.81–1.74 (m, 2H), 1.71–1.64 (m, 2H), 1.49–1.44 (m, 2H), 1.26 (t, *J* = 7.1 Hz, 3H). Analytical data is consistent with literature values.^40^
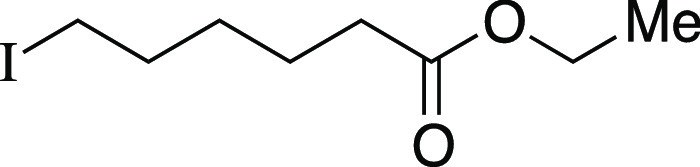


##### From Mesylate

Iodide **9** was prepared according to a modified Method B. The following amounts of reagents were used: mesylate **51** (25 mg, 0.10 mmol, 1.0 equiv), MeMgI (34 μL, 0.10 mmol, 1.0 equiv, 3.0 M in Et_2_O), and PhMe (1.0 mL, 0.10 M in substrate). The reaction mixture was allowed to stir at −78 °C for 3 h. The residue was purified by flash column chromatography (0–10% EtOAc/hexanes) to afford the title compound as a pale-yellow oil (18 mg, 68 μmol, 66%). **TLC R**_**f**_ = 0.4 (10% EtOAc/hexanes); ^**1**^**H NMR** (400 MHz, CDCl_3_) δ 4.13 (q, *J* = 7.1 Hz, 2H), 3.19 (t, *J* = 7.0 Hz, 2H), 2.31 (t, *J* = 7.4 Hz, 2H), 1.85 (quint, *J* = 7.2 Hz, 2H), 1.65 (quint, *J* = 7.6 Hz, 2H), 1.48–1.42 (m, 2H), 1.26 (t, *J* = 7.1 Hz, 3H). Analytical data is consistent with literature values.^41^

##### From Alcohol

Iodide **9** was prepared according to a modified Method C. The following amounts of reagents were used: ethyl 6-hydroxyhexanoate (20. μL, 0.12 mmol, 1.0 equiv), MsCl (10. μL, 0.12 mmol, 1.0 equiv), Et_3_N (17 μL, 0.12 mmol, 1.0 equiv), DCM (0.62 mL, 0.20 M in substrate), followed by MeMgI (40 μL, 0.12 mmol, 1.0 equiv, 3.0 M in Et_2_O), and PhMe (0.62 mL, 0.20 M in substrate). Upon addition of MeMgI, the reaction mixture was allowed to stir at −78 °C for 3 h. The residue was purified by flash column chromatography (0–10% Et_2_O/hexanes) to afford the title compound as a pale-yellow oil (24 mg, 90. μmol, 73%). Refer to iodide **9** above for analytical data.
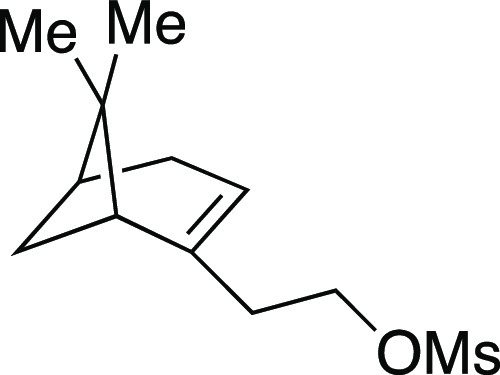


Mesylate **52** was prepared according to Method A. The following amounts of reagents were used: (1*R*)-(−)-nopol (0.17 mL, 1.0 mmol, 1.0 equiv), MsCl (0.12 mL, 1.5 mmol, 1.5 equiv), Et_3_N (0.21 mL, 1.5 mmol, 1.5 equiv), and DCM (5.0 mL, 0.20 M in substrate). The reaction mixture was allowed to stir overnight. The residue was purified by flash column chromatography (0–20% EtOAc/hexanes) to afford the title compound as a pale-yellow oil (0.24 g, 0.98 mmol, 98%). **TLC R**_**f**_ = 0.4 (20% EtOAc/hexanes, PMA stain); ^**1**^**H NMR** (400 MHz, CDCl_3_) δ 5.36 (br s, 1H), 4.21 (at, *J* = 7.1 Hz, 2H), 2.99 (s, 3H), 2.43–2.36 (m, 3H), 2.30–2.18 (m, 2H), 2.10–2.04 (m, 2H), 1.29 (s, 3H), 1.16 (d, *J* = 8.6 Hz, 1H), 0.84 (s, 3H); ^**13**^**C{**^**1**^**H} NMR** (100 MHz, CDCl_3_) δ 142.6, 119.8, 68.0, 45.5, 40.6, 38.0, 37.4, 36.3, 31.5, 31.3, 26.2, 21.1. **HRMS** (TOF MS CI+) *m*/*z*: [M]^+^ calculated for C_12_H_20_O_3_S 244.1133, found 244.1126.
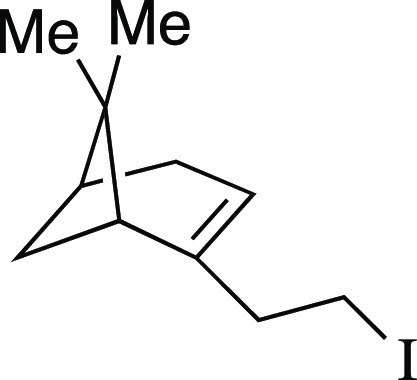


##### From Mesylate

Iodide **10** was prepared according to Method B. The following amounts of reagents were used: mesylate **52** (25 mg, 0.10 mmol, 1.0 equiv), MeMgI (32 μL, 0.10 mmol, 1.0 equiv, 3.2 M in Et_2_O), and PhMe (1.0 mL, 0.10 M in substrate). The residue was purified by flash column chromatography (100% hexanes) to afford the title compound as a colorless oil (23 mg, 83 μmol, 81%). **TLC R**_**f**_ = 0.8 (100% hexanes, PMA stain); ^**1**^**H NMR** (400 MHz, CDCl_3_) δ 5.31 (br s, 1H), 3.17–3.10 (m, 2H), 2.56–2.53 (m, 2H), 2.37 (dt, *J* = 8.6, 5.6 Hz, 1H), 2.27–2.24 (m, 1H), 2.19–2.15 (m, 1H), 2.08 (br s, 1H), 2.00 (td, *J* = 5.6, 1.4 Hz, 1H), 1.27 (s, 3H), 1.18 (d, *J* = 8.6 Hz, 1H), 0.84 (s, 3H). Analytical data is consistent with literature values.^21^

##### From Alcohol, 0.10 mmol Scale

Iodide **10** was prepared according to a modified Method C. The following amounts of reagents were used: (1*R*)-(−)-nopol (20. μL, 0.10 mmol, 1.0 equiv), MsCl (12 μL, 0.15 mmol, 1.5 equiv), Et_3_N (21 μL, 0.15 mmol, 1.5 equiv), DCM (0.50 mL, 0.20 M in substrate), followed by MeMgI (94 μL, 0.30 mmol, 3.0 equiv, 3.2 M in Et_2_O), and PhMe (0.50 mL, 0.20 M in substrate). The residue was purified by flash column chromatography (100% hexanes) to afford the title compound as a colorless oil (25 mg, 91 μmol, 91%). Refer to iodide **10** above for analytical data.

##### From Alcohol, 12 mmol Scale

To a flame-dried round-bottom flask equipped with a stir bar was added (1*R*)-(−)-nopol (2.1 mL, 12 mmol, 1.0 equiv), anhydrous DCM (60. mL, 0.20 M in substrate), then Et_3_N (2.5 mL, 18 mmol, 1.5 equiv). The reaction mixture was allowed to stir at rt for 5 min before adding MsCl (1.4 mL, 18 mmol, 1.5 equiv). The reaction mixture was allowed to stir at rt for 1 h before adding PhMe (60. mL, 0.20 M in substrate) and cooling to 0 °C. After 5 min at 0 °C, commercial MeMgI (15 mL, 36 mmol, 2.4 M in Et_2_O) was added, and the reaction mixture was allowed to stir at 0 °C for 5 min. To quench, sat. NH_4_Cl solution was added, and the biphasic mixture was extracted with DCM (×3), washed with brine, dried over Na_2_SO_4_, and concentrated in vacuo. The residue was purified by flash column chromatography (100% hexanes) to afford the title compound as a colorless oil (3.1 g, 11 mmol, 92% yield). Refer to iodide **10** above for analytical data.
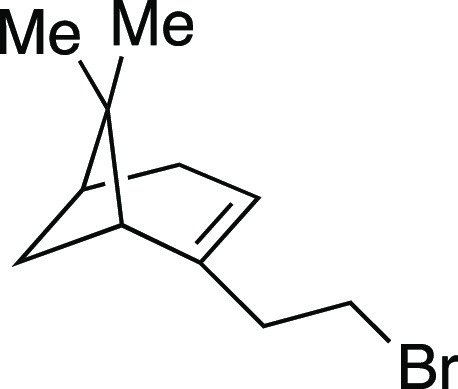


##### From Mesylate

Bromide **32** was prepared according to Method D. The following amounts of reagents were used: mesylate **52** (29 mg, 0.12 mmol, 1.0 equiv), MeMgBr (90. μL, 0.23 mmol, 2.0 equiv, 2.7 M in Et_2_O), and PhMe (1.2 mL, 0.10 M in substrate). The residue was purified by flash column chromatography (100% hexanes) to afford the title compound as a colorless oil (18 mg, 77 μmol, 60%). **TLC R**_**f**_ = 0.7 (100% hexanes, PMA stain); ^**1**^**H NMR** (400 MHz, CDCl_3_) δ 5.32 (br s, 1H), 3.38–3.34 (m, 2H), 2.52 (at, *J* = 7.8 Hz, 2H), 2.40–2.35 (m, 1H), 2.30–2.17 (m, 2H), 2.09 (br s, 1H), 2.02 (at, *J* = 5.5 Hz, 1H), 1.28 (s, 3H), 1.17 (d, *J* = 8.8 Hz, 1H), 0.84 (s, 3H). Analytical data is consistent with literature values.^42^

##### From Alcohol

Bromide **32** was prepared according to a modified Method E. The following amounts of reagents were used: (1*R*)-(−)-nopol (20. μL, 0.10 mmol, 1.0 equiv), MsCl (12 μL, 0.15 mmol, 1.5 equiv), Et_3_N (21 μL, 0.15 mmol, 1.5 equiv), DCM (0.50 mL, 0.20 M in substrate), followed by MeMgBr (0.15 mL, 0.40 mmol, 4.0 equiv, 2.7 M in Et_2_O), and PhMe (0.50 mL, 0.20 M in substrate). The residue was purified by flash column chromatography (100% hexanes) to afford the title compound as a colorless oil (17 mg, 75 μmol, 75%). Refer to bromide **32** above for analytical data.
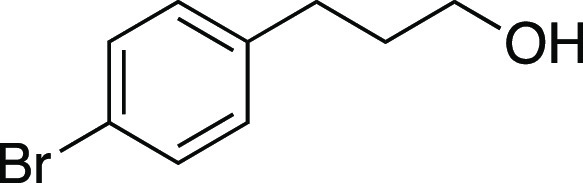


Alcohol **53** was prepared according to a modified procedure reported by Cole.^43^ Under a N_2_ atmosphere, a flame-dried round-bottom flask equipped with a stir bar was charged with the 3-(4-bromophenyl)propionic acid (1.1 g, 5.0 mmol, 1.0 equiv) and THF (10. mL, 0.50 M in substrate). The reaction mixture was cooled to 0 °C and BH_3_·THF (15 mL, 1.5 mmol, 3.0 equiv, 1.0 M in THF) was added dropwise. The mixture was brought to rt and allowed to stir for 16 h. Then, glacial acetic acid was added dropwise until quenched, followed by the addition of sat. aqueous NaHCO_3_ until a pH of 7 was achieved. This mixture was extracted with EtOAc (×3), and the organic layers were combined, dried with Na_2_SO_4_, and concentrated in vacuo. The residue was purified by column chromatography (0–20% EtOAc/hexanes) to afford the title compound as a yellow oil (0.94 g, 4.4 mmol, 88% yield). **TLC R**_**f**_ = 0.3 (25% EtOAc/hexanes); ^**1**^**H NMR** (500 MHz, CDCl_3_) δ 7.40 (d, *J* = 8.2 Hz, 2H), 7.07 (d, *J* = 8.4 Hz, 2H), 3.67 (t, *J* = 6.3 Hz, 2H), 2.67 (at, *J* = 7.7 Hz, 2H), 1.86 (tt, *J* = 7.8, 6.4 Hz, 2H), 1.29 (br s, 1H). Analytical data is consistent with literature values.^44^
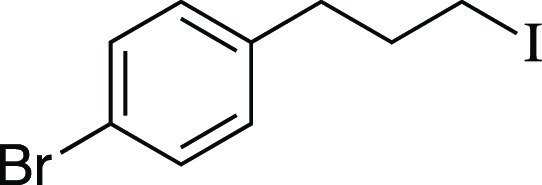


Iodide **11** was prepared according to Method C. The following amounts of reagents were used: alcohol **53** (0.22 g, 1.0 mmol, 1.0 equiv), MsCl (0.12 mL, 1.5 mmol, 1.5 equiv), Et_3_N (0.21 mL, 1.5 mmol, 1.5 equiv), DCM (5.0 mL, 0.20 M in substrate), MeMgI (0.69 mL, 2.0 mmol, 2.0 equiv, 2.9 M in Et_2_O), PhMe (5.0 mL, 0.20 M in substrate). The residue was purified by column chromatography (0–5% EtOAc/hexanes) to afford the title compound as a colorless oil (0.29 g, 0.88 mmol, 88% yield). **TLC R**_**f**_ = 0.7 (5% EtOAc/hexanes, CAM stain); ^**1**^**H NMR** (400 MHz, CDCl_3_) δ 7.41 (d, *J* = 8.4 Hz, 2H), 7.07 (d, *J* = 8.3 Hz, 2H), 3.15 (t, *J* = 6.8 Hz, 2H), 2.69 (t, *J* = 7.4 Hz, 2H), 2.10 (aquint, *J* = 7.0 Hz, 2H); ^**13**^**C{**^**1**^**H} NMR** (125.8 MHz, CDCl_3_) δ 129.4, 131.6 (2C), 130.4 (2C), 120.0, 35.6, 34.6, 5.9; **IR** (neat) 2928, 2855, 1487, 1445, 1424, 1403, 1210, 1071, 1010, 860, 812, 780, 746, 709, 649, 632 cm^–1^; **HRMS** (TOF MS CI+) *m*/*z*: [M]^+^ calcd for C_9_H_10_BrI, 323.9011; found, 323.9016. Analytical data is consistent with literature values.^44^
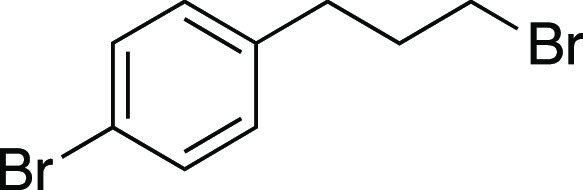


Bromide **33** was prepared according to Method E. The following amounts of reagents were used: alcohol **53** (22 mg, 0.10 mmol, 1.0 equiv), MsCl (12 μL, 0.15 mmol, 1.5 equiv), Et_3_N (21 μL, 0.15 mmol, 1.5 equiv), DCM (0.50 mL, 0.20 M in substrate), MeMgBr (0.10 mL, 0.30 mmol, 3.0 equiv, 3.0 M in Et_2_O), PhMe (0.50 mL, 0.20 M in substrate). The residue was purified by column chromatography (0–5% EtOAc/hexanes) to afford the title compound as a pale-yellow oil (21 mg, 77 μmol, 77% yield). **TLC R**_**f**_ = 0.5 (5% EtOAc/hexanes, CAM stain); ^**1**^**H NMR** (400 MHz, CDCl_3_) δ 7.41 (d, *J* = 8.4 Hz, 2H), 7.07 (d, *J* = 8.3 Hz, 2H), 3.37 (t, *J* = 6.5 Hz, 2H), 2.74 (t, *J* = 7.3 Hz, 2H), 2.13 (aquint, *J* = 6.9 Hz, 2H); ^**13**^**C{**^**1**^**H} NMR** (125.8 MHz, CDCl_3_) δ 139.5, 131.6 (2C), 130.4 (2C), 120.0, 33.9, 33.4, 32.8; **IR** (neat) 2918, 2849, 1488, 1435, 1240, 1072, 1011, 865, 823, 795, 560 cm^–1^; **HRMS** (TOF MS CI+) *m*/*z*: [M]^+^ calcd for C_9_H_10_Br_2_, 275.9149; found, 275.9146. Analytical data is consistent with literature values.^45^
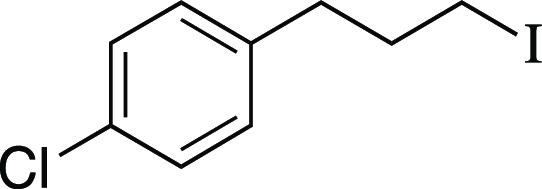


Iodide **12** was prepared according to Method C. The following amounts of reagents were used: 3-(4-chlorophenyl)-1-propanol (18 mg, 0.11 mmol, 1.0 equiv), MsCl (13 μL, 0.17 mmol, 1.5 equiv), Et_3_N (23 μL, 0.17 mmol, 1.5 equiv), DCM (0.55 mL, 0.20 M in substrate), MeMgI (73 μL, 0.22 mmol, 2.0 equiv, 3.0 M in Et_2_O), PhMe (0.55 mL, 0.20 M in substrate). The residue was purified by column chromatography (0–5% EtOAc/hexanes) to afford the title compound as a yellow oil (25 mg, 87 μmol, 82% yield). **TLC R**_**f**_ = 0.4 (100% hexanes, CAM stain); ^**1**^**H NMR** (400 MHz, CDCl_3_) δ 7.29–7.21 (m, 2H), 7.12 (d, *J* = 8.5 Hz, 2H), 3.15 (t, *J* = 6.7 Hz, 2H), 2.70 (t, *J* = 7.3 Hz, 2H), 2.09 (quint, *J* = 7.1 Hz, 2H). Analytical data is consistent with literature values.^46^
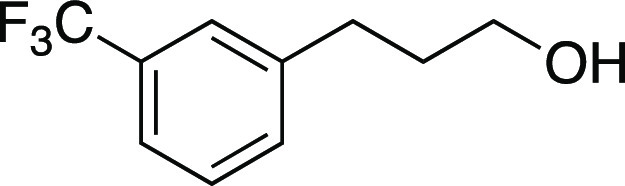


Alcohol **54** was prepared according to the following procedure. Open to air, a round-bottom flask with a stir bar was charged with aldehyde **SI-5** (0.11 g, 0.53 mmol, 1.0 equiv), NaBH_4_ (40. mg, 1.0 mmol, 2.0 equiv), and MeOH (2.7 mL, 0.20 M in substrate). The reaction mixture was stirred for 1 h. After completion, the reaction mixture was concentrated in vacuo, and then dissolved in DCM. H_2_O was added and the aqueous layer was extracted with DCM. The combined organic layers were dried and concentrated in vacuo. The residue was purified by column chromatography (0–25% EtOAc/hexanes) to afford the title compound as a colorless oil (75 mg, 0.37 mmol, 70% yield). **TLC R**_**f**_ = 0.2 (20% EtOAc/hexanes, KMnO_4_ stain); ^**1**^**H NMR** (400 MHz, CDCl_3_) δ 7.48–7.36 (m, 4H), 3.69 (t, *J* = 6.4 Hz, 2H), 2.78 (at, *J* = 7.8 Hz, 2H), 1.95–1.87 (m, 2H), 1.30 (br s, 1H); ^**13**^**C{**^**1**^**H} NMR** (150.9 MHz, CDCl_3_) δ 142.8, 131.9 (q, *J* = 1.0 Hz), 130.7 (q, *J* = 32.1 Hz), 128.8, 125.2 (q, *J* = 3.8 Hz), 124.3 (q, *J* = 272.1 Hz), 122.8 (q, *J* = 3.9 Hz), 62.0, 34.0, 31.9; ^**19**^**F NMR** (376.5 MHz, CDCl_3_) δ −62.6 (3F); **IR** (neat) 3328, 2939, 1450, 1331, 1200, 1162, 112, 1073, 800, 702, 661 cm^–1^; **HRMS** (TOF MS ES−) *m*/*z*: [M – H]^−^ calcd for C_10_H_10_F_3_O, 203.0684; found, 203.0680. Analytical data is consistent with literature values.^47^
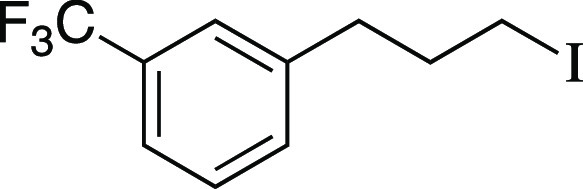


Iodide **13** was prepared according to Method C. The following amounts of reagents were used: alcohol **54** (20 mg, 0.10 mmol, 1.0 equiv), MsCl (12 μL, 0.15 mmol, 1.5 equiv), Et_3_N (21 μL, 0.15 mmol, 1.5 equiv), DCM (0.50 mL, 0.20 M in substrate), MeMgI (69 μL, 0.20 mmol, 2.0 equiv, 2.9 M in Et_2_O), PhMe (0.50 mL, 0.20 M in substrate). The residue was purified by column chromatography (0–5% EtOAc/hexane) to afford the title compound as a yellow oil (27 mg, 85 μmol, 85% yield). **TLC R**_**f**_ = 0.7 (5% EtOAc/hexanes, CAM stain); ^**1**^**H NMR** (400 MHz, CDCl_3_) δ 7.50–7.36 (m, 4H), 3.17 (t, *J* = 6.8 Hz, 2H), 2.80 (at, *J* = 7.4 Hz, 2H), 2.15 (aquint, *J* = 7.1 Hz, 2H); ^**13**^**C{**^**1**^**H} NMR** (150.9 MHz, CDCl_3_) δ 141.4, 132.0, 130.9 (q, *J* = 32.0 Hz), 129.0, 125.3 (q, *J* = 3.7 Hz), 124.2 (q, *J* = 272.5 Hz), 123.2 (q, *J* = 3.9 Hz), 36.1, 34.6, 5.7; ^**19**^**F NMR** (376.5 MHz, CDCl_3_) δ −62.6 (3F); **IR** (neat) 2923, 2851, 1450, 1328, 1213, 1199, 1164, 1125, 1073, 797, 702, 661 cm^–1^; **HRMS** (TOF MS CI+) *m*/*z*: [M]^+^ calcd for C_10_H_10_F_3_I, 313.9779; found, 313.9778. Analytical data is consistent with literature values.^48^
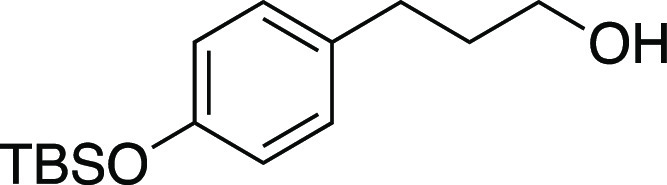


Alcohol **55** was prepared according to the following procedure. In a glovebox, a flame-dried round-bottom flask with a stir bar was charged with LiAlH_4_ (88 mg, 2.3 mmol, 2.2 equiv), capped, and brought out of the glovebox. A N_2_ inlet and THF (5.3 mL, 0.20 M in substrate) was added. The mixture was cooled to 0 °C, and carboxylic acid **SI-6** (0.29 g, 1.1 mmol, 1.0 equiv) was added as a solution in THF (1.0 M in substrate). The reaction mixture was warmed to rt and allowed to stir overnight. Then, sat. aqueous NH_4_Cl was added, and the crude mixture was extracted with EtOAc (3 × 20 mL). The combined organic layers were washed with brine, dried over Na_2_SO_4_, and concentrated in vacuo. The residue was purified by column chromatography (0–25% EtOAc/hexanes) to afford the title compound as a colorless oil (0.14 g, 0.51 mmol, 49% yield). **TLC R**_**f**_ = 0.5 (33% EtOAc/hexanes); ^**1**^**H NMR** (400 MHz, CDCl_3_) δ 7.04 (ad, *J* = 8.4 Hz, 2H), 6.75 (ad, *J* = 8.5 Hz, 2H), 3.66 (t, *J* = 6.4 Hz, 2H), 2.64 (at, *J* = 7.6 Hz, 2H), 1.86 (tt, *J* = 7.6, 6.5 Hz, 2H), 1.23 (br s, 1H), 0.98 (s, 9H), 0.18 (s, 6H). Analytical data is consistent with literature values.^49^
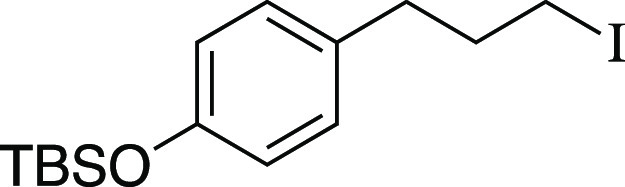


Iodide **14** was prepared according to Method C. The following amounts of reagents were used: alcohol **55** (27 mg, 0.10 mmol, 1.0 equiv), MsCl (12 μL, 0.15 mmol, 1.5 equiv), Et_3_N (21 μL, 0.15 mmol, 1.5 equiv), DCM (0.50 mL, 0.20 M in substrate), MeMgI (68 μL, 0.20 mmol, 2.0 equiv, 3.0 M in Et_2_O), PhMe (0.50 mL, 0.20 M in substrate). The residue was purified by column chromatography (0–5% EtOAc/hexanes) to afford the title compound as a pale-yellow oil (36 mg, 95 μmol, 95% yield). **TLC R**_**f**_ = 0.8 (5% EtOAc/hexanes, CAM stain); ^**1**^**H NMR** (500 MHz, CDCl_3_) δ 7.03 (d, *J* = 8.2 Hz, 2H), 6.75 (d, *J* = 8.1 Hz, 2H); 3.15 (t, *J* = 6.8 Hz, 2H), 2.65 (t, *J* = 7.2 Hz, 2H), 2.09 (quint, *J* = 7.1 Hz, 2H), 0.98 (s, 9H), 0.18 (s, 6H); ^**13**^**C{**^**1**^**H} NMR** (125.4 MHz, CDCl_3_) δ 154.0, 133.1, 129.5 (2C), 120.1 (2C), 35.4, 35.1, 25.8 (3C), 18.2, 6.5, −4.4 (2C); **IR** (neat) 2955, 2928, 2856, 1609, 1508, 1471, 1252, 1212, 1168, 912, 837, 808, 779, 747, 687 cm^–1^; **HRMS** (TOF MS CI+) *m*/*z*: [M]^+^ calcd for C_15_H_25_IOSi, 376.0720; found, 376.0704. Analytical data is consistent with literature values.^50^
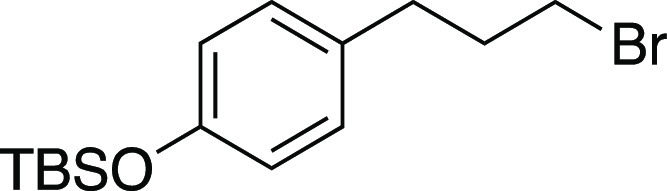


Bromide **34** was prepared according to Method E. The following amounts of reagents were used: alcohol **55** (27 mg, 0.10 mmol, 1.0 equiv), MsCl (12 μL, 0.15 mmol, 1.5 equiv), Et_3_N (21 μL, 0.15 mmol, 1.5 equiv), DCM (0.50 mL, 0.20 M in substrate), MeMgBr (0.10 mL, 0.30 mmol, 3.0 equiv, 3.0 M in Et_2_O), PhMe (0.50 mL, 0.20 M in substrate). The residue was purified by column chromatography (0–5% EtOAc/hexanes) to afford the title compound as a yellow oil (26 mg, 80. μmol, 80% yield). **TLC R**_**f**_ = 0.6 (5% EtOAc/hexanes, CAM stain); ^**1**^**H NMR** (400 MHz, CDCl_3_) δ 7.04 (ad, *J* = 8.4 Hz, 2H), 6.76 (ad, *J* = 8.4 Hz, 2H), 3.37 (t, *J* = 6.7 Hz, 2H), 2.70 (t, *J* = 7.3 Hz, 2H), 2.12 (quint, *J* = 7.1 Hz, 2H), 0.98 (s, 9H), 0.18 (s, 6H); ^**13**^**C{**^**1**^**H} NMR** (125.8 MHz, CDCl_3_) δ 154.0, 133.2, 129.5 (2C), 120.1 (2C), 34.4, 33.24, 33.19, 25.8 (3C), 18.3, −4.4 (2C); **IR** (neat) 2956, 2929, 2857, 1609, 1509, 1472, 1252, 1169, 913, 838, 810, 780, 687 cm^–1^; **HRMS** (TOF MS CI+) *m*/*z*: [M]^+^ calcd for C_15_H_25_BrOSi, 328.0858; found, 328.0868. Analytical data is consistent with literature values.^51^
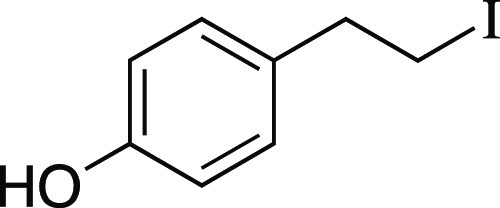


Iodide **15** was prepared according to a modified Method C. The following amounts of reagents were used: 2-(4-hydroxyphenyl) ethanol (14 mg, 0.10 mmol, 1.0 equiv), MsCl (19 μL, 0.25 mmol, 2.5 equiv), Et_3_N (35 μL, 0.25 mmol, 2.5 equiv), DCM (0.50 mL, 0.20 M in substrate), MeMgI (83 μL, 0.25 mmol, 2.5 equiv, 3.0 M in Et_2_O), PhMe (0.50 mL, 0.20 M in substrate). Mesylation was allowed to stir for 5 min. Upon addition of MeMgI, the reaction mixture was stirred at 0 °C for 5 mins, then the flask was warmed up to rt and MeMgCl (33 μL, 0.10 mmol, 1.0 equiv) was added dropwise. In the development of this reaction, we observed that subjecting an alkyl mesylate to MeMgCl resulted in conversion back to the alcohol instead of to the alkyl chloride, so to obtain the phenol as the exclusive product, we carried out this subsequent step. The reaction mixture was stirred for 1 h at rt, quenched with MeOH, filtered through a pad of silica gel eluting with 100% Et_2_O, and concentrated in vacuo. The residue was purified by column chromatography (0–20% EtOAc/hexanes) to afford the title compound as a white solid (21 mg, 85 μmol, 85% yield). **TLC R**_**f**_ = 0.4 (20% EtOAc/hexanes, CAM stain); ^**1**^**H NMR** (500 MHz, CDCl_3_) δ 7.06 (d, *J* = 8.5 Hz, 2H), 6.78 (d, *J* = 8.4 Hz, 2H), 4.63 (s, 1H), 3.31 (t, *J* = 7.8 Hz, 2H), 3.10 (t, *J* = 7.8 Hz, 2H). Analytical data is consistent with literature values.^52^
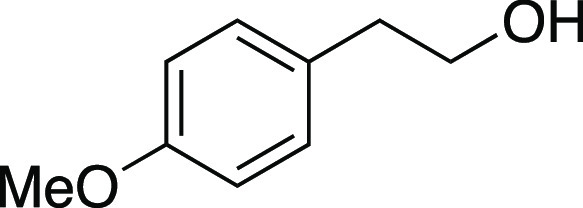


Alcohol **56** was prepared according to a modified procedure reported by Cole.^43^ Under a N_2_ atmosphere, a flame-dried round-bottom flask equipped with a stir bar was charged with 2-(4-methoxyphenyl) acetic acid (2.5 g, 15 mmol, 1.0 equiv) and THF (30. mL, 0.50 M in substrate). The reaction mixture was cooled to 0 °C and BH_3_·THF (45 mL, 45 mmol, 3.0 equiv, 1.0 M in THF) was added dropwise. The mixture was brought to rt and allowed to stir for 16 h. Then, glacial acetic acid was added dropwise until quenched, followed by the addition of sat. aqueous NaHCO_3_ until a pH of 7 was achieved. This mixture was extracted with EtOAc (×3), and the organic layers were combined, dried with Na_2_SO_4_, and concentrated in vacuo. The residue was purified by column chromatography (0–50% EtOAc/hexanes) to afford the title compound as a colorless oil (2.3 g, 15 mmol, >99% yield). **TLC R**_**f**_ = 0.2 (20% EtOAc/hexanes, KMnO_4_ stain); ^**1**^**H NMR** (400 MHz, CDCl_3_) δ 7.14 (ad, *J* = 8.4 Hz, 2H), 6.86 (ad, *J* = 8.6 Hz, 2H), 3.86–3.76 (m, 5H), 2.81 (t, *J* = 6.6 Hz, 2H), 1.41 (br s, 1H); ^**13**^**C{**^**1**^**H} NMR** (150.9 MHz, CDCl_3_) δ 158.3, 130.4, 130.0 (2C), 114.1 (2C), 63.9, 55.3, 38.3; **IR** (neat) 3348, 2935, 1612, 1512, 1464, 1300, 1244, 1178, 1110, 1037, 821 cm^–1^; **HRMS** (TOF MS CI+) *m*/*z*: [M]^+^ calcd for C_9_H_12_O_2_, 152.0837; found, 152.0831. Analytical data is consistent with literature values.^53^
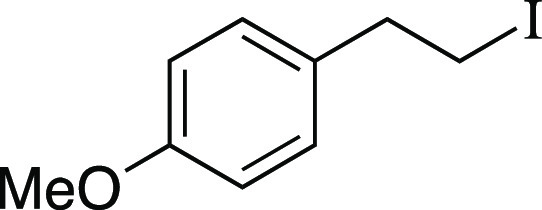


Iodide **16** was prepared according to a modified Method C. The following amounts of reagents were used: alcohol **56** (4.6 g, 30. mmol, 1.0 equiv), MsCl (3.5 mL, 45 mmol, 1.5 equiv), Et_3_N (6.3 mL, 45 mmol, 1.5 equiv), DCM (150 mL, 0.20 M in substrate), MeMgI (25 mL, 60. mmol, 2.0 equiv, 2.4 M in Et_2_O), PhMe (150 mL, 0.20 M in substrate). Commercial MeMgI was used. Upon addition of MeMgI, the reaction mixture was allowed to stir at 0 °C for 1 h. The residue was purified by column chromatography (0–5% EtOAc/hexanes) to afford the title compound as a yellow oil (6.5 g, 25 mmol, 82% yield). **TLC R**_**f**_ = 0.6 (5% EtOAc/hexanes, CAM stain); ^**1**^**H NMR** (400 MHz, CDCl_3_) δ 7.11 (ad, *J* = 8.6 Hz, 2H), 6.85 (ad, *J* = 8.7 Hz, 2H), 3.79 (s, 3H), 3.31 (at, *J* = 7.7 Hz, 2H), 3.11 (t, *J* = 7.8 Hz, 2H); ^**13**^**C{**^**1**^**H} NMR** (150.9 MHz, CDCl_3_) δ 158.5, 132.9, 129.4 (2C), 114.1 (2C), 55.3, 39.6, 6.4; **IR** (neat) 2954, 2833, 1611, 1510, 1463, 1301, 1245, 1176, 1122, 1034, 818, 754 cm^–1^; **HRMS** (TOF MS CI+) *m*/*z*: [M]^+^ calcd for C_9_H_11_IO, 261.9855; found, 261.9842. Analytical data is consistent with literature values.^54^
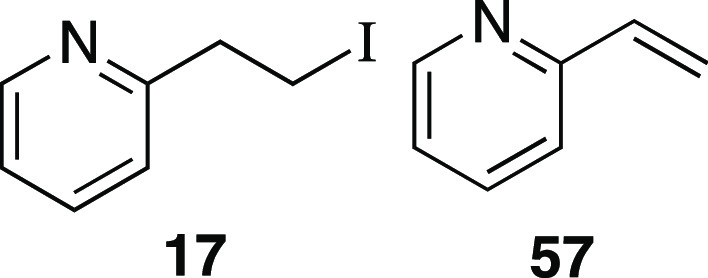


Iodide **17** was prepared according to a modified Method C. The following amounts of reagents were used: 2-pyridine-ethanol (11 μL, 0.10 mmol, 1.0 equiv), MsCl (12 μL, 0.15 mmol, 1.5 equiv), Et_3_N (21 μL, 0.15 mmol, 1.5 equiv), DCM (0.50 mL, 0.20 M in substrate), MeMgI (68 μL, 0.20 mmol, 2.0 equiv, 3.0 M in Et_2_O), PhMe (0.50 mL, 0.20 M in substrate). Upon addition of MeMgI, the reaction mixture was allowed to stir at 0 °C for 1 h. The residue was purified by column chromatography (0–25% EtOAc/hexanes) to afford the title compound as a yellow oil (19 mg, 82 μmol, 82% yield) containing alkenes **57** (0.2 mg, 2 μmol, 2%). To remove the alkenes, a modified Sharpless asymmetric dihydroxylation was performed.^30^ To a flame-dried 20 mL vial was added AD-mix-β (0.14 g, 1.4 g/mmol). Then *t*-BuOH (1.0 mL) and H_2_O (1.0 mL) were added via syringe and the vial was capped. The vial was cooled to 0 °C and then the mixture of iodide **17** and alkenes **57** was added dropwise as a solution in *t*-BuOH (1.0 mL) and H_2_O (1.0 mL) via syringe. The mixture was allowed to stir at 0 °C for 24 h. To quench, Na_2_SO_3_ (30. mg) was added and the mixture was allowed to warm to rt and stir for 30 min. Then the mixture was transferred to a separatory funnel, and the organic layer was extracted with EtOAc (×3). The combined organic layers were washed with brine, dried over Na_2_SO_4_, filtered, and concentrated in vacuo. The residue was purified by column chromatography (0–25% EtOAc/hexanes) to afford the title compound as a yellow oil (17 mg, 73 μmol, 73% yield over 2 steps). **TLC R**_**f**_ = 0.3 (25% EtOAc/hexanes, KMnO_4_ stain); ^**1**^**H NMR** (500 MHz, CDCl_3_) δ 8.58 (ad, *J* = 4.8 Hz, 1H), 7.68 (td, *J* = 7.7, 1.7 Hz, 1H), 7.25–7.19 (m, 2H), 3.56 (t, J = 7.2 Hz, 2H), 3.39 (t, *J* = 7.2 Hz, 2H); ^**13**^**C{**^**1**^**H} NMR** (125.8 MHz, CDCl_3_) δ 159.7, 149.7, 136.5, 123.3, 121.9, 42.0, 4.1; **IR** (neat) 2921, 2850, 1591, 1569, 1473, 1436, 1241, 1167, 1149, 1050, 994, 767, 751 cm^–1^; **HRMS** (TOF MS ES+) *m*/*z*: [M + H]^+^ calcd for C_7_H_8_INH, 233.9780; found, 233.9785.
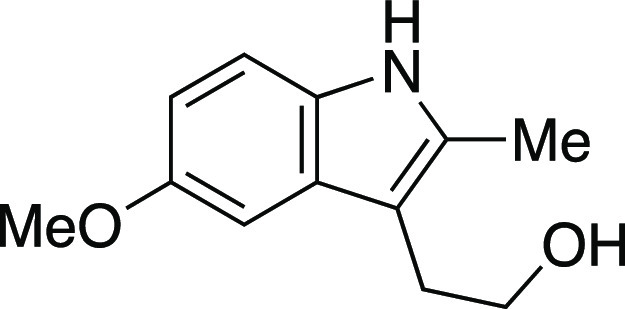


Alcohol **58** was prepared according to a modified procedure reported by Cole.^43^ Under a N_2_ atmosphere, a flame-dried round-bottom flask equipped with a stir bar was charged with indomethacin (0.36 g, 1.0 mmol, 1.0 equiv) and THF (2.0 mL, 0.50 M in substrate). The reaction mixture was cooled to 0 °C and BH_3_·THF (3.0 mL, 3.0 mmol, 3.0 equiv) was added dropwise. The mixture was brought to rt and allowed to stir for 16 h. Then, glacial acetic acid was added dropwise until quenched, followed by the addition of sat. aqueous NaHCO_3_ until a pH of 7 was achieved. This mixture was extracted with EtOAc (×3), and the organic layers were combined, dried with Na_2_SO_4_, and concentrated in vacuo. The residue was purified by flash column chromatography (0–30% EtOAc/hexanes) to afford the title compound as a brown oil (23 mg, 0.11 mmol, 11%). **TLC R**_**f**_ = 0.2 (50% EtOAc/hexanes); ^**1**^**H NMR** (500 MHz, CDCl_3_) δ 7.70 (br s, 1H), 7.16 (d, *J* = 8.7 Hz, 1H), 6.97 (ad, *J* = 2.2 Hz, 1H), 6.78 (dd, *J* = 8.7, 2.4 Hz, 1H), 3.85 (s, 3H), 3.85–3.82 (m, 2H), 2.95 (t, *J* = 6.5 Hz, 2H), 2.39 (s, 3H), 1.43 (br s, 1H); **HRMS** (TOF MS CI+) *m*/*z*: [M + H]^+^ calculated for C_12_H_15_NO_2_H 206.1181, found 206.1183. Analytical data is consistent with literature values.^55^
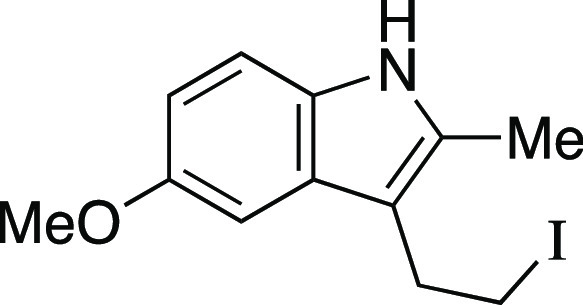


Iodide **18** was prepared according to Method C. The following amounts of reagents were used: alcohol **58** (23 mg, 0.11 mmol, 1.0 equiv), MsCl (20. μL, 0.28 mmol, 2.5 equiv), Et_3_N (40. μL, 0.28 mmol, 2.5 equiv), DCM (0.56 mL, 0.20 M in substrate), followed by MeMgI (0.10 mL, 0.28 mmol, 2.5 equiv, 2.9 M in Et_2_O), and PhMe (0.56 mL, 0.20 M in substrate). The residue was purified by flash column chromatography (0–30% EtOAc/hexanes) to afford the title compound as a bright yellow oil (18 mg, 56 μmol, 50%). **TLC R**_**f**_ = 0.4 (20% EtOAc/hexanes); ^**1**^**H NMR** (500 MHz, CDCl_3_) δ 7.71 (br s, 1H), 7.16 (d, *J* = 8.7 Hz, 1H), 6.91 (br s, 1H), 6.78 (d, *J* = 8.7 Hz, 1H), 3.86 (s, 3H), 3.36–3.23 (m, 4H), 2.37 (s, 3H); ^**13**^**C{**^**1**^**H} NMR** (125.8 MHz, CDCl_3_) δ 154.2, 132.8, 130.4, 128.5, 111.4, 111.2, 110.9, 100.3, 56.2, 29.6, 12.1, 6.4; **HRMS** (TOF MS ES+) *m*/*z*: [M + H]^+^ calculated for C_12_H_14_INOH 316.0198, found 316.0202.
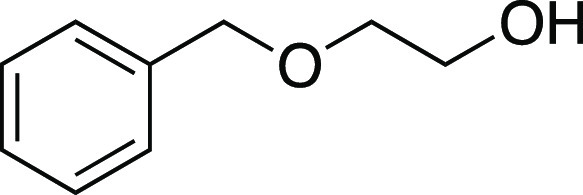


Alcohol **59** was prepared according to the following procedure. Open to air, a round-bottom flask with a stir bar was charged with benzyloxyacetaldehyde (0.70 mL, 5.0 mmol, 1.0 equiv), NaBH_4_ (0.38 g, 10. mmol, 2.0 equiv), and MeOH (25 mL, 0.20 M in substrate). The reaction mixture was stirred for 30 min. After completion, the reaction mixture was concentrated in vacuo, and then dissolved in DCM. H_2_O was added and the aqueous layer was extracted with DCM. The combined organic layers were dried and concentrated in vacuo. The residue was purified by flash column chromatography (0–50% EtOAc/hexanes) to afford the title compound as a pale-yellow oil (0.65 g, 4.2 mmol, 85%). **TLC R**_**f**_ = 0.3 (30% EtOAc/hexanes, KMnO_4_ stain); ^**1**^**H NMR** (400 MHz, CDCl_3_) δ 7.38–7.27 (m, 5H), 4.57 (s, 2H), 3.78–3.74 (m, 2H), 3.60 (at, *J* = 4.6 Hz, 2H), 2.02 (t, *J* = 6.2 Hz, 1H). Analytical data is consistent with literature values.^56^
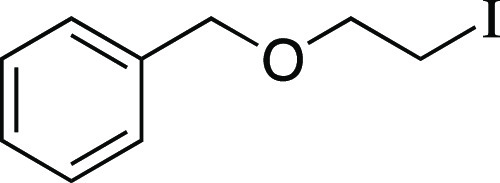


Iodide **19** was prepared according to a modified Method C. The following amounts of reagents were used: alcohol **59** (18 mg, 0.12 mmol, 1.0 equiv), MsCl (13 μL, 0.17 mmol, 1.5 equiv), Et_3_N (24 μL, 0.17 mmol, 1.5 equiv), DCM (0.58 mL, 0.20 M in substrate), followed by MeMgI (96 μL, 0.29 mmol, 2.5 equiv, 3.0 M in Et_2_O), and PhMe (0.58 mL, 0.20 M in substrate). Mesylation was allowed to stir for 6 h. Upon addition of MeMgI, the reaction mixture was allowed to stir at 0 °C for 2 h. The residue was purified by flash column chromatography (0–10% Et_2_O/hexanes) to afford the title compound as a pale-yellow oil (26 mg, 97 μmol, 84%). **TLC R**_**f**_ = 0.6 (10% EtOAc/hexanes, PMA stain); ^**1**^**H NMR** (400 MHz, CDCl_3_) δ 7.36–7.27 (m, 5H), 4.58 (s, 2H), 3.74 (t, *J* = 6.8 Hz, 2H), 3.28 (t, *J* = 6.8 Hz, 2H); ^**13**^**C{**^**1**^**H} NMR** (100 MHz, CDCl_3_) δ 137.9, 128.6 (2C), 128.0, 127.9 (2C), 73.0, 70.9, 3.0; **HRMS** (TOF MS ES+) *m*/*z*: [M]^+^ calculated for C_9_H_11_IO 261.9855, found 261.9851. Analytical data is consistent with literature values.^57^
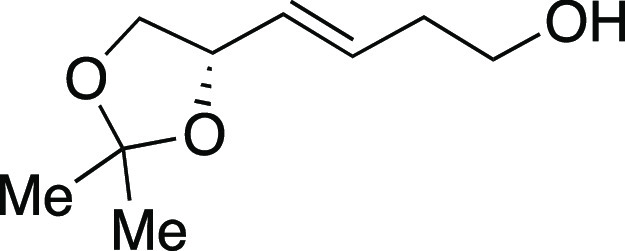


Alcohol **60** was prepared according to a procedure reported by Molander.^58^ To a flame-dried round-bottom flask equipped with a stir bar was added 3-hydroxypropyltriphenylphosphonium bromide (0.48 g, 1.2 mmol, 1.2 equiv), then THF (5.0 mL, 0.20 M in substrate). The reaction mixture was cooled to 0 °C before adding *n*-BuLi (1.3 mL, 3.2 mmol, 3.2 equiv, 2.5 M in hexanes). The reaction mixture was allowed to stir at 0 °C for 30 min. Then, 2,3-isopropylidene-glyceraldehyde (260 mg, 1.0 mmol, 1.0 equiv, 50% w/w in DCM) was added dropwise via a syringe, and the reaction mixture was allowed to stir at 0 °C rt for 16 h. To quench, sat. NH_4_Cl solution was added. The reaction mixture was extracted with EtOAc (3 ×10 mL), and the combined organic layers were washed with brine, dried over Na_2_SO_4_, and concentrated in vacuo. The residue was purified by flash column chromatography (0–30% EtOAc/hexanes) to afford the title compound as a mixture of diastereomers as a yellow oil (0.12 g, 0.71 mmol, 71%, 1.6:1 dr). **TLC R**_**f**_ = 0.2 (50% EtOAc/hexanes, KMnO_4_ stain) For clarity, the ^1^H NMR data of the major and minor diastereomers have been tabulated separately. Analytical data is consistent with literature values.^58^

##### Major Diastereomer

^**1**^**H NMR** (400 MHz, CDCl_3_) δ 5.69–5.65 (m, 1H), 5.59–5.52 (m, 1H), 4.85 (aq, *J* = 7.3 Hz, 1H), 4.11–4.06 (m, 1H), 3.65–3.53 (m, 3H), 2.54 (br s, 1H), 2.43–2.31 (m, 2H), 1.42 (s, 3H), 1.39 (s, 3H).

##### Minor Diastereomer

^**1**^**H NMR** (400 MHz, CDCl_3_) δ 5.82–5.76 (m, 1H), 5.59–5.52 (m, 1H), 4.48 (aq, *J* = 7.1 Hz, 1H), 4.11–4.06 (m, 1H), 3.65–3.53 (m, 3H), 2.54 (br s, 1H), 2.43–2.31 (m, 2H), 1.42 (s, 3H), 1.38 (s, 3H).
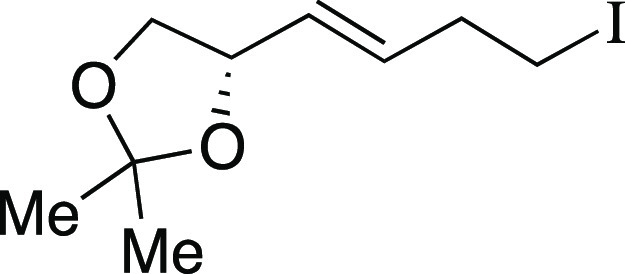


Iodide **20** was prepared according to Method C. The following amounts of reagents were used: alcohol **60** (22 mg, 0.13 mmol, 1.0 equiv), MsCl (20. μL, 0.19 mmol, 1.5 equiv), Et_3_N (30. μL, 0.19 mmol, 1.5 equiv), DCM (0.65 mL, 0.20 M in substrate), followed by MeMgI (90. μL, 0.26 mmol, 2.0 equiv, 3.0 M in Et_2_O), and PhMe (0.65 mL, 0.20 M in substrate). The residue was purified by flash column chromatography (0–50% EtOAc/hexanes) to afford the title compound as a yellow oil (21 mg, 73 μmol, 56%, 1.6:1 dr). **TLC R**_**f**_ = 0.6 (20% EtOAc/hexanes, CAM stain). For clarity, the ^1^H NMR data of the major and minor diastereomers have been tabulated separately. Analytical data is consistent with literature values.^58^

##### Major Diastereomer

^**1**^**H NMR** (400 MHz, CDCl_3_) δ 5.59–5.53 (m, 2H), 4.79 (aq, *J* = 7.0 Hz, 1H), 4.10 (aq, *J* = 7.3 Hz, 1H), 3.61–3.55 (m, 1H), 3.23–3.08 (m, 2H), 2.78–2.59 (m, 2H), 1.43 (s, 3H), 1.40 (s, 3H).

##### Minor Diastereomer

^**1**^**H NMR** (400 MHz, CDCl_3_) δ 5.77–5.70 (m, 1H), 5.59–5.53 (m, 1H), 4.49 (aq, *J* = 7.0 Hz, 1H), 4.10 (aq, *J* = 7.3 Hz, 1H), 3.61–3.55 (m, 1H), 3.23–3.08 (m, 2H), 2.78–2.59 (m, 2H), 1.43 (s, 3H), 1.39 (s, 3H).
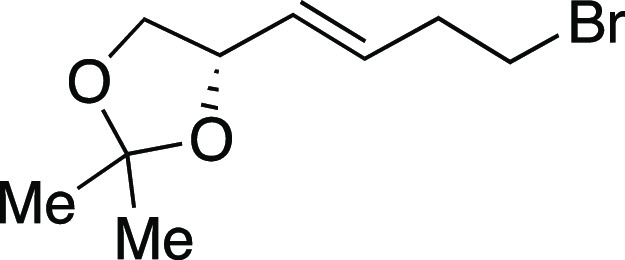


Bromide **35** was prepared according to a modified Method E. The following amounts of reagents were used: alcohol **60** (18 mg, 0.10 mmol, 1.0 equiv), MsCl (12 μL, 0.16 mmol, 1.5 equiv), Et_3_N (22 μL, 0.16 mmol, 1.5 equiv), DCM (0.52 mL, 0.20 M in substrate), followed by MeMgBr (0.12 mL, 0.31 mmol, 3.0 equiv, 2.7 M in Et_2_O), and PhMe (0.52 mL, 0.20 M in substrate). Upon addition of MeMgBr, the reaction mixture was allowed to stir at 0 °C for 1 h. The residue was purified by flash column chromatography (0–50% Et_2_O/hexanes) to afford the title compound as a mixture of diastereomers as a pale-yellow oil (13 mg, 54 μmol, 52%, 1.7:1 dr). **TLC R**_**f**_ = 0.5 (20% hexanes, PMA stain); **HRMS** (TOF MS ES+) *m*/*z*: [M + H]^+^ calculated for C_9_H_15_BrO_2_H 235.0334, found 235.0323. For clarity, the ^1^H NMR and ^13^C{^1^H} NMR data for the major and minor diastereomers have been tabulated separately.

##### Major Diastereomer

^**1**^**H NMR** (400 MHz, CDCl_3_) δ 5.66–5.54 (m, 2H), 4.81 (aq, *J* = 7.1 Hz, 1H), 4.12–4.07 (m, 1H), 3.61–3.54 (m, 1H), 3.46–3.31 (m, 2H), 2.77–2.57 (m, 2H), 1.43 (s, 3H), 1.40 (s, 3H); ^**13**^**C{**^**1**^**H} NMR** (100 MHz, CDCl_3_) δ 130.9, 130.5, 69.6, 35.6, 32.0, 31.8, 31.2, 26.9, 26.1.

##### Minor Diastereomer

^**1**^**H NMR** (400 MHz, CDCl_3_) δ 5.82–5.75 (m, 1H), 5.66–5.54 (m, 1H), 4.49 (aq, *J* = 7.1 Hz, 1H), 4.12–4.07 (m, 1H), 3.61–3.54 (m, 1H), 3.46–3.31 (m, 2H), 2.77–2.57 (m, 2H), 1.43 (s, 3H), 1.39 (s, 3H); ^**13**^**C{**^**1**^**H} NMR** (100 MHz, CDCl_3_) δ 131.5, 130.8, 72.0, 35.6, 32.0, 31.8, 31.2, 26.8, 26.0.
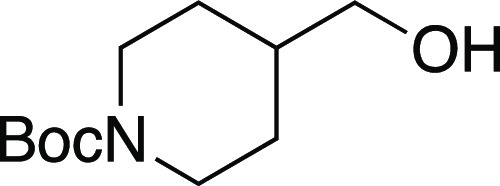


Alcohol **61** was prepared according to the following procedure. Open to air, a round-bottom flask with a stir bar was charged with N-Boc-piperidine-4-carboxaldehyde (0.21 g, 1.0 mmol, 1.0 equiv), NaBH_4_ (78 mg, 2.0 mmol, 2.0 equiv), and MeOH (5.0 mL, 0.20 M in substrate). The reaction mixture was stirred for 1.5 h. After completion, the reaction mixture was concentrated in vacuo, and then dissolved in DCM. H_2_O was added and the aqueous layer was extracted with DCM. The combined organic layers were dried and concentrated in vacuo. The residue was purified by column chromatography (0–50% EtOAc/hexanes) to afford the title compound as a white solid (0.19 g, 0.87 mmol, 87% yield). **TLC R**_**f**_ = 0.3 (50% EtOAc/hexanes); ^**1**^**H NMR** (400 MHz, CDCl_3_) δ 4.13 (as, 2H), 3.50 (t, *J* = 5.6 Hz, 2H), 2.70 (t, *J* = 12.7 Hz, 2H), 1.78–1.59 (m, 3H), 1.46 (s, 9H), 1.36 (t, *J* = 5.4 Hz, 1H), 1.15 (qd, *J* = 12.4, 4.4 Hz, 2H). Analytical data is consistent with literature values.^59^
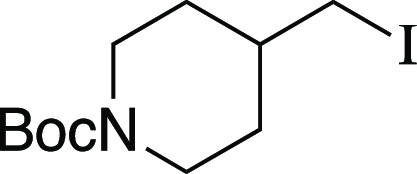


Iodide **21** was prepared according to a modified Method C. The following amounts of reagents were used: alcohol **61** (22 mg, 0.10 mmol, 1.0 equiv), MsCl (12 μL, 0.15 mmol, 1.5 equiv), Et_3_N (21 μL, 0.15 mmol, 1.5 equiv), DCM (0.50 mL, 0.20 M in substrate), MeMgI (68 μL, 0.20 mmol, 2.0 equiv, 3.0 M in Et_2_O), PhMe (0.50 mL, 0.20 M in substrate). The reaction mixture was allowed to stir for 4 h after the addition of MeMgI. The residue was purified by column chromatography (0–20% EtOAc/hexanes) to afford the title compound as a white solid (26 mg, 80. μmol, 80% yield). **TLC R**_**f**_ = 0.6 (20% EtOAc/hexanes, PMA stain); ^**1**^**H NMR** (400 MHz, CDCl_3_) δ 4.12 (as, 2H), 3.10 (d, *J* = 6.6 Hz, 2H), 2.69 (t, *J* = 12.5 Hz, 2H), 1.83 (ad, *J* = 13.5 Hz, 2H), 1.67–1.54 (m, 1H), 1.46 (s, 9H), 1.14 (qd, *J* = 12.5, 4.4 Hz, 2H). Analytical data is consistent with literature values.^60^
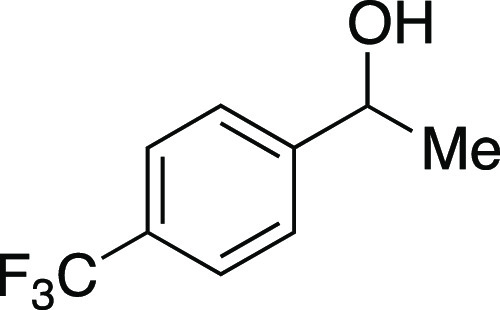


Alcohol **62** was prepared according to the following procedure. A flame-dried round-bottom flask with a stir bar was charged with 4-(trifluoromethyl)benzaldehyde (0.14 mL, 1.0 mmol, 1.0 equiv) and anhydrous THF (5.0 mL, 0.20 M in substrate), and cooled to 0 °C. Then, MeMgCl (0.50 mL, 1.5 mmol, 1.5 equiv, 3.0 M in Et_2_O) was added dropwise. The reaction mixture was allowed to stir at rt overnight. The reaction was quenched with sat. aqueous NH_4_Cl (10 mL), and the mixture was extracted with Et_2_O (3 ×20 mL). The combined organic layers were washed with brine, dried over Na_2_SO_4_, and concentrated in vacuo. The residue was purified by column chromatography (0–20% EtOAc/hexanes) to afford the title compound as a colorless oil (0.11 g, 0.59 mmol, 59% yield). **TLC R**_**f**_ = 0.3 (20% EtOAc/hexanes, CAM stain); ^**1**^**H NMR** (400 MHz, CDCl_3_) δ 7.61 (d, *J* = 8.1 Hz, 2H), 7.49 (d, *J* = 8.2 Hz, 2H), 4.97 (q, *J* = 6.5 Hz, 1H), 1.87 (br s, 1H), 1.51 (d, *J* = 6.5 Hz, 3H); ^**13**^**C{**^**1**^**H} NMR** (150.9 MHz, CDCl_3_) δ 149.7 (q, *J* = 1.1 Hz), 129.7 (q, *J* = 32.4 Hz), 125.7 (2C), 125.5 (q, *J* = 3.8 Hz, 2C), 124.2 (q, *J* = 272.5 Hz), 69.9, 25.5; ^**19**^**F NMR** (376.5 MHz, CDCl_3_) δ −62.5 (3F); **IR** (neat) 3337, 2977, 1622, 1417, 1326, 1164, 1121, 1090, 1068, 1016, 900, 842, 738 cm^–1^; **HRMS** (TOF MS ES−) *m*/*z*: [M – H]^−^ calcd for C_9_H_8_F_3_O, 189.0527; found, 189.0519. Compound **62** is commercially available: CAS 1737-26-4.
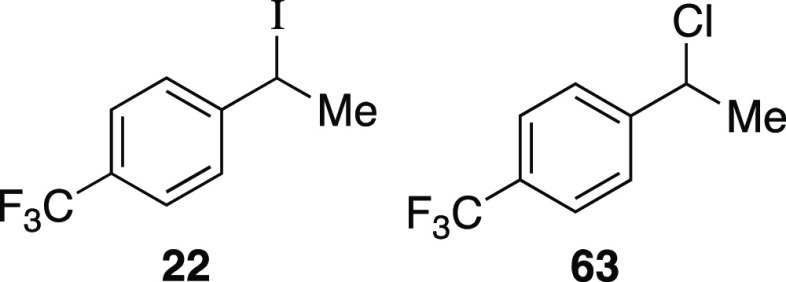


Iodide **22** was prepared according to Method C. The following amounts of reagents were used: alcohol **62** (19 mg, 0.10 mmol, 1.0 equiv), MsCl (12 μL, 0.15 mmol, 1.5 equiv), Et_3_N (21 μL, 0.15 mmol, 1.5 equiv), DCM (0.50 mL, 0.20 M in substrate), MeMgI (69 μL, 0.20 mmol, 2.0 equiv, 2.9 M in Et_2_O), PhMe (0.50 mL, 0.20 M in substrate). The residue was purified by column chromatography (0–5% EtOAc/hexanes) to afford the title compound as an orange oil (22 mg, 73 μmol, 73% yield) containing chloride **63** (2 mg, 7 μmol, 7% yield). **TLC R**_**f**_ = 0.7 (5% EtOAc/hexanes, CAM stain); ^**1**^**H NMR** (400 MHz, CDCl_3_) δ 7.58–7.50 (m, 4H), 5.36 (q, *J* = 7.1 Hz, 1H), 2.21 (d, *J* = 7.1 Hz, 3H); ^**19**^**F NMR** (376.5 MHz, CDCl_3_) δ −62.7 (3F). Analytical data is consistent with literature values.^61^
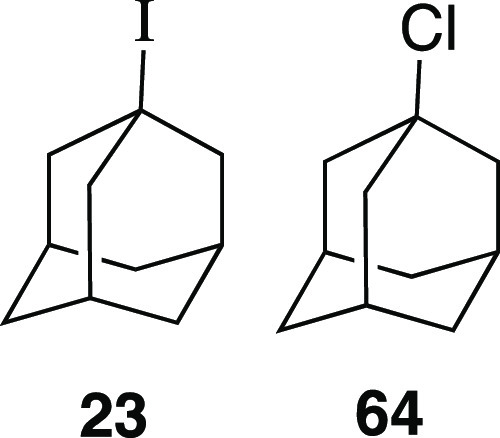


Iodide **23** was prepared according to a modified Method C. The following amounts of reagents were used: 1-adamantanol (0.15 g, 1.0 mmol, 1.0 equiv), MsCl (0.12 mL, 1.5 mmol, 1.5 equiv), Et_3_N (0.21 mL, 1.5 mmol, 1.5 equiv), DCM (5.0 mL, 0.20 M in substrate), MeMgI (0.69 mL, 2.0 mmol, 2.0 equiv, 2.9 M in Et_2_O), PhMe (5.0 mL, 0.20 M in substrate). The extraction procedure described in the method was carried out instead of a silica plug, using Et_2_O instead of DCM. The residue was purified by column chromatography (0–5% EtOAc/hexanes) to afford the title compound as a white waxy solid (0.15 g, 0.59 mmol, 59% yield) containing chloride **64** (29 mg, 0.17 mmol, 17% yield). **TLC R**_**f**_ = 0.8 (100% hexanes, CAM stain); ^**1**^**H NMR** (400 MHz, CDCl_3_) δ 2.65 (d, *J* = 2.8 Hz, 6H), 1.96 (s, 3H), 1.82 (m, 6H). Compound **23** is commercially available: CAS 768-93-4.
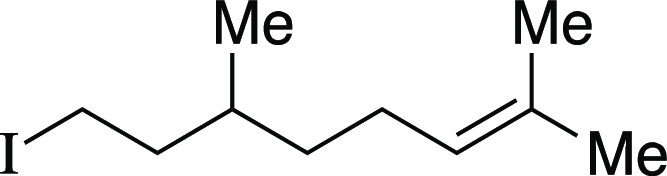


Iodide **24** was prepared according to Method C. The following amounts of reagents were used: citronellol (20. μL, 0.11 mmol, 1.0 equiv), MsCl (13 μL, 0.16 mmol, 1.5 equiv), Et_3_N (23 μL, 0.16 mmol, 1.5 equiv), DCM (0.55 mL, 0.20 M in substrate), followed by MeMgI (72 μL, 0.22 mmol, 2.0 equiv, 3.0 M in Et_2_O), and PhMe (0.55 mL, 0.20 M in substrate). The residue was purified by flash column chromatography (0–25% Et_2_O/hexanes) to afford the title compound as a colorless oil (23 mg, 87 μmol, 79%). **TLC R**_**f**_ = 0.5 (100% hexanes, PMA stain); ^**1**^**H NMR** (400 MHz, CDCl_3_) δ 5.10 (at, *J* = 7.0 Hz, 1H), 3.74–3.63 (m, 2H), 2.03–1.92 (m, 3H), 1.68 (s, 3H), 1.71–1.53 (m, 2H), 1.60 (s, 3H), 1.41–1.31 (m, 1H), 1.23–1.14 (m, 1H), 0.91 (d, *J* = 6.5 Hz, 3H). Analytical data is consistent with literature values.^62^
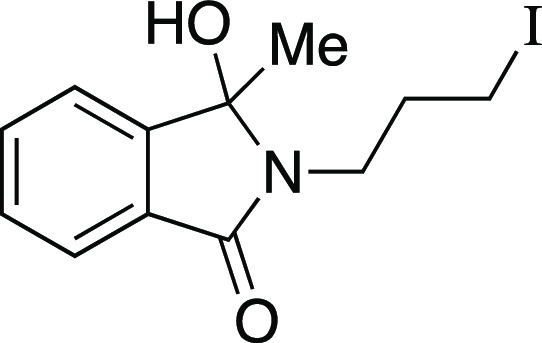


Iodide **25** was prepared according to Method C. The following amounts of reagents were used: N-(3-hydroxypropyl)phthalimide (21 mg, 0.10 mmol, 1.0 equiv), MsCl (12 μL, 0.15 mmol, 1.5 equiv), Et_3_N (21 μL, 0.15 mmol, 1.5 equiv), DCM (0.50 mL, 0.20 M in substrate), MeMgI (66 μL, 0.20 mmol, 2.0 equiv, 3.0 M in Et_2_O), PhMe (0.50 mL, 0.20 M in substrate). The residue was purified by column chromatography (0–50% EtOAc/hexanes) to afford the title compound as a yellow oil (29 mg, 87 μmol, 87% yield). **TLC R**_**f**_ = 0.6 (50% EtOAc/hexanes, CAM stain); ^**1**^**H NMR** (500 MHz, CDCl_3_) δ 7.58–7.52 (m, 3H), 7.45–7.40 (m, 1H), 3.87 (br s, 1H), 3.49 (ddd, *J* = 14.4, 9.2, 5.7 Hz, 1H), 3.21–3.11 (m, 3H), 2.29–2.19 (m, 1H), 2.15–2.03 (m, 1H), 1.69 (s, 3H); ^**13**^**C{**^**1**^**H} NMR** (125.8 MHz, CDCl_3_) δ 167.4, 148.1, 132.5, 130.1, 129.6, 123.3, 121.7, 88.8, 39.5, 32.9, 24.4, 3.1; **IR** (neat) 3306, 2925, 1675, 1616, 1470, 1407, 1373, 1199, 1141, 1092, 948, 764, 698 cm^–1^; **HRMS** (TOF MS ES+) *m*/*z*: [M + H]^+^ calcd for C_12_H_14_INO_2_H, 332.0148; found, 332.0156.
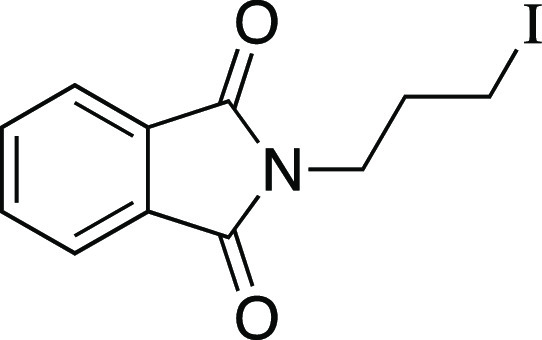


Iodide **26** was prepared according to a modified Method C. The following amounts of reagents were used: *N*-(3-hydroxypropyl)phthalimide (21 mg, 0.10 mmol, 1.0 equiv), MsCl (12 μL, 0.15 mmol, 1.5 equiv), Et_3_N (21 μL, 0.15 mmol, 1.5 equiv), DCM (0.50 mL, 0.20 M in substrate), MeMgI (66 μL, 0.20 mmol, 2.0 equiv, 3.0 M in Et_2_O), PhMe (0.50 mL, 0.20 M in substrate). Upon addition of MeMgI, the reaction mixture was allowed to stir at −78 °C for 5 min. The residue was purified by column chromatography (0–50% EtOAc/hexanes) to afford the title compound as a white solid (13 mg, 41 μmol, 41% yield). **TLC R**_**f**_ = 0.5 (25% EtOAc/hexanes, CAM stain); ^**1**^**H NMR** (400 MHz, CDCl_3_) δ 7.86 (dd, *J* = 5.4, 3.1 Hz, 2H), 7.73 (dd, *J* = 5.4, 3.1 Hz, 2H), 3.78 (t, *J* = 6.8 Hz, 2H), 3.17 (t, *J* = 7.1 Hz, 2H), 2.25 (quint, *J* = 7.0 Hz, 2H). Analytical data is consistent with literature values.^63^
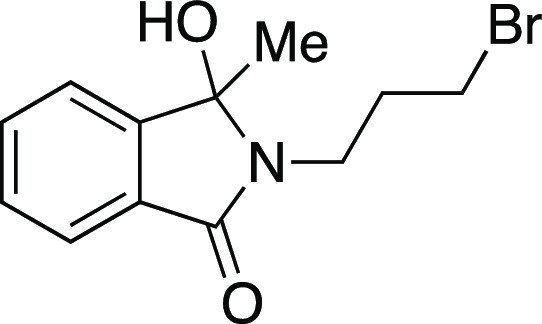


Bromide **36** was prepared according to Method E. The following amounts of reagents were used: N-(3-hydroxypropyl)phthalimide (21 mg, 0.10 mmol, 1.0 equiv), MsCl (12 μL, 0.15 mmol, 1.5 equiv), Et_3_N (21 μL, 0.15 mmol, 1.5 equiv), DCM (0.50 mL, 0.20 M in substrate), MeMgBr (0.11 mL, 0.30 mmol, 3.0 equiv, 2.7 M in Et_2_O), PhMe (0.50 mL, 0.20 M in substrate). The residue was purified by column chromatography (0–50% EtOAc/hexanes) to afford the title compound as a pale-yellow solid (24 mg, 83 μmol, 83% yield). **m.p.** 85–88 °C; **TLC R**_**f**_ = 0.4 (50% EtOAc/hexanes, KMnO_4_ stain); ^**1**^**H NMR** (500 MHz, CDCl_3_) δ 7.71 (d, *J* = 7.5 Hz, 1H), 7.61–7.55 (m, 2H), 7.50–7.46 (m, 1H), 3.69 (ddd, *J* = 14.3, 8.6, 5.8 Hz, 1H), 3.50–3.45 (m, 2H), 3.41 (ddd, *J* = 14.4, 8.5, 6.2 Hz, 1H), 2.76 (s, 1H), 2.42–2.32 (m, 1H), 2.22 (sext, *J* = 7.1 Hz, 1H), 1.74 (s, 3H); ^**13**^**C{**^**1**^**H} NMR** (125.8 MHz, CDCl_3_) δ 167.2, 147.9, 132.6, 130.4, 129.8, 123.4, 121.6, 88.8, 37.6, 32.2, 31.3, 24.3; **IR** (neat) 3311, 2926, 1678, 1470, 1409, 1374, 1141, 1095, 949, 764, 698 cm^–1^; **HRMS** (TOF MS ES+) *m*/*z*: [M + Na]^+^ calcd for C_12_H_14_BrNO_2_Na, 306.0106; found, 306.0104.
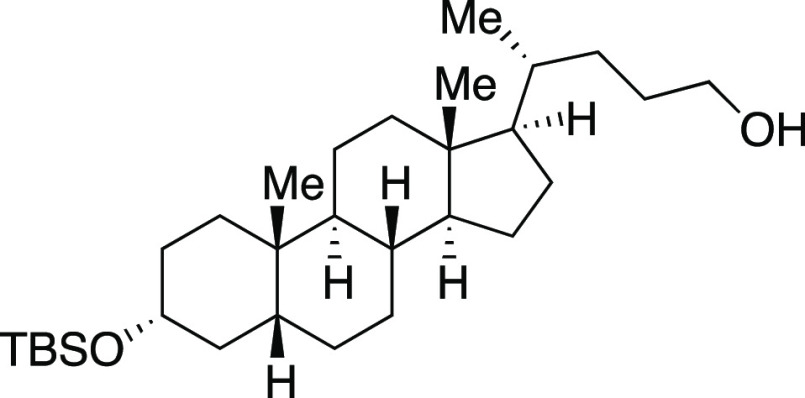


Alcohol **65** was prepared according to a procedure reported by Hu.^64^ In a glovebox, a flame-dried round-bottom flask equipped with a stir bar was added LiAlH_4_ (77 mg, 2.0 mmol, 2.6 equiv). The flask with sealed with a septum and removed from the glovebox. Under N_2_, THF (3.9 mL, 0.20 M in substrate) was added, and the reaction mixture was cooled to 0 °C. After 5 min, **SI-7** (390 mg, 0.78 mmol, 1.0 equiv) was added dropwise. The reaction mixture was allowed to stir at 0 °C for 3 h. To quench, 1 M HCl was added dropwise at 0 °C. The reaction mixture was extracted with EtOAc (3 ×20 mL), and the combined organic layers were washed with brine, dried over Na_2_SO_4_, and concentrated in vacuo. The residue was purified by flash column chromatography (0–20% EtOAc/hexanes) to afford the title compound as a white solid (310 mg, 0.65 mmol, 84%). **TLC R**_**f**_ = 0.4 (20% EtOAc/hexanes, CAM stain); ^**1**^**H NMR** (400 MHz, CDCl_3_) δ 3.63–3.54 (m, 3H), 1.97–0.89 (m, 44H), 0.64 (s, 3H), 0.06 (s, 6H). Analytical data is consistent with literature values.^64^
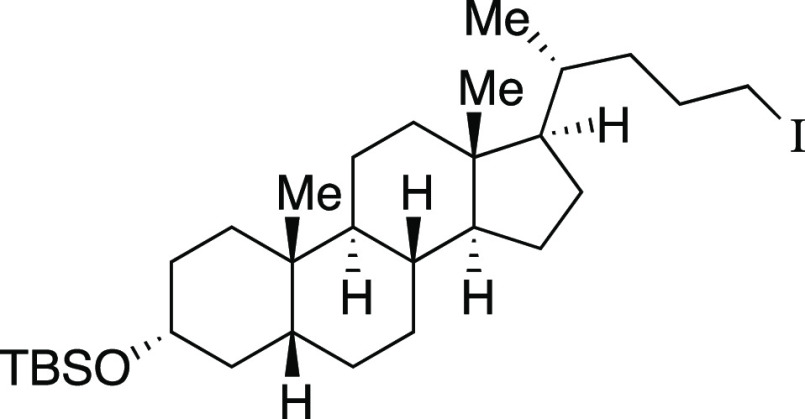


Iodide **27** was prepared according to a modified Method C. The following amounts of reagents were used: alcohol **65** (46 mg, 97 μmol, 1.0 equiv), MsCl (11 μL, 0.15 mmol, 1.5 equiv), Et_3_N (20. μL, 0.15 mmol, 1.5 equiv), DCM (0.48 mL, 0.20 M in substrate), followed by MeMgI (96 μL, 0.29 mmol, 3.0 equiv, 3.0 M in Et_2_O), and PhMe (0.48 mL, 0.20 M in substrate). The residue was purified by flash column chromatography (0–10% Et_2_O/hexanes) to afford the title compound as a white solid (48 mg, 81 μmol, 84%, 8.2% DCM by NMR). **TLC R**_**f**_ = 0.3 (100% hexanes); ^**1**^**H NMR** (400 MHz, CDCl_3_) δ 3.62–3.55 (m, 1H), 3.21–3.10 (m, 2H), 1.95–1.73 (m, 7H), 1.56–0.89 (m, 36H), 0.63 (s, 3H), 0.06 (s, 6H). Analytical data is consistent with literature values.^64^
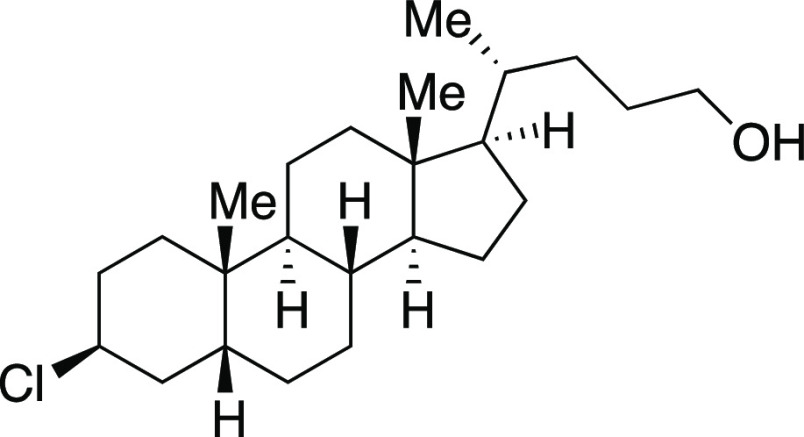


Alcohol **66** was prepared according to the following procedure. In a glovebox, a flame-dried round-bottom flask with a stir bar was charged with LiAlH_4_ (59 mg, 1.6 mmol, 2.6 equiv), capped, and brought out of the glovebox. A N_2_ inlet and THF (3.0 mL, 0.20 M in substrate) were added. The mixture was cooled to 0 °C, and ester **68** (0.25 g, 0.60 mmol, 1.0 equiv) was added as a solution in THF (1.0 M in substrate). The reaction mixture was warmed to rt and allowed to stir for 2 h. Then, sat. aqueous NH_4_Cl was added, and the crude mixture was extracted with EtOAc (3 × 20 mL). The combined organic layers were washed with brine, dried over Na_2_SO_4_, and concentrated in vacuo. The residue was purified by column chromatography (0–20% EtOAc/hexanes) to afford the title compound as a sticky white solid (0.19 g, 0.50 mmol, 83% yield). **m.p.** 39–46 °C; **TLC R**_**f**_ = 0.3 (20% EtOAc/hexanes, CAM stain); ^**1**^**H NMR** (500 MHz, CDCl_3_) δ 4.58 (as, 1H), 3.61 (m, 2H), 2.27–2.18 (m, 1H), 1.98 (ad, *J* = 12.8 Hz, 1H), 1.95–1.75 (m, 4H), 1.74–1.50 (m, 7H), 1.50–1.33 (m, 6H), 1.33–1.01 (m, 10H), 1.01–0.96 (m, 3H), 0.92 (d, *J* = 6.3 Hz, 3H), 0.65 (s, 3H); ^**13**^**C{**^**1**^**H} NMR** (125.8 MHz, CDCl_3_) δ 63.7, 61.7, 56.7, 56.3, 42.8, 40.5, 40.3, 36.6, 35.7, 35.6, 35.2, 34.6, 31.9, 30.0, 29.4, 29.0, 28.3, 26.6, 26.4, 24.2, 23.8, 21.0, 18.9, 12.1; **IR** (neat) 3331, 2926, 2863, 1444, 1376, 1278, 1056, 1014, 983, 952, 890, 712, 615, 577 cm^–1^; **HRMS** (TOF MS CI+) *m*/*z*: [M]^+^ calcd for C_24_H_41_ClO, 380.2846; found, 380.2831.
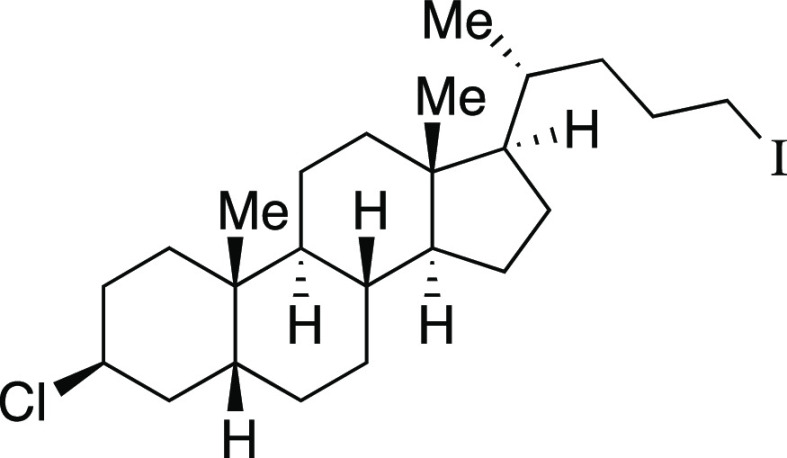


Iodide **28** was prepared according to Method C. The following amounts of reagents were used: alcohol **66** (38 mg, 0.10 mmol, 1.0 equiv), MsCl (12 μL, 0.15 mmol, 1.5 equiv), Et_3_N (21 μL, 0.15 mmol, 1.5 equiv), DCM (0.50 mL, 0.20 M in substrate), MeMgI (66 μL, 0.20 mmol, 2.0 equiv, 3.0 M in Et_2_O), PhMe (0.50 mL, 0.20 M in substrate). The residue was purified by column chromatography (100% hexanes) to afford the title compound as a yellow oil (42 mg, 86 μmol, 86% yield). **TLC R**_**f**_ = 0.4 (100% hexanes, CAM stain); ^**1**^**H NMR** (500 MHz, CDCl_3_) δ 4.58 (br s, 1H), 3.19 (dt, *J* = 9.2, 6.9 Hz, 1H), 3.12 (dt, *J* = 9.0, 7.5 Hz, 1H), 2.26–2.18 (m, 1H), 2.00–1.65 (m, 8H), 1.62–1.51 (m, 4H), 1.51–1.33 (m, 5H), 1.32–0.95 (m, 13H), 0.91 (d, *J* = 6.6 Hz, 3H), 0.65 (s, 3H); ^**13**^**C{**^**1**^**H} NMR** (125.8 MHz, CDCl_3_) δ 61.7, 56.7, 56.1, 42.8, 40.5, 40.2, 36.9, 36.6, 35.7, 35.2, 35.1, 34.6, 30.4, 30.0, 29.0, 28.4, 26.6, 26.4, 24.2, 23.8, 21.0, 18.8, 12.1, 7.8; **IR** (neat) 2926, 2861, 1444, 1376, 1278, 1251, 1236, 1171, 984, 906, 733, 711, 616 cm^–1^; **HRMS** (TOF MS CI+) *m*/*z*: [M]^+^ calcd for C_24_H_40_ClI, 490.1863; found, 490.1877.
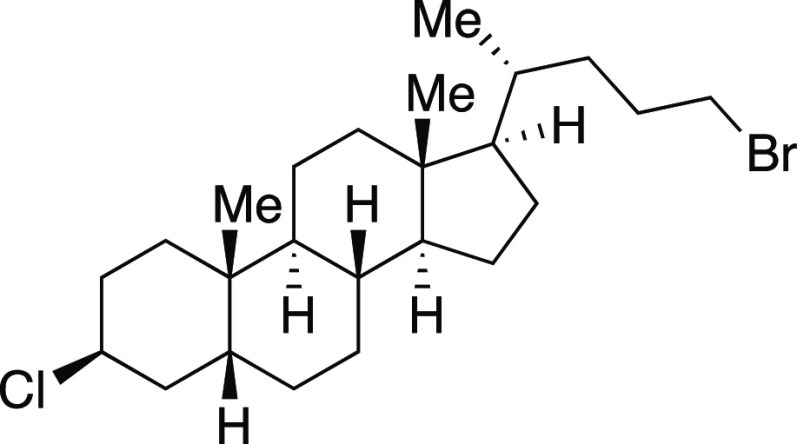


Bromide **37** was prepared according to Method E. The following amounts of reagents were used: alcohol **66** (38 mg, 0.10 mmol, 1.0 equiv), MsCl (12 μL, 0.15 mmol, 1.5 equiv), Et_3_N (21 μL, 0.15 mmol, 1.5 equiv), DCM (0.50 mL, 0.20 M in substrate), MeMgBr (0.11 mL, 0.20 mmol, 2.0 equiv, 2.7 M in Et_2_O), PhMe (0.50 mL, 0.20 M in substrate). The residue was purified by column chromatography (100% hexanes) to afford the title compound as a white solid (34 mg, 77 μmol, 77% yield). **m.p.** 59–63 °C; **TLC R**_**f**_ = 0.4 (100% hexanes, CAM stain); ^**1**^**H NMR** (500 MHz, CDCl_3_) δ 4.58 (as, 1H), 3.44–3.32 (m, 2H), 2.27–2.18 (m, 1H), 2.00–1.66 (m, 8H), 1.61–1.47 (m, 5H), 1.47–1.33 (m, 4H), 1.32–0.95 (m, 13H), 0.92 (d, *J* = 6.6 Hz, 3H), 0.65 (s, 3H); ^**13**^**C{**^**1**^**H} NMR** (125.8 MHz, CDCl_3_) δ 61.7, 56.7, 56.1, 42.8, 40.4, 40.2, 36.6, 35.7, 35.26, 35.25, 34.64, 34.55, 34.5, 30.0, 29.6, 28.7, 28.3, 26.6, 26.4, 24.2, 23.8, 21.0, 18.7, 12.1; **IR** (neat) 2928, 2863, 1444, 1377, 1278, 1253, 984, 712, 615 cm^–1^; **HRMS** (TOF MS CI+) *m*/*z*: [M]^+^ calcd for C_24_H_40_ClBr, 442.2002; found, 442.2011.
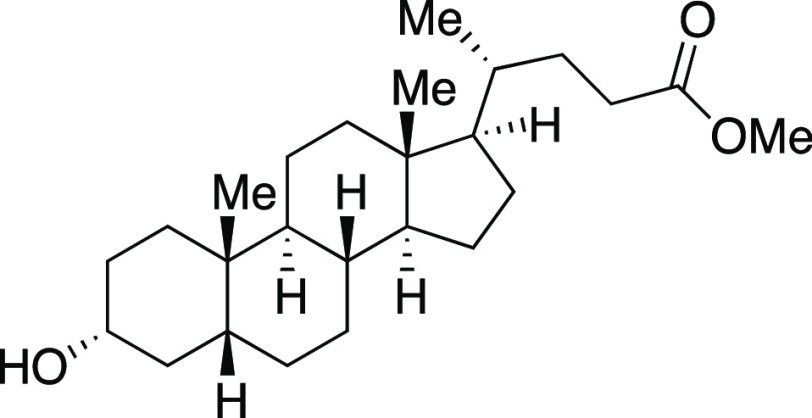


Alcohol **67** was prepared according to a modified procedure reported by Huo.^65^ Under a N_2_ atmosphere, a round-bottom flask with a stir bar was charged with lithocholic acid (1.9 g, 5.0 mmol, 1.0 equiv) and MeOH (17 mL, 0.30 M in substrate). H_2_SO_4_ (0.82 mL, 15 mmol, 3.0 equiv) was quickly added, and then the flask was equipped with a reflux condenser and heated to reflux in an oil bath. The reaction mixture was allowed to stir for 3 h before cooling and quenching dropwise with sat. aqueous NaHCO_3_ until the bubbling stopped. The reaction mixture was extracted with DCM (×3), dried with Na_2_SO_4_, and concentrated in vacuo. The residue was purified by column chromatography (0–30% EtOAc/hexanes) to afford the title compound as a white solid (1.7 g, 4.5 mmol, 89% yield). **TLC R**_**f**_ = 0.4 (30% EtOAc/hexanes, KMnO_4_ stain); ^**1**^**H NMR** (400 MHz, CDCl_3_) δ 3.66 (s, 3H), 3.66–3.57 (m, 1H), 2.35 (ddd, *J* = 15.4, 10.3, 5.2 Hz, 1H), 2.27–2.17 (m, 1H), 1.95 (ad, *J* = 11.9 Hz, 1H), 1.91–1.70 (m, 5H), 1.66 (ad, *J* = 12.9 Hz, 1H), 1.61–1.47 (m, 2H), 1.46–0.85 (m, 24H), 0.64 (s, 3H). Analytical data is consistent with literature values.^66^
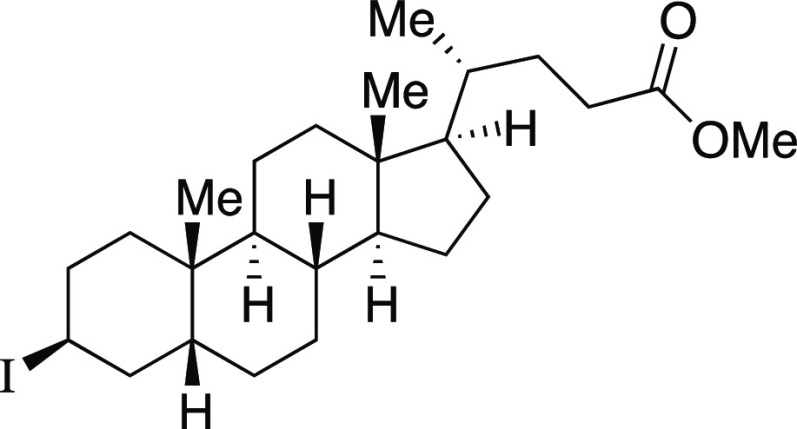


Iodide **29** was prepared according to a modified Method C. The following amounts of reagents were used: alcohol **67** (0.16 g, 0.40 mmol, 1.0 equiv), MsCl (47 μL, 0.60 mmol, 1.5 equiv), Et_3_N (84 μL, 0.60 mmol, 1.5 equiv), DCM (2.0 mL, 0.20 M in substrate), MeMgI (0.27 mL, 0.80 mmol, 2.0 equiv, 3.0 M in Et_2_O), PhMe (2.0 mL, 0.20 M in substrate). Upon addition of MeMgI, the reaction mixture was allowed to stir at 0 °C for 1 h. The residue was purified by column chromatography (0–10% EtOAc/hexanes) to afford the title compound as a white solid (0.17 g, 0.33 mmol, 83% yield). **TLC R**_**f**_ = 0.4 (10% EtOAc/hexanes, CAM stain); ^**1**^**H NMR** (500 MHz, CDCl_3_) δ 5.00 (as, 1H), 3.66 (s, 3H), 2.35 (ddd, *J* = 15.5, 10.3, 5.3 Hz, 1H), 2.21 (ddd, J = 16.2, 9.9, 6.5 Hz, 1H), 2.04–1.71 (m, 7H), 1.70–1.46 (m, 9H), 1.46–1.17 (m, 6H), 1.16–0.86 (m, 10H), 0.65 (s, 3H). Analytical data is consistent with literature values.^67^
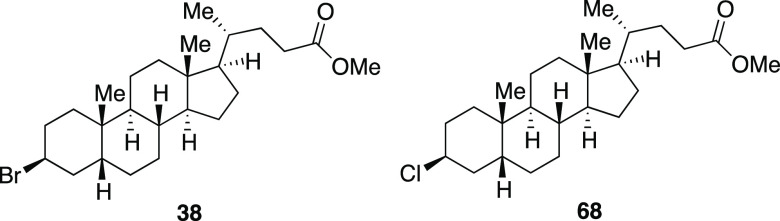


Bromide **38** was prepared according to a modified Method E. The following amounts of reagents were used: alcohol **67** (39 mg, 0.10 mmol, 1.0 equiv), MsCl (12 μL, 0.15 mmol, 1.5 equiv), Et_3_N (21 μL, 0.15 mmol, 1.5 equiv), DCM (0.50 mL, 0.20 M in substrate), MeMgBr (73 μL, 0.20 mmol, 2.0 equiv, 2.7 M in Et_2_O), PhMe (0.50 mL, 0.20 M in substrate). Mesylation was allowed to stir for 30 min. Upon addition of MeMgBr, the reaction mixture was allowed to stir at 0 °C for 2 h. The residue was purified by column chromatography (0–10% EtOAc/hexanes) to afford the title compound as a white solid (29 mg, 63 μmol, 63% yield) containing chloride **68** (2.3 mg, 6.0 μmol, 6% yield). **TLC R**_**f**_ = 0.5 (10% EtOAc/hexanes, CAM stain); ^**1**^**H NMR** (500 MHz, CDCl_3_) δ 4.80 (aquint, *J* = 2.8 Hz, 1H), 3.66 (s, 3H), 2.35 (ddd, *J* = 15.5, 10.5, 5.2 Hz, 1H), 2.29–2.16 (m, 2H), 2.00–1.74 (m, 7H), 1.67–1.51 (m, 4H), 1.49–1.22 (m, 8H), 1.22–0.85 (m, 12H), 0.65 (s, 3H). Analytical data is consistent with literature values.^68^

## Supporting Information Available

Full experimental procedures and characterization data for all new compounds, as well as copies of ^1^H and ^13^C{^1^H} NMR spectra for each (PDF)
