# Supplementary material for: Halogenation Reactions of Alkyl Alcohols Employing Methyl Grignard Reagents
Source: J Org Chem. 2022 Sep 1;87(18):12352–69. doi: 10.1021/acs.joc.2c01590 (PMC9486953; doi:10.1021/acs.joc.2c01590)
Supplement: Supplementary file 1 — jo2c01590_si_001.pdf [file jo2c01590_si_001.pdf]

Supporting Information

Halogenation Reactions of Alkyl Alcohols Employing Methyl Grignard Reagents

Nadia Hirbawi,‡ Patricia C. Lin,‡ Elizabeth R. Jarvo*

Department of Chemistry, University of California, Irvine, CA 92697-2025
*Corresponding Author: erjarvo@uci.edu

Table of Contents

I. Experimental S-1
1) Control Experiments S-1
2) Optimization Data for One-Pot Reaction to Form Iodides S-2
3) Optimization Data for Bromination Reaction S-3
4) Characterization Data for Intermediates S-3
5) Synthesis of Products and Starting Materials for Scheme 4 S-7
a) Synthesis and Characterization Data for Enantioenriched Iodide S-7
b) Synthesis and Characterization Data for Tetrahydropyrans S-8
II. References for Supporting Information S-11
III. ^{1}H, ^{13}C, ^{19}F, COSY, and nOe NMR Spectra S-12

I. Experimental

1) Control Experiments

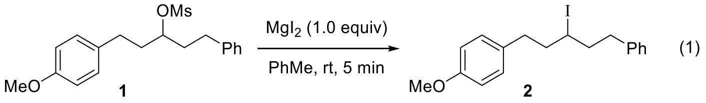

(Table 1, entry 7) To a flame-dried 7-mL vial wrapped in aluminum foil was added mesylate **1** (34 mg, 98 μmol, 1.0 equiv), MgI_2 (27 mg, 98 μmol, 1.0 equiv), PhMe (0.98 mL, 0.10 M in substrate), and a stir bar. The reaction was allowed to stir at rt for 5 min. After 5 min, the reaction mixture was filtered over a plug of silica eluting with Et_2O and concentrated in vacuo. Phenyltrimethylsilane (PhTMS; 8.6 μL, 50. μmol) was added to determine the yield by ^{1}H NMR based on comparison to PhTMS as internal standard. Iodide **2** was observed in 27% yield, with 69% recovered mesylate **1**.

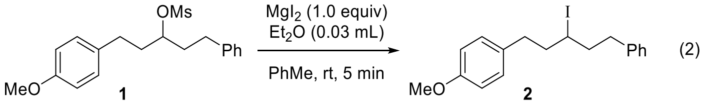

(Table 1, entry 8) To a flame-dried 7-mL vial wrapped in aluminum foil was added mesylate **1** (28 mg, 0.080 mmol, 1.0 equiv), MgI_2 (22 mg, 0.080 mmol, 1.0 equiv), Et_2O (0.03 mL), PhMe (0.80 mL, 0.10 M in substrate), and a stir bar. The reaction was allowed to stir at rt for 5 min. After 5 min, the reaction mixture was filtered over a plug of silica eluting with Et_2O and concentrated in vacuo. Phenyltrimethylsilane (PhTMS; 8.6 μL, 50. μmol) was added to determine the yield by ^{1}H NMR based on comparison to PhTMS as internal standard. Iodide **2** was observed in 69% yield, with 21% recovered mesylate **1** (6% alkenes **3**).

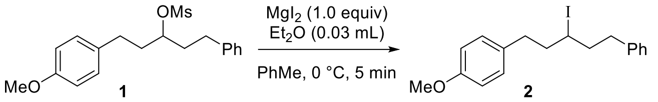

(3)

(Table 1, entry 9) To a flame-dried 7-mL vial wrapped in aluminum foil was added mesylate **1** (35 mg, 0.10 mmol, 1.0 equiv), MgI_2 (28 mg, 0.10 mmol, 1.0 equiv), Et_2O (0.03 mL), PhMe (1.0 mL, 0.10 M in substrate), and a stir bar. The reaction was allowed to stir at 0 ºC for 5 min. After 5 min, the reaction mixture was filtered over a plug of silica eluting with Et_2O and concentrated in vacuo. Phenyltrimethylsilane (PhTMS; 8.6 μL, 50. μmol) was added to determine the yield by ^{1}H NMR based on comparison to PhTMS as internal standard. Iodide **2** was observed in 6% yield, with 81% recovered mesylate **1**.

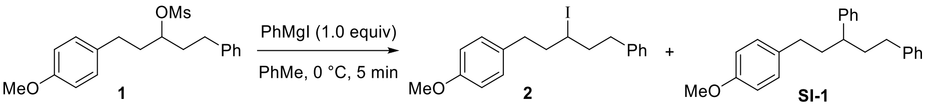

(4)

(Table 1, entry 10) Following Method B: The following amounts of reagents were added: mesylate **1** (33 mg, 0.093 mmol, 1.0 equiv), PhMgI (0.044 mL, 0.093 mmol, 1.0 equiv, 2.1 M in Et_2O), PhMe (0.93 mL, 0.10 M in substrate), and a stir bar. The reaction was allowed to stir at 0 ºC for 5 min. After 5 min, the reaction mixture was allowed to warm to rt, filtered over a plug of silica eluting with Et_2O and concentrated in vacuo. The residue was purified by flash column chromatography (0–15% Et_2O/hexanes) to afford a mixture of products: 54% iodides **2**, 24% alkenes **3**, and 14% of the phenyl-substituted product **SI-1**.

2) Optimization Data for One-Pot Reaction to Form Iodides

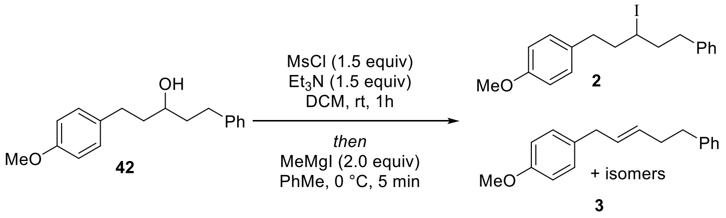

entry	deviation from standard conditions	yield **2** (%)[a]	yield **3** (%)[a]	yield OMs (%)[a]	RSM (%)[a]
**1**	**none**	**90 (91)[b]**	**6 (6)[b]**	**ND**	**<3**
2	1.0 equiv MsCl and Et_3N, 2 h mesylation	62	4	8	21
3	5 min mesylation	81	6	<3	<3
4	2 h mesylation	79	6	<3	<3
5[c]	using commercial MeMgI (2.43 M)	83 (85)[b]	6 (6)[b]	<3	<3

[a]Determined by ^{1}H NMR based on comparison to PhTMS as an internal standard.
[b]Isolated yield. [c]1 h iodination

Table S-1: Optimization of One-Pot Mesylation and Iodination

3) Optimization Data for Bromination Reaction

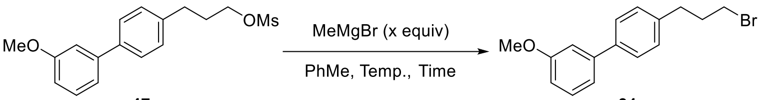

47 31

entry	MeMgBr equiv.	Temp. (°C)	Time (h)	yield **31** (%)[a]	yield OH (%)[a]	RSM (%)[a]
1	1.0	0	1	48	<3	46
2	1.0	0	24	68	12	12
3	2.0	0	1	63	<3	30
4	2.0	0	4	79	10	7
**5**	**2.0**	**25**	**1**	**77(82)[b]**	**9**	**7**
6	2.0	25	4	77	11	<3

[a]Determined by ^{1}H NMR based on comparison to PhTMS as an internal standard.
[b]Isolated yield.

Table S-2: Optimization Data for Bromination Reaction

4) Characterization Data for Intermediates

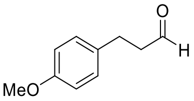

Aldehyde **SI-2** was prepared according to a modified procedure reported by Su.[1] To a flame-dried pressure tube under N_2 was added a stir bar, 4-iodoanisole (2.3 g, 10. mmol, 1.0 equiv), $Pd(OAc)_2$ (23 mg, 0.10 mmol, 0.010 equiv, 1.0 mol %), $BnEt_3NCl$ (2.28 g, 10. mmol, 1.0 equiv), and $NaHCO_3$ (2.1 g, 25 mmol, 2.5 equiv). Allyl alcohol (1.0 mL, 15 mmol, 1.5 equiv) and DMF (50. mL, 0.20 M in substrate) were added, and a Teflon screwcap was used to seal the pressure tube. The reaction mixture was allowed to stir at 50 ºC for 16 h. After cooling to rt, the reaction mixture was filtered through a pad of celite and washed with ethyl acetate (50 mL). The filtrate was concentrated using a rotary evaporator, then extracted with water (3x) and then brine. The organic layer was dried with anhydrous Na_2SO_4 and concentrated in vacuo. The residue was purified by column chromatography (0–10% EtOAc/hexanes) to afford the title compound as a colorless oil (1.24 g, 7.5 mmol, 75% yield). **TLC R_f** = 0.4 (10% EtOAc/hexanes); **^{1}H NMR** (400 MHz, $CDCl_3$) δ 9.81 (t, *J* = 1.5 Hz, 1H), 7.12 (ad, *J* = 8.6 Hz, 2H), 6.83 (ad, *J* = 8.7 Hz, 2H), 3.78 (s, 3H), 2.90 (t, *J* = 7.5 Hz, 2H), 2.74 (at, *J* = 7.6 Hz, 2H). Compound **SI-1** is commercially available: CAS 20401-88-1.

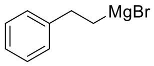

Grignard reagent **SI-3** was prepared using a modified procedure reported by Hopmann and Repo.[2] Under a N_2 atmosphere, a 2-neck round bottom flask equipped with a stir bar and reflux condenser was charged with magnesium turnings (2.2 g, 90. mmol, 1.5 equiv). The flask and magnesium turnings were placed under vacuum and flame-dried, and then back-filled with N_2. A crystal of iodine (ca. 2 mg) was added to the flask, followed by anhydrous Et_2O (20 mL). 2-bromoethylbenzene (8.2 mL, 90 mmol, 1.0 equiv) was added dropwise until reaction initiated, and then the reaction mixture was cooled to 0 °C and the remaining 2-bromoethylbenzene was added slowly over 30 min to maintain a gentle reflux. The mixture was stirred for 2 h at rt, and then transferred to a flame-dried pear-shaped flask using a syringe. The concentration of the resulting Grignard reagent was 1.6 M, as titrated by Knochel's method.[3] The Grignard reagent was stored in a –20 °C freezer and used within 1 week.

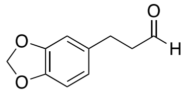

Aldehyde **SI-4** was prepared according to a modified procedure reported by Su.[4] To a flame-dried pressure tube under N_2 was added a stir bar, 5-iodo-1,3-benzodioxole (1.2 g, 5.0 mmol, 1.0 equiv), $Pd(OAc)_2$ (11 mg, 50. µmol, 1.0 mol %), $BnEt_3NCl$ (1.1 g, 5.0 mmol, 1.0 equiv), and $NaHCO_3$ (1.1 g, 12.5 mmol, 2.5 equiv). Allyl alcohol (0.85 mL, 12.5 mmol, 2.5 equiv) and DMF (25 mL, 0.20 M in substrate) were added, and a Teflon screwcap was used to seal the pressure tube. The reaction mixture was allowed to stir at 50 ºC for 16 h. After cooling to rt, the reaction mixture was filtered through a pad of celite and washed with ethyl acetate (50 mL). The filtrate was concentrated using a rotary evaporator, then extracted with water (3x) and then brine. The organic layer was dried with anhydrous Na_2SO_4 and concentrated in vacuo. The residue was purified by flash column chromatography (0–20% EtOAc/hexanes) to afford the title compound as a pale-yellow oil (0.78 g, 4.4 mmol, 88%). **TLC R_f** = 0.5 (20% EtOAc/hexanes); **^{1}H NMR** (400 MHz, $CDCl_3$) δ 9.80 (s, 1H), 6.73 (d, *J* = 7.9 Hz, 1H), 6.68 (s, 1H), 6.64 (d, *J* = 7.9 Hz, 1H), 5.92 (s, 2H), 2.88 (t, *J* = 7.4 Hz, 2H), 2.73 (t, *J* = 7.4 Hz, 2H). Analytical data is consistent with literature values.[5]

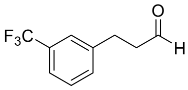

Aldehyde **SI-5** was prepared according to a modified procedure reported by Su.[6] To a flame-dried pressure tube under N_2 was added a stir bar, 3-trifluoromethyl iodide (0.43 mL, 3.0 mmol, 1.0 equiv), $Pd(OAc)_2$ (6.7 mg, 30. µmol, 0.010 equiv, 1.0 mol %), $BnEt_3NCl$ (0.68 g, 3.0 mmol, 1.0 equiv), and $NaHCO_3$ (0.63 g, 7.5 mmol, 2.5 equiv). Allyl alcohol (0.31 mL, 4.5 mmol, 1.5 equiv) and DMF (15 mL, 0.2 M in substrate) were added, and a Teflon screwcap was used to seal the pressure tube. The reaction mixture was allowed to stir at 50 ºC for 16 h. After cooling to rt, the reaction mixture was filtered through a pad of celite and washed with ethyl acetate (50 mL). The filtrate was concentrated using a rotary evaporator, then extracted with water (3x) and then brine. The organic layer was dried with anhydrous Na_2SO_4 and concentrated in vacuo. The residue was purified by column chromatography (0–10% EtOAc/hexanes) to afford the title compound as a yellow oil (0.32 g, 1.6 mmol, 53% yield). **TLC R_f** = 0.6 (20% EtOAc/hexanes, $KMnO_4$ stain); **^{1}H NMR** (400 MHz, $CDCl_3$) δ 9.83 (s, 1H), 7.50–7.35 (m, 4H), 3.02 (t, *J* = 7.5 Hz, 2H), 2.82 (t, *J* = 7.4 Hz, 2H). Analytical data are consistent with literature values.[7]

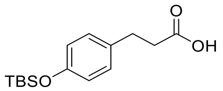

Carboxylic acid **SI-6** was prepared according to a procedure reported by Bower.[8] A flame-dried round bottom flask with a stir bar was charged with 4-hydroxyphenyl propionic acid (0.66 g, 4.0 mmol, 1.0 equiv) and DMF (8.0 mL, 0.5 M in substrate), and then cooled to 0 °C. Imidazole (0.90 g, 13 mmol, 3.3 equiv) and TBSCl (1.3 g, 8.8 mmol, 2.2 equiv) were then added. The reaction mixture was stirred at rt for at least 3 h. The reaction was quenched with H_2O, extracted with hexanes, dried over Na_2SO_4, and concentrated in vacuo. Then, K_2CO_3 (1.1 g, 8.0 mmol, 2.0 equiv), MeOH (4.0 mL, 1.0 M in substrate), and THF (4.0 mL, 1.0 M in substrate) were added. The reaction mixture was stirred for 12 h, and then quenched with aq. 1M HCl at 0 °C until a pH of 3 was reached. The crude mixture was extracted with Et_2O, dried over Na_2SO_4, and concentrated in vacuo. The residue was purified by flash column chromatography (0–30% EtOAc/hexanes) to afford the title compound as a white solid (0.29 g, 1.1 mmol, 26% yield). **TLC R$_f$** = 0.3 (20% EtOAc/hexanes); **^{1}H NMR** (400 MHz, $CDCl_3$) δ 7.05 (ad, *J* = 8.4 Hz, 2H), 6.76 (ad, *J* = 8.4 Hz, 2H), 2.89 (at, *J* = 7.8 Hz, 2H), 2.64 (at, *J* = 7.8 Hz, 2H), 0.98 (s, 9H), 0.18 (s, 6H). Analytical data are consistent with literature values.[9]

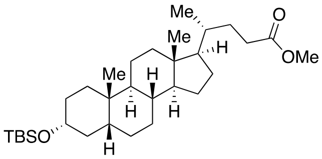

Ester **SI-7** was synthesized according to a procedure reported by Hu.[10] To a flame-dried round-bottom flask equipped with a stir bar was added DMAP (24 mg, 0.20 mmol, 0.20 equiv) and then alcohol **67** (0.40 g, 1.0 mmol, 1.0 equiv). Then, DCM (5.0 mL, 0.20 M in substrate) and NEt_3 (0.21 mL, 1.5 mmol, 1.5 equiv) were added followed by TBSCl (180 mg, 1.2 mmol, 1.2 equiv). The reaction mixture was allowed to stir for 16 h at rt. To quench, saturated NH_4Cl solution was added. The reaction mixture was extracted with DCM (3 x 20 mL), and the combined organic layers were washed with brine, dried over Na_2SO_4, and concentrated in vacuo. The residue was purified by flash column chromatography (0–10% EtOAc/hexanes) to afford the title compound as a viscous colorless oil (0.38 g, 0.77 mmol, 77%, 4.9% EtOAc by NMR). **TLC R$_f$** = 0.5 (10% EtOAc/hexanes, CAM stain); **^{1}H NMR** (400 MHz, $CDCl_3$) δ 3.75 (s, 3H), 3.71–3.63 (m, 1H), 2.48–2.40 (m, 1H), 2.34–2.26 (m, 1H), 2.08–1.82 (m, 7H), 1.66–0.99 (m, 34H), 0.73 (s, 3H), 0.15 (s, 6H). Analytical data is consistent with literature values.[11]

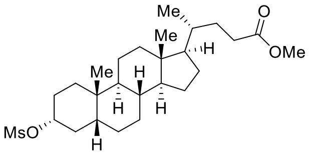

Mesylate **SI-8** was prepared according to Method A. The following amounts of reagents were used: alcohol **67** (0.59 g, 1.5 mmol, 1.0 equiv), MsCl (0.17 mL, 2.3 mmol, 1.5 equiv), Et_3N (0.31 mL, 2.3 mmol, 1.5 equiv), DCM (7.5 mL, 0.20 M in substrate). The unpurified residue was carried into the next step. **TLC R$_f$** = 0.5 (25% EtOAc/hexanes, CAM stain); **^{1}H NMR** (500 MHz, $CDCl_3$) δ 4.65 (tt, *J* = 11.5, 4.9 Hz, 1H), 3.67 (s, 3H), 3.00 (s, 3H), 2.35 (ddd, *J* = 15.5, 10.2, 5.2 Hz, 1H), 2.22 (ddd, *J* = 15.9, 9.8, 6.6 Hz, 1H), 2.05 (aq, *J* = 12.5 Hz, 1H), 1.97 (ad, *J* = 12.5 Hz, 1H), 1.91–1.75 (m, 5H), 1.75–1.68 (m, 1H), 1.68–1.56 (m, 2H), 1.50–0.97 (m, 16H), 0.95–0.89 (m, 6H), 0.64 (s, 3H). Analytical data are consistent with literature values.[12]

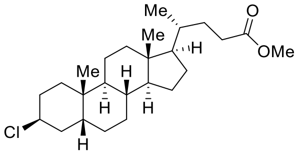

Ester **68** was prepared according to a modified procedure reported by Cahiez.[13] In a glovebox, *n*-Bu_4NCl (0.83 g, 3.0 mmol, 2.0 equiv) was added to a flame-dried round bottom flask equipped with a stir bar. The flask was sealed, removed from the glovebox, and placed under an atmosphere of N_2. In a separate flask under an atmosphere of N_2, a solution of mesylate **SI-7** (0.70 g, 1.5 mmol, 1.0 equiv) in THF (2.0 mL, 0.10 M in substrate) was prepared and transferred to the reaction flask via syringe. The reaction mixture was heated to reflux in an oil bath and allowed to stir overnight. Then, the reaction mixture was cooled to rt, quenched with H_2O, and extracted with EtOAc (x2) then hexanes. The organic layer was washed with brine, dried, and concentrated in vacuo. The residue was purified by column chromatography (0–20% EtOAc/hexanes) to afford the title compound as a white solid (0.55 g, 1.3 mmol, 89% yield over 2 steps). **m.p.** 81–84 °C; **TLC R$_f$** = 0.7 (20% EtOAc/hexanes, CAM stain); **^{1}H NMR** (500 MHz, $CDCl_3$) δ 4.58 (at, *J* = 2.7 Hz, 1H), 3.66 (s, 3H), 2.35 (ddd, *J* = 15.4, 10.3, 5.2 Hz, 1H), 2.26–2.17 (m, 2H), 2.00–1.74 (m, 6H), 1.70 (ddt, *J* = 14.6, 5.6, 3.1 Hz, 1H), 1.62–1.50 (m, 4H), 1.48–0.93 (m, 17H), 0.91 (d, *J* = 6.6 Hz, 3H), 0.65 (s, 3H); **^{13}C{^{1}H} NMR** (125.8 MHz, $CDCl_3$) δ 174.9, 61.8, 56.8, 56.1, 51.6, 42.9, 40.5, 40.3, 36.7, 35.8, 35.5, 35.3, 34.6, 31.2, 31.1, 30.1, 29.1, 28.3, 26.7, 26.5, 24.3, 23.9, 21.1, 18.4, 12.2; **IR** (neat) 2934, 2848, 1740, 1441, 1299, 1278, 1249, 1171, 110, 1017, 982, 857, 709, 613, 569 cm^{-1}; **HRMS** (TOF MS CI+) *m/z*: $[M]^+$ calcd for $C_{25}H_{41}ClO_2$, 408.2795; found, 408.2795. Analytical data are consistent with literature values.[14]

5) Synthesis of Products and Starting Materials for Scheme 4

a) Synthesis and Characterization Data for Enantioenriched Iodide

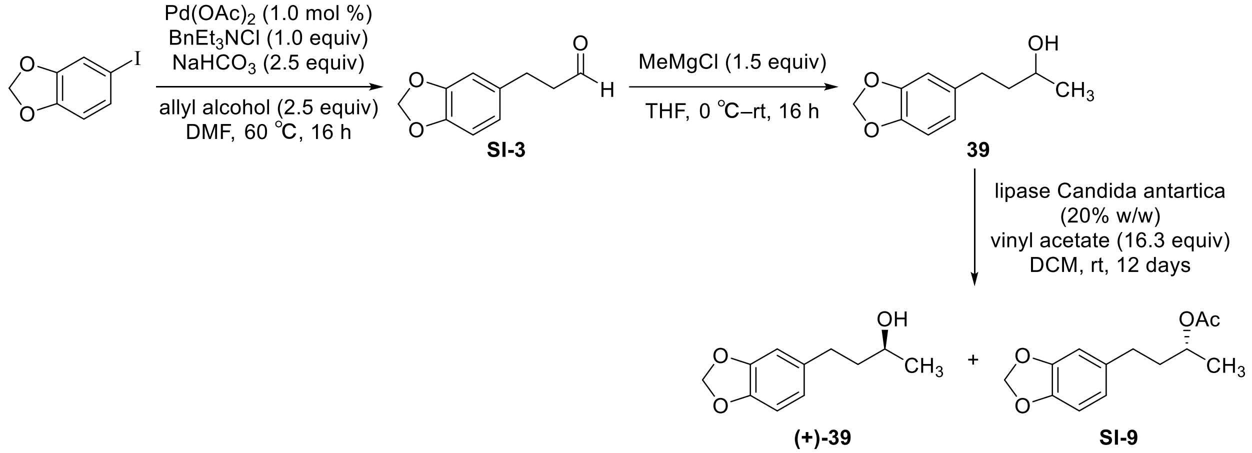

Scheme S-1: Synthesis of Alcohol (+)**-39**

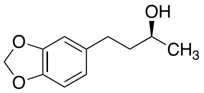

Alcohol **(+)-39** was prepared according to a procedure reported by Janda.[15] To a round-bottom flask equipped with a stir bar was added alcohol **39** (0.19 g, 1.0 mmol, 1.0 equiv), lipase from *Candida Antarctica* (0.40 g, 20% w/w), DCM (20. mL, 0.050 M in substrate), then vinyl acetate (1.5 mL, 16.3 mmol, 16.3 equiv). The reaction mixture was allowed to stir open to air for 12 days before filtering through a pad of celite and concentrated in vacuo. The residue was purified by flash column chromatography (0–30% EtOAc/hexanes) to afford the title compound as a pale-yellow oil (92 mg, 0.48 mmol, 48%). ^{1}H NMR (400 MHz, $CDCl_3$) δ 6.72 (d, *J* = 7.9 Hz, 1H), 6.69 (s, 1H), 6.65 (d, *J* = 7.9 Hz, 1H), 5.91 (s, 2H), 3.81 (br s, 1H), 2.71–2.56 (m, 2H), 1.75–1.69 (m, 2H), 1.32 (br s, 1H), 1.22 (d, *J* = 6.2 Hz, 3H). **$[\alpha]^{25}_D$** + 9.6° (c 10.8 mg/1.5 mL $CHCl_3$); **SFC Analysis** (Chiralcel AD, 1% IPA, 2.0 mL/min, 230 nm) indicated >99% ee: t_R (major enantiomer) = 34.6 min. Analytical data is consistent with literature values.[16]

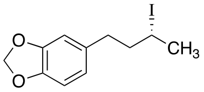

Iodide **(–)-5** was prepared according to Method C. The following amounts of reagents were used: alcohol **(+)-39** (20 mg, 97 μmol, 1.0 equiv), MsCl (11 μL, 0.15 mmol, 1.5 equiv), NEt_3 (20. μL, 0.15 mmol, 1.5 equiv), DCM (0.49 mL, 0.20 M in substrate), followed by MeMgI (66 μL, 0.20 mmol, 2.0 equiv, 2.9 M in Et_2O), and PhMe (0.49 mL, 0.20 M in substrate). Upon addition of MeMgI, the reaction mixture was allowed to stir at 0 °C for 30 min. The residue was purified by flash column chromatography (0–10% Et_2O/hexanes) to afford the title compound as a pale-yellow oil (26 mg, 86 μmol, 89%). **^{1}H NMR** (600 MHz, $CDCl_3$) δ 6.73 (d, *J* = 7.9 Hz, 1H), 6.69 (s, 1H), 6.66 (d, *J* = 7.9 Hz, 1H), 5.92 (s, 2H), 4.12–4.06 (m, 1H), 2.78–2.73 (m, 1H), 2.64–2.59 (m, 1H), 2.13–2.07 (m, 1H), 1.94 (d, *J* = 6.8 Hz, 3H), 1.85–1.79 (m, 1H). **$[\alpha]^{25}_D$** –133.4° (c 7.3 mg/1.5 mL $CHCl_3$); **SFC Analysis** (Chiralcel AD, 1% IPA, 2.0 mL/min, 230 nm) indicated >99% ee: t_R (major enantiomer) = 9.3 min. Analytical data is consistent with literature values.[17]

b) Synthesis and Characterization Data for Tetrahydropyrans

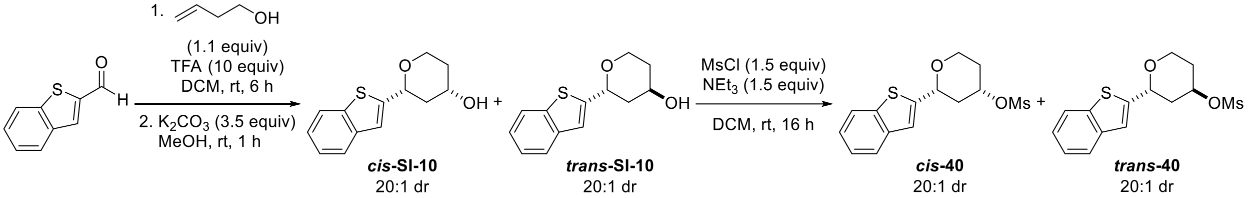

Scheme S-2: Synthesis of Mesylate **40**

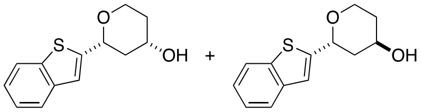

Alcohols ***cis*-SI-10** and ***trans*-SI-10** was prepared following a procedure reported by Jarvo.[18] To a flame-dried round-bottom flask equipped with a stir bar was added benzo[b]thiophene-2-carbaldehyde (0.24 g, 1.5 mmol, 1.0 equiv), 3-buten-1-ol (0.14 mL, 1.7 mmol, 1.1 equiv), and anhydrous DCM (7.5 mL, 0.20 M in substrate). Trifluoroacetic acid (1.2 mL, 15 mmol, 10. equiv) was then added dropwise, and the reaction mixture was allowed to stir at rt for 7 h. After 7 h, sat. aqueous $NaHCO_3$ was added dropwise, then the pH was adjusted to >7 by addition of NEt_3. The aqueous layer was extracted with DCM (x 3), and the combined organic layers were washed with brine, dried over Na_2SO_4, and filtered and concentrated in vacuo to yield a residue. The residue was dissolved in MeOH (7.5 mL, 0.20 M in substrate), and K_2CO_3 (0.73 g, 5.3 mmol, 3.5 equiv) was added. The reaction mixture was allowed to stir for 1 h at rt. After 1 h, the reaction mixture was concentrated in vacuo to remove MeOH, and the residue was diluted with H_2O and extracted with DCM (x 3). The combined organic layers were washed with brine, dried over Na_2SO_4, and concentrated in vacuo. The residue was purified by flash column chromatography (0–30% EtOAc/hexanes) to afford the title compound as two diastereomers as white solids. The dr was determined based on the integration of the benzylic methines in the ^1H NMR spectrum. For clarity, the yields, ^1H NMR, and $^{13}C\{^1H\}$ NMR data of each diastereomer have been tabulated individually.

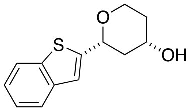

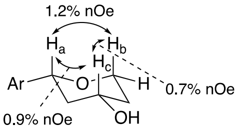

Alcohol ***cis*-SI-10** was isolated as a white solid (60. mg, 17%, >20:1 dr). Irradiation of the benzylic proton (H_a) gave a 0.9% nOe enhancement of the proton geminal to the alcohol (H_c), indicating a cis relationship. **m.p.** 131–133 °C; **TLC R$_f$** = 0.2 (40% EtOAc/hexanes, $KMnO_4$ stain); **^{1}H NMR** (500 MHz, $CDCl_3$) δ 7.81 (d, *J* = 7.7 Hz, 1H), 7.72 (d, *J* = 7.5 Hz, 1H), 7.34–7.28 (m, 2H), 7.19 (s, 1H), 4.67 (d, *J* = 11.3 Hz, 1H), 4.20 (dd, *J* = 11.8, 4.6 Hz, 1H), 4.00–3.94 (m, 1H), 3.63 (at, *J* = 12.2 Hz, 1H), 2.40 (dd, *J* = 12.4, 2.1 Hz, 1H), 2.00 (ad, *J* = 12.5 Hz, 1H), 1.78–1.64 (m, 3H); **^{13}C{^{1}H} NMR** (125.7 MHz, $CDCl_3$) δ 145.9, 139.50, 139.48, 124.4, 124.3, 123.7, 122.5, 120.0, 74.7, 68.1, 66.6, 43.0, 35.5; **HRMS** (TOF MS CI+) *m/z*: $[M]^+$ calculated for $C_{13}H_{14}O_2S$ 234.0715, found 234.0718.

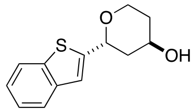

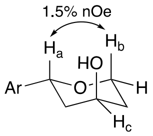

Alcohol ***trans*-SI-10** was isolated as a pale-yellow oil (22 mg, 6%, >20:1 dr). Irradiation of the benzylic proton (H_a) gave no nOe enhancement of the proton geminal to the alcohol (H_c), indicating a trans relationship. **TLC R_f** = 0.3 (40% EtOAc/hexanes, $KMnO_4$ stain); **^{1}H NMR** (500 MHz, $CDCl_3$) δ 7.81 (d, *J* = 7.9 Hz, 1H), 7.71 (d, *J* = 7.8 Hz, 1H), 7.32 (at, *J* = 7.2 Hz, 1H), 7.28 (at, *J* = 6.9 Hz, 1H), 7.17 (s, 1H), 5.18 (dd, *J* = 7.9, 5.4 Hz, 1H), 4.36–4.35 (m, 1H), 4.10 (td, *J* = 11.8, 2.4 Hz, 1H), 3.95 (ddd, *J* = 11.6, 5.0, 2.5 Hz, 1H), 2.10–2.08 (m, 2H), 2.03–1.97 (m, 1H), 1.66 (dd, *J* = 14.0, 2.2 Hz, 1H), 1.58 (br s, 1H); **^{13}C{^{1}H} NMR** (125.8 MHz, $CDCl_3$) δ 147.0, 139.6, 139.5, 124.3, 124.2, 123.6, 122.5, 120.0, 70.6, 64.1, 63.0, 40.4, 33.1.

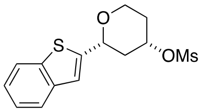

Mesylate ***cis*-40** was prepared according to Method A. The following amounts of reagents were used: ***cis*-SI-10** (20 mg, 85 μmol, 1.0 equiv), MsCl (10. μL, 0.13 mmol, 1.5 equiv), NEt_3 (20. μL, 0.13 mmol, 1.5 equiv), and DCM (0.43 mL, 0.20 M in substrate). The residue was purified by flash column chromatography (0–30% EtOAc/hexanes) to afford the title compound as a white solid (18 mg, 58 μmol, 67%). **m.p.** 115–118 °C; **TLC R_f** = 0.2 (30% EtOAc/hexanes); **^{1}H NMR** (400 MHz, $CDCl_3$) δ 7.81 (d, *J* = 7.5 Hz, 1H), 7.72 (d, *J* = 6.9 Hz, 1H), 7.36–7.29 (m, 2H), 7.20 (s, 1H), 5.02–4.94 (m, 1H), 4.73 (d, *J* = 11.4 Hz, 1H), 4.24 (ddd, *J* = 12.1, 5.0, 1.7 Hz, 1H), 3.68 (td, *J* = 12.3, 2.1 Hz, 1H), 3.06 (s, 3H), 2.60–2.56 (m, 1H), 2.20–2.15 (m, 1H), 2.07–1.92 (m, 2H); **^{13}C{^{1}H} NMR** (100. MHz, $CDCl_3$) δ 144.6, 139.5, 139.4, 124.53, 124.50, 123.8, 123.1, 122.6, 120.4, 74.5, 66.1, 40.4, 39.2, 33.0; **HRMS** (TOF MS CI+) *m/z*: $[M]^+$ calculated for $C_{14}H_{16}O_4S$, 312.0490; found 312.0482.

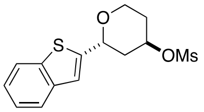

Mesylate ***trans*-40** was prepared according to Method A. The following amounts of reagents were used: ***trans*-SI-10** (22 mg, 94 μmol, 1.0 equiv), MsCl (11 μL, 0.14 mmol, 1.5 equiv), NEt_3 (20. μL, 0.14 mmol, 1.5 equiv), and DCM (0.47 mL, 0.20 M in substrate). The residue was purified by flash column chromatography (0–30% EtOAc/hexanes) to afford the title compound as a colorless oil (22 mg, 70. μmol, 75%). **TLC R_f** = 0.3 (30% EtOAc/hexanes); **^{1}H NMR** (400 MHz, $CDCl_3$) δ 7.81 (d, *J* = 7.7 Hz, 1H), 7.72 (d, *J* = 6.5 Hz, 1H), 7.36–7.28 (m, 2H), 7.20 (s, 1H), 5.25–5.24 (m, 1H), 5.13 (d, *J* = 10.8 Hz, 1H), 4.05 (dd, *J* = 8.2, 2.8 Hz, 2H), 3.10 (s, 3H), 2.41–2.38 (m, 1H), 2.27–1.99 (m, 3H).

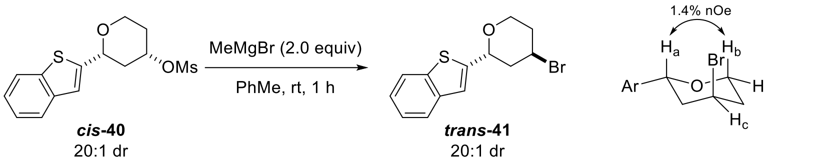

(23)

Bromide ***trans*-41** was prepared according to Method D. The following amounts of reagents were used: mesylate ***cis*-40** (18 mg, 58 µmol, 1.0 equiv), MeMgBr (40. µL, 0.12 mmol, 2.0 equiv, 2.7 M in Et_2O), and PhMe (0.58 mL, 0.10 M in substrate). The reaction mixture was allowed to stir at rt for 1 h. The residue was purified by flash column chromatography (0–30% Et_2O/hexanes) to afford the title compound as a pale-yellow oil (5 mg, 17 µmol, 29%, >20:1 dr). The dr was determined based on the integration of the benzylic methines in the ^{1}H NMR spectrum. Irradiation of the benzylic proton (H_a) gave no nOe enhancement of the proton geminal to the bromide (H_c), indicating a trans relationship. **TLC R_f** = 0.7 (20% EtOAc/hexanes); **^{1}H NMR** (500 MHz, $CDCl_3$) δ 7.81 (d, *J* = 7.8 Hz, 1H), 7.72 (d, *J* = 7.3 Hz, 1H), 7.31 (aquint, *J* = 7.8 Hz, 2H), 7.19 (s, 1H), 5.24 (d, *J* = 9.9 Hz, 1H), 4.79 (br s, 1H), 4.16 (td, *J* = 17.4, 1.9 Hz, 1H), 4.04 (ad, *J* = 11.7 Hz, 1H), 2.42–2.21 (m, 3H), 2.00 (d, *J* = 14.6 Hz, 1H); **^{13}C{^{1}H} NMR** (125.7 MHz, $CDCl_3$) δ 145.8, 139.53, 139.51, 124.4, 124.3, 123.7, 122.5, 120.3, 71.3, 63.7, 49.1, 41.5, 34.1; **HRMS** (TOF MS CI+) *m/z*: $[M]^+$ calculated for $C_{13}H_{13}BrOS$ 295.9871, found 295.9859.

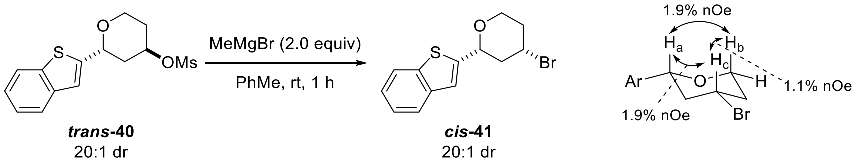

(24)

Bromide ***cis*-41** was prepared according to Method D. The following amounts of reagents were used: mesylate ***trans*-40** (22 mg, 70 µmol, 1.0 equiv), MeMgBr (50. µL, 0.14 mmol, 2.0 equiv, 2.7 M in Et_2O), and PhMe (1.4 mL, 0.050 M in substrate). The reaction mixture was allowed to stir at rt for 1 h. The residue was purified by flash column chromatography (0–30% Et_2O/hexanes) to afford the title compound as a white solid (5 mg, 17 µmol, 24%, >20:1 dr). The dr was determined based on the integration of the benzylic methines in the ^{1}H NMR spectrum. Irradiation of the benzylic proton (H_a) gave a 1.9% nOe enhancement of the proton geminal to the bromide (H_c), indicating a cis relationship. **m.p.** 90–94 °C; **TLC R_f** = 0.7 (20% EtOAc/hexanes); **^{1}H NMR** (600 MHz, $CDCl_3$) δ 7.81 (d, *J* = 7.7 Hz, 1H), 7.72 (d, *J* = 7.9 Hz, 1H), 7.34 (at, *J* = 7.3 Hz, 1H), 7.31 (at, *J* = 7.5 Hz, 1H), 7.19 (s, 1H), 4.67 (d, *J* = 10.6 Hz, 1H), 4.31–4.25 (m, 1H), 4.17 (dd, *J* = 11.8, 4.7 Hz, 1H), 3.64 (td, *J* = 12.1, 1.7 Hz, 1H), 2.69–2.66 (m, 1H), 2.27 (q, *J* = 12.1 Hz, 2H), 2.19 (ddd, *J* = 24.8, 12.3, 4.9 Hz, 1H); **^{13}C{^{1}H} NMR** (150.9 MHz, $CDCl_3$) δ 144.9, 139.44, 139.40, 124.5 (2C), 123.7, 122.6, 120.3, 76.4, 68.5, 45.4, 45.0, 37.5.

II. References for Supporting Information

[1] Shang, Y.; Jie, X.; Jonnada, K.; Zafar, S. N.; Su, W. Dehydrogenative desaturation-relay via formation of multicenter-stabilized radical intermediates. *Nature Commun*. **2017**, *8*, 2273.
[2] Sahari, A.; Do, C. D.; Mannisto, J. K.; Antico, E.; Amaratunga, A.; Hopmann, K. H.; Repo, T. Titanium Isopropoxide-Mediated *Cis*-Selective Synthesis of 3,4-Substituted Butyrolactones from CO_2. *Chem. Commun.* **2022**, *58*, 3027–3030.
[3] Krasoviskiy, A.; Knochel, P. Convenient Titration Method for Organometallic Zinc, Magnesium, and Lanthanide Reagents. *Synthesis* **2006**, *5*, 890–891.
[4] See reference 1.
[5] Bellotte, P.; Glorius, F.; Heidrich, B.; Huang, H.-M.; Pflüger, P. M.; Schwarz, J. L. Three-Component, Interrupted Radical Heck/Allylic Substitution Cascade Involving Unactivated Alkyl Bromides. *J. Am. Chem. Soc*. **2020**, *142*, 10173–10183.
[6] See reference 1.
[7] Lee, J.-H.; Shin, S. C.; Seo, S. H.; Seo, Y. H.; Jeong, N.; Kim, C.-W.; Kim, E. E.; Keum, G. Synthesis and in vitro Antiproliferative Activity of C5-Benzyl Substituted 2-Amino-pyrrolo[2,3-d]pyrimidines as Potent Hsp90 Inhibitors. *Bioorg. Med. Chem. Lett.* **2017**, *27*, 237–241.
[8] Farndon, J. J.; Ma, X.; Bower, J. F. Transition Metal Free C–N Bond Forming Dearomatizations and Aryl C–H Aminations by in Situ Release of a Hydroxylamine-Based Aminating Agent. *J. Am. Chem. Soc.* **2017**, *139*, 14005–14008.
[9] See reference 8.
[10] Bera, S.; Mao, R.; Hu, X. Enantioselective C(sp^3)–C(sp^3) Cross-Coupling of Non-Activated Alkyl Electrophiles via Nickel Hydride Catalysis. *Nat. Chem*. **2021**, *13*, 270–277.
[11] See reference 10.
[12] Iida, T.; Chang, F. C. Potential Bile Acid Metabolites. 6. Stereoisomeric 3,7-Dihydroxy-5β-cholanic Acids. *J. Org. Chem.* **1982**, *47*, 2966–2972.
[13] (a) Cahiez, G.; Lefèvre, N.; Poizat, M. Moyeux, A. A User-Friendly Procedure for the Preparation of Secondary Alkyl Chlorides. *Synthesis*, **2013**, *45*, 213–216. (b) Hewitt, K. A.; Xie, P.-P. Thane, T. A.; Hirbawi, N.; Zhang, S.-Q.; Matus, A. C.; Lucas, E. L.; Hong, X.; Jarvo, E. R. Nickel-Catalyzed Domino Cross-Electrophile Coupling Dicarbofunctionalization Reaction to Afford Vinylcyclopropanes. *ACS Catal.* **2021**, *11*, 14369–14380.
[14] Begue, J. P.; Bonnet-Delpon, D. Configurational Assignment by ^{13}C NMR of Stereoisomeric 3-Bromo-3-acyl Derivatives of 5α- and 5β- Androstane. *Org. Magn. Reson.* **1982**, *18*, 190–192.
[15] Córdova, A.; Tremblay, M. R.; Clapham, B.; Janda, K. D. A Highly Chemo- and Stereoselective Synthesis of β-Keto Esters via a Polymer-Supported Lipase Catalyzed Transesterfication. *J. Org. Chem*. **2001**, *66*, 5645–5648.
[16] Roque Pena, J. E.; Alexanian, E. J. Cobalt-Catalyzed Silylcar-bonylation of Unactivated Secondary Alkyl Tosylates at Low Pressure. *Org. Lett*. **2017**, *19*, 4413–4415.
[17] Chen, Y.; Ma, G.; Gong, H. Copper-Catalyzed Reductive Trifluo-romethylation of Alkyl Iodides with Togni's Reagent. *Org. Lett*. **2018**, *20*, 4677–4680.
[18] Tollefson, E. J.; Erickson, L. W. Jarvo, E. R. Stereospecific Intramolecular Reductive Cross-Electrophile Coupling Reactions for Cyclopropane Synthesis. *J. Am. Chem. Soc.* **2015**, *137*, 9760–9763.

1H spectrum

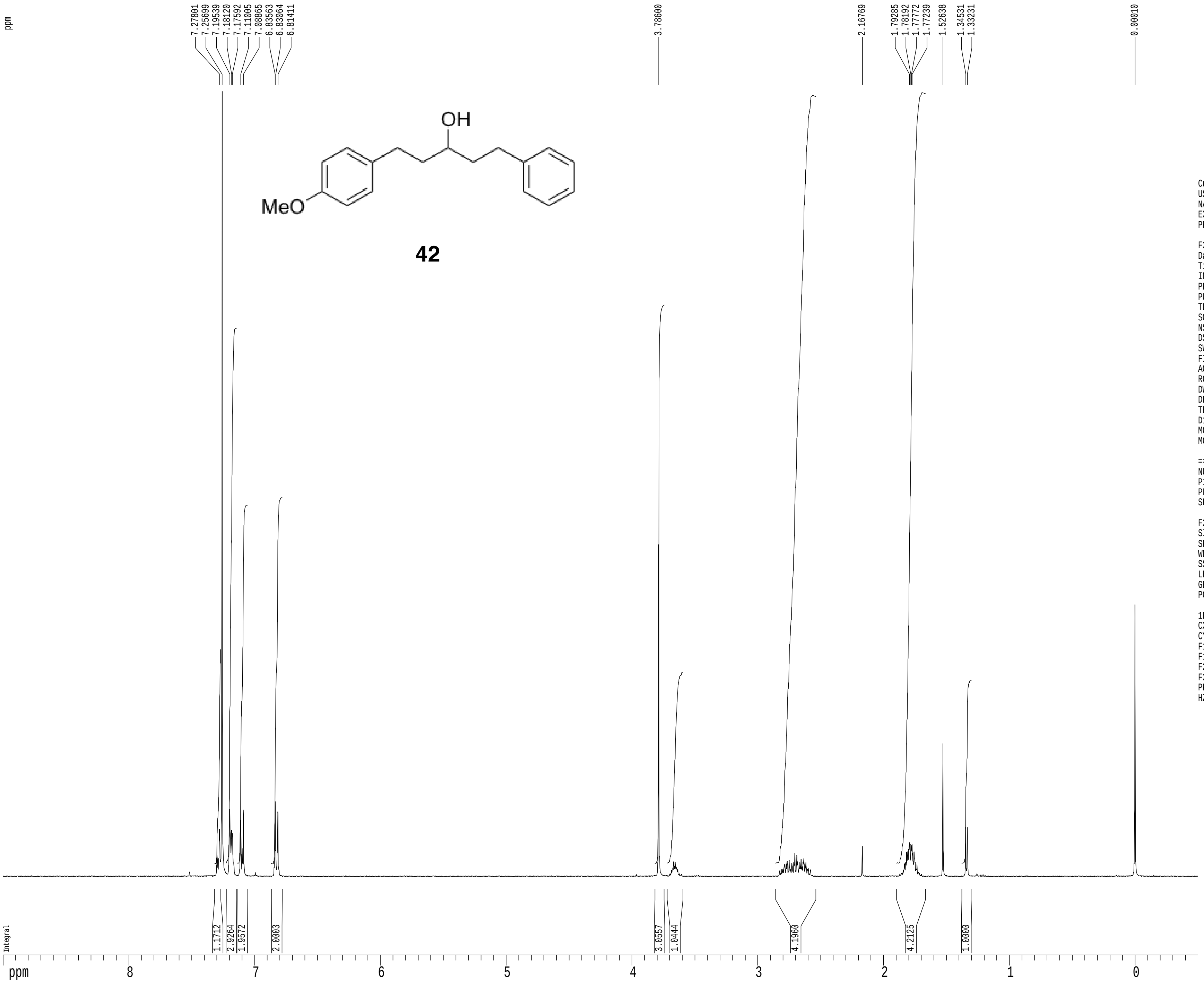


```
Current Data Parameters
USER              nhirbawi
NAME          NH-2-150-ISCO
EXPNO                    1
PROCNO                   1

F2 - Acquisition Parameters
Date_             20220118
Time                 16.55
INSTRUM             drx400
PROBHD   5 mm QNP H/F/P
PULPROG               zg30
TD                   65536
SOLVENT             CDCl3T
NS                       8
DS                       2
SWH               6410.256 Hz
FIDRES            0.097813 Hz
AQ               5.1118579 sec
RG                    1024
DW                  78.000 usec
DE                    4.50 usec
TE                   298.0 K
D1              0.10000000 sec
MCREST          0.00000000 sec
MCWRK           0.01500000 sec

======== CHANNEL f1 ========
NUC1                    1H
P1                   12.00 usec
PL1                  -0.90 dB
SFO1           400.1328009 MHz

F2 - Processing parameters
SI                   65536
SF             400.1300226 MHz
WDW                     EM
SSB                      0
LB                    0.30 Hz
GB                       0
PC                    2.00

1D NMR plot parameters
CX                   22.80 cm
CY                   15.00 cm
F1P                  9.000 ppm
F1                 3601.17 Hz
F2P                 -0.500 ppm
F2                 -200.06 Hz
PPMCM              0.41667 ppm/cm
HZCM             166.72086 Hz/cm
```

1H spectrum

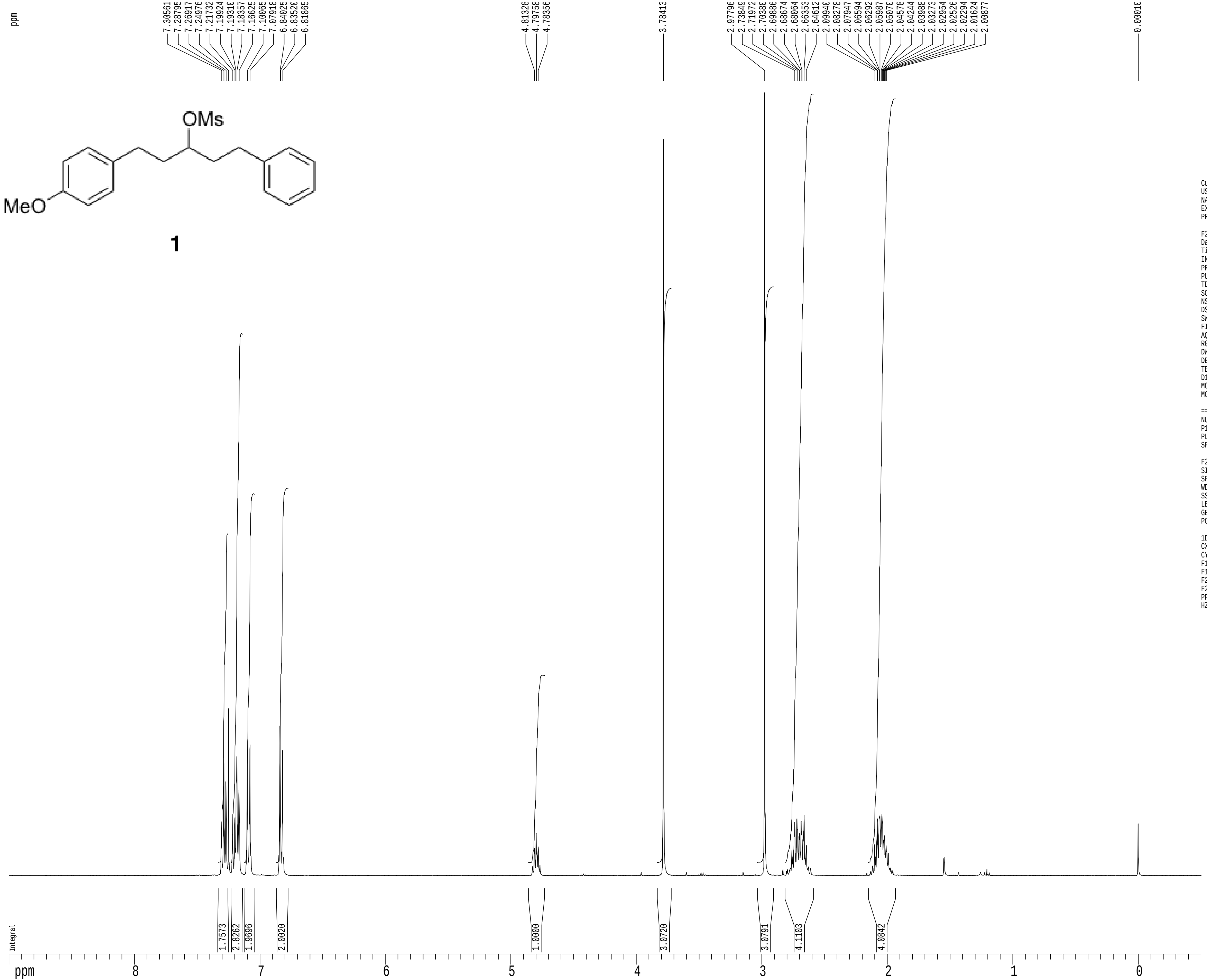

1

```
Current Data Parameters
USER          nhirbawi
NAME     NH-2-28-frac43-48-dry8-27
EXPNO                1
PROCNO               1

F2 - Acquisition Parameters
Date_          20210827
Time              16.04
INSTRUM          drx400
PROBHD   5 mm Multinucl
PULPROG            zg30
TD                65536
SOLVENT          CDCl3T
NS                    8
DS                    2
SWH            6410.256 Hz
FIDRES         0.097813 Hz
AQ            5.1118579 sec
RG                322.5
DW               78.000 usec
DE                 4.50 usec
TE                297.9 K
D1           0.10000000 sec
MCREST       0.00000000 sec
MCWRK        0.01500000 sec

======== CHANNEL f1 ========
NUC1                 1H
P1                12.00 usec
PL1               -1.10 dB
SFO1        400.1328009 MHz

F2 - Processing parameters
SI                65536
SF          400.1300256 MHz
WDW                  EM
SSB                   0
LB                 0.30 Hz
GB                    0
PC                 2.00

1D NMR plot parameters
CX                22.80 cm
CY                15.00 cm
F1P               9.000 ppm
F1              3601.17 Hz
F2P              -0.500 ppm
F2              -200.06 Hz
PPMCM           0.41667 ppm/cm
HZCM          166.72086 Hz/cm
```

Z-restored spin-echo 13C spectrum with 1H decoupling

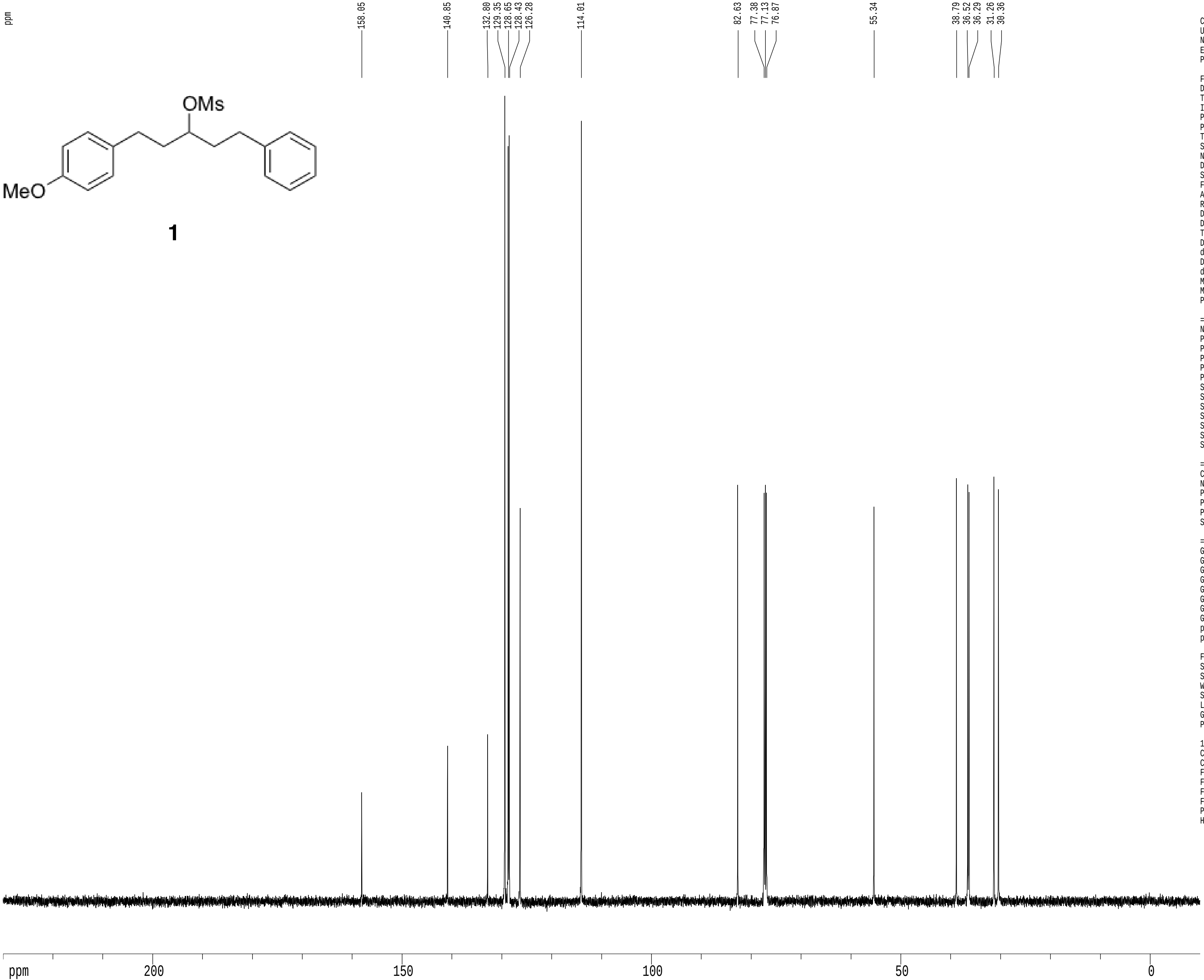


```
Current Data Parameters
USER           nhirbawi
NAME        NH-2-28-HandC
EXPNO                 2
PROCNO                1

F2 - Acquisition Parameters
Date_          20210831
Time              15.27
INSTRUM         cryo500
PROBHD   5 mm CPTCI 1H-
PULPROG  SpinEchopg30gp2.prd
TD                65536
SOLVENT           CDCl3
NS                  512
DS                   16
SWH           30303.031 Hz
FIDRES         0.462388 Hz
AQ            1.0813940 sec
RG               7298.2
DW               16.500 usec
DE                 6.00 usec
TE                288.2 K
D1           0.25000000 sec
d11          0.03000000 sec
D16          0.00020000 sec
d17          0.00019600 sec
MCREST       0.00000000 sec
MCWRK        0.01500000 sec
P2                37.70 usec

======== CHANNEL f1 ========
NUC1                13C
P1                18.85 usec
P12             2000.00 usec
P20              500.00 usec
PL0              120.00 dB
PL1               -1.00 dB
SFO1        125.7942548 MHz
SP2                1.55 dB
SP4                1.55 dB
SPNAM2      Crp60comp.4
SPNAM4   Crp60,0.5,20.1
SPOFF2             0.00 Hz
SPOFF4             0.00 Hz

======== CHANNEL f2 ========
CPDPRG2          waltz16
NUC2                  1H
PCPD2            100.00 usec
PL2                1.60 dB
PL12              22.00 dB
SFO2        500.2225011 MHz

====== GRADIENT CHANNEL =====
GPNAM1         SINE.100
GPNAM2         SINE.100
GPX1               0.00 %
GPX2               0.00 %
GPY1               0.00 %
GPY2               0.00 %
GPZ1              30.00 %
GPZ2              50.00 %
p15              500.00 usec
p16             1000.00 usec

F2 - Processing parameters
SI                65536
SF          125.7804190 MHz
WDW                  EM
SSB                   0
LB                 1.00 Hz
GB                    0
PC                 2.00

1D NMR plot parameters
CX                22.80 cm
CY                15.65 cm
F1P             230.000 ppm
F1             28929.50 Hz
F2P             -10.000 ppm
F2             -1257.80 Hz
PPMCM          10.52632 ppm/cm
HZCM         1324.00439 Hz/cm
```

1H spectrum

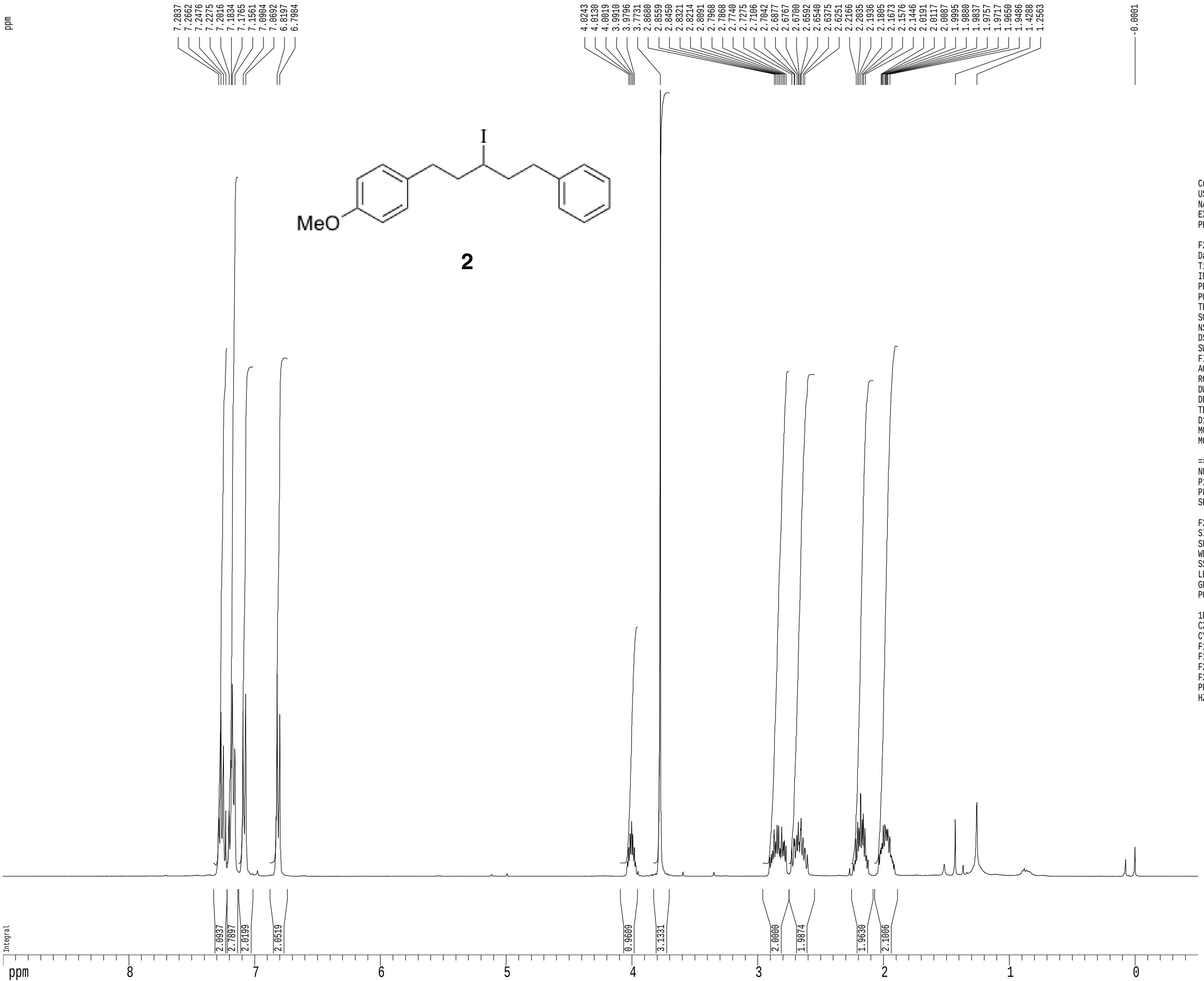


```
Current Data Parameters
USER          nhirbawi
NAME     NH-2-99-column
EXPNO                1
PROCNO               1

F2 - Acquisition Parameters
Date_          20211113
Time              15.01
INSTRUM          drx400
PROBHD   5 mm QNP H/F/P
PULPROG            zg30
TD                65536
SOLVENT          CDCl3T
NS                    8
DS                    2
SWH           6410.256 Hz
FIDRES        0.097813 Hz
AQ           5.1118579 sec
RG                101.6
DW               78.000 usec
DE                 4.50 usec
TE                298.0 K
D1           0.10000000 sec
MCREST       0.00000000 sec
MCWRK        0.01500000 sec

======== CHANNEL f1 ========
NUC1                 1H
P1                12.00 usec
PL1               -0.90 dB
SFO1        400.1328009 MHz

F2 - Processing parameters
SI                65536
SF          400.1300340 MHz
WDW                  EM
SSB                   0
LB                 0.30 Hz
GB                    0
PC                 2.00

1D NMR plot parameters
CX                22.80 cm
CY                15.00 cm
F1P               9.000 ppm
F1              3601.17 Hz
F2P              -0.500 ppm
F2              -200.06 Hz
PPMCM           0.41667 ppm/cm
HZCM          166.72086 Hz/cm
```

Z-restored spin-echo 13C spectrum with 1H decoupling

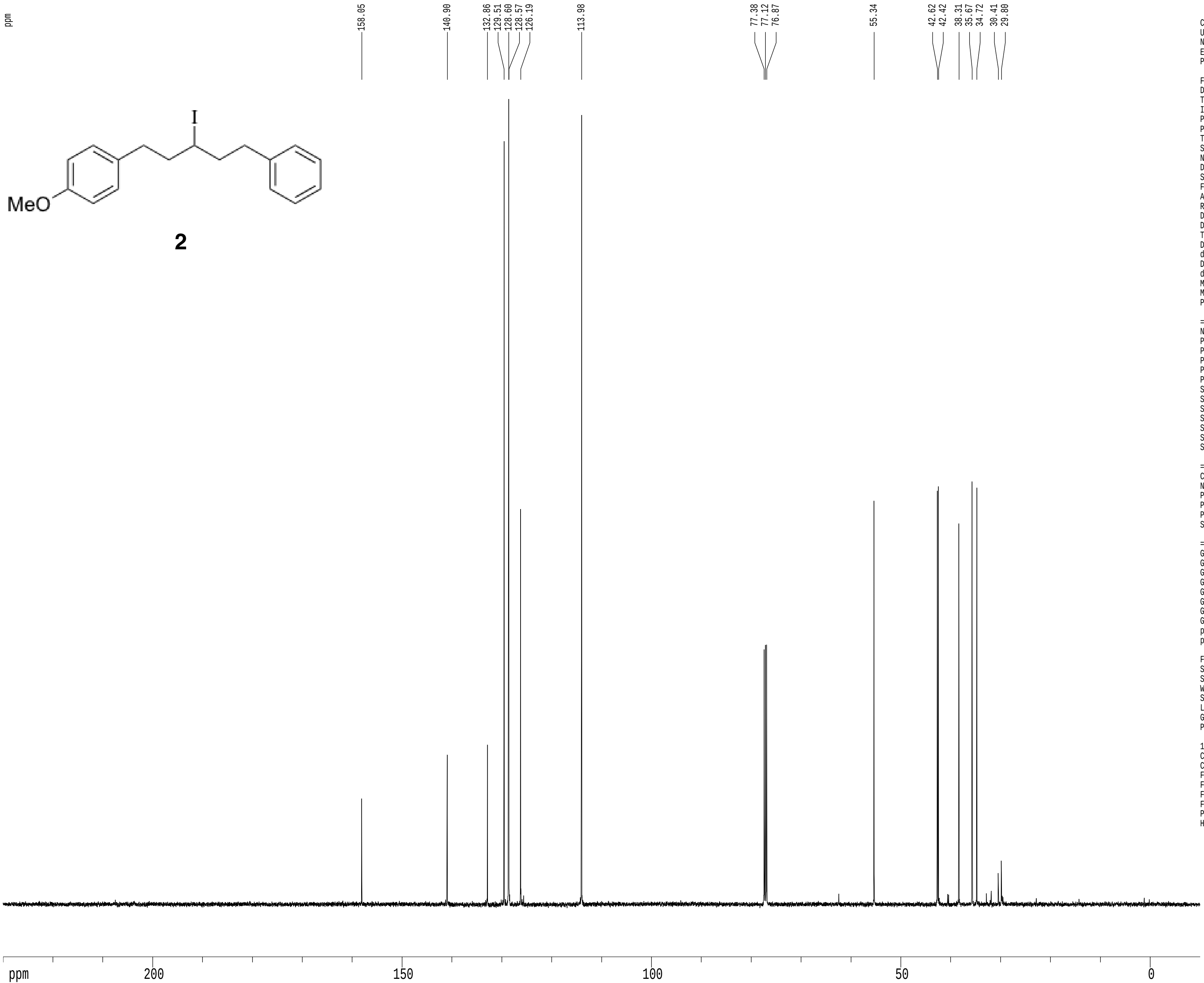


```
Current Data Parameters
USER            nhirbawi
NAME      NH-2-99-HandC
EXPNO                  2
PROCNO                 1

F2 - Acquisition Parameters
Date_           20211113
Time               15.24
INSTRUM          cryo500
PROBHD   5 mm CPTCI 1H-
PULPROG  SpinEchopg30gp2.prd
TD                 65536
SOLVENT            CDCl3
NS                   623
DS                    16
SWH            30303.031 Hz
FIDRES          0.462388 Hz
AQ             1.0813940 sec
RG                2580.3
DW                16.500 usec
DE                  6.00 usec
TE                 298.0 K
D1            0.25000000 sec
d11           0.03000000 sec
D16           0.00020000 sec
d17           0.00019600 sec
MCREST        0.00000000 sec
MCWRK         0.01500000 sec
P2                 37.70 usec

======== CHANNEL f1 ========
NUC1                 13C
P1                 18.85 usec
P12              2000.00 usec
P20               500.00 usec
PL0               120.00 dB
PL1                -1.00 dB
SFO1         125.7942548 MHz
SP2                 1.55 dB
SP4                 1.55 dB
SPNAM2      Crp60comp.4
SPNAM4   Crp60,0.5,20.1
SPOFF2              0.00 Hz
SPOFF4              0.00 Hz

======== CHANNEL f2 ========
CPDPRG2           waltz16
NUC2                   1H
PCPD2             100.00 usec
PL2                 1.60 dB
PL12               22.00 dB
SFO2         500.2225011 MHz

====== GRADIENT CHANNEL =====
GPNAM1          SINE.100
GPNAM2          SINE.100
GPX1                0.00 %
GPX2                0.00 %
GPY1                0.00 %
GPY2                0.00 %
GPZ1               30.00 %
GPZ2               50.00 %
p15               500.00 usec
p16              1000.00 usec

F2 - Processing parameters
SI                 65536
SF           125.7804190 MHz
WDW                   EM
SSB                    0
LB                  1.00 Hz
GB                     0
PC                  2.00

1D NMR plot parameters
CX                 22.80 cm
CY                 15.65 cm
F1P              230.000 ppm
F1              28929.50 Hz
F2P              -10.000 ppm
F2              -1257.80 Hz
PPMCM           10.52632 ppm/cm
HZCM          1324.00439 Hz/cm
```

1H spectrum

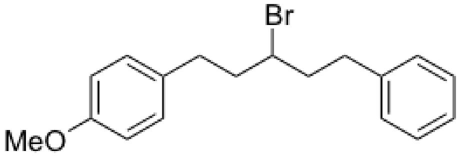

30

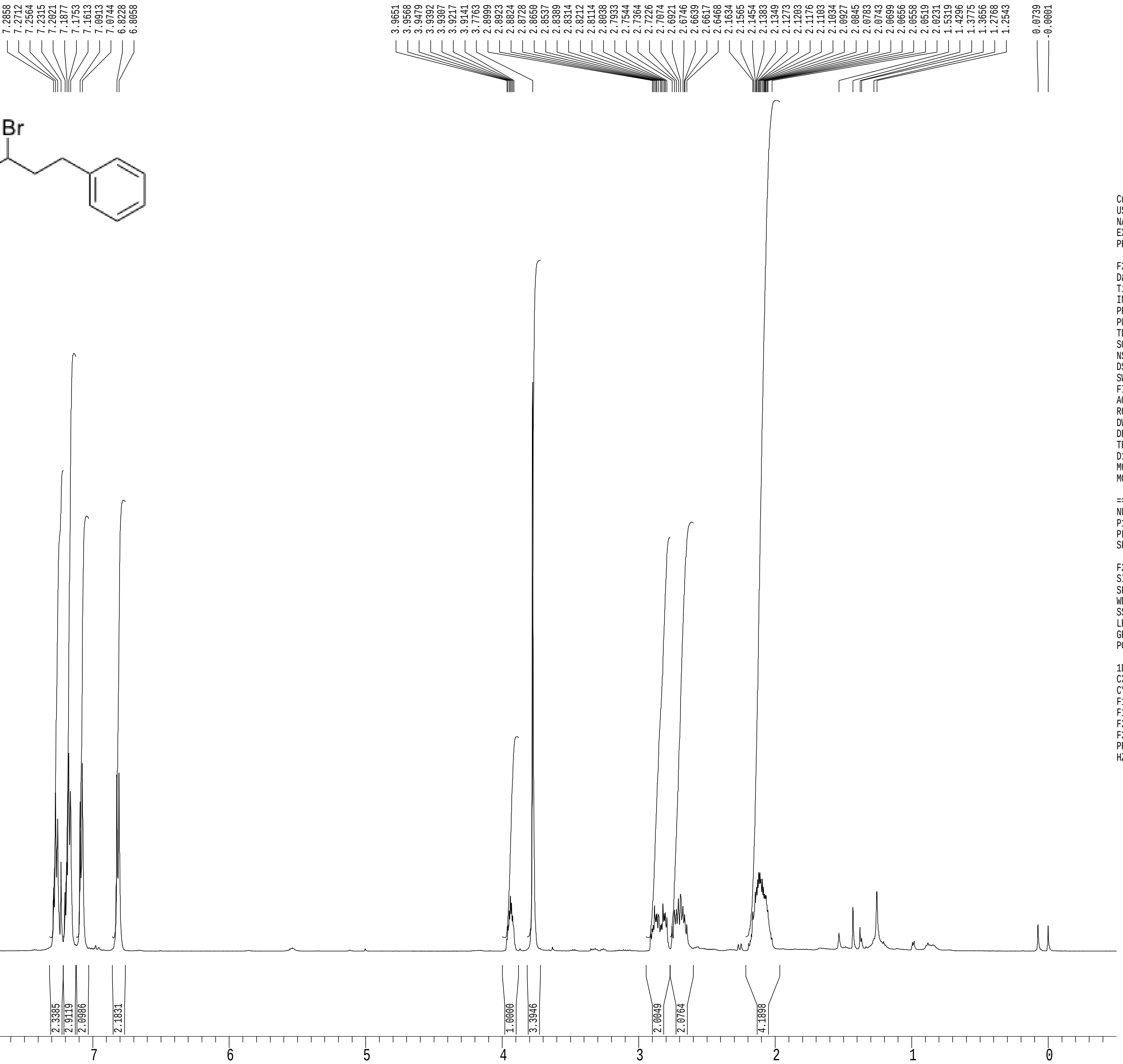

Current Data Parameters
USER nhirbawi
NAME NH-2-146-HandC
EXPNO 1
PROCNO 1

F2 - Acquisition Parameters
Date_ 20220113
Time 17.44
INSTRUM cryo500
PROBHD 5 mm CPTCI 1H-
PULPROG zg30
TD 81728
SOLVENT CDCl3T
NS 8
DS 2
SWH 8012.820 Hz
FIDRES 0.098043 Hz
AQ 5.0998774 sec
RG 8
DW 62.400 usec
DE 6.00 usec
TE 298.0 K
D1 0.10000000 sec
MCREST 0.00000000 sec
MCWRK 0.01500000 sec

======== CHANNEL f1 ========
NUC1 1H
P1 9.75 usec
PL1 1.60 dB
SFO1 500.2235015 MHz

F2 - Processing parameters
SI 65536
SF 500.2200455 MHz
WDW EM
SSB 0
LB 0.30 Hz
GB 0
PC 1.00

1D NMR plot parameters
CX 22.80 cm
CY 10.00 cm
F1P 9.000 ppm
F1 4501.98 Hz
F2P -0.500 ppm
F2 -250.11 Hz
PPMCM 0.41667 ppm/cm
HZCM 208.42502 Hz/cm

Z-restored spin-echo 13C spectrum with 1H decoupling

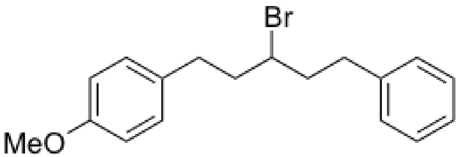

30

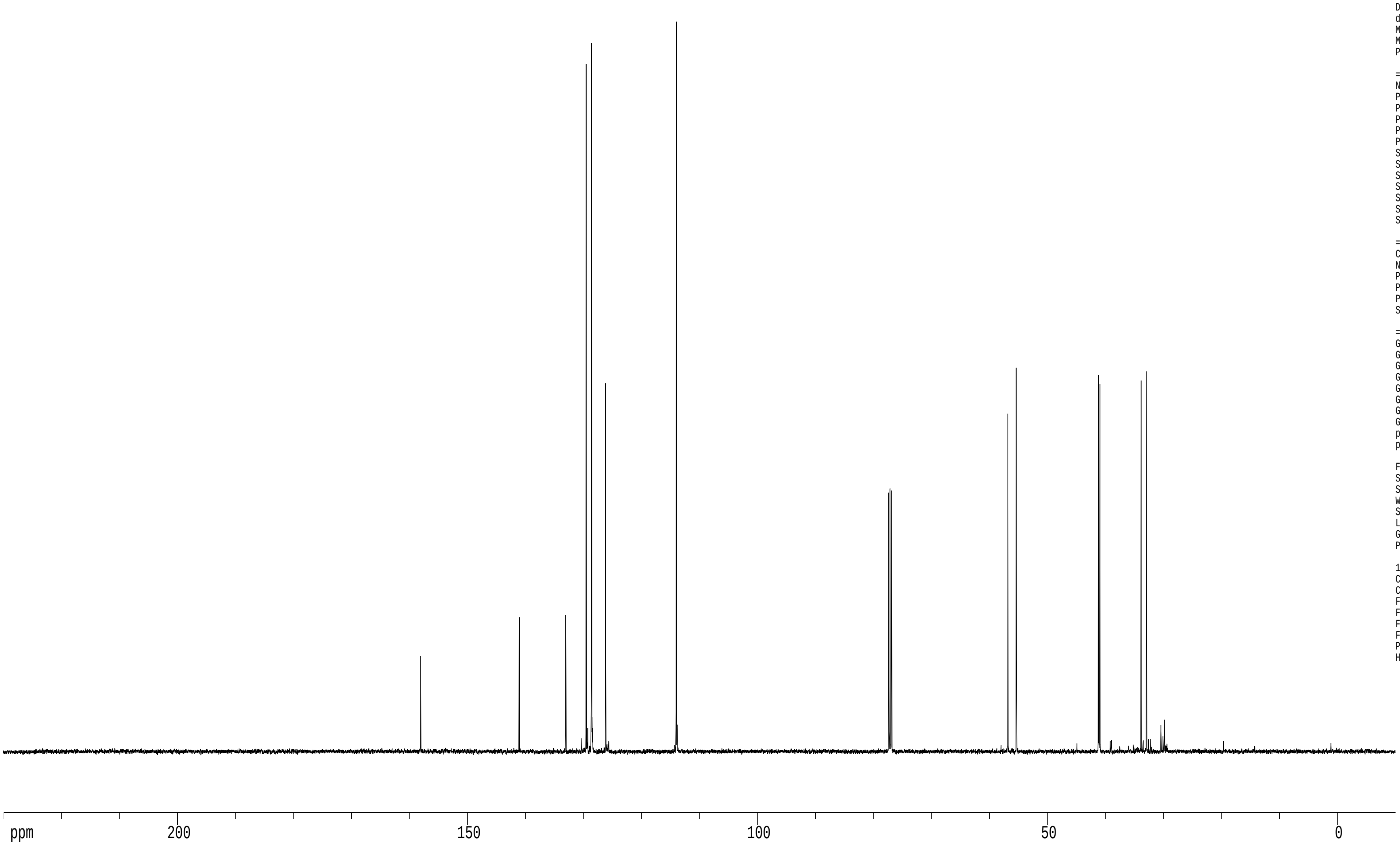

Current Data Parameters
USER nhirbawi
NAME NH-2-146-HandC
EXPNO 2
PROCNO 1

F2 - Acquisition Parameters
Date_ 20220113
Time 17.51
INSTRUM cryo500
PROBHD 5 mm CPTCI 1H-
PULPROG SpinEchopg30gp2.prd
TD 65536
SOLVENT CDCl3
NS 540
DS 16
SWH 30303.031 Hz
FIDRES 0.462388 Hz
AQ 1.0813940 sec
RG 5160.6
DW 16.500 usec
DE 6.00 usec
TE 298.0 K
D1 0.25000000 sec
d11 0.03000000 sec
D16 0.00020000 sec
d17 0.00019600 sec
MCREST 0.00000000 sec
MCWRK 0.01500000 sec
P2 37.70 usec

======== CHANNEL f1 ========
NUC1 13C
P1 18.85 usec
P12 2000.00 usec
P20 500.00 usec
PL0 120.00 dB
PL1 -1.00 dB
SFO1 125.7942548 MHz
SP2 1.55 dB
SP4 1.55 dB
SPNAM2 Crp60comp.4
SPNAM4 Crp60,0.5,20.1
SPOFF2 0.00 Hz
SPOFF4 0.00 Hz

======== CHANNEL f2 ========
CPDPRG2 waltz16
NUC2 1H
PCPD2 100.00 usec
PL2 1.60 dB
PL12 22.00 dB
SFO2 500.2225011 MHz

====== GRADIENT CHANNEL =====
GPNAM1 SINE.100
GPNAM2 SINE.100
GPX1 0.00 %
GPX2 0.00 %
GPY1 0.00 %
GPY2 0.00 %
GPZ1 30.00 %
GPZ2 50.00 %
p15 500.00 usec
p16 1000.00 usec

F2 - Processing parameters
SI 65536
SF 125.7804190 MHz
WDW EM
SSB 0
LB 1.00 Hz
GB 0
PC 2.00

1D NMR plot parameters
CX 22.80 cm
CY 12.00 cm
F1P 230.000 ppm
F1 28929.50 Hz
F2P -10.000 ppm
F2 -1257.80 Hz
PPMCM 10.52632 ppm/cm
HZCM 1324.00439 Hz/cm

1H spectrum

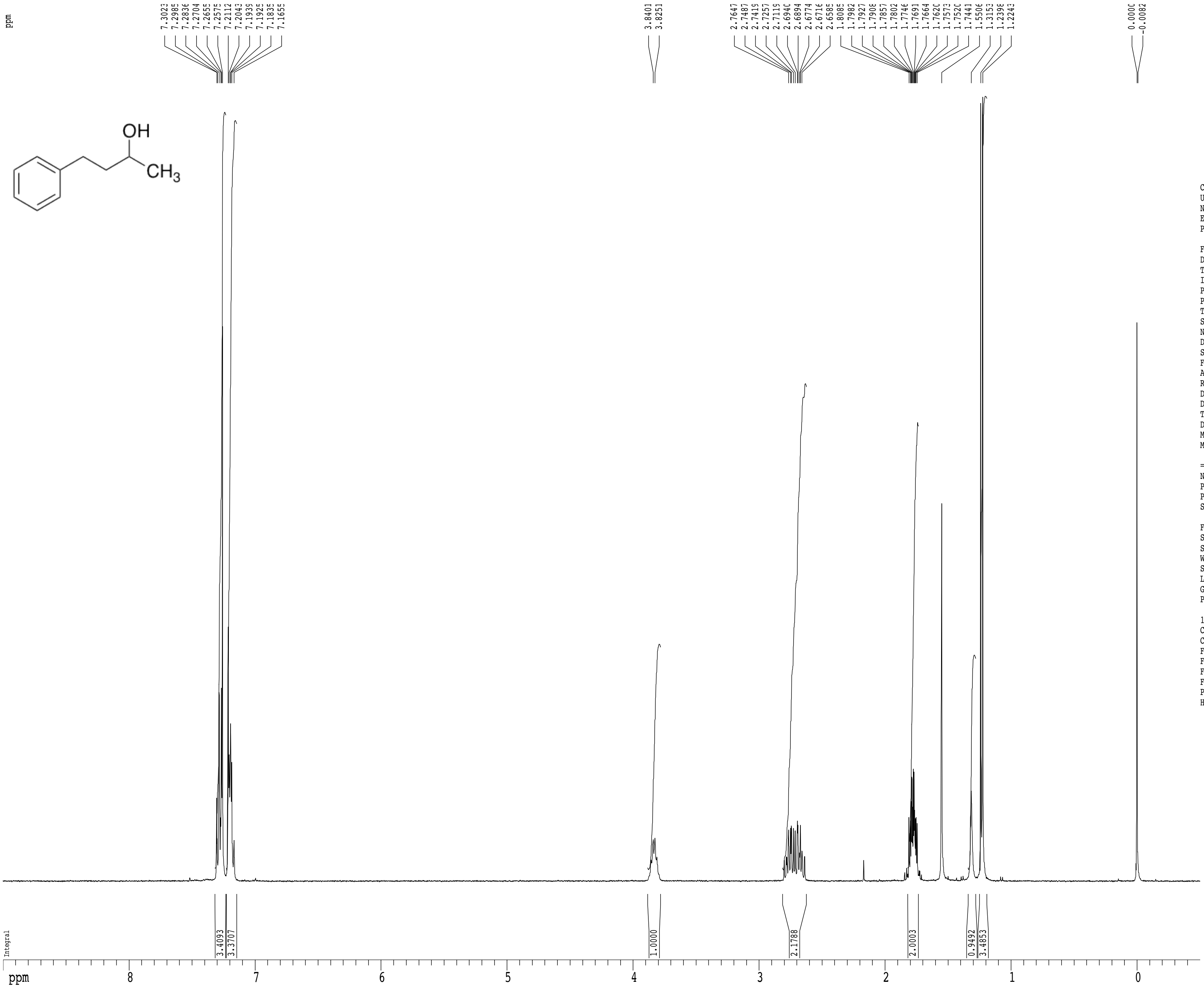


```
Current Data Parameters
USER            linpc2
NAME        pcl-2-174
EXPNO               2
PROCNO              1

F2 - Acquisition Parameters
Date_        20210915
Time             8.40
INSTRUM        drx400
PROBHD   5 mm Multinucl
PULPROG          zg30
TD              65536
SOLVENT         CDCl3
NS                  8
DS                  2
SWH          6410.256 Hz
FIDRES       0.097813 Hz
AQ          5.1118579 sec
RG               1024
DW             78.000 usec
DE               4.50 usec
TE              298.0 K
D1         0.10000000 sec
MCREST     0.00000000 sec
MCWRK      0.01500000 sec

======== CHANNEL f1 ========
NUC1               1H
P1              12.00 usec
PL1             -1.10 dB
SFO1      400.1328009 MHz

F2 - Processing parameters
SI              65536
SF        400.1300226 MHz
WDW                EM
SSB                 0
LB               0.30 Hz
GB                  0
PC               2.00

1D NMR plot parameters
CX              22.80 cm
CY              15.00 cm
F1P             9.000 ppm
F1            3601.17 Hz
F2P            -0.500 ppm
F2            -200.06 Hz
PPMCM         0.41667 ppm/cm
HZCM        166.72086 Hz/cm
```

1H spectrum

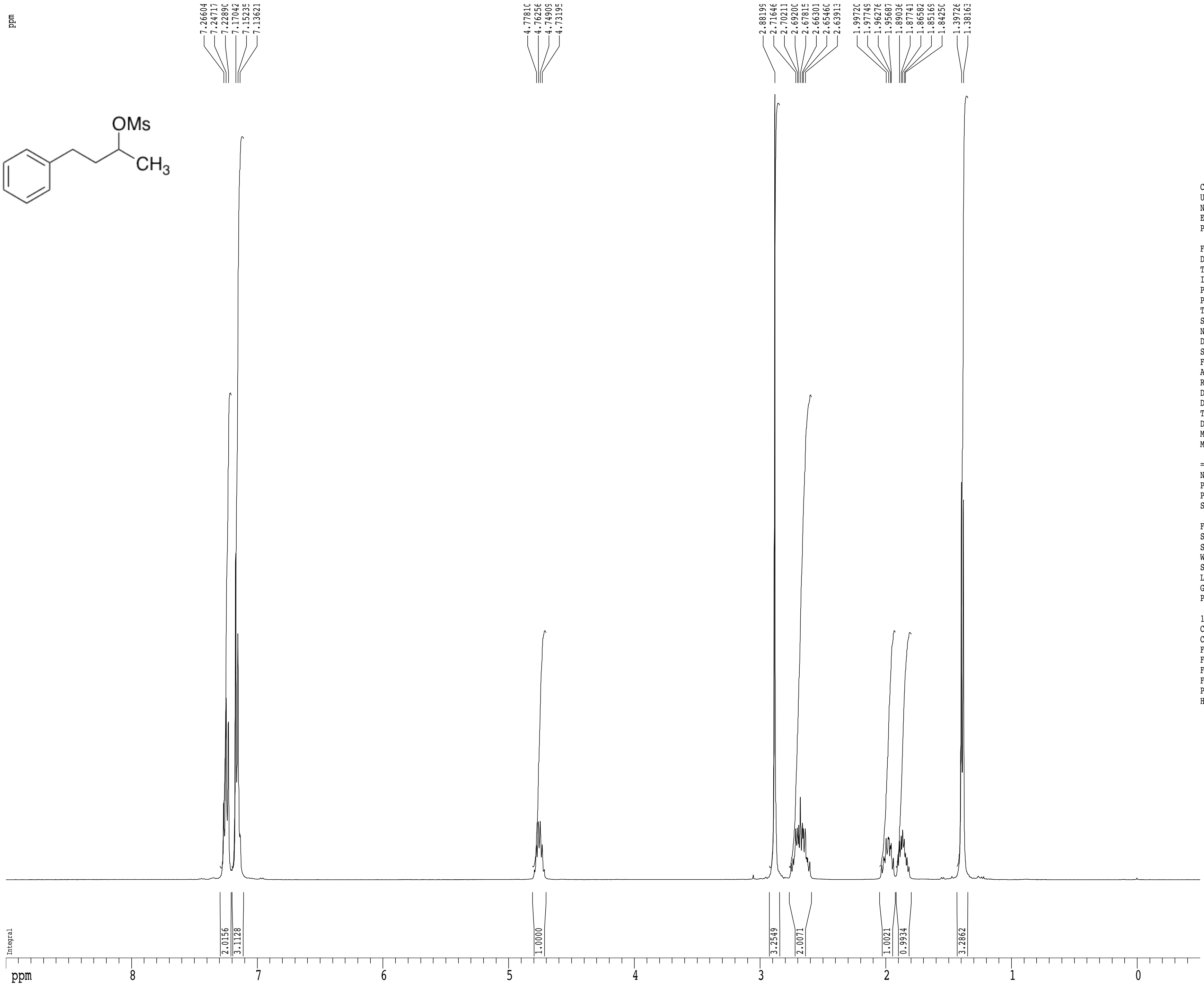

Current Data Parameters
USER linpc2
NAME pcl-2-177
EXPNO 1
PROCNO 1

F2 - Acquisition Parameters
Date_ 20210915
Time 8.33
INSTRUM drx400
PROBHD 5 mm Multinucl
PULPROG zg30
TD 65536
SOLVENT CDCl3
NS 8
DS 2
SWH 6410.256 Hz
FIDRES 0.097813 Hz
AQ 5.1118579 sec
RG 20.2
DW 78.000 usec
DE 4.50 usec
TE 298.0 K
D1 0.10000000 sec
MCREST 0.00000000 sec
MCWRK 0.01500000 sec

======== CHANNEL f1 ========
NUC1 1H
P1 12.00 usec
PL1 -1.10 dB
SFO1 400.1328009 MHz

F2 - Processing parameters
SI 65536
SF 400.1300429 MHz
WDW EM
SSB 0
LB 0.30 Hz
GB 0
PC 2.00

1D NMR plot parameters
CX 22.80 cm
CY 15.00 cm
F1P 9.000 ppm
F1 3601.17 Hz
F2P -0.500 ppm
F2 -200.06 Hz
PPMCM 0.41667 ppm/cm
HZCM 166.72086 Hz/cm

13C spectrum with 1H decoupling

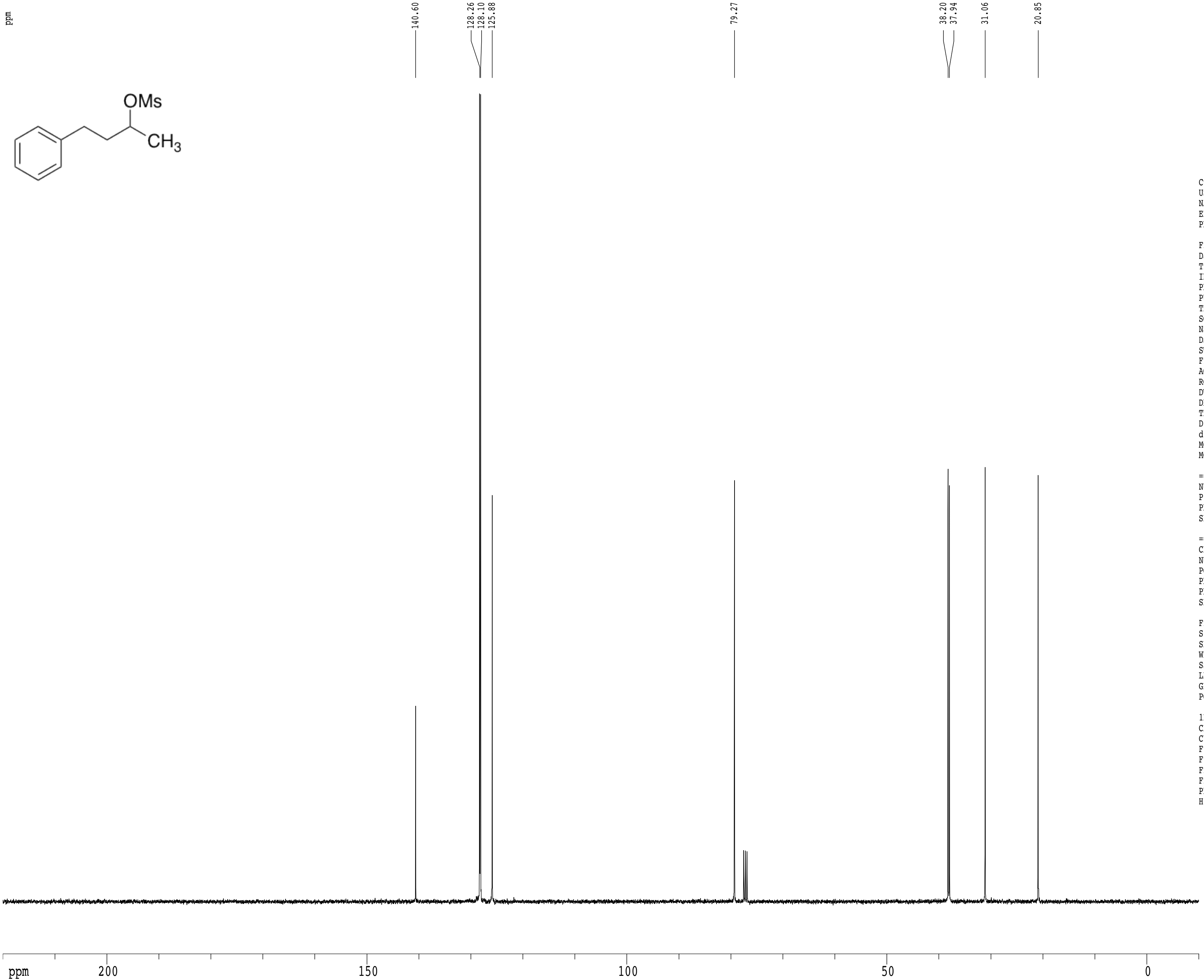

Current Data Parameters
USER linpc2
NAME pcl-2-177
EXPNO 2
PROCNO 1

F2 - Acquisition Parameters
Date_ 20210915
Time 8.35
INSTRUM drx400
PROBHD 5 mm Multinucl
PULPROG zgdc30
TD 65536
SOLVENT CDCl3
NS 88
DS 4
SWH 24154.590 Hz
FIDRES 0.368570 Hz
AQ 1.3566452 sec
RG 14596.5
DW 20.700 usec
DE 20.39 usec
TE 298.0 K
D1 0.10000000 sec
d11 0.03000000 sec
MCREST 0.00000000 sec
MCWRK 0.01500000 sec

======== CHANNEL f1 ========
NUC1 13C
P1 9.00 usec
PL1 -2.50 dB
SFO1 100.6237964 MHz

======== CHANNEL f2 ========
CPDPRG2 waltz16
NUC2 1H
PCPD2 80.00 usec
PL2 -1.10 dB
PL12 15.60 dB
SFO2 400.1328009 MHz

F2 - Processing parameters
SI 65536
SF 100.6128070 MHz
WDW EM
SSB 0
LB 1.00 Hz
GB 0
PC 1.00

1D NMR plot parameters
CX 22.80 cm
CY 15.50 cm
F1P 220.000 ppm
F1 22134.82 Hz
F2P -10.000 ppm
F2 -1006.13 Hz
PPMCM 10.08772 ppm/cm
HZCM 1014.95380 Hz/cm

1H spectrum

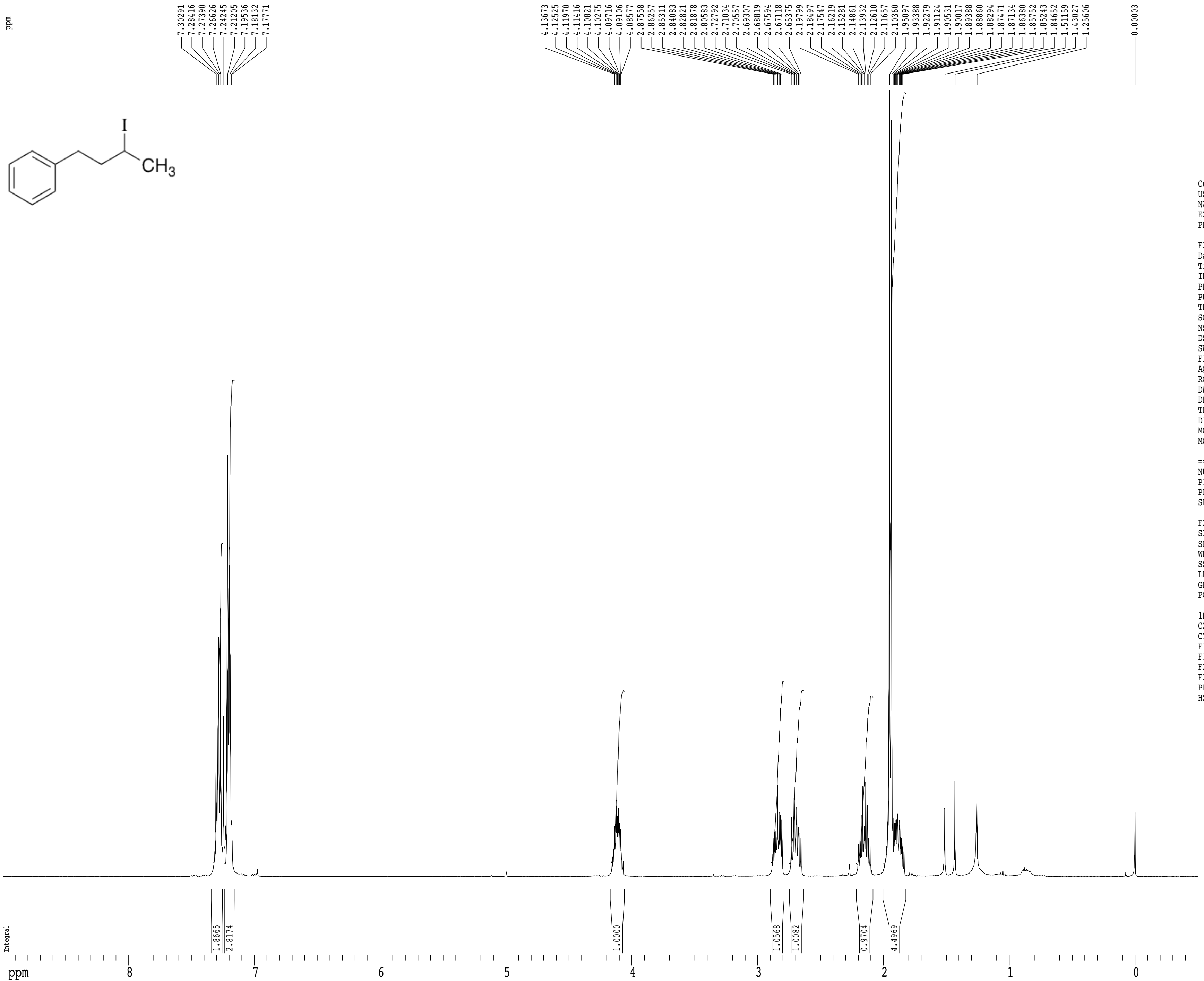

Current Data Parameters
USER linpc2
NAME pcl-2-228
EXPNO 2
PROCNO 1

F2 - Acquisition Parameters
Date_ 20211108
Time 14.28
INSTRUM drx400
PROBHD 5 mm QNP H/F/P
PULPROG zg30
TD 65536
SOLVENT CDCl3
NS 8
DS 2
SWH 6410.256 Hz
FIDRES 0.097813 Hz
AQ 5.1118579 sec
RG 161.3
DW 78.000 usec
DE 4.50 usec
TE 298.0 K
D1 0.10000000 sec
MCREST 0.00000000 sec
MCWRK 0.01500000 sec

======== CHANNEL f1 ========
NUC1 1H
P1 12.00 usec
PL1 -0.90 dB
SFO1 400.1328009 MHz

F2 - Processing parameters
SI 65536
SF 400.1300281 MHz
WDW EM
SSB 0
LB 0.30 Hz
GB 0
PC 2.00

1D NMR plot parameters
CX 22.80 cm
CY 15.00 cm
F1P 9.000 ppm
F1 3601.17 Hz
F2P -0.500 ppm
F2 -200.06 Hz
PPMCM 0.41667 ppm/cm
HZCM 166.72086 Hz/cm

1H spectrum

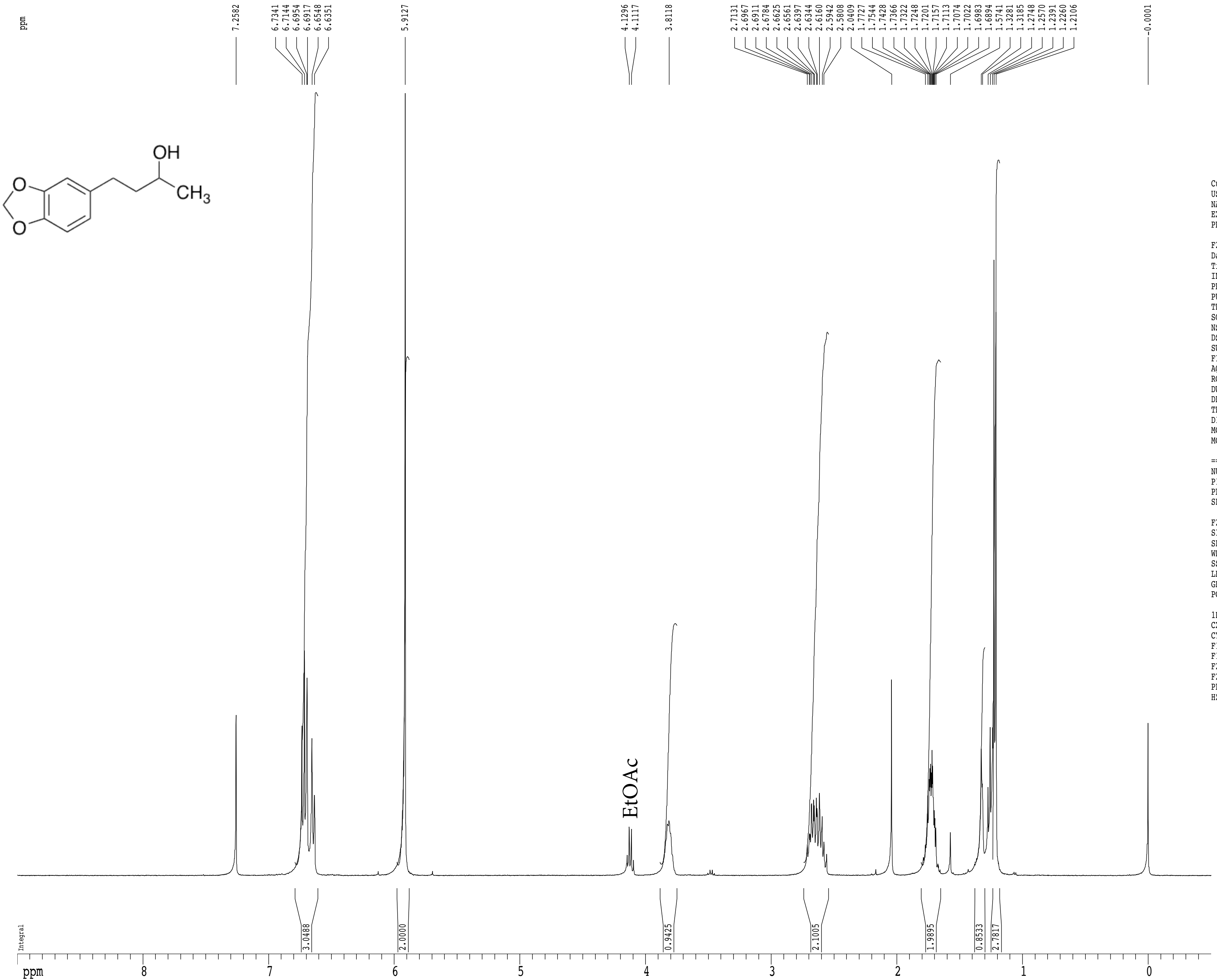

Current Data Parameters
USER linpc2
NAME pcl-2-262
EXPNO 1
PROCNO 1

F2 - Acquisition Parameters
Date_ 20211210
Time 15.22
INSTRUM drx400
PROBHD 5 mm QNP H/F/P
PULPROG zg30
TD 65536
SOLVENT CDCl3
NS 8
DS 2
SWH 6410.256 Hz
FIDRES 0.097813 Hz
AQ 5.1118579 sec
RG 287.4
DW 78.000 usec
DE 4.50 usec
TE 298.0 K
D1 0.10000000 sec
MCREST 0.00000000 sec
MCWRK 0.01500000 sec

======== CHANNEL f1 ========
NUC1 1H
P1 12.00 usec
PL1 -0.90 dB
SFO1 400.1328009 MHz

F2 - Processing parameters
SI 65536
SF 400.1300215 MHz
WDW EM
SSB 0
LB 0.30 Hz
GB 0
PC 2.00

1D NMR plot parameters
CX 22.80 cm
CY 15.00 cm
F1P 9.000 ppm
F1 3601.17 Hz
F2P -0.500 ppm
F2 -200.06 Hz
PPMCM 0.41667 ppm/cm
HZCM 166.72086 Hz/cm

1H spectrum

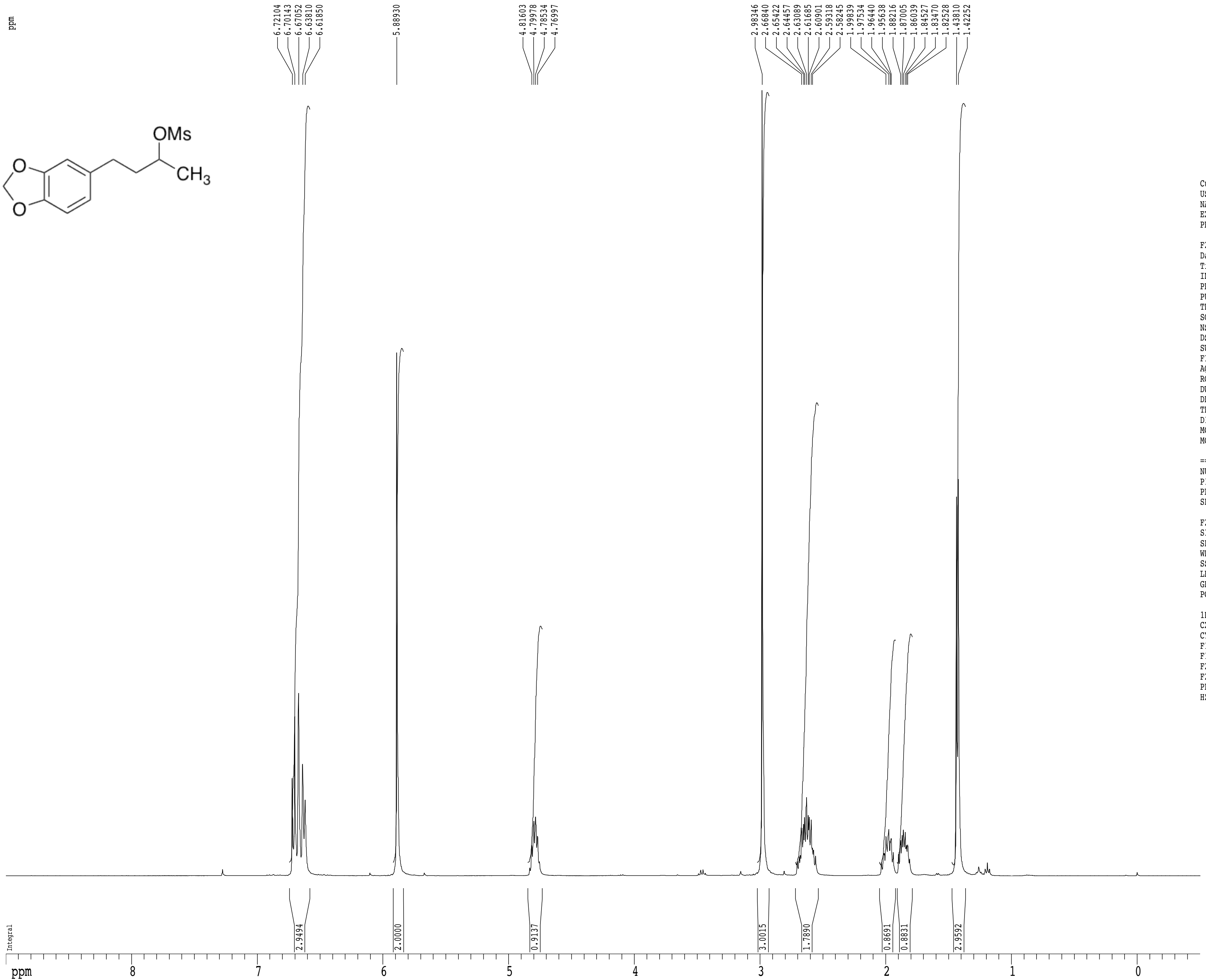

Current Data Parameters
USER linpc2
NAME pcl-2-264
EXPNO 2
PROCNO 1

F2 - Acquisition Parameters
Date_ 20211213
Time 15.46
INSTRUM drx400
PROBHD 5 mm QNP H/F/P
PULPROG zg30
TD 65536
SOLVENT CDCl3
NS 8
DS 2
SWH 6410.256 Hz
FIDRES 0.097813 Hz
AQ 5.1118579 sec
RG 32
DW 78.000 usec
DE 4.50 usec
TE 298.0 K
D1 0.10000000 sec
MCREST 0.00000000 sec
MCWRK 0.01500000 sec

======== CHANNEL f1 ========
NUC1 1H
P1 12.00 usec
PL1 -0.90 dB
SFO1 400.1328009 MHz

F2 - Processing parameters
SI 65536
SF 400.1300144 MHz
WDW EM
SSB 0
LB 0.30 Hz
GB 0
PC 2.00

1D NMR plot parameters
CX 22.80 cm
CY 15.00 cm
F1P 9.000 ppm
F1 3601.17 Hz
F2P -0.500 ppm
F2 -200.06 Hz
PPMCM 0.41667 ppm/cm
HZCM 166.72084 Hz/cm

13C spectrum with 1H decoupling

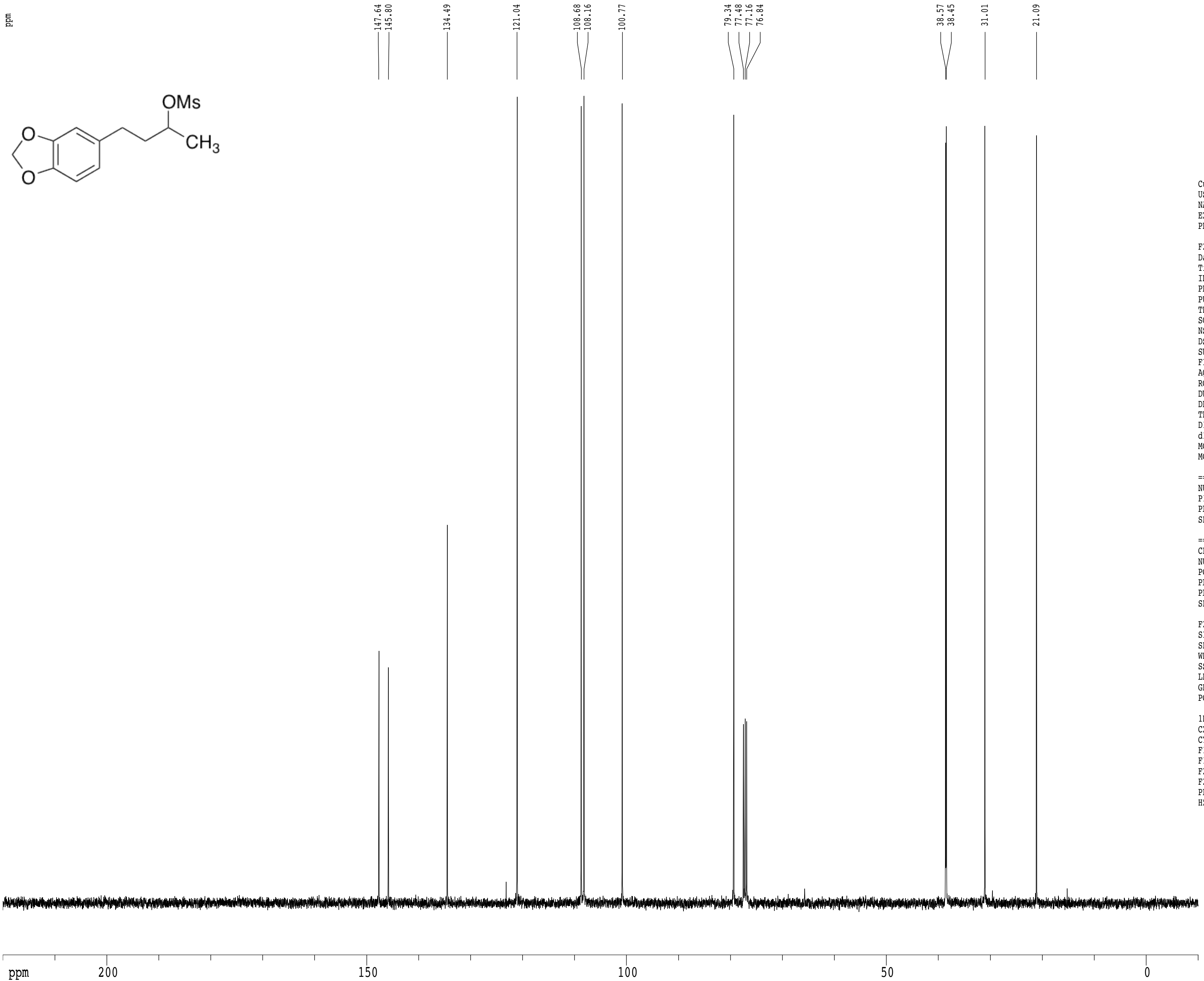


```
Current Data Parameters
USER              linpc2
NAME          pcl-2-264
EXPNO                  3
PROCNO                 1

F2 - Acquisition Parameters
Date_           20211213
Time               15.50
INSTRUM           drx400
PROBHD   5 mm QNP H/F/P
PULPROG           zgdc30
TD                 65536
SOLVENT            CDCl3
NS                   128
DS                     4
SWH            24154.590 Hz
FIDRES          0.368570 Hz
AQ             1.3566452 sec
RG                9195.2
DW                20.700 usec
DE                 20.39 usec
TE                 298.0 K
D1            0.10000000 sec
d11           0.03000000 sec
MCREST        0.00000000 sec
MCWRK         0.01500000 sec

======== CHANNEL f1 ========
NUC1                 13C
P1                  7.90 usec
PL1                -3.00 dB
SFO1         100.6237964 MHz

======== CHANNEL f2 ========
CPDPRG2           waltz16
NUC2                   1H
PCPD2               90.00 usec
PL2                 -0.90 dB
PL12                17.00 dB
SFO2          400.1328009 MHz

F2 - Processing parameters
SI                  65536
SF            100.6127801 MHz
WDW                    EM
SSB                     0
LB                   1.00 Hz
GB                      0
PC                   1.00

1D NMR plot parameters
CX                  22.80 cm
CY                  15.50 cm
F1P               220.000 ppm
F1               22134.81 Hz
F2P               -10.000 ppm
F2               -1006.13 Hz
PPMCM            10.08772 ppm/cm
HZCM           1014.95349 Hz/cm
```

1H spectrum

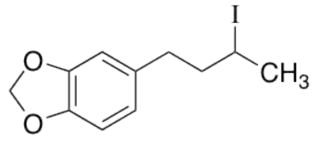

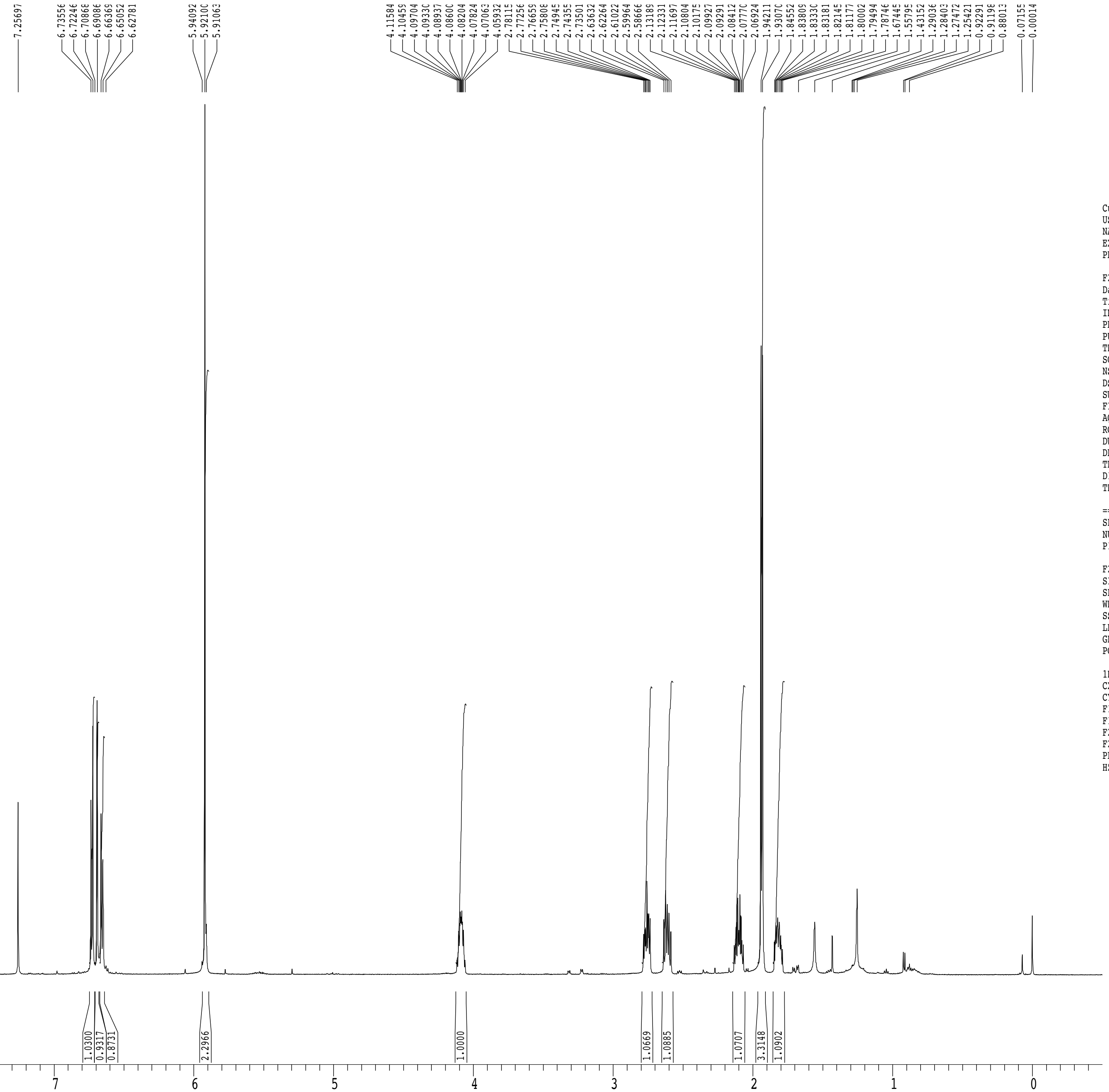

Current Data Parameters
USER linpc2
NAME pcl-2-285
EXPNO 3
PROCNO 1

F2 - Acquisition Parameters
Date_ 20220107
Time 8.16
INSTRUM av600
PROBHD 5 mm CPBBO BB-
PULPROG zg30
TD 98074
SOLVENT CDCl3T
NS 8
DS 2
SWH 9615.385 Hz
FIDRES 0.098042 Hz
AQ 5.0998979 sec
RG 10
DW 52.000 usec
DE 14.23 usec
TE 298.0 K
D1 0.10000000 sec
TD0 1

======== CHANNEL f1 ========
SFO1 600.1342009 MHz
NUC1 1H
P1 9.50 usec

F2 - Processing parameters
SI 65536
SF 600.1300373 MHz
WDW no
SSB 0
LB 0.00 Hz
GB 0
PC 1.00

1D NMR plot parameters
CX 22.80 cm
CY 15.00 cm
F1P 9.000 ppm
F1 5401.17 Hz
F2P -0.500 ppm
F2 -300.06 Hz
PPMCM 0.41667 ppm/cm
HZCM 250.05420 Hz/cm

1H spectrum

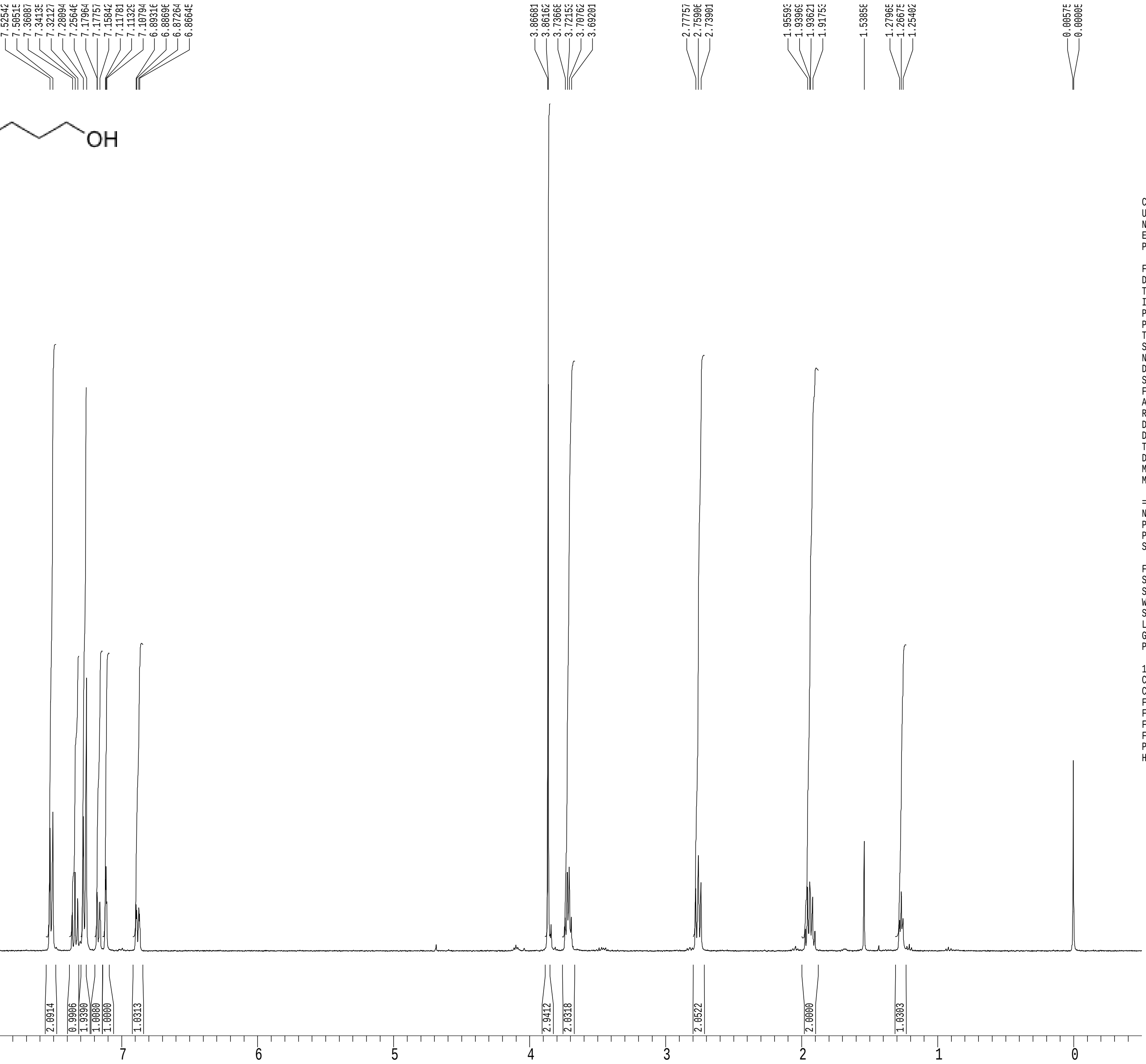

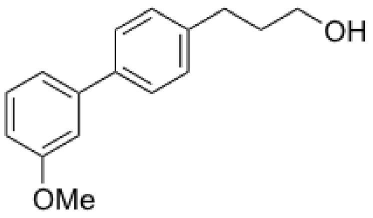

46

```
Current Data Parameters
USER          nhirbawi
NAME     NH-2-48-column
EXPNO                2
PROCNO               1

F2 - Acquisition Parameters
Date_         20210913
Time             12.17
INSTRUM         drx400
PROBHD   5 mm Multinucl
PULPROG           zg30
TD               65536
SOLVENT         CDCl3T
NS                   8
DS                   2
SWH           6410.256 Hz
FIDRES        0.097813 Hz
AQ           5.1118579 sec
RG              1149.4
DW              78.000 usec
DE                4.50 usec
TE               298.1 K
D1          0.10000000 sec
MCREST      0.00000000 sec
MCWRK       0.01500000 sec

======== CHANNEL f1 ========
NUC1                1H
P1               12.00 usec
PL1              -1.10 dB
SFO1       400.1328009 MHz

F2 - Processing parameters
SI               65536
SF         400.1300226 MHz
WDW                 EM
SSB                  0
LB                0.30 Hz
GB                   0
PC                2.00

1D NMR plot parameters
CX               22.80 cm
CY               10.00 cm
F1P              9.000 ppm
F1             3601.17 Hz
F2P             -0.500 ppm
F2             -200.06 Hz
PPMCM          0.41667 ppm/cm
HZCM         166.72086 Hz/cm
```

Z-restored spin-echo 13C spectrum with 1H decoupling

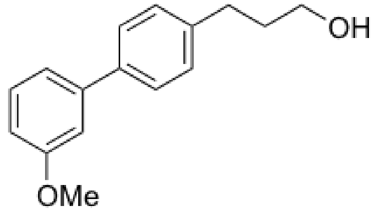

46

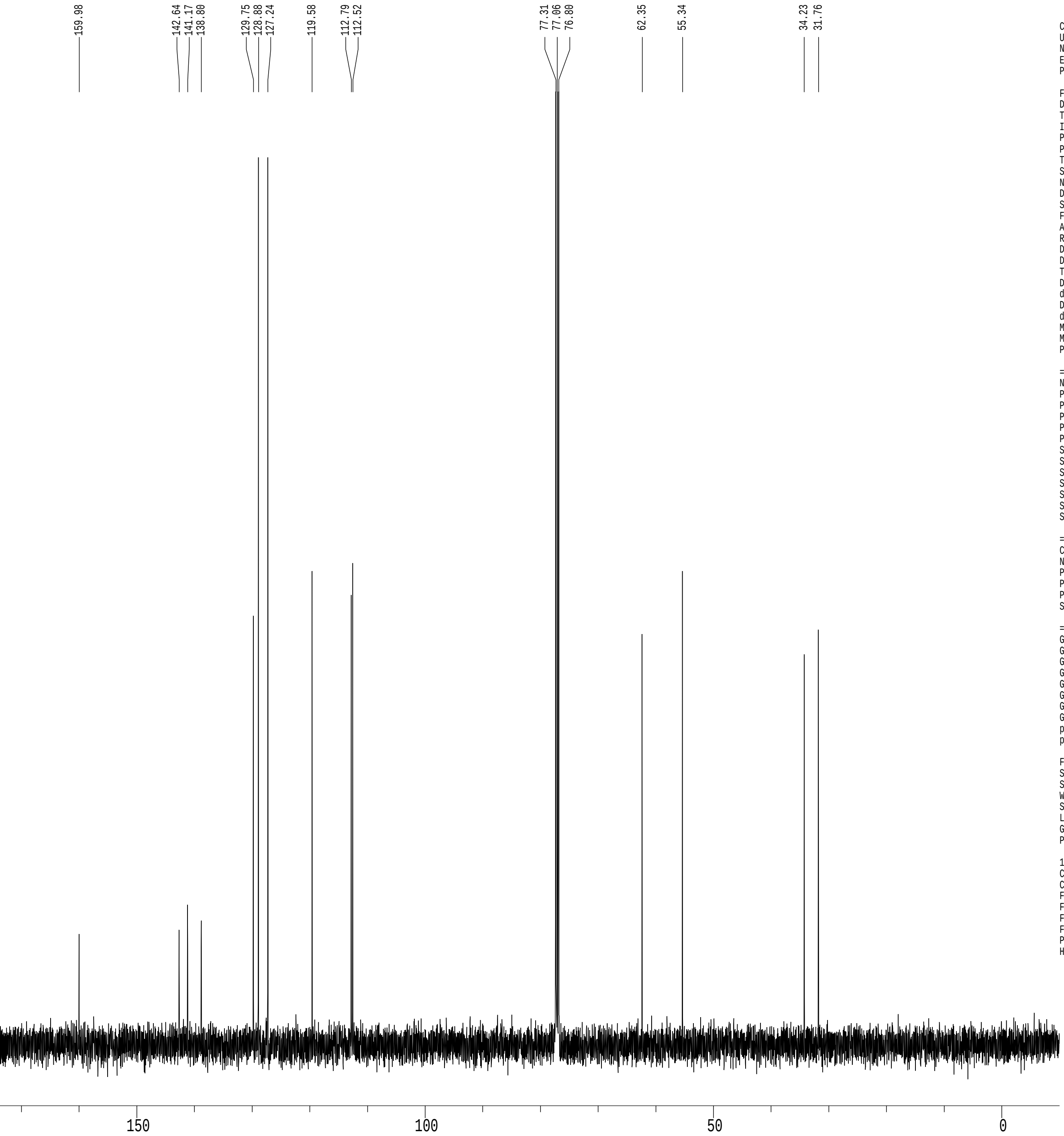


```
Current Data Parameters
USER          nhirbawi
NAME     NH-2-48-carbon
EXPNO                1
PROCNO               1

F2 - Acquisition Parameters
Date_         20210917
Time             16.53
INSTRUM        cryo500
PROBHD   5 mm CPTCI 1H-
PULPROG  SpinEchopg30gp2.prd
TD               65536
SOLVENT          CDCl3T
NS                 512
DS                  16
SWH          30303.031 Hz
FIDRES        0.462388 Hz
AQ           1.0813940 sec
RG                8192
DW              16.500 usec
DE                6.00 usec
TE               298.0 K
D1          0.25000000 sec
d11         0.03000000 sec
D16         0.00020000 sec
d17         0.00019600 sec
MCREST      0.00000000 sec
MCWRK       0.01500000 sec
P2               37.70 usec

======== CHANNEL f1 ========
NUC1               13C
P1               18.85 usec
P12            2000.00 usec
P20             500.00 usec
PL0             120.00 dB
PL1              -1.00 dB
SFO1       125.7942548 MHz
SP2               1.55 dB
SP4               1.55 dB
SPNAM2     Crp60comp.4
SPNAM4   Crp60,0.5,20.1
SPOFF2            0.00 Hz
SPOFF4            0.00 Hz

======== CHANNEL f2 ========
CPDPRG2         waltz16
NUC2                1H
PCPD2           100.00 usec
PL2               1.60 dB
PL12             22.00 dB
SFO2       500.2225011 MHz

====== GRADIENT CHANNEL =====
GPNAM1        SINE.100
GPNAM2        SINE.100
GPX1              0.00 %
GPX2              0.00 %
GPY1              0.00 %
GPY2              0.00 %
GPZ1             30.00 %
GPZ2             50.00 %
p15             500.00 usec
p16            1000.00 usec

F2 - Processing parameters
SI               65536
SF         125.7804190 MHz
WDW                 EM
SSB                  0
LB                1.00 Hz
GB                   0
PC                2.00

1D NMR plot parameters
CX               22.80 cm
CY               30.00 cm
F1P            230.000 ppm
F1            28929.50 Hz
F2P            -10.000 ppm
F2            -1257.80 Hz
PPMCM         10.52632 ppm/cm
HZCM        1324.00439 Hz/cm
```

1H spectrum

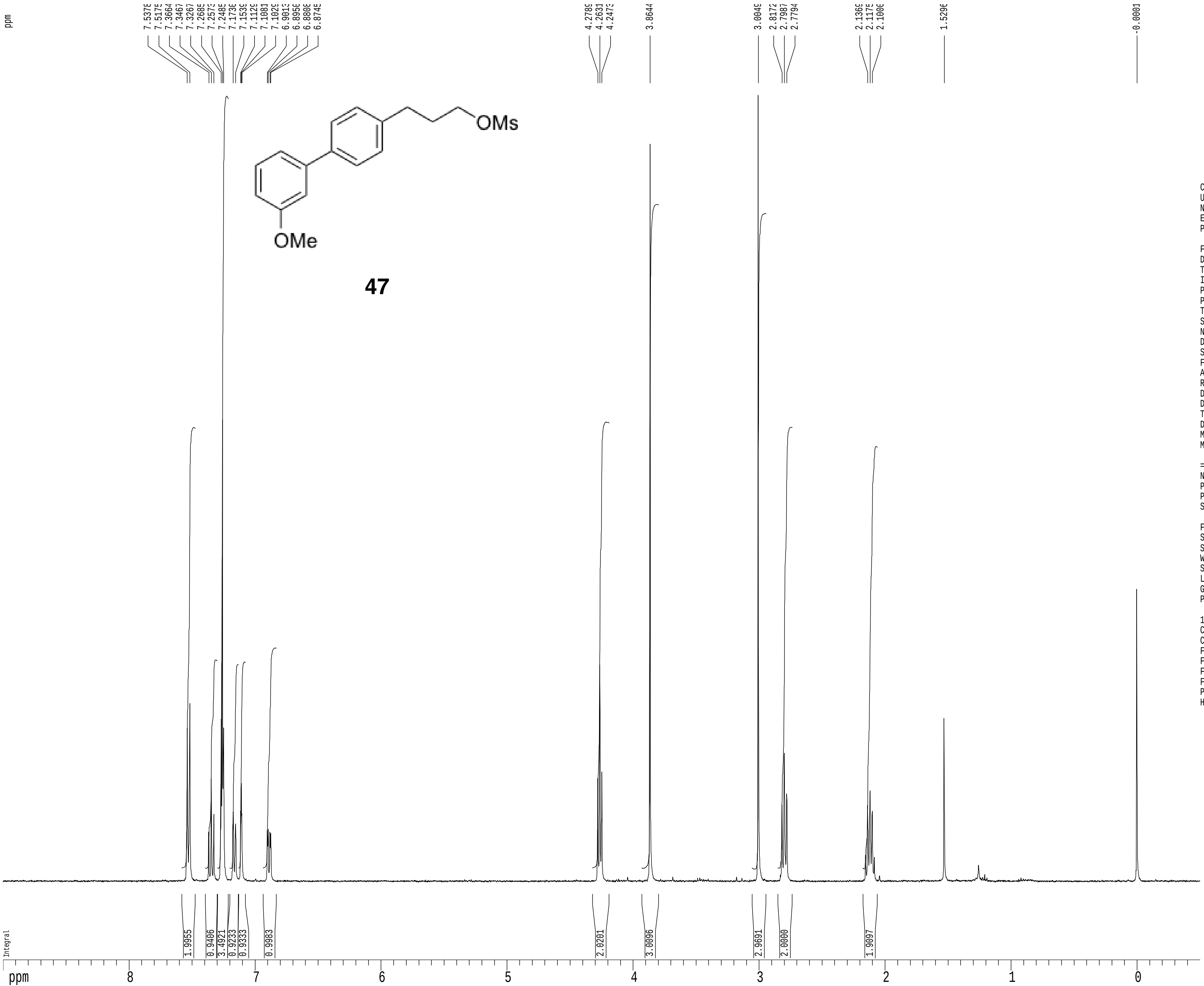


```
Current Data Parameters
USER          nhirbawi
NAME     NH-2-55-column
EXPNO                1
PROCNO               1

F2 - Acquisition Parameters
Date_         20210916
Time             11.38
INSTRUM         drx400
PROBHD   5 mm Multinucl
PULPROG           zg30
TD               65536
SOLVENT          CDCl3T
NS                   8
DS                   2
SWH           6410.256 Hz
FIDRES        0.097813 Hz
AQ           5.1118579 sec
RG                1024
DW              78.000 usec
DE                4.50 usec
TE               298.1 K
D1          0.10000000 sec
MCREST      0.00000000 sec
MCWRK       0.01500000 sec

======== CHANNEL f1 ========
NUC1                1H
P1               12.00 usec
PL1              -1.10 dB
SFO1       400.1328009 MHz

F2 - Processing parameters
SI               65536
SF         400.1300225 MHz
WDW                 EM
SSB                  0
LB                0.30 Hz
GB                   0
PC                2.00

1D NMR plot parameters
CX               22.80 cm
CY               15.00 cm
F1P              9.000 ppm
F1             3601.17 Hz
F2P             -0.500 ppm
F2             -200.06 Hz
PPMCM          0.41667 ppm/cm
HZCM         166.72086 Hz/cm
```

Z-restored spin-echo 13C spectrum with 1H decoupling

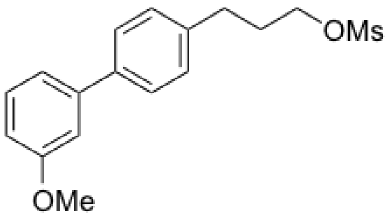

47

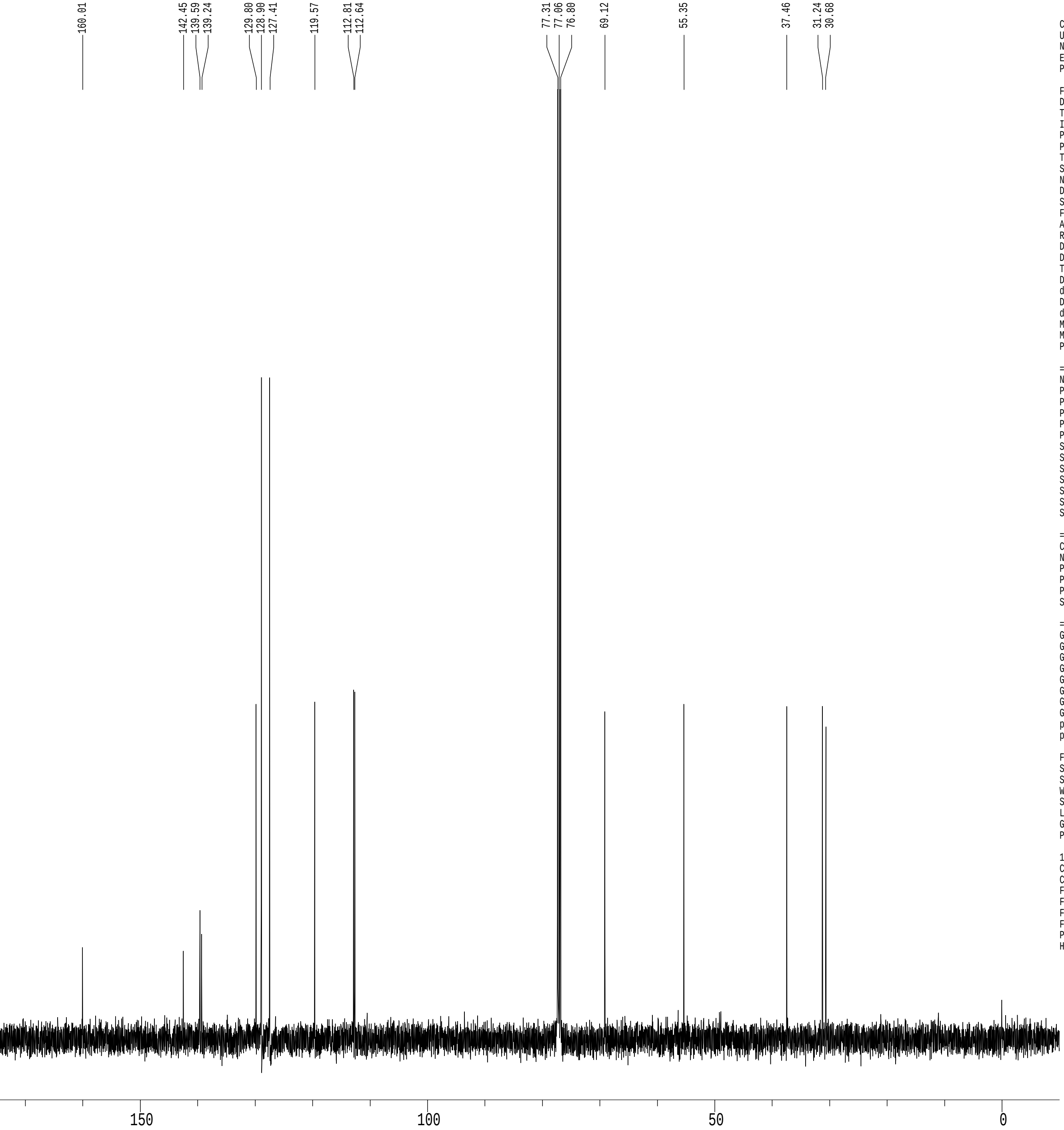

Current Data Parameters
USER nhirbawi
NAME NH-2-55-carbon
EXPNO 1
PROCNO 1

F2 - Acquisition Parameters
Date_ 20210917
Time 17.08
INSTRUM cryo500
PROBHD 5 mm CPTCI 1H-
PULPROG SpinEchopg30gp2.prd
TD 65536
SOLVENT CDCl3T
NS 512
DS 16
SWH 30303.031 Hz
FIDRES 0.462388 Hz
AQ 1.0813940 sec
RG 3251
DW 16.500 usec
DE 6.00 usec
TE 298.0 K
D1 0.25000000 sec
d11 0.03000000 sec
D16 0.00020000 sec
d17 0.00019600 sec
MCREST 0.00000000 sec
MCWRK 0.01500000 sec
P2 37.70 usec

======== CHANNEL f1 ========
NUC1 13C
P1 18.85 usec
P12 2000.00 usec
P20 500.00 usec
PL0 120.00 dB
PL1 -1.00 dB
SFO1 125.7942548 MHz
SP2 1.55 dB
SP4 1.55 dB
SPNAM2 Crp60comp.4
SPNAM4 Crp60,0.5,20.1
SPOFF2 0.00 Hz
SPOFF4 0.00 Hz

======== CHANNEL f2 ========
CPDPRG2 waltz16
NUC2 1H
PCPD2 100.00 usec
PL2 1.60 dB
PL12 22.00 dB
SFO2 500.2225011 MHz

====== GRADIENT CHANNEL =====
GPNAM1 SINE.100
GPNAM2 SINE.100
GPX1 0.00 %
GPX2 0.00 %
GPY1 0.00 %
GPY2 0.00 %
GPZ1 30.00 %
GPZ2 50.00 %
p15 500.00 usec
p16 1000.00 usec

F2 - Processing parameters
SI 65536
SF 125.7804190 MHz
WDW EM
SSB 0
LB 1.00 Hz
GB 0
PC 2.00

1D NMR plot parameters
CX 22.80 cm
CY 30.00 cm
F1P 230.000 ppm
F1 28929.50 Hz
F2P -10.000 ppm
F2 -1257.80 Hz
PPMCM 10.52632 ppm/cm
HZCM 1324.00439 Hz/cm

1H spectrum

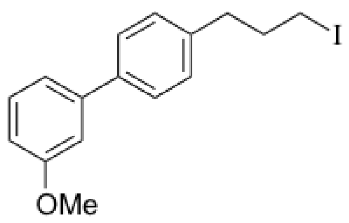

6

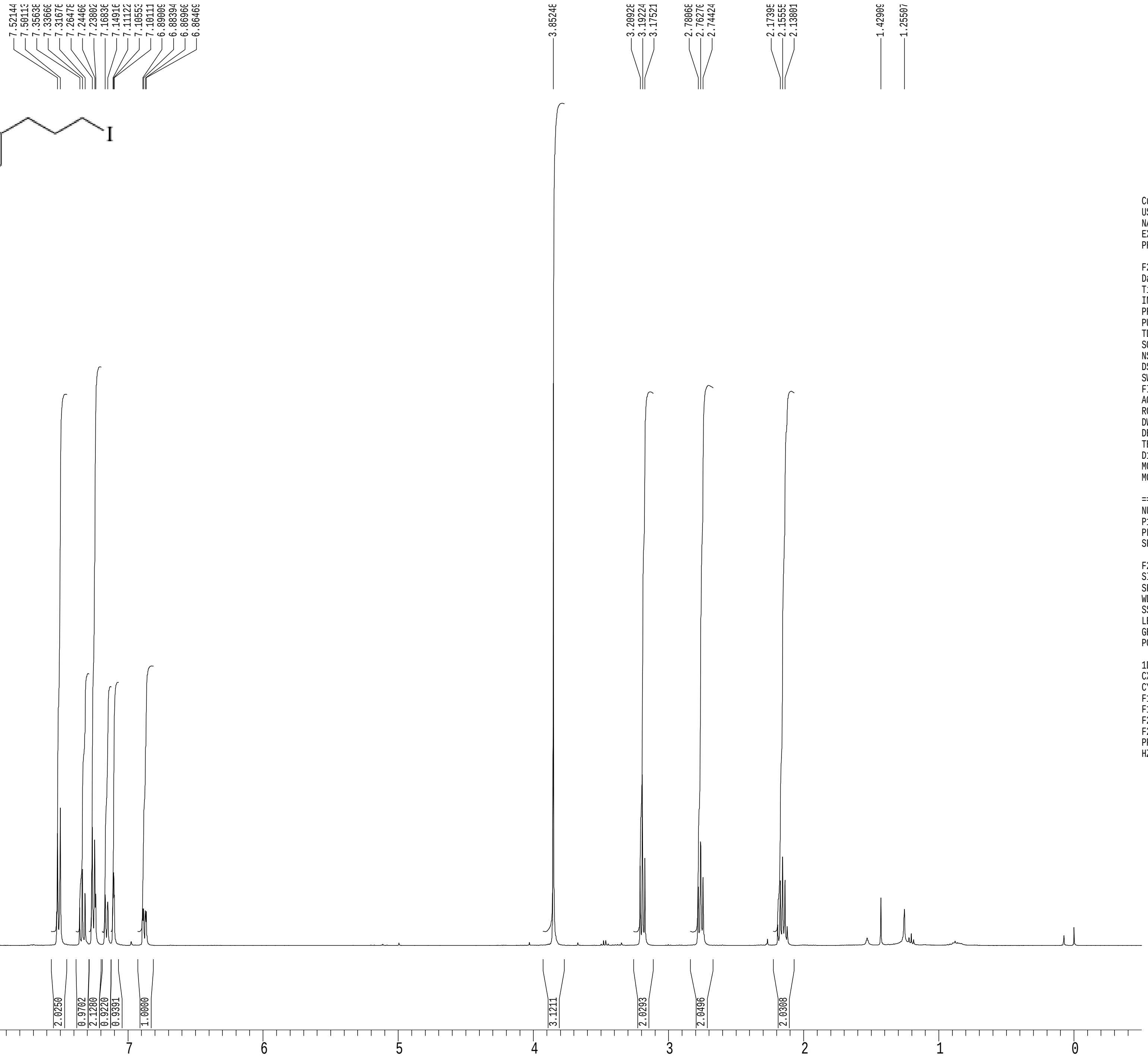

Current Data Parameters
USER nhirbawi
NAME NH-2-54-column
EXPNO 1
PROCNO 1

F2 - Acquisition Parameters
Date_ 20210915
Time 13.58
INSTRUM drx400
PROBHD 5 mm Multinucl
PULPROG zg30
TD 65536
SOLVENT CDCl3T
NS 8
DS 2
SWH 6410.256 Hz
FIDRES 0.097813 Hz
AQ 5.1118579 sec
RG 228.1
DW 78.000 usec
DE 4.50 usec
TE 298.0 K
D1 0.10000000 sec
MCREST 0.00000000 sec
MCWRK 0.01500000 sec

======== CHANNEL f1 ========
NUC1 1H
P1 12.00 usec
PL1 -1.10 dB
SFO1 400.1328009 MHz

F2 - Processing parameters
SI 65536
SF 400.1300307 MHz
WDW EM
SSB 0
LB 0.30 Hz
GB 0
PC 2.00

1D NMR plot parameters
CX 22.80 cm
CY 10.00 cm
F1P 9.000 ppm
F1 3601.17 Hz
F2P -0.500 ppm
F2 -200.06 Hz
PPMCM 0.41667 ppm/cm
HZCM 166.72086 Hz/cm

Z-restored spin-echo 13C spectrum with 1H decoupling

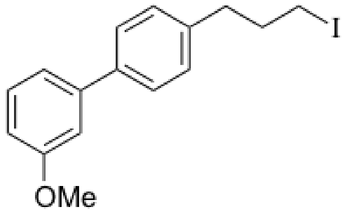

6

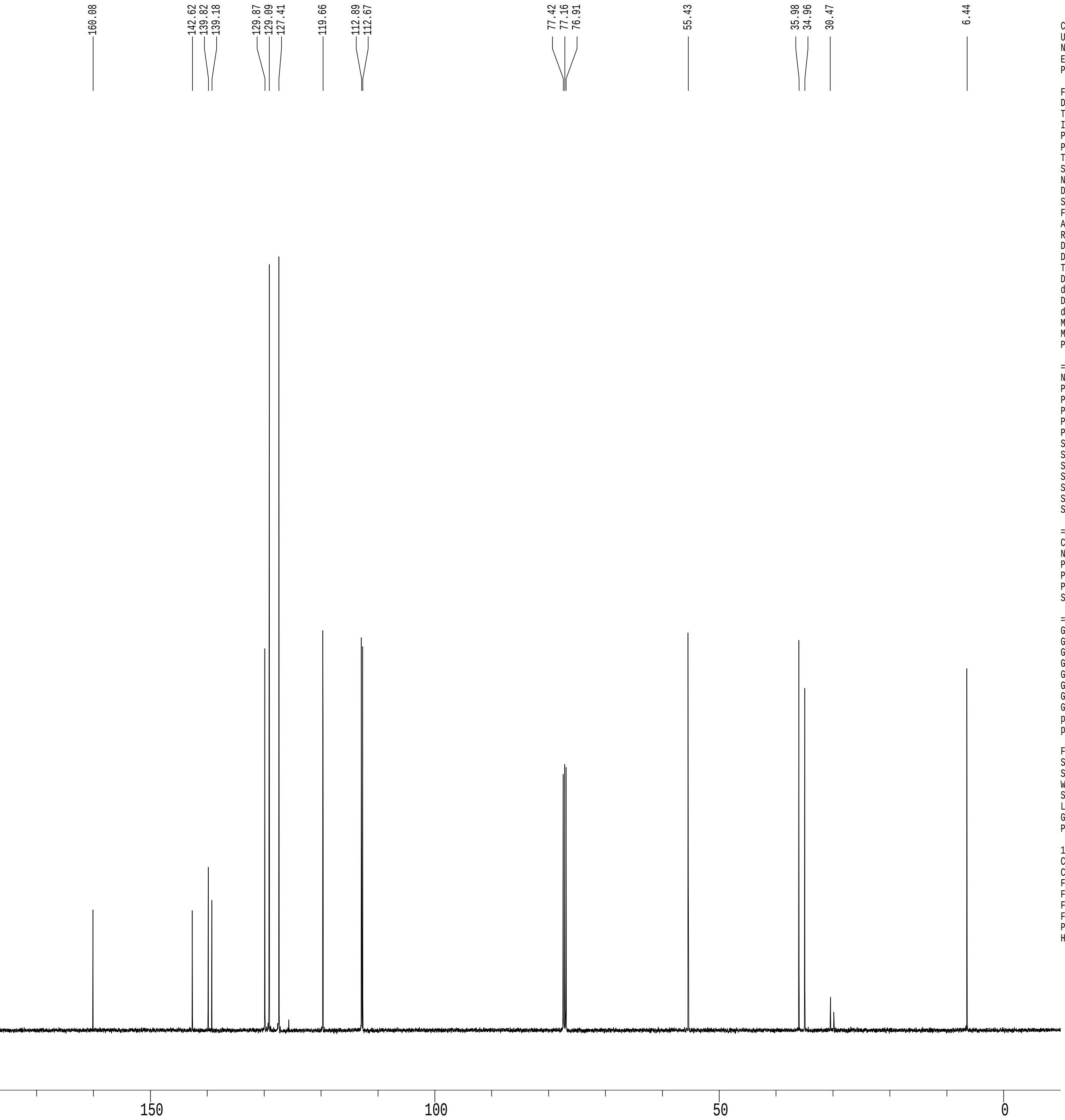


```
Current Data Parameters
USER           nhirbawi
NAME     NH-2-63-carbon
EXPNO                 2
PROCNO                1

F2 - Acquisition Parameters
Date_          20210921
Time              12.08
INSTRUM         cryo500
PROBHD   5 mm CPTCI 1H-
PULPROG  SpinEchopg30gp2.prd
TD                65536
SOLVENT           CDCl3
NS                  512
DS                   16
SWH          30303.031 Hz
FIDRES        0.462388 Hz
AQ           1.0813940 sec
RG                16384
DW               16.500 usec
DE                 6.00 usec
TE                298.0 K
D1           0.25000000 sec
d11          0.03000000 sec
D16          0.00020000 sec
d17          0.00019600 sec
MCREST       0.00000000 sec
MCWRK        0.01500000 sec
P2                37.70 usec

======== CHANNEL f1 ========
NUC1                13C
P1                18.85 usec
P12             2000.00 usec
P20              500.00 usec
PL0              120.00 dB
PL1               -1.00 dB
SFO1       125.7942548 MHz
SP2                1.55 dB
SP4                1.55 dB
SPNAM2      Crp60comp.4
SPNAM4   Crp60,0.5,20.1
SPOFF2             0.00 Hz
SPOFF4             0.00 Hz

======== CHANNEL f2 ========
CPDPRG2         waltz16
NUC2                 1H
PCPD2            100.00 usec
PL2                1.60 dB
PL12              22.00 dB
SFO2       500.2225011 MHz

====== GRADIENT CHANNEL =====
GPNAM1         SINE.100
GPNAM2         SINE.100
GPX1               0.00 %
GPX2               0.00 %
GPY1               0.00 %
GPY2               0.00 %
GPZ1              30.00 %
GPZ2              50.00 %
p15              500.00 usec
p16             1000.00 usec

F2 - Processing parameters
SI                65536
SF          125.7804108 MHz
WDW                  EM
SSB                   0
LB                 1.00 Hz
GB                    0
PC                 2.00

1D NMR plot parameters
CX                22.80 cm
CY                13.00 cm
F1P             230.000 ppm
F1             28929.49 Hz
F2P             -10.000 ppm
F2             -1257.80 Hz
PPMCM          10.52632 ppm/cm
HZCM         1324.00439 Hz/cm
```

1H spectrum

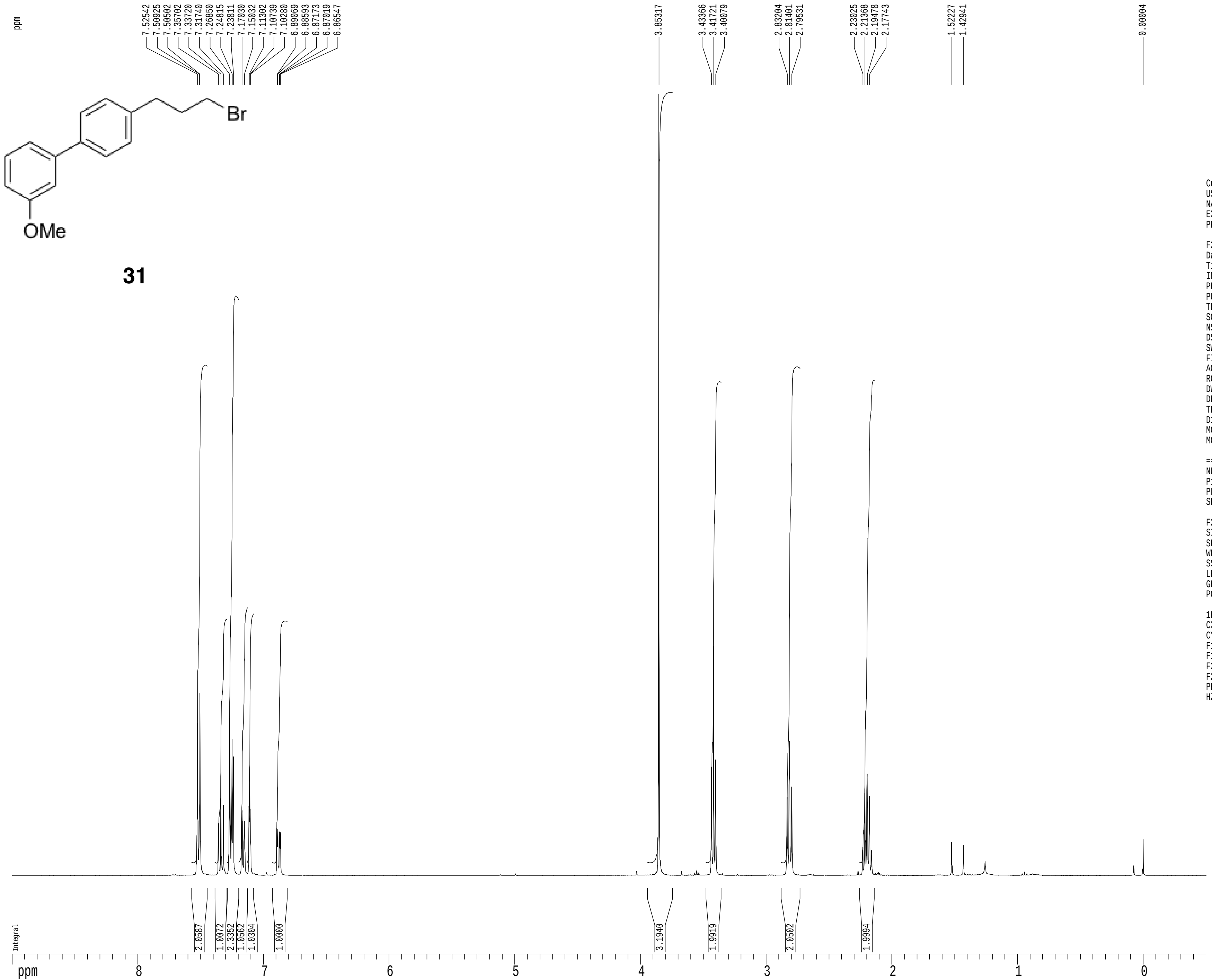

31

```
Current Data Parameters
USER           nhirbawi
NAME      NH-2-147-column
EXPNO                 1
PROCNO                1

F2 - Acquisition Parameters
Date_          20220117
Time              11.05
INSTRUM          drx400
PROBHD   5 mm QNP H/F/P
PULPROG            zg30
TD                65536
SOLVENT          CDCl3T
NS                    8
DS                    2
SWH            6410.256 Hz
FIDRES         0.097813 Hz
AQ            5.1118579 sec
RG                161.3
DW               78.000 usec
DE                 4.50 usec
TE                298.0 K
D1           0.10000000 sec
MCREST       0.00000000 sec
MCWRK        0.01500000 sec

======== CHANNEL f1 ========
NUC1                 1H
P1                12.00 usec
PL1               -0.90 dB
SFO1        400.1328009 MHz

F2 - Processing parameters
SI                65536
SF          400.1300301 MHz
WDW                  EM
SSB                   0
LB                 0.30 Hz
GB                    0
PC                 2.00

1D NMR plot parameters
CX                22.80 cm
CY                15.00 cm
F1P               9.000 ppm
F1              3601.17 Hz
F2P              -0.500 ppm
F2              -200.06 Hz
PPMCM           0.41667 ppm/cm
HZCM          166.72086 Hz/cm
```

Z-restored spin-echo 13C spectrum with 1H decoupling

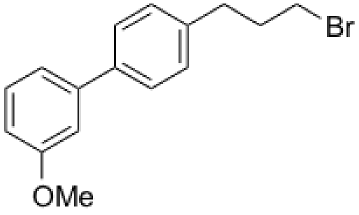

31

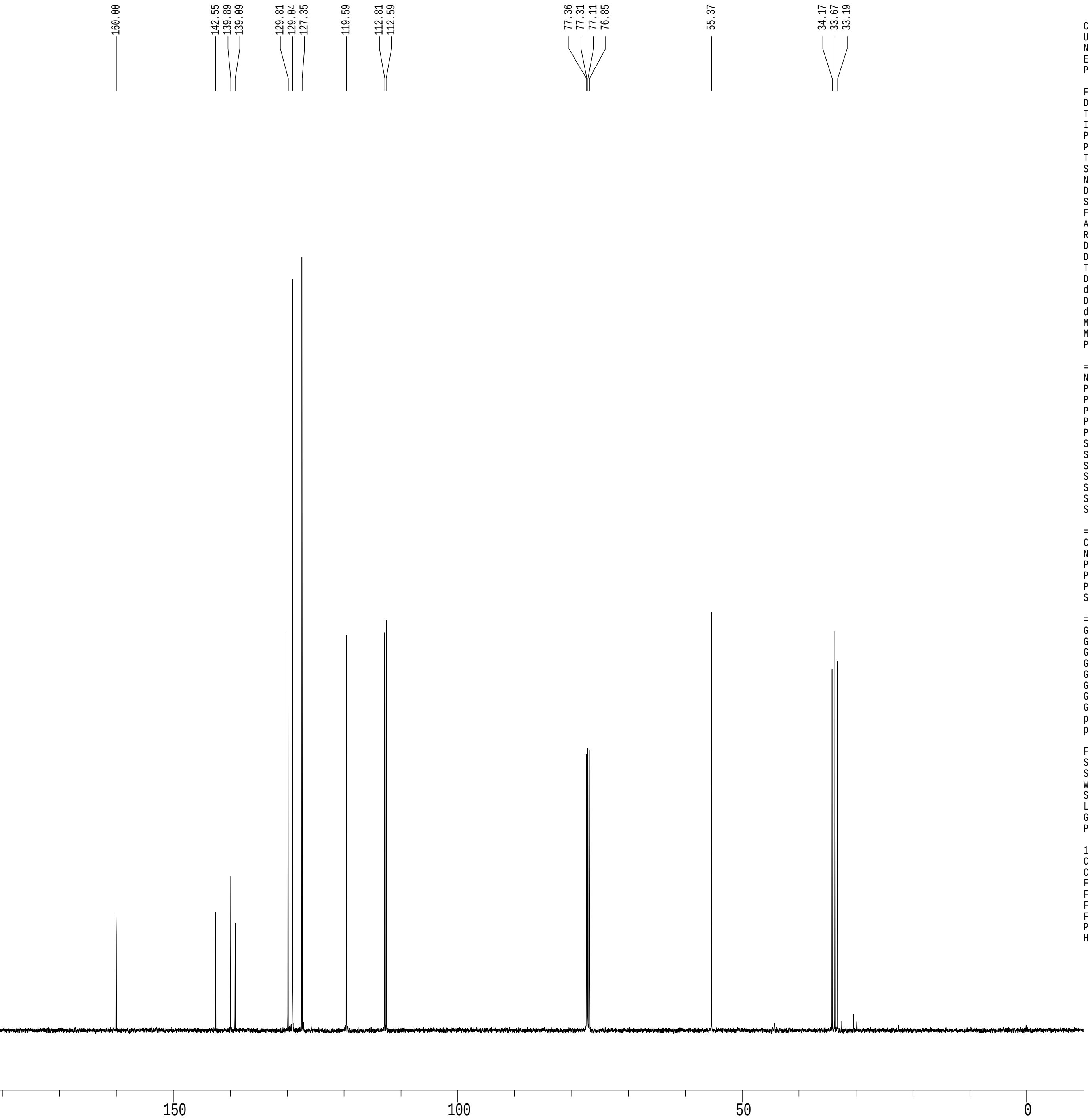

Current Data Parameters
USER nhirbawi
NAME NH-2-147-HandC
EXPNO 2
PROCNO 1

F2 - Acquisition Parameters
Date_ 20220119
Time 16.14
INSTRUM cryo500
PROBHD 5 mm CPTCI 1H-
PULPROG SpinEchopg30gp2.prd
TD 65536
SOLVENT CDCl3
NS 363
DS 16
SWH 30303.031 Hz
FIDRES 0.462388 Hz
AQ 1.0813940 sec
RG 3649.1
DW 16.500 usec
DE 6.00 usec
TE 298.0 K
D1 0.25000000 sec
d11 0.03000000 sec
D16 0.00020000 sec
d17 0.00019600 sec
MCREST 0.00000000 sec
MCWRK 0.01500000 sec
P2 37.70 usec

======== CHANNEL f1 ========
NUC1 13C
P1 18.85 usec
P12 2000.00 usec
P20 500.00 usec
PL0 120.00 dB
PL1 -1.00 dB
SFO1 125.7942548 MHz
SP2 1.55 dB
SP4 1.55 dB
SPNAM2 Crp60comp.4
SPNAM4 Crp60,0.5,20.1
SPOFF2 0.00 Hz
SPOFF4 0.00 Hz

======== CHANNEL f2 ========
CPDPRG2 waltz16
NUC2 1H
PCPD2 100.00 usec
PL2 1.60 dB
PL12 22.00 dB
SFO2 500.2225011 MHz

====== GRADIENT CHANNEL =====
GPNAM1 SINE.100
GPNAM2 SINE.100
GPX1 0.00 %
GPX2 0.00 %
GPY1 0.00 %
GPY2 0.00 %
GPZ1 30.00 %
GPZ2 50.00 %
p15 500.00 usec
p16 1000.00 usec

F2 - Processing parameters
SI 65536
SF 125.7804190 MHz
WDW EM
SSB 0
LB 1.00 Hz
GB 0
PC 2.00

1D NMR plot parameters
CX 22.80 cm
CY 13.00 cm
F1P 230.000 ppm
F1 28929.50 Hz
F2P -10.000 ppm
F2 -1257.80 Hz
PPMCM 10.52632 ppm/cm
HZCM 1324.00439 Hz/cm

1H spectrum

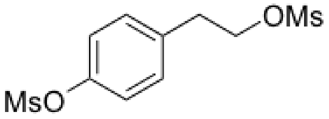

48

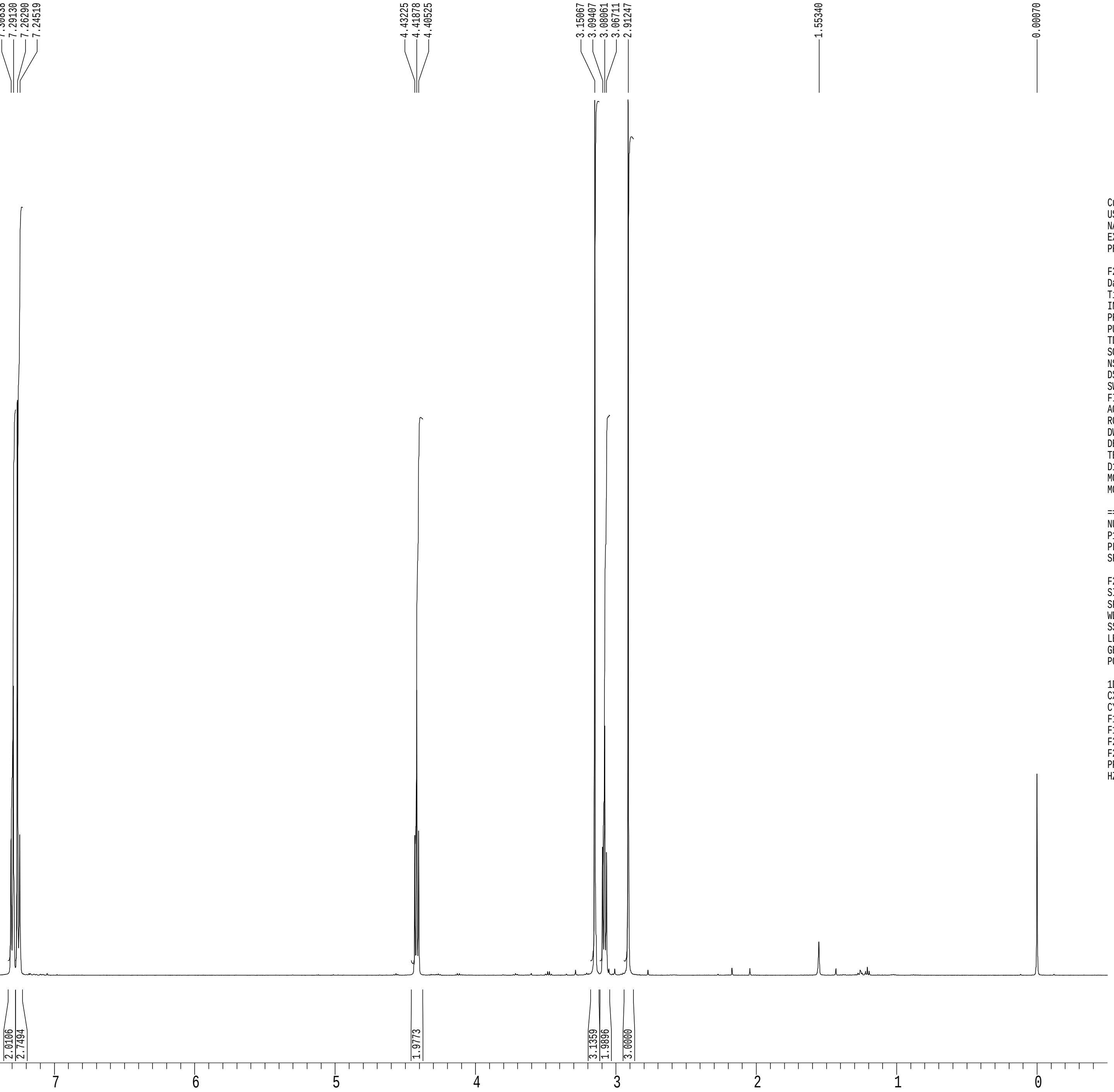

Current Data Parameters
USER nhirbawi
NAME NH-2-164-1H
EXPNO 1
PROCNO 1

F2 - Acquisition Parameters
Date_ 20220509
Time 15.58
INSTRUM cryo500
PROBHD 5 mm CPTCI 1H-
PULPROG zg30
TD 81728
SOLVENT CDCl3T
NS 8
DS 2
SWH 8012.820 Hz
FIDRES 0.098043 Hz
AQ 5.0998774 sec
RG 5.7
DW 62.400 usec
DE 6.00 usec
TE 298.0 K
D1 0.10000000 sec
MCREST 0.00000000 sec
MCWRK 0.01500000 sec

======== CHANNEL f1 ========
NUC1 1H
P1 9.75 usec
PL1 1.60 dB
SFO1 500.2235015 MHz

F2 - Processing parameters
SI 65536
SF 500.2200309 MHz
WDW EM
SSB 0
LB 0.30 Hz
GB 0
PC 1.00

1D NMR plot parameters
CX 22.80 cm
CY 15.00 cm
F1P 9.000 ppm
F1 4501.98 Hz
F2P -0.500 ppm
F2 -250.11 Hz
PPMCM 0.41667 ppm/cm
HZCM 208.42502 Hz/cm

1H spectrum

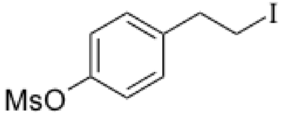

7

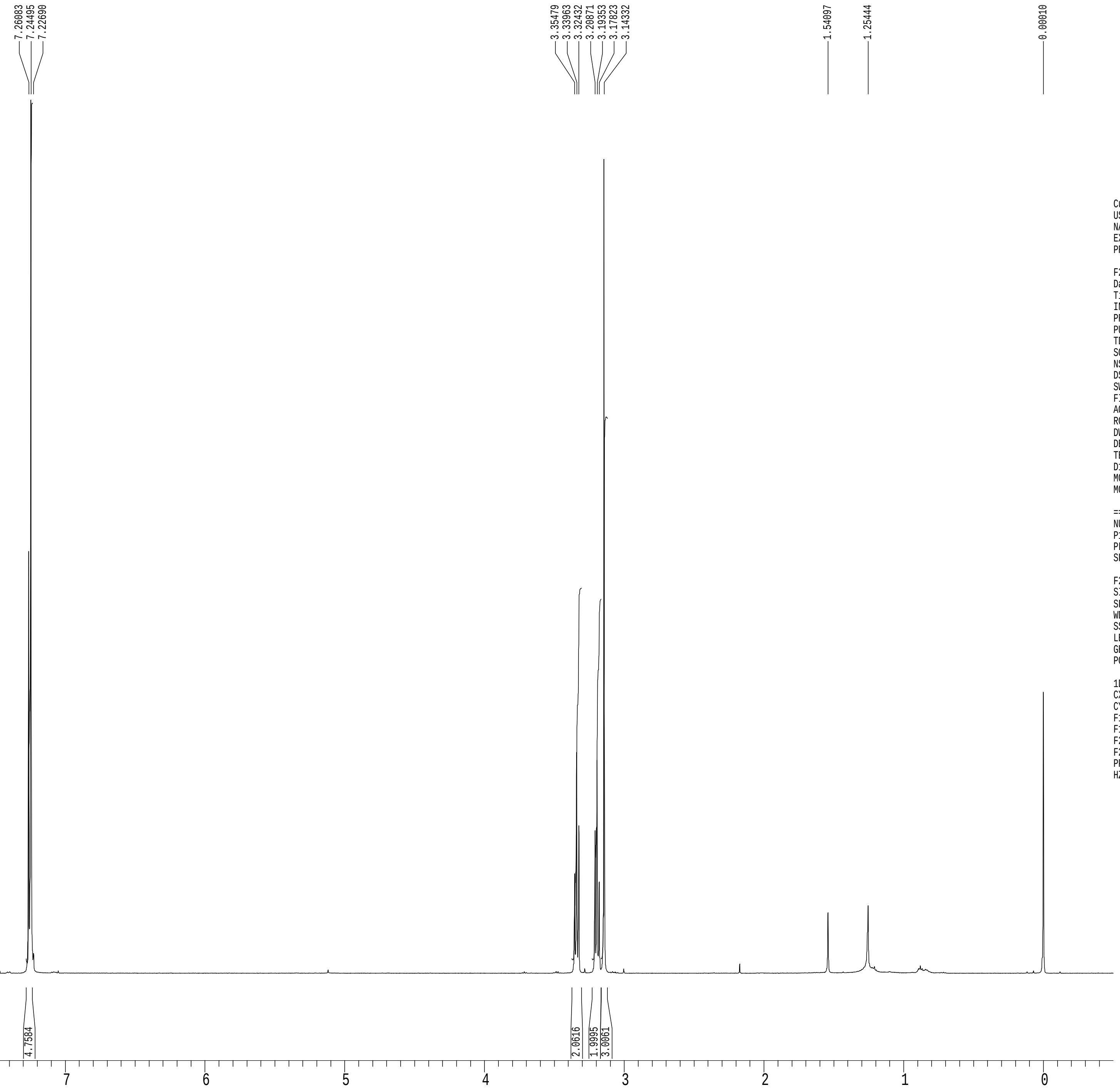


```
Current Data Parameters
USER          nhirbawi
NAME        NH-2-169-1H
EXPNO                1
PROCNO               1

F2 - Acquisition Parameters
Date_         20220509
Time             16.02
INSTRUM        cryo500
PROBHD   5 mm CPTCI 1H-
PULPROG           zg30
TD               81728
SOLVENT         CDCl3T
NS                   8
DS                   2
SWH           8012.820 Hz
FIDRES        0.098043 Hz
AQ           5.0998774 sec
RG                 6.3
DW              62.400 usec
DE                6.00 usec
TE               298.0 K
D1          0.10000000 sec
MCREST      0.00000000 sec
MCWRK       0.01500000 sec

======== CHANNEL f1 ========
NUC1                1H
P1                9.75 usec
PL1               1.60 dB
SFO1       500.2235015 MHz

F2 - Processing parameters
SI               65536
SF         500.2200319 MHz
WDW                 EM
SSB                  0
LB                0.30 Hz
GB                   0
PC                1.00

1D NMR plot parameters
CX               22.80 cm
CY               15.00 cm
F1P              9.000 ppm
F1             4501.98 Hz
F2P             -0.500 ppm
F2             -250.11 Hz
PPMCM          0.41667 ppm/cm
HZCM         208.42502 Hz/cm
```

Z-restored spin-echo 13C spectrum with 1H decoupling

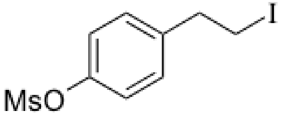

7

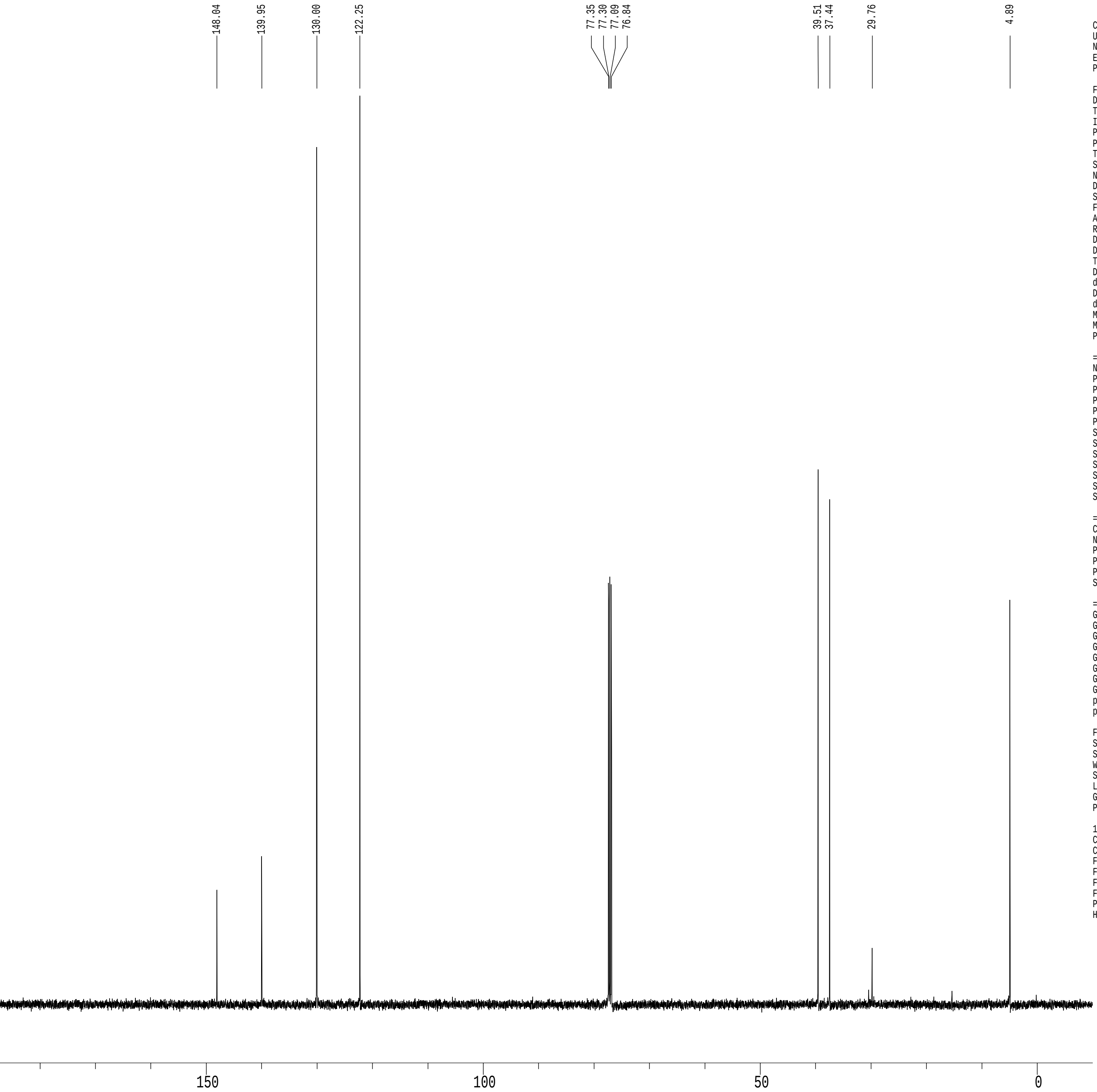


```
Current Data Parameters
USER          nhirbawi
NAME     NH-2-169-carbon
EXPNO                2
PROCNO               1

F2 - Acquisition Parameters
Date_         20220201
Time             16.29
INSTRUM        cryo500
PROBHD   5 mm CPTCI 1H-
PULPROG  SpinEchopg30gp2.prd
TD               65536
SOLVENT          CDCl3
NS                 311
DS                  16
SWH          30303.031 Hz
FIDRES        0.462388 Hz
AQ           1.0813940 sec
RG                2048
DW              16.500 usec
DE                6.00 usec
TE               298.0 K
D1          0.25000000 sec
d11         0.03000000 sec
D16         0.00020000 sec
d17         0.00019600 sec
MCREST      0.00000000 sec
MCWRK       0.01500000 sec
P2               37.70 usec

======== CHANNEL f1 ========
NUC1               13C
P1               18.85 usec
P12            2000.00 usec
P20             500.00 usec
PL0             120.00 dB
PL1              -1.00 dB
SFO1       125.7942548 MHz
SP2               1.55 dB
SP4               1.55 dB
SPNAM2     Crp60comp.4
SPNAM4   Crp60,0.5,20.1
SPOFF2            0.00 Hz
SPOFF4            0.00 Hz

======== CHANNEL f2 ========
CPDPRG2        waltz16
NUC2                1H
PCPD2           100.00 usec
PL2               1.60 dB
PL12             22.00 dB
SFO2       500.2225011 MHz

====== GRADIENT CHANNEL =====
GPNAM1        SINE.100
GPNAM2        SINE.100
GPX1              0.00 %
GPX2              0.00 %
GPY1              0.00 %
GPY2              0.00 %
GPZ1             30.00 %
GPZ2             50.00 %
p15             500.00 usec
p16            1000.00 usec

F2 - Processing parameters
SI               65536
SF         125.7804190 MHz
WDW                 EM
SSB                  0
LB                1.00 Hz
GB                   0
PC                2.00

1D NMR plot parameters
CX               22.80 cm
CY               15.65 cm
F1P            230.000 ppm
F1            28929.50 Hz
F2P            -10.000 ppm
F2            -1257.80 Hz
PPMCM         10.52632 ppm/cm
HZCM        1324.00439 Hz/cm
```

1H spectrum

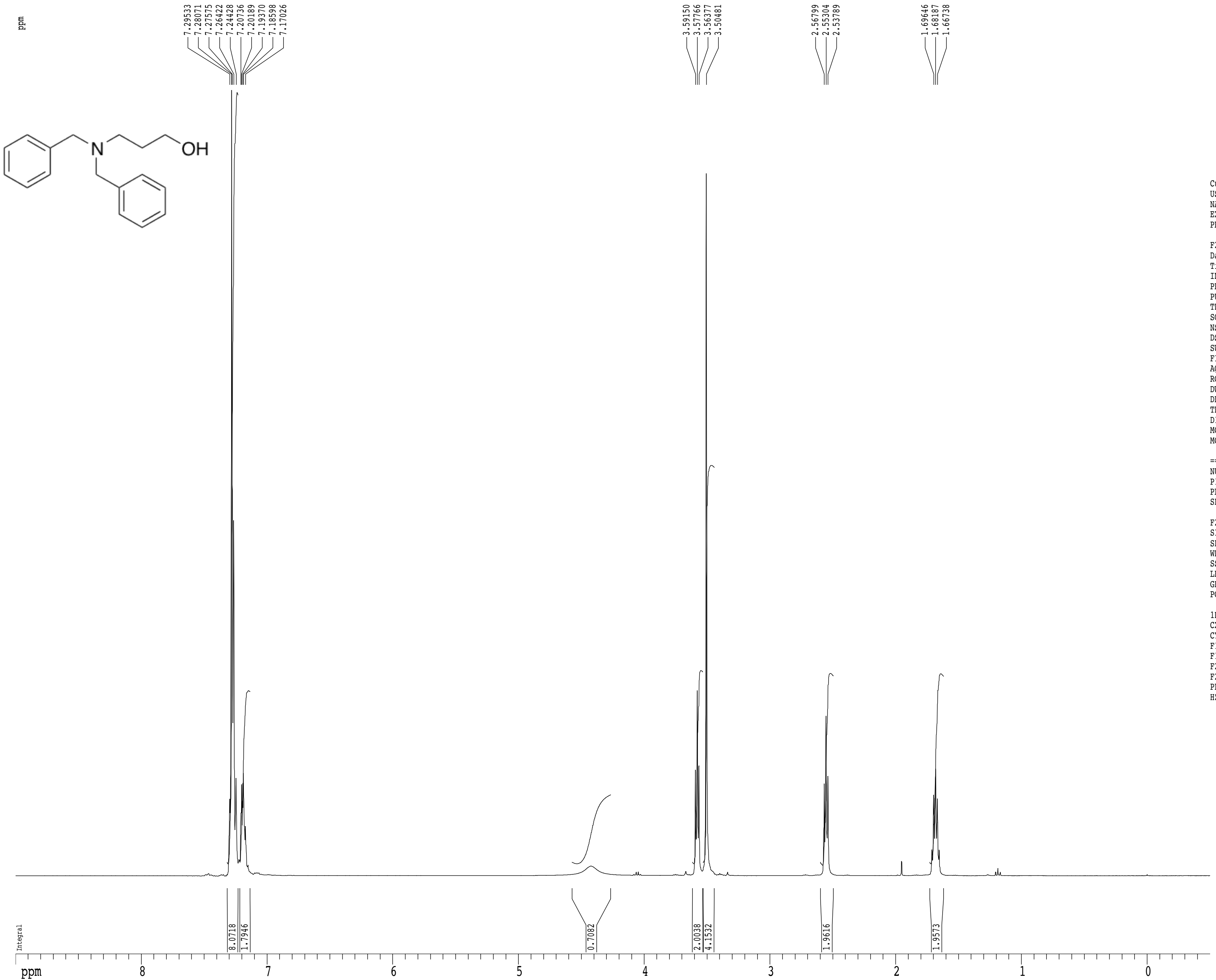


```
Current Data Parameters
USER              linpc2
NAME           pcl-3-020
EXPNO                  1
PROCNO                 1

F2 - Acquisition Parameters
Date_           20220126
Time               13.02
INSTRUM           drx400
PROBHD   5 mm QNP H/F/P
PULPROG             zg30
TD                 65536
SOLVENT            CDCl3
NS                     8
DS                     2
SWH             6410.256 Hz
FIDRES          0.097813 Hz
AQ             5.1118579 sec
RG                  14.3
DW                78.000 usec
DE                  4.50 usec
TE                 298.0 K
D1            0.10000000 sec
MCREST        0.00000000 sec
MCWRK         0.01500000 sec

======== CHANNEL f1 ========
NUC1                  1H
P1                 12.00 usec
PL1                -0.90 dB
SFO1         400.1328009 MHz

F2 - Processing parameters
SI                 65536
SF           400.1300648 MHz
WDW                   EM
SSB                    0
LB                  0.30 Hz
GB                     0
PC                  2.00

1D NMR plot parameters
CX                 22.80 cm
CY                 15.00 cm
F1P                9.000 ppm
F1               3601.17 Hz
F2P               -0.500 ppm
F2               -200.06 Hz
PPMCM            0.41667 ppm/cm
HZCM           166.72087 Hz/cm
```

1H spectrum

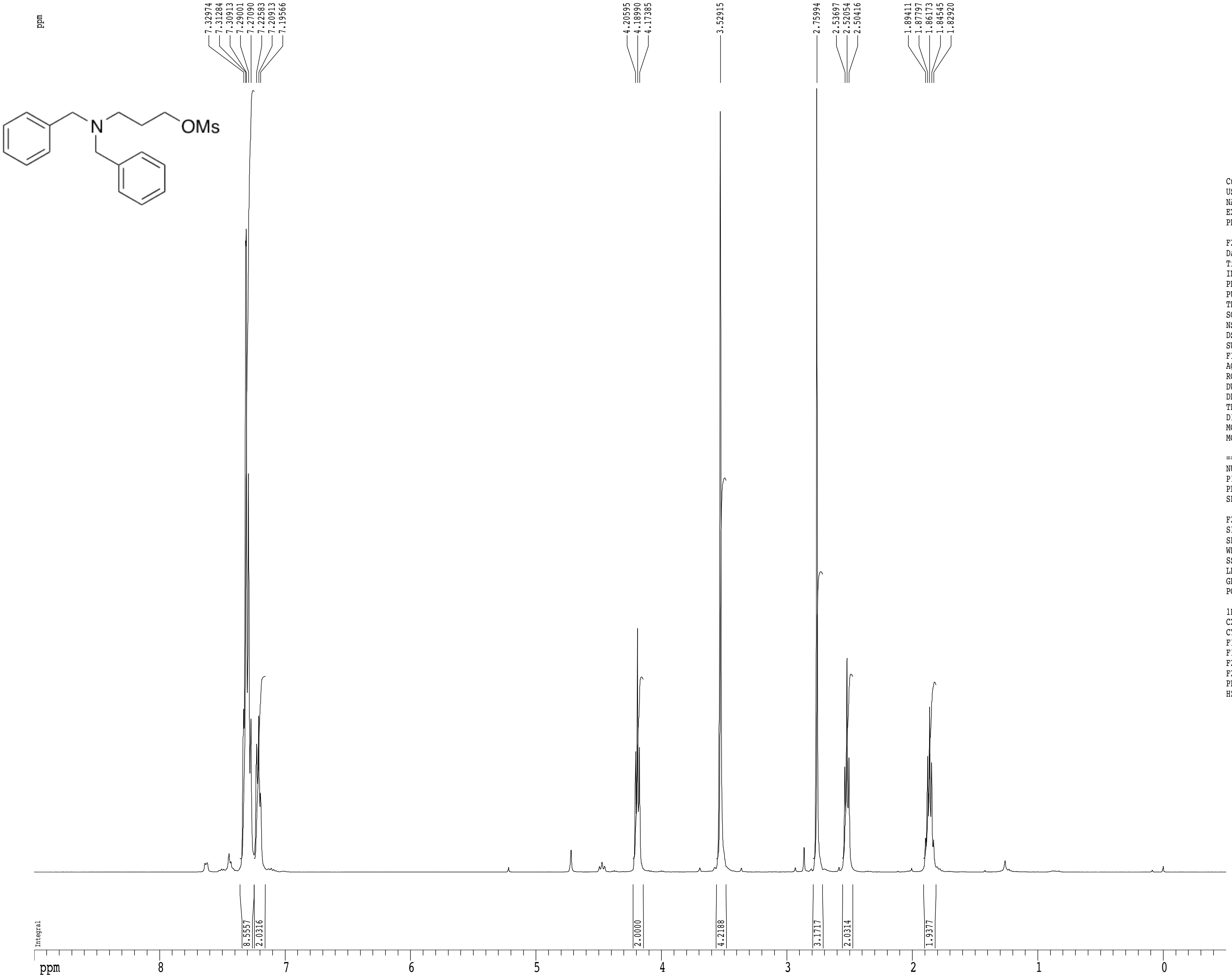


```
Current Data Parameters
USER              linpc2
NAME           pcl-3-030
EXPNO                  2
PROCNO                 1

F2 - Acquisition Parameters
Date_           20220209
Time               12.26
INSTRUM           drx400
PROBHD   5 mm QNP H/F/P
PULPROG             zg30
TD                 65536
SOLVENT            CDCl3
NS                     8
DS                     2
SWH             6410.256 Hz
FIDRES          0.097813 Hz
AQ             5.1118579 sec
RG                    32
DW                78.000 usec
DE                  4.50 usec
TE                 298.0 K
D1            0.10000000 sec
MCREST        0.00000000 sec
MCWRK         0.01500000 sec

======== CHANNEL f1 ========
NUC1                  1H
P1                 12.00 usec
PL1                -0.90 dB
SFO1         400.1328009 MHz

F2 - Processing parameters
SI                 65536
SF           400.1300464 MHz
WDW                   EM
SSB                    0
LB                  0.30 Hz
GB                     0
PC                  2.00

1D NMR plot parameters
CX                 22.80 cm
CY                 15.00 cm
F1P                9.000 ppm
F1               3601.17 Hz
F2P               -0.500 ppm
F2               -200.06 Hz
PPMCM            0.41667 ppm/cm
HZCM           166.72086 Hz/cm
```

1H spectrum

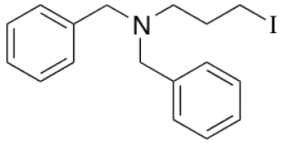

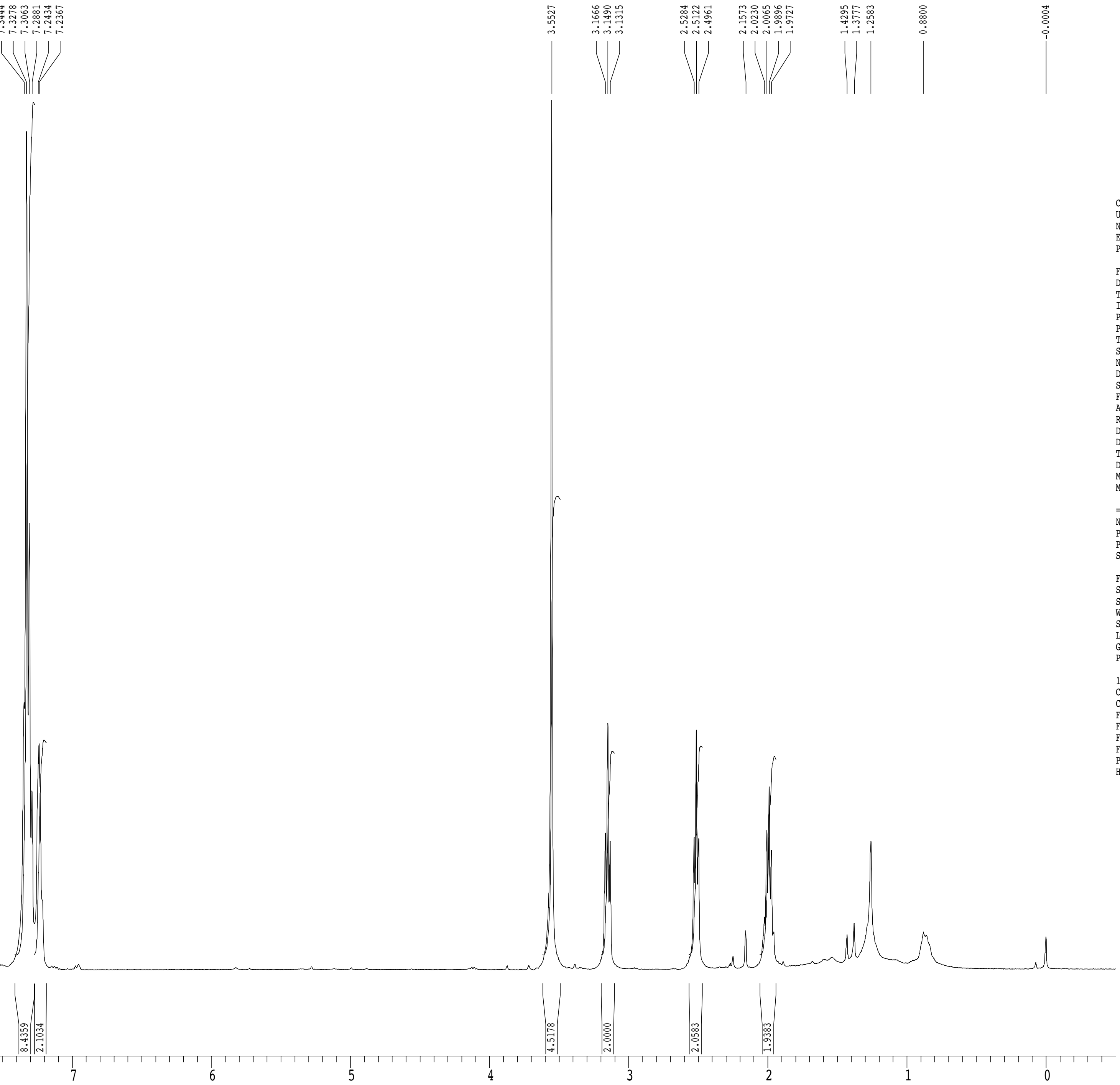


```
Current Data Parameters
USER              linpc2
NAME           pcl-3-043
EXPNO                  2
PROCNO                 1

F2 - Acquisition Parameters
Date_           20220211
Time               15.10
INSTRUM           drx400
PROBHD   5 mm QNP H/F/P
PULPROG             zg30
TD                 65536
SOLVENT            CDCl3
NS                     8
DS                     2
SWH            6410.256 Hz
FIDRES         0.097813 Hz
AQ            5.1118579 sec
RG                 143.7
DW                78.000 usec
DE                  4.50 usec
TE                 298.0 K
D1            0.10000000 sec
MCREST        0.00000000 sec
MCWRK         0.01500000 sec

======== CHANNEL f1 ========
NUC1                  1H
P1                 12.00 usec
PL1                -0.90 dB
SFO1         400.1328009 MHz

F2 - Processing parameters
SI                 65536
SF           400.1300297 MHz
WDW                   EM
SSB                    0
LB                  0.30 Hz
GB                     0
PC                  2.00

1D NMR plot parameters
CX                 22.80 cm
CY                 15.00 cm
F1P                9.000 ppm
F1               3601.17 Hz
F2P               -0.500 ppm
F2               -200.06 Hz
PPMCM            0.41667 ppm/cm
HZCM           166.72086 Hz/cm
```

1H spectrum

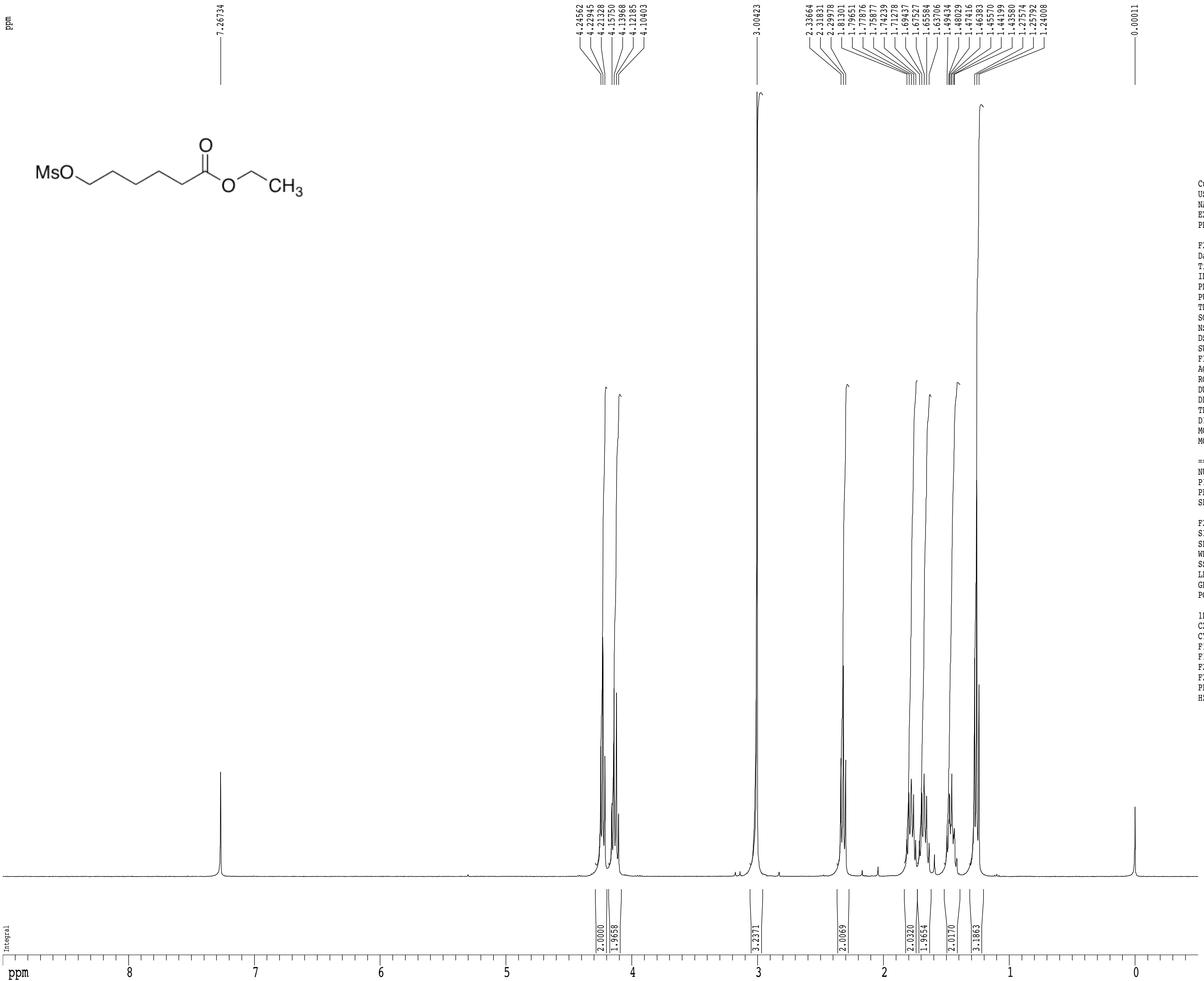

Current Data Parameters
USER linpc2
NAME pcl-3-021
EXPNO 1
PROCNO 1

F2 - Acquisition Parameters
Date_ 20220127
Time 9.54
INSTRUM drx400
PROBHD 5 mm QNP H/F/P
PULPROG zg30
TD 65536
SOLVENT CDCl3
NS 8
DS 2
SWH 6410.256 Hz
FIDRES 0.097813 Hz
AQ 5.1118579 sec
RG 256
DW 78.000 usec
DE 4.50 usec
TE 298.0 K
D1 0.10000000 sec
MCREST 0.00000000 sec
MCWRK 0.01500000 sec

======== CHANNEL f1 ========
NUC1 1H
P1 12.00 usec
PL1 -0.90 dB
SFO1 400.1328009 MHz

F2 - Processing parameters
SI 65536
SF 400.1300181 MHz
WDW EM
SSB 0
LB 0.30 Hz
GB 0
PC 2.00

1D NMR plot parameters
CX 22.80 cm
CY 15.00 cm
F1P 9.000 ppm
F1 3601.17 Hz
F2P -0.500 ppm
F2 -200.06 Hz
PPMCM 0.41667 ppm/cm
HZCM 166.72084 Hz/cm

1H spectrum

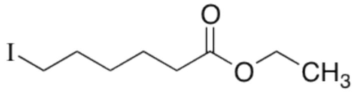

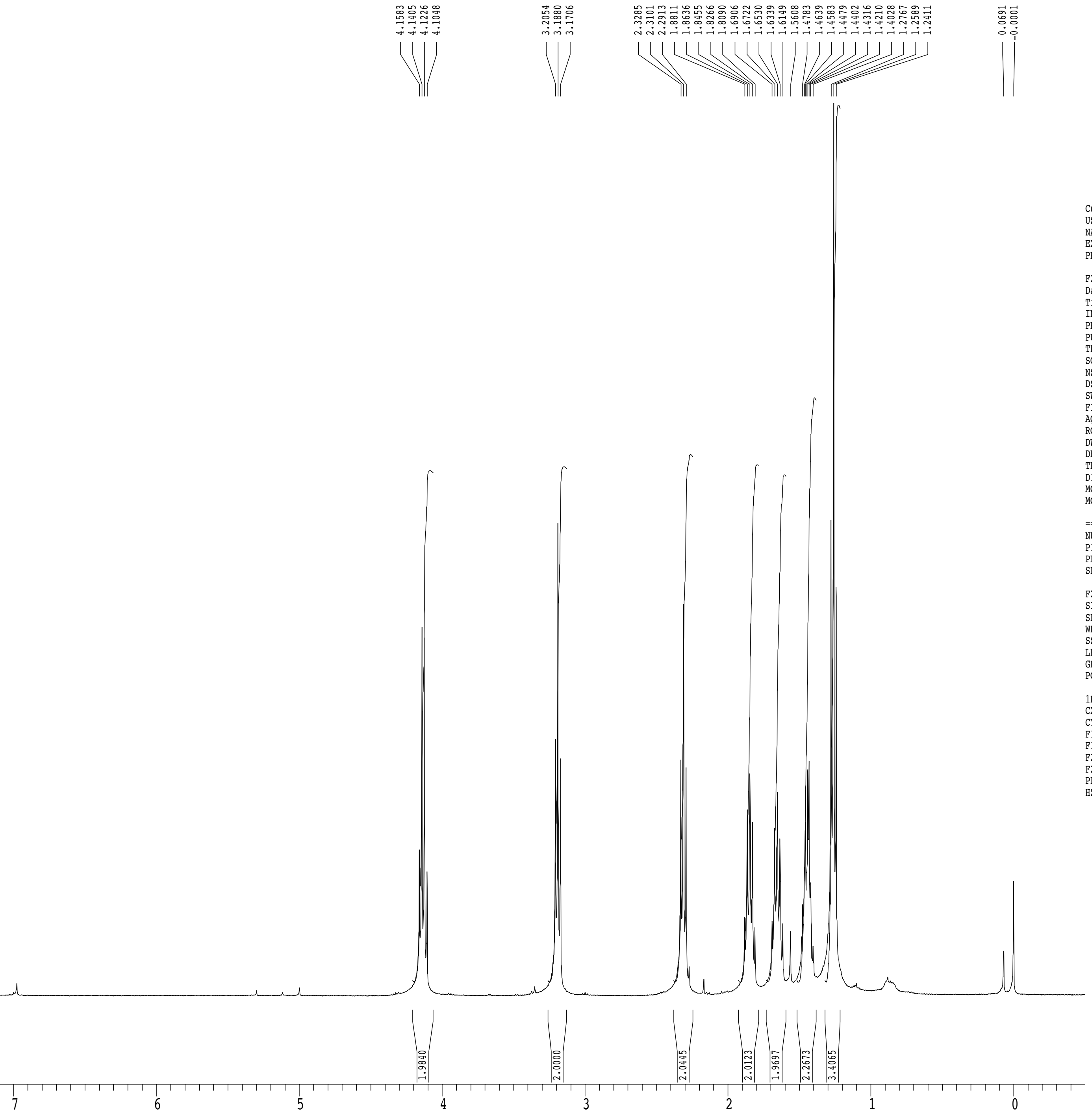


```
Current Data Parameters
USER              linpc2
NAME           pcl-3-029
EXPNO                  2
PROCNO                 1

F2 - Acquisition Parameters
Date_           20220205
Time               13.16
INSTRUM           drx400
PROBHD   5 mm QNP H/F/P
PULPROG             zg30
TD                 65536
SOLVENT            CDCl3
NS                     8
DS                     2
SWH            6410.256 Hz
FIDRES         0.097813 Hz
AQ            5.1118579 sec
RG                 287.4
DW                78.000 usec
DE                  4.50 usec
TE                 298.0 K
D1            0.10000000 sec
MCREST        0.00000000 sec
MCWRK         0.01500000 sec

======== CHANNEL f1 ========
NUC1                  1H
P1                 12.00 usec
PL1                -0.90 dB
SFO1         400.1328009 MHz

F2 - Processing parameters
SI                 65536
SF           400.1300198 MHz
WDW                   EM
SSB                    0
LB                  0.30 Hz
GB                     0
PC                  2.00

1D NMR plot parameters
CX                 22.80 cm
CY                 15.00 cm
F1P                9.000 ppm
F1               3601.17 Hz
F2P               -0.500 ppm
F2               -200.06 Hz
PPMCM            0.41667 ppm/cm
HZCM           166.72084 Hz/cm
```

1H spectrum

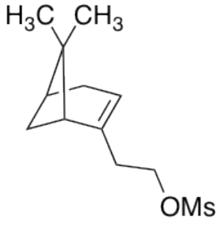

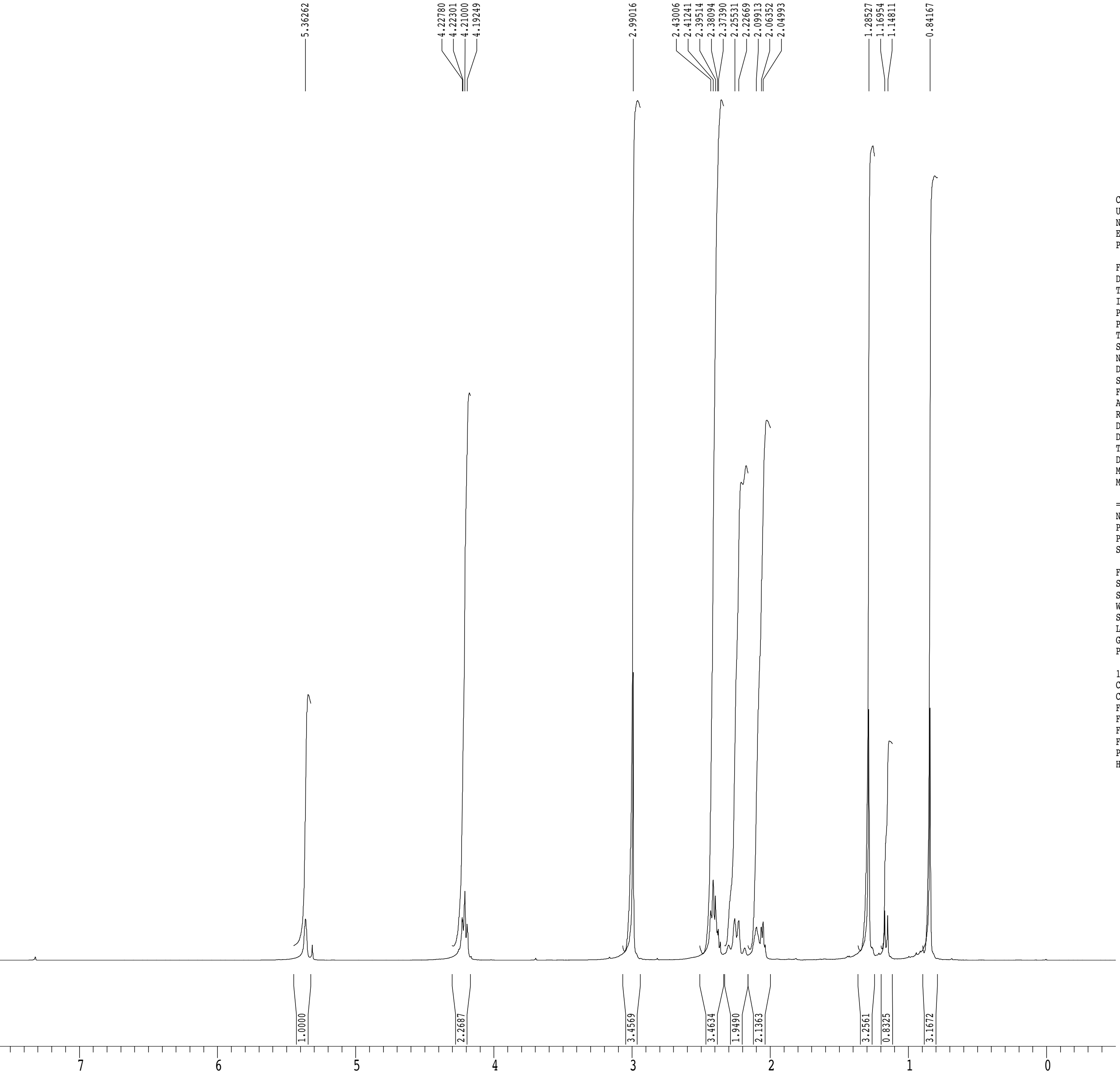

Current Data Parameters
USER linpc2
NAME pcl-2-297
EXPNO 1
PROCNO 1

F2 - Acquisition Parameters
Date_ 20220117
Time 10.45
INSTRUM drx400
PROBHD 5 mm QNP H/F/P
PULPROG zg30
TD 65536
SOLVENT CDCl3
NS 8
DS 2
SWH 6410.256 Hz
FIDRES 0.097813 Hz
AQ 5.1118579 sec
RG 32
DW 78.000 usec
DE 4.50 usec
TE 298.0 K
D1 0.10000000 sec
MCREST 0.00000000 sec
MCWRK 0.01500000 sec

======== CHANNEL f1 ========
NUC1 1H
P1 12.00 usec
PL1 -0.90 dB
SFO1 400.1328009 MHz

F2 - Processing parameters
SI 65536
SF 400.1299973 MHz
WDW EM
SSB 0
LB 0.30 Hz
GB 0
PC 2.00

1D NMR plot parameters
CX 22.80 cm
CY 5.00 cm
F1P 9.000 ppm
F1 3601.17 Hz
F2P -0.500 ppm
F2 -200.06 Hz
PPMCM 0.41667 ppm/cm
HZCM 166.72084 Hz/cm

13C spectrum with 1H decoupling

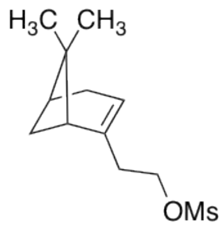

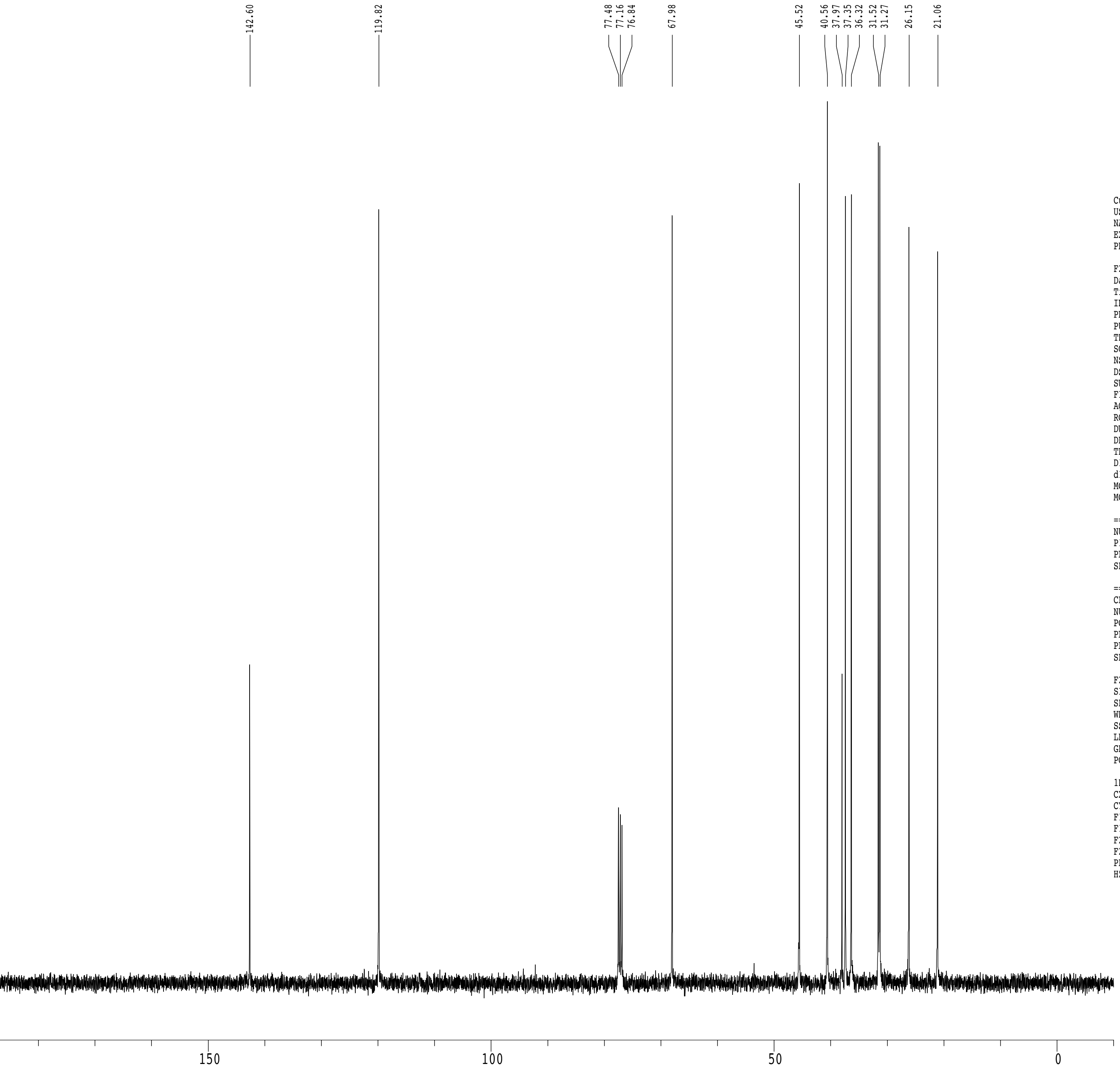


```
Current Data Parameters
USER            linpc2
NAME          pcl-2-297
EXPNO                2
PROCNO               1

F2 - Acquisition Parameters
Date_         20220117
Time             10.48
INSTRUM         drx400
PROBHD   5 mm QNP H/F/P
PULPROG         zgdc30
TD               65536
SOLVENT          CDCl3
NS                  80
DS                   4
SWH          24154.590 Hz
FIDRES        0.368570 Hz
AQ           1.3566452 sec
RG              9195.2
DW              20.700 usec
DE               20.39 usec
TE               298.0 K
D1          0.10000000 sec
d11         0.03000000 sec
MCREST      0.00000000 sec
MCWRK       0.01500000 sec

======== CHANNEL f1 ========
NUC1               13C
P1                7.90 usec
PL1              -3.00 dB
SFO1       100.6237964 MHz

======== CHANNEL f2 ========
CPDPRG2        waltz16
NUC2                1H
PCPD2            90.00 usec
PL2              -0.90 dB
PL12             17.00 dB
SFO2       400.1328009 MHz

F2 - Processing parameters
SI               65536
SF         100.6127757 MHz
WDW                 EM
SSB                  0
LB                1.00 Hz
GB                   0
PC                1.00

1D NMR plot parameters
CX               22.80 cm
CY               15.50 cm
F1P            220.000 ppm
F1            22134.81 Hz
F2P            -10.000 ppm
F2            -1006.13 Hz
PPMCM         10.08772 ppm/cm
HZCM        1014.95349 Hz/cm
```

1H spectrum

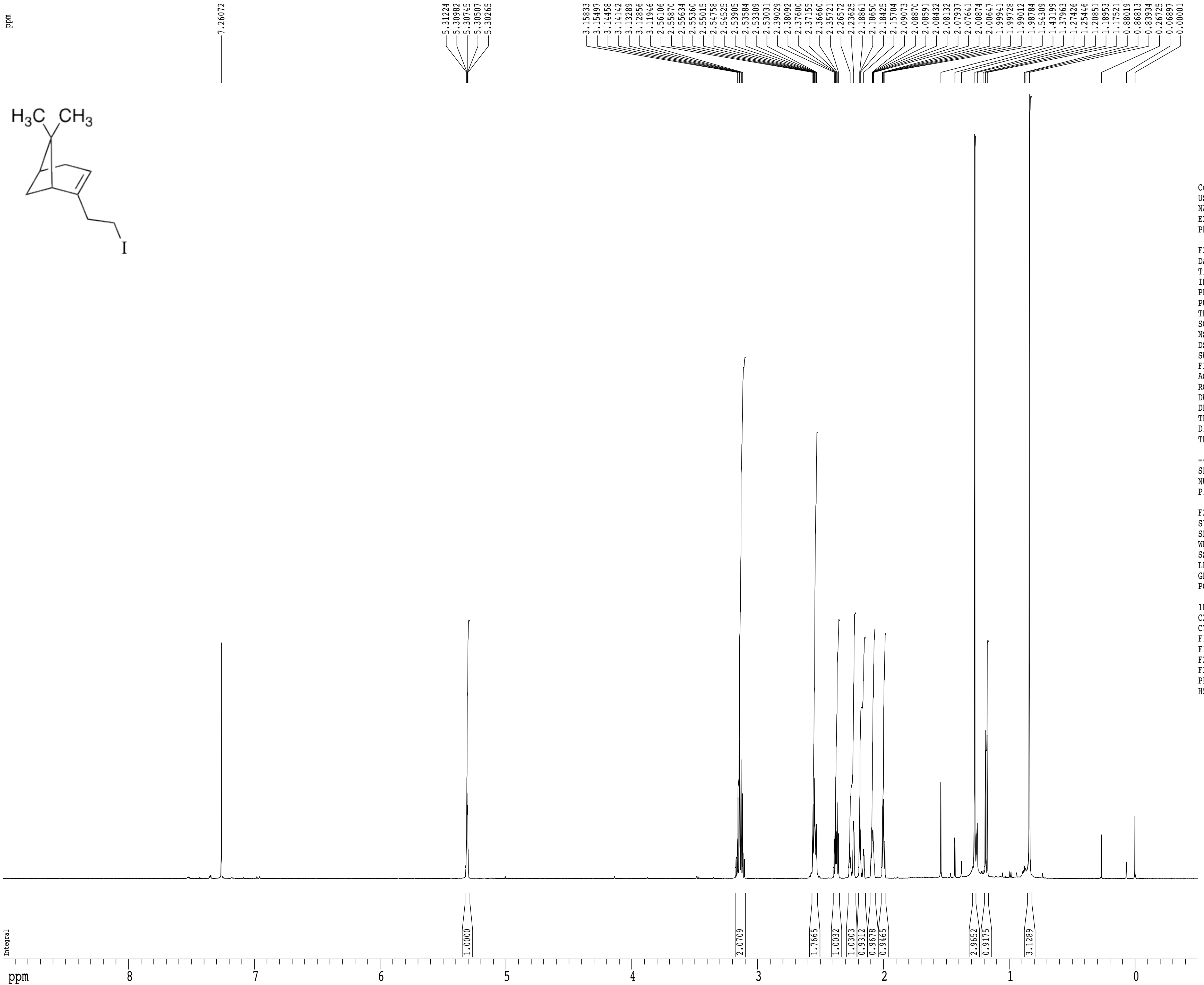

Current Data Parameters
USER linpc2
NAME pcl-2-303
EXPNO 4
PROCNO 1

F2 - Acquisition Parameters
Date_ 20220119
Time 13.36
INSTRUM av600
PROBHD 5 mm CPBBO BB-
PULPROG zg30
TD 98074
SOLVENT CDCl3T
NS 8
DS 2
SWH 9615.385 Hz
FIDRES 0.098042 Hz
AQ 5.0998979 sec
RG 10
DW 52.000 usec
DE 14.23 usec
TE 297.9 K
D1 0.10000000 sec
TD0 1

======== CHANNEL f1 ========
SFO1 600.1342009 MHz
NUC1 1H
P1 9.50 usec

F2 - Processing parameters
SI 65536
SF 600.1300351 MHz
WDW no
SSB 0
LB 0.00 Hz
GB 0
PC 1.00

1D NMR plot parameters
CX 22.80 cm
CY 15.00 cm
F1P 9.000 ppm
F1 5401.17 Hz
F2P -0.500 ppm
F2 -300.06 Hz
PPMCM 0.41667 ppm/cm
HZCM 250.05418 Hz/cm

1H spectrum

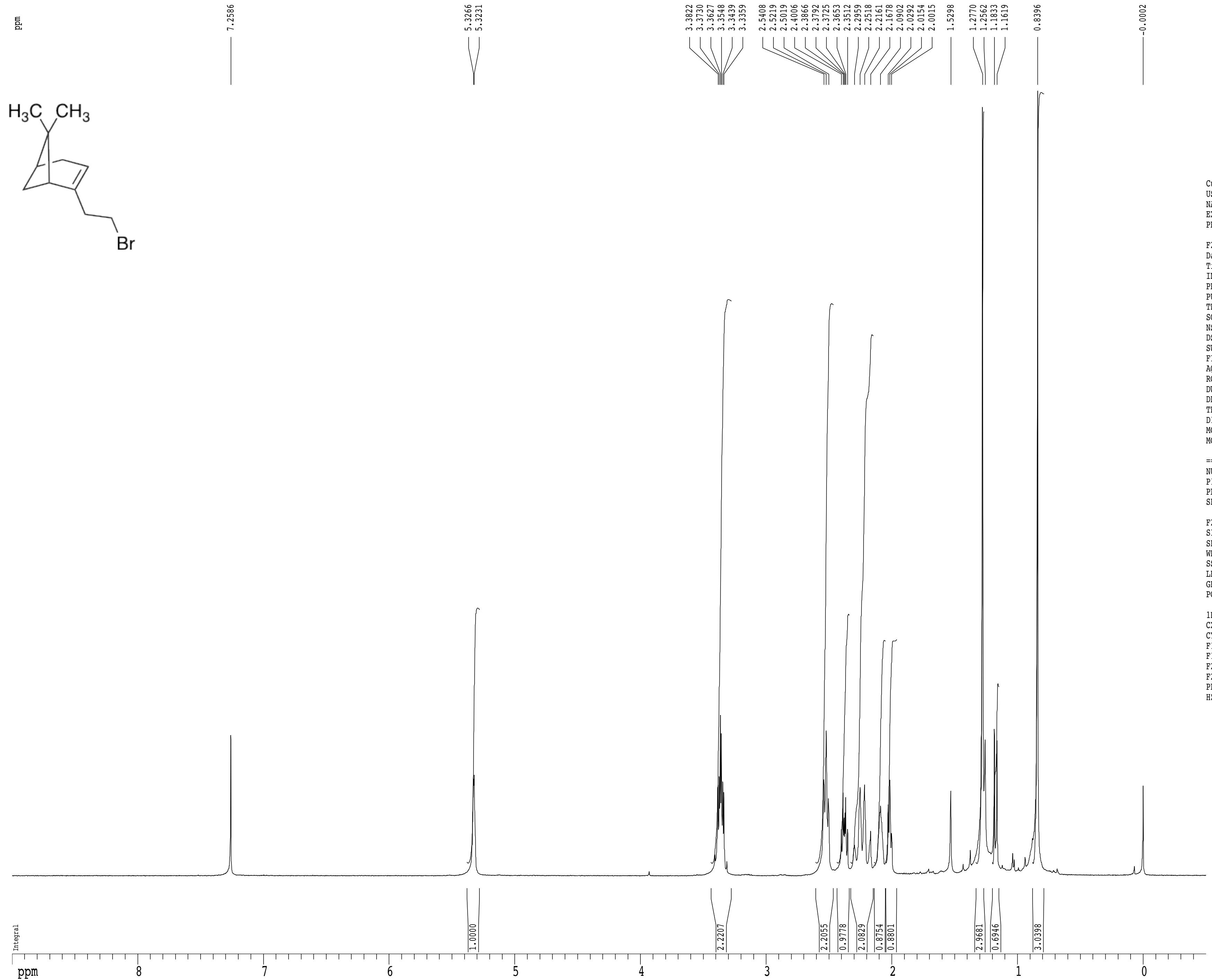

Current Data Parameters
USER linpc2
NAME pcl-3-017
EXPNO 2
PROCNO 1

F2 - Acquisition Parameters
Date_ 20220121
Time 9.12
INSTRUM drx400
PROBHD 5 mm QNP H/F/P
PULPROG zg30
TD 65536
SOLVENT CDCl3
NS 8
DS 2
SWH 6410.256 Hz
FIDRES 0.097813 Hz
AQ 5.1118579 sec
RG 287.4
DW 78.000 usec
DE 4.50 usec
TE 298.0 K
D1 0.10000000 sec
MCREST 0.00000000 sec
MCWRK 0.01500000 sec

======== CHANNEL f1 ========
NUC1 1H
P1 12.00 usec
PL1 -0.90 dB
SFO1 400.1328009 MHz

F2 - Processing parameters
SI 65536
SF 400.1300217 MHz
WDW EM
SSB 0
LB 0.30 Hz
GB 0
PC 2.00

1D NMR plot parameters
CX 22.80 cm
CY 15.00 cm
F1P 9.000 ppm
F1 3601.17 Hz
F2P -0.500 ppm
F2 -200.06 Hz
PPMCM 0.41667 ppm/cm
HZCM 166.72086 Hz/cm

1H spectrum

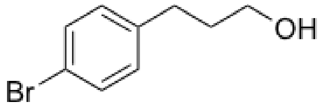

53

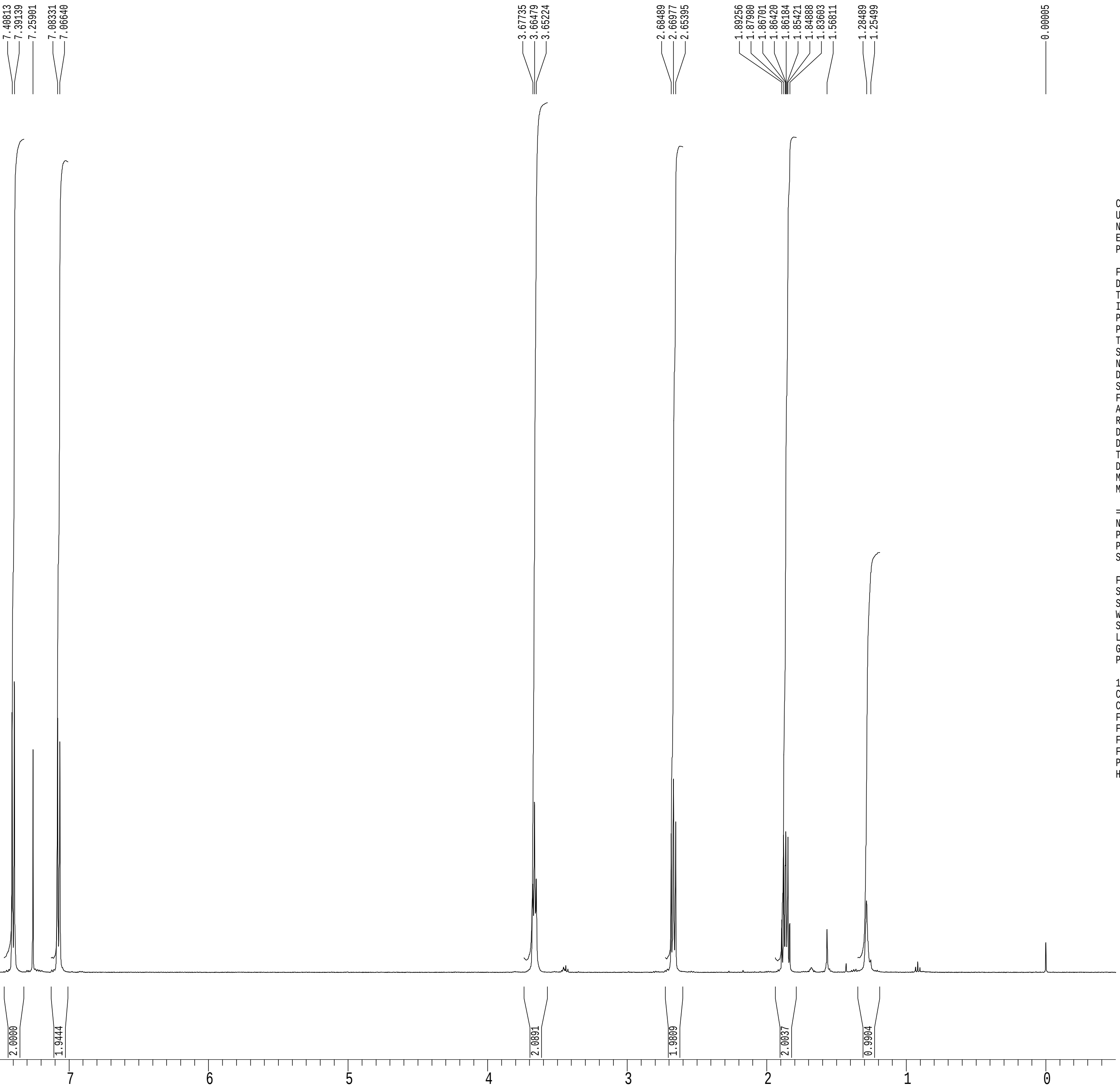


```
Current Data Parameters
USER          nhirbawi
NAME     NH-2-47-washed
EXPNO                 1
PROCNO                1

F2 - Acquisition Parameters
Date_          20211005
Time              15.00
INSTRUM           gn500
PROBHD   5 mm broadband
PULPROG            zg30
TD                81728
SOLVENT          CDCl3T
NS                    8
DS                    2
SWH            8012.820 Hz
FIDRES         0.098043 Hz
AQ            5.0998774 sec
RG               1290.2
DW               62.400 usec
DE                 6.00 usec
TE                298.0 K
D1           0.10000000 sec
MCREST       0.00000000 sec
MCWRK        0.01500000 sec

======== CHANNEL f1 ========
NUC1                 1H
P1                12.00 usec
PL1               -6.00 dB
SFO1        498.6534906 MHz

F2 - Processing parameters
SI                65536
SF          498.6500291 MHz
WDW                  EM
SSB                   0
LB                 0.30 Hz
GB                    0
PC                 1.00

1D NMR plot parameters
CX                22.80 cm
CY                 5.00 cm
F1P               9.000 ppm
F1              4487.85 Hz
F2P              -0.500 ppm
F2              -249.32 Hz
PPMCM           0.41667 ppm/cm
HZCM          207.77084 Hz/cm
```

1H spectrum

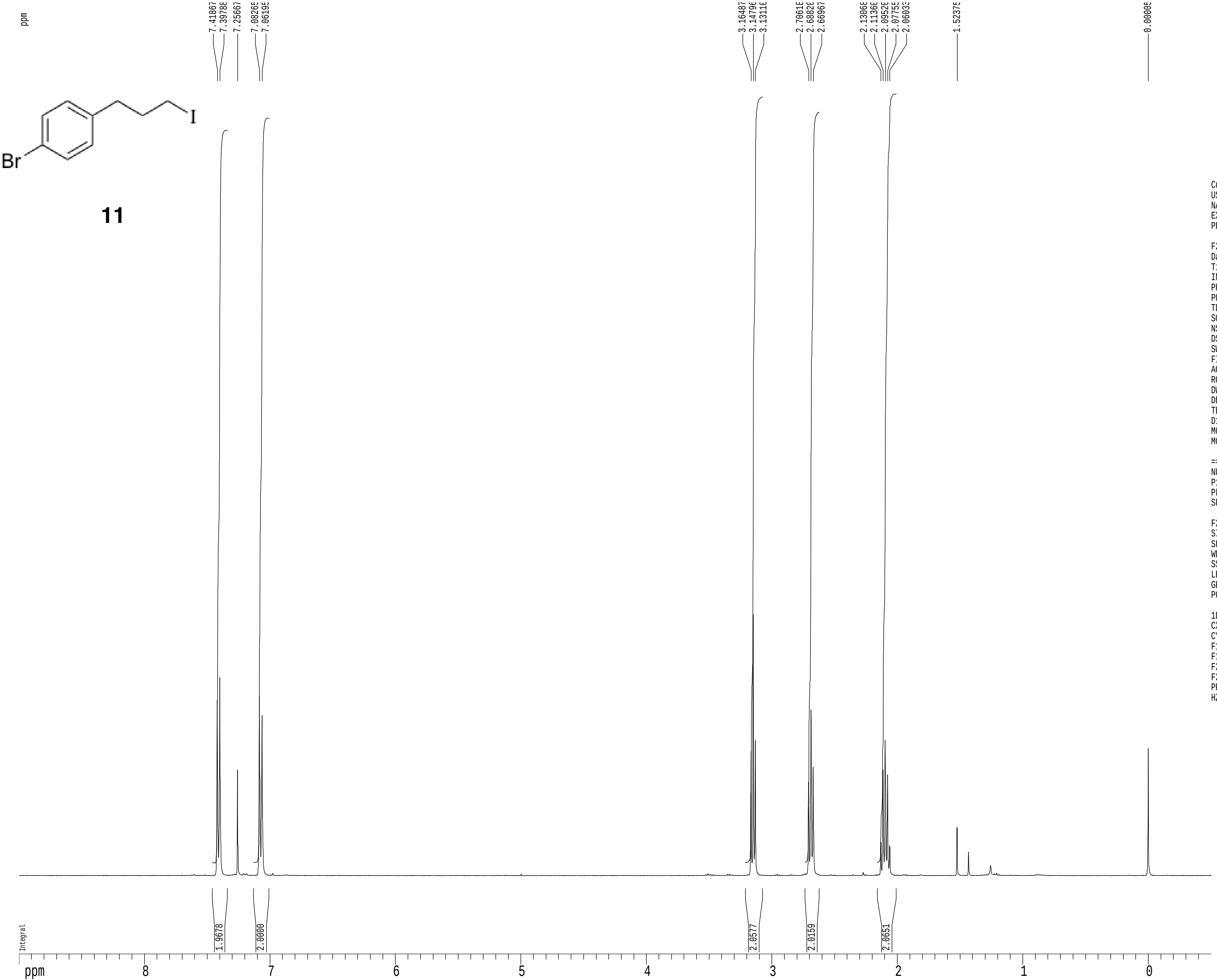

11

```
Current Data Parameters
USER          nhirbawi
NAME     NH-2-80-column
EXPNO                 1
PROCNO                1

F2 - Acquisition Parameters
Date_          20211007
Time              18.29
INSTRUM          drx400
PROBHD   5 mm Multinucl
PULPROG            zg30
TD                65536
SOLVENT          CDCl3T
NS                    8
DS                    2
SWH            6410.256 Hz
FIDRES         0.097813 Hz
AQ            5.1118579 sec
RG                645.1
DW               78.000 usec
DE                 4.50 usec
TE                298.0 K
D1           0.10000000 sec
MCREST       0.00000000 sec
MCWRK        0.01500000 sec

======== CHANNEL f1 ========
NUC1                 1H
P1                12.00 usec
PL1               -1.10 dB
SFO1        400.1328009 MHz

F2 - Processing parameters
SI                65536
SF          400.1300228 MHz
WDW                  EM
SSB                   0
LB                 0.30 Hz
GB                    0
PC                 2.00

1D NMR plot parameters
CX                22.80 cm
CY                 5.00 cm
F1P               9.000 ppm
F1              3601.17 Hz
F2P              -0.500 ppm
F2              -200.06 Hz
PPMCM           0.41667 ppm/cm
HZCM           166.72086 Hz/cm
```

Z-restored spin-echo 13C spectrum with 1H decoupling

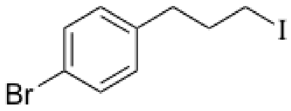

11

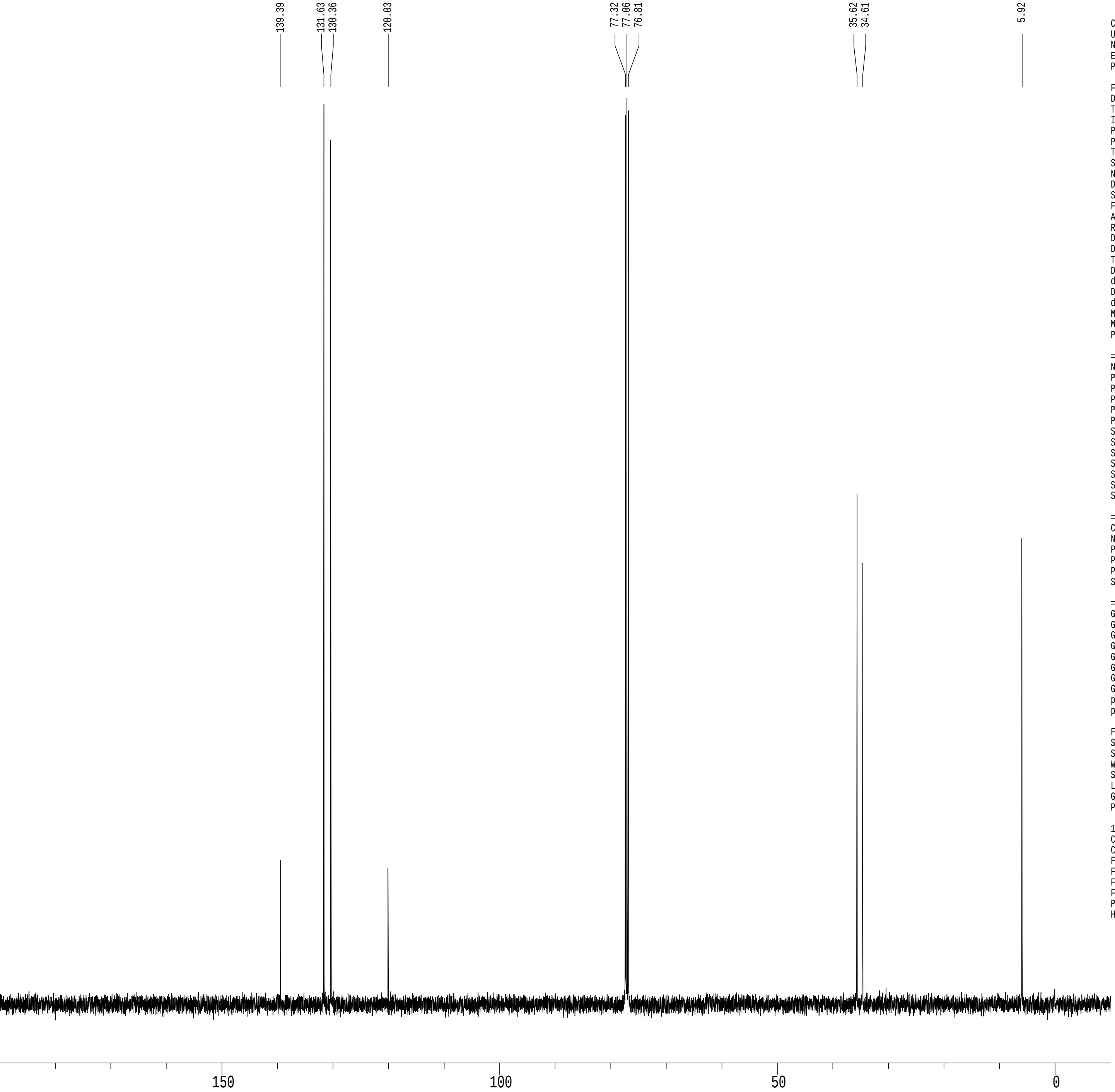


```
Current Data Parameters
USER          nhirbawi
NAME     NH-2-80-carbon
EXPNO                1
PROCNO               1

F2 - Acquisition Parameters
Date_         20211012
Time             17.06
INSTRUM        cryo500
PROBHD   5 mm CPTCI 1H-
PULPROG  SpinEchopg30gp2.prd
TD               65536
SOLVENT          CDCl3T
NS                 341
DS                  16
SWH          30303.031 Hz
FIDRES        0.462388 Hz
AQ           1.0813940 sec
RG              4597.6
DW              16.500 usec
DE                6.00 usec
TE               298.0 K
D1          0.25000000 sec
d11         0.03000000 sec
D16         0.00020000 sec
d17         0.00019600 sec
MCREST      0.00000000 sec
MCWRK       0.01500000 sec
P2               37.70 usec

======== CHANNEL f1 ========
NUC1               13C
P1               18.85 usec
P12            2000.00 usec
P20             500.00 usec
PL0             120.00 dB
PL1              -1.00 dB
SFO1       125.7942548 MHz
SP2               1.55 dB
SP4               1.55 dB
SPNAM2     Crp60comp.4
SPNAM4   Crp60,0.5,20.1
SPOFF2            0.00 Hz
SPOFF4            0.00 Hz

======== CHANNEL f2 ========
CPDPRG2         waltz16
NUC2                 1H
PCPD2           100.00 usec
PL2               1.60 dB
PL12             22.00 dB
SFO2       500.2225011 MHz

====== GRADIENT CHANNEL =====
GPNAM1        SINE.100
GPNAM2        SINE.100
GPX1              0.00 %
GPX2              0.00 %
GPY1              0.00 %
GPY2              0.00 %
GPZ1             30.00 %
GPZ2             50.00 %
p15             500.00 usec
p16            1000.00 usec

F2 - Processing parameters
SI               65536
SF         125.7804190 MHz
WDW                 EM
SSB                  0
LB                1.00 Hz
GB                   0
PC                2.00

1D NMR plot parameters
CX               22.80 cm
CY               15.65 cm
F1P            230.000 ppm
F1            28929.50 Hz
F2P            -10.000 ppm
F2            -1257.80 Hz
PPMCM         10.52632 ppm/cm
HZCM        1324.00439 Hz/cm
```

1H spectrum

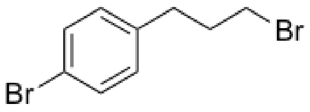

33

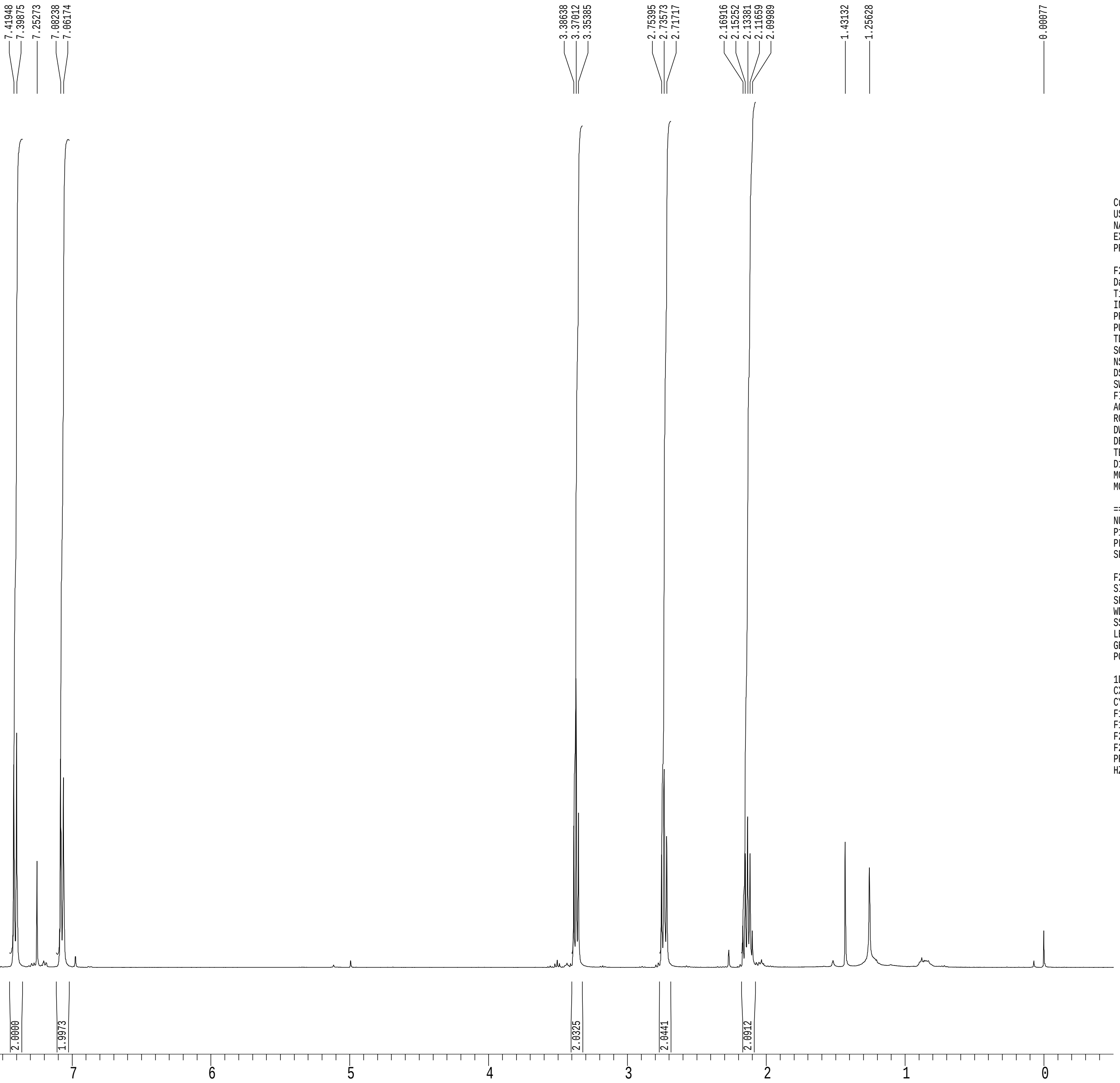


```
Current Data Parameters
USER          nhirbawi
NAME      NH-2-141-column
EXPNO                1
PROCNO               1

F2 - Acquisition Parameters
Date_          20220111
Time              14.24
INSTRUM          drx400
PROBHD   5 mm QNP H/F/P
PULPROG            zg30
TD                65536
SOLVENT          CDCl3T
NS                    8
DS                    2
SWH            6410.256 Hz
FIDRES         0.097813 Hz
AQ            5.1118579 sec
RG                203.2
DW               78.000 usec
DE                 4.50 usec
TE                298.0 K
D1           0.10000000 sec
MCREST       0.00000000 sec
MCWRK        0.01500000 sec

======== CHANNEL f1 ========
NUC1                 1H
P1                12.00 usec
PL1               -0.90 dB
SFO1        400.1328009 MHz

F2 - Processing parameters
SI                65536
SF          400.1300243 MHz
WDW                  EM
SSB                   0
LB                 0.30 Hz
GB                    0
PC                 2.00

1D NMR plot parameters
CX                22.80 cm
CY                 5.00 cm
F1P               9.000 ppm
F1              3601.17 Hz
F2P              -0.500 ppm
F2              -200.06 Hz
PPMCM           0.41667 ppm/cm
HZCM          166.72086 Hz/cm
```

Z-restored spin-echo 13C spectrum with 1H decoupling

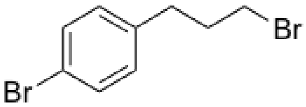

33

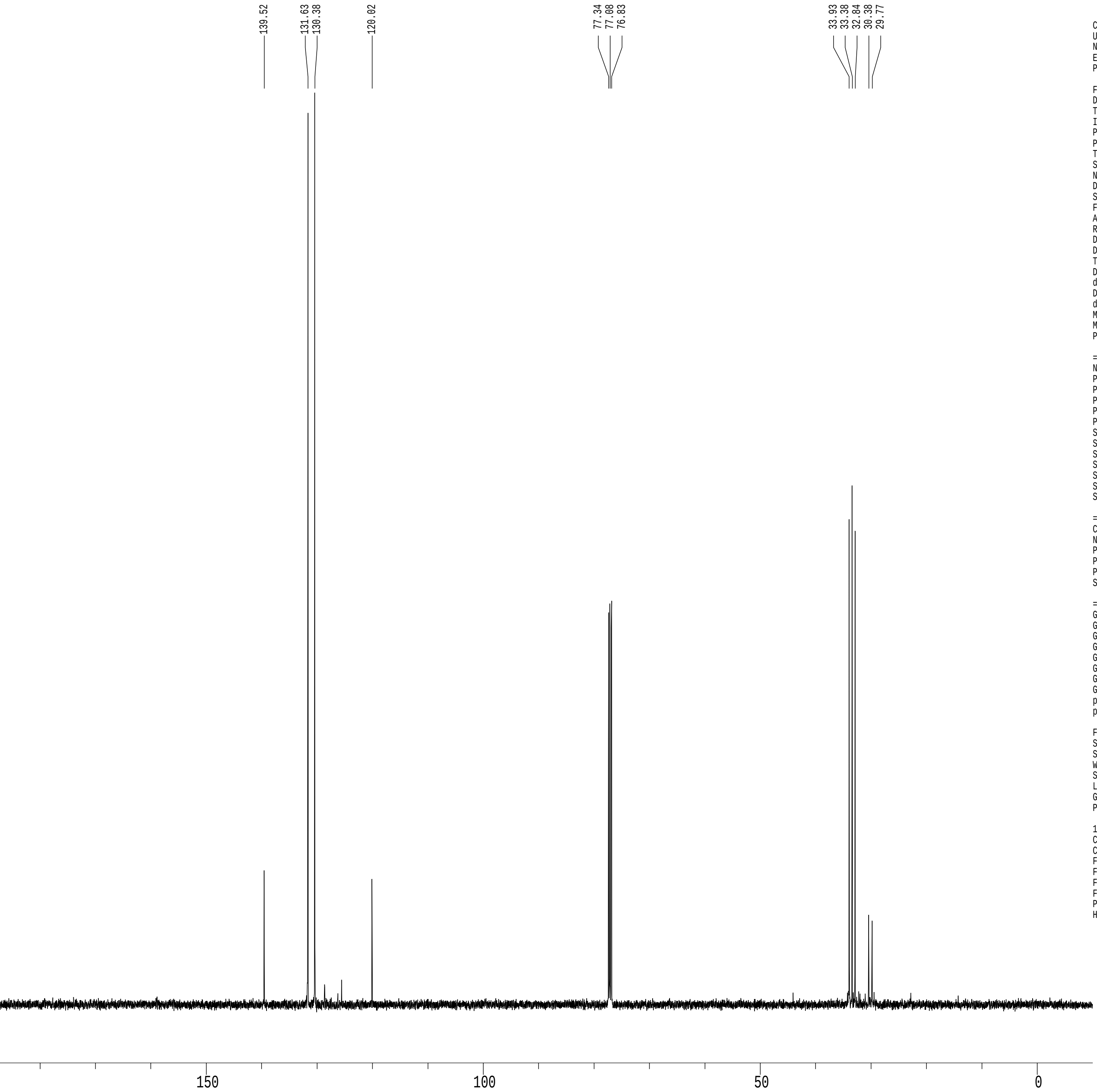


```
Current Data Parameters
USER          nhirbawi
NAME     NH-2-141-carbon
EXPNO                2
PROCNO               1

F2 - Acquisition Parameters
Date_         20220112
Time             11.42
INSTRUM        cryo500
PROBHD   5 mm CPTCI 1H-
PULPROG  SpinEchopg30gp2.prd
TD               65536
SOLVENT          CDCl3
NS                 405
DS                  16
SWH          30303.031 Hz
FIDRES        0.462388 Hz
AQ           1.0813940 sec
RG              2298.8
DW              16.500 usec
DE                6.00 usec
TE               298.0 K
D1          0.25000000 sec
d11         0.03000000 sec
D16         0.00020000 sec
d17         0.00019600 sec
MCREST      0.00000000 sec
MCWRK       0.01500000 sec
P2               37.70 usec

======== CHANNEL f1 ========
NUC1               13C
P1               18.85 usec
P12            2000.00 usec
P20             500.00 usec
PL0             120.00 dB
PL1              -1.00 dB
SFO1       125.7942548 MHz
SP2               1.55 dB
SP4               1.55 dB
SPNAM2     Crp60comp.4
SPNAM4   Crp60,0.5,20.1
SPOFF2            0.00 Hz
SPOFF4            0.00 Hz

======== CHANNEL f2 ========
CPDPRG2        waltz16
NUC2                1H
PCPD2           100.00 usec
PL2               1.60 dB
PL12             22.00 dB
SFO2       500.2225011 MHz

====== GRADIENT CHANNEL =====
GPNAM1        SINE.100
GPNAM2        SINE.100
GPX1              0.00 %
GPX2              0.00 %
GPY1              0.00 %
GPY2              0.00 %
GPZ1             30.00 %
GPZ2             50.00 %
p15             500.00 usec
p16            1000.00 usec

F2 - Processing parameters
SI               65536
SF         125.7804190 MHz
WDW                 EM
SSB                  0
LB                1.00 Hz
GB                   0
PC                2.00

1D NMR plot parameters
CX               22.80 cm
CY               15.65 cm
F1P            230.000 ppm
F1            28929.50 Hz
F2P            -10.000 ppm
F2            -1257.80 Hz
PPMCM         10.52632 ppm/cm
HZCM        1324.00439 Hz/cm
```

1H spectrum

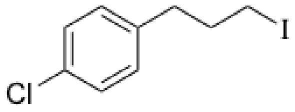

12

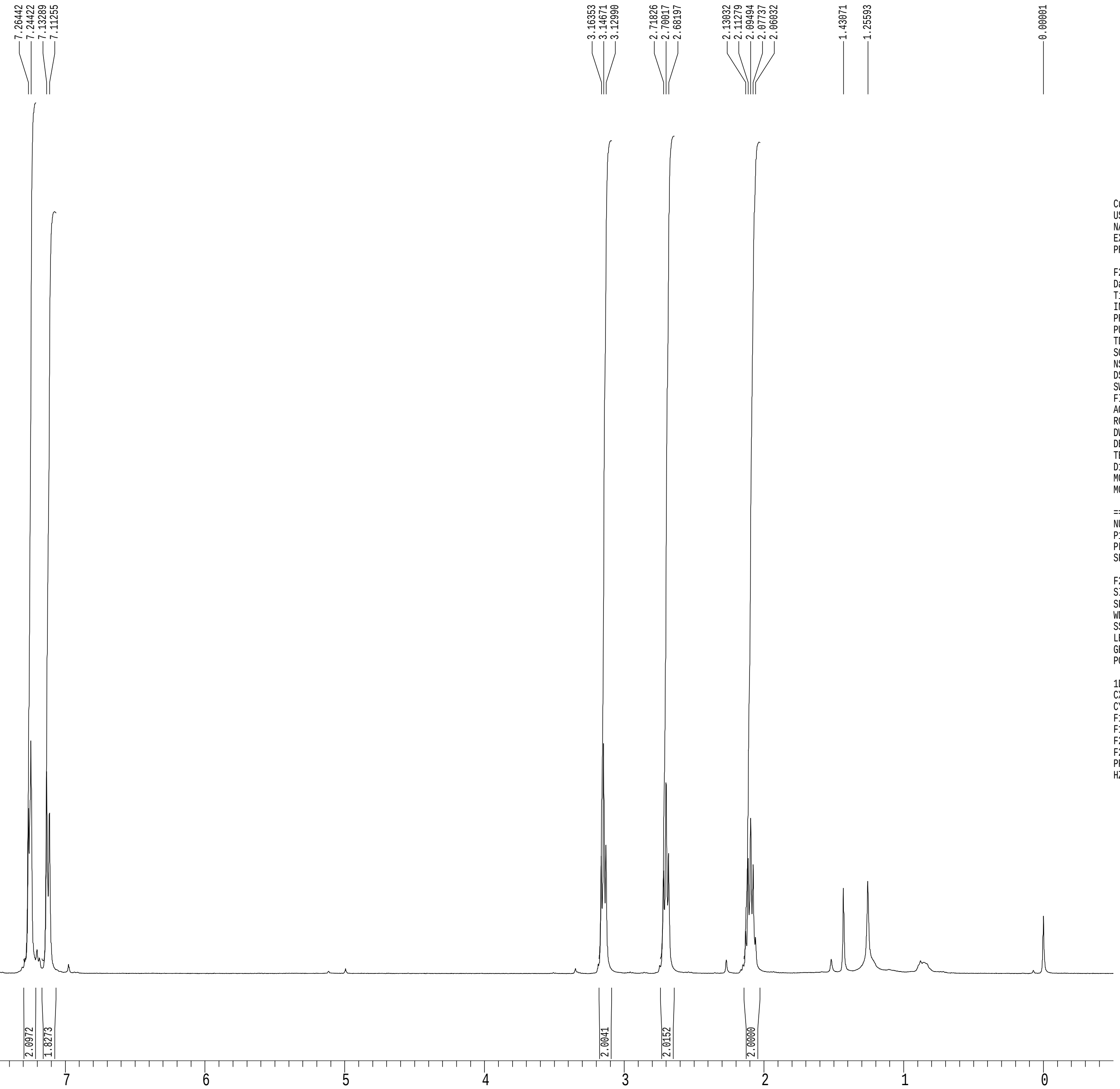


```
Current Data Parameters
USER            nhirbawi
NAME      NH-3-27-column
EXPNO                  1
PROCNO                 1

F2 - Acquisition Parameters
Date_           20220225
Time               11.19
INSTRUM           drx400
PROBHD   5 mm QNP H/F/P
PULPROG             zg30
TD                 65536
SOLVENT           CDCl3T
NS                     8
DS                     2
SWH             6410.256 Hz
FIDRES          0.097813 Hz
AQ             5.1118579 sec
RG                 228.1
DW                78.000 usec
DE                  4.50 usec
TE                 298.0 K
D1            0.10000000 sec
MCREST        0.00000000 sec
MCWRK         0.01500000 sec

======== CHANNEL f1 ========
NUC1                  1H
P1                 12.00 usec
PL1                -0.90 dB
SFO1         400.1328009 MHz

F2 - Processing parameters
SI                 65536
SF           400.1300245 MHz
WDW                   EM
SSB                    0
LB                  0.30 Hz
GB                     0
PC                  2.00

1D NMR plot parameters
CX                 22.80 cm
CY                  4.00 cm
F1P                9.000 ppm
F1               3601.17 Hz
F2P               -0.500 ppm
F2               -200.06 Hz
PPMCM            0.41667 ppm/cm
HZCM           166.72086 Hz/cm
```

1H spectrum

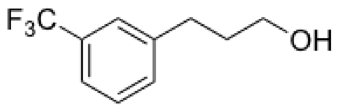

54

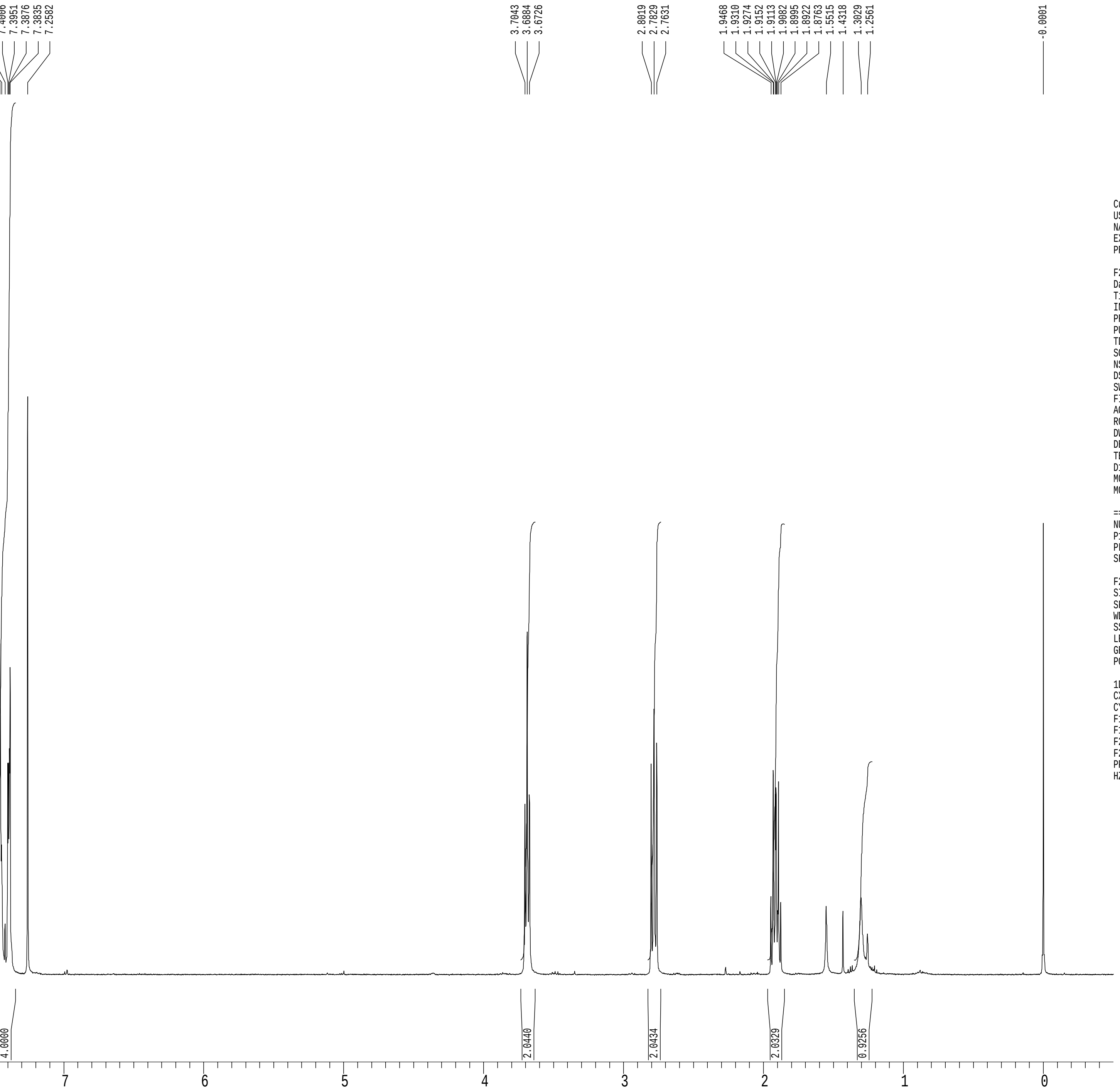


```
Current Data Parameters
USER          nhirbawi
NAME      NH-2-89-isco
EXPNO                1
PROCNO               1

F2 - Acquisition Parameters
Date_         20211026
Time             18.14
INSTRUM         drx400
PROBHD   5 mm QNP H/F/P
PULPROG           zg30
TD               65536
SOLVENT          CDCl3T
NS                   8
DS                   2
SWH           6410.256 Hz
FIDRES        0.097813 Hz
AQ           5.1118579 sec
RG               574.7
DW              78.000 usec
DE                4.50 usec
TE               298.0 K
D1          0.10000000 sec
MCREST      0.00000000 sec
MCWRK       0.01500000 sec

======== CHANNEL f1 ========
NUC1                1H
P1               12.00 usec
PL1              -0.90 dB
SFO1       400.1328009 MHz

F2 - Processing parameters
SI               65536
SF         400.1300221 MHz
WDW                 EM
SSB                  0
LB                0.30 Hz
GB                   0
PC                2.00

1D NMR plot parameters
CX               22.80 cm
CY               10.00 cm
F1P              9.000 ppm
F1             3601.17 Hz
F2P             -0.500 ppm
F2             -200.06 Hz
PPMCM          0.41667 ppm/cm
HZCM         166.72086 Hz/cm
```

13C spectrum with 1H decoupling

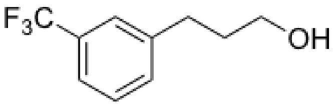

54

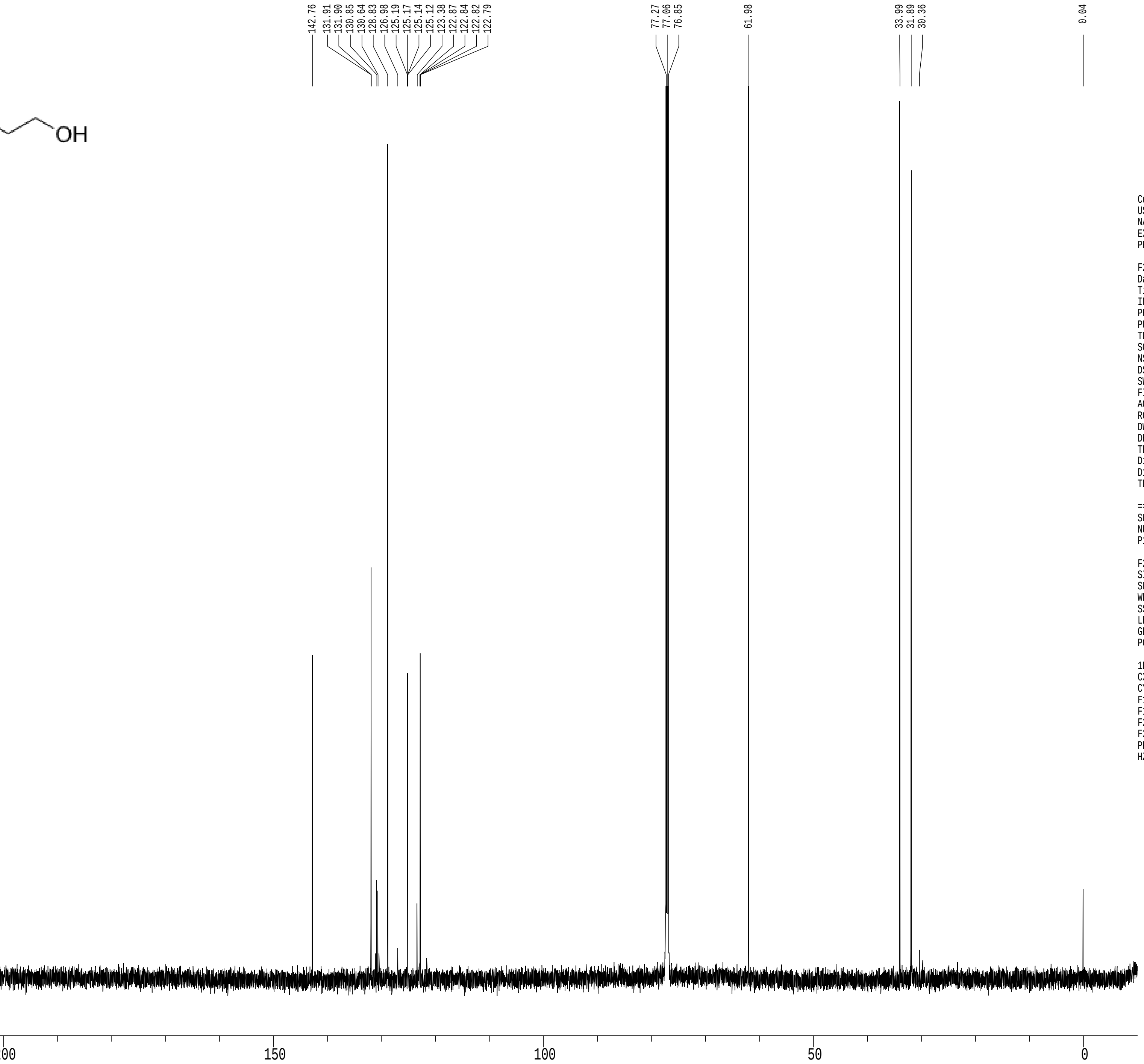

Current Data Parameters
USER nhirbawi
NAME NH-2-89-600-13C
EXPNO 1
PROCNO 1

F2 - Acquisition Parameters
Date_ 20211106
Time 16.48
INSTRUM av600
PROBHD 5 mm CPBBO BB-
PULPROG zgdc30
TD 65536
SOLVENT CDCl3T
NS 347
DS 4
SWH 36231.883 Hz
FIDRES 0.552855 Hz
AQ 0.9044468 sec
RG 2050
DW 13.800 usec
DE 19.63 usec
TE 298.0 K
D1 0.40000001 sec
D11 0.03000000 sec
TD0 1

======== CHANNEL f1 ========
SFO1 150.9194080 MHz
NUC1 13C
P1 10.10 usec

F2 - Processing parameters
SI 65536
SF 150.9028085 MHz
WDW EM
SSB 0
LB 1.00 Hz
GB 0
PC 1.00

1D NMR plot parameters
CX 22.80 cm
CY 100.00 cm
F1P 230.000 ppm
F1 34707.64 Hz
F2P -10.000 ppm
F2 -1509.03 Hz
PPMCM 10.52632 ppm/cm
HZCM 1588.45056 Hz/cm

19F spectrum

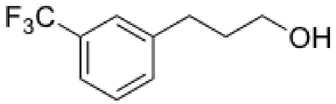

54

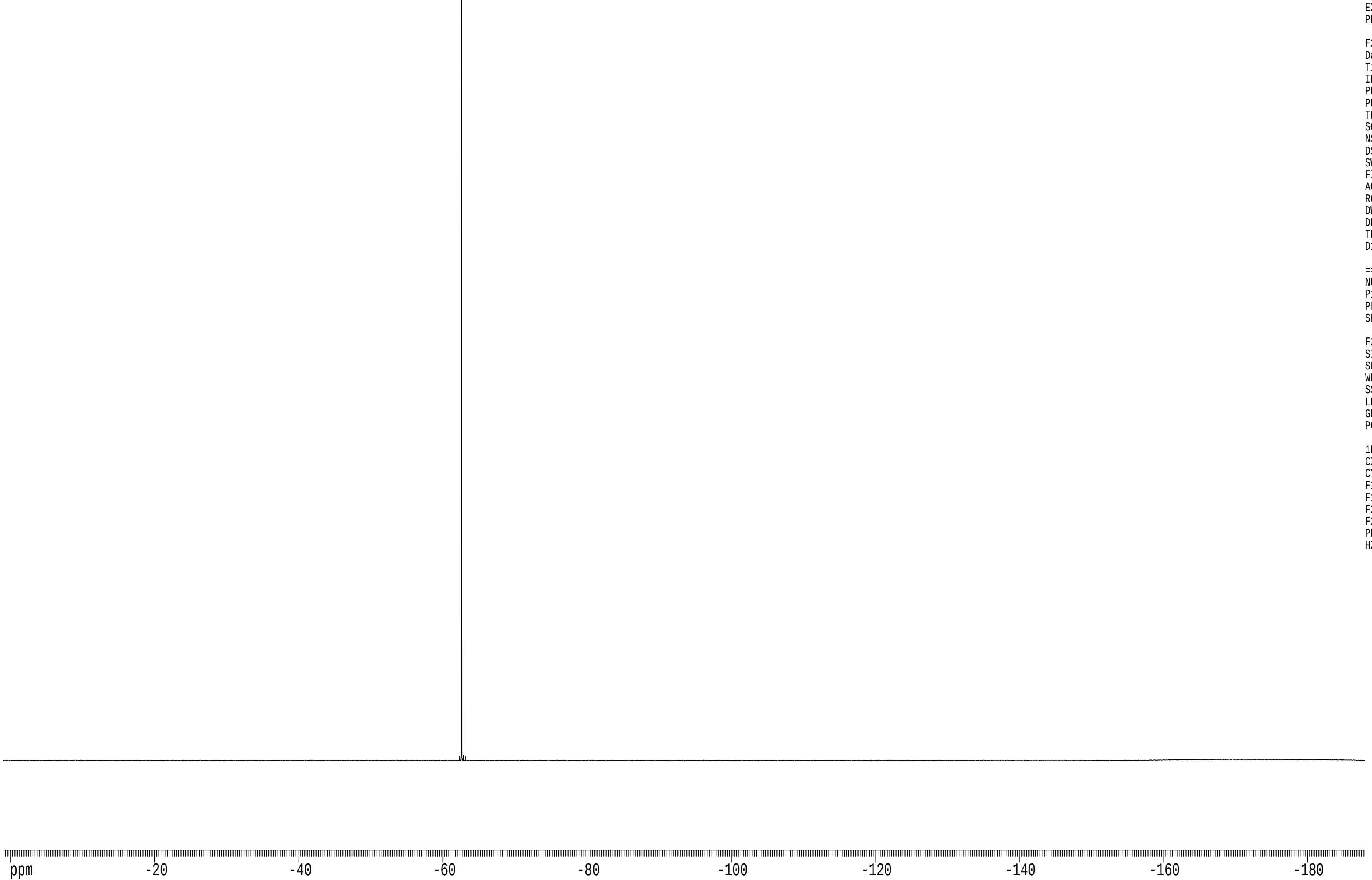


```
Current Data Parameters
USER          nhirbawi
NAME        NH-2-89-19F
EXPNO                2
PROCNO               1

F2 - Acquisition Parameters
Date_          20211106
Time              14.38
INSTRUM          drx400
PROBHD   5 mm QNP H/F/P
PULPROG        zgflqn30
TD                65536
SOLVENT           CDCl3
NS                   54
DS                    2
SWH           75187.969 Hz
FIDRES         1.147277 Hz
AQ            0.4358644 sec
RG               1149.4
DW                6.650 usec
DE                 9.46 usec
TE                298.1 K
D1           2.00000000 sec

======== CHANNEL f1 ========
NUC1                19F
P1                21.75 usec
PL1               -6.00 dB
SFO1        376.4646491 MHz

F2 - Processing parameters
SI                65536
SF          376.4983868 MHz
WDW                  EM
SSB                   0
LB                 1.00 Hz
GB                    0
PC                 1.00

1D NMR plot parameters
CX                22.80 cm
CY                15.00 cm
F1P               1.000 ppm
F1               376.50 Hz
F2P            -188.000 ppm
F2            -70781.70 Hz
PPMCM           8.28947 ppm/cm
HZCM         3120.97363 Hz/cm
```

1H spectrum

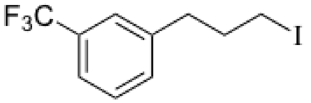

13

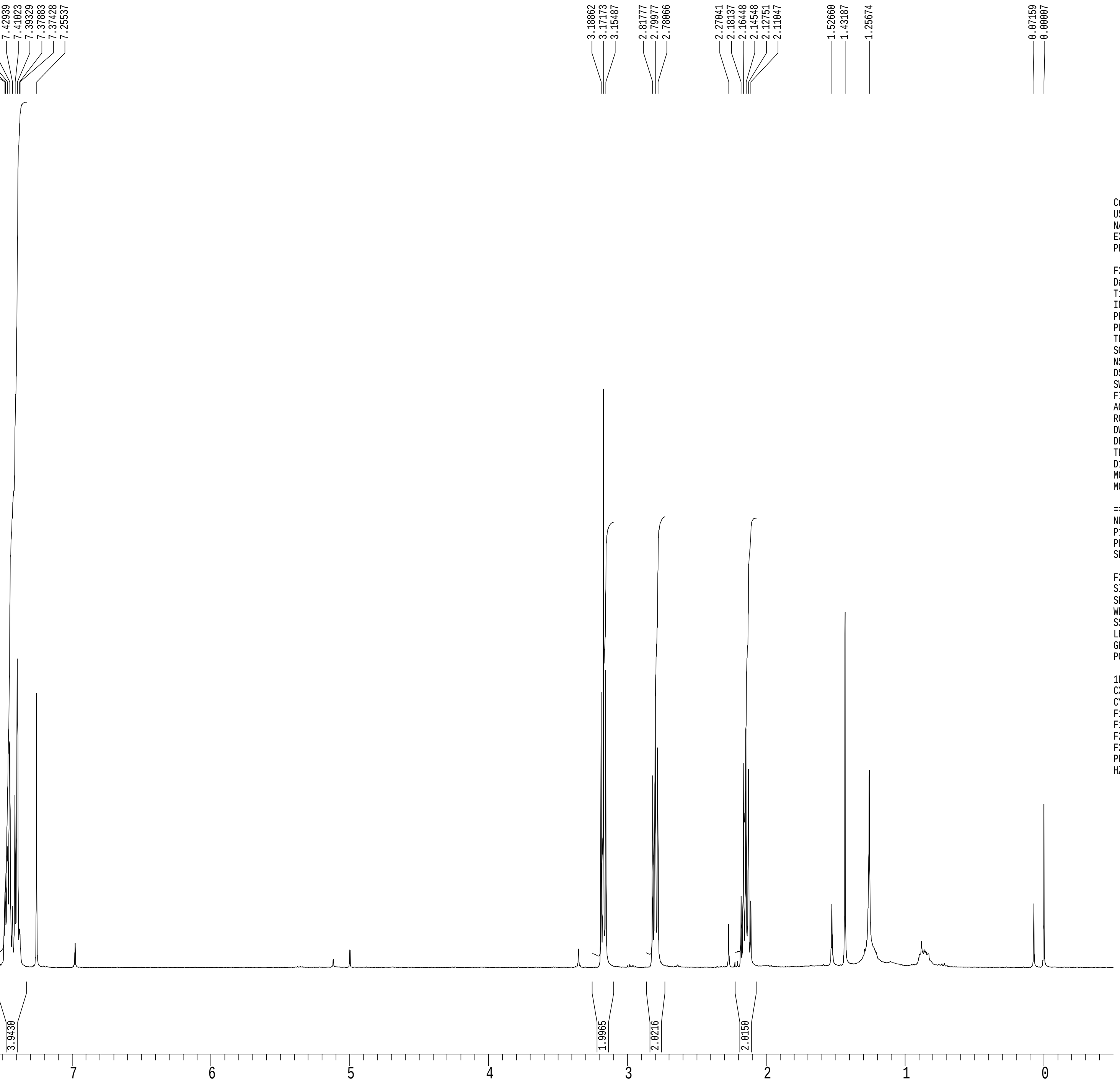

Current Data Parameters
USER nhirbawi
NAME NH-2-93-HandF
EXPNO 1
PROCNO 1

F2 - Acquisition Parameters
Date_ 20211106
Time 14.20
INSTRUM drx400
PROBHD 5 mm QNP H/F/P
PULPROG zg30
TD 65536
SOLVENT CDCl3T
NS 8
DS 2
SWH 6410.256 Hz
FIDRES 0.097813 Hz
AQ 5.1118579 sec
RG 256
DW 78.000 usec
DE 4.50 usec
TE 297.9 K
D1 0.10000000 sec
MCREST 0.00000000 sec
MCWRK 0.01500000 sec

======== CHANNEL f1 ========
NUC1 1H
P1 12.00 usec
PL1 -0.90 dB
SFO1 400.1328009 MHz

F2 - Processing parameters
SI 65536
SF 400.1300231 MHz
WDW EM
SSB 0
LB 0.30 Hz
GB 0
PC 2.00

1D NMR plot parameters
CX 22.80 cm
CY 10.00 cm
F1P 9.000 ppm
F1 3601.17 Hz
F2P -0.500 ppm
F2 -200.06 Hz
PPMCM 0.41667 ppm/cm
HZCM 166.72086 Hz/cm

13C spectrum with 1H decoupling

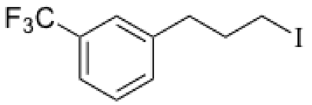

13

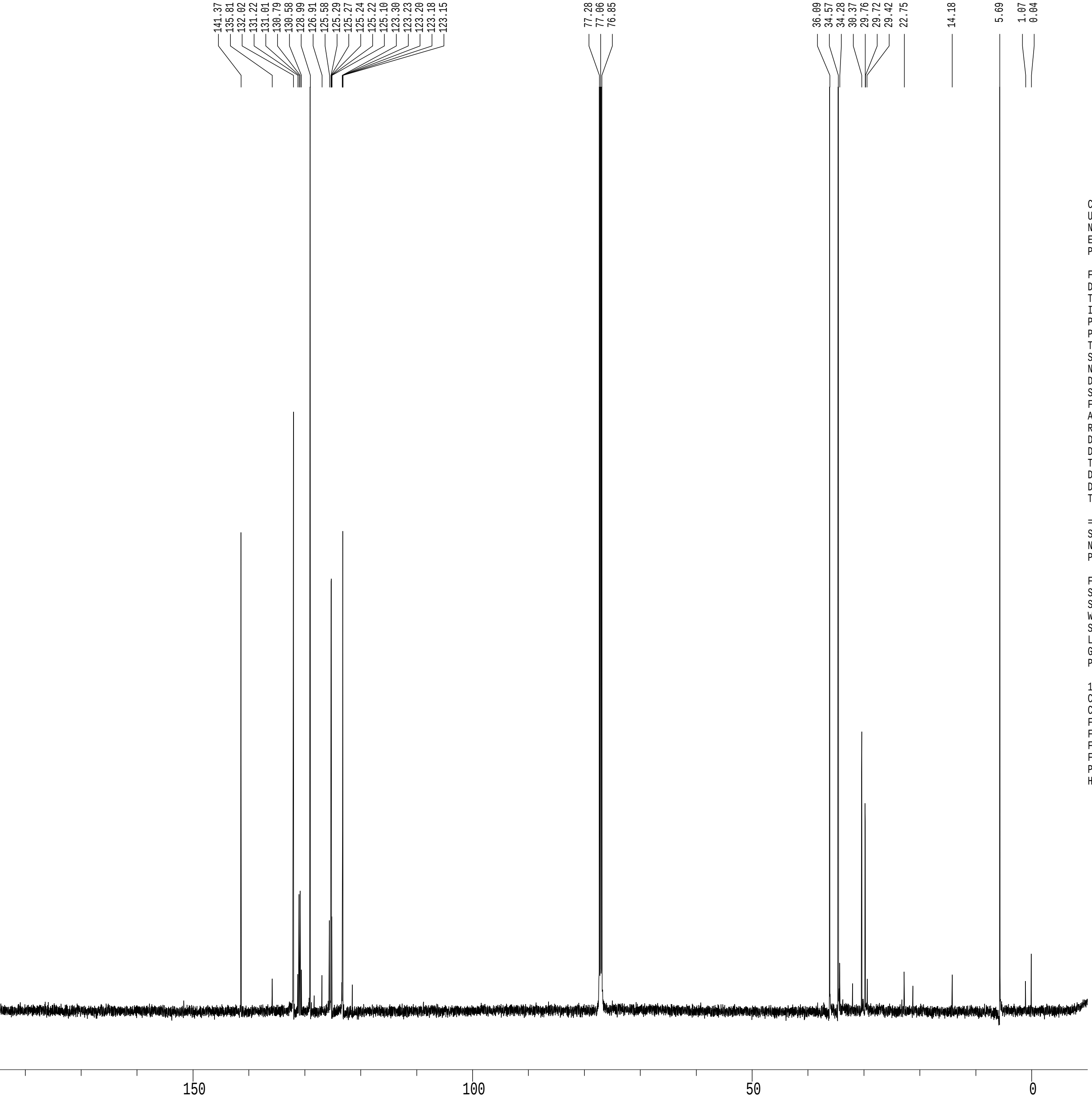

Current Data Parameters
USER nhirbawi
NAME NH-2-93-carbon
EXPNO 1
PROCNO 1

F2 - Acquisition Parameters
Date_ 20211106
Time 16.16
INSTRUM av600
PROBHD 5 mm CPBBO BB-
PULPROG zgdc30
TD 65536
SOLVENT CDCl3T
NS 400
DS 4
SWH 36231.883 Hz
FIDRES 0.552855 Hz
AQ 0.9044468 sec
RG 2050
DW 13.800 usec
DE 19.63 usec
TE 298.0 K
D1 0.40000001 sec
D11 0.03000000 sec
TD0 1

======== CHANNEL f1 ========
SFO1 150.9194080 MHz
NUC1 13C
P1 10.10 usec

F2 - Processing parameters
SI 65536
SF 150.9028085 MHz
WDW EM
SSB 0
LB 1.00 Hz
GB 0
PC 1.00

1D NMR plot parameters
CX 22.80 cm
CY 50.00 cm
F1P 230.000 ppm
F1 34707.64 Hz
F2P -10.000 ppm
F2 -1509.03 Hz
PPMCM 10.52632 ppm/cm
HZCM 1588.45056 Hz/cm

19F spectrum

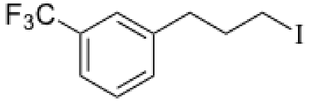

13

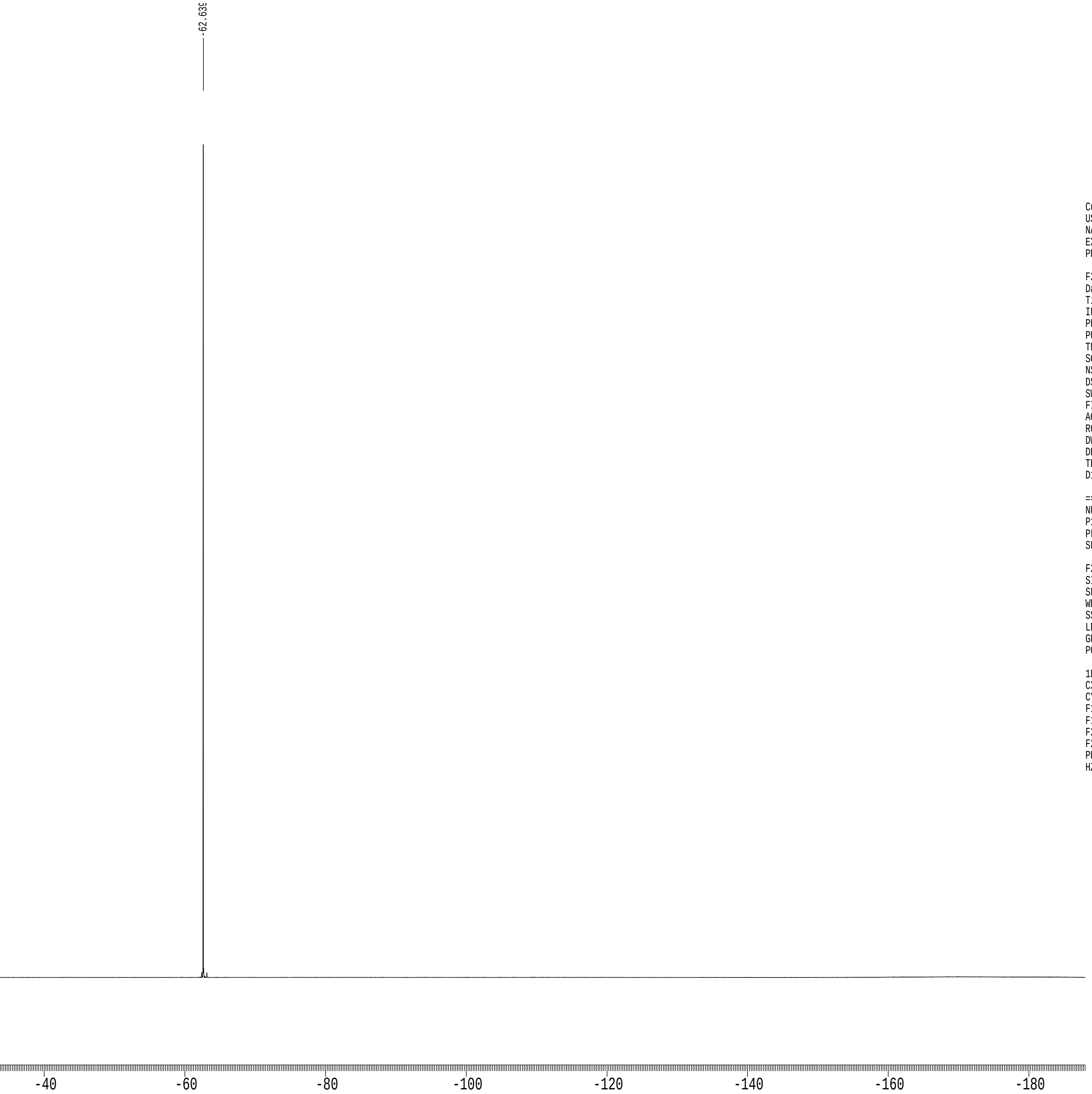


```
Current Data Parameters
USER          nhirbawi
NAME      NH-2-93-HandF
EXPNO                2
PROCNO               1

F2 - Acquisition Parameters
Date_         20211106
Time             14.23
INSTRUM         drx400
PROBHD   5 mm QNP H/F/P
PULPROG       zgflqn30
TD               65536
SOLVENT          CDCl3
NS                  51
DS                   2
SWH          75187.969 Hz
FIDRES        1.147277 Hz
AQ           0.4358644 sec
RG                1024
DW               6.650 usec
DE                9.46 usec
TE               298.0 K
D1          2.00000000 sec

======== CHANNEL f1 ========
NUC1               19F
P1               21.75 usec
PL1              -6.00 dB
SFO1       376.4646491 MHz

F2 - Processing parameters
SI               65536
SF         376.4983880 MHz
WDW                 EM
SSB                  0
LB                1.00 Hz
GB                   0
PC                1.00

1D NMR plot parameters
CX               22.80 cm
CY               15.00 cm
F1P              1.000 ppm
F1              376.50 Hz
F2P           -188.000 ppm
F2           -70781.70 Hz
PPMCM          8.28947 ppm/cm
HZCM        3120.97363 Hz/cm
```

1H spectrum

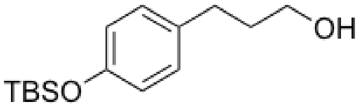

55

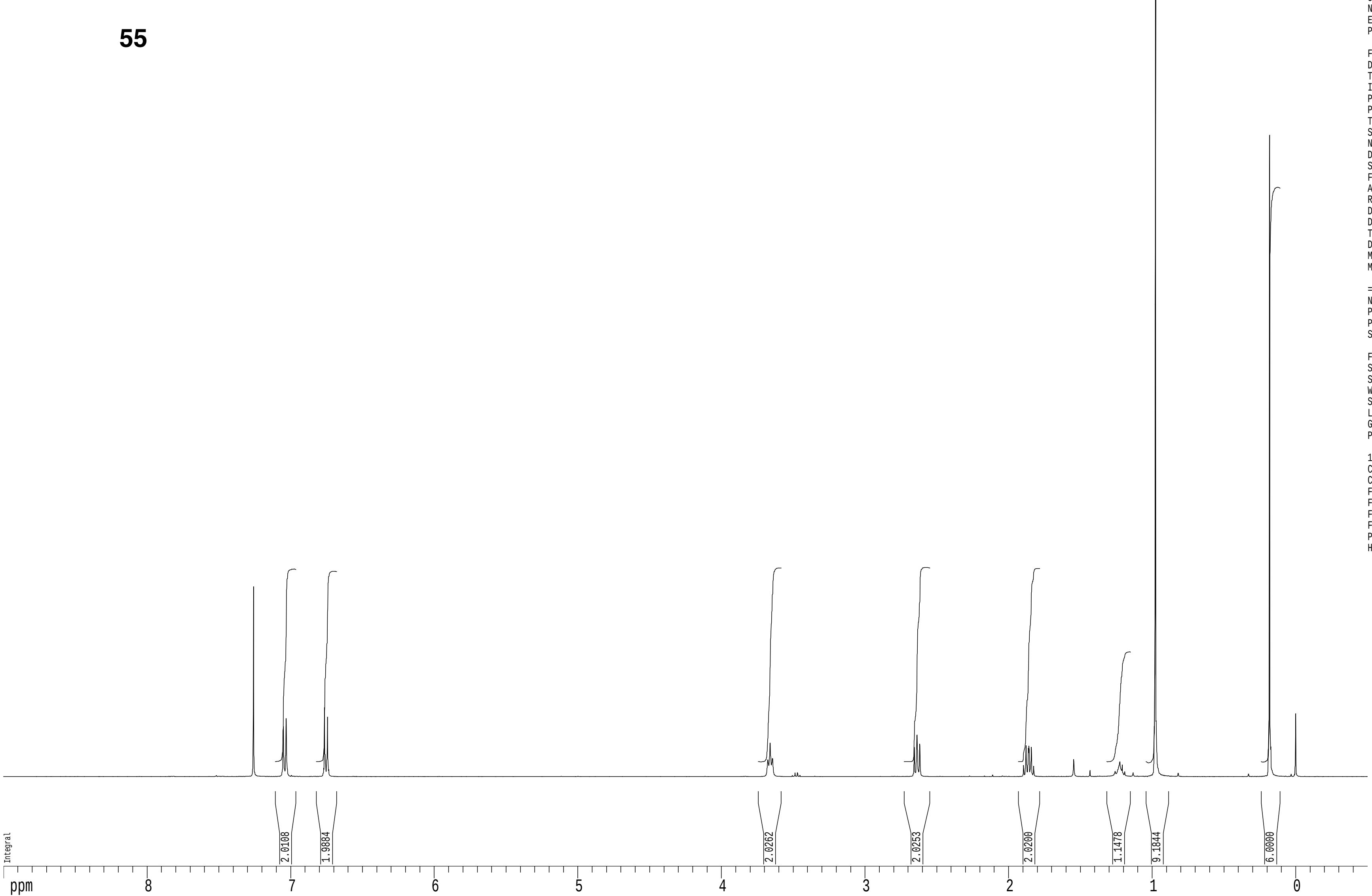

Current Data Parameters
USER nhirbawi
NAME NH-2-121-frac20-30
EXPNO 1
PROCNO 1

F2 - Acquisition Parameters
Date_ 20211207
Time 17.16
INSTRUM drx400
PROBHD 5 mm QNP H/F/P
PULPROG zg30
TD 65536
SOLVENT CDCl3T
NS 8
DS 2
SWH 6410.256 Hz
FIDRES 0.097813 Hz
AQ 5.1118579 sec
RG 362
DW 78.000 usec
DE 4.50 usec
TE 298.0 K
D1 0.10000000 sec
MCREST 0.00000000 sec
MCWRK 0.01500000 sec

======== CHANNEL f1 ========
NUC1 1H
P1 12.00 usec
PL1 -0.90 dB
SFO1 400.1328009 MHz

F2 - Processing parameters
SI 65536
SF 400.1300225 MHz
WDW EM
SSB 0
LB 0.30 Hz
GB 0
PC 2.00

1D NMR plot parameters
CX 22.80 cm
CY 15.00 cm
F1P 9.000 ppm
F1 3601.17 Hz
F2P -0.500 ppm
F2 -200.06 Hz
PPMCM 0.41667 ppm/cm
HZCM 166.72086 Hz/cm

1H spectrum

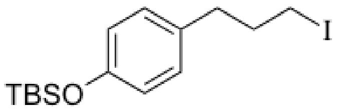

14

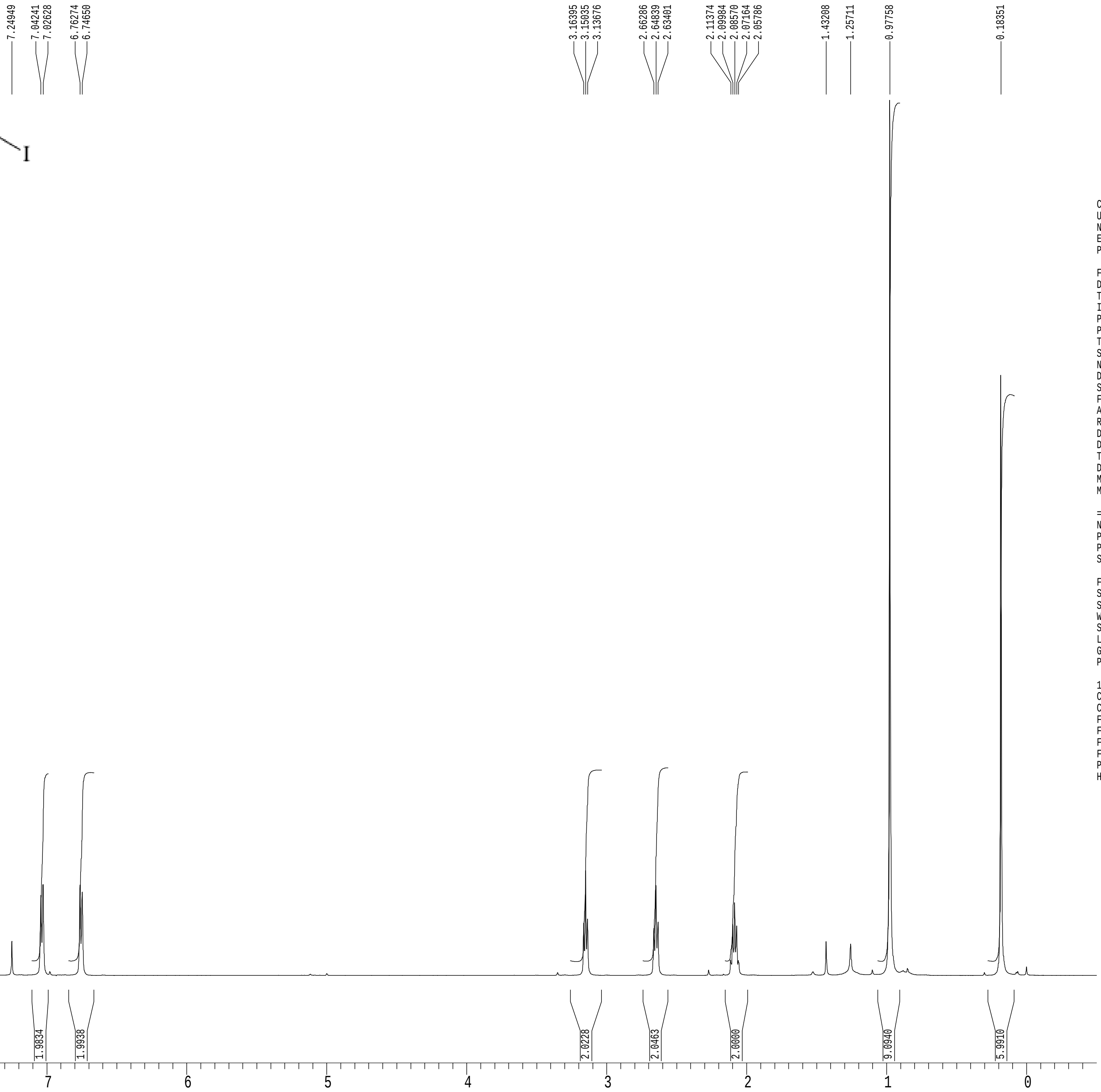

Current Data Parameters
USER nhirbawi
NAME NH-2-123-HandC
EXPNO 1
PROCNO 1

F2 - Acquisition Parameters
Date_ 20211211
Time 14.34
INSTRUM gn500
PROBHD 5 mm broadband
PULPROG zg30
TD 81728
SOLVENT CDCl3T
NS 8
DS 2
SWH 8012.820 Hz
FIDRES 0.098043 Hz
AQ 5.0998774 sec
RG 101.6
DW 62.400 usec
DE 6.00 usec
TE 298.0 K
D1 0.10000000 sec
MCREST 0.00000000 sec
MCWRK 0.01500000 sec

======== CHANNEL f1 ========
NUC1 1H
P1 12.00 usec
PL1 -6.00 dB
SFO1 498.6534906 MHz

F2 - Processing parameters
SI 65536
SF 498.6500338 MHz
WDW EM
SSB 0
LB 0.30 Hz
GB 0
PC 1.00

1D NMR plot parameters
CX 22.80 cm
CY 15.00 cm
F1P 9.000 ppm
F1 4487.85 Hz
F2P -0.500 ppm
F2 -249.32 Hz
PPMCM 0.41667 ppm/cm
HZCM 207.77084 Hz/cm

13C spectrum with 1H decoupling

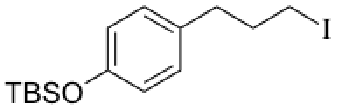

14

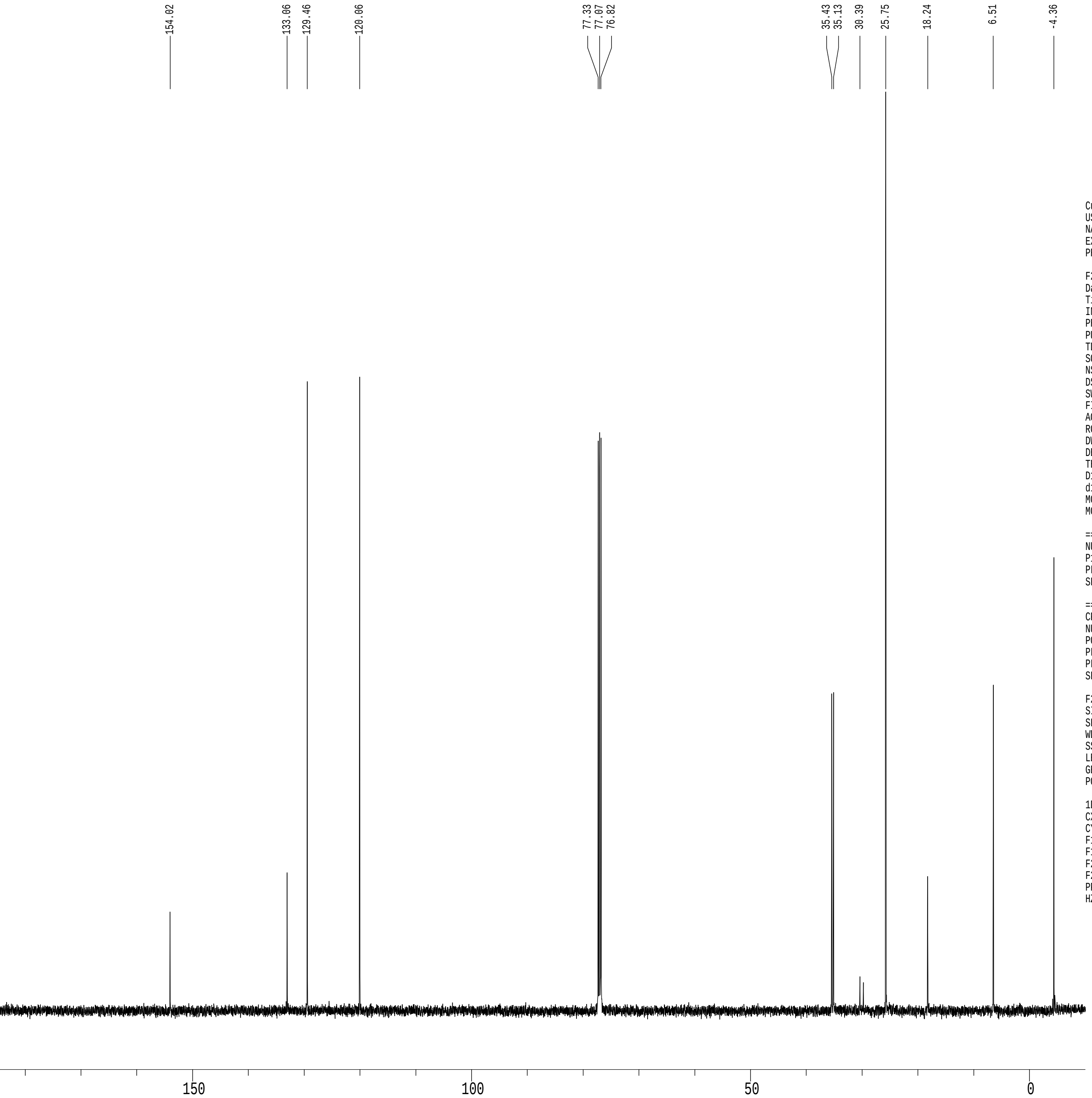


```
Current Data Parameters
USER          nhirbawi
NAME     NH-2-123-HandC
EXPNO                 2
PROCNO                1

F2 - Acquisition Parameters
Date_          20211211
Time              14.40
INSTRUM           gn500
PROBHD   5 mm broadband
PULPROG          zgdc30
TD                65536
SOLVENT           CDCl3
NS                  451
DS                    4
SWH          30303.031 Hz
FIDRES        0.462388 Hz
AQ           1.0813940 sec
RG                 6502
DW               16.500 usec
DE                 6.00 usec
TE                298.0 K
D1          0.25000000 sec
d11         0.03000000 sec
MCREST      0.00000000 sec
MCWRK       0.01500000 sec

======== CHANNEL f1 ========
NUC1                13C
P1                14.20 usec
PL1               -6.00 dB
SFO1        125.3994349 MHz

======== CHANNEL f2 ========
CPDPRG2         waltz16
NUC2                 1H
PCPD2            100.00 usec
PL2               -6.00 dB
PL12              12.30 dB
SFO2        498.6524933 MHz

F2 - Processing parameters
SI                65536
SF          125.3856420 MHz
WDW                  EM
SSB                   0
LB                 1.00 Hz
GB                    0
PC                 2.00

1D NMR plot parameters
CX                22.80 cm
CY                15.65 cm
F1P             230.000 ppm
F1             28838.70 Hz
F2P             -10.000 ppm
F2             -1253.86 Hz
PPMCM          10.52632 ppm/cm
HZCM         1319.84888 Hz/cm
```

1H spectrum

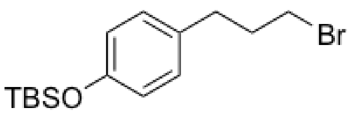

34

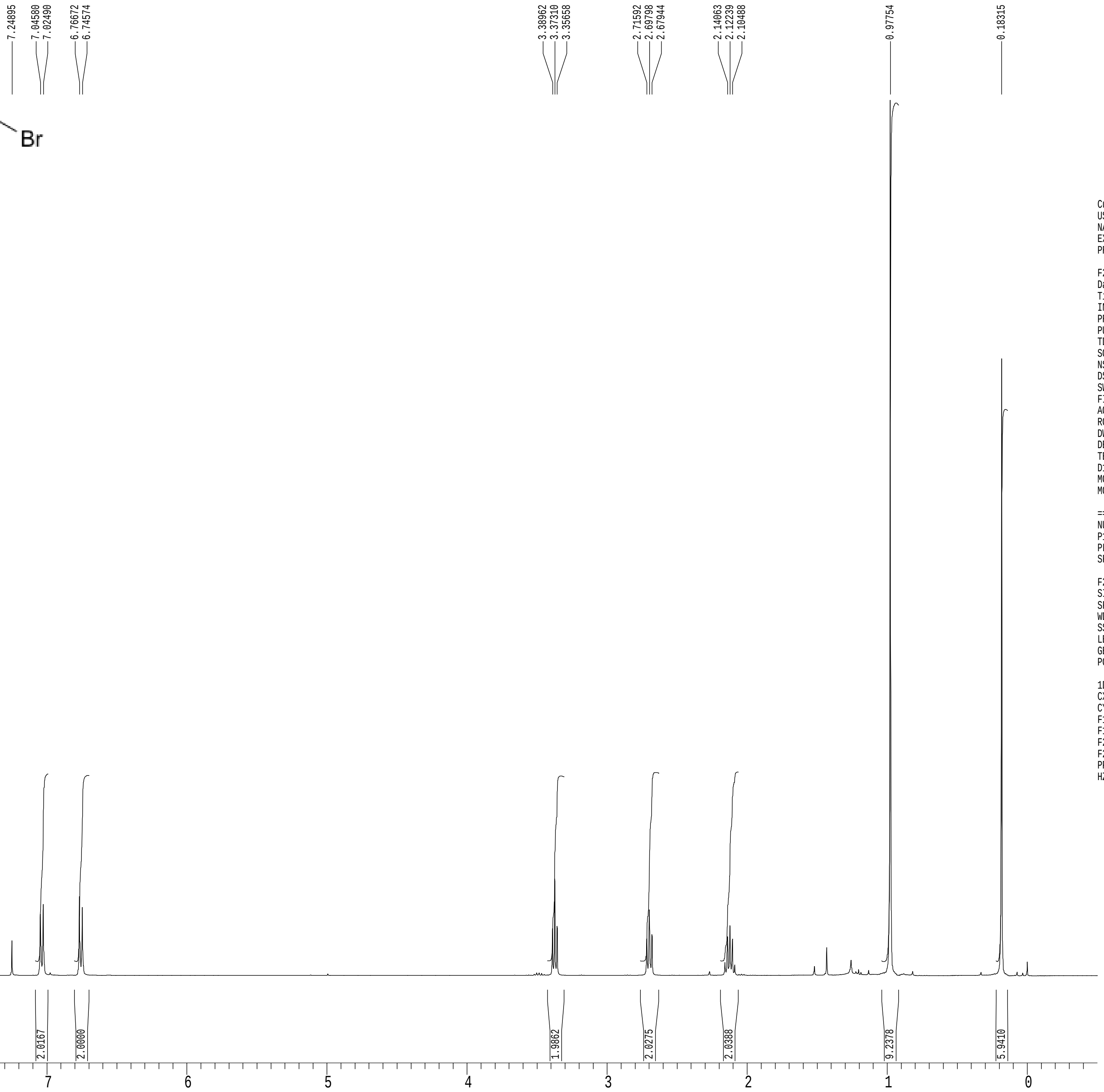

Current Data Parameters
USER nhirbawi
NAME NH-2-144-column
EXPNO 1
PROCNO 1

F2 - Acquisition Parameters
Date_ 20220107
Time 16.17
INSTRUM drx400
PROBHD 5 mm QNP H/F/P
PULPROG zg30
TD 65536
SOLVENT CDCl3T
NS 8
DS 2
SWH 6410.256 Hz
FIDRES 0.097813 Hz
AQ 5.1118579 sec
RG 114
DW 78.000 usec
DE 4.50 usec
TE 298.0 K
D1 0.10000000 sec
MCREST 0.00000000 sec
MCWRK 0.01500000 sec

======== CHANNEL f1 ========
NUC1 1H
P1 12.00 usec
PL1 -0.90 dB
SFO1 400.1328009 MHz

F2 - Processing parameters
SI 65536
SF 400.1300258 MHz
WDW EM
SSB 0
LB 0.30 Hz
GB 0
PC 2.00

1D NMR plot parameters
CX 22.80 cm
CY 15.00 cm
F1P 9.000 ppm
F1 3601.17 Hz
F2P -0.500 ppm
F2 -200.06 Hz
PPMCM 0.41667 ppm/cm
HZCM 166.72086 Hz/cm

Z-restored spin-echo 13C spectrum with 1H decoupling

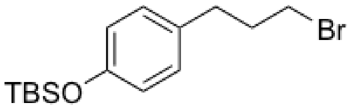

34

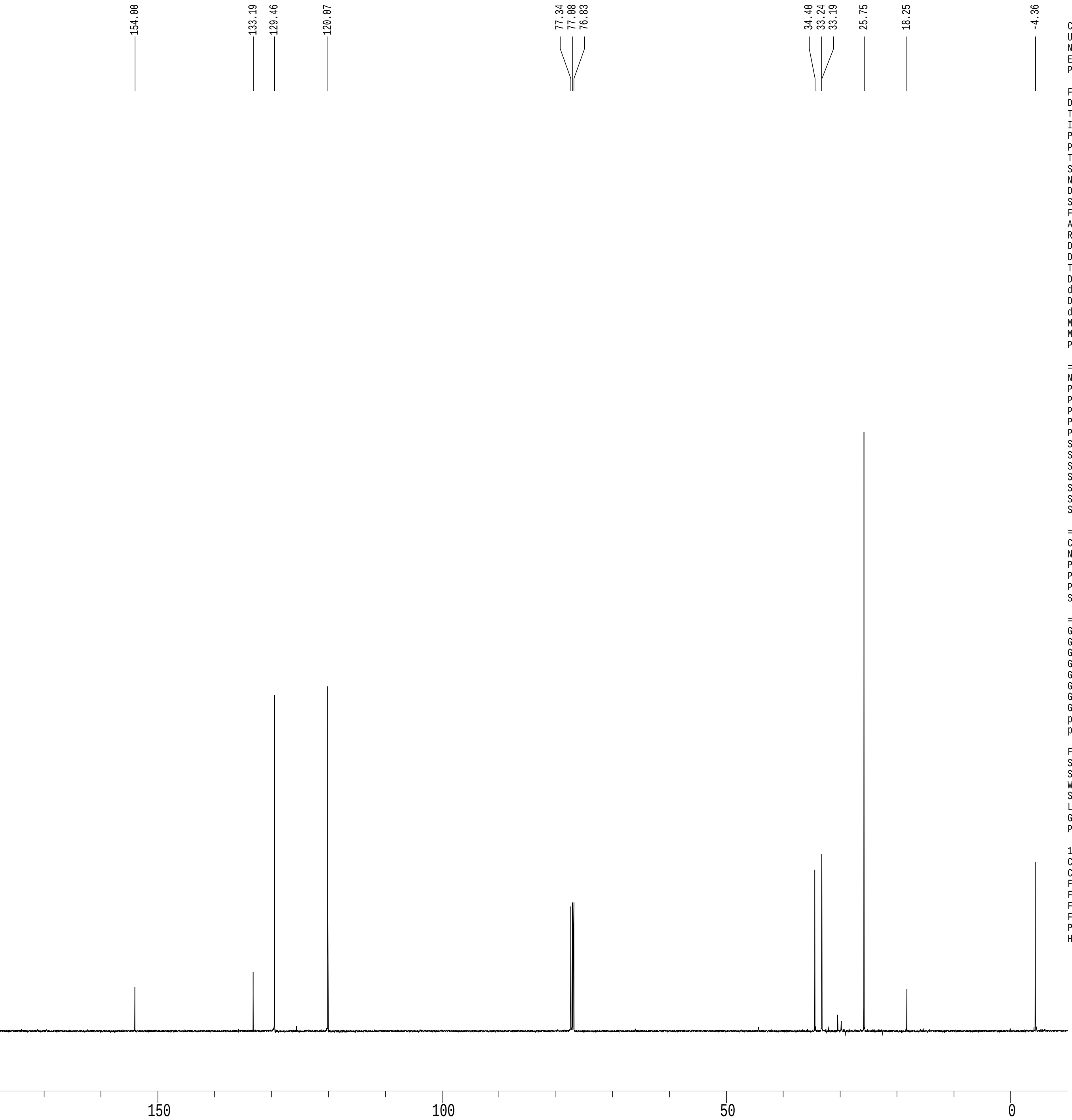


```
Current Data Parameters
USER           nhirbawi
NAME     NH-2-144-carbon
EXPNO                 2
PROCNO                1

F2 - Acquisition Parameters
Date_          20220112
Time              11.55
INSTRUM         cryo500
PROBHD   5 mm CPTCI 1H-
PULPROG  SpinEchopg30gp2.prd
TD                65536
SOLVENT           CDCl3
NS                  399
DS                   16
SWH           30303.031 Hz
FIDRES         0.462388 Hz
AQ            1.0813940 sec
RG               7298.2
DW               16.500 usec
DE                 6.00 usec
TE                298.0 K
D1           0.25000000 sec
d11          0.03000000 sec
D16          0.00020000 sec
d17          0.00019600 sec
MCREST       0.00000000 sec
MCWRK        0.01500000 sec
P2                37.70 usec

======== CHANNEL f1 ========
NUC1                13C
P1                18.85 usec
P12             2000.00 usec
P20              500.00 usec
PL0              120.00 dB
PL1               -1.00 dB
SFO1        125.7942548 MHz
SP2                1.55 dB
SP4                1.55 dB
SPNAM2      Crp60comp.4
SPNAM4   Crp60,0.5,20.1
SPOFF2             0.00 Hz
SPOFF4             0.00 Hz

======== CHANNEL f2 ========
CPDPRG2         waltz16
NUC2                 1H
PCPD2            100.00 usec
PL2                1.60 dB
PL12              22.00 dB
SFO2        500.2225011 MHz

====== GRADIENT CHANNEL =====
GPNAM1         SINE.100
GPNAM2         SINE.100
GPX1               0.00 %
GPX2               0.00 %
GPY1               0.00 %
GPY2               0.00 %
GPZ1              30.00 %
GPZ2              50.00 %
p15              500.00 usec
p16             1000.00 usec

F2 - Processing parameters
SI                65536
SF          125.7804190 MHz
WDW                  EM
SSB                   0
LB                 1.00 Hz
GB                    0
PC                 2.00

1D NMR plot parameters
CX                22.80 cm
CY                10.00 cm
F1P             230.000 ppm
F1             28929.50 Hz
F2P             -10.000 ppm
F2             -1257.80 Hz
PPMCM          10.52632 ppm/cm
HZCM         1324.00439 Hz/cm
```

1H spectrum

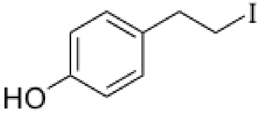

15

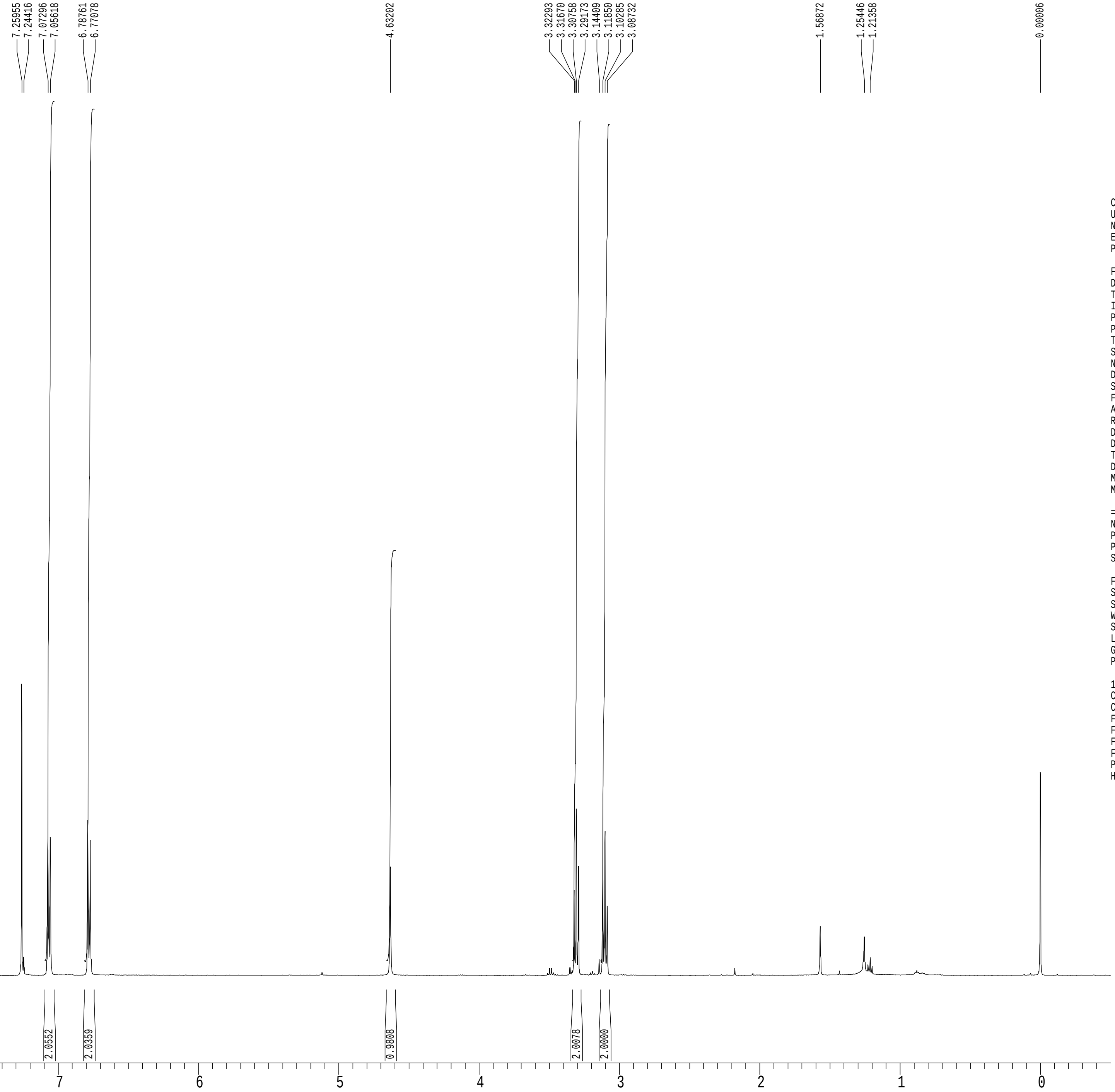


```
Current Data Parameters
USER          nhirbawi
NAME         NH-3-18-1H
EXPNO                 1
PROCNO                1

F2 - Acquisition Parameters
Date_          20220509
Time              16.06
INSTRUM         cryo500
PROBHD   5 mm CPTCI 1H-
PULPROG            zg30
TD                81728
SOLVENT          CDCl3T
NS                    8
DS                    2
SWH            8012.820 Hz
FIDRES         0.098043 Hz
AQ            5.0998774 sec
RG                  6.3
DW               62.400 usec
DE                 6.00 usec
TE                298.0 K
D1           0.10000000 sec
MCREST       0.00000000 sec
MCWRK        0.01500000 sec

======== CHANNEL f1 ========
NUC1                 1H
P1                 9.75 usec
PL1                1.60 dB
SFO1        500.2235015 MHz

F2 - Processing parameters
SI                65536
SF          500.2200325 MHz
WDW                  EM
SSB                   0
LB                 0.30 Hz
GB                    0
PC                 1.00

1D NMR plot parameters
CX                22.80 cm
CY                 5.00 cm
F1P               9.000 ppm
F1              4501.98 Hz
F2P              -0.500 ppm
F2              -250.11 Hz
PPMCM           0.41667 ppm/cm
HZCM          208.42502 Hz/cm
```

1H spectrum

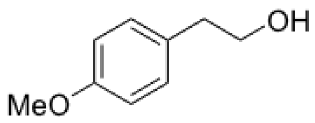

56

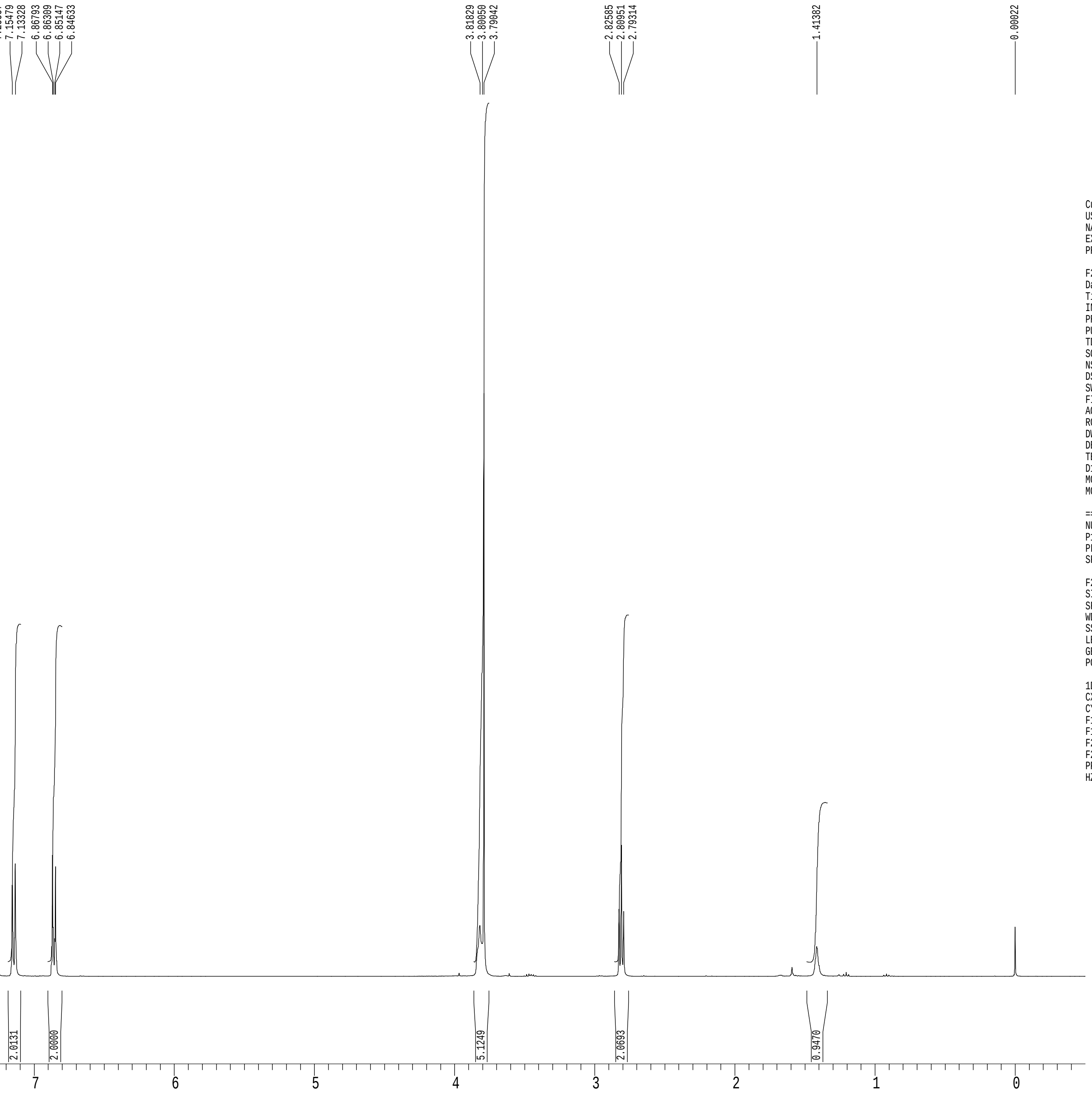


```
Current Data Parameters
USER            nhirbawi
NAME       NH-CAH-1-291-RSM
EXPNO                  1
PROCNO                 1

F2 - Acquisition Parameters
Date_           20211106
Time               14.13
INSTRUM           drx400
PROBHD    5 mm QNP H/F/P
PULPROG             zg30
TD                 65536
SOLVENT           CDCl3T
NS                     8
DS                     2
SWH             6410.256 Hz
FIDRES          0.097813 Hz
AQ             5.1118579 sec
RG                 287.4
DW                78.000 usec
DE                  4.50 usec
TE                 298.1 K
D1            0.10000000 sec
MCREST        0.00000000 sec
MCWRK         0.01500000 sec

======== CHANNEL f1 ========
NUC1                  1H
P1                 12.00 usec
PL1                -0.90 dB
SFO1         400.1328009 MHz

F2 - Processing parameters
SI                 65536
SF           400.1300233 MHz
WDW                   EM
SSB                    0
LB                  0.30 Hz
GB                     0
PC                  2.00

1D NMR plot parameters
CX                 22.80 cm
CY                 10.00 cm
F1P                9.000 ppm
F1               3601.17 Hz
F2P               -0.500 ppm
F2               -200.06 Hz
PPMCM            0.41667 ppm/cm
HZCM           166.72086 Hz/cm
```

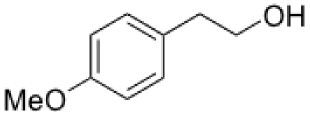

56

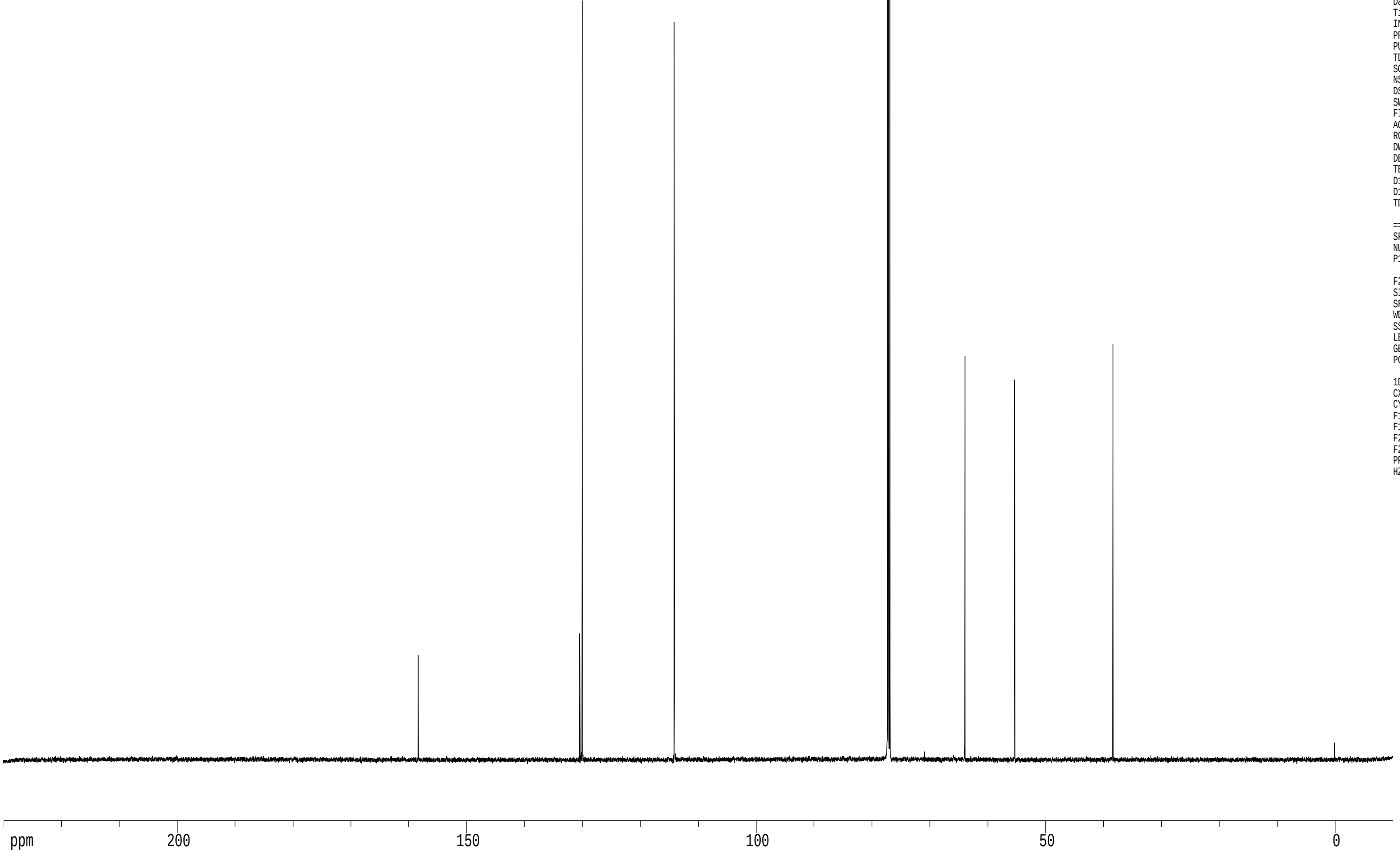


```
Current Data Parameters
USER          nhirbawi
NAME     NH-CAH-1-291-carbon
EXPNO                1
PROCNO               1

F2 - Acquisition Parameters
Date_          20211106
Time              15.56
INSTRUM           av600
PROBHD   5 mm CPBBO BB-
PULPROG          zgdc30
TD                65536
SOLVENT          CDCl3T
NS                  158
DS                    4
SWH           36231.883 Hz
FIDRES         0.552855 Hz
AQ            0.9044468 sec
RG                 2050
DW               13.800 usec
DE                19.63 usec
TE                298.0 K
D1           0.40000001 sec
D11          0.03000000 sec
TD0                   1

======== CHANNEL f1 ========
SFO1        150.9194080 MHz
NUC1                13C
P1                10.10 usec

F2 - Processing parameters
SI                65536
SF          150.9028085 MHz
WDW                  EM
SSB                   0
LB                 1.00 Hz
GB                    0
PC                 1.00

1D NMR plot parameters
CX                22.80 cm
CY                15.65 cm
F1P             230.000 ppm
F1             34707.64 Hz
F2P             -10.000 ppm
F2             -1509.03 Hz
PPMCM          10.52632 ppm/cm
HZCM         1588.45056 Hz/cm
```

1H spectrum

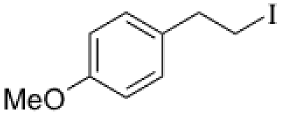

16

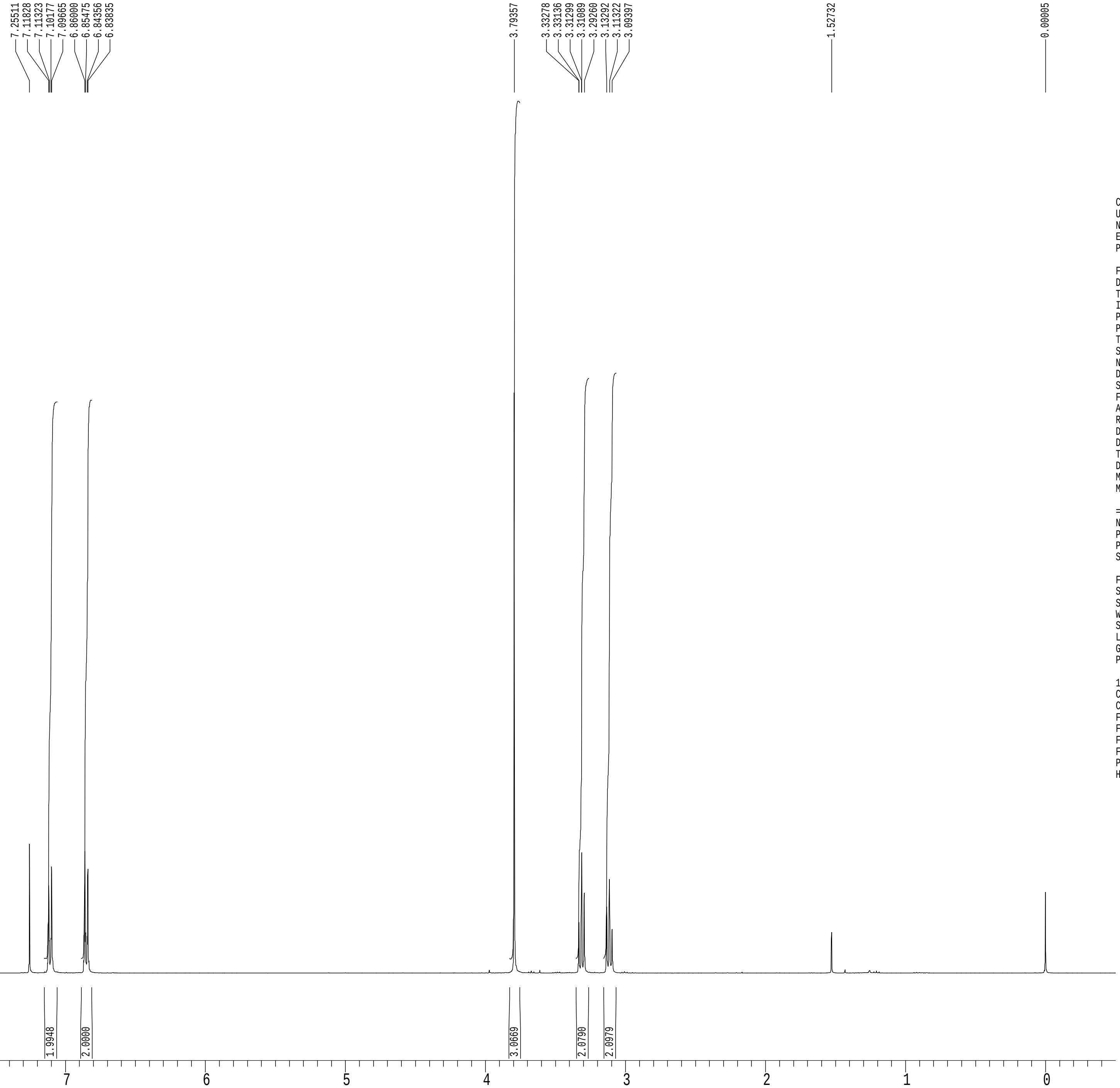

Current Data Parameters
USER nhirbawi
NAME NH-CAH-2-21
EXPNO 1
PROCNO 1

F2 - Acquisition Parameters
Date_ 20211106
Time 14.16
INSTRUM drx400
PROBHD 5 mm QNP H/F/P
PULPROG zg30
TD 65536
SOLVENT CDCl3T
NS 8
DS 2
SWH 6410.256 Hz
FIDRES 0.097813 Hz
AQ 5.1118579 sec
RG 406.4
DW 78.000 usec
DE 4.50 usec
TE 297.9 K
D1 0.10000000 sec
MCREST 0.00000000 sec
MCWRK 0.01500000 sec

======== CHANNEL f1 ========
NUC1 1H
P1 12.00 usec
PL1 -0.90 dB
SFO1 400.1328009 MHz

F2 - Processing parameters
SI 65536
SF 400.1300234 MHz
WDW EM
SSB 0
LB 0.30 Hz
GB 0
PC 2.00

1D NMR plot parameters
CX 22.80 cm
CY 10.00 cm
F1P 9.000 ppm
F1 3601.17 Hz
F2P -0.500 ppm
F2 -200.06 Hz
PPMCM 0.41667 ppm/cm
HZCM 166.72086 Hz/cm

13C spectrum with 1H decoupling

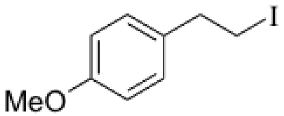

16

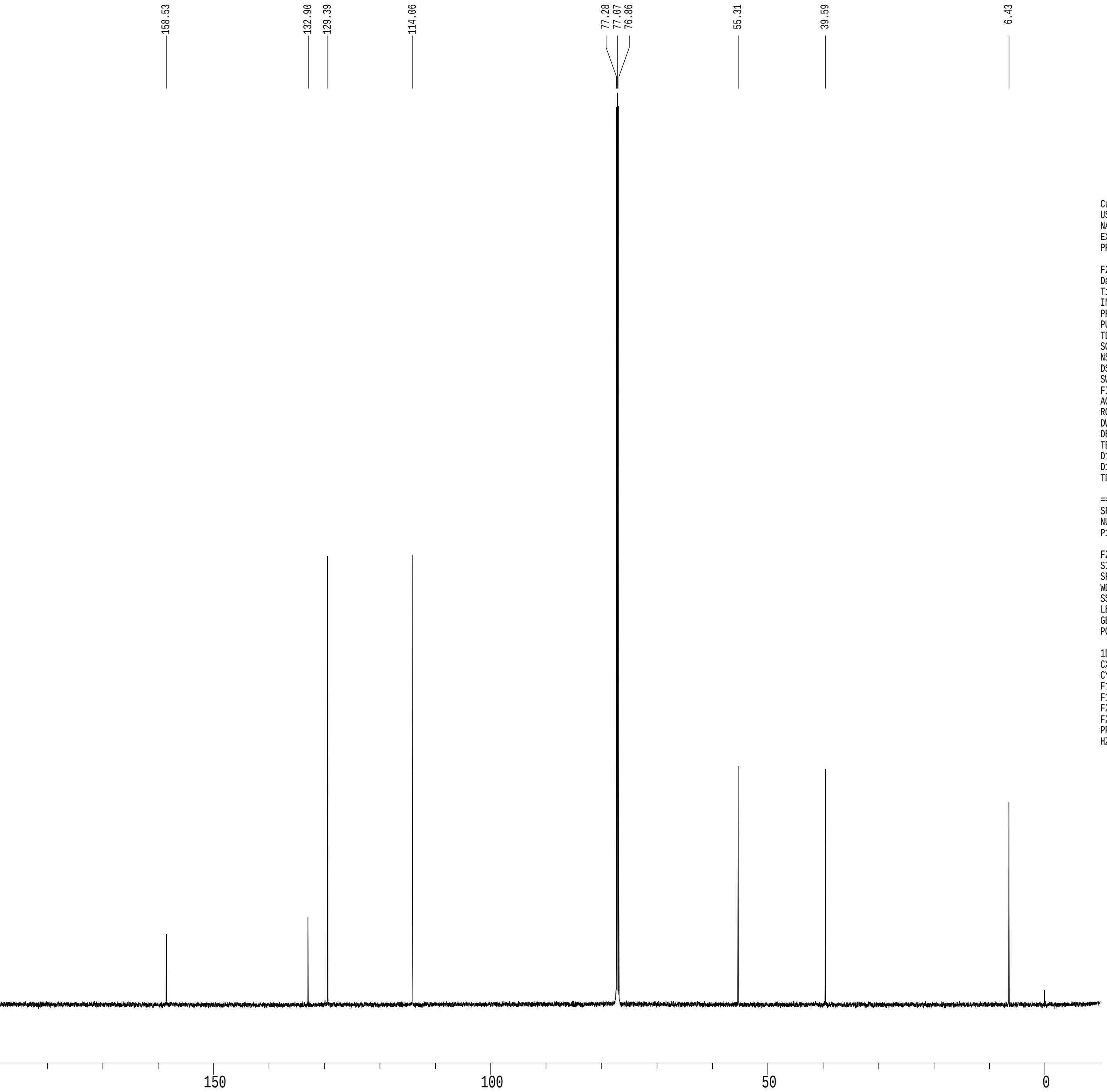

Current Data Parameters
USER nhirbawi
NAME NH-CAH-2-21-carbon
EXPNO 1
PROCNO 1

F2 - Acquisition Parameters
Date_ 20211106
Time 16.07
INSTRUM av600
PROBHD 5 mm CPBBO BB-
PULPROG zgdc30
TD 65536
SOLVENT CDCl3T
NS 151
DS 4
SWH 36231.883 Hz
FIDRES 0.552855 Hz
AQ 0.9044468 sec
RG 2050
DW 13.800 usec
DE 19.63 usec
TE 297.9 K
D1 0.40000001 sec
D11 0.03000000 sec
TD0 1

======== CHANNEL f1 ========
SFO1 150.9194080 MHz
NUC1 13C
P1 10.10 usec

F2 - Processing parameters
SI 65536
SF 150.9028085 MHz
WDW EM
SSB 0
LB 1.00 Hz
GB 0
PC 1.00

1D NMR plot parameters
CX 22.80 cm
CY 15.65 cm
F1P 230.000 ppm
F1 34707.64 Hz
F2P -10.000 ppm
F2 -1509.03 Hz
PPMCM 10.52632 ppm/cm
HZCM 1588.45056 Hz/cm

1H spectrum

17

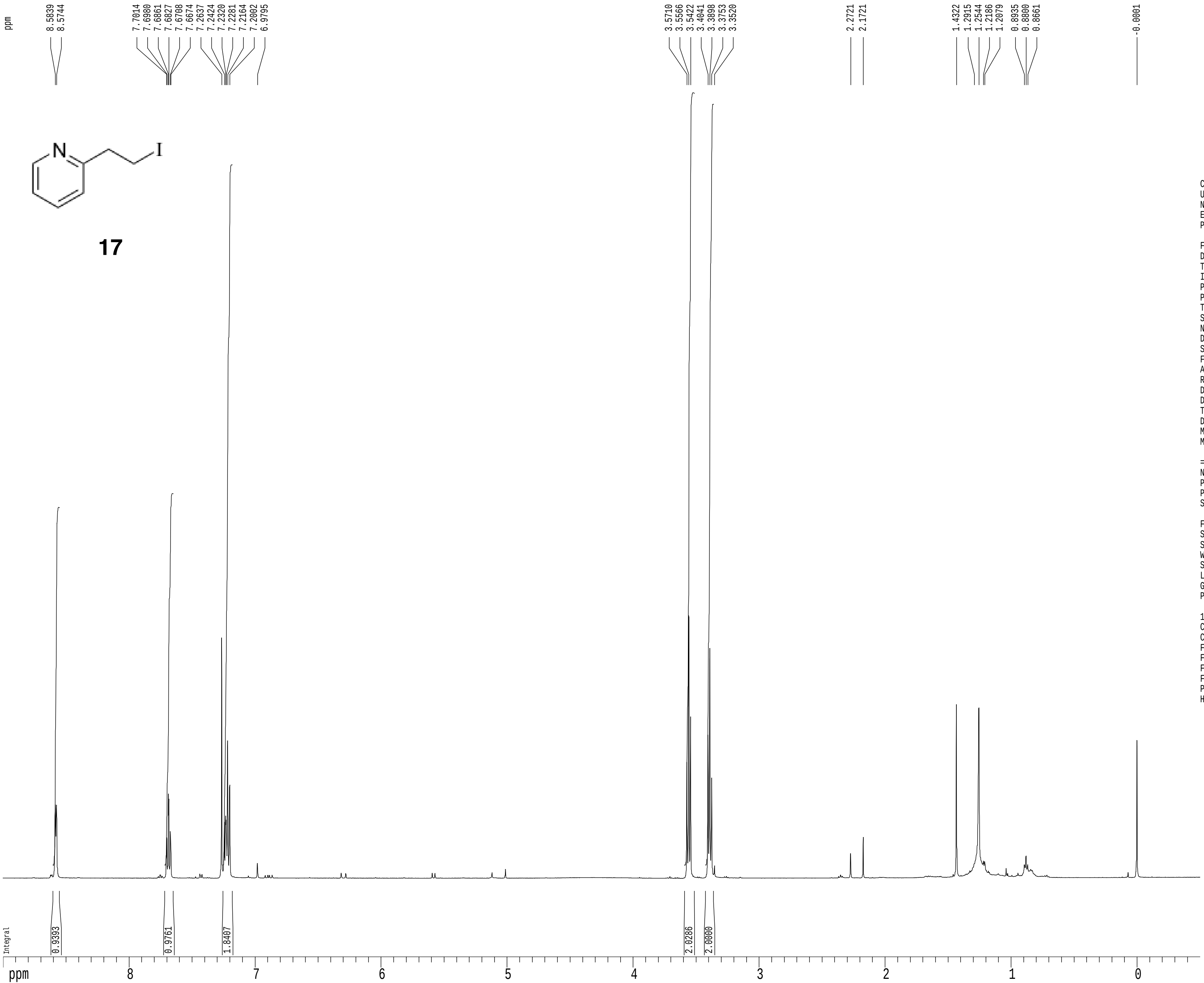

Current Data Parameters
USER nhirbawi
NAME NH-2-172-1H
EXPNO 1
PROCNO 1

F2 - Acquisition Parameters
Date_ 20220514
Time 14.21
INSTRUM cryo500
PROBHD 5 mm CPTCI 1H-
PULPROG zg30
TD 81728
SOLVENT CDCl3T
NS 8
DS 2
SWH 8012.820 Hz
FIDRES 0.098043 Hz
AQ 5.0998774 sec
RG 5.7
DW 62.400 usec
DE 6.00 usec
TE 298.0 K
D1 0.10000000 sec
MCREST 0.00000000 sec
MCWRK 0.01500000 sec

======== CHANNEL f1 ========
NUC1 1H
P1 9.75 usec
PL1 1.60 dB
SFO1 500.2235015 MHz

F2 - Processing parameters
SI 65536
SF 500.2200304 MHz
WDW EM
SSB 0
LB 0.30 Hz
GB 0
PC 1.00

1D NMR plot parameters
CX 22.80 cm
CY 5.00 cm
F1P 9.000 ppm
F1 4501.98 Hz
F2P -0.500 ppm
F2 -250.11 Hz
PPMCM 0.41667 ppm/cm
HZCM 208.42502 Hz/cm

Z-restored spin-echo 13C spectrum with 1H decoupling

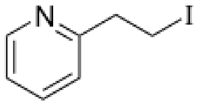

17

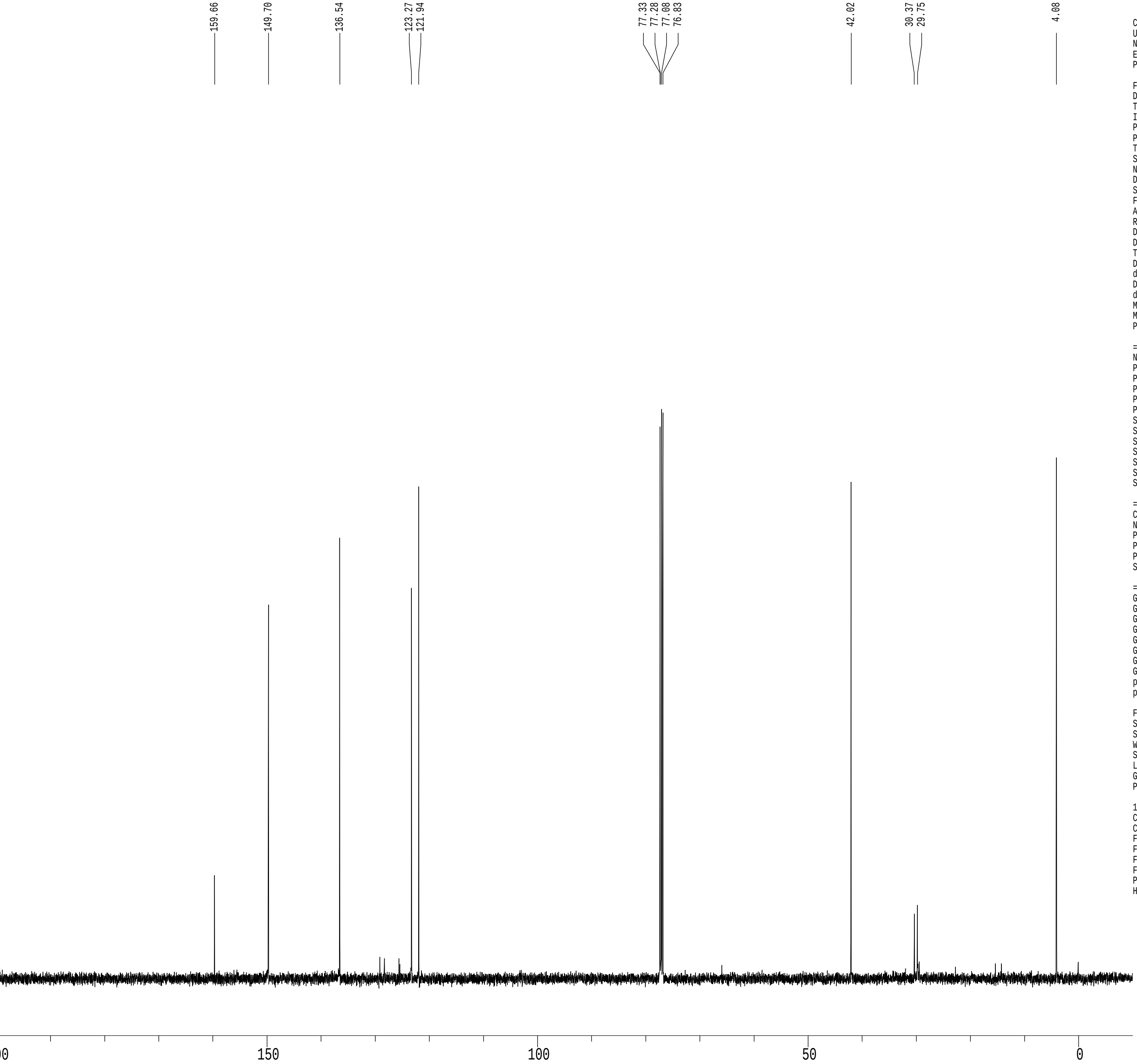


```
Current Data Parameters
USER          nhirbawi
NAME     NH-2-172-carbon
EXPNO                2
PROCNO               1

F2 - Acquisition Parameters
Date_         20220202
Time             17.26
INSTRUM        cryo500
PROBHD   5 mm CPTCI 1H-
PULPROG  SpinEchopg30gp2.prd
TD               65536
SOLVENT          CDCl3
NS                 303
DS                  16
SWH          30303.031 Hz
FIDRES        0.462388 Hz
AQ           1.0813940 sec
RG              7298.2
DW              16.500 usec
DE                6.00 usec
TE               298.0 K
D1          0.25000000 sec
d11         0.03000000 sec
D16         0.00020000 sec
d17         0.00019600 sec
MCREST      0.00000000 sec
MCWRK       0.01500000 sec
P2               37.70 usec

======== CHANNEL f1 ========
NUC1               13C
P1               18.85 usec
P12            2000.00 usec
P20             500.00 usec
PL0             120.00 dB
PL1              -1.00 dB
SFO1       125.7942548 MHz
SP2               1.55 dB
SP4               1.55 dB
SPNAM2      Crp60comp.4
SPNAM4   Crp60,0.5,20.1
SPOFF2            0.00 Hz
SPOFF4            0.00 Hz

======== CHANNEL f2 ========
CPDPRG2         waltz16
NUC2                 1H
PCPD2           100.00 usec
PL2               1.60 dB
PL12             22.00 dB
SFO2       500.2225011 MHz

====== GRADIENT CHANNEL =====
GPNAM1         SINE.100
GPNAM2         SINE.100
GPX1              0.00 %
GPX2              0.00 %
GPY1              0.00 %
GPY2              0.00 %
GPZ1             30.00 %
GPZ2             50.00 %
p15             500.00 usec
p16            1000.00 usec

F2 - Processing parameters
SI               65536
SF         125.7804190 MHz
WDW                 EM
SSB                  0
LB                1.00 Hz
GB                   0
PC                2.00

1D NMR plot parameters
CX               22.80 cm
CY               10.00 cm
F1P            230.000 ppm
F1            28929.50 Hz
F2P            -10.000 ppm
F2            -1257.80 Hz
PPMCM         10.52632 ppm/cm
HZCM        1324.00439 Hz/cm
```

1H spectrum

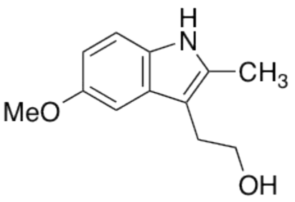

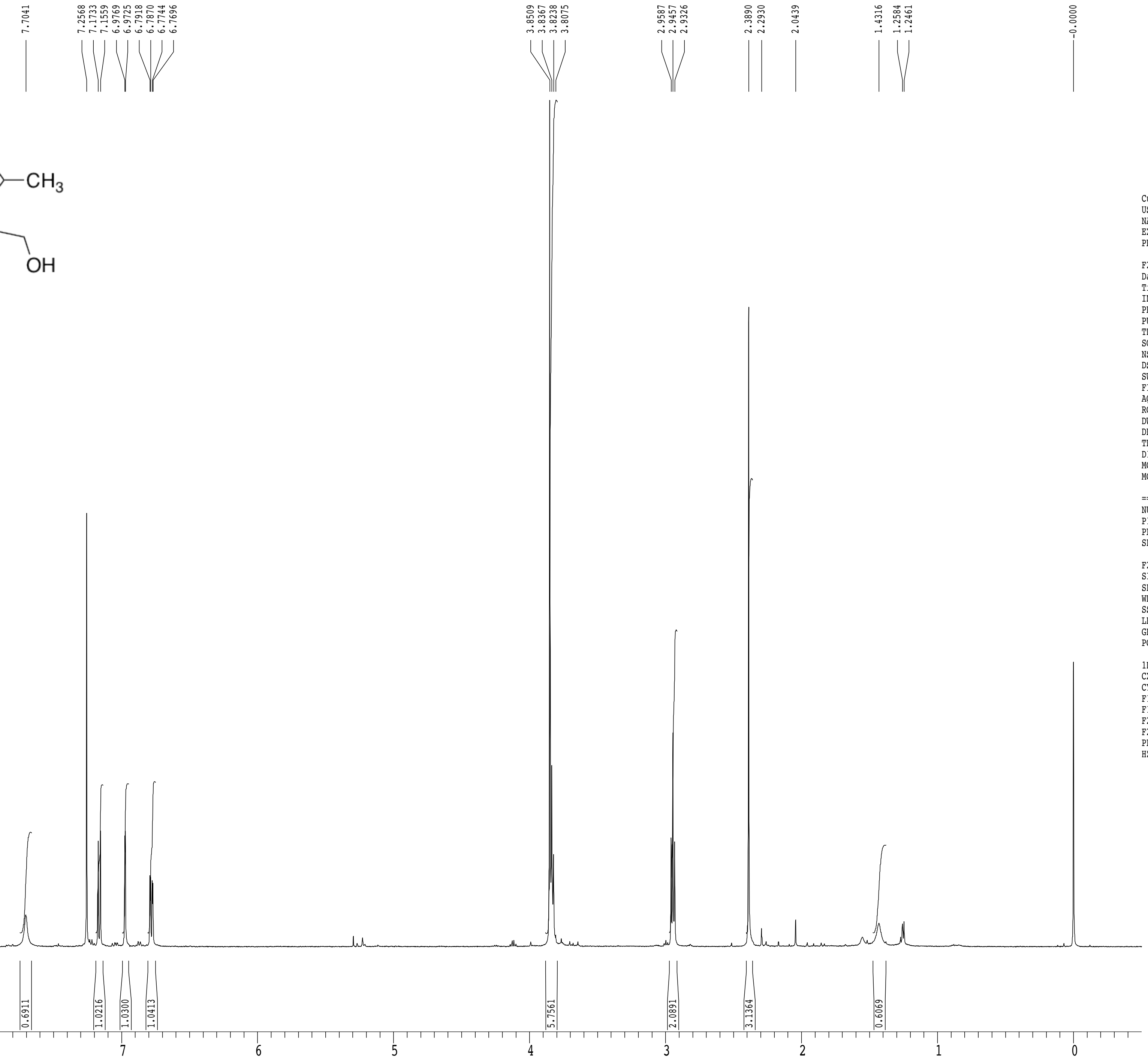

Current Data Parameters
USER linpc2
NAME pcl-3-096
EXPNO 4
PROCNO 1

F2 - Acquisition Parameters
Date_ 20220412
Time 8.14
INSTRUM gn500
PROBHD 5 mm broadband
PULPROG zg30
TD 81728
SOLVENT CDCl3
NS 8
DS 2
SWH 8012.820 Hz
FIDRES 0.098043 Hz
AQ 5.0998774 sec
RG 2896.3
DW 62.400 usec
DE 6.00 usec
TE 298.0 K
D1 0.10000000 sec
MCREST 0.00000000 sec
MCWRK 0.01500000 sec

======== CHANNEL f1 ========
NUC1 1H
P1 12.00 usec
PL1 -6.00 dB
SFO1 498.6534906 MHz

F2 - Processing parameters
SI 65536
SF 498.6500299 MHz
WDW EM
SSB 0
LB 0.30 Hz
GB 0
PC 1.00

1D NMR plot parameters
CX 22.80 cm
CY 15.00 cm
F1P 9.000 ppm
F1 4487.85 Hz
F2P -0.500 ppm
F2 -249.32 Hz
PPMCM 0.41667 ppm/cm
HZCM 207.77084 Hz/cm

1H spectrum

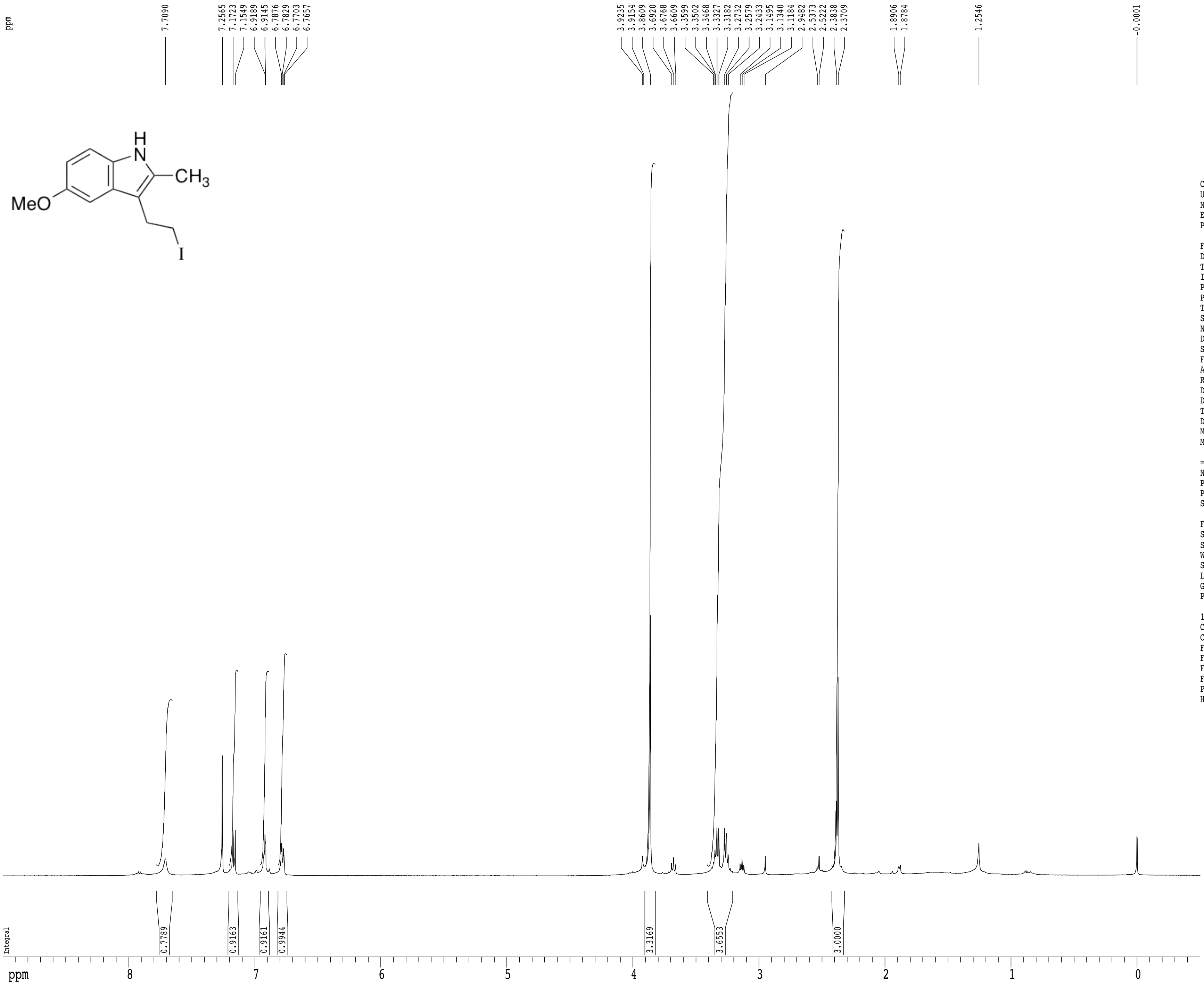


```
Current Data Parameters
USER               linpc2
NAME            pcl-3-107
EXPNO                   5
PROCNO                  1

F2 - Acquisition Parameters
Date_            20220421
Time                14.25
INSTRUM          cryo500
PROBHD   5 mm CPTCI 1H-
PULPROG              zg30
TD                  81728
SOLVENT             CDCl3
NS                      8
DS                      2
SWH              8012.820 Hz
FIDRES           0.098043 Hz
AQ              5.0998774 sec
RG                    5.7
DW                 62.400 usec
DE                   6.00 usec
TE                  298.0 K
D1             0.10000000 sec
MCREST         0.00000000 sec
MCWRK          0.01500000 sec

======== CHANNEL f1 ========
NUC1                   1H
P1                   9.75 usec
PL1                  1.60 dB
SFO1          500.2235015 MHz

F2 - Processing parameters
SI                  65536
SF            500.2200335 MHz
WDW                    EM
SSB                     0
LB                   0.30 Hz
GB                      0
PC                   1.00

1D NMR plot parameters
CX                  22.80 cm
CY                   5.00 cm
F1P                 9.000 ppm
F1                4501.98 Hz
F2P                -0.500 ppm
F2                -250.11 Hz
PPMCM             0.41667 ppm/cm
HZCM            208.42502 Hz/cm
```

Z-restored spin-echo 13C spectrum with 1H decoupling

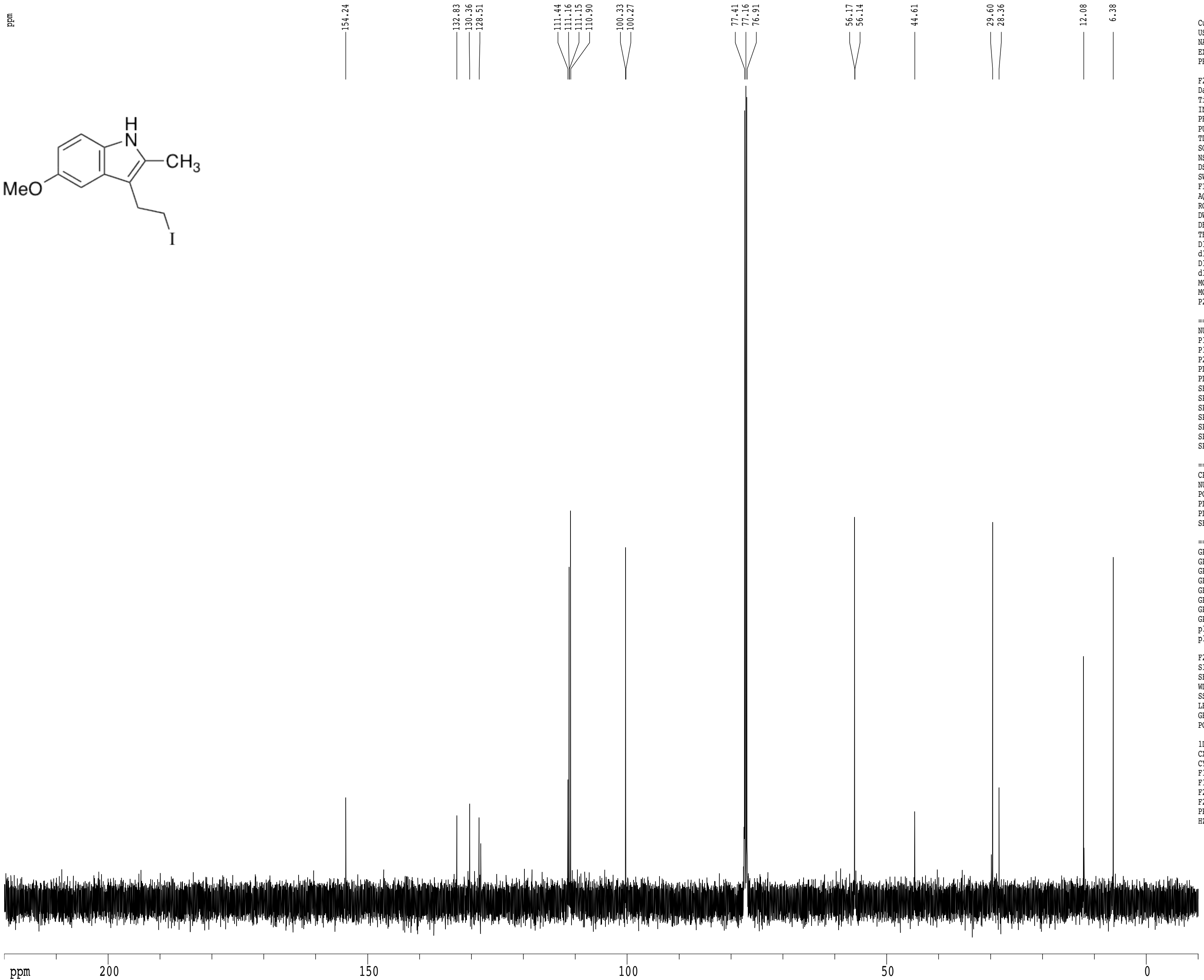


```
Current Data Parameters
USER              linpc2
NAME           pcl-3-107
EXPNO                  6
PROCNO                 1

F2 - Acquisition Parameters
Date_           20220421
Time               14.29
INSTRUM          cryo500
PROBHD   5 mm CPTCI 1H-
PULPROG  SpinEchopg30gp2.prd
TD                 65536
SOLVENT            CDCl3
NS                   496
DS                    16
SWH            30303.031 Hz
FIDRES          0.462388 Hz
AQ             1.0813940 sec
RG                2896.3
DW                16.500 usec
DE                  6.00 usec
TE                 298.0 K
D1            0.25000000 sec
d11           0.03000000 sec
D16           0.00020000 sec
d17           0.00019600 sec
MCREST        0.00000000 sec
MCWRK         0.01500000 sec
P2                 37.70 usec

======== CHANNEL f1 ========
NUC1                 13C
P1                 18.85 usec
P12              2000.00 usec
P20               500.00 usec
PL0               120.00 dB
PL1                -1.00 dB
SFO1         125.7942548 MHz
SP2                 1.55 dB
SP4                 1.55 dB
SPNAM2       Crp60comp.4
SPNAM4    Crp60,0.5,20.1
SPOFF2              0.00 Hz
SPOFF4              0.00 Hz

======== CHANNEL f2 ========
CPDPRG2          waltz16
NUC2                  1H
PCPD2             100.00 usec
PL2                 1.60 dB
PL12               22.00 dB
SFO2         500.2225011 MHz

====== GRADIENT CHANNEL =====
GPNAM1          SINE.100
GPNAM2          SINE.100
GPX1                0.00 %
GPX2                0.00 %
GPY1                0.00 %
GPY2                0.00 %
GPZ1               30.00 %
GPZ2               50.00 %
p15               500.00 usec
p16              1000.00 usec

F2 - Processing parameters
SI                 65536
SF           125.7804090 MHz
WDW                   no
SSB                    0
LB                  0.00 Hz
GB                     0
PC                  2.00

1D NMR plot parameters
CX                 22.80 cm
CY                 15.65 cm
F1P              220.000 ppm
F1              27671.69 Hz
F2P              -10.000 ppm
F2              -1257.80 Hz
PPMCM           10.08772 ppm/cm
HZCM          1268.83752 Hz/cm
```

1H spectrum

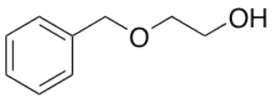

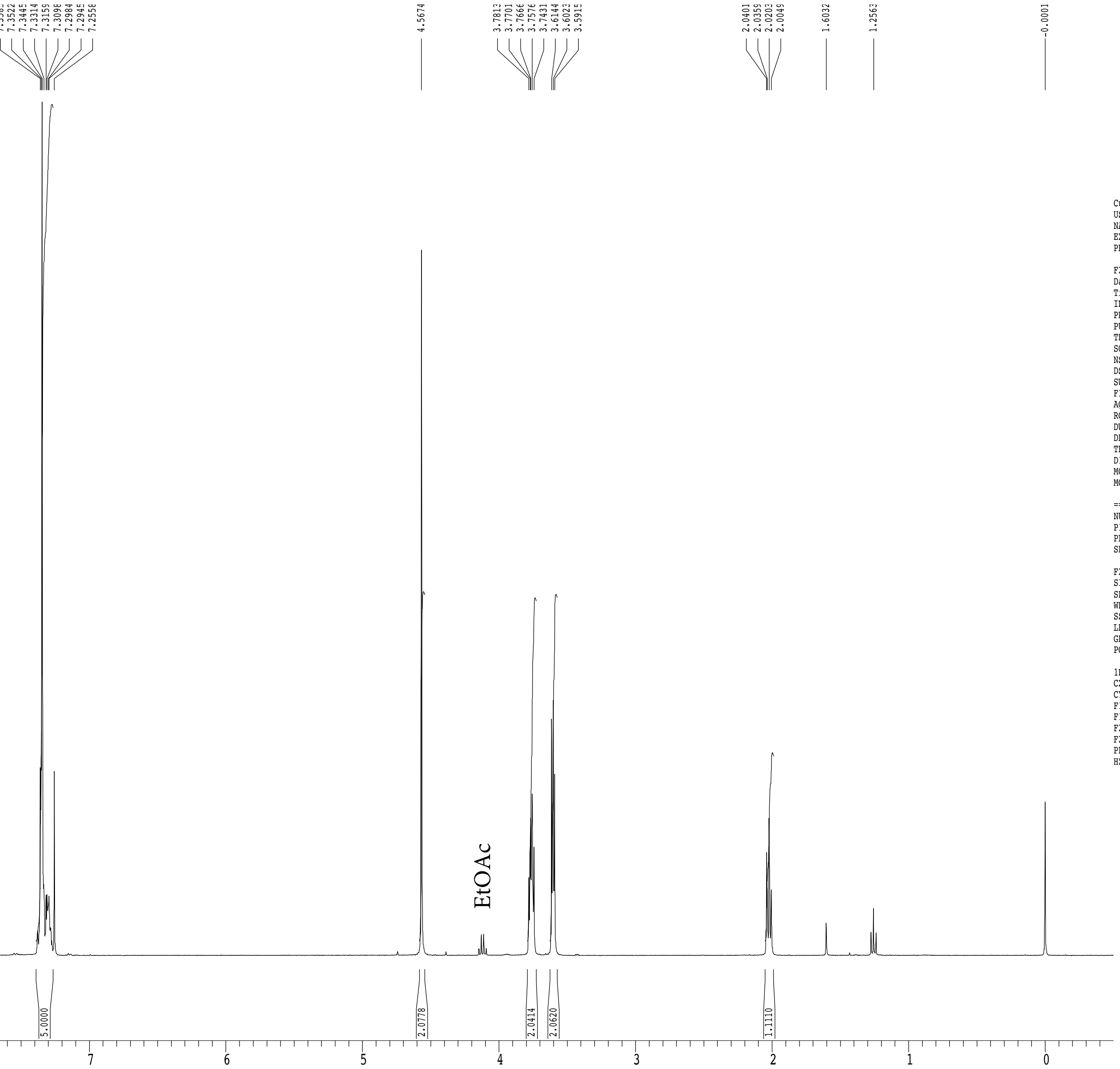


```
Current Data Parameters
USER              linpc2
NAME           pcl-2-212
EXPNO                  1
PROCNO                 1

F2 - Acquisition Parameters
Date_           20211019
Time               17.23
INSTRUM           drx400
PROBHD   5 mm Multinucl
PULPROG             zg30
TD                 65536
SOLVENT            CDCl3
NS                     8
DS                     2
SWH             6410.256 Hz
FIDRES          0.097813 Hz
AQ             5.1118579 sec
RG                 322.5
DW                78.000 usec
DE                  4.50 usec
TE                 298.0 K
D1            0.10000000 sec
MCREST        0.00000000 sec
MCWRK         0.01500000 sec

======== CHANNEL f1 ========
NUC1                  1H
P1                 12.00 usec
PL1                -1.10 dB
SFO1         400.1328009 MHz

F2 - Processing parameters
SI                 65536
SF           400.1300232 MHz
WDW                   EM
SSB                    0
LB                  0.30 Hz
GB                     0
PC                  2.00

1D NMR plot parameters
CX                 22.80 cm
CY                 15.00 cm
F1P                9.000 ppm
F1               3601.17 Hz
F2P               -0.500 ppm
F2               -200.06 Hz
PPMCM            0.41667 ppm/cm
HZCM           166.72086 Hz/cm
```

1H spectrum

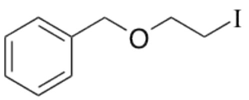

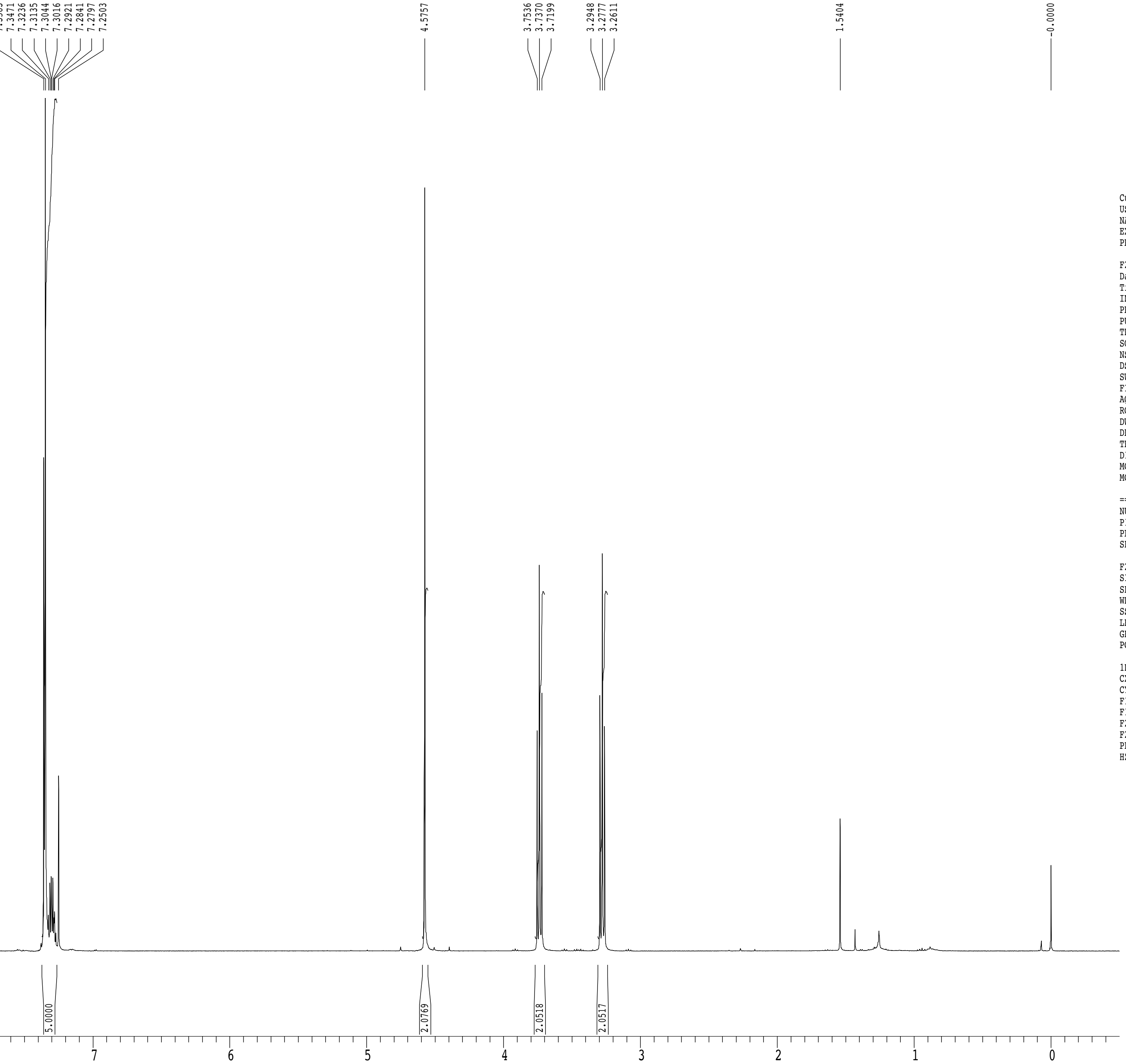


```
Current Data Parameters
USER            linpc2
NAME         pcl-2-225
EXPNO                2
PROCNO               1

F2 - Acquisition Parameters
Date_         20211103
Time             15.33
INSTRUM         drx400
PROBHD   5 mm QNP H/F/P
PULPROG           zg30
TD               65536
SOLVENT          CDCl3
NS                   8
DS                   2
SWH           6410.256 Hz
FIDRES        0.097813 Hz
AQ           5.1118579 sec
RG                 256
DW              78.000 usec
DE                4.50 usec
TE               298.0 K
D1          0.10000000 sec
MCREST      0.00000000 sec
MCWRK       0.01500000 sec

======== CHANNEL f1 ========
NUC1                1H
P1               12.00 usec
PL1              -0.90 dB
SFO1       400.1328009 MHz

F2 - Processing parameters
SI               65536
SF         400.1300253 MHz
WDW                 EM
SSB                  0
LB                0.30 Hz
GB                   0
PC                2.00

1D NMR plot parameters
CX               22.80 cm
CY               15.00 cm
F1P              9.000 ppm
F1             3601.17 Hz
F2P             -0.500 ppm
F2             -200.06 Hz
PPMCM          0.41667 ppm/cm
HZCM         166.72086 Hz/cm
```

13C spectrum with 1H decoupling

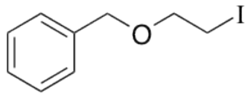

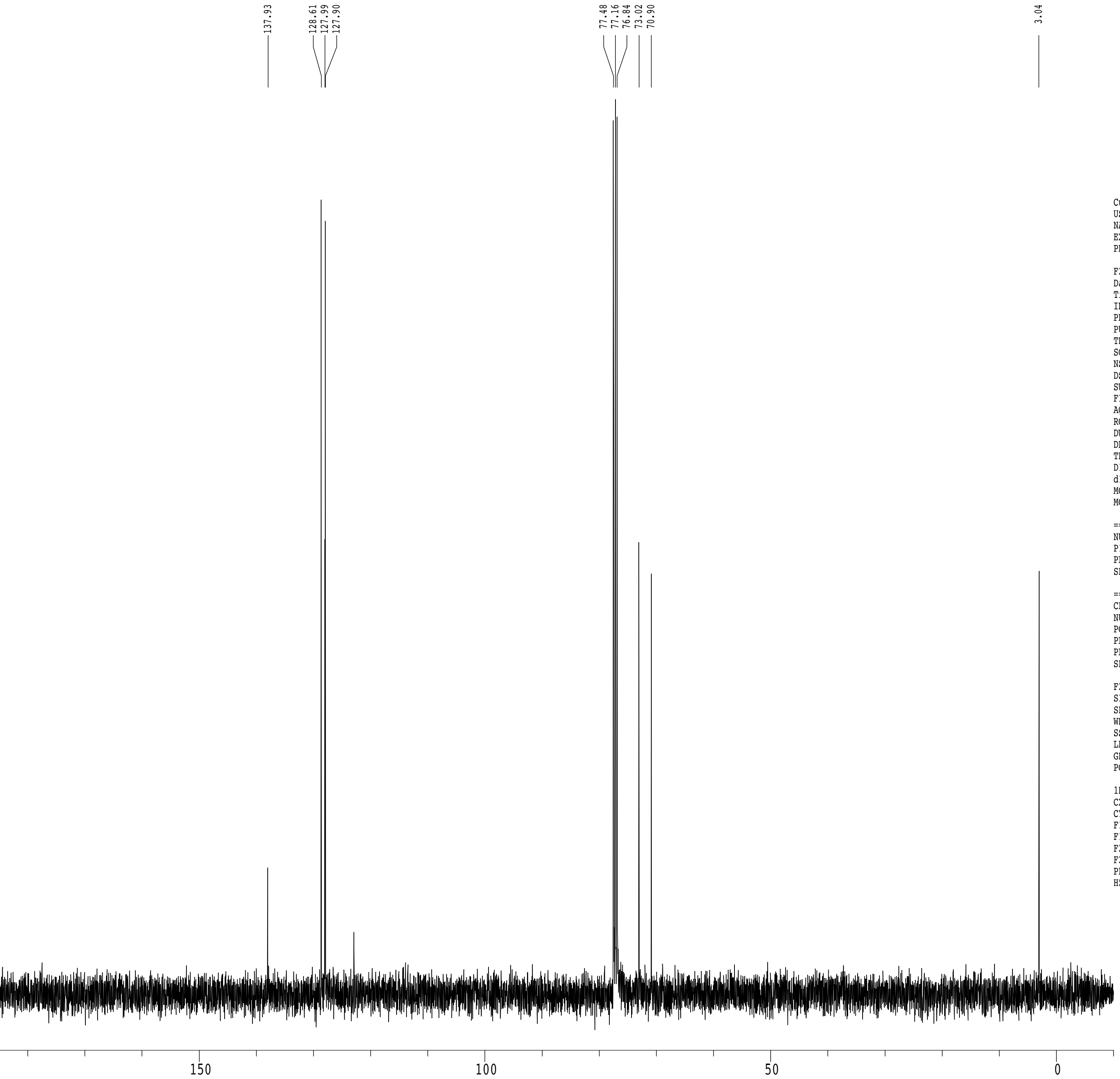


```
Current Data Parameters
USER            linpc2
NAME         pcl-2-225
EXPNO                3
PROCNO               1

F2 - Acquisition Parameters
Date_         20211103
Time             15.35
INSTRUM         drx400
PROBHD   5 mm QNP H/F/P
PULPROG         zgdc30
TD               65536
SOLVENT          CDCl3
NS                 104
DS                   4
SWH          24154.590 Hz
FIDRES        0.368570 Hz
AQ           1.3566452 sec
RG              9195.2
DW              20.700 usec
DE               20.39 usec
TE               298.0 K
D1          0.10000000 sec
d11         0.03000000 sec
MCREST      0.00000000 sec
MCWRK       0.01500000 sec

======== CHANNEL f1 ========
NUC1               13C
P1                7.90 usec
PL1              -3.00 dB
SFO1       100.6237964 MHz

======== CHANNEL f2 ========
CPDPRG2        waltz16
NUC2                1H
PCPD2            90.00 usec
PL2              -0.90 dB
PL12             17.00 dB
SFO2       400.1328009 MHz

F2 - Processing parameters
SI               65536
SF         100.6127591 MHz
WDW                 EM
SSB                  0
LB                1.00 Hz
GB                   0
PC                1.00

1D NMR plot parameters
CX               22.80 cm
CY               15.50 cm
F1P            220.000 ppm
F1            22134.81 Hz
F2P            -10.000 ppm
F2            -1006.13 Hz
PPMCM         10.08772 ppm/cm
HZCM        1014.95337 Hz/cm
```

1H spectrum

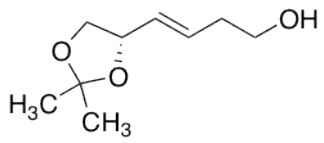

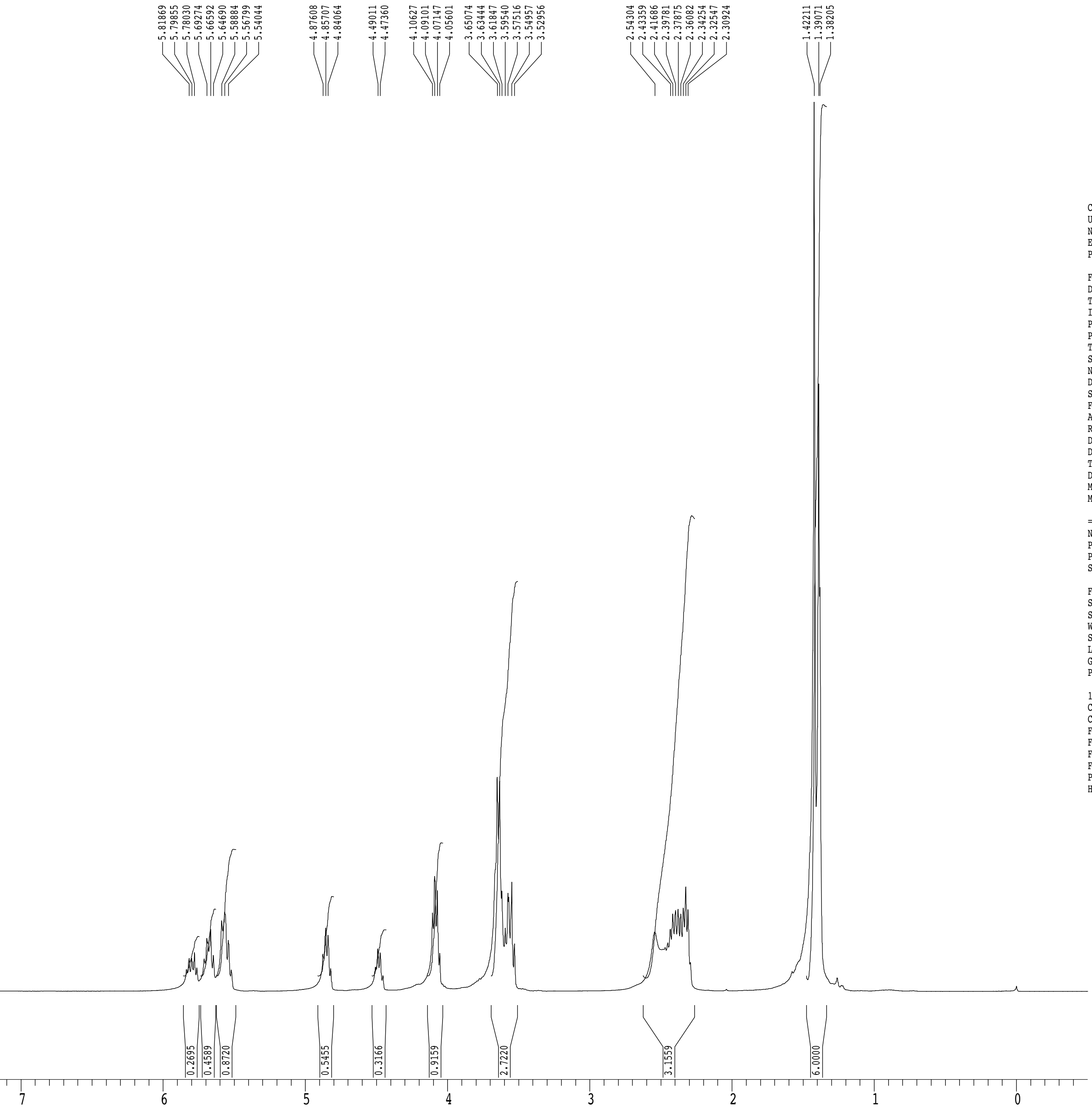

Current Data Parameters
USER linpc2
NAME pcl-3-067
EXPNO 3
PROCNO 1

F2 - Acquisition Parameters
Date_ 20220309
Time 10.14
INSTRUM drx400
PROBHD 5 mm QNP H/F/P
PULPROG zg30
TD 38460
SOLVENT CDCl3
NS 8
DS 2
SWH 6410.256 Hz
FIDRES 0.166673 Hz
AQ 2.9999299 sec
RG 32
DW 78.000 usec
DE 4.50 usec
TE 298.0 K
D1 0.10000000 sec
MCREST 0.00000000 sec
MCWRK 0.01500000 sec

======== CHANNEL f1 ========
NUC1 1H
P1 12.00 usec
PL1 -0.90 dB
SFO1 400.1328009 MHz

F2 - Processing parameters
SI 65536
SF 400.1299960 MHz
WDW EM
SSB 0
LB 0.30 Hz
GB 0
PC 2.00

1D NMR plot parameters
CX 22.80 cm
CY 15.00 cm
F1P 9.000 ppm
F1 3601.17 Hz
F2P -0.500 ppm
F2 -200.06 Hz
PPMCM 0.41667 ppm/cm
HZCM 166.72084 Hz/cm

1H spectrum

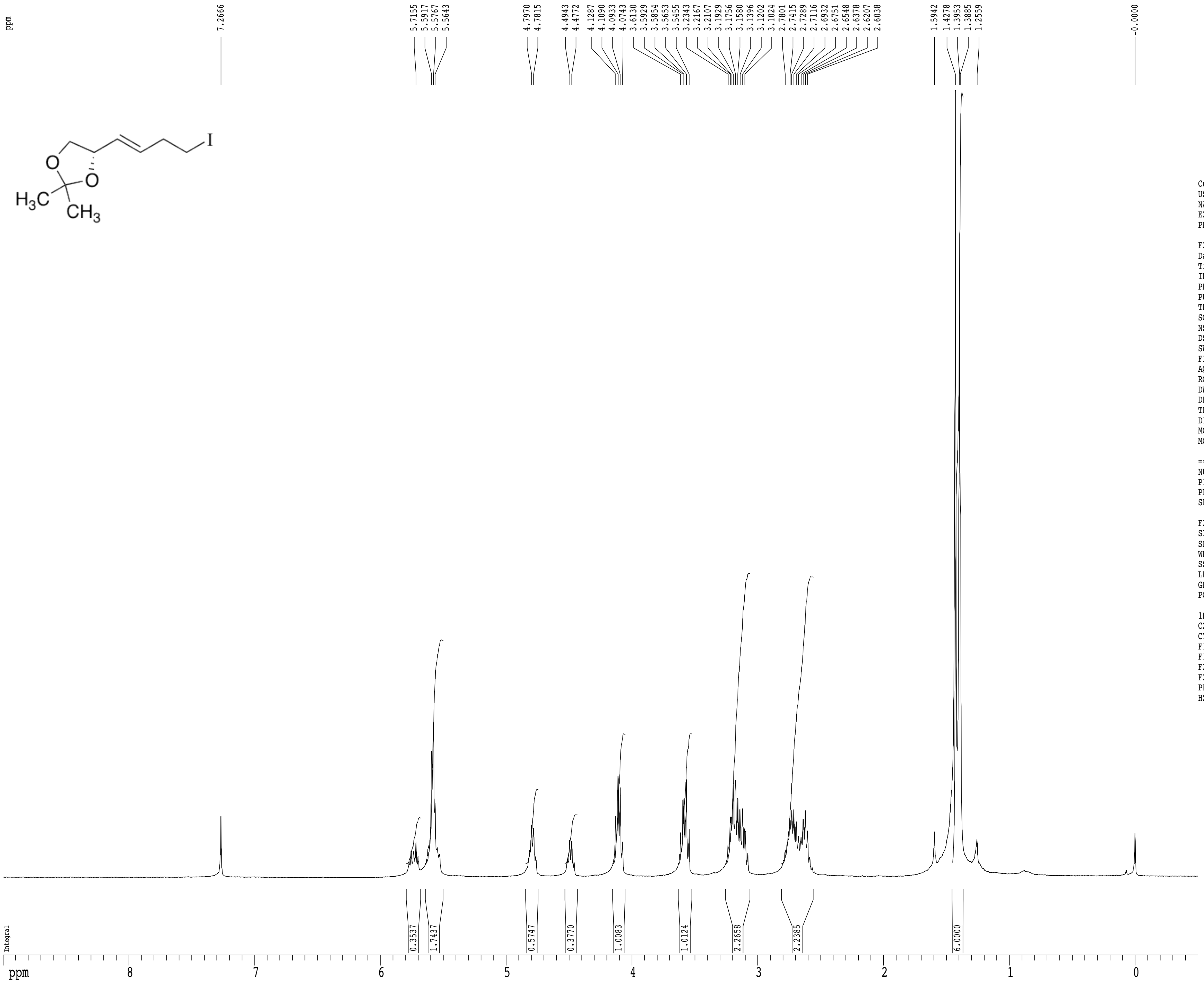

Current Data Parameters
USER linpc2
NAME pcl-3-069
EXPNO 2
PROCNO 1

F2 - Acquisition Parameters
Date_ 20220310
Time 13.40
INSTRUM drx400
PROBHD 5 mm QNP H/F/P
PULPROG zg30
TD 38460
SOLVENT CDCl3
NS 8
DS 2
SWH 6410.256 Hz
FIDRES 0.166673 Hz
AQ 2.9999299 sec
RG 181
DW 78.000 usec
DE 4.50 usec
TE 298.0 K
D1 0.10000000 sec
MCREST 0.00000000 sec
MCWRK 0.01500000 sec

======== CHANNEL f1 ========
NUC1 1H
P1 12.00 usec
PL1 -0.90 dB
SFO1 400.1328009 MHz

F2 - Processing parameters
SI 65536
SF 400.1300184 MHz
WDW EM
SSB 0
LB 0.30 Hz
GB 0
PC 2.00

1D NMR plot parameters
CX 22.80 cm
CY 15.00 cm
F1P 9.000 ppm
F1 3601.17 Hz
F2P -0.500 ppm
F2 -200.06 Hz
PPMCM 0.41667 ppm/cm
HZCM 166.72084 Hz/cm

1H spectrum

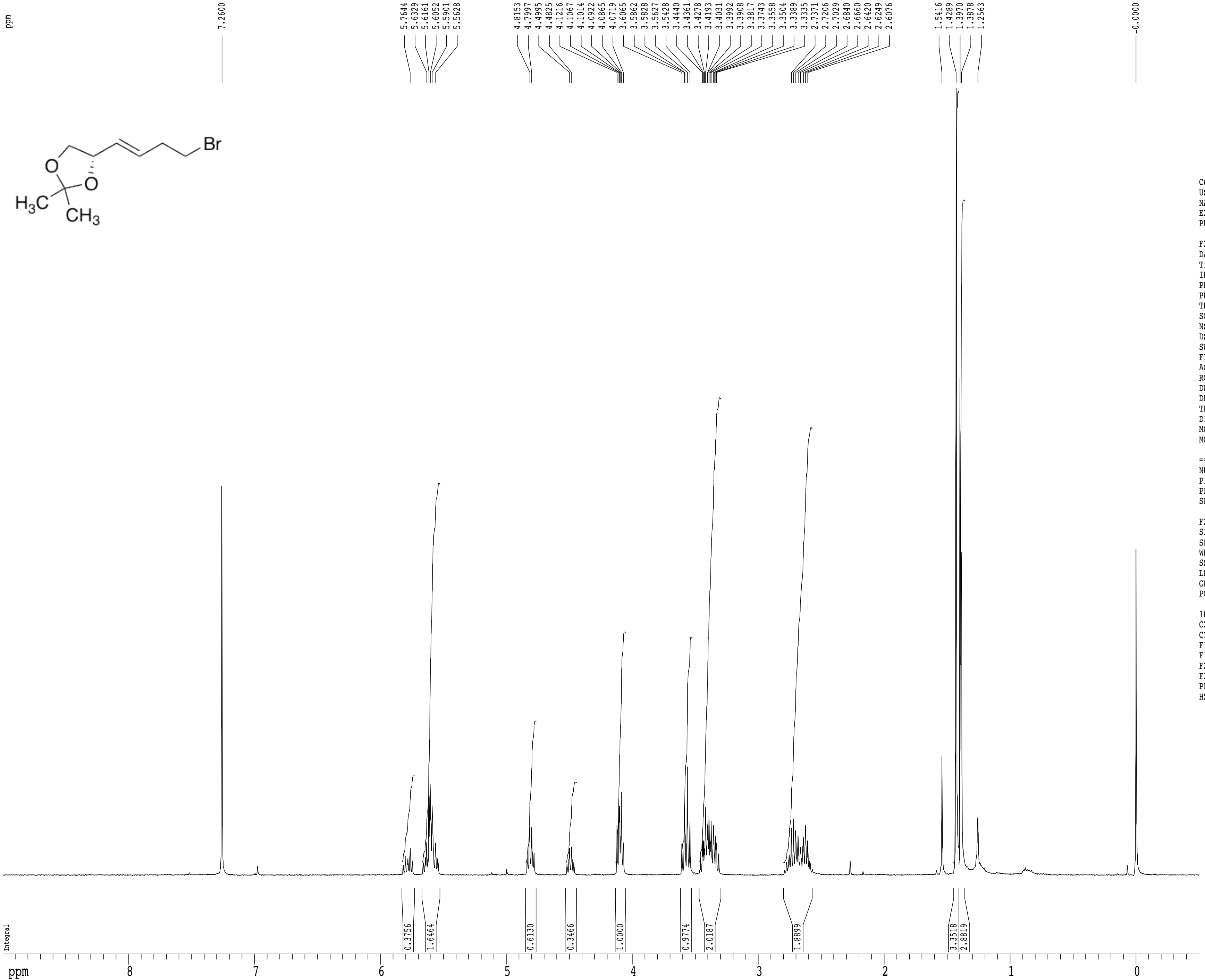

Current Data Parameters
USER linpc2
NAME pcl-3-086
EXPNO 3
PROCNO 1

F2 - Acquisition Parameters
Date_ 20220323
Time 8.10
INSTRUM drx400
PROBHD 5 mm QNP H/F/P
PULPROG zg30
TD 38460
SOLVENT CDCl3
NS 8
DS 2
SWH 6410.256 Hz
FIDRES 0.166673 Hz
AQ 2.9999299 sec
RG 512
DW 78.000 usec
DE 4.50 usec
TE 298.0 K
D1 0.10000000 sec
MCREST 0.00000000 sec
MCWRK 0.01500000 sec

======== CHANNEL f1 ========
NUC1 1H
P1 12.00 usec
PL1 -0.90 dB
SFO1 400.1328009 MHz

F2 - Processing parameters
SI 65536
SF 400.1300214 MHz
WDW EM
SSB 0
LB 0.30 Hz
GB 0
PC 2.00

1D NMR plot parameters
CX 22.80 cm
CY 15.00 cm
F1P 9.000 ppm
F1 3601.17 Hz
F2P -0.500 ppm
F2 -200.06 Hz
PPMCM 0.41667 ppm/cm
HZCM 166.72086 Hz/cm

13C spectrum with 1H decoupling

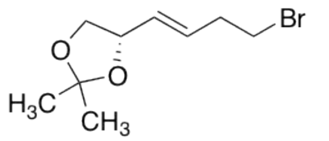

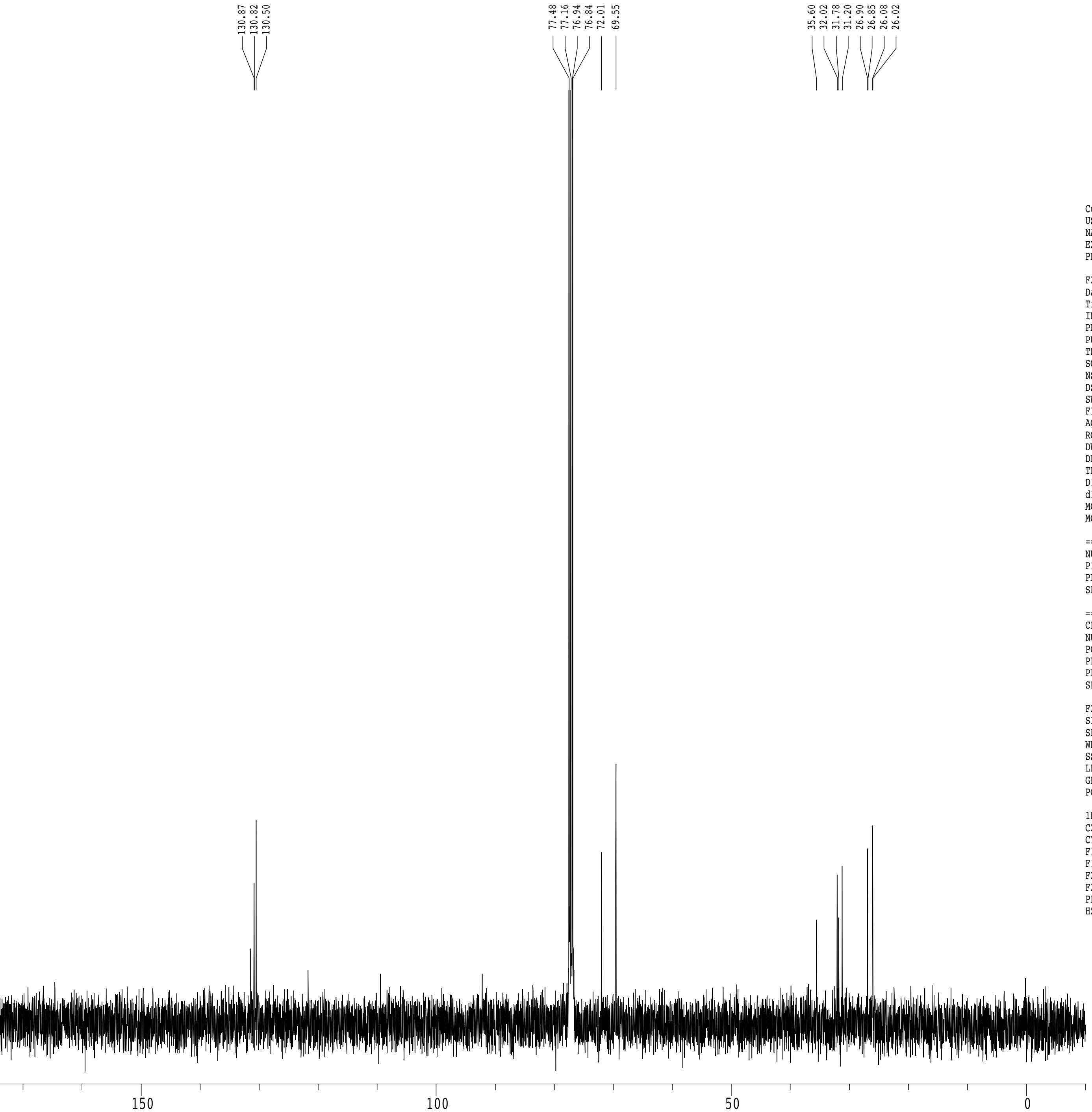

Current Data Parameters
USER linpc2
NAME pcl-3-086
EXPNO 4
PROCNO 1

F2 - Acquisition Parameters
Date_ 20220323
Time 8.13
INSTRUM drx400
PROBHD 5 mm QNP H/F/P
PULPROG zgdc30
TD 65536
SOLVENT CDCl3
NS 256
DS 4
SWH 24154.590 Hz
FIDRES 0.368570 Hz
AQ 1.3566452 sec
RG 14596.5
DW 20.700 usec
DE 20.39 usec
TE 298.0 K
D1 0.10000000 sec
d11 0.03000000 sec
MCREST 0.00000000 sec
MCWRK 0.01500000 sec

======== CHANNEL f1 ========
NUC1 13C
P1 7.90 usec
PL1 -3.00 dB
SFO1 100.6237964 MHz

======== CHANNEL f2 ========
CPDPRG2 waltz16
NUC2 1H
PCPD2 90.00 usec
PL2 -0.90 dB
PL12 17.00 dB
SFO2 400.1328009 MHz

F2 - Processing parameters
SI 65536
SF 100.6127562 MHz
WDW EM
SSB 0
LB 1.00 Hz
GB 0
PC 1.00

1D NMR plot parameters
CX 22.80 cm
CY 30.00 cm
F1P 220.000 ppm
F1 22134.81 Hz
F2P -10.000 ppm
F2 -1006.13 Hz
PPMCM 10.08772 ppm/cm
HZCM 1014.95325 Hz/cm

1H spectrum

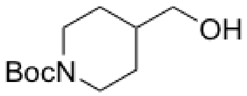

61

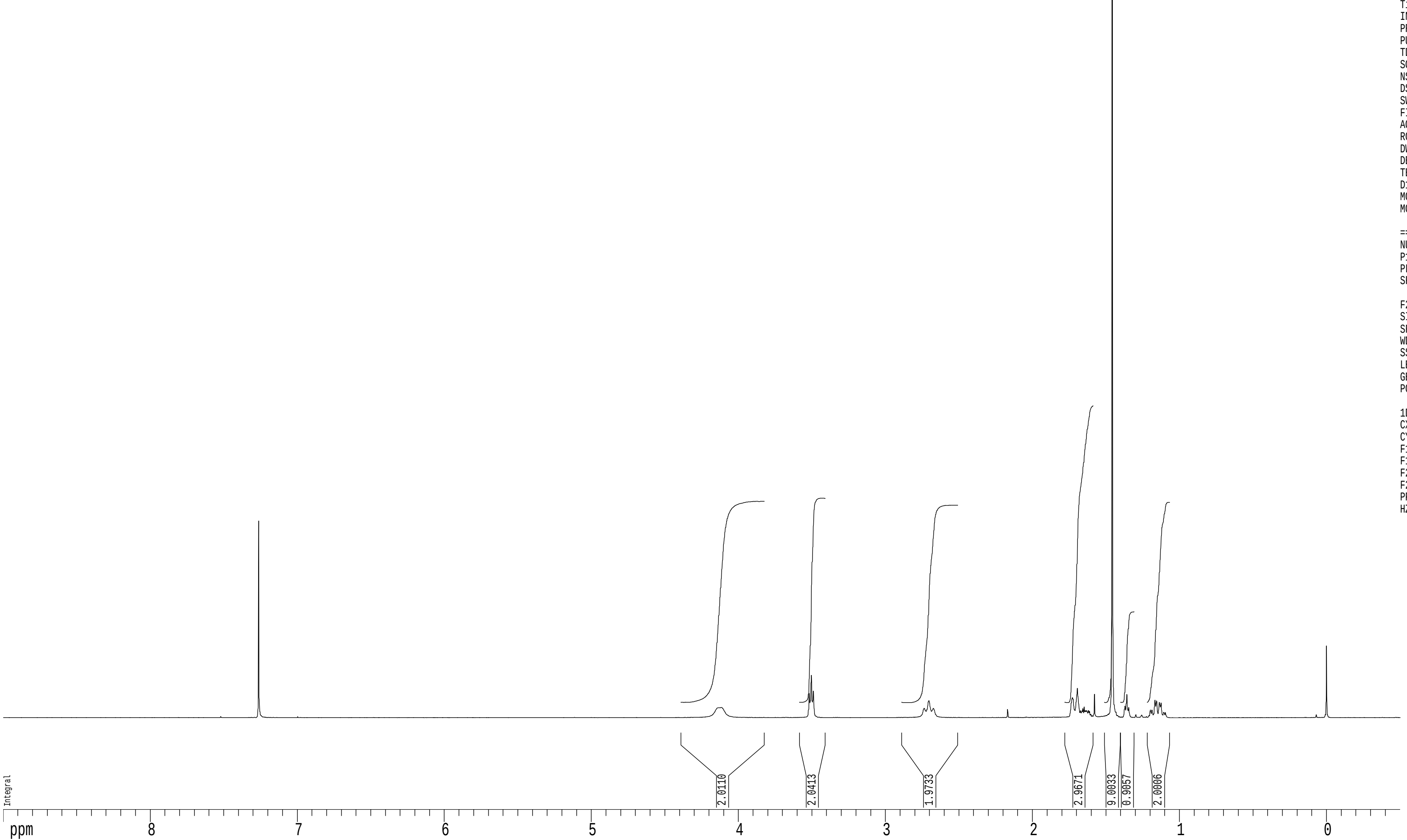

Current Data Parameters

USER	nhirbawi
NAME	NH-2-149-column
EXPNO	1
PROCNO	1

F2 - Acquisition Parameters

Date_	20220117
Time	11.09
INSTRUM	drx400
PROBHD	5 mm QNP H/F/P
PULPROG	zg30
TD	65536
SOLVENT	CDCl3T
NS	8
DS	2
SWH	6410.256 Hz
FIDRES	0.097813 Hz
AQ	5.1118579 sec
RG	406.4
DW	78.000 usec
DE	4.50 usec
TE	298.0 K
D1	0.10000000 sec
MCREST	0.00000000 sec
MCWRK	0.01500000 sec

======== CHANNEL f1 ========

NUC1	1H
P1	12.00 usec
PL1	-0.90 dB
SFO1	400.1328009 MHz

F2 - Processing parameters

SI	65536
SF	400.1300207 MHz
WDW	EM
SSB	0
LB	0.30 Hz
GB	0
PC	2.00

1D NMR plot parameters

CX	22.80 cm
CY	15.00 cm
F1P	9.000 ppm
F1	3601.17 Hz
F2P	-0.500 ppm
F2	-200.06 Hz
PPMCM	0.41667 ppm/cm
HZCM	166.72086 Hz/cm

1H spectrum

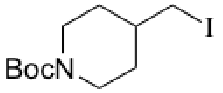

21

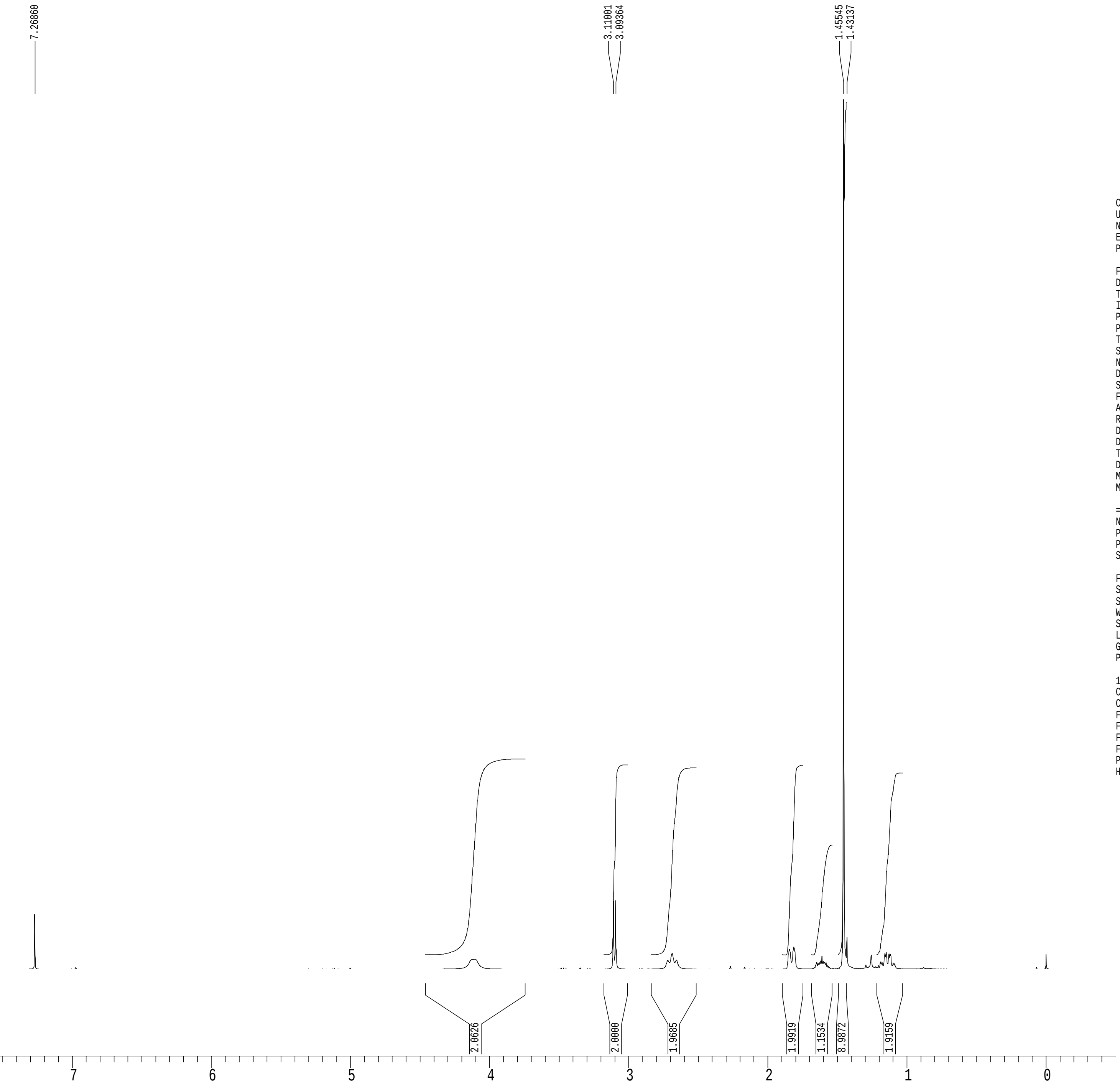

Current Data Parameters
USER nhirbawi
NAME NH-2-160-column
EXPNO 1
PROCNO 1

F2 - Acquisition Parameters
Date_ 20220126
Time 17.36
INSTRUM drx400
PROBHD 5 mm QNP H/F/P
PULPROG zg30
TD 65536
SOLVENT CDCl3T
NS 8
DS 2
SWH 6410.256 Hz
FIDRES 0.097813 Hz
AQ 5.1118579 sec
RG 143.7
DW 78.000 usec
DE 4.50 usec
TE 298.0 K
D1 0.10000000 sec
MCREST 0.00000000 sec
MCWRK 0.01500000 sec

======== CHANNEL f1 ========
NUC1 1H
P1 12.00 usec
PL1 -0.90 dB
SFO1 400.1328009 MHz

F2 - Processing parameters
SI 65536
SF 400.1300178 MHz
WDW EM
SSB 0
LB 0.30 Hz
GB 0
PC 2.00

1D NMR plot parameters
CX 22.80 cm
CY 15.00 cm
F1P 9.000 ppm
F1 3601.17 Hz
F2P -0.500 ppm
F2 -200.06 Hz
PPMCM 0.41667 ppm/cm
HZCM 166.72084 Hz/cm

1H spectrum

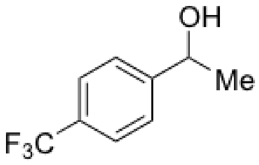

62

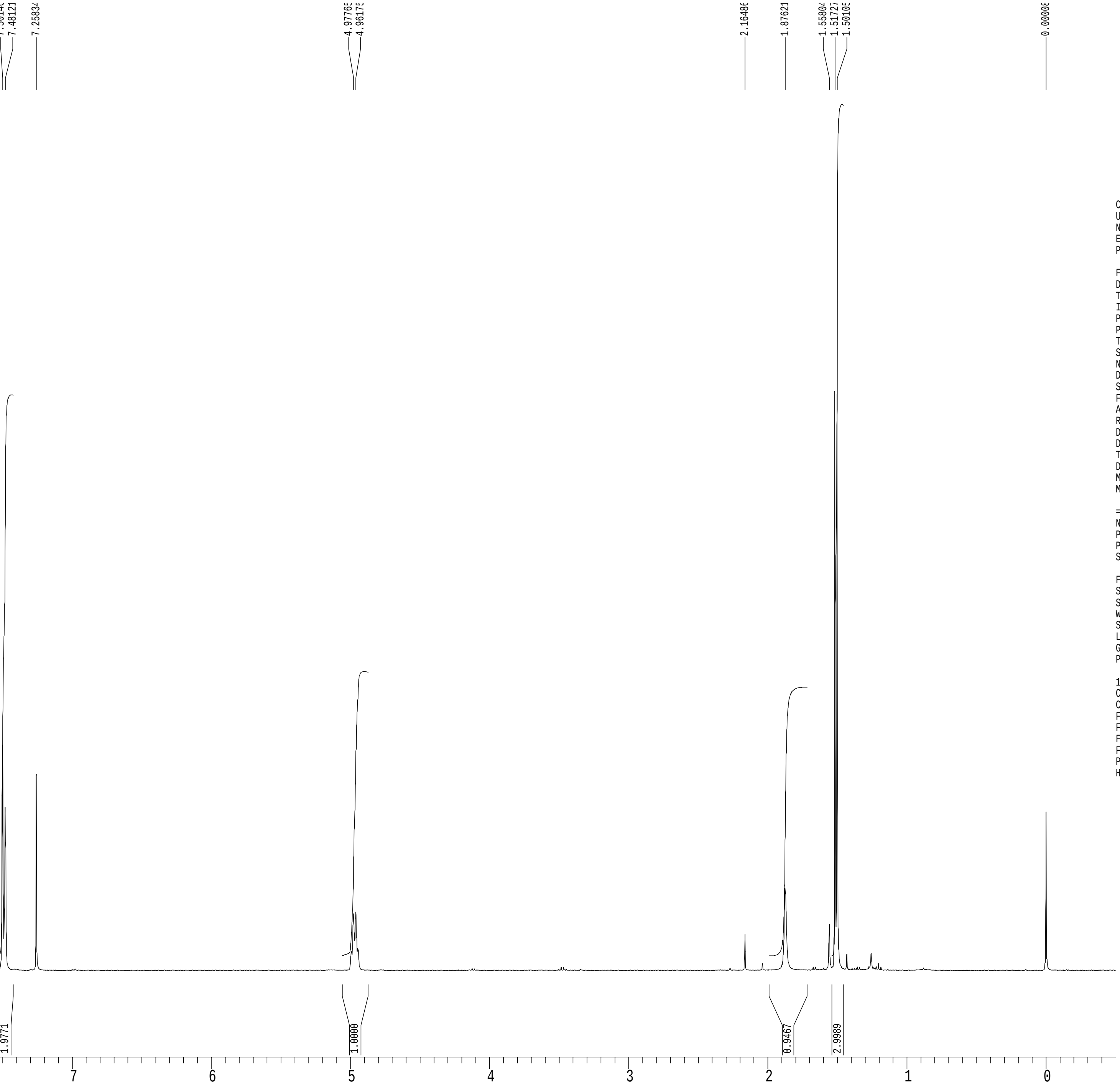


```
Current Data Parameters
USER            nhirbawi
NAME        NH-2-87-isco
EXPNO                  1
PROCNO                 1

F2 - Acquisition Parameters
Date_           20211025
Time               14.30
INSTRUM           drx400
PROBHD   5 mm Multinucl
PULPROG             zg30
TD                 65536
SOLVENT           CDCl3T
NS                     8
DS                     2
SWH             6410.256 Hz
FIDRES          0.097813 Hz
AQ             5.1118579 sec
RG                 645.1
DW                78.000 usec
DE                  4.50 usec
TE                 297.9 K
D1            0.10000000 sec
MCREST        0.00000000 sec
MCWRK         0.01500000 sec

======== CHANNEL f1 ========
NUC1                  1H
P1                 12.00 usec
PL1                -1.10 dB
SFO1         400.1328009 MHz

F2 - Processing parameters
SI                 65536
SF           400.1300221 MHz
WDW                   EM
SSB                    0
LB                  0.30 Hz
GB                     0
PC                  2.00

1D NMR plot parameters
CX                 22.80 cm
CY                 10.00 cm
F1P                9.000 ppm
F1               3601.17 Hz
F2P               -0.500 ppm
F2               -200.06 Hz
PPMCM            0.41667 ppm/cm
HZCM           166.72086 Hz/cm
```

13C spectrum with 1H decoupling

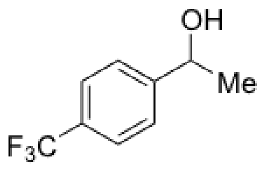

62

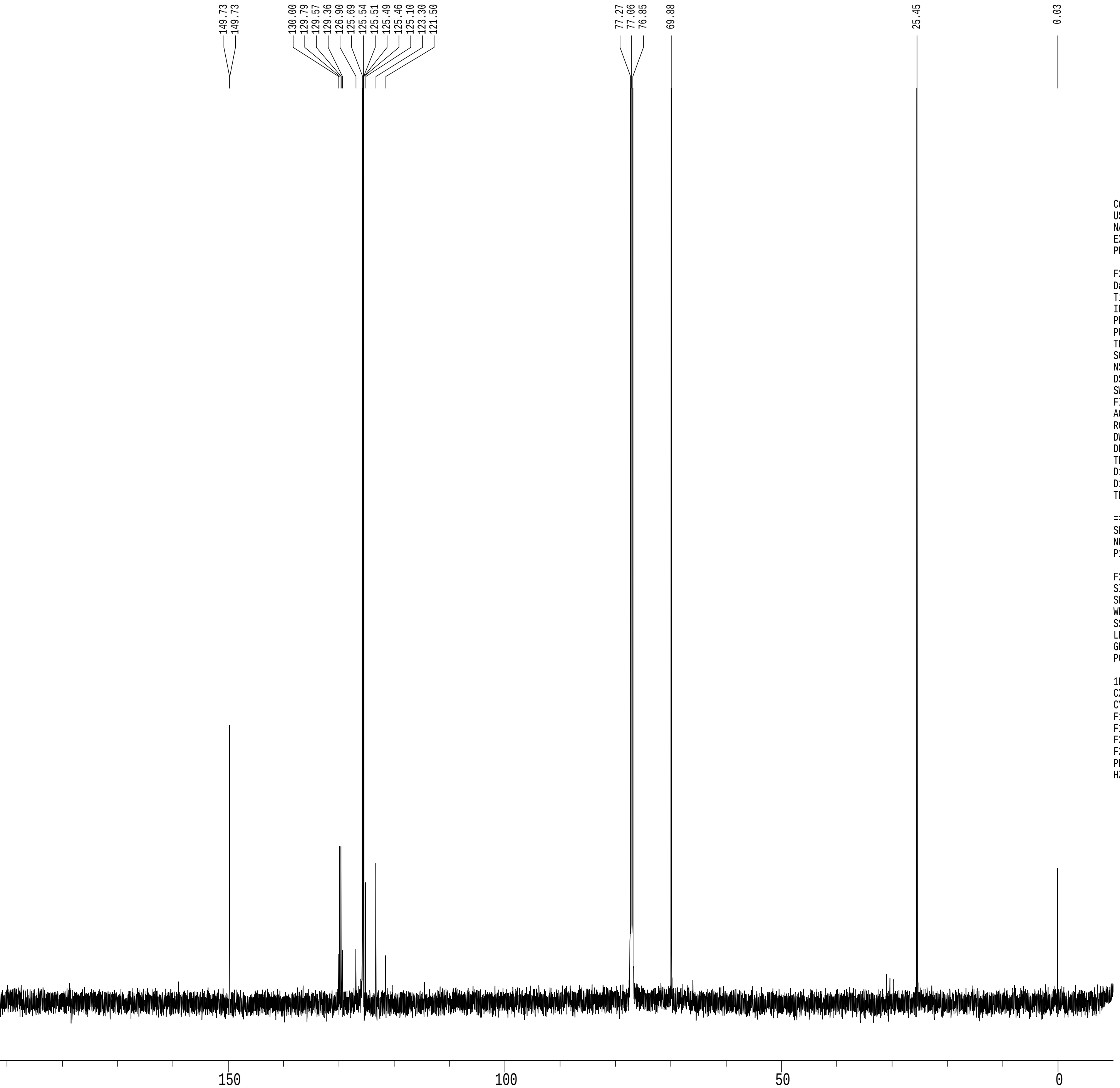


```
Current Data Parameters
USER            nhirbawi
NAME     NH-2-87-600-13C
EXPNO                  1
PROCNO                 1

F2 - Acquisition Parameters
Date_           20211106
Time               16.35
INSTRUM            av600
PROBHD   5 mm CPBBO BB-
PULPROG           zgdc30
TD                 65536
SOLVENT           CDCl3T
NS                   302
DS                     4
SWH           36231.883 Hz
FIDRES         0.552855 Hz
AQ            0.9044468 sec
RG                  2050
DW                13.800 usec
DE                 19.63 usec
TE                 298.0 K
D1            0.40000001 sec
D11           0.03000000 sec
TD0                    1

======== CHANNEL f1 ========
SFO1         150.9194080 MHz
NUC1                 13C
P1                 10.10 usec

F2 - Processing parameters
SI                 65536
SF           150.9028085 MHz
WDW                   EM
SSB                    0
LB                  1.00 Hz
GB                     0
PC                  1.00

1D NMR plot parameters
CX                 22.80 cm
CY                100.00 cm
F1P              230.000 ppm
F1              34707.64 Hz
F2P              -10.000 ppm
F2              -1509.03 Hz
PPMCM           10.52632 ppm/cm
HZCM          1588.45056 Hz/cm
```

19F spectrum

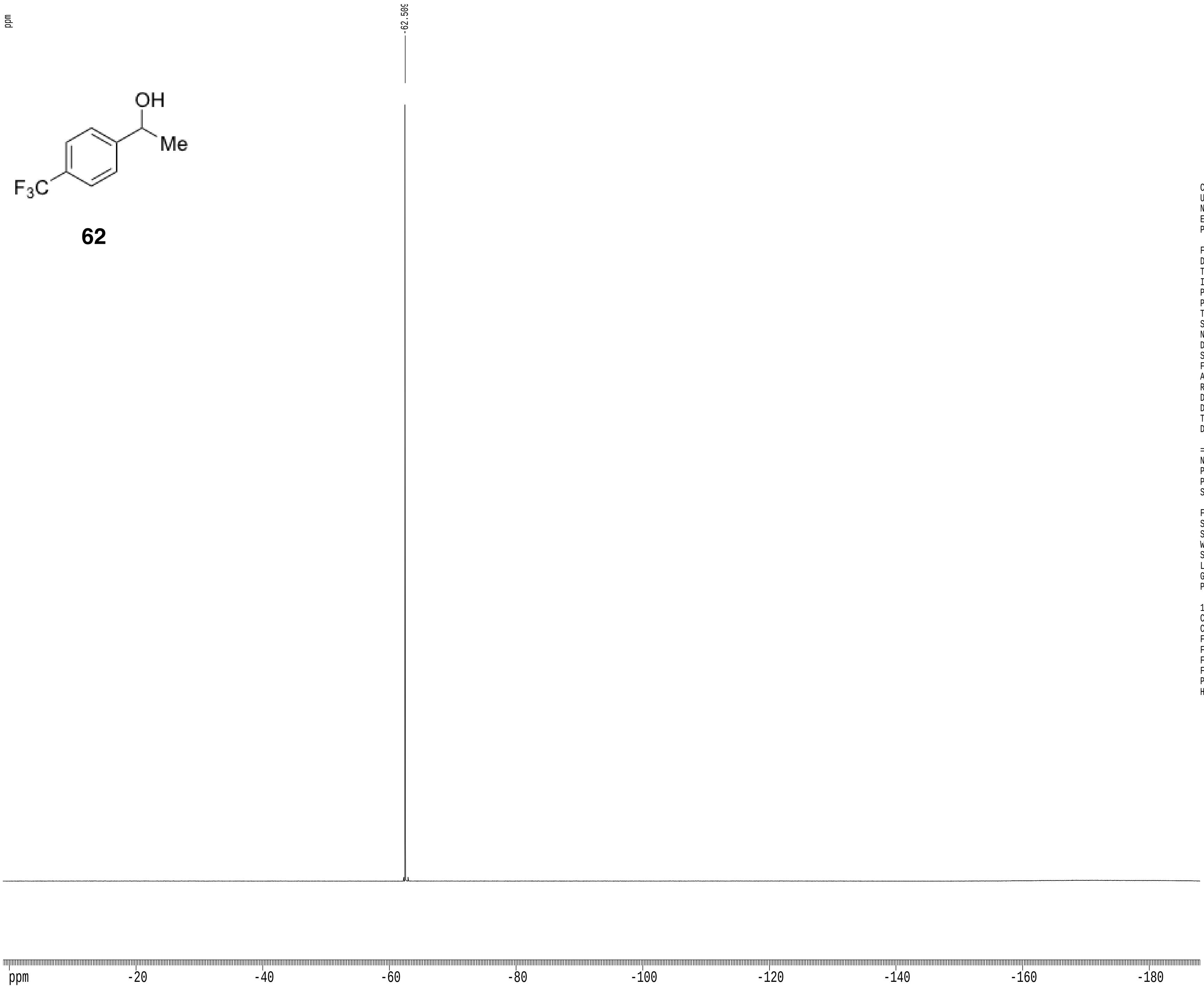


```
Current Data Parameters
USER            nhirbawi
NAME         NH-2-87-19F
EXPNO                  2
PROCNO                 1

F2 - Acquisition Parameters
Date_           20211106
Time               14.30
INSTRUM           drx400
PROBHD   5 mm QNP H/F/P
PULPROG         zgflqn30
TD                 65536
SOLVENT            CDCl3
NS                    51
DS                     2
SWH            75187.969 Hz
FIDRES          1.147277 Hz
AQ             0.4358644 sec
RG                 912.3
DW                 6.650 usec
DE                  9.46 usec
TE                 298.0 K
D1            2.00000000 sec

======== CHANNEL f1 ========
NUC1                 19F
P1                 21.75 usec
PL1                -6.00 dB
SFO1         376.4646491 MHz

F2 - Processing parameters
SI                 65536
SF           376.4983866 MHz
WDW                   EM
SSB                    0
LB                  1.00 Hz
GB                     0
PC                  1.00

1D NMR plot parameters
CX                 22.80 cm
CY                 15.00 cm
F1P                1.000 ppm
F1                376.50 Hz
F2P             -188.000 ppm
F2             -70781.70 Hz
PPMCM            8.28947 ppm/cm
HZCM          3120.97363 Hz/cm
```

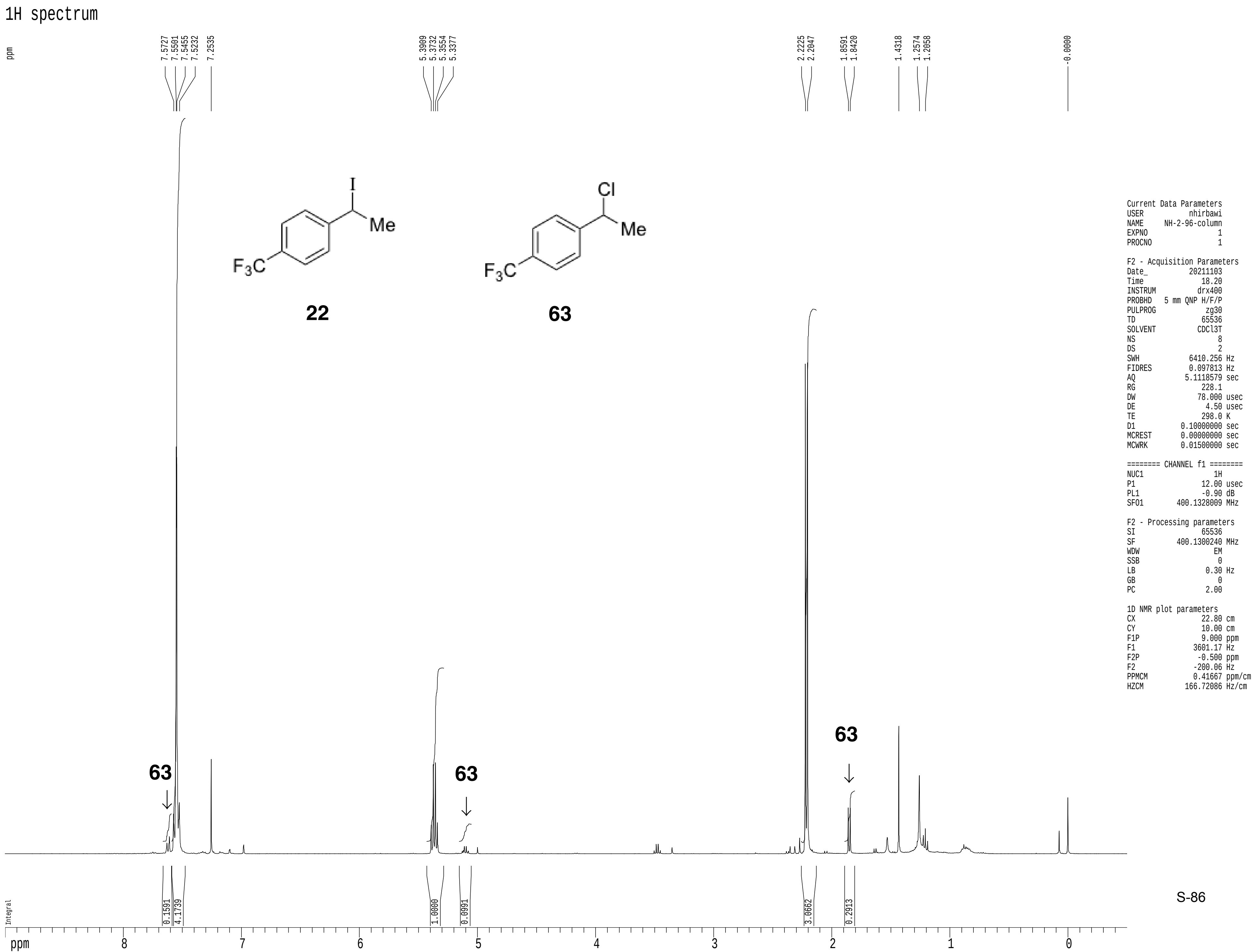
1H spectrum
ppm
7.5727
7.5501
7.5455
7.5232
7.2535
5.3909
5.3732
5.3554
5.3377
2.2225
2.2047
1.8591
1.8420
1.4318
1.2574
1.2058
-0.0000
I
Me
F3C
22
Cl
Me
F3C
63
63
63
63
Integral
0.1591
4.1739
1.0000
0.0991
3.0662
0.2913
ppm
8
7
6
5
4
3
2
1
0
Current Data Parameters
USER nhirbawi
NAME NH-2-96-column
EXPNO 1
PROCNO 1
F2 - Acquisition Parameters
Date_ 20211103
Time 18.20
INSTRUM drx400
PROBHD 5 mm QNP H/F/P
PULPROG zg30
TD 65536
SOLVENT CDCl3T
NS 8
DS 2
SWH 6410.256 Hz
FIDRES 0.097813 Hz
AQ 5.1118579 sec
RG 228.1
DW 78.000 usec
DE 4.50 usec
TE 298.0 K
D1 0.10000000 sec
MCREST 0.00000000 sec
MCWRK 0.01500000 sec
======== CHANNEL f1 ========
NUC1 1H
P1 12.00 usec
PL1 -0.90 dB
SFO1 400.1328009 MHz
F2 - Processing parameters
SI 65536
SF 400.1300240 MHz
WDW EM
SSB 0
LB 0.30 Hz
GB 0
PC 2.00
1D NMR plot parameters
CX 22.80 cm
CY 10.00 cm
F1P 9.000 ppm
F1 3601.17 Hz
F2P -0.500 ppm
F2 -200.06 Hz
PPMCM 0.41667 ppm/cm
HZCM 166.72086 Hz/cm

19F spectrum

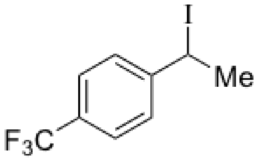

22

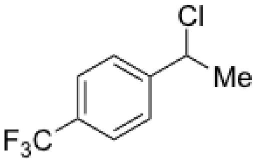

63

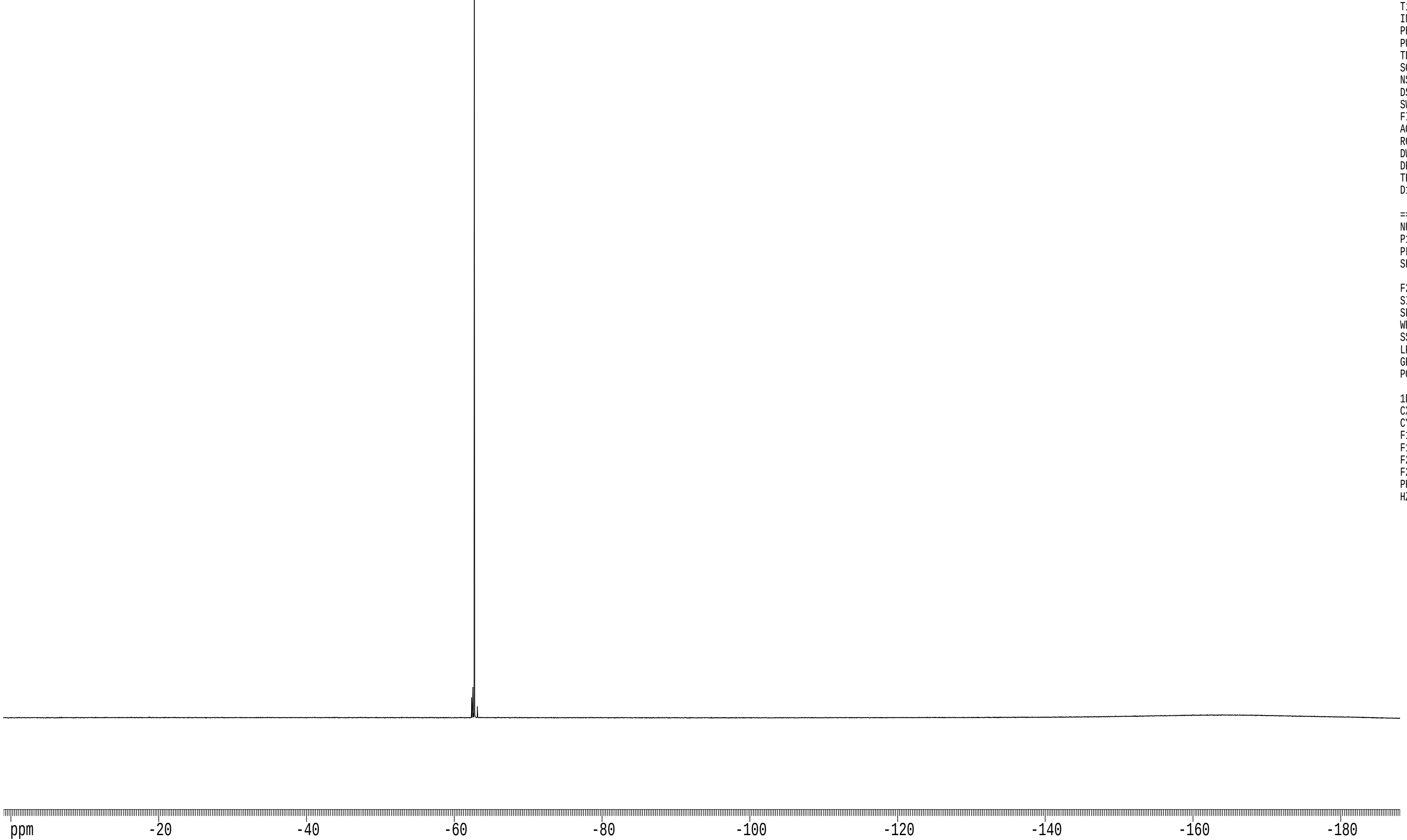


```
Current Data Parameters
USER          nhirbawi
NAME      NH-2-96-HandF
EXPNO                 2
PROCNO                1

F2 - Acquisition Parameters
Date_          20220514
Time              13.46
INSTRUM          drx400
PROBHD   5 mm QNP H/F/P
PULPROG        zgflqn30
TD                65536
SOLVENT           CDCl3
NS                   84
DS                    2
SWH           75187.969 Hz
FIDRES         1.147277 Hz
AQ            0.4358644 sec
RG               1625.5
DW                6.650 usec
DE                 9.46 usec
TE                298.0 K
D1           2.00000000 sec

======== CHANNEL f1 ========
NUC1                19F
P1                21.75 usec
PL1               -6.00 dB
SFO1        376.4646491 MHz

F2 - Processing parameters
SI                65536
SF          376.4983865 MHz
WDW                  EM
SSB                   0
LB                 1.00 Hz
GB                    0
PC                 1.00

1D NMR plot parameters
CX                22.80 cm
CY                15.00 cm
F1P               1.000 ppm
F1               376.50 Hz
F2P            -188.000 ppm
F2            -70781.70 Hz
PPMCM           8.28947 ppm/cm
HZCM         3120.97363 Hz/cm
```

1H spectrum

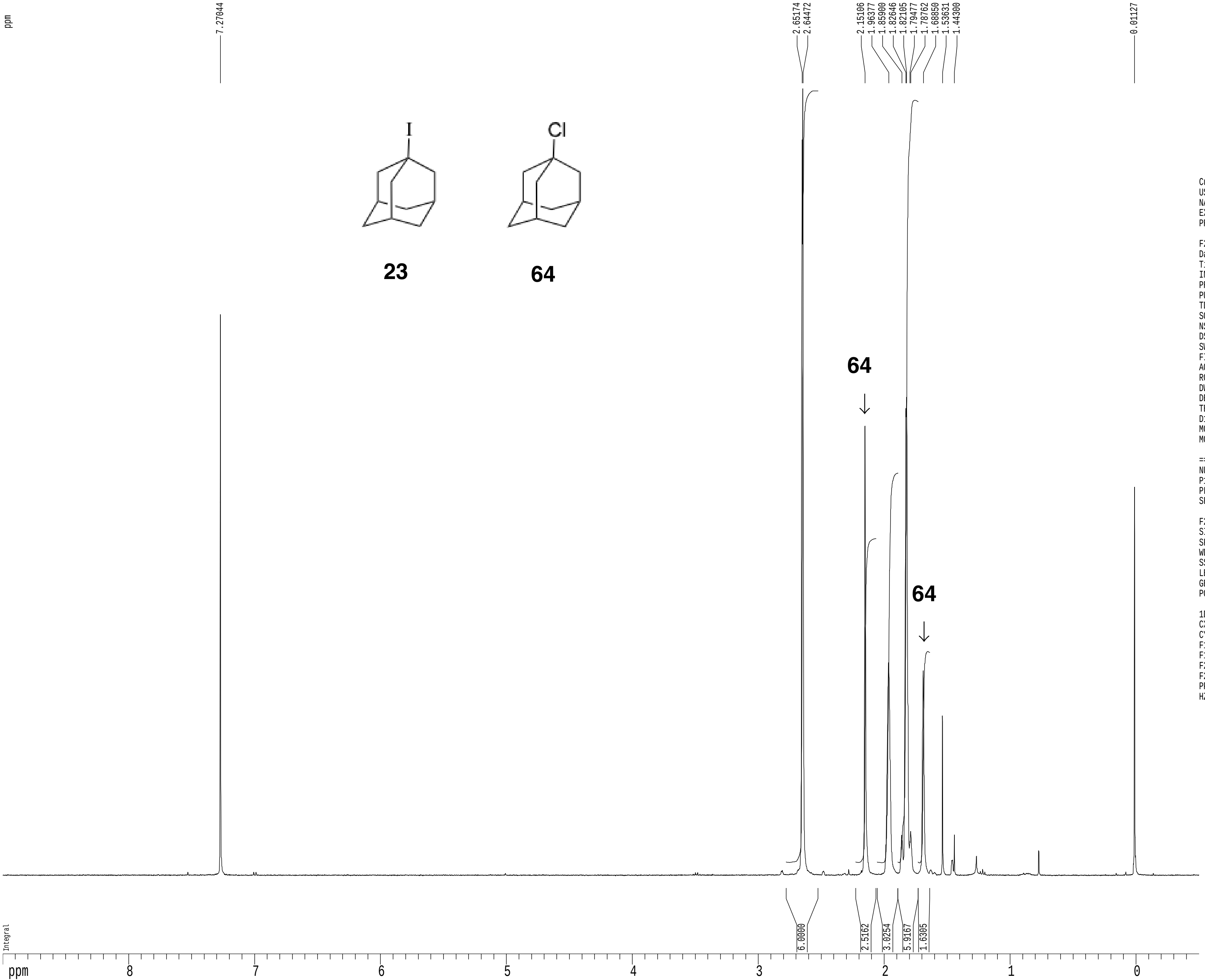


```
Current Data Parameters
USER            nhirbawi
NAME         NH-2-95-ISCO
EXPNO                  1
PROCNO                 1

F2 - Acquisition Parameters
Date_           20211103
Time               18.16
INSTRUM           drx400
PROBHD   5 mm QNP H/F/P
PULPROG             zg30
TD                 65536
SOLVENT           CDCl3T
NS                     8
DS                     2
SWH             6410.256 Hz
FIDRES          0.097813 Hz
AQ             5.1118579 sec
RG                 456.1
DW                78.000 usec
DE                  4.50 usec
TE                 298.0 K
D1            0.10000000 sec
MCREST        0.00000000 sec
MCWRK         0.01500000 sec

======== CHANNEL f1 ========
NUC1                  1H
P1                 12.00 usec
PL1                -0.90 dB
SFO1         400.1328009 MHz

F2 - Processing parameters
SI                 65536
SF           400.1300175 MHz
WDW                   EM
SSB                    0
LB                  0.30 Hz
GB                     0
PC                  2.00

1D NMR plot parameters
CX                 22.80 cm
CY                 15.00 cm
F1P                9.000 ppm
F1               3601.17 Hz
F2P               -0.500 ppm
F2               -200.06 Hz
PPMCM            0.41667 ppm/cm
HZCM           166.72084 Hz/cm
```

1H spectrum

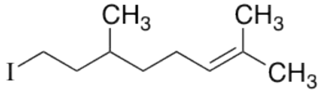

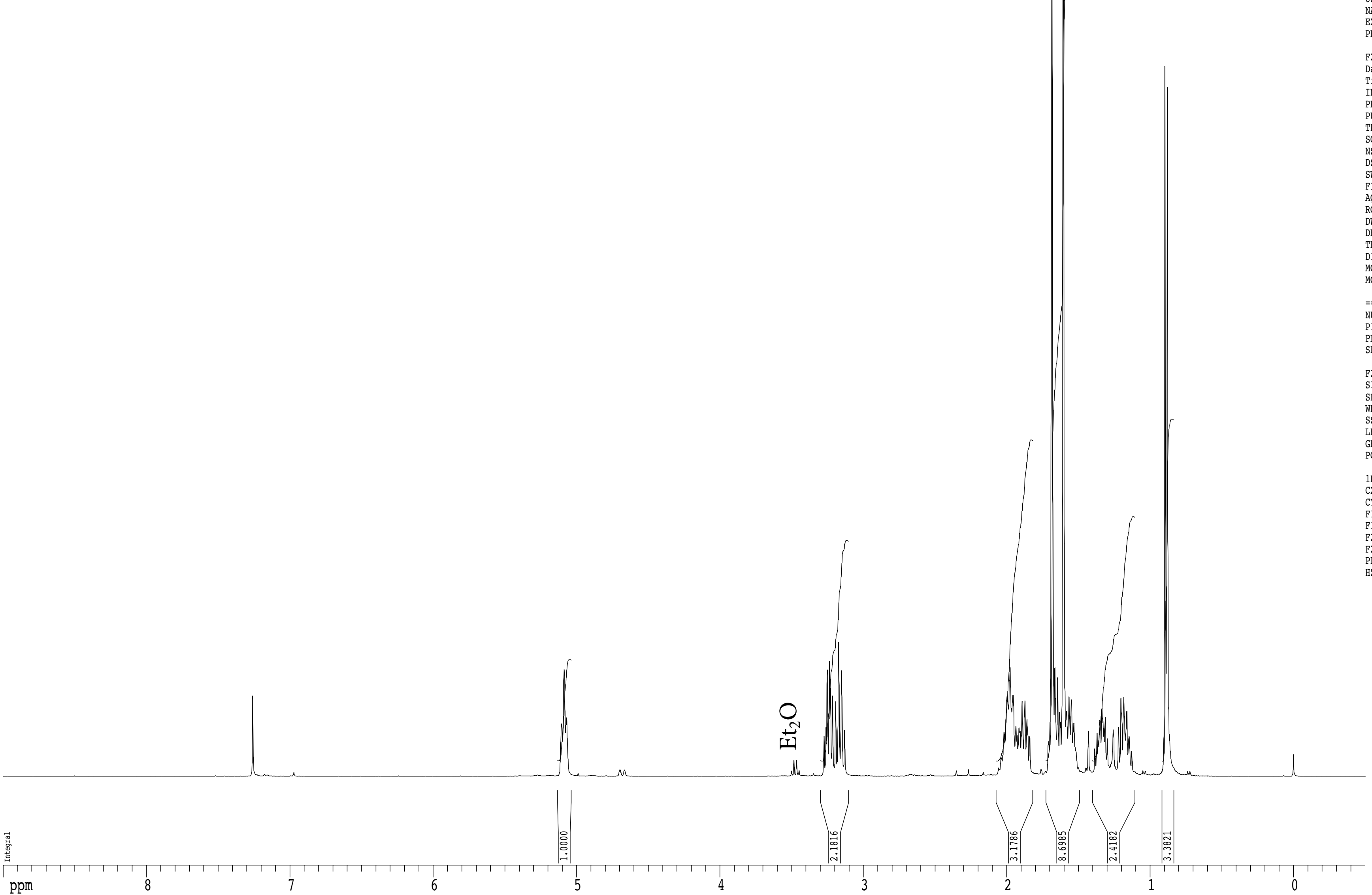

Current Data Parameters

USER	linpc2
NAME	pcl-3-048
EXPNO	1
PROCNO	1

F2 - Acquisition Parameters

Date_	20220217
Time	15.14
INSTRUM	drx400
PROBHD	5 mm QNP H/F/P
PULPROG	zg30
TD	38460
SOLVENT	CDCl3
NS	8
DS	2
SWH	6410.256 Hz
FIDRES	0.166673 Hz
AQ	2.9999299 sec
RG	71.8
DW	78.000 usec
DE	4.50 usec
TE	298.0 K
D1	0.10000000 sec
MCREST	0.00000000 sec
MCWRK	0.01500000 sec

======== CHANNEL f1 ========

NUC1	1H
P1	12.00 usec
PL1	-0.90 dB
SFO1	400.1328009 MHz

F2 - Processing parameters

SI	65536
SF	400.1300221 MHz
WDW	EM
SSB	0
LB	0.30 Hz
GB	0
PC	2.00

1D NMR plot parameters

CX	22.80 cm
CY	15.00 cm
F1P	9.000 ppm
F1	3601.17 Hz
F2P	-0.500 ppm
F2	-200.06 Hz
PPMCM	0.41667 ppm/cm
HZCM	166.72086 Hz/cm

1H spectrum

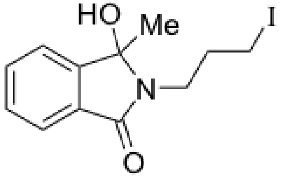

25

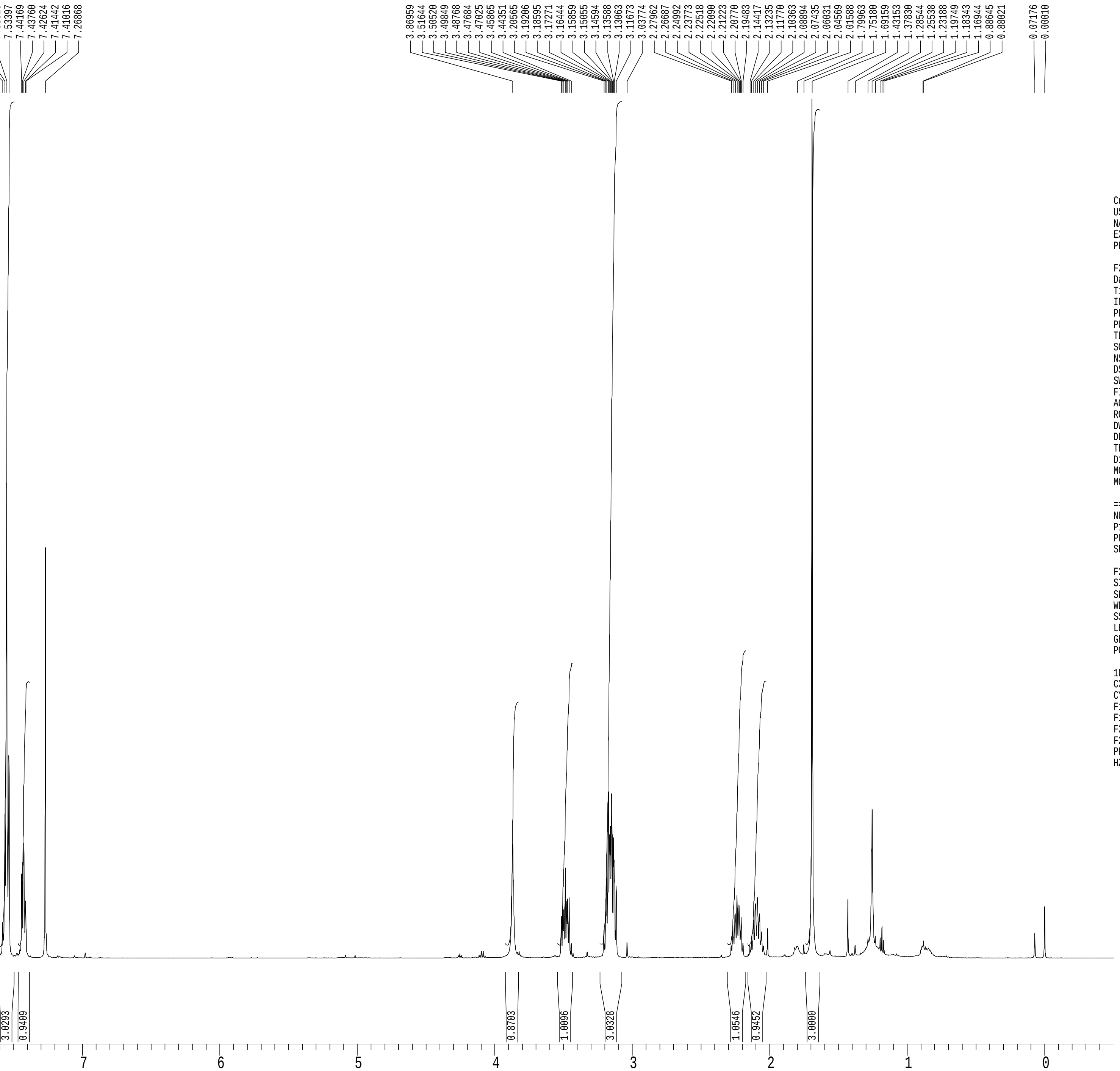


```
Current Data Parameters
USER            nhirbawi
NAME      NH-2-183-HCCOSY
EXPNO                  1
PROCNO                 1

F2 - Acquisition Parameters
Date_           20220225
Time               16.34
INSTRUM          cryo500
PROBHD   5 mm CPTCI 1H-
PULPROG             zg30
TD                 81728
SOLVENT           CDCl3T
NS                     8
DS                     2
SWH             8012.820 Hz
FIDRES          0.098043 Hz
AQ             5.0998774 sec
RG                   5.7
DW                62.400 usec
DE                  6.00 usec
TE                 298.0 K
D1            0.10000000 sec
MCREST        0.00000000 sec
MCWRK         0.01500000 sec

======== CHANNEL f1 ========
NUC1                  1H
P1                  9.75 usec
PL1                 1.60 dB
SFO1         500.2235015 MHz

F2 - Processing parameters
SI                 65536
SF           500.2200265 MHz
WDW                   EM
SSB                    0
LB                  0.30 Hz
GB                     0
PC                  1.00

1D NMR plot parameters
CX                 22.80 cm
CY                 15.00 cm
F1P                9.000 ppm
F1               4501.98 Hz
F2P               -0.500 ppm
F2               -250.11 Hz
PPMCM            0.41667 ppm/cm
HZCM           208.42502 Hz/cm
```

Z-restored spin-echo 13C spectrum with 1H decoupling

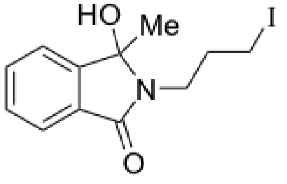

25

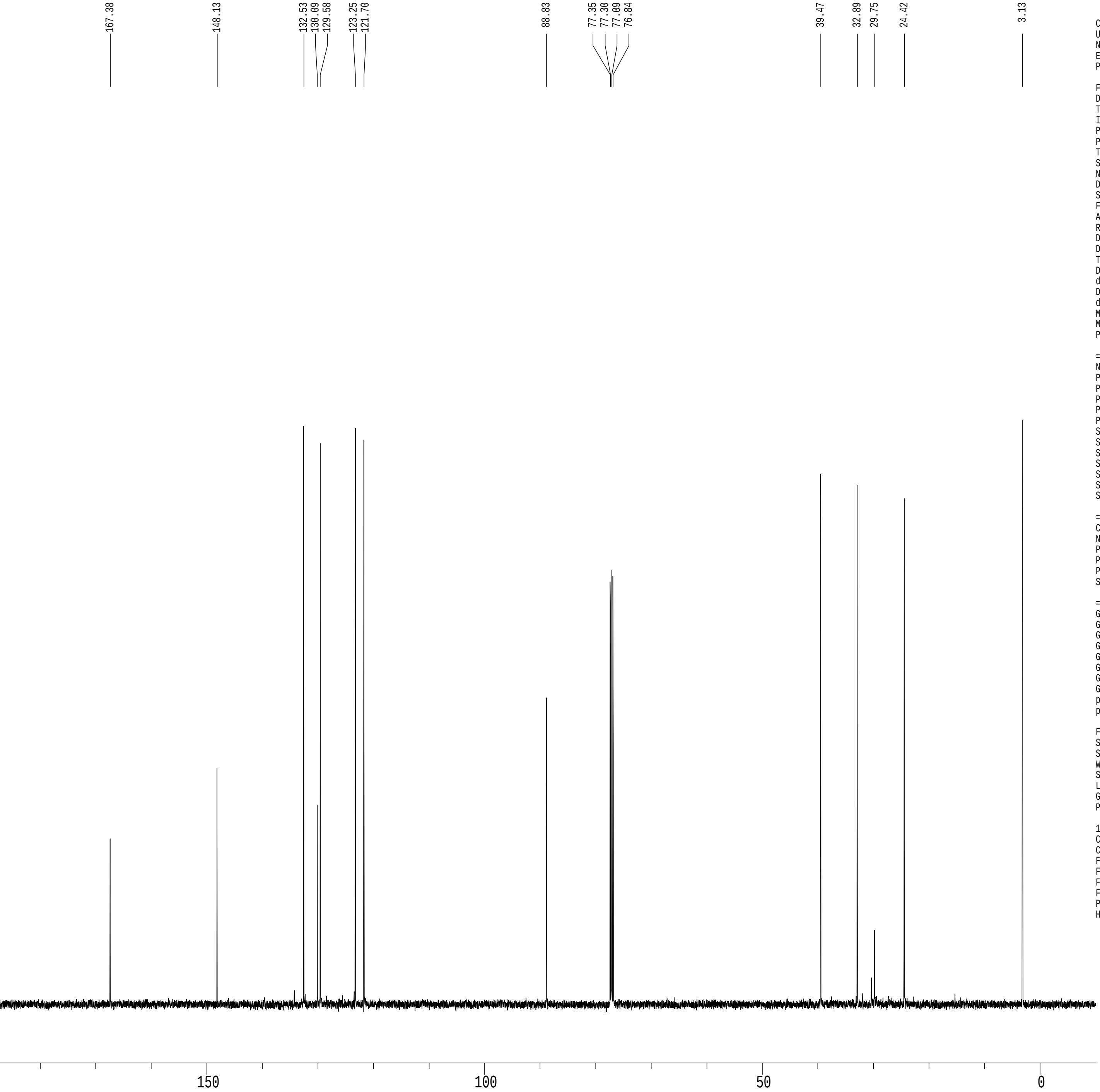


```
Current Data Parameters
USER          nhirbawi
NAME     NH-2-183-HCCOSY
EXPNO                2
PROCNO               1

F2 - Acquisition Parameters
Date_         20220225
Time             16.37
INSTRUM        cryo500
PROBHD   5 mm CPTCI 1H-
PULPROG  SpinEchopg30gp2.prd
TD               65536
SOLVENT          CDCl3
NS                 353
DS                  16
SWH          30303.031 Hz
FIDRES        0.462388 Hz
AQ           1.0813940 sec
RG                6502
DW              16.500 usec
DE                6.00 usec
TE               298.0 K
D1          0.25000000 sec
d11         0.03000000 sec
D16         0.00020000 sec
d17         0.00019600 sec
MCREST      0.00000000 sec
MCWRK       0.01500000 sec
P2               37.70 usec

======== CHANNEL f1 ========
NUC1               13C
P1               18.85 usec
P12            2000.00 usec
P20             500.00 usec
PL0             120.00 dB
PL1              -1.00 dB
SFO1       125.7942548 MHz
SP2               1.55 dB
SP4               1.55 dB
SPNAM2      Crp60comp.4
SPNAM4   Crp60,0.5,20.1
SPOFF2            0.00 Hz
SPOFF4            0.00 Hz

======== CHANNEL f2 ========
CPDPRG2        waltz16
NUC2                1H
PCPD2           100.00 usec
PL2               1.60 dB
PL12             22.00 dB
SFO2       500.2225011 MHz

====== GRADIENT CHANNEL =====
GPNAM1        SINE.100
GPNAM2        SINE.100
GPX1              0.00 %
GPX2              0.00 %
GPY1              0.00 %
GPY2              0.00 %
GPZ1             30.00 %
GPZ2             50.00 %
p15             500.00 usec
p16            1000.00 usec

F2 - Processing parameters
SI               65536
SF         125.7804190 MHz
WDW                 EM
SSB                  0
LB                1.00 Hz
GB                   0
PC                2.00

1D NMR plot parameters
CX               22.80 cm
CY               10.00 cm
F1P            230.000 ppm
F1            28929.50 Hz
F2P            -10.000 ppm
F2            -1257.80 Hz
PPMCM         10.52632 ppm/cm
HZCM        1324.00439 Hz/cm
```

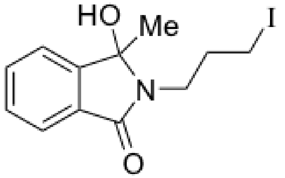

gcosy60

25

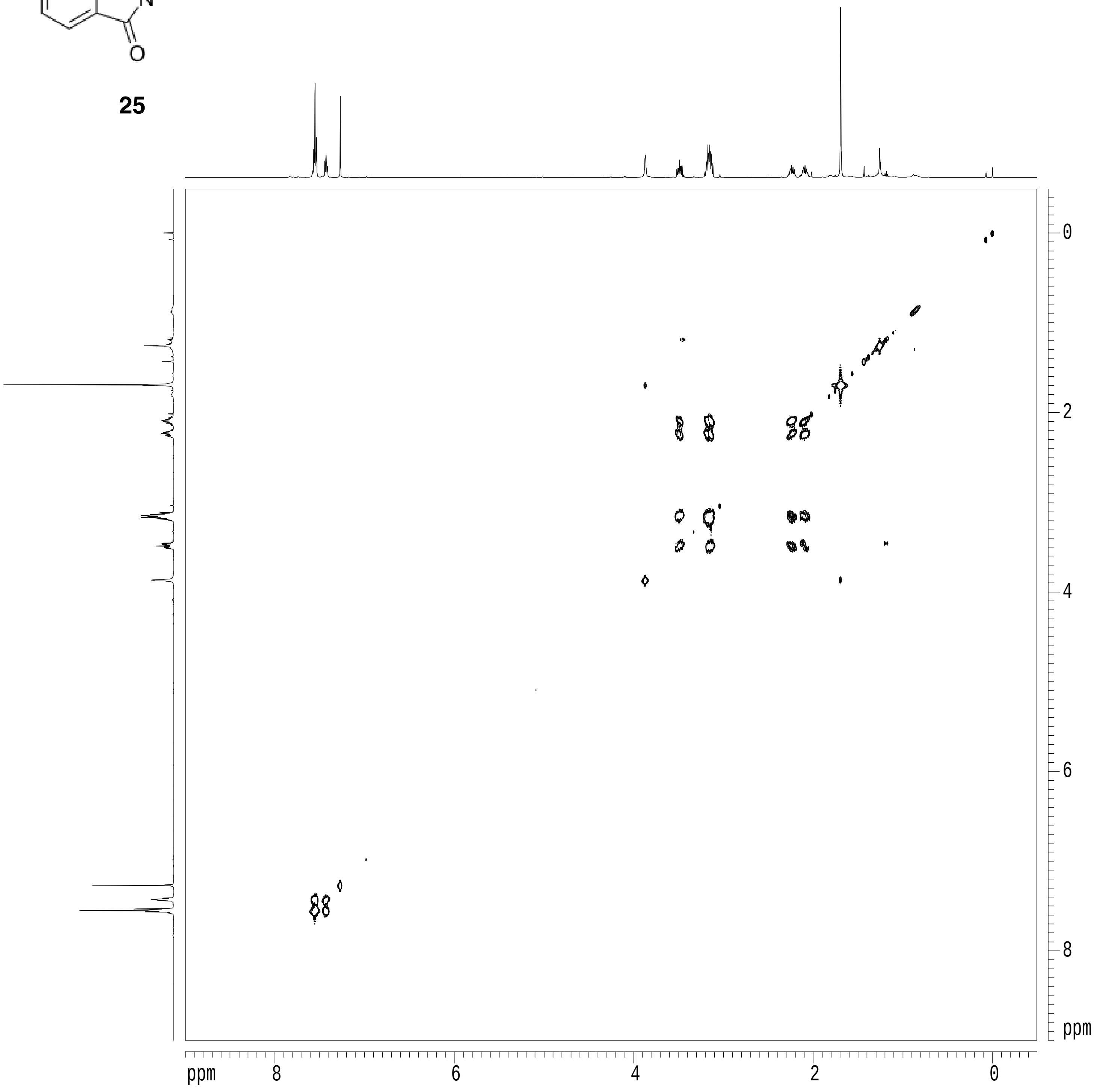

Current Data Parameters
USER nhirbawi
NAME NH-2-183-HCCOSY
EXPNO 3
PROCNO 1

F2 - Acquisition Parameters
Date_ 20220225
Time 16.43
INSTRUM cryo500
PROBHD 5 mm CPTCI 1H-
PULPROG cosygp60.prd
TD 2048
SOLVENT CDCl3T
NS 2
DS 16
SWH 5000.000 Hz
FIDRES 2.441406 Hz
AQ 0.2048500 sec
RG 90.5
DW 100.000 usec
DE 6.00 usec
TE 298.0 K
d0 0.00000300 sec
D1 1.00000000 sec
d13 0.00000300 sec
D16 0.00020000 sec
IN0 0.00020000 sec

======== CHANNEL f1 ========
NUC1 1H
P1 9.75 usec
PL1 1.60 dB
SFO1 500.2222774 MHz

====== GRADIENT CHANNEL =====
GPNAM1 SMSQ10.100
GPNAM2 SMSQ10.100
GPX1 0.00 %
GPX2 0.00 %
GPY1 0.00 %
GPY2 0.00 %
GPZ1 17.00 %
GPZ2 17.00 %
P16 1000.00 usec

F1 - Acquisition parameters
ND0 1
TD 175
SFO1 500.2223 MHz
FIDRES 28.571428 Hz
SW 9.996 ppm
FnMODE QF

F2 - Processing parameters
SI 1024
SF 500.2200237 MHz
WDW SINE
SSB 0
LB 0.00 Hz
GB 0
PC 1.00

F1 - Processing parameters
SI 1024
MC2 QF
SF 500.2200223 MHz
WDW SINE
SSB 0
LB 0.00 Hz
GB 0

2D NMR plot parameters
CX2 15.00 cm
CX1 15.00 cm
F2PLO 9.000 ppm
F2LO 4501.98 Hz
F2PHI -0.493 ppm
F2HI -246.38 Hz
F1PLO 9.000 ppm
F1LO 4501.98 Hz
F1PHI -0.490 ppm
F1HI -24[illegible]
F2PPMCM 0.63284 ppm/cm
F2HZCM 316.55725 Hz/cm
F1PPMCM 0.63264 ppm/cm
F1HZCM 316.46097 Hz/cm

1H spectrum

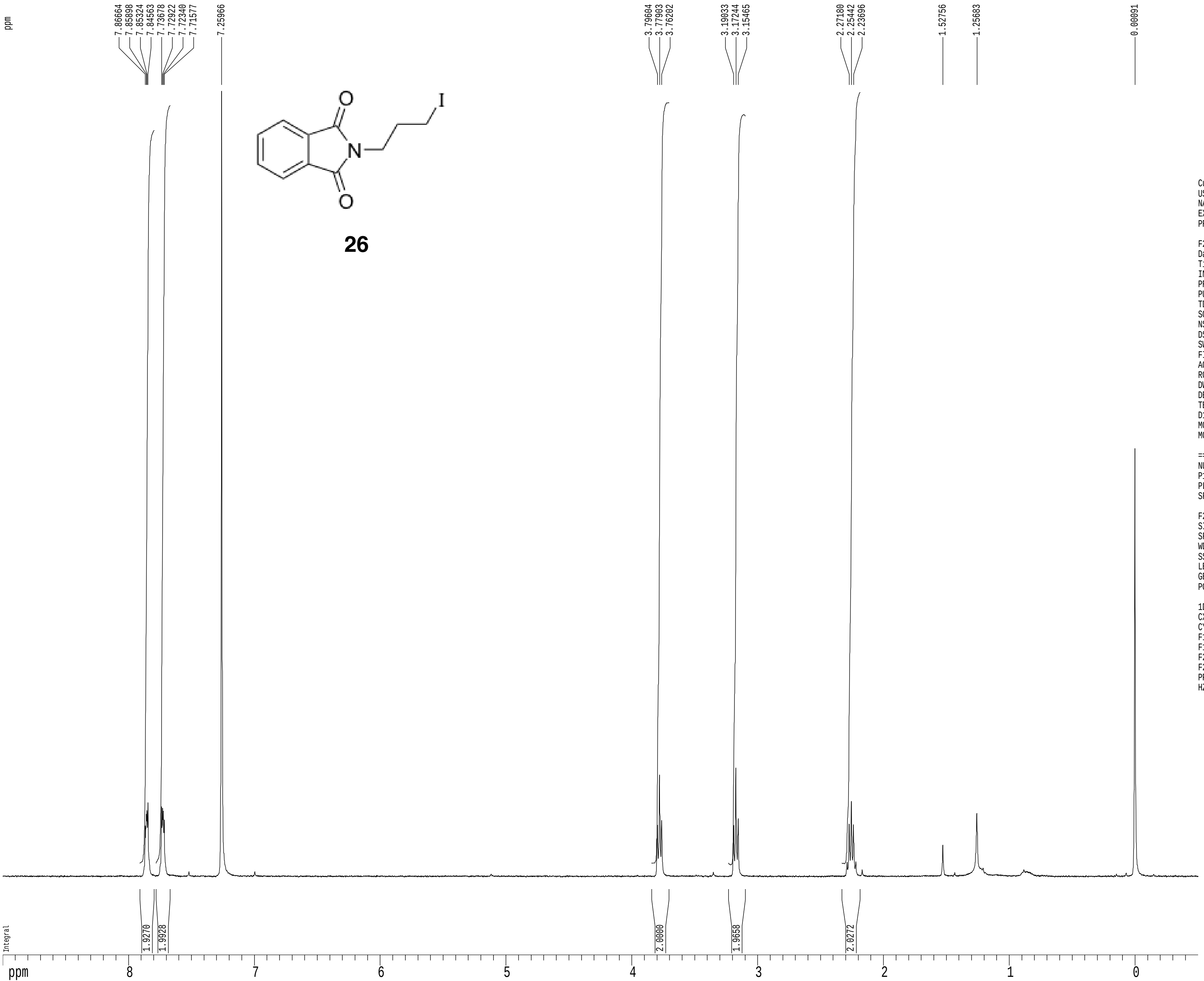


```
Current Data Parameters
USER            nhirbawi
NAME     NH-3-11-frac24-33
EXPNO                    1
PROCNO                   1

F2 - Acquisition Parameters
Date_           20220301
Time               14.15
INSTRUM           drx400
PROBHD   5 mm QNP H/F/P
PULPROG             zg30
TD                 65536
SOLVENT           CDCl3T
NS                     8
DS                     2
SWH             6410.256 Hz
FIDRES          0.097813 Hz
AQ             5.1118579 sec
RG                1149.4
DW                78.000 usec
DE                  4.50 usec
TE                 298.0 K
D1            0.10000000 sec
MCREST        0.00000000 sec
MCWRK         0.01500000 sec

======== CHANNEL f1 ========
NUC1                  1H
P1                 12.00 usec
PL1                -0.90 dB
SFO1         400.1328009 MHz

F2 - Processing parameters
SI                 65536
SF           400.1300216 MHz
WDW                   EM
SSB                    0
LB                  0.30 Hz
GB                     0
PC                  2.00

1D NMR plot parameters
CX                 22.80 cm
CY                 15.00 cm
F1P                9.000 ppm
F1               3601.17 Hz
F2P               -0.500 ppm
F2               -200.06 Hz
PPMCM            0.41667 ppm/cm
HZCM           166.72086 Hz/cm
```

1H spectrum

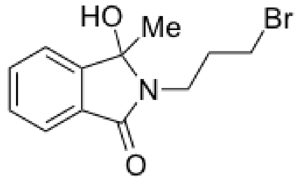

36

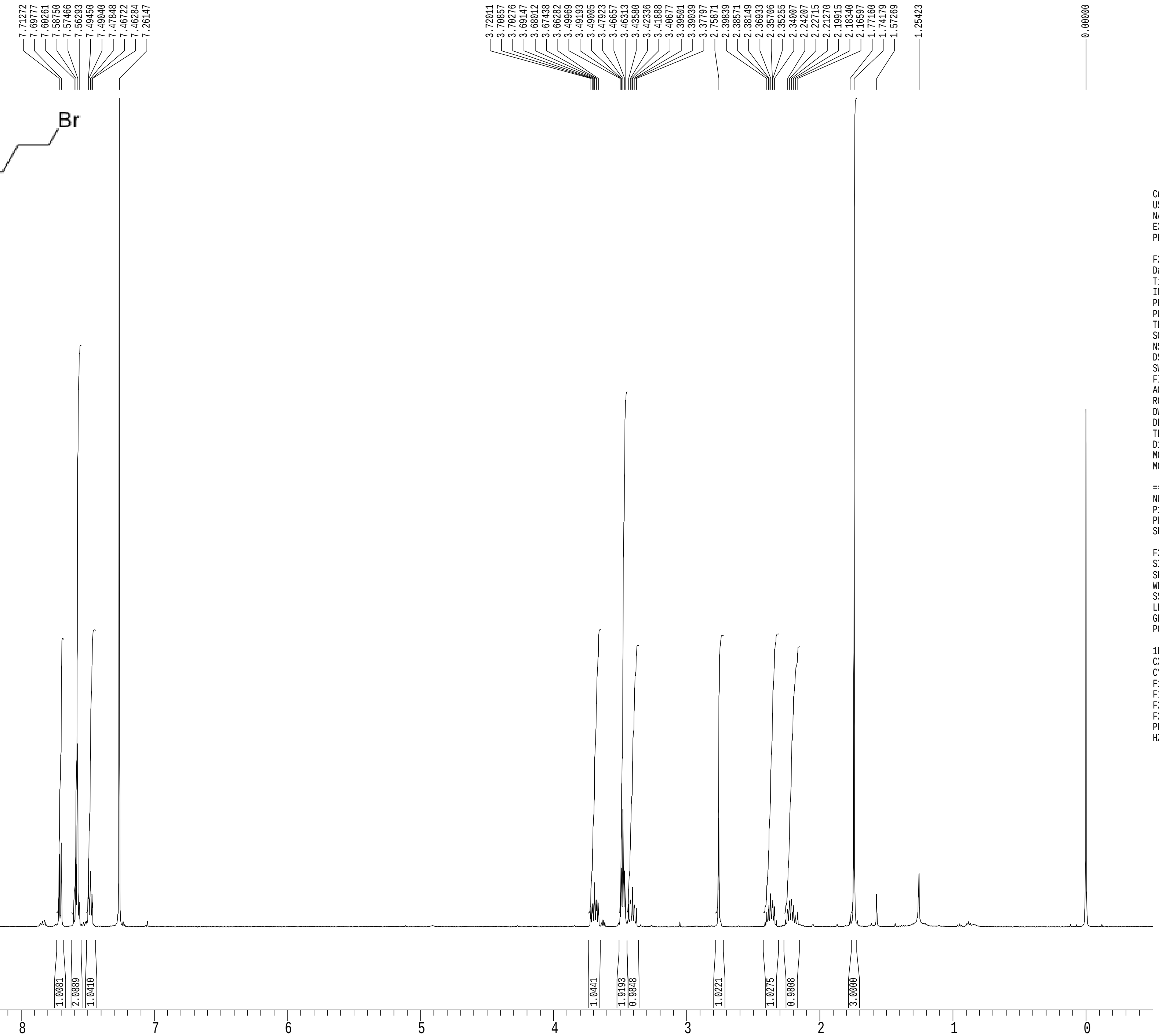

Current Data Parameters
USER nhirbawi
NAME NH-3-24-HCCOSY
EXPNO 1
PROCNO 1

F2 - Acquisition Parameters
Date_ 20220226
Time 14.45
INSTRUM cryo500
PROBHD 5 mm CPTCI 1H-
PULPROG zg30
TD 81728
SOLVENT CDCl3T
NS 8
DS 2
SWH 8012.820 Hz
FIDRES 0.098043 Hz
AQ 5.0998774 sec
RG 5.7
DW 62.400 usec
DE 6.00 usec
TE 298.0 K
D1 0.10000000 sec
MCREST 0.00000000 sec
MCWRK 0.01500000 sec

======== CHANNEL f1 ========
NUC1 1H
P1 9.75 usec
PL1 1.60 dB
SFO1 500.2235015 MHz

F2 - Processing parameters
SI 65536
SF 500.2200303 MHz
WDW EM
SSB 0
LB 0.30 Hz
GB 0
PC 1.00

1D NMR plot parameters
CX 22.80 cm
CY 15.00 cm
F1P 9.000 ppm
F1 4501.98 Hz
F2P -0.500 ppm
F2 -250.11 Hz
PPMCM 0.41667 ppm/cm
HZCM 208.42502 Hz/cm

Z-restored spin-echo 13C spectrum with 1H decoupling

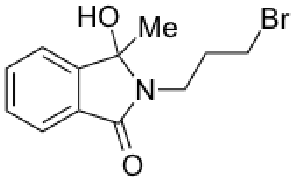

36

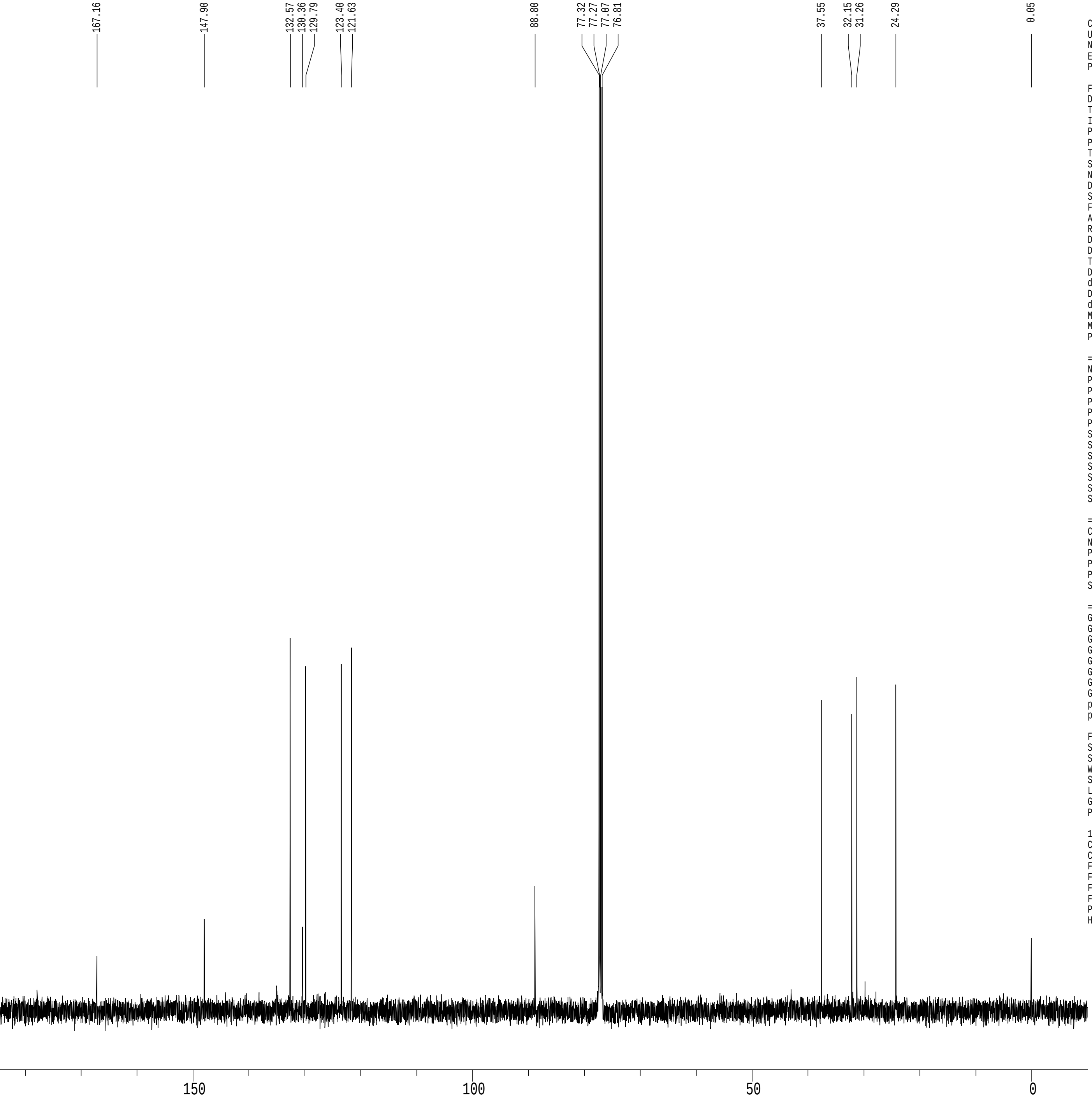

Current Data Parameters
USER nhirbawi
NAME NH-3-24-HCCOSY
EXPNO 2
PROCNO 1

F2 - Acquisition Parameters
Date_ 20220226
Time 14.48
INSTRUM cryo500
PROBHD 5 mm CPTCI 1H-
PULPROG SpinEchopg30gp2.prd
TD 65536
SOLVENT CDCl3
NS 726
DS 16
SWH 30303.031 Hz
FIDRES 0.462388 Hz
AQ 1.0813940 sec
RG 7298.2
DW 16.500 usec
DE 6.00 usec
TE 298.0 K
D1 0.25000000 sec
d11 0.03000000 sec
D16 0.00020000 sec
d17 0.00019600 sec
MCREST 0.00000000 sec
MCWRK 0.01500000 sec
P2 37.70 usec

======== CHANNEL f1 ========
NUC1 13C
P1 18.85 usec
P12 2000.00 usec
P20 500.00 usec
PL0 120.00 dB
PL1 -1.00 dB
SFO1 125.7942548 MHz
SP2 1.55 dB
SP4 1.55 dB
SPNAM2 Crp60comp.4
SPNAM4 Crp60,0.5,20.1
SPOFF2 0.00 Hz
SPOFF4 0.00 Hz

======== CHANNEL f2 ========
CPDPRG2 waltz16
NUC2 1H
PCPD2 100.00 usec
PL2 1.60 dB
PL12 22.00 dB
SFO2 500.2225011 MHz

====== GRADIENT CHANNEL =====
GPNAM1 SINE.100
GPNAM2 SINE.100
GPX1 0.00 %
GPX2 0.00 %
GPY1 0.00 %
GPY2 0.00 %
GPZ1 30.00 %
GPZ2 50.00 %
p15 500.00 usec
p16 1000.00 usec

F2 - Processing parameters
SI 65536
SF 125.7804190 MHz
WDW EM
SSB 0
LB 1.00 Hz
GB 0
PC 2.00

1D NMR plot parameters
CX 22.80 cm
CY 30.00 cm
F1P 230.000 ppm
F1 28929.50 Hz
F2P -10.000 ppm
F2 -1257.80 Hz
PPMCM 10.52632 ppm/cm
HZCM 1324.00439 Hz/cm

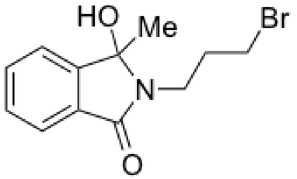

36

gcosy60

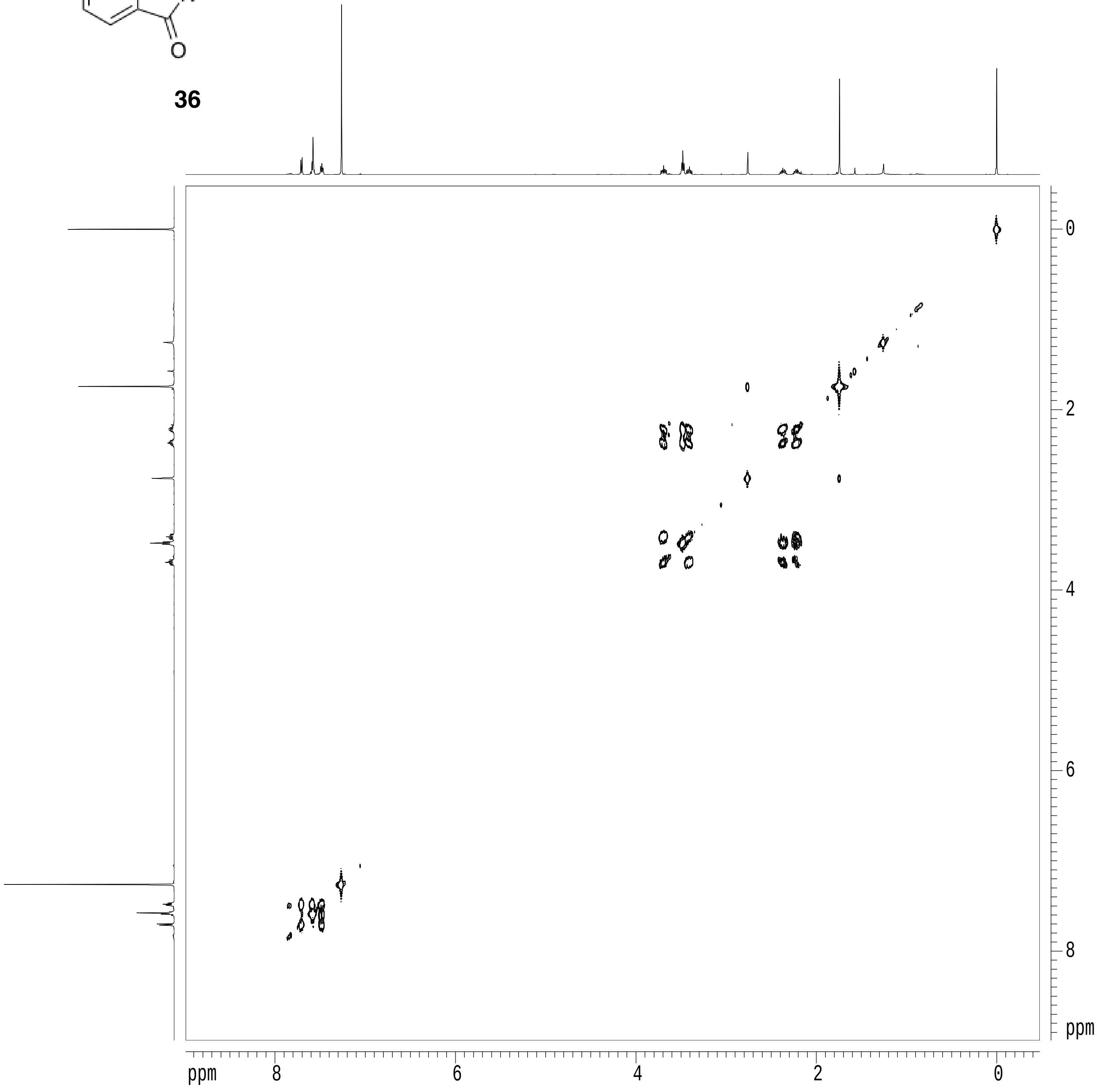


```
        Current Data Parameters
USER             nhirbawi
NAME       NH-3-24-HCCOSY
EXPNO                   3
PROCNO                  1

      F2 - Acquisition Parameters
Date_            20220226
Time                15.03
INSTRUM           cryo500
PROBHD   5 mm CPTCI 1H-
PULPROG      cosygp60.prd
TD                   2048
SOLVENT             CDCl3T
NS                      2
DS                     16
SWH             4734.849 Hz
FIDRES          2.311938 Hz
AQ             0.2163188 sec
RG                  812.7
DW                105.600 usec
DE                   6.00 usec
TE                  298.0 K
d0             0.00000300 sec
D1             1.00000000 sec
d13            0.00000300 sec
D16            0.00020000 sec
IN0            0.00021120 sec

     ======== CHANNEL f1 ========
NUC1                   1H
P1                   9.75 usec
PL1                  1.60 dB
SFO1          500.2221562 MHz

    ====== GRADIENT CHANNEL =====
GPNAM1         SMSQ10.100
GPNAM2         SMSQ10.100
GPX1                 0.00 %
GPX2                 0.00 %
GPY1                 0.00 %
GPY2                 0.00 %
GPZ1                17.00 %
GPZ2                17.00 %
P16               1000.00 usec

      F1 - Acquisition parameters
ND0                     1
TD                    154
SFO1             500.2222 MHz
FIDRES          30.745770 Hz
SW                  9.465 ppm
FnMODE                 QF

       F2 - Processing parameters
SI                   1024
SF            500.2200272 MHz
WDW                  SINE
SSB                     0
LB                   0.00 Hz
GB                      0
PC                   1.00

       F1 - Processing parameters
SI                   1024
MC2                    QF
SF            500.2200275 MHz
WDW                  SINE
SSB                     0
LB                   0.00 Hz
GB                      0

         2D NMR plot parameters
CX2                 15.00 cm
CX1                 15.00 cm
F2PLO               8.989 ppm
F2LO              4496.49 Hz
F2PHI              -0.477 ppm
F2HI              -238.36 Hz
F1PLO               8.988 ppm
F1LO              4496.10 Hz
F1PHI              -0.477 ppm
F1HI              -238[illegible]
F2PPMCM           0.63104 ppm/cm
F2HZCM          315.65659 Hz/cm
F1PPMCM           0.63104 ppm/cm
F1HZCM          315.65659 Hz/cm
```

1H spectrum

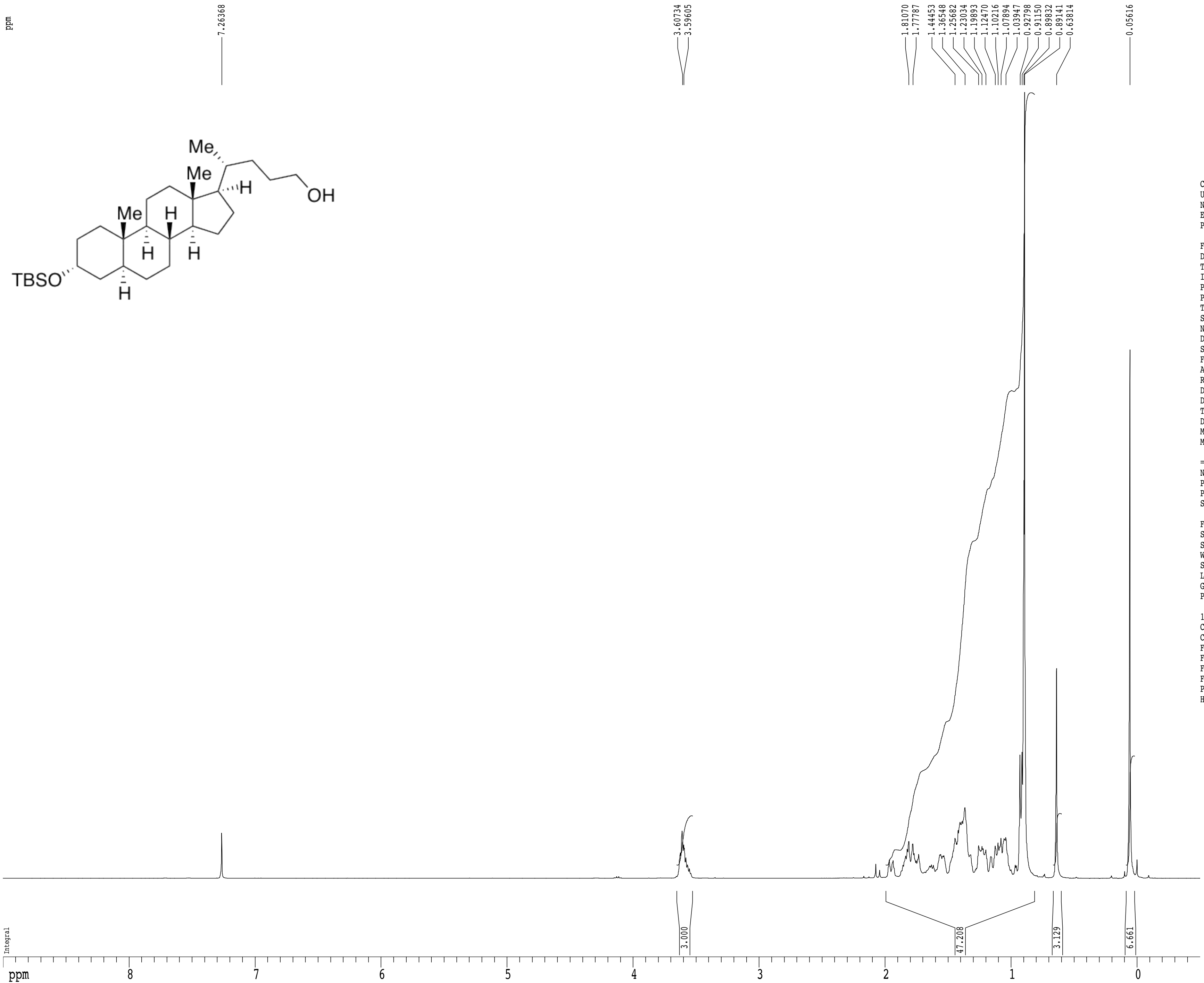


```
Current Data Parameters
USER              linpc2
NAME           pcl-3-044
EXPNO                  1
PROCNO                 1

F2 - Acquisition Parameters
Date_           20220214
Time               14.21
INSTRUM           drx400
PROBHD   5 mm QNP H/F/P
PULPROG             zg30
TD                 65536
SOLVENT            CDCl3
NS                     8
DS                     2
SWH             6410.256 Hz
FIDRES          0.097813 Hz
AQ             5.1118579 sec
RG                    64
DW                78.000 usec
DE                  4.50 usec
TE                 298.0 K
D1            0.10000000 sec
MCREST        0.00000000 sec
MCWRK         0.01500000 sec

======== CHANNEL f1 ========
NUC1                  1H
P1                 12.00 usec
PL1                -0.90 dB
SFO1         400.1328009 MHz

F2 - Processing parameters
SI                 65536
SF           400.1300197 MHz
WDW                   EM
SSB                    0
LB                  0.30 Hz
GB                     0
PC                  2.00

1D NMR plot parameters
CX                 22.80 cm
CY                 15.00 cm
F1P                9.000 ppm
F1               3601.17 Hz
F2P               -0.500 ppm
F2               -200.06 Hz
PPMCM            0.41667 ppm/cm
HZCM           166.72084 Hz/cm
```

1H spectrum

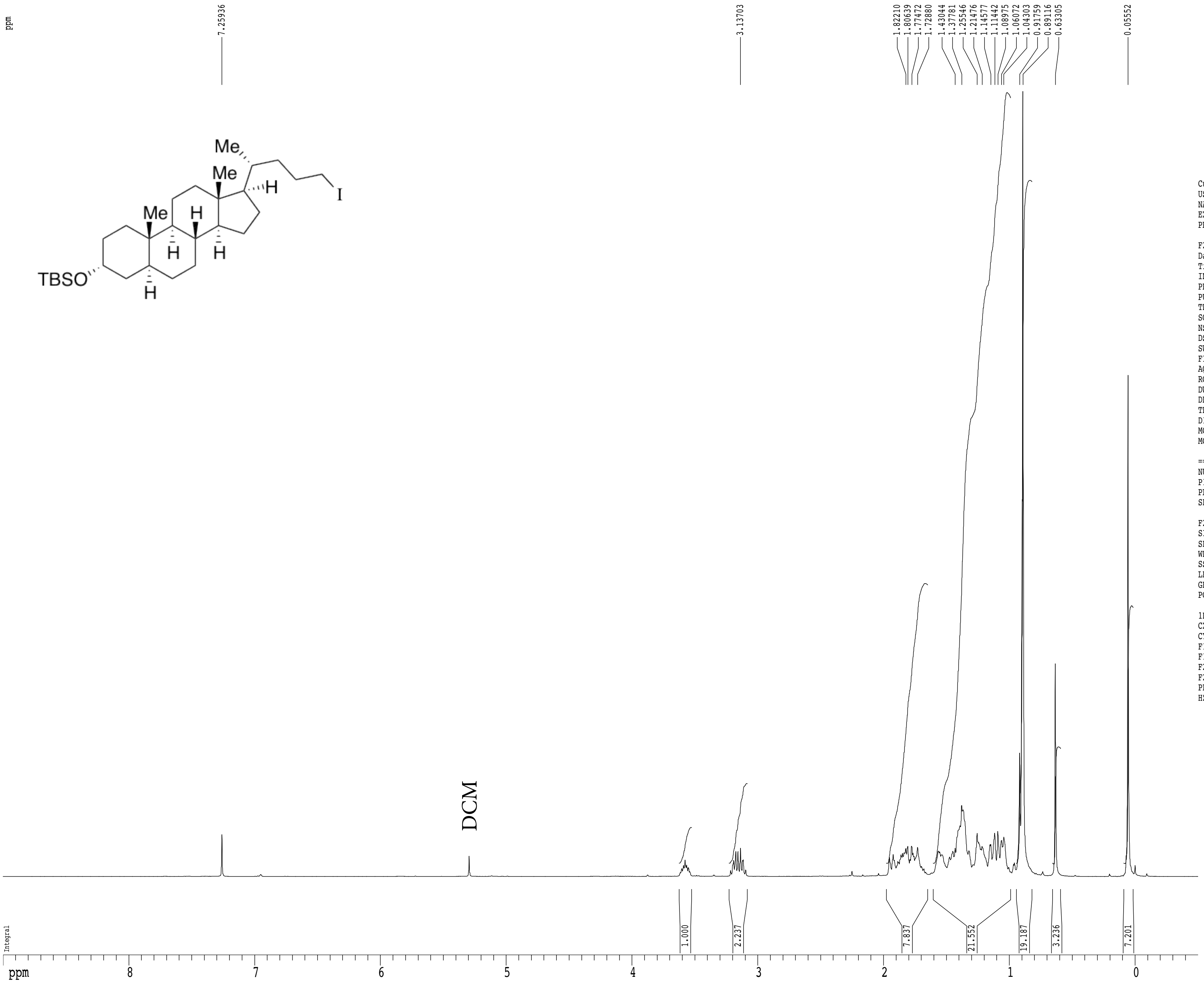


```
Current Data Parameters
USER          linpc2
NAME          pcl-3-046
EXPNO         4
PROCNO        1

F2 - Acquisition Parameters
Date_         20220218
Time          15.23
INSTRUM       drx400
PROBHD   5 mm QNP H/F/P
PULPROG       zg30
TD            65536
SOLVENT       CDCl3
NS            8
DS            2
SWH           6410.256 Hz
FIDRES        0.097813 Hz
AQ            5.1118579 sec
RG            57
DW            78.000 usec
DE            4.50 usec
TE            298.0 K
D1            0.10000000 sec
MCREST        0.00000000 sec
MCWRK         0.01500000 sec

======== CHANNEL f1 ========
NUC1          1H
P1            12.00 usec
PL1           -0.90 dB
SFO1          400.1328009 MHz

F2 - Processing parameters
SI            65536
SF            400.1300219 MHz
WDW           EM
SSB           0
LB            0.30 Hz
GB            0
PC            2.00

1D NMR plot parameters
CX            22.80 cm
CY            15.00 cm
F1P           9.000 ppm
F1            3601.17 Hz
F2P           -0.500 ppm
F2            -200.06 Hz
PPMCM         0.41667 ppm/cm
HZCM          166.72086 Hz/cm
```

1H spectrum

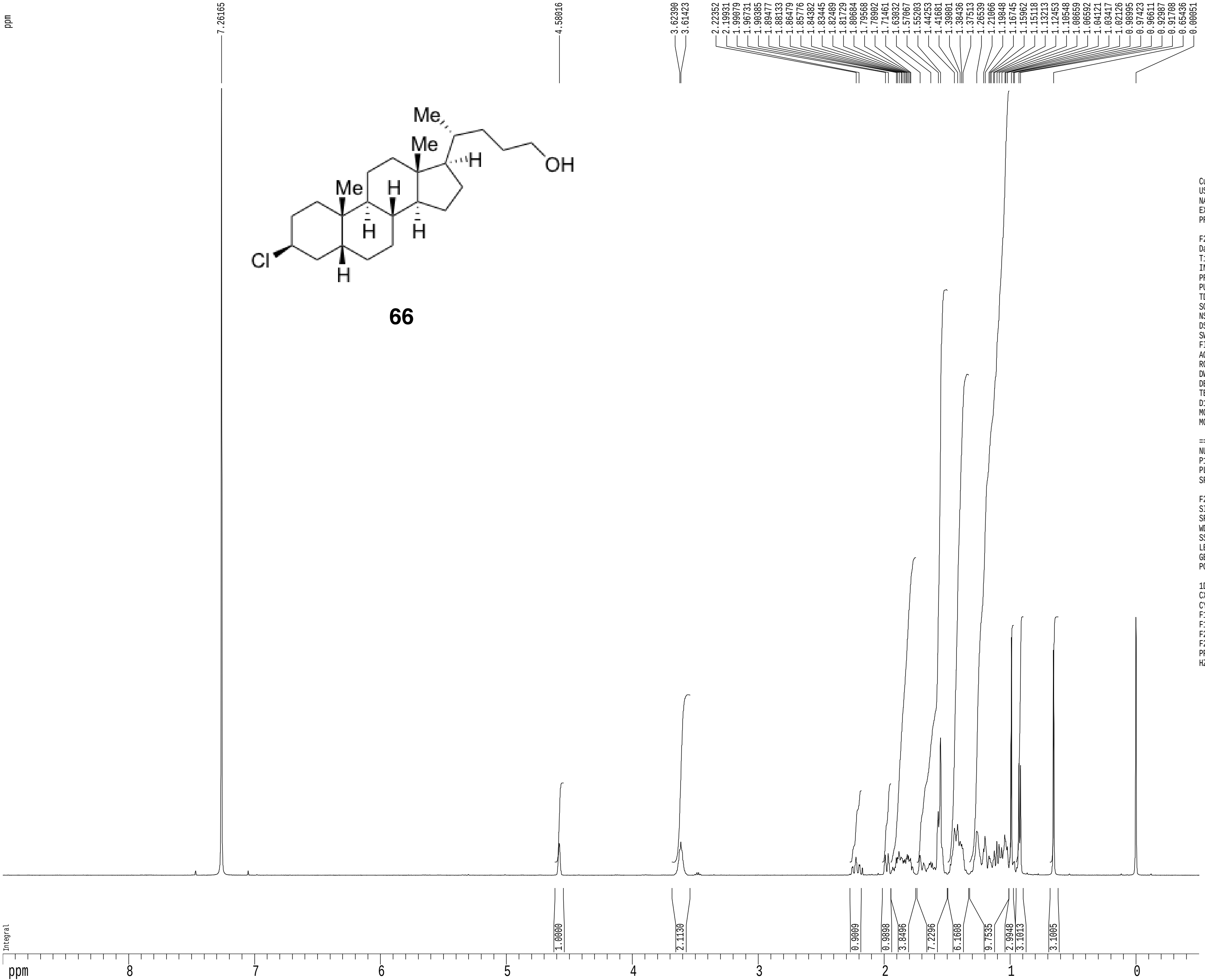

66

Current Data Parameters
USER nhirbawi
NAME NH-3-38-HCCOSY-good
EXPNO 1
PROCNO 1

F2 - Acquisition Parameters
Date_ 20220402
Time 15.12
INSTRUM cryo500
PROBHD 5 mm CPTCI 1H-
PULPROG zg30
TD 81728
SOLVENT CDCl3T
NS 8
DS 2
SWH 8012.820 Hz
FIDRES 0.098043 Hz
AQ 5.0998774 sec
RG 6.3
DW 62.400 usec
DE 6.00 usec
TE 298.0 K
D1 0.10000000 sec
MCREST 0.00000000 sec
MCWRK 0.01500000 sec

======== CHANNEL f1 ========
NUC1 1H
P1 9.75 usec
PL1 1.60 dB
SFO1 500.2235015 MHz

F2 - Processing parameters
SI 65536
SF 500.2200299 MHz
WDW EM
SSB 0
LB 0.30 Hz
GB 0
PC 1.00

1D NMR plot parameters
CX 22.80 cm
CY 15.00 cm
F1P 9.000 ppm
F1 4501.98 Hz
F2P -0.500 ppm
F2 -250.11 Hz
PPMCM 0.41667 ppm/cm
HZCM 208.42502 Hz/cm

Z-restored spin-echo 13C spectrum with 1H decoupling

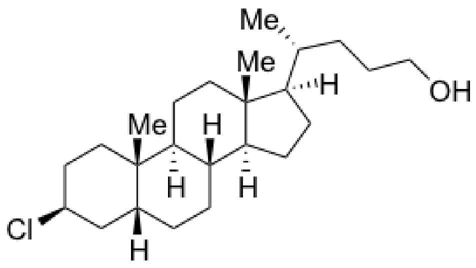

66

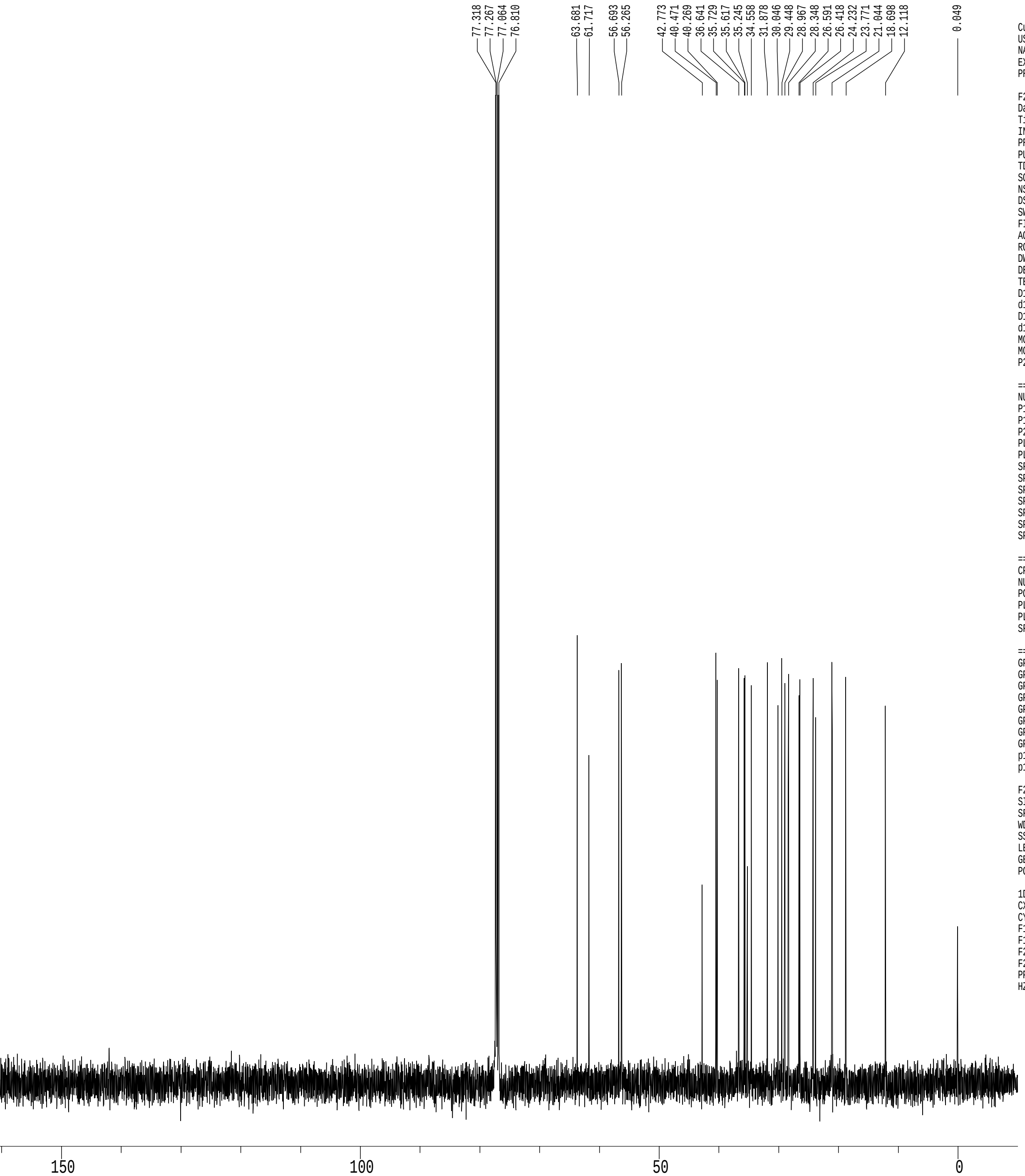


```
Current Data Parameters
USER            nhirbawi
NAME      NH-3-38-HCCOSY-good
EXPNO                  2
PROCNO                 1

F2 - Acquisition Parameters
Date_           20220402
Time               15.18
INSTRUM          cryo500
PROBHD   5 mm CPTCI 1H-
PULPROG  SpinEchopg30gp2.prd
TD                 65536
SOLVENT            CDCl3
NS                   699
DS                    16
SWH            30303.031 Hz
FIDRES          0.462388 Hz
AQ             1.0813940 sec
RG               11585.2
DW                16.500 usec
DE                  6.00 usec
TE                 298.0 K
D1            0.25000000 sec
d11           0.03000000 sec
D16           0.00020000 sec
d17           0.00019600 sec
MCREST        0.00000000 sec
MCWRK         0.01500000 sec
P2                 37.70 usec

======== CHANNEL f1 ========
NUC1                 13C
P1                 18.85 usec
P12              2000.00 usec
P20               500.00 usec
PL0               120.00 dB
PL1                -1.00 dB
SFO1         125.7942548 MHz
SP2                 1.55 dB
SP4                 1.55 dB
SPNAM2       Crp60comp.4
SPNAM4    Crp60,0.5,20.1
SPOFF2              0.00 Hz
SPOFF4              0.00 Hz

======== CHANNEL f2 ========
CPDPRG2          waltz16
NUC2                  1H
PCPD2             100.00 usec
PL2                 1.60 dB
PL12               22.00 dB
SFO2         500.2225011 MHz

====== GRADIENT CHANNEL =====
GPNAM1          SINE.100
GPNAM2          SINE.100
GPX1                0.00 %
GPX2                0.00 %
GPY1                0.00 %
GPY2                0.00 %
GPZ1               30.00 %
GPZ2               50.00 %
p15               500.00 usec
p16              1000.00 usec

F2 - Processing parameters
SI                 65536
SF           125.7804190 MHz
WDW                   EM
SSB                    0
LB                  1.00 Hz
GB                     0
PC                  2.00

1D NMR plot parameters
CX                 22.80 cm
CY                 50.00 cm
F1P              230.000 ppm
F1              28929.50 Hz
F2P              -10.000 ppm
F2              -1257.80 Hz
PPMCM           10.52632 ppm/cm
HZCM          1324.00439 Hz/cm
```

gcosy60

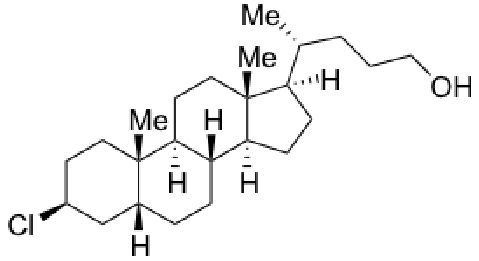

66

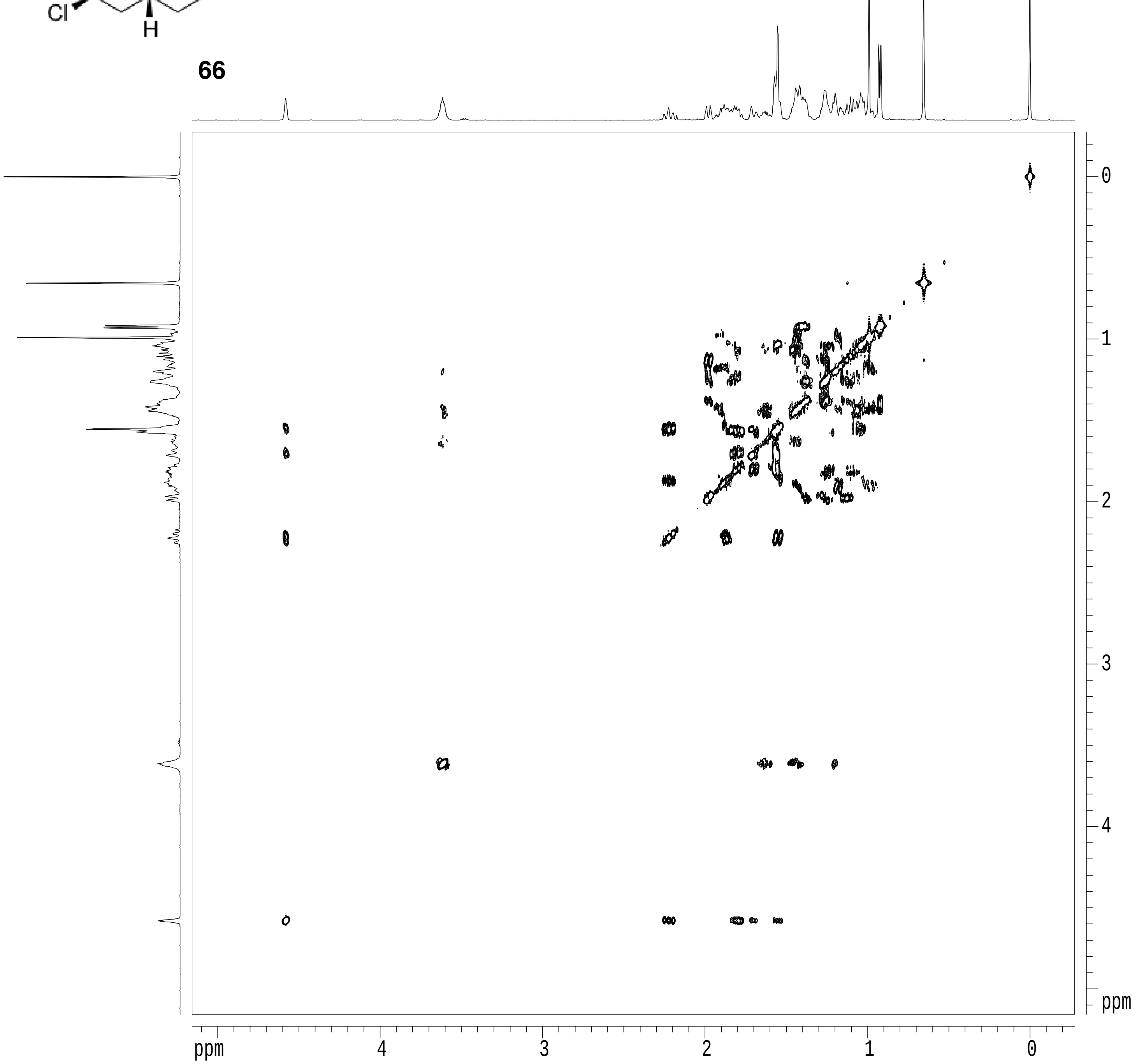


```
Current Data Parameters
USER          nhirbawi
NAME     NH-3-38-HCCOSY-good
EXPNO                3
PROCNO               1

F2 - Acquisition Parameters
Date_         20220402
Time             15.31
INSTRUM        cryo500
PROBHD   5 mm CPTCI 1H-
PULPROG    cosygp60.prd
TD                2048
SOLVENT         CDCl3T
NS                   2
DS                  16
SWH           2717.391 Hz
FIDRES        1.326851 Hz
AQ           0.3768820 sec
RG                 181
DW             184.000 usec
DE                6.00 usec
TE               298.0 K
d0          0.00000300 sec
D1          1.00000000 sec
d13         0.00000300 sec
D16         0.00020000 sec
IN0         0.00036800 sec

======== CHANNEL f1 ========
NUC1                1H
P1                9.75 usec
PL1               1.60 dB
SFO1       500.2212511 MHz

====== GRADIENT CHANNEL =====
GPNAM1      SMSQ10.100
GPNAM2      SMSQ10.100
GPX1              0.00 %
GPX2              0.00 %
GPY1              0.00 %
GPY2              0.00 %
GPZ1             17.00 %
GPZ2             17.00 %
P16            1000.00 usec

F1 - Acquisition parameters
ND0                  1
TD                 167
SFO1          500.2213 MHz
FIDRES       16.271805 Hz
SW               5.432 ppm
FnMODE              QF

F2 - Processing parameters
SI                1024
SF         500.2200299 MHz
WDW               SINE
SSB                  0
LB                0.00 Hz
GB                   0
PC                2.00

F1 - Processing parameters
SI                1024
MC2                 QF
SF         500.2200299 MHz
WDW               SINE
SSB                  0
LB                0.00 Hz
GB                   0

2D NMR plot parameters
CX2              15.00 cm
CX1              15.00 cm
F2PLO            5.158 ppm
F2LO           2579.91 Hz
F2PHI           -0.275 ppm
F2HI           -137.48 Hz
F1PLO            5.158 ppm
F1LO           2579.91 Hz
F1PHI           -0.275 ppm
F1HI         [illegible] Hz
F2PPMCM        0.36216 ppm/cm
F2HZCM       181.15942 Hz/cm
F1PPMCM        0.36216 ppm/cm
F1HZCM       181.15942 Hz/cm
```

1H spectrum

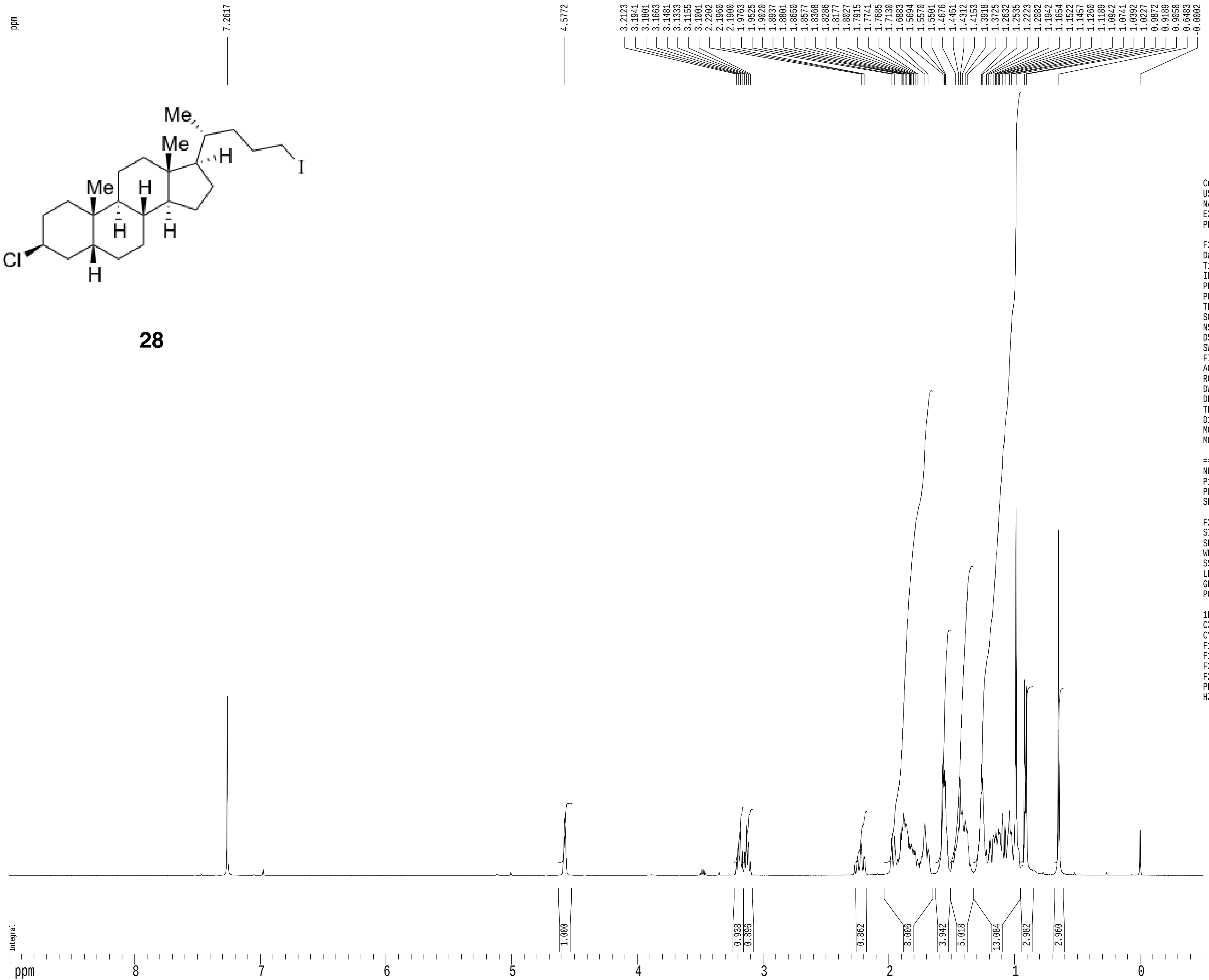

Current Data Parameters
USER nhirbawi
NAME NH-3-52-HCCOSY
EXPNO 1
PROCNO 1

F2 - Acquisition Parameters
Date_ 20220330
Time 13.27
INSTRUM cryo500
PROBHD 5 mm CPTCI 1H-
PULPROG zg30
TD 81728
SOLVENT CDCl3T
NS 8
DS 2
SWH 8012.820 Hz
FIDRES 0.098043 Hz
AQ 5.0998774 sec
RG 3.6
DW 62.400 usec
DE 6.00 usec
TE 298.0 K
D1 0.10000000 sec
MCREST 0.00000000 sec
MCWRK 0.01500000 sec

======== CHANNEL f1 ========
NUC1 1H
P1 9.75 usec
PL1 1.60 dB
SFO1 500.2235015 MHz

F2 - Processing parameters
SI 65536
SF 500.2200299 MHz
WDW EM
SSB 0
LB 0.30 Hz
GB 0
PC 1.00

1D NMR plot parameters
CX 22.80 cm
CY 7.00 cm
F1P 9.000 ppm
F1 4501.98 Hz
F2P -0.500 ppm
F2 -250.11 Hz
PPMCM 0.41667 ppm/cm
HZCM 208.42502 Hz/cm

Z-restored spin-echo 13C spectrum with 1H decoupling

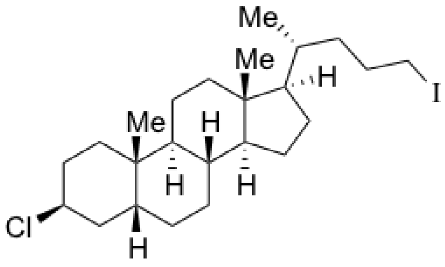

28

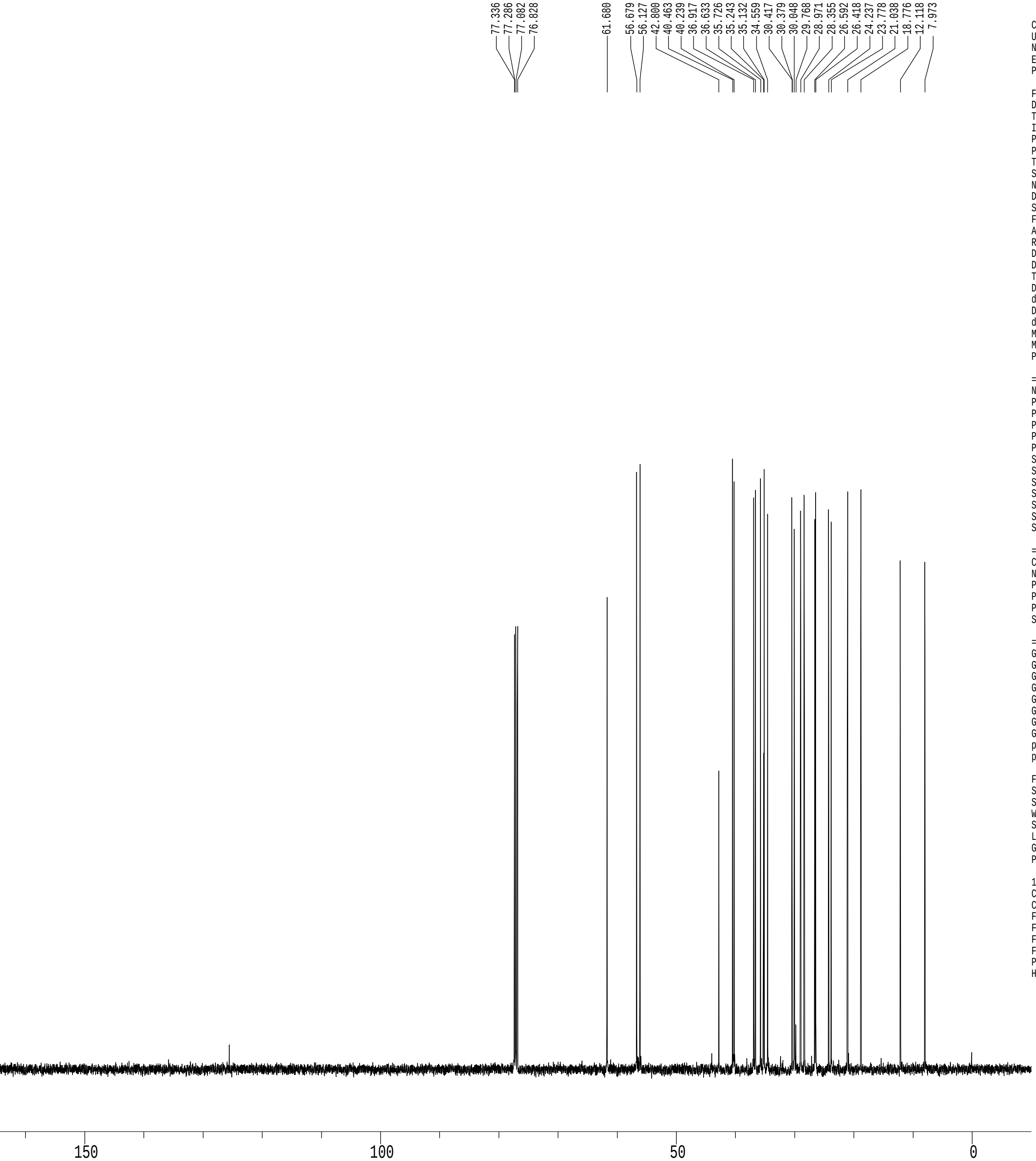


```
Current Data Parameters
USER           nhirbawi
NAME     NH-3-52-HCCOSY
EXPNO                 2
PROCNO                1

F2 - Acquisition Parameters
Date_          20220330
Time              13.29
INSTRUM         cryo500
PROBHD   5 mm CPTCI 1H-
PULPROG  SpinEchopg30gp2.prd
TD                65536
SOLVENT           CDCl3
NS                  281
DS                   16
SWH           30303.031 Hz
FIDRES         0.462388 Hz
AQ            1.0813940 sec
RG               7298.2
DW               16.500 usec
DE                 6.00 usec
TE                298.0 K
D1           0.25000000 sec
d11          0.03000000 sec
D16          0.00020000 sec
d17          0.00019600 sec
MCREST       0.00000000 sec
MCWRK        0.01500000 sec
P2                37.70 usec

======== CHANNEL f1 ========
NUC1                13C
P1                18.85 usec
P12             2000.00 usec
P20              500.00 usec
PL0              120.00 dB
PL1               -1.00 dB
SFO1        125.7942548 MHz
SP2                1.55 dB
SP4                1.55 dB
SPNAM2      Crp60comp.4
SPNAM4   Crp60,0.5,20.1
SPOFF2             0.00 Hz
SPOFF4             0.00 Hz

======== CHANNEL f2 ========
CPDPRG2         waltz16
NUC2                 1H
PCPD2            100.00 usec
PL2                1.60 dB
PL12              22.00 dB
SFO2        500.2225011 MHz

====== GRADIENT CHANNEL =====
GPNAM1         SINE.100
GPNAM2         SINE.100
GPX1               0.00 %
GPX2               0.00 %
GPY1               0.00 %
GPY2               0.00 %
GPZ1              30.00 %
GPZ2              50.00 %
p15              500.00 usec
p16             1000.00 usec

F2 - Processing parameters
SI                65536
SF          125.7804190 MHz
WDW                  EM
SSB                   0
LB                 1.00 Hz
GB                    0
PC                 2.00

1D NMR plot parameters
CX                22.80 cm
CY                10.00 cm
F1P             230.000 ppm
F1             28929.50 Hz
F2P             -10.000 ppm
F2             -1257.80 Hz
PPMCM          10.52632 ppm/cm
HZCM         1324.00439 Hz/cm
```

gcosy60

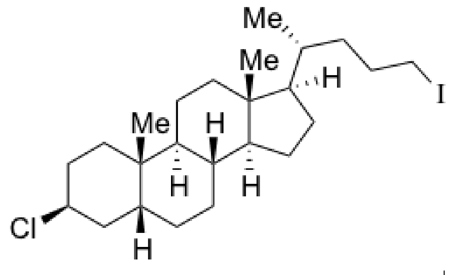

28

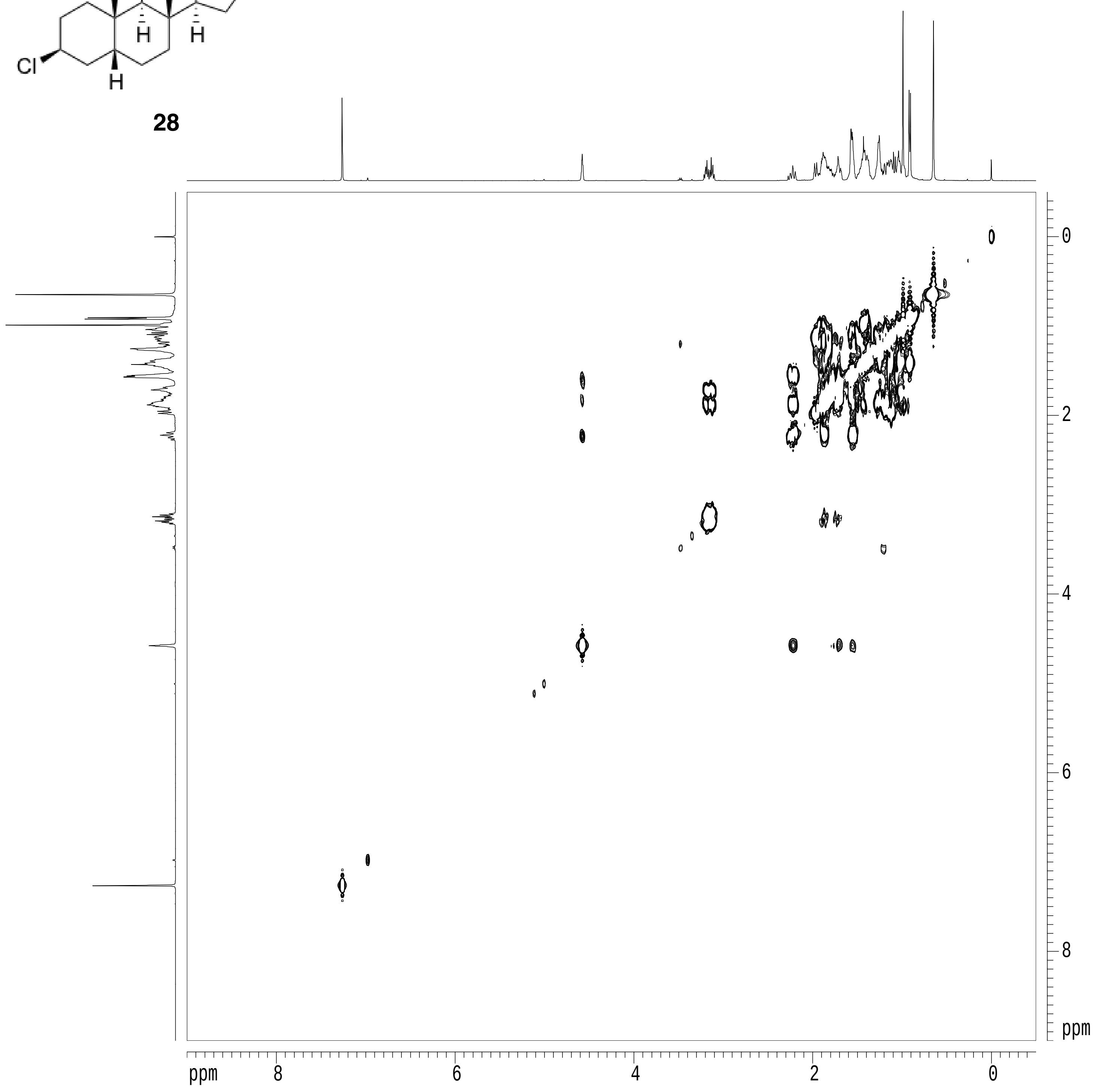


```
Current Data Parameters
USER        nhirbawi
NAME        NH-3-52-HCCOSY
EXPNO       3
PROCNO      1

F2 - Acquisition Parameters
Date_       20220330
Time        13.35
INSTRUM     cryo500
PROBHD   5 mm CPTCI 1H-
PULPROG     cosygp60.prd
TD          2048
SOLVENT     CDCl3
NS          2
DS          16
SWH         8012.820 Hz
FIDRES      3.912510 Hz
AQ          0.1278452 sec
RG          35.9
DW          62.400 usec
DE          6.00 usec
TE          298.0 K
d0          0.00000300 sec
D1          1.00000000 sec
d13         0.00000300 sec
D16         0.00020000 sec
IN0         0.00012480 sec

======== CHANNEL f1 ========
NUC1        1H
P1          9.75 usec
PL1         1.60 dB
SFO1        500.2235015 MHz

====== GRADIENT CHANNEL =====
GPNAM1      SMSQ10.100
GPNAM2      SMSQ10.100
GPX1        0.00 %
GPX2        0.00 %
GPY1        0.00 %
GPY2        0.00 %
GPZ1        17.00 %
GPZ2        17.00 %
P16         1000.00 usec

F1 - Acquisition parameters
ND0         1
TD          137
SFO1        500.2235 MHz
FIDRES      58.487740 Hz
SW          16.018 ppm
FnMODE      QF

F2 - Processing parameters
SI          1024
SF          500.2200298 MHz
WDW         SINE
SSB         0
LB          0.00 Hz
GB          0
PC          1.00

F1 - Processing parameters
SI          1024
MC2         QF
SF          500.2200298 MHz
WDW         SINE
SSB         0
LB          0.00 Hz
GB          0

2D NMR plot parameters
CX2         15.00 cm
CX1         15.00 cm
F2PLO       9.000 ppm
F2LO        4501.98 Hz
F2PHI       -0.500 ppm
F2HI        -250.11 Hz
F1PLO       9.000 ppm
F1LO        4501.98 Hz
F1PHI       -0.500 ppm
F1HI        -25[illegible] Hz
F2PPMCM     0.63333 ppm/cm
F2HZCM      316.80603 Hz/cm
F1PPMCM     0.63333 ppm/cm
F1HZCM      316.80603 Hz/cm
```

1H spectrum

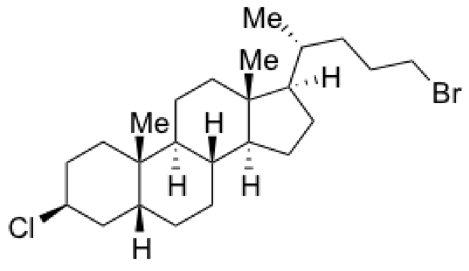

37

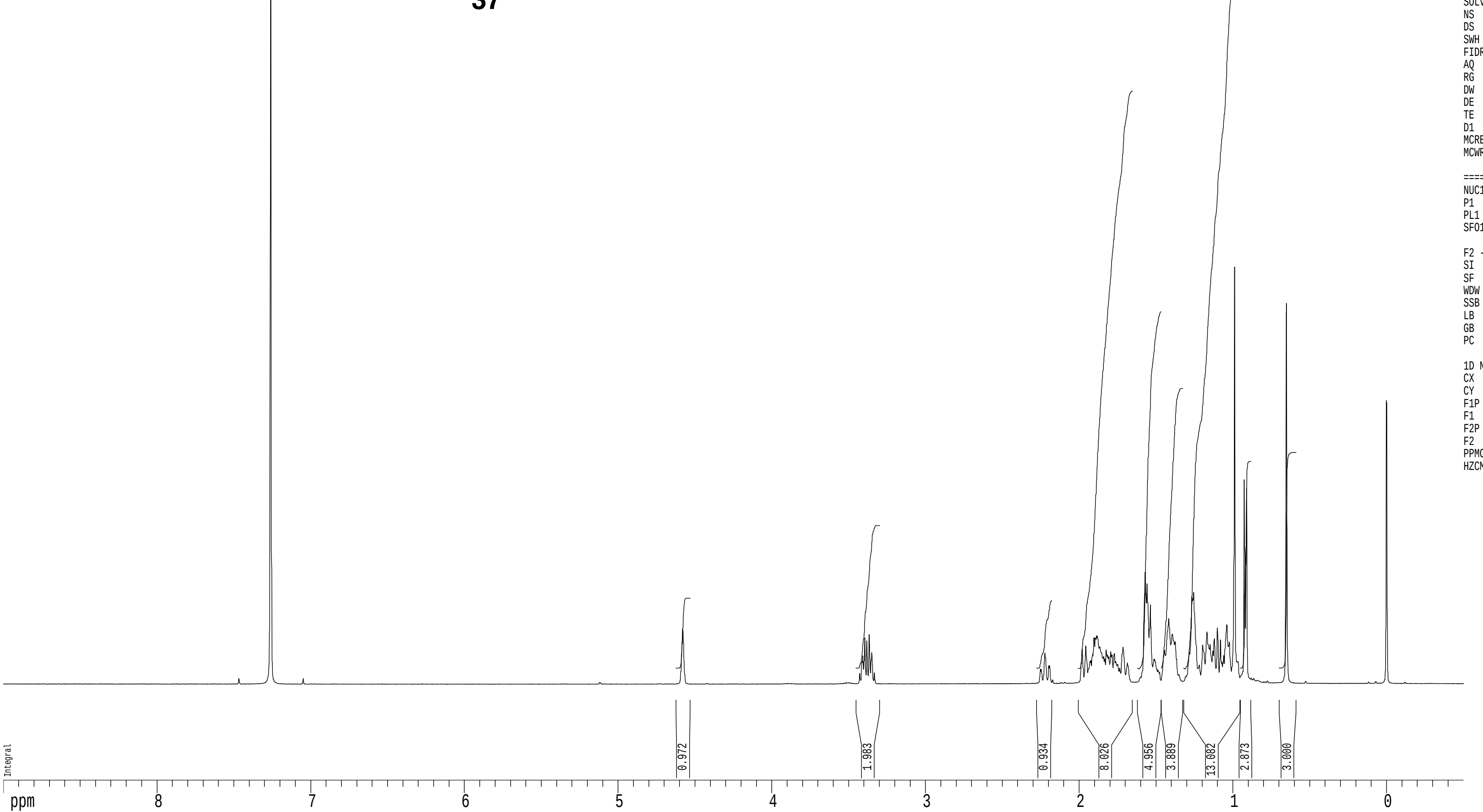


```
Current Data Parameters
USER            nhirbawi
NAME     NH-3-54-HCCOSY
EXPNO                  1
PROCNO                 1

F2 - Acquisition Parameters
Date_           20220402
Time               14.44
INSTRUM          cryo500
PROBHD   5 mm CPTCI 1H-
PULPROG             zg30
TD                 81728
SOLVENT           CDCl3T
NS                     8
DS                     2
SWH            8012.820 Hz
FIDRES         0.098043 Hz
AQ            5.0998774 sec
RG                     5
DW               62.400 usec
DE                 6.00 usec
TE                298.0 K
D1           0.10000000 sec
MCREST       0.00000000 sec
MCWRK        0.01500000 sec

======== CHANNEL f1 ========
NUC1                  1H
P1                  9.75 usec
PL1                 1.60 dB
SFO1         500.2235015 MHz

F2 - Processing parameters
SI                 65536
SF           500.2200308 MHz
WDW                   EM
SSB                    0
LB                  0.30 Hz
GB                     0
PC                  1.00

1D NMR plot parameters
CX                 22.80 cm
CY                 15.00 cm
F1P                9.000 ppm
F1               4501.98 Hz
F2P               -0.500 ppm
F2               -250.11 Hz
PPMCM            0.41667 ppm/cm
HZCM           208.42502 Hz/cm
```

Z-restored spin-echo 13C spectrum with 1H decoupling

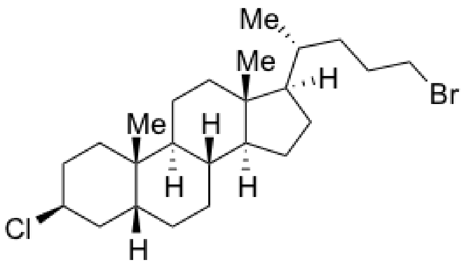

37

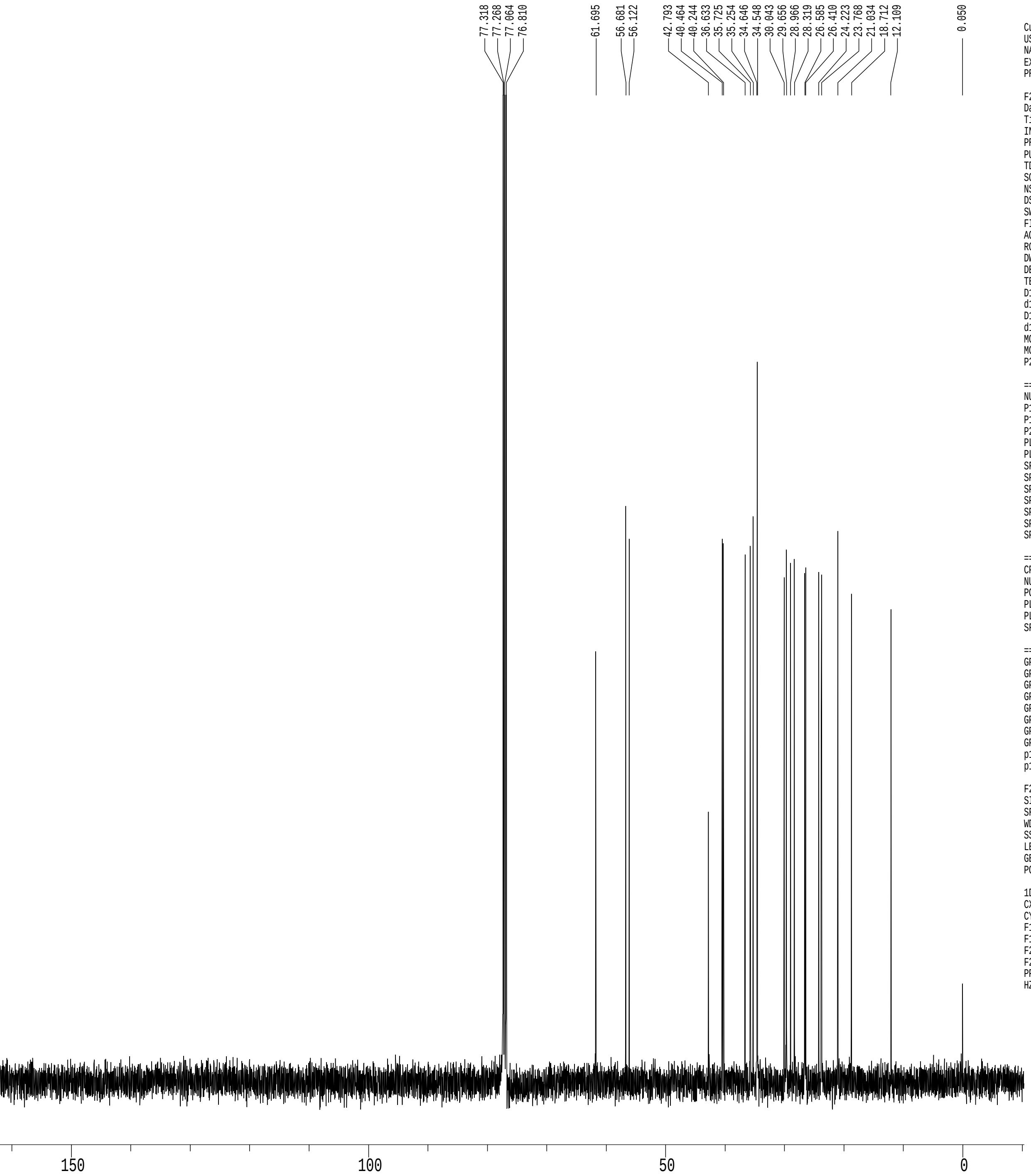

Current Data Parameters
USER nhirbawi
NAME NH-3-54-HCCOSY
EXPNO 2
PROCNO 1

F2 - Acquisition Parameters
Date_ 20220402
Time 14.47
INSTRUM cryo500
PROBHD 5 mm CPTCI 1H-
PULPROG SpinEchopg30gp2.prd
TD 65536
SOLVENT CDCl3
NS 613
DS 16
SWH 30303.031 Hz
FIDRES 0.462388 Hz
AQ 1.0813940 sec
RG 16384
DW 16.500 usec
DE 6.00 usec
TE 298.0 K
D1 0.25000000 sec
d11 0.03000000 sec
D16 0.00020000 sec
d17 0.00019600 sec
MCREST 0.00000000 sec
MCWRK 0.01500000 sec
P2 37.70 usec

======== CHANNEL f1 ========
NUC1 13C
P1 18.85 usec
P12 2000.00 usec
P20 500.00 usec
PL0 120.00 dB
PL1 -1.00 dB
SFO1 125.7942548 MHz
SP2 1.55 dB
SP4 1.55 dB
SPNAM2 Crp60comp.4
SPNAM4 Crp60,0.5,20.1
SPOFF2 0.00 Hz
SPOFF4 0.00 Hz

======== CHANNEL f2 ========
CPDPRG2 waltz16
NUC2 1H
PCPD2 100.00 usec
PL2 1.60 dB
PL12 22.00 dB
SFO2 500.2225011 MHz

====== GRADIENT CHANNEL =====
GPNAM1 SINE.100
GPNAM2 SINE.100
GPX1 0.00 %
GPX2 0.00 %
GPY1 0.00 %
GPY2 0.00 %
GPZ1 30.00 %
GPZ2 50.00 %
p15 500.00 usec
p16 1000.00 usec

F2 - Processing parameters
SI 65536
SF 125.7804190 MHz
WDW EM
SSB 0
LB 1.00 Hz
GB 0
PC 2.00

1D NMR plot parameters
CX 22.80 cm
CY 35.00 cm
F1P 230.637 ppm
F1 29009.68 Hz
F2P -10.287 ppm
F2 -1293.96 Hz
PPMCM 10.56688 ppm/cm
HZCM 1329.10706 Hz/cm

gcosy60

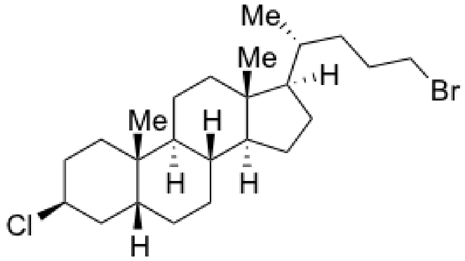

37

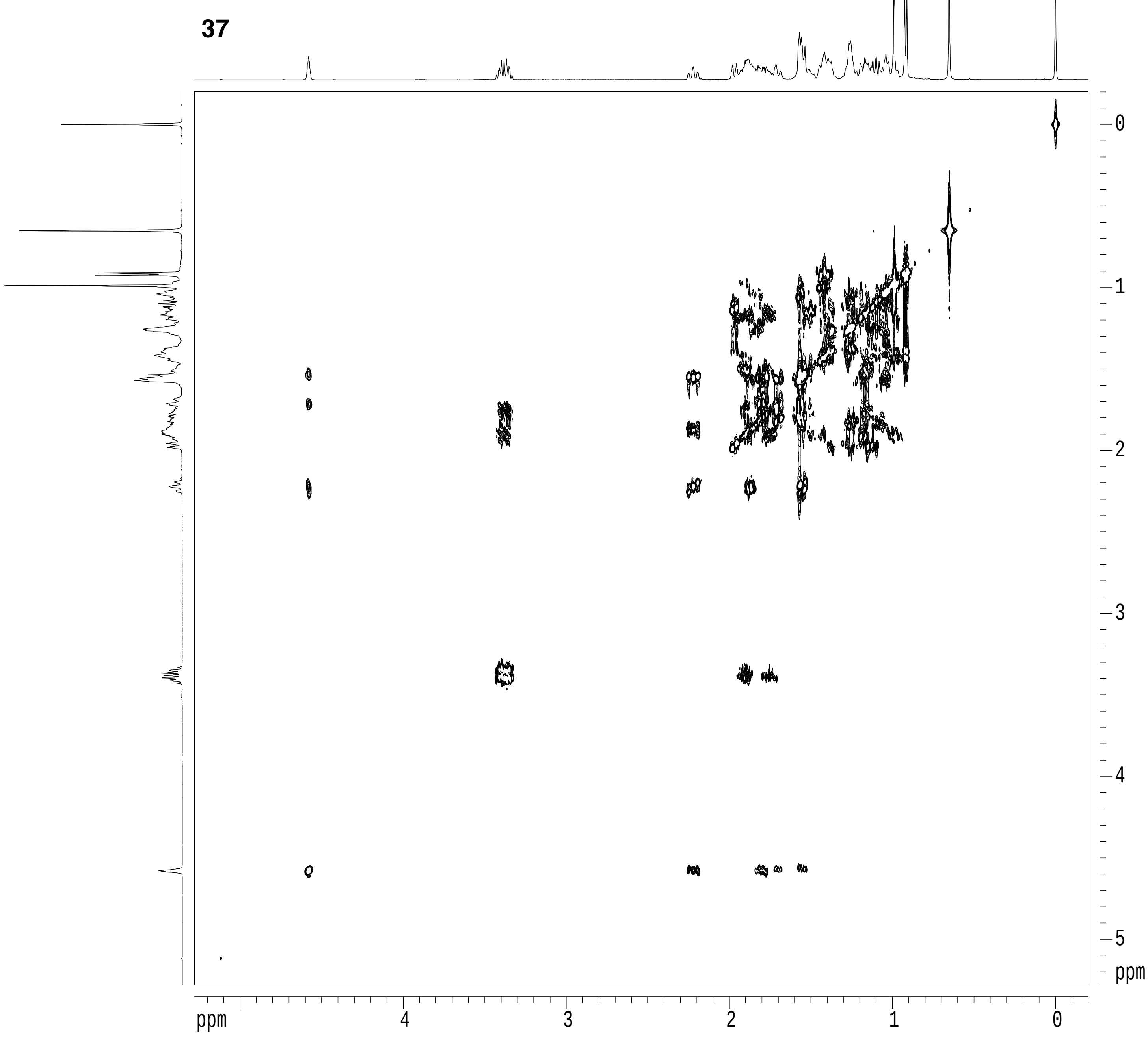

Current Data Parameters
USER nhirbawi
NAME NH-3-54-HCCOSY
EXPNO 3
PROCNO 1

F2 - Acquisition Parameters
Date_ 20220402
Time 15.01
INSTRUM cryo500
PROBHD 5 mm CPTCI 1H-
PULPROG cosygp60.prd
TD 2048
SOLVENT CDCl3T
NS 2
DS 16
SWH 2741.228 Hz
FIDRES 1.338490 Hz
AQ 0.3736052 sec
RG 128
DW 182.400 usec
DE 6.00 usec
TE 298.0 K
d0 0.00000300 sec
D1 1.00000000 sec
d13 0.00000300 sec
D16 0.00020000 sec
IN0 0.00036480 sec

======== CHANNEL f1 ========
NUC1 1H
P1 9.75 usec
PL1 1.60 dB
SFO1 500.2213011 MHz

====== GRADIENT CHANNEL =====
GPNAM1 SMSQ10.100
GPNAM2 SMSQ10.100
GPX1 0.00 %
GPX2 0.00 %
GPY1 0.00 %
GPY2 0.00 %
GPZ1 17.00 %
GPZ2 17.00 %
P16 1000.00 usec

F1 - Acquisition parameters
ND0 1
TD 150
SFO1 500.2213 MHz
FIDRES 18.274855 Hz
SW 5.480 ppm
FnMODE QF

F2 - Processing parameters
SI 1024
SF 500.2200308 MHz
WDW SINE
SSB 0
LB 0.00 Hz
GB 0
PC 1.00

F1 - Processing parameters
SI 1024
MC2 QF
SF 500.2200308 MHz
WDW SINE
SSB 0
LB 0.00 Hz
GB 0

2D NMR plot parameters
CX2 15.00 cm
CX1 15.00 cm
F2PLO 5.280 ppm
F2LO 2640.97 Hz
F2PHI -0.200 ppm
F2HI -100.26 Hz
F1PLO 5.280 ppm
F1LO 2640.97 Hz
F1PHI -0.200 ppm
F1HI [illegible]
F2PPMCM 0.36534 ppm/cm
F2HZCM 182.74854 Hz/cm
F1PPMCM 0.36534 ppm/cm
F1HZCM 182.74854 Hz/cm

1H spectrum

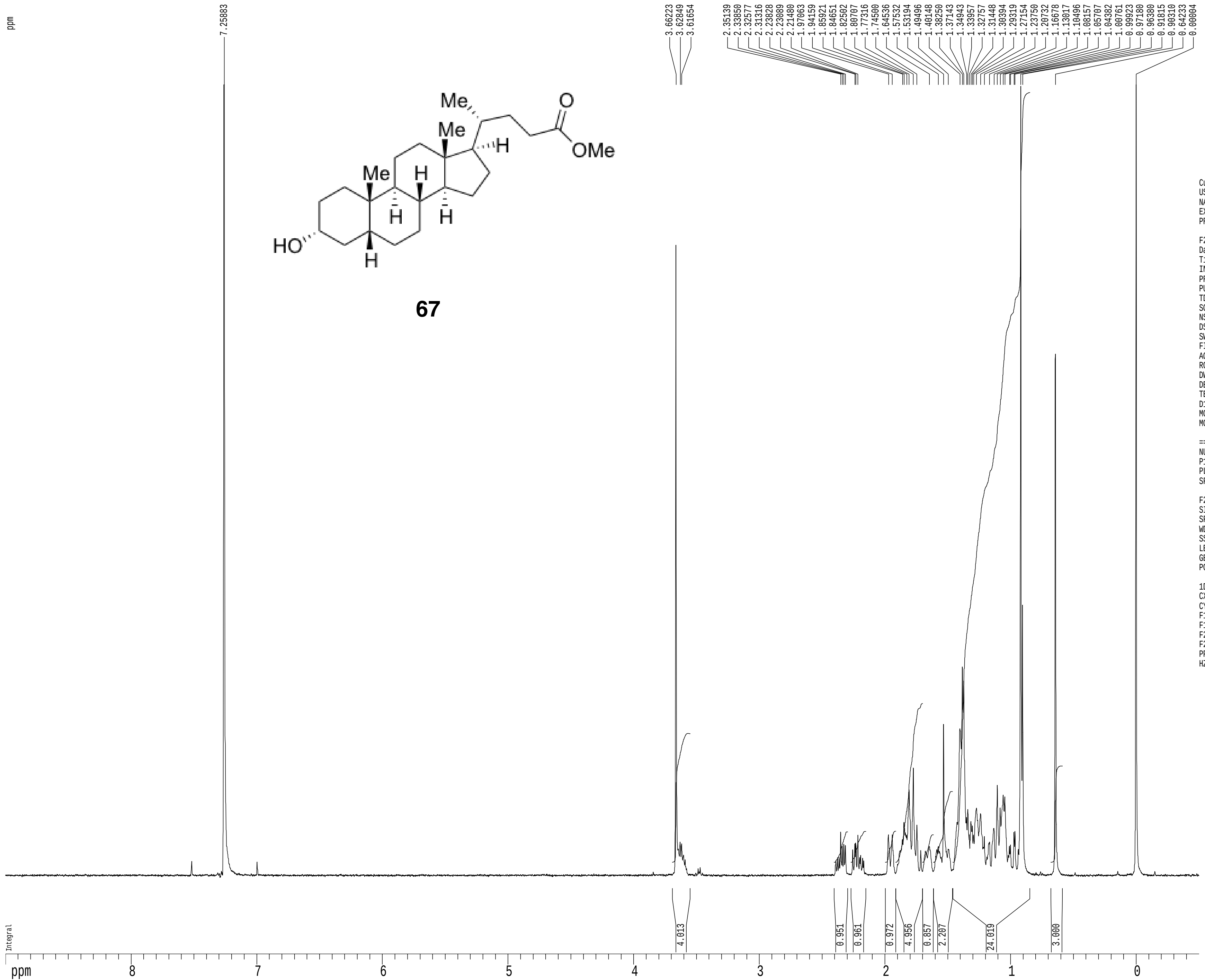

67

```
Current Data Parameters
USER          nhirbawi
NAME     NH-3-41-column-dry2
EXPNO                1
PROCNO               1

F2 - Acquisition Parameters
Date_          20220322
Time              13.58
INSTRUM          drx400
PROBHD   5 mm QNP H/F/P
PULPROG            zg30
TD                65536
SOLVENT          CDCl3T
NS                    8
DS                    2
SWH            6410.256 Hz
FIDRES         0.097813 Hz
AQ            5.1118579 sec
RG                574.7
DW               78.000 usec
DE                 4.50 usec
TE                298.0 K
D1           0.10000000 sec
MCREST       0.00000000 sec
MCWRK        0.01500000 sec

======== CHANNEL f1 ========
NUC1                 1H
P1                12.00 usec
PL1               -0.90 dB
SFO1        400.1328009 MHz

F2 - Processing parameters
SI                65536
SF          400.1300219 MHz
WDW                  EM
SSB                   0
LB                 0.30 Hz
GB                    0
PC                 2.00

1D NMR plot parameters
CX                22.80 cm
CY                50.00 cm
F1P               9.000 ppm
F1              3601.17 Hz
F2P              -0.500 ppm
F2              -200.06 Hz
PPMCM           0.41667 ppm/cm
HZCM          166.72086 Hz/cm
```

1H spectrum

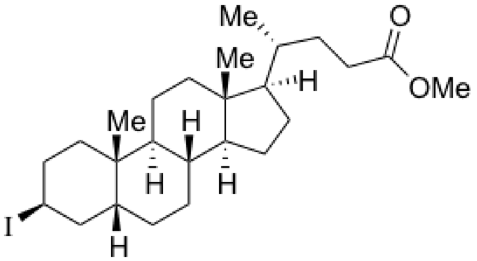

29

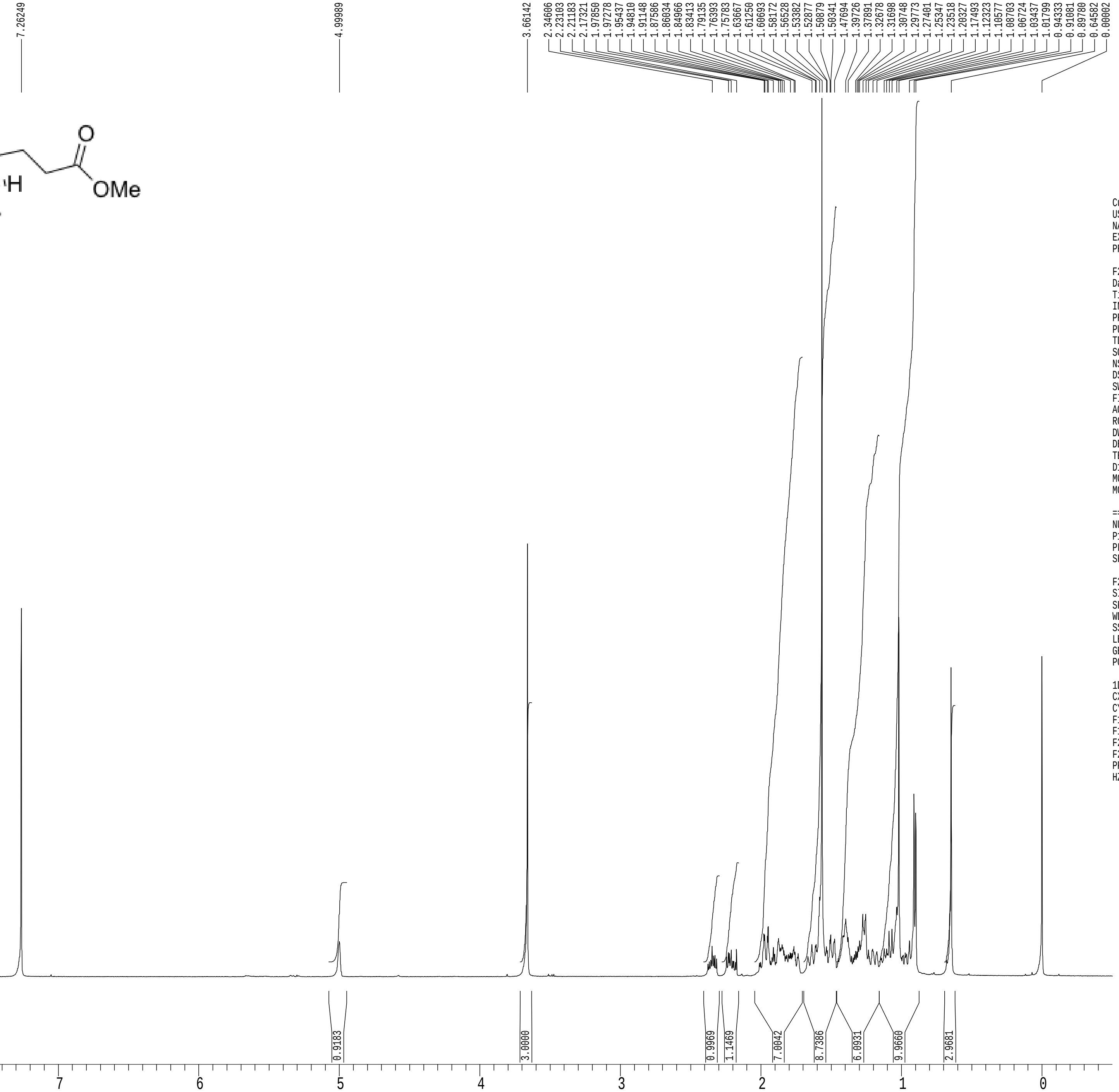


```
Current Data Parameters
USER            nhirbawi
NAME          NH-3-78-1H
EXPNO                  1
PROCNO                 1

F2 - Acquisition Parameters
Date_           20220507
Time               12.41
INSTRUM          cryo500
PROBHD   5 mm CPTCI 1H-
PULPROG             zg30
TD                 81728
SOLVENT           CDCl3T
NS                     8
DS                     2
SWH             8012.820 Hz
FIDRES          0.098043 Hz
AQ             5.0998774 sec
RG                   6.3
DW                62.400 usec
DE                  6.00 usec
TE                 298.0 K
D1            0.10000000 sec
MCREST        0.00000000 sec
MCWRK         0.01500000 sec

======== CHANNEL f1 ========
NUC1                  1H
P1                  9.75 usec
PL1                 1.60 dB
SFO1         500.2235015 MHz

F2 - Processing parameters
SI                 65536
SF           500.2200309 MHz
WDW                   EM
SSB                    0
LB                  0.30 Hz
GB                     0
PC                  1.00

1D NMR plot parameters
CX                 22.80 cm
CY                 15.00 cm
F1P                9.000 ppm
F1               4501.98 Hz
F2P               -0.500 ppm
F2               -250.11 Hz
PPMCM            0.41667 ppm/cm
HZCM           208.42502 Hz/cm
```

1H spectrum

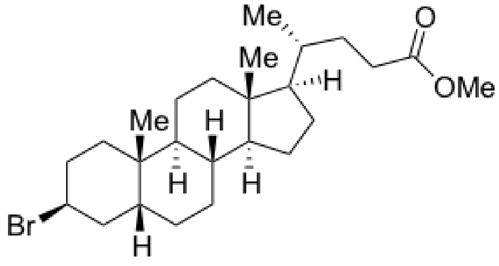

38

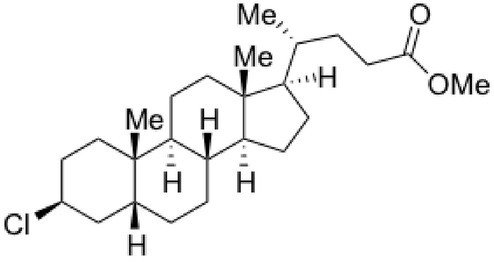

68

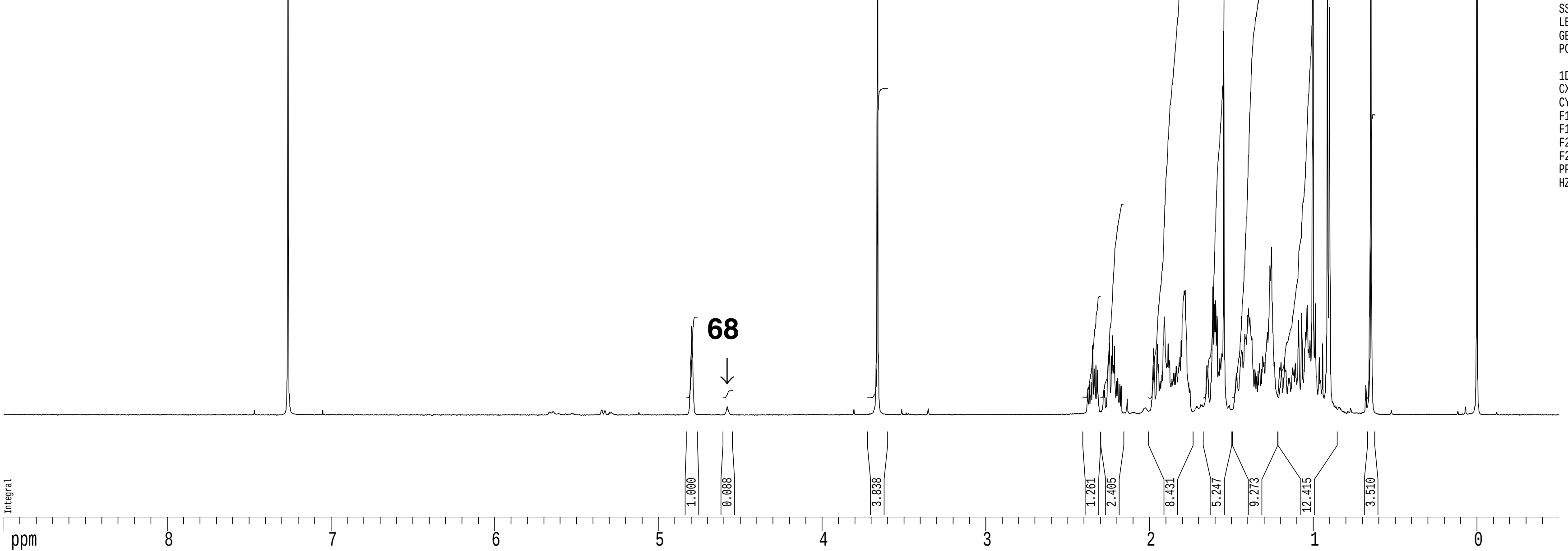


```
Current Data Parameters
USER           nhirbawi
NAME         NH-3-68-1H
EXPNO                 1
PROCNO                1

F2 - Acquisition Parameters
Date_          20220507
Time              12.33
INSTRUM         cryo500
PROBHD   5 mm CPTCI 1H-
PULPROG            zg30
TD                81728
SOLVENT          CDCl3T
NS                    8
DS                    2
SWH            8012.820 Hz
FIDRES         0.098043 Hz
AQ            5.0998774 sec
RG                  5.7
DW               62.400 usec
DE                 6.00 usec
TE                298.0 K
D1           0.10000000 sec
MCREST       0.00000000 sec
MCWRK        0.01500000 sec

======== CHANNEL f1 ========
NUC1                 1H
P1                 9.75 usec
PL1                1.60 dB
SFO1        500.2235015 MHz

F2 - Processing parameters
SI                65536
SF          500.2200316 MHz
WDW                  EM
SSB                   0
LB                 0.30 Hz
GB                    0
PC                 1.00

1D NMR plot parameters
CX                22.80 cm
CY                15.00 cm
F1P               9.000 ppm
F1              4501.98 Hz
F2P              -0.500 ppm
F2              -250.11 Hz
PPMCM           0.41667 ppm/cm
HZCM          208.42502 Hz/cm
```

1H spectrum

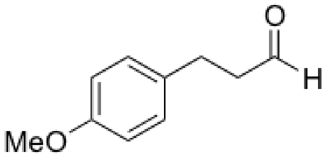

SI-2

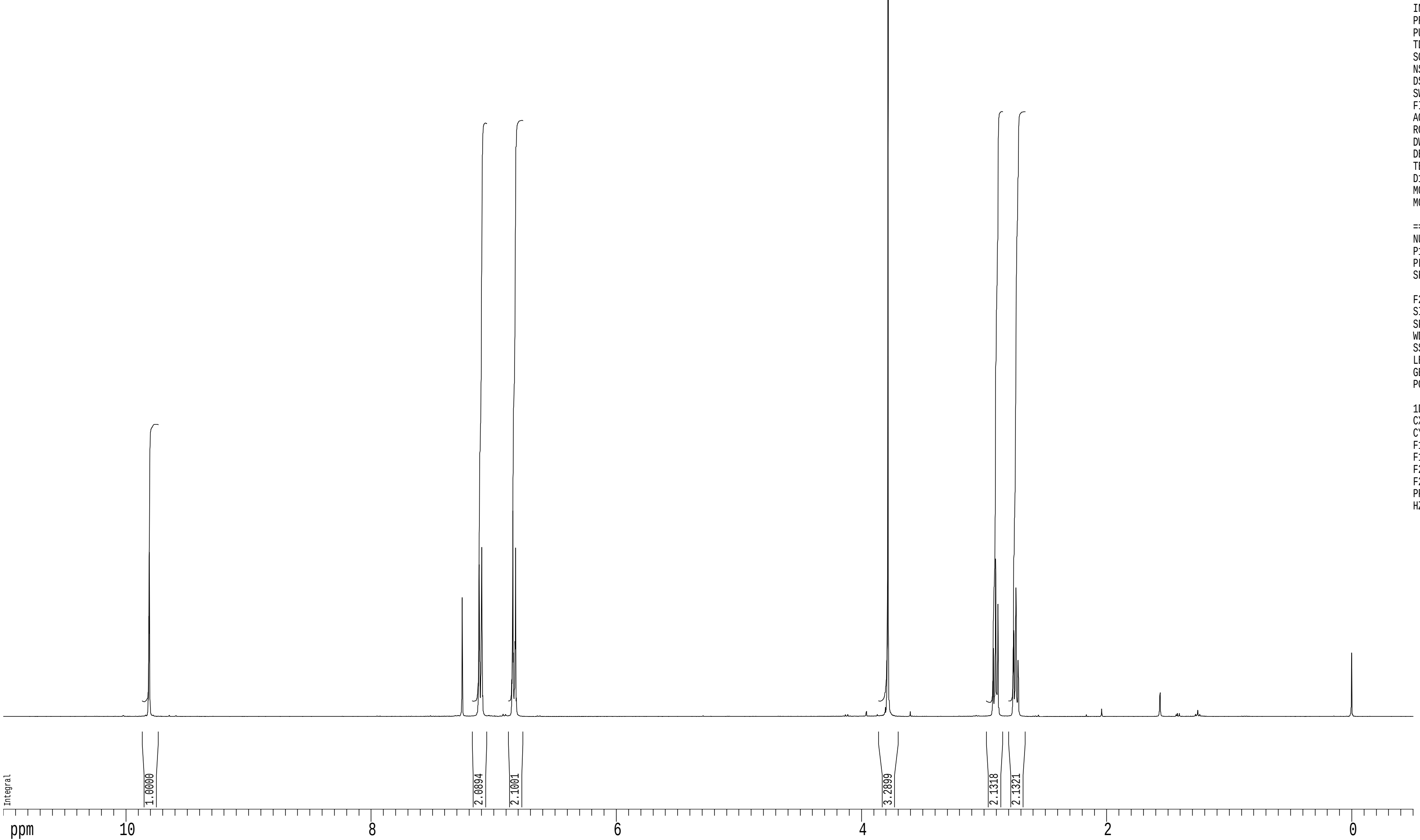


```
Current Data Parameters
USER          nhirbawi
NAME        NH-2-24-pdt
EXPNO                 1
PROCNO                1

F2 - Acquisition Parameters
Date_          20210809
Time              15.13
INSTRUM          drx400
PROBHD   5 mm QNP H/F/P
PULPROG            zg30
TD                65536
SOLVENT          CDCl3T
NS                    8
DS                    2
SWH            6410.256 Hz
FIDRES         0.097813 Hz
AQ            5.1118579 sec
RG                322.5
DW               78.000 usec
DE                 4.50 usec
TE                298.0 K
D1           0.10000000 sec
MCREST       0.00000000 sec
MCWRK        0.01500000 sec

======== CHANNEL f1 ========
NUC1                 1H
P1                12.00 usec
PL1               -1.60 dB
SFO1        400.1328009 MHz

F2 - Processing parameters
SI                65536
SF          400.1300229 MHz
WDW                  EM
SSB                   0
LB                 0.30 Hz
GB                    0
PC                 2.00

1D NMR plot parameters
CX                22.80 cm
CY                15.00 cm
F1P              11.000 ppm
F1              4401.43 Hz
F2P              -0.500 ppm
F2              -200.07 Hz
PPMCM           0.50439 ppm/cm
HZCM          201.81998 Hz/cm
```

1H spectrum

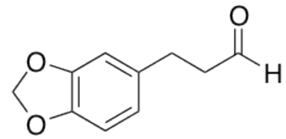

SI-4

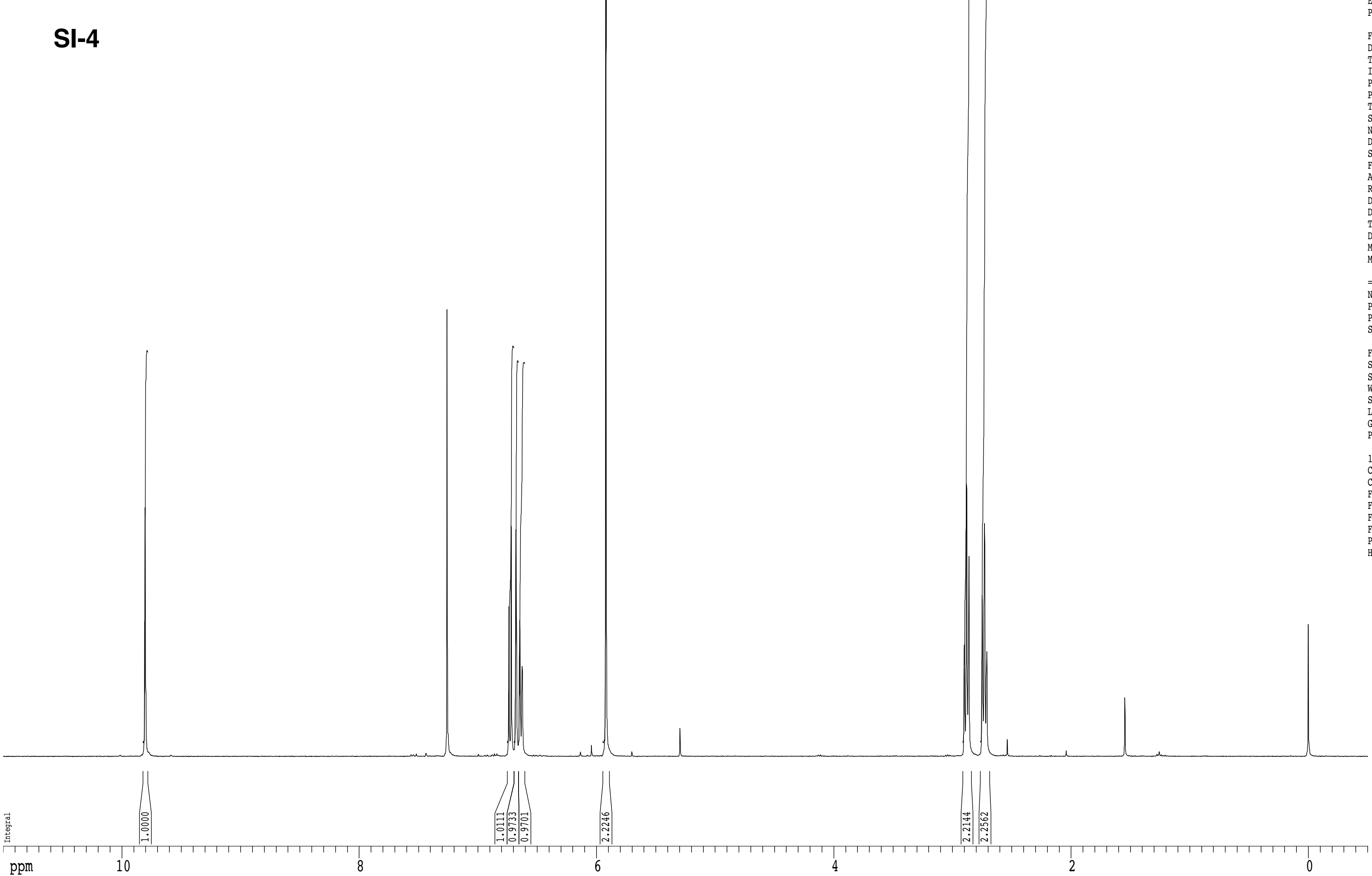

Current Data Parameters
USER linpc2
NAME pcl-2-261
EXPNO 1
PROCNO 1

F2 - Acquisition Parameters
Date_ 20211209
Time 14.54
INSTRUM drx400
PROBHD 5 mm QNP H/F/P
PULPROG zg30
TD 65536
SOLVENT CDCl3
NS 8
DS 2
SWH 6410.256 Hz
FIDRES 0.097813 Hz
AQ 5.1118579 sec
RG 322.5
DW 78.000 usec
DE 4.50 usec
TE 298.0 K
D1 0.10000000 sec
MCREST 0.00000000 sec
MCWRK 0.01500000 sec

======== CHANNEL f1 ========
NUC1 1H
P1 12.00 usec
PL1 -0.90 dB
SFO1 400.1328009 MHz

F2 - Processing parameters
SI 65536
SF 400.1300221 MHz
WDW EM
SSB 0
LB 0.30 Hz
GB 0
PC 2.00

1D NMR plot parameters
CX 22.80 cm
CY 15.00 cm
F1P 11.000 ppm
F1 4401.43 Hz
F2P -0.500 ppm
F2 -200.07 Hz
PPMCM 0.50439 ppm/cm
HZCM 201.81998 Hz/cm

1H spectrum

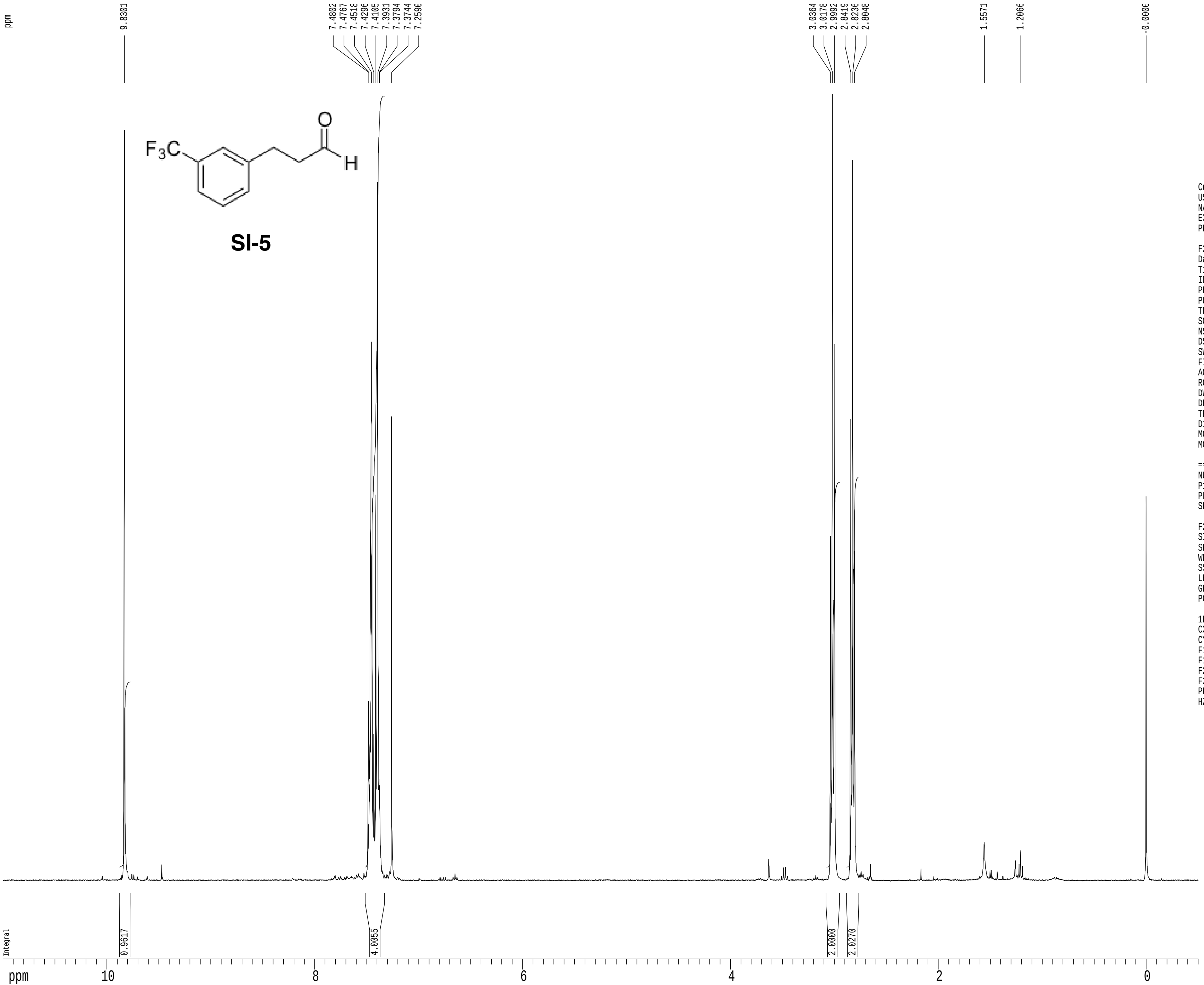

Current Data Parameters
USER nhirbawi
NAME NH-2-88-column
EXPNO 1
PROCNO 1

F2 - Acquisition Parameters
Date_ 20211022
Time 16.58
INSTRUM drx400
PROBHD 5 mm Multinucl
PULPROG zg30
TD 65536
SOLVENT CDCl3T
NS 8
DS 2
SWH 6410.256 Hz
FIDRES 0.097813 Hz
AQ 5.1118579 sec
RG 362
DW 78.000 usec
DE 4.50 usec
TE 298.1 K
D1 0.10000000 sec
MCREST 0.00000000 sec
MCWRK 0.01500000 sec

======== CHANNEL f1 ========
NUC1 1H
P1 12.00 usec
PL1 -1.10 dB
SFO1 400.1328009 MHz

F2 - Processing parameters
SI 65536
SF 400.1300221 MHz
WDW EM
SSB 0
LB 0.30 Hz
GB 0
PC 2.00

1D NMR plot parameters
CX 22.80 cm
CY 15.00 cm
F1P 11.000 ppm
F1 4401.43 Hz
F2P -0.500 ppm
F2 -200.07 Hz
PPMCM 0.50439 ppm/cm
HZCM 201.81998 Hz/cm

1H spectrum

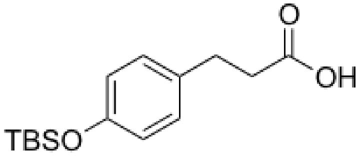

SI-6

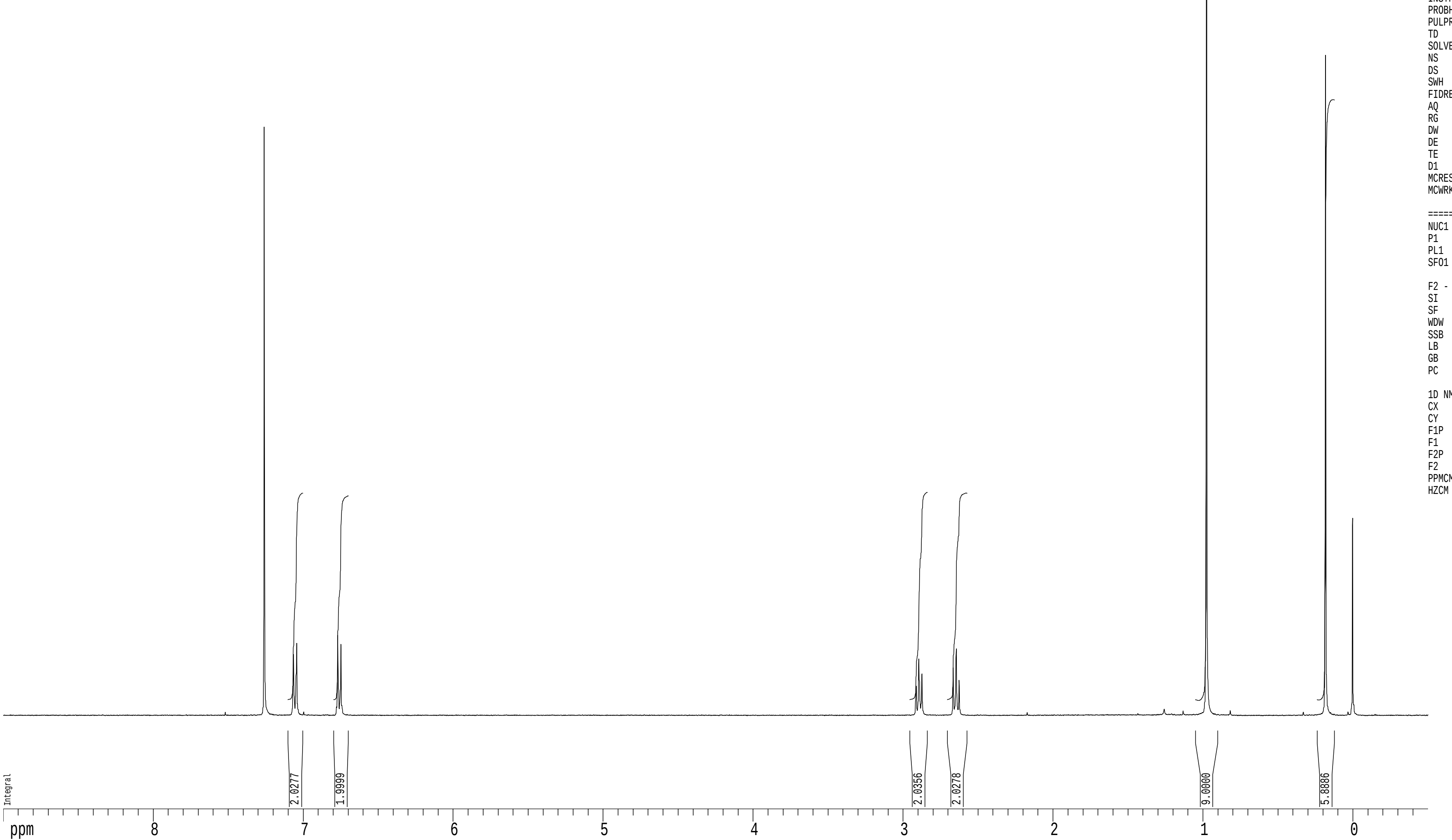

Current Data Parameters
USER nhirbawi
NAME NH-2-102-frac30-4
EXPNO 1
PROCNO 1

F2 - Acquisition Parameters
Date_ 20211206
Time 14.36
INSTRUM drx400
PROBHD 5 mm QNP H/F/P
PULPROG zg30
TD 65536
SOLVENT CDCl3T
NS 8
DS 2
SWH 6410.256 Hz
FIDRES 0.097813 Hz
AQ 5.1118579 sec
RG 912.3
DW 78.000 usec
DE 4.50 usec
TE 298.0 K
D1 0.10000000 sec
MCREST 0.00000000 sec
MCWRK 0.01500000 sec

======== CHANNEL f1 ========
NUC1 1H
P1 12.00 usec
PL1 -0.90 dB
SFO1 400.1328009 MHz

F2 - Processing parameters
SI 65536
SF 400.1300215 MHz
WDW EM
SSB 0
LB 0.30 Hz
GB 0
PC 2.00

1D NMR plot parameters
CX 22.80 cm
CY 15.00 cm
F1P 9.000 ppm
F1 3601.17 Hz
F2P -0.500 ppm
F2 -200.06 Hz
PPMCM 0.41667 ppm/cm
HZCM 166.72086 Hz/cm

1H spectrum

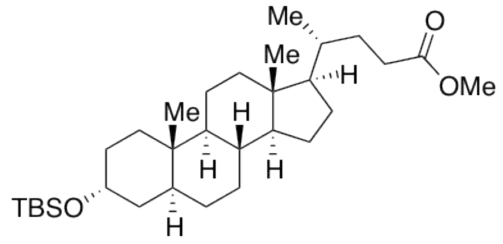

SI-7

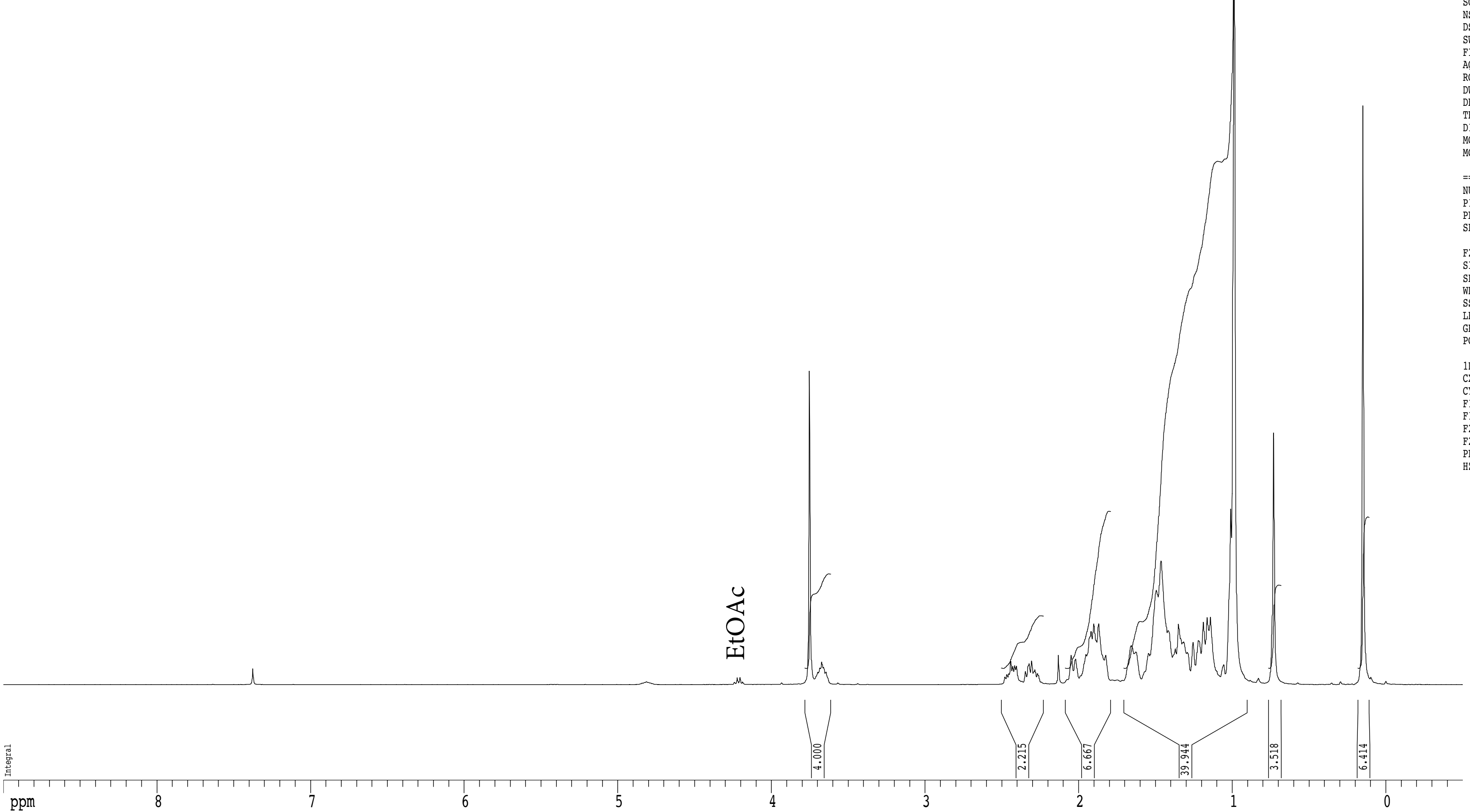


```
Current Data Parameters
USER              linpc2
NAME          pcl-3-042
EXPNO                  1
PROCNO                 1

F2 - Acquisition Parameters
Date_           20220211
Time               15.06
INSTRUM           drx400
PROBHD   5 mm QNP H/F/P
PULPROG             zg30
TD                 65536
SOLVENT            CDCl3
NS                     8
DS                     2
SWH           6410.256 Hz
FIDRES        0.097813 Hz
AQ           5.1118579 sec
RG                    32
DW              78.000 usec
DE                4.50 usec
TE               298.0 K
D1          0.10000000 sec
MCREST      0.00000000 sec
MCWRK       0.01500000 sec

======== CHANNEL f1 ========
NUC1                  1H
P1               12.00 usec
PL1              -0.90 dB
SFO1         400.1328009 MHz

F2 - Processing parameters
SI                 65536
SF           400.1299751 MHz
WDW                   EM
SSB                    0
LB                  0.30 Hz
GB                     0
PC                  2.00

1D NMR plot parameters
CX                 22.80 cm
CY                 15.00 cm
F1P                9.000 ppm
F1               3601.17 Hz
F2P               -0.500 ppm
F2               -200.06 Hz
PPMCM            0.41667 ppm/cm
HZCM           166.72083 Hz/cm
```

1H spectrum

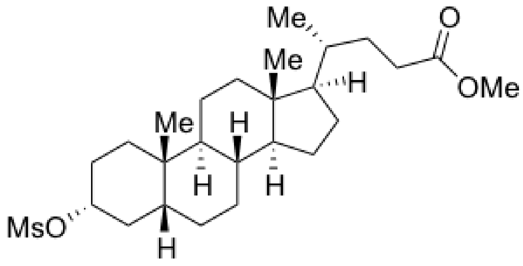

SI-8

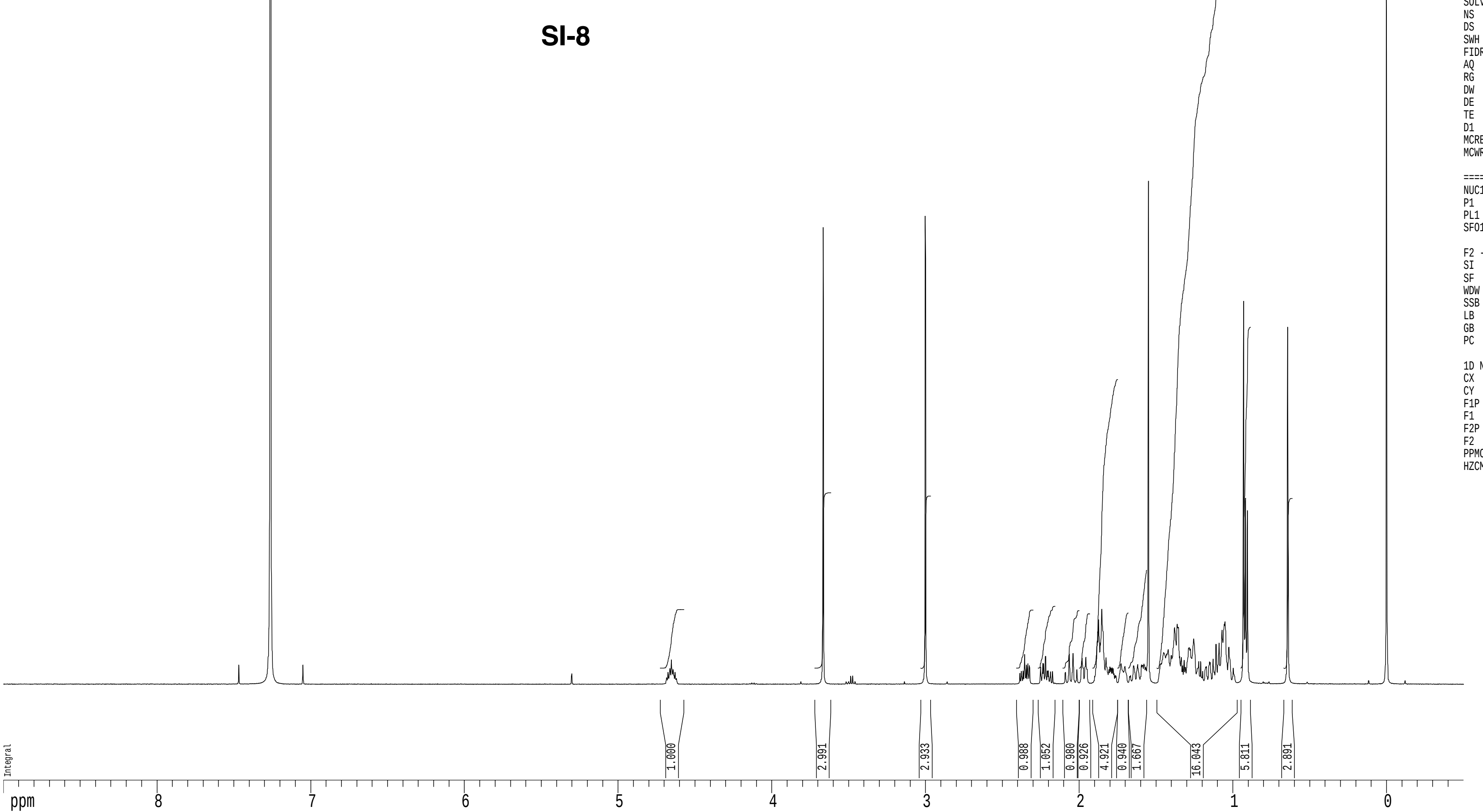


```
Current Data Parameters
USER          nhirbawi
NAME        NH-3-70-1H
EXPNO                1
PROCNO               1

F2 - Acquisition Parameters
Date_         20220507
Time             12.45
INSTRUM        cryo500
PROBHD   5 mm CPTCI 1H-
PULPROG           zg30
TD               81728
SOLVENT         CDCl3T
NS                   8
DS                   2
SWH           8012.820 Hz
FIDRES        0.098043 Hz
AQ           5.0998774 sec
RG                 6.3
DW              62.400 usec
DE                6.00 usec
TE               298.0 K
D1          0.10000000 sec
MCREST      0.00000000 sec
MCWRK       0.01500000 sec

======== CHANNEL f1 ========
NUC1                1H
P1                9.75 usec
PL1               1.60 dB
SFO1       500.2235015 MHz

F2 - Processing parameters
SI               65536
SF         500.2200318 MHz
WDW                 EM
SSB                  0
LB                0.30 Hz
GB                   0
PC                1.00

1D NMR plot parameters
CX               22.80 cm
CY               50.00 cm
F1P              9.000 ppm
F1             4501.98 Hz
F2P             -0.500 ppm
F2             -250.11 Hz
PPMCM          0.41667 ppm/cm
HZCM         208.42502 Hz/cm
```

1H spectrum

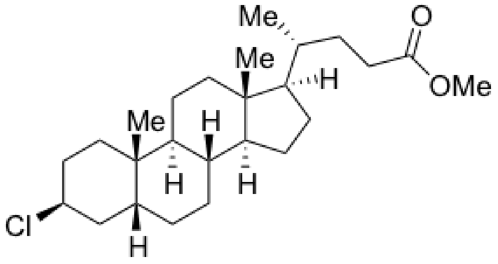

68

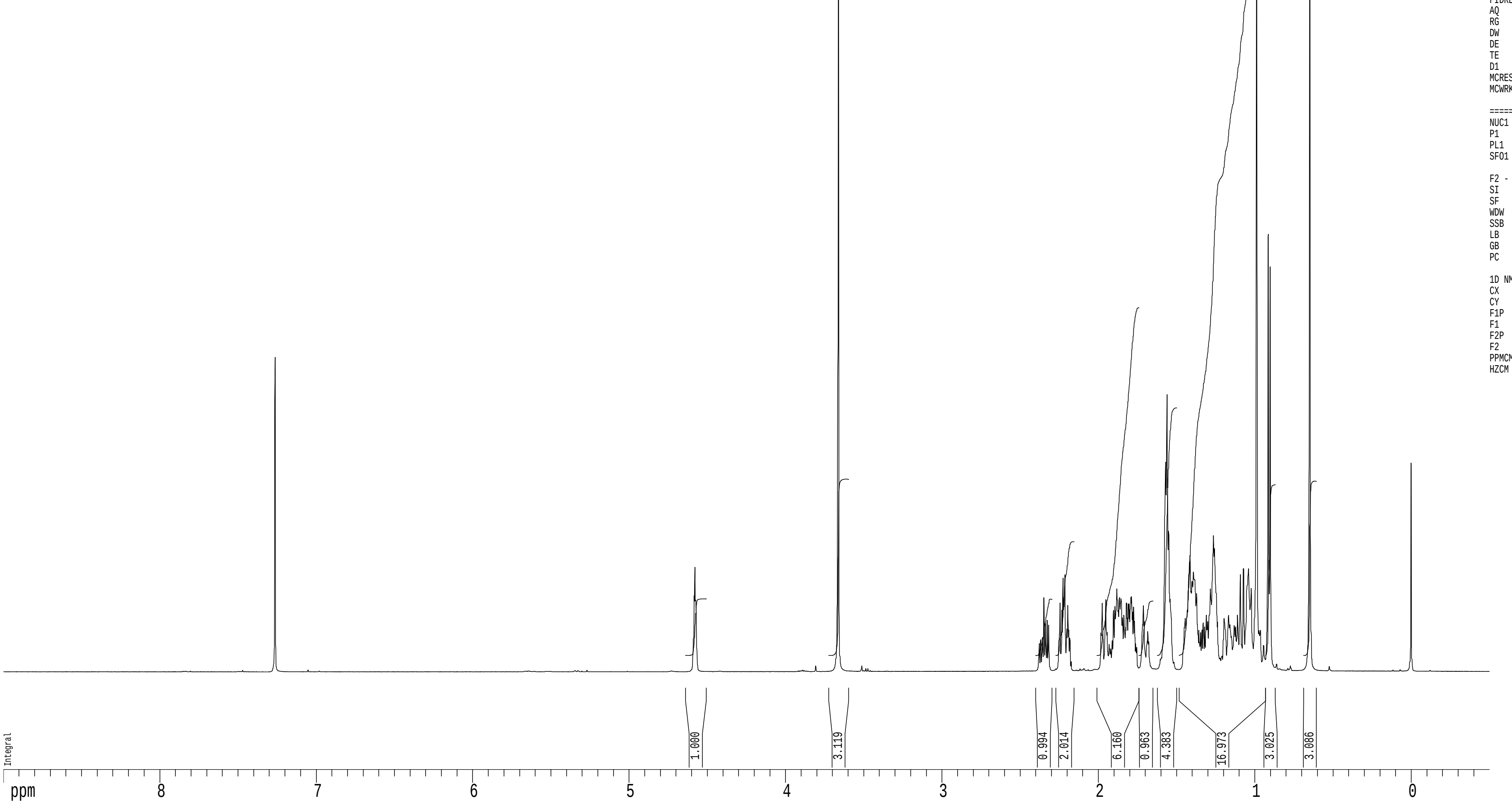


```
Current Data Parameters
USER          nhirbawi
NAME     NH-3-46-HCCOSY-correct
EXPNO               1
PROCNO              1

F2 - Acquisition Parameters
Date_          20220514
Time              14.24
INSTRUM         cryo500
PROBHD   5 mm CPTCI 1H-
PULPROG            zg30
TD                81728
SOLVENT           CDCl3T
NS                    8
DS                    2
SWH           8012.820 Hz
FIDRES        0.098043 Hz
AQ           5.0998774 sec
RG                  5.7
DW               62.400 usec
DE                 6.00 usec
TE                298.0 K
D1           0.10000000 sec
MCREST       0.00000000 sec
MCWRK        0.01500000 sec

======== CHANNEL f1 ========
NUC1                 1H
P1                 9.75 usec
PL1                1.60 dB
SFO1        500.2235015 MHz

F2 - Processing parameters
SI                65536
SF          500.2200310 MHz
WDW                  EM
SSB                   0
LB                 0.30 Hz
GB                    0
PC                 1.00

1D NMR plot parameters
CX                22.80 cm
CY                15.00 cm
F1P               9.000 ppm
F1              4501.98 Hz
F2P              -0.500 ppm
F2              -250.11 Hz
PPMCM           0.41667 ppm/cm
HZCM          208.42502 Hz/cm
```

Z-restored spin-echo 13C spectrum with 1H decoupling

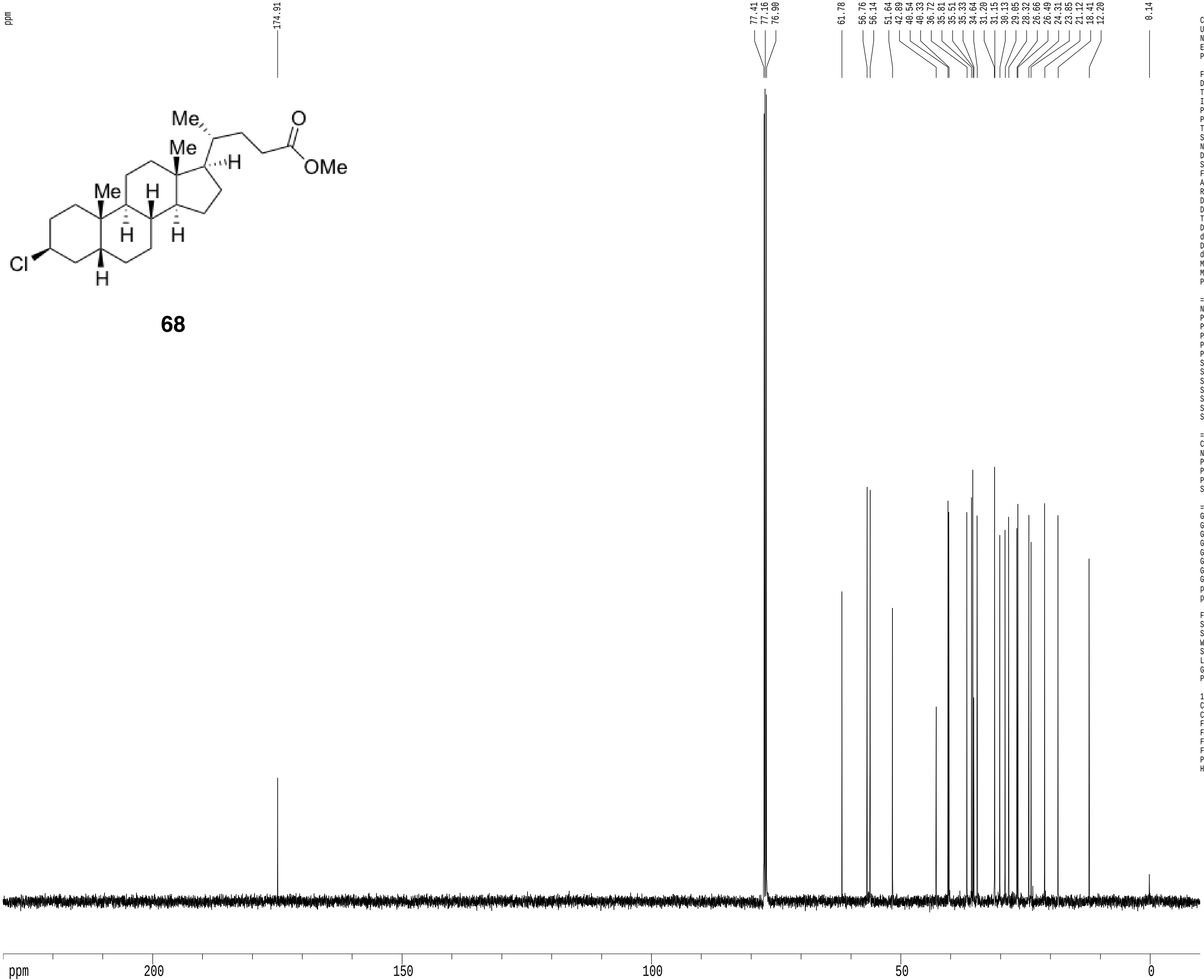

68

Current Data Parameters
USER nhirbawi
NAME NH-3-46-HCCOSY-correct
EXPNO 2
PROCNO 1

F2 - Acquisition Parameters
Date_ 20220514
Time 14.26
INSTRUM cryo500
PROBHD 5 mm CPTCI 1H-
PULPROG SpinEchopg30gp2.prd
TD 65536
SOLVENT CDCl3
NS 626
DS 16
SWH 30303.031 Hz
FIDRES 0.462388 Hz
AQ 1.0813940 sec
RG 3251
DW 16.500 usec
DE 6.00 usec
TE 298.0 K
D1 0.25000000 sec
d11 0.03000000 sec
D16 0.00020000 sec
d17 0.00019600 sec
MCREST 0.00000000 sec
MCWRK 0.01500000 sec
P2 37.70 usec

======== CHANNEL f1 ========
NUC1 13C
P1 18.85 usec
P12 2000.00 usec
P20 500.00 usec
PL0 120.00 dB
PL1 -1.00 dB
SFO1 125.7942548 MHz
SP2 1.55 dB
SP4 1.55 dB
SPNAM2 Crp60comp.4
SPNAM4 Crp60,0.5,20.1
SPOFF2 0.00 Hz
SPOFF4 0.00 Hz

======== CHANNEL f2 ========
CPDPRG2 waltz16
NUC2 1H
PCPD2 100.00 usec
PL2 1.60 dB
PL12 22.00 dB
SFO2 500.2225011 MHz

====== GRADIENT CHANNEL =====
GPNAM1 SINE.100
GPNAM2 SINE.100
GPX1 0.00 %
GPX2 0.00 %
GPY1 0.00 %
GPY2 0.00 %
GPZ1 30.00 %
GPZ2 50.00 %
p15 500.00 usec
p16 1000.00 usec

F2 - Processing parameters
SI 65536
SF 125.7804080 MHz
WDW EM
SSB 0
LB 1.00 Hz
GB 0
PC 2.00

1D NMR plot parameters
CX 22.80 cm
CY 15.65 cm
F1P 230.000 ppm
F1 28929.49 Hz
F2P -10.000 ppm
F2 -1257.80 Hz
PPMCM 10.52632 ppm/cm
HZCM 1324.00439 Hz/cm

gcosy60

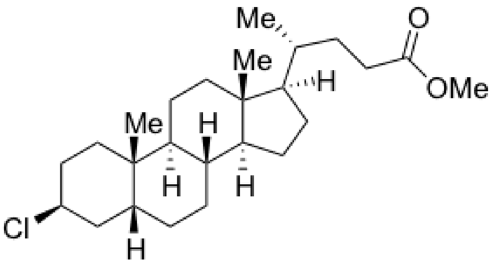

68

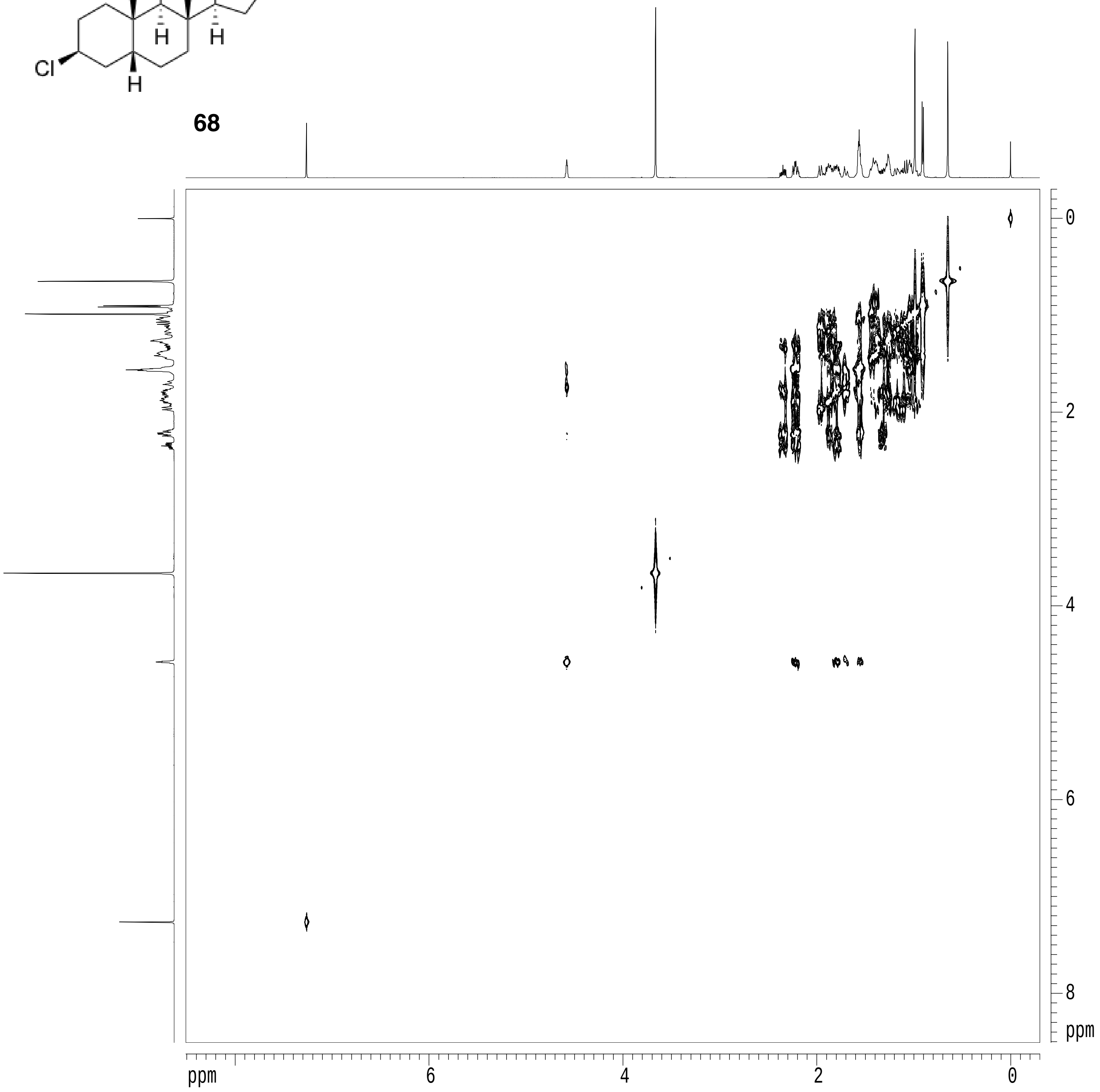

Current Data Parameters
USER nhirbawi
NAME NH-3-46-HCCOSY-correct
EXPNO 3
PROCNO 1

F2 - Acquisition Parameters
Date_ 20220514
Time 14.40
INSTRUM cryo500
PROBHD 5 mm CPTCI 1H-
PULPROG cosygp60.prd
TD 2048
SOLVENT CDCl3T
NS 2
DS 16
SWH 4562.044 Hz
FIDRES 2.227561 Hz
AQ 0.2245108 sec
RG 57
DW 109.600 usec
DE 6.00 usec
TE 298.0 K
d0 0.00000300 sec
D1 1.00000000 sec
d13 0.00000300 sec
D16 0.00020000 sec
IN0 0.00021920 sec

======== CHANNEL f1 ========
NUC1 1H
P1 9.75 usec
PL1 1.60 dB
SFO1 500.2221219 MHz

====== GRADIENT CHANNEL =====
GPNAM1 SMSQ10.100
GPNAM2 SMSQ10.100
GPX1 0.00 %
GPX2 0.00 %
GPY1 0.00 %
GPY2 0.00 %
GPZ1 17.00 %
GPZ2 17.00 %
P16 1000.00 usec

F1 - Acquisition parameters
ND0 1
TD 179
SFO1 500.2221 MHz
FIDRES 25.486279 Hz
SW 9.120 ppm
FnMODE QF

F2 - Processing parameters
SI 1024
SF 500.2200310 MHz
WDW SINE
SSB 0
LB 0.00 Hz
GB 0
PC 1.00

F1 - Processing parameters
SI 1024
MC2 QF
SF 500.2200310 MHz
WDW SINE
SSB 0
LB 0.00 Hz
GB 0

2D NMR plot parameters
CX2 15.00 cm
CX1 15.00 cm
F2PLO 8.508 ppm
F2LO 4256.02 Hz
F2PHI -0.300 ppm
F2HI -150.10 Hz
F1PLO 8.508 ppm
F1LO 4256.02 Hz
F1PHI -0.300 ppm
F1HI [illegible] Hz
F2PPMCM 0.58722 ppm/cm
F2HZCM 293.74100 Hz/cm
F1PPMCM 0.58722 ppm/cm
F1HZCM 293.74100 Hz/cm

1H spectrum

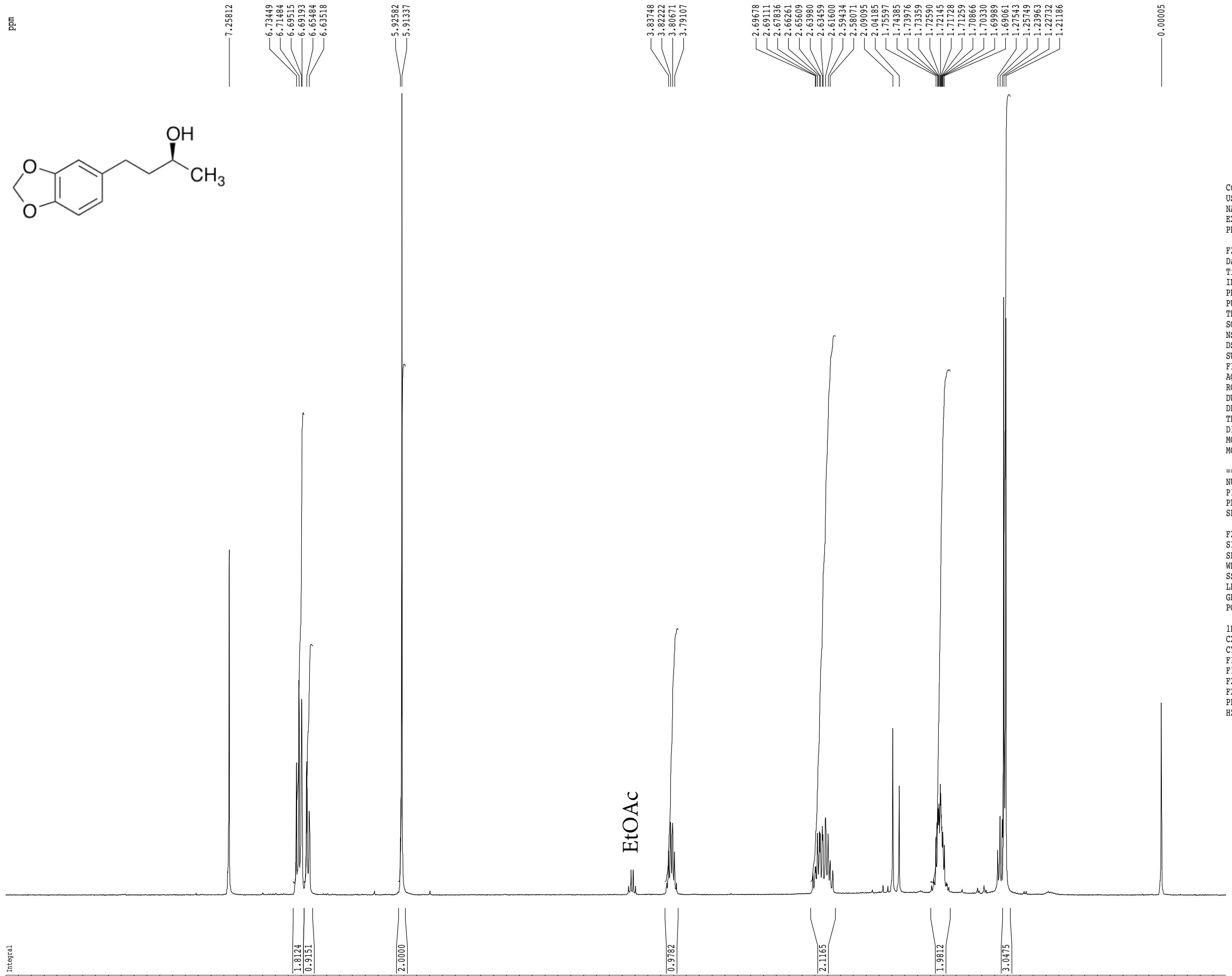


```
Current Data Parameters
USER          linpc2
NAME          pcl-2-280
EXPNO         3
PROCNO        1

F2 - Acquisition Parameters
Date_         20220103
Time          15.34
INSTRUM       drx400
PROBHD   5 mm QNP H/F/P
PULPROG       zg30
TD            65536
SOLVENT       CDCl3
NS            8
DS            2
SWH           6410.256 Hz
FIDRES        0.097813 Hz
AQ            5.1118579 sec
RG            406.4
DW            78.000 usec
DE            4.50 usec
TE            298.0 K
D1            0.10000000 sec
MCREST        0.00000000 sec
MCWRK         0.01500000 sec

======== CHANNEL f1 ========
NUC1          1H
P1            12.00 usec
PL1           -0.90 dB
SFO1          400.1328009 MHz

F2 - Processing parameters
SI            65536
SF            400.1300218 MHz
WDW           EM
SSB           0
LB            0.30 Hz
GB            0
PC            2.00

1D NMR plot parameters
CX            22.80 cm
CY            15.00 cm
F1P           9.000 ppm
F1            3601.17 Hz
F2P           -0.500 ppm
F2            -200.06 Hz
PPMCM         0.41667 ppm/cm
HZCM          166.72086 Hz/cm
```

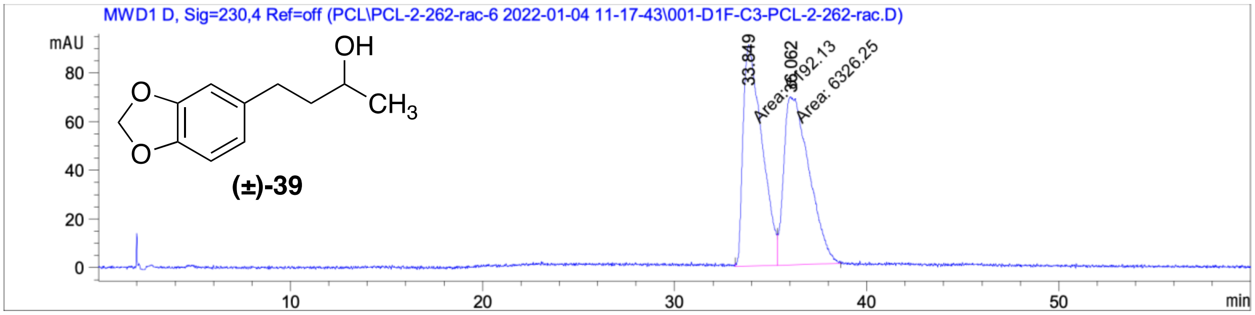

Signal 4: MWD1 D, Sig=230,4 Ref=off

Peak #	RetTime [min]	Type	Width [min]	Area [mAU*s]	Height [mAU]	Area %
1	33.849	MF	1.1366	6192.12646	90.79979	49.4643
2	36.062	FM	1.5297	6326.24756	68.92910	50.5357
Totals :				1.25184e4	159.72889	

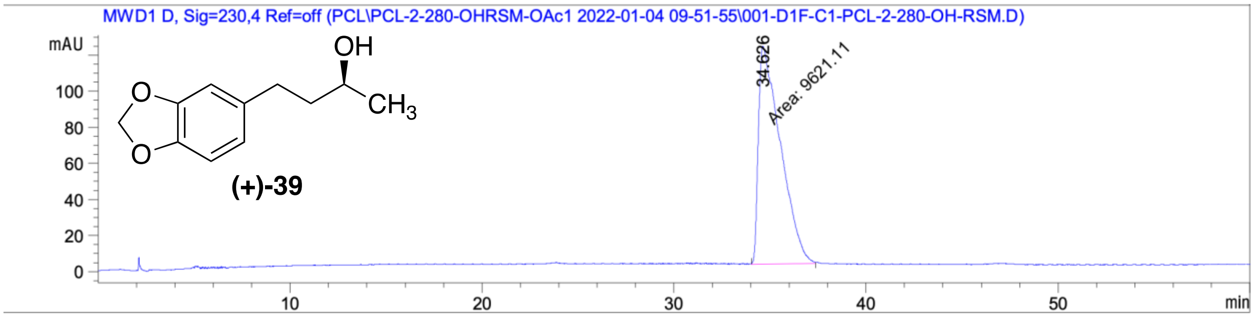

Signal 3: MWD1 C, Sig=210,4 Ref=off

Peak #	RetTime [min]	Type	Width [min]	Area [mAU*s]	Height [mAU]	Area %
1	0.319	BV	0.2466	37.60947	1.86995	100.0000
Totals :				37.60947	1.86995	

1H spectrum

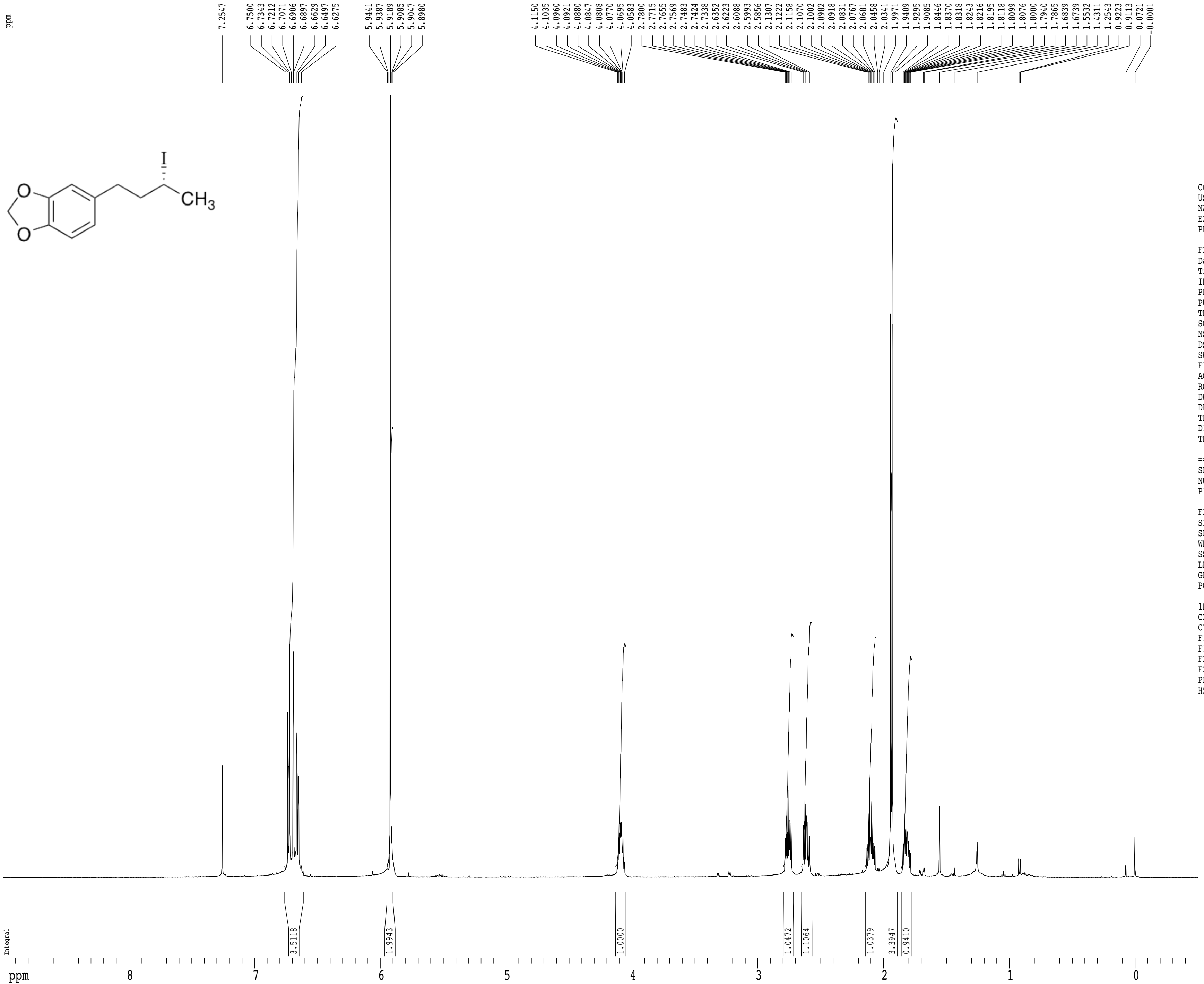

Current Data Parameters
USER linpc2
NAME pcl-2-286
EXPNO 2
PROCNO 1

F2 - Acquisition Parameters
Date_ 20220107
Time 8.27
INSTRUM av600
PROBHD 5 mm CPBBO BB-
PULPROG zg30
TD 98074
SOLVENT CDCl3T
NS 8
DS 2
SWH 9615.385 Hz
FIDRES 0.098042 Hz
AQ 5.0998979 sec
RG 10
DW 52.000 usec
DE 14.23 usec
TE 298.0 K
D1 0.10000000 sec
TD0 1

======== CHANNEL f1 ========
SFO1 600.1342009 MHz
NUC1 1H
P1 9.50 usec

F2 - Processing parameters
SI 65536
SF 600.1300378 MHz
WDW no
SSB 0
LB 0.00 Hz
GB 0
PC 1.00

1D NMR plot parameters
CX 22.80 cm
CY 15.00 cm
F1P 9.000 ppm
F1 5401.17 Hz
F2P -0.500 ppm
F2 -300.06 Hz
PPMCM 0.41667 ppm/cm
HZCM 250.05420 Hz/cm

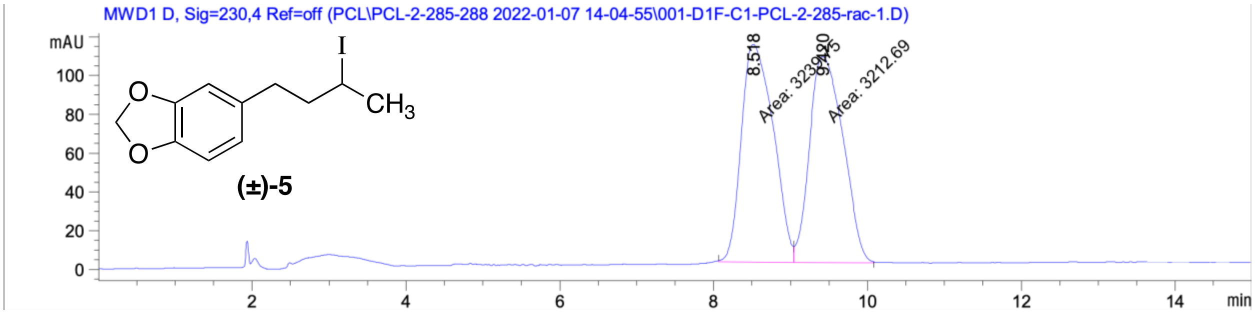

Signal 4: MWD1 D, Sig=230,4 Ref=off

Peak #	RetTime [min]	Type	Width [min]	Area [mAU*s]	Height [mAU]	Area %
1	8.518	MF	0.4808	3239.74780	112.31530	50.2097
2	9.420	FM	0.5047	3212.68896	106.08627	49.7903
Totals :				6452.43677	218.40157	

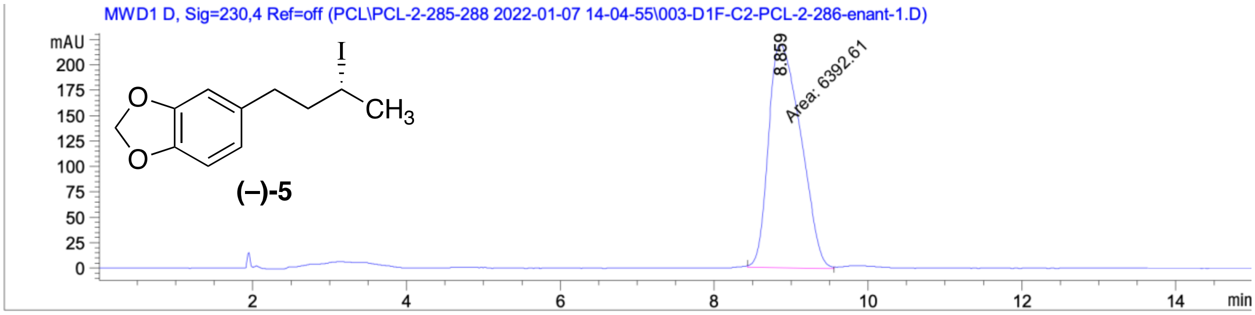

Signal 4: MWD1 D, Sig=230,4 Ref=off

Peak #	RetTime [min]	Type	Width [min]	Area [mAU*s]	Height [mAU]	Area %
1	8.859	MM	0.4844	6392.60986	219.95943	100.0000
Totals :				6392.60986	219.95943	

1H spectrum

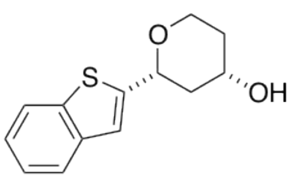

***cis*-SI-10**

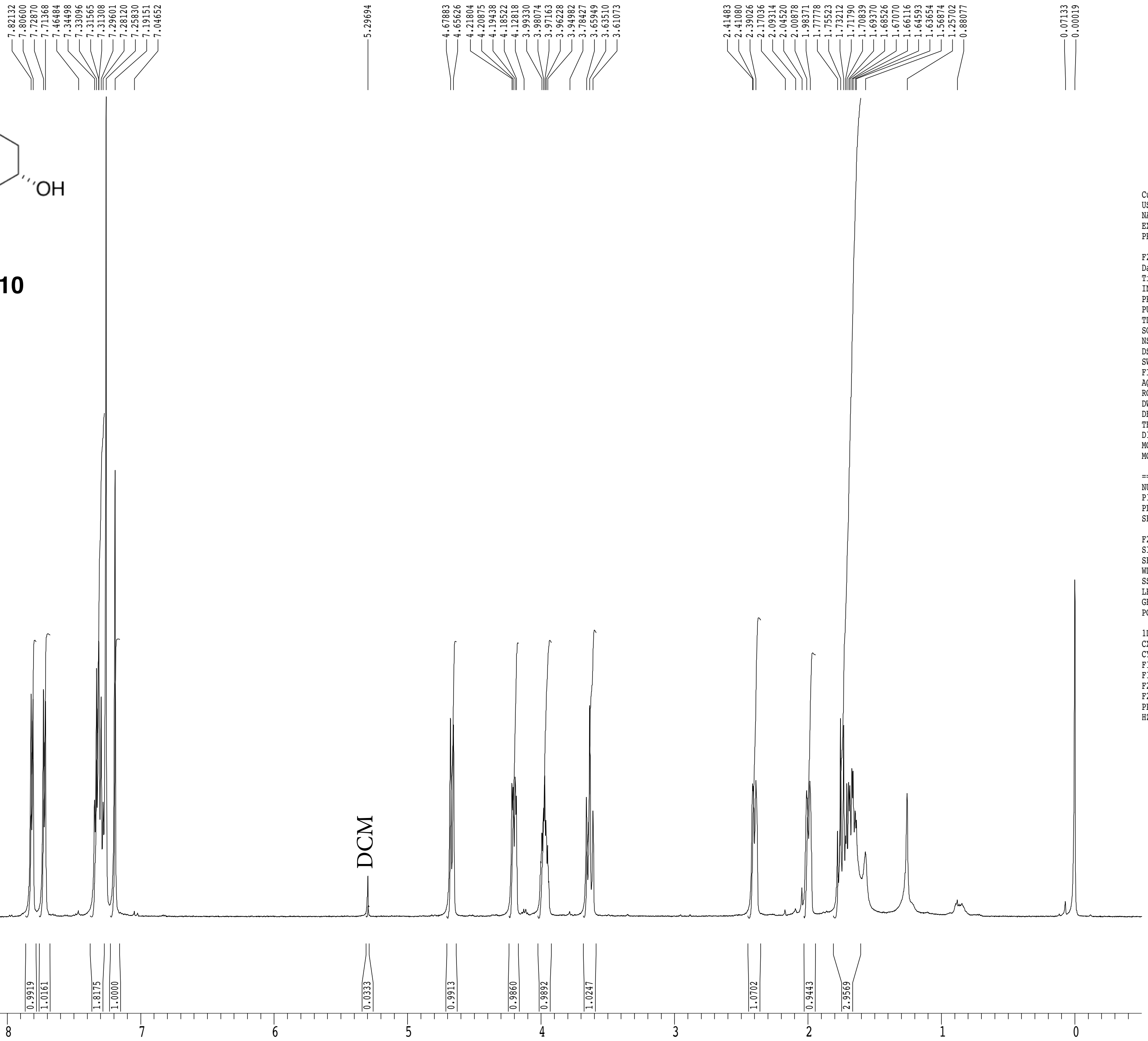


```
Current Data Parameters
USER              linpc2
NAME     pcl-3-108-cis-COSY
EXPNO                  1
PROCNO                 1

F2 - Acquisition Parameters
Date_          20220426
Time              15.10
INSTRUM         cryo500
PROBHD   5 mm CPTCI 1H-
PULPROG            zg30
TD                48074
SOLVENT           CDCl3
NS                    8
DS                    2
SWH            8012.820 Hz
FIDRES         0.166677 Hz
AQ            2.9998677 sec
RG                  5.7
DW               62.400 usec
DE                 6.00 usec
TE                298.0 K
D1           0.10000000 sec
MCREST       0.00000000 sec
MCWRK        0.01500000 sec

======== CHANNEL f1 ========
NUC1                 1H
P1                 9.75 usec
PL1                1.60 dB
SFO1        500.2235015 MHz

F2 - Processing parameters
SI                65536
SF          500.2200326 MHz
WDW                  EM
SSB                   0
LB                 0.30 Hz
GB                    0
PC                 1.00

1D NMR plot parameters
CX                22.80 cm
CY                15.00 cm
F1P               9.000 ppm
F1              4501.98 Hz
F2P              -0.500 ppm
F2              -250.11 Hz
PPMCM           0.41667 ppm/cm
HZCM          208.42502 Hz/cm
```

Z-restored spin-echo 13C spectrum with 1H decoupling

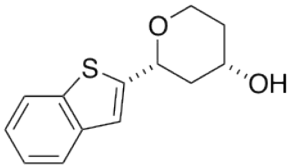

cis-SI-10

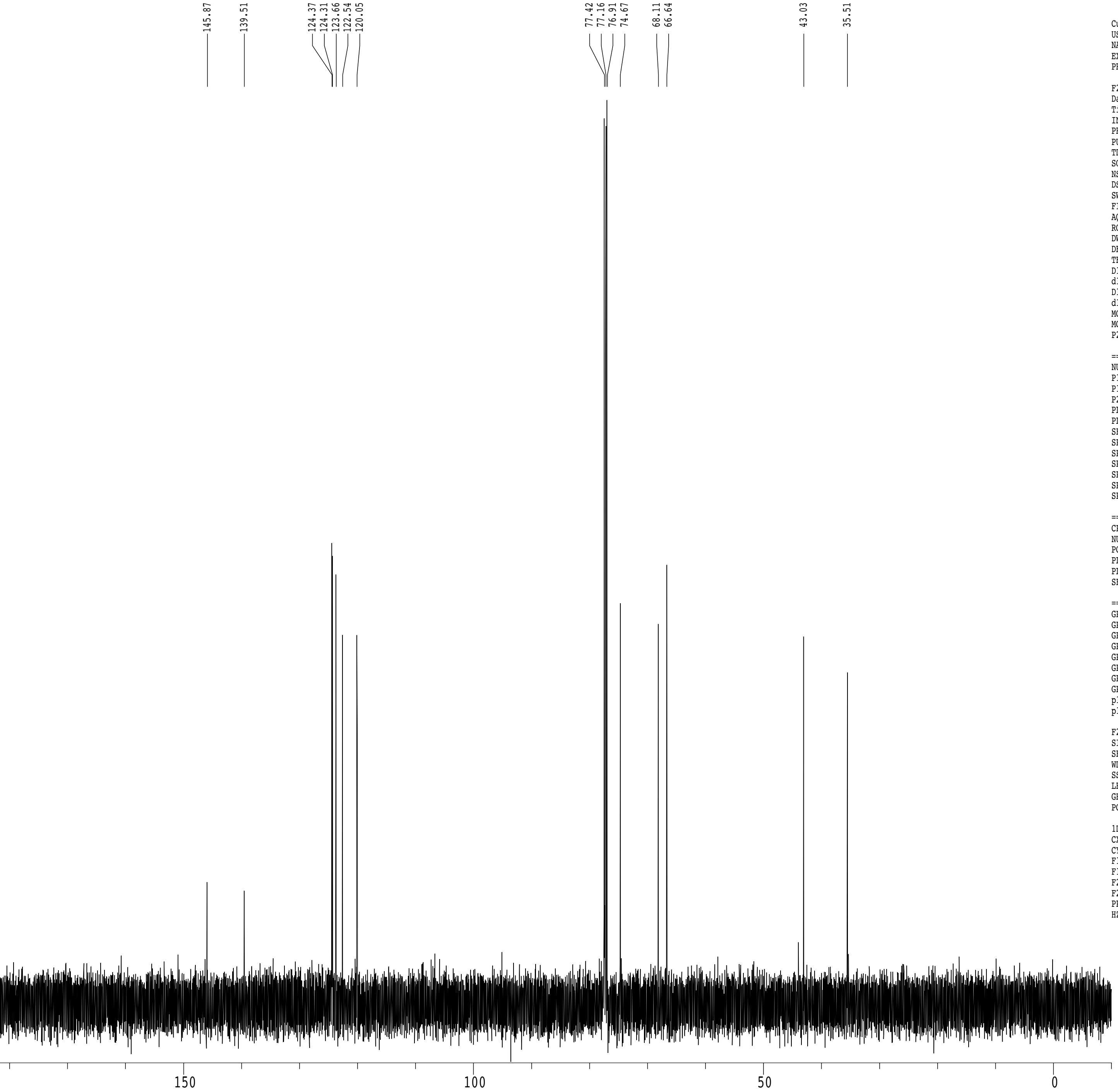

Current Data Parameters
USER linpc2
NAME pcl-3-108-cis-noe
EXPNO 4
PROCNO 1

F2 - Acquisition Parameters
Date_ 20220427
Time 8.20
INSTRUM cryo500
PROBHD 5 mm CPTCI 1H-
PULPROG SpinEchopg30gp2.prd
TD 65536
SOLVENT CDCl3
NS 152
DS 16
SWH 30303.031 Hz
FIDRES 0.462388 Hz
AQ 1.0813940 sec
RG 2580.3
DW 16.500 usec
DE 6.00 usec
TE 298.0 K
D1 0.25000000 sec
d11 0.03000000 sec
D16 0.00020000 sec
d17 0.00019600 sec
MCREST 0.00000000 sec
MCWRK 0.01500000 sec
P2 37.70 usec

======== CHANNEL f1 ========
NUC1 13C
P1 18.85 usec
P12 2000.00 usec
P20 500.00 usec
PL0 120.00 dB
PL1 -1.00 dB
SFO1 125.7942548 MHz
SP2 1.55 dB
SP4 1.55 dB
SPNAM2 Crp60comp.4
SPNAM4 Crp60,0.5,20.1
SPOFF2 0.00 Hz
SPOFF4 0.00 Hz

======== CHANNEL f2 ========
CPDPRG2 waltz16
NUC2 1H
PCPD2 100.00 usec
PL2 1.60 dB
PL12 22.00 dB
SFO2 500.2225011 MHz

====== GRADIENT CHANNEL =====
GPNAM1 SINE.100
GPNAM2 SINE.100
GPX1 0.00 %
GPX2 0.00 %
GPY1 0.00 %
GPY2 0.00 %
GPZ1 30.00 %
GPZ2 50.00 %
p15 500.00 usec
p16 1000.00 usec

F2 - Processing parameters
SI 65536
SF 125.7804085 MHz
WDW no
SSB 0
LB 0.00 Hz
GB 0
PC 2.00

1D NMR plot parameters
CX 22.80 cm
CY 15.65 cm
F1P 220.000 ppm
F1 27671.69 Hz
F2P -10.000 ppm
F2 -1257.80 Hz
PPMCM 10.08772 ppm/cm
HZCM 1268.83752 Hz/cm

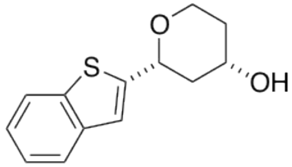

gcosy60

***cis*-SI-10**

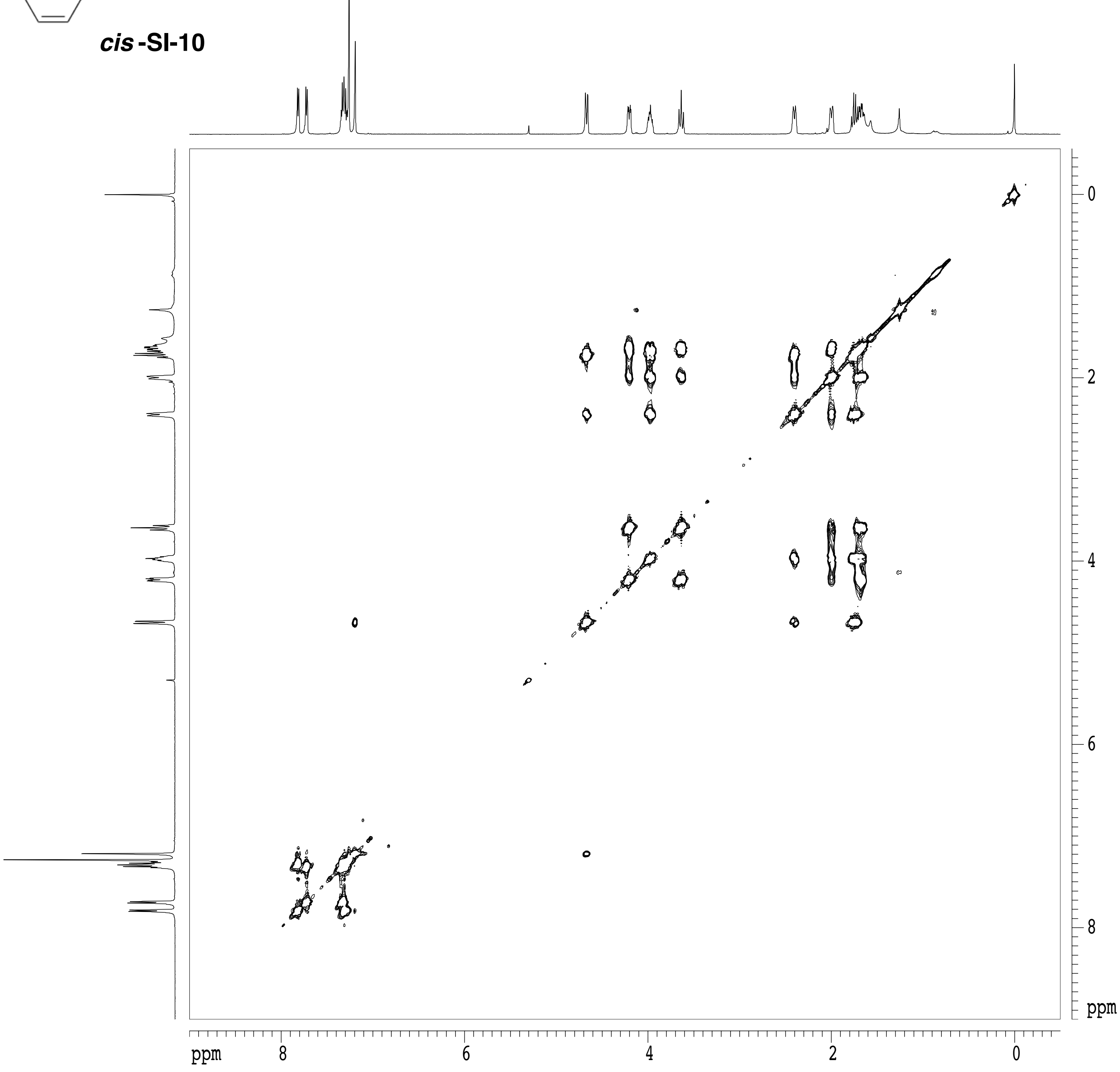


```
       Current Data Parameters
USER               linpc2
NAME     pcl-3-108-cis-COSY
EXPNO                   2
PROCNO                  1

     F2 - Acquisition Parameters
Date_            20220426
Time                15.13
INSTRUM           cryo500
PROBHD   5 mm CPTCI 1H-
PULPROG    cosygp60.prd
TD                   2048
SOLVENT             CDCl3
NS                      1
DS                     16
SWH             8012.820 Hz
FIDRES          3.912510 Hz
AQ             0.1278452 sec
RG                  406.4
DW                 62.400 usec
DE                   6.00 usec
TE                  298.0 K
d0             0.00000300 sec
D1             1.00000000 sec
d13            0.00000300 sec
D16            0.00020000 sec
IN0            0.00012480 sec

 ======== CHANNEL f1 ========
NUC1                    1H
P1                    9.75 usec
PL1                   1.60 dB
SFO1           500.2235015 MHz

 ====== GRADIENT CHANNEL =====
GPNAM1          SMSQ10.100
GPNAM2          SMSQ10.100
GPX1                  0.00 %
GPX2                  0.00 %
GPY1                  0.00 %
GPY2                  0.00 %
GPZ1                 17.00 %
GPZ2                 17.00 %
P16                1000.00 usec

     F1 - Acquisition parameters
ND0                      1
TD                     512
SFO1              500.2235 MHz
FIDRES           15.650040 Hz
SW                  16.018 ppm
FnMODE                  QF

     F2 - Processing parameters
SI                    1024
SF             500.2200326 MHz
WDW                   SINE
SSB                      0
LB                    0.00 Hz
GB                       0
PC                    1.00

     F1 - Processing parameters
SI                    1024
MC2                     QF
SF             500.2200326 MHz
WDW                   SINE
SSB                      0
LB                    0.00 Hz
GB                       0

     2D NMR plot parameters
CX2                  15.00 cm
CX1                  15.00 cm
F2PPLO               9.000 ppm
F2LO               4501.98 Hz
F2PHI               -0.500 ppm
F2HI               -250.11 Hz
F1PPLO               9.000 ppm
F1LO               4501.98 Hz
F1PHI               -0.500 ppm
F1HI           [illegible] Hz
F2PPMCM            0.63333 ppm/cm
F2HZCM           316.80603 Hz/cm
F1PPMCM            0.63333 ppm/cm
F1HZCM           316.80603 Hz/cm
```

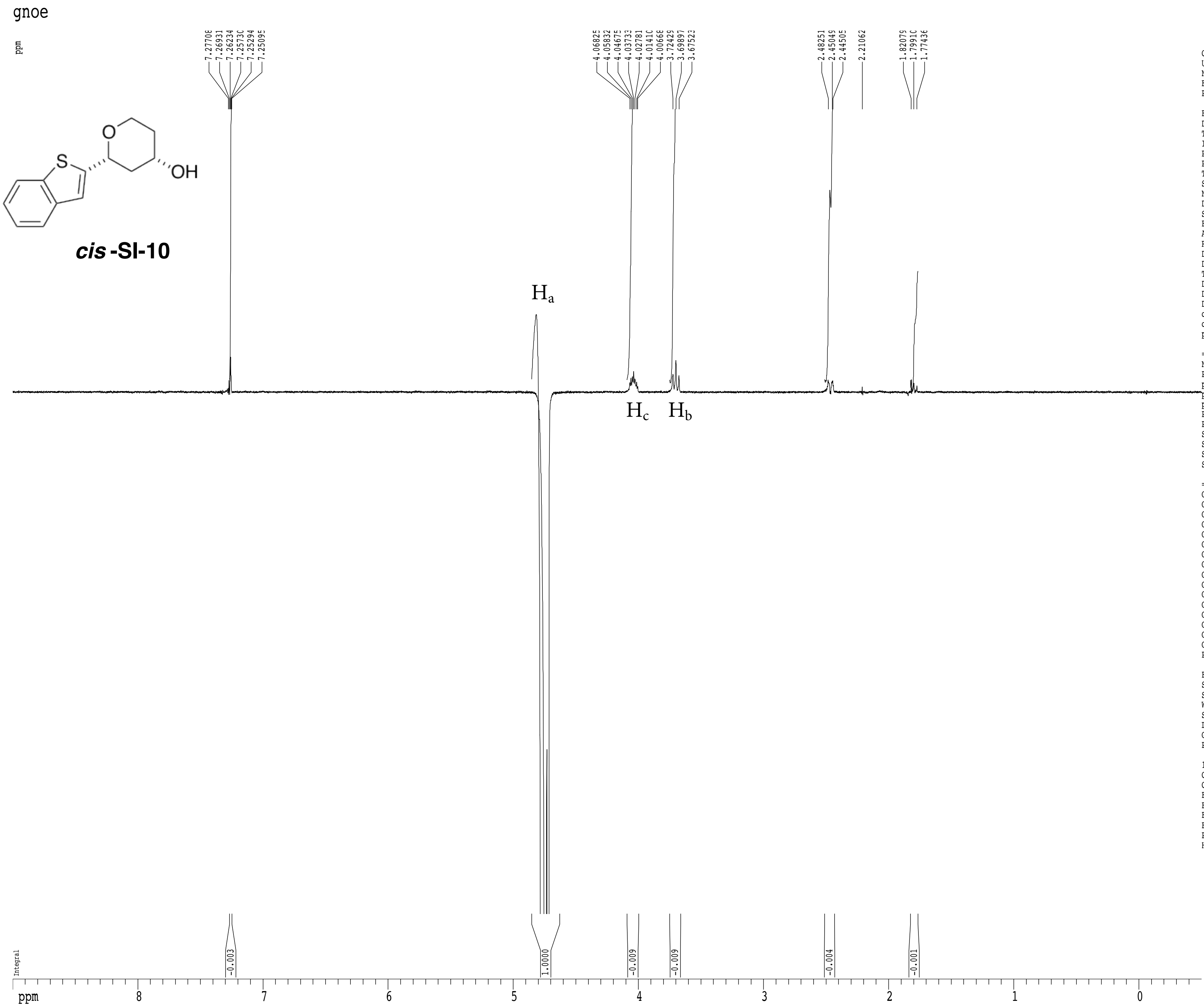


```
Current Data Parameters
USER              linpc2
NAME     pcl-3-108-cis-noe
EXPNO                  2
PROCNO                 1

F2 - Acquisition Parameters
Date_           20220427
Time                7.59
INSTRUM          cryo500
PROBHD   5 mm CPTCI 1H-
PULPROG   gnoe1cc22.prd
TD                 65536
SOLVENT            CDCl3
NS                   128
DS                     8
SWH            8012.820 Hz
FIDRES         0.122266 Hz
AQ            4.0894966 sec
RG                  45.3
DW                62.400 usec
DE                  6.00 usec
TE                 298.0 K
D1            1.00000000 sec
D8            0.50000000 sec
D16           0.00020000 sec
d21           0.33375451 sec
d22           0.16399699 sec
p2                 19.50 usec

======== CHANNEL f1 ========
NUC1                  1H
P1                  9.75 usec
p3                 29.25 usec
p4                 39.00 usec
p5                 26.00 usec
P29             40000.00 usec
PL1                 1.60 dB
SFO1         500.2223670 MHz
SP9                60.00 dB
SPNAM9         gauss1.512
SPOFF9              0.00 Hz

====== GRADIENT CHANNEL =====
GPNAM1       SMSQ10.100
GPNAM2       SMSQ10.100
GPNAM3       SMSQ10.100
GPNAM4       SMSQ10.100
GPX1                0.00 %
GPX2                0.00 %
GPX3                0.00 %
GPX4                0.00 %
GPY1                0.00 %
GPY2                0.00 %
GPY3                0.00 %
GPY4                0.00 %
GPZ1                7.00 %
GPZ2                3.00 %
GPZ3                2.30 %
GPZ4               -2.30 %
P16              1000.00 usec

F2 - Processing parameters
SI                 65536
SF           500.2200000 MHz
WDW                   no
SSB                    0
LB                  0.00 Hz
GB                     0
PC                  1.00

1D NMR plot parameters
CX                 22.80 cm
CY                 50.00 cm
F1P                9.000 ppm
F1               4501.98 Hz
F2P               -0.500 ppm
F2               -250.11 Hz
PPMCM            0.41667 ppm/cm
HZCM           208.42500 Hz/cm
```

gnoe

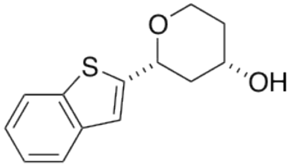

***cis*-SI-10**

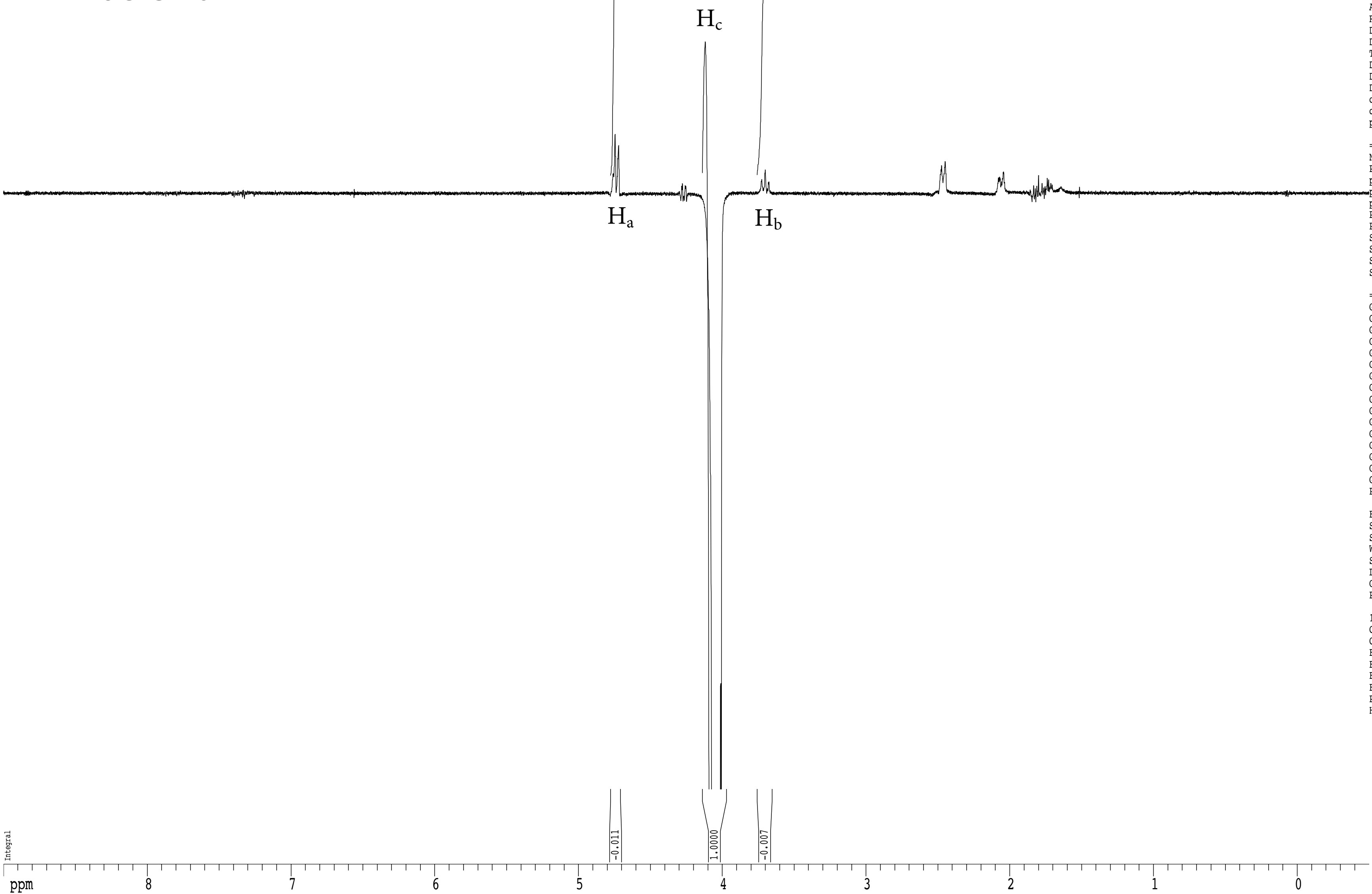

Current Data Parameters
USER linpc2
NAME pcl-3-108-cis-noe
EXPNO 3
PROCNO 1

F2 - Acquisition Parameters
Date_ 20220427
Time 8.16
INSTRUM cryo500
PROBHD 5 mm CPTCI 1H-
PULPROG gnoe1cc22.prd
TD 65536
SOLVENT CDCl3
NS 128
DS 8
SWH 8012.820 Hz
FIDRES 0.122266 Hz
AQ 4.0894966 sec
RG 90.5
DW 62.400 usec
DE 6.00 usec
TE 298.0 K
D1 1.00000000 sec
D8 0.50000000 sec
D16 0.00020000 sec
d21 0.33375451 sec
d22 0.16399699 sec
p2 19.50 usec

======== CHANNEL f1 ========
NUC1 1H
P1 9.75 usec
p3 29.25 usec
p4 39.00 usec
p5 26.00 usec
P29 40000.00 usec
PL1 1.60 dB
SFO1 500.2220187 MHz
SP9 60.00 dB
SPNAM9 gauss1.512
SPOFF9 0.00 Hz

====== GRADIENT CHANNEL =====
GPNAM1 SMSQ10.100
GPNAM2 SMSQ10.100
GPNAM3 SMSQ10.100
GPNAM4 SMSQ10.100
GPX1 0.00 %
GPX2 0.00 %
GPX3 0.00 %
GPX4 0.00 %
GPY1 0.00 %
GPY2 0.00 %
GPY3 0.00 %
GPY4 0.00 %
GPZ1 7.00 %
GPZ2 3.00 %
GPZ3 2.30 %
GPZ4 -2.30 %
P16 1000.00 usec

F2 - Processing parameters
SI 65536
SF 500.2200000 MHz
WDW no
SSB 0
LB 0.00 Hz
GB 0
PC 1.00

1D NMR plot parameters
CX 22.80 cm
CY 50.00 cm
F1P 9.000 ppm
F1 4501.98 Hz
F2P -0.500 ppm
F2 -250.11 Hz
PPMCM 0.41667 ppm/cm
HZCM 208.42500 Hz/cm

1H spectrum

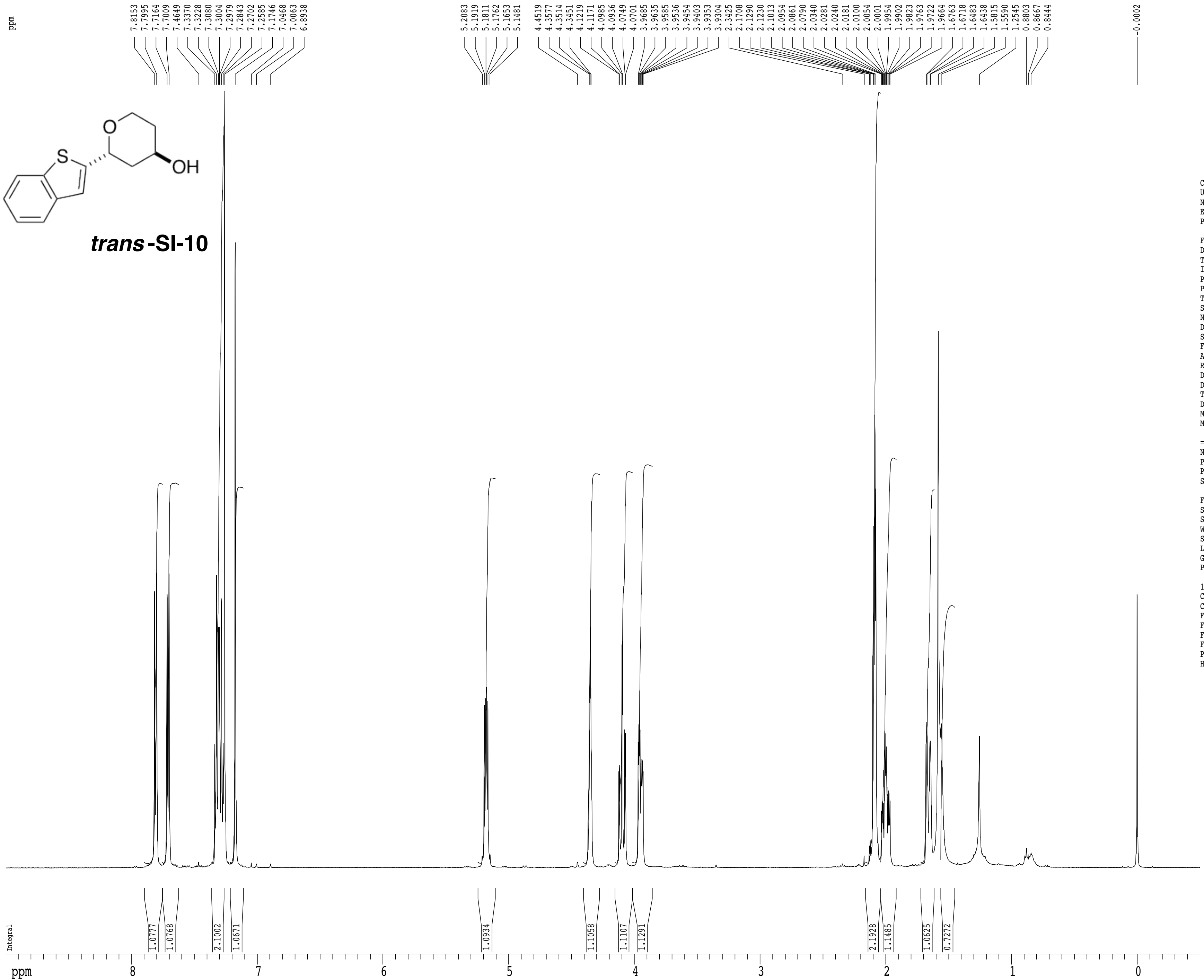

Current Data Parameters
USER linpc2
NAME pcl-3-112-trans-noe
EXPNO 1
PROCNO 1

F2 - Acquisition Parameters
Date_ 20220504
Time 7.52
INSTRUM cryo500
PROBHD 5 mm CPTCI 1H-
PULPROG zg30
TD 81728
SOLVENT CDCl3
NS 8
DS 2
SWH 8012.820 Hz
FIDRES 0.098043 Hz
AQ 5.0998774 sec
RG 11.3
DW 62.400 usec
DE 6.00 usec
TE 298.0 K
D1 0.10000000 sec
MCREST 0.00000000 sec
MCWRK 0.01500000 sec

======== CHANNEL f1 ========
NUC1 1H
P1 9.75 usec
PL1 1.60 dB
SFO1 500.2235015 MHz

F2 - Processing parameters
SI 65536
SF 500.2200329 MHz
WDW EM
SSB 0
LB 0.30 Hz
GB 0
PC 1.00

1D NMR plot parameters
CX 22.80 cm
CY 15.00 cm
F1P 9.000 ppm
F1 4501.98 Hz
F2P -0.500 ppm
F2 -250.11 Hz
PPMCM 0.41667 ppm/cm
HZCM 208.42502 Hz/cm

Z-restored spin-echo 13C spectrum with 1H decoupling

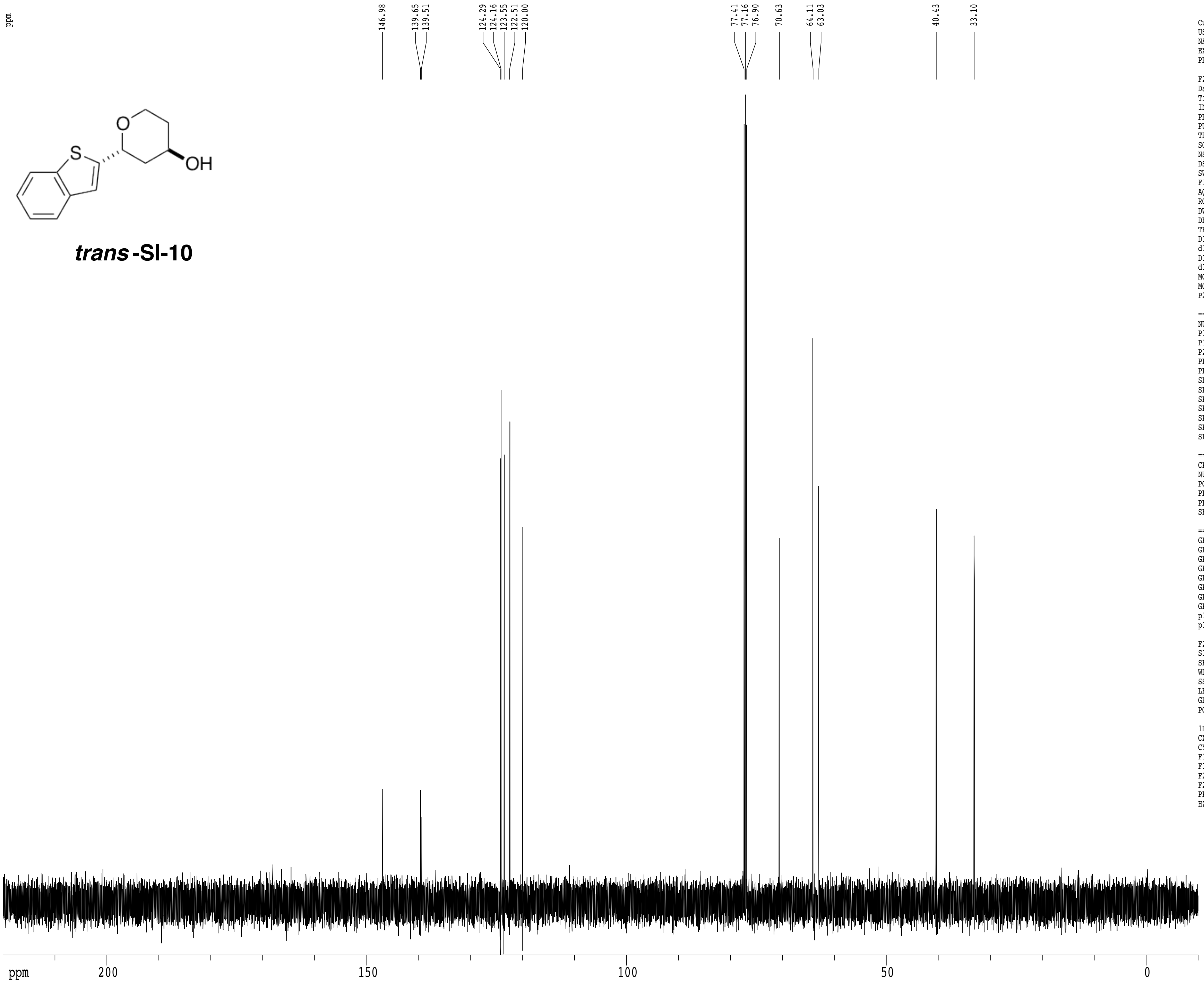

Current Data Parameters
USER linpc2
NAME pcl-3-112-trans-cosy
EXPNO 3
PROCNO 1

F2 - Acquisition Parameters
Date_ 20220504
Time 7.49
INSTRUM cryo500
PROBHD 5 mm CPTCI 1H-
PULPROG SpinEchopg30gp2.prd
TD 65536
SOLVENT CDCl3
NS 136
DS 16
SWH 30303.031 Hz
FIDRES 0.462388 Hz
AQ 1.0813940 sec
RG 2580.3
DW 16.500 usec
DE 6.00 usec
TE 298.0 K
D1 0.25000000 sec
d11 0.03000000 sec
D16 0.00020000 sec
d17 0.00019600 sec
MCREST 0.00000000 sec
MCWRK 0.01500000 sec
P2 37.70 usec

======== CHANNEL f1 ========
NUC1 13C
P1 18.85 usec
P12 2000.00 usec
P20 500.00 usec
PL0 120.00 dB
PL1 -1.00 dB
SFO1 125.7942548 MHz
SP2 1.55 dB
SP4 1.55 dB
SPNAM2 Crp60comp.4
SPNAM4 Crp60,0.5,20.1
SPOFF2 0.00 Hz
SPOFF4 0.00 Hz

======== CHANNEL f2 ========
CPDPRG2 waltz16
NUC2 1H
PCPD2 100.00 usec
PL2 1.60 dB
PL12 22.00 dB
SFO2 500.2225011 MHz

====== GRADIENT CHANNEL =====
GPNAM1 SINE.100
GPNAM2 SINE.100
GPX1 0.00 %
GPX2 0.00 %
GPY1 0.00 %
GPY2 0.00 %
GPZ1 30.00 %
GPZ2 50.00 %
p15 500.00 usec
p16 1000.00 usec

F2 - Processing parameters
SI 65536
SF 125.7804090 MHz
WDW no
SSB 0
LB 0.00 Hz
GB 0
PC 2.00

1D NMR plot parameters
CX 22.80 cm
CY 15.65 cm
F1P 220.000 ppm
F1 27671.69 Hz
F2P -10.000 ppm
F2 -1257.80 Hz
PPMCM 10.08772 ppm/cm
HZCM 1268.83752 Hz/cm

gcosy60

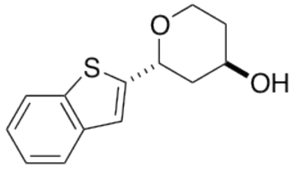

***trans*-SI-10**

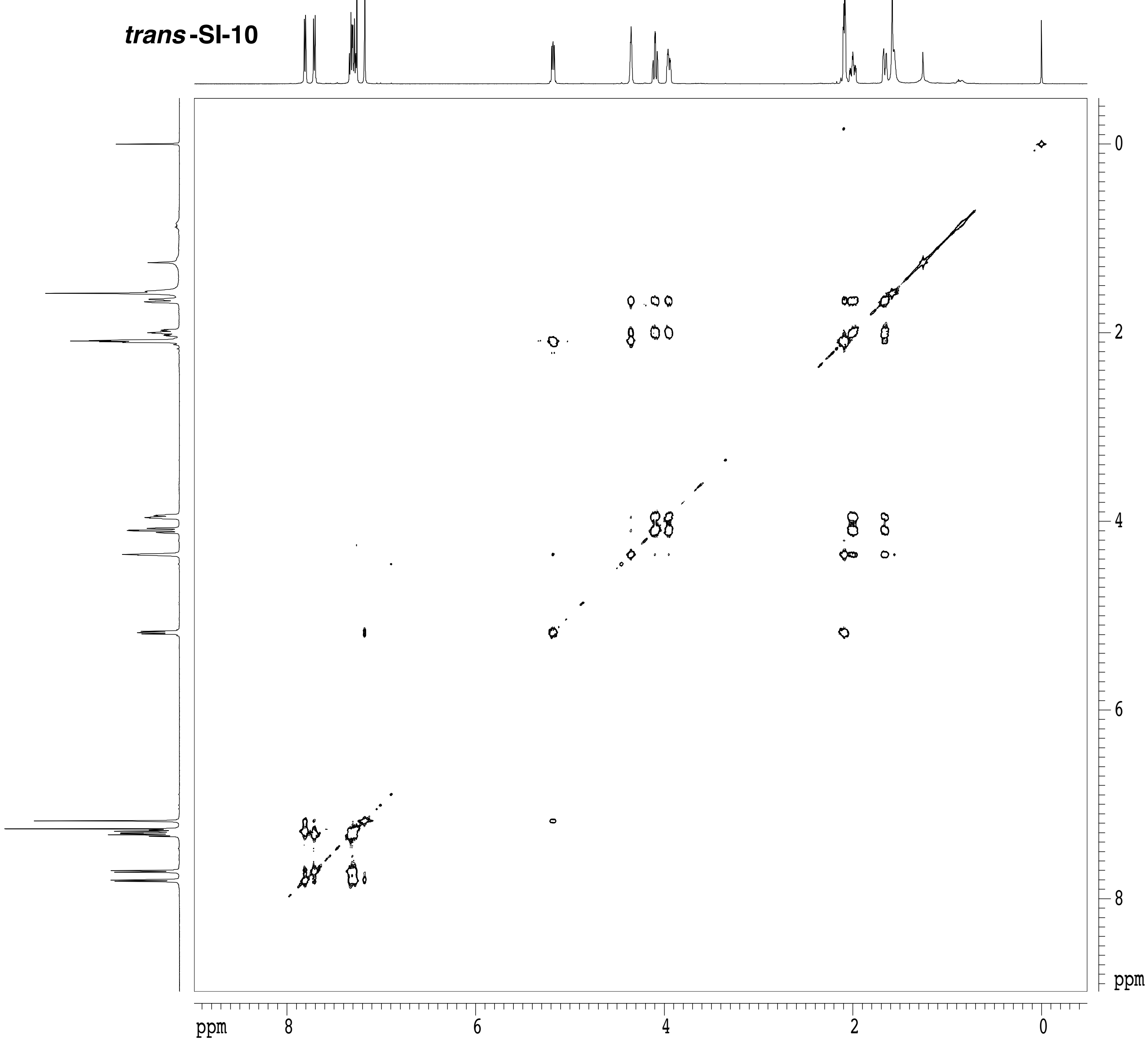


```
         Current Data Parameters
USER                   linpc2
NAME        pcl-3-112-trans-cosy
EXPNO                       2
PROCNO                      1

       F2 - Acquisition Parameters
Date_               20220504
Time                    7.35
INSTRUM              cryo500
PROBHD   5 mm CPTCI 1H-
PULPROG       cosygp60.prd
TD                      2048
SOLVENT                CDCl3
NS                         1
DS                        16
SWH               4734.849 Hz
FIDRES            2.311938 Hz
AQ               0.2163188 sec
RG                       362
DW                   105.600 usec
DE                      6.00 usec
TE                     298.0 K
d0             0.00000300 sec
D1             1.00000000 sec
d13            0.00000300 sec
D16            0.00020000 sec
IN0            0.00021120 sec

======== CHANNEL f1 ========
NUC1                      1H
P1                      9.75 usec
PL1                     1.60 dB
SFO1          500.2221583 MHz

====== GRADIENT CHANNEL =====
GPNAM1          SMSQ10.100
GPNAM2          SMSQ10.100
GPX1                    0.00 %
GPX2                    0.00 %
GPY1                    0.00 %
GPY2                    0.00 %
GPZ1                   17.00 %
GPZ2                   17.00 %
P16                  1000.00 usec

       F1 - Acquisition parameters
ND0                        1
TD                       512
SFO1               500.2222 MHz
FIDRES             9.247751 Hz
SW                     9.465 ppm
FnMODE                    QF

       F2 - Processing parameters
SI                      1024
SF               500.2200324 MHz
WDW                     SINE
SSB                        0
LB                      0.00 Hz
GB                         0
PC                      1.00

       F1 - Processing parameters
SI                      1024
MC2                       QF
SF               500.2200324 MHz
WDW                     SINE
SSB                        0
LB                      0.00 Hz
GB                         0

       2D NMR plot parameters
CX2                    15.00 cm
CX1                    15.00 cm
F2PLO                  8.983 ppm
F2LO                 4493.36 Hz
F2PHI                 -0.483 ppm
F2HI                 -241.49 Hz
F1PLO                  8.983 ppm
F1LO                 4493.36 Hz
F1PHI                 -0.483 ppm
F1HI                 [illegible]
F2PPMCM              0.63104 ppm/cm
F2HZCM             315.65659 Hz/cm
F1PPMCM              0.63104 ppm/cm
F1HZCM             315.65659 Hz/cm
```

gnoe

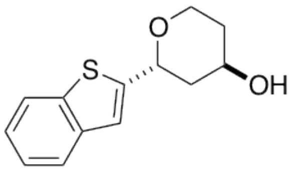

***trans*-SI-10**

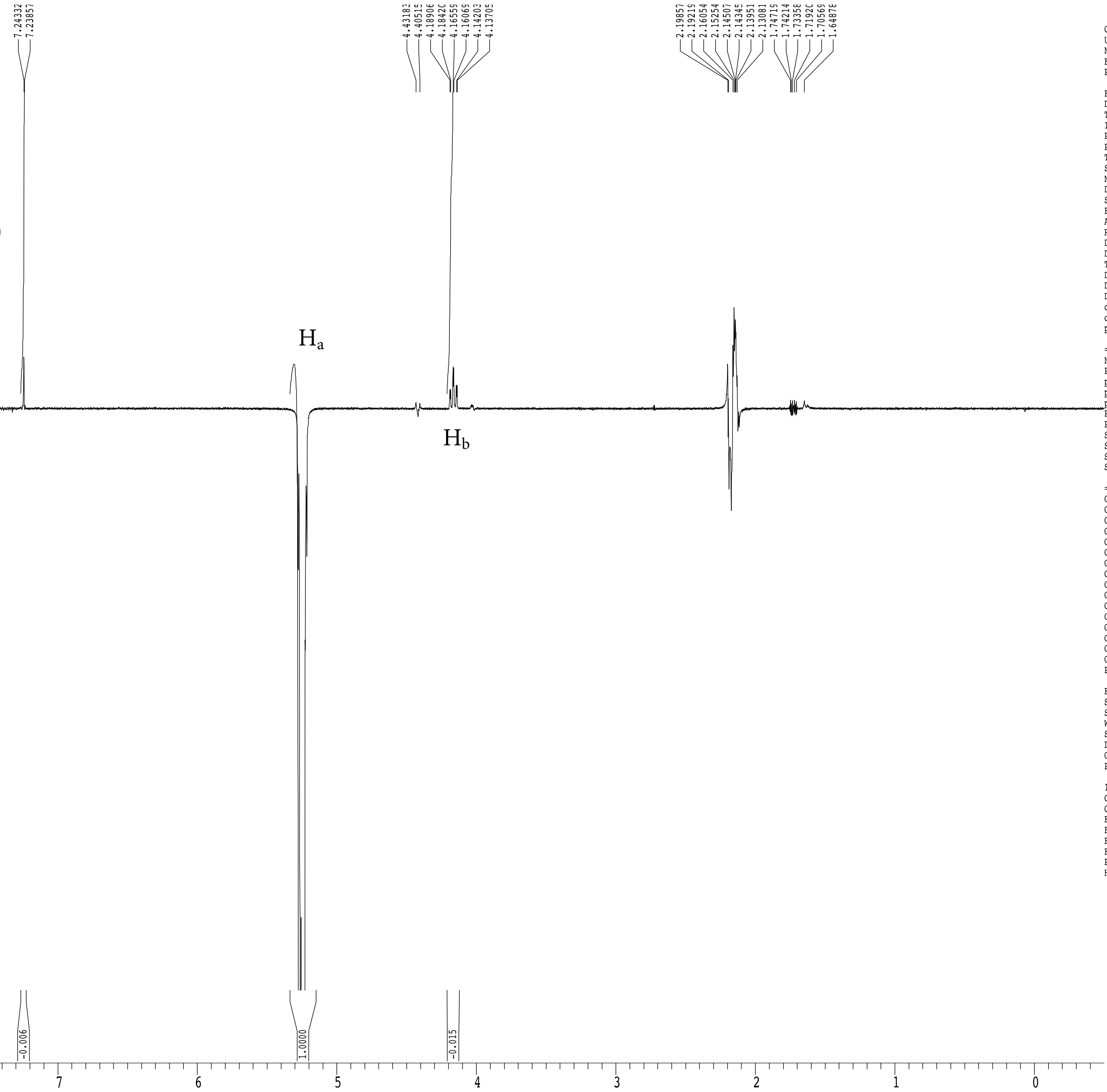

Current Data Parameters
USER linpc2
NAME pcl-3-112-trans-noe
EXPNO 2
PROCNO 1

F2 - Acquisition Parameters
Date_ 20220504
Time 8.08
INSTRUM cryo500
PROBHD 5 mm CPTCI 1H-
PULPROG gnoe1cc22.prd
TD 65536
SOLVENT CDCl3
NS 128
DS 8
SWH 8012.820 Hz
FIDRES 0.122266 Hz
AQ 4.0894966 sec
RG 90.5
DW 62.400 usec
DE 6.00 usec
TE 298.0 K
D1 1.00000000 sec
D8 0.50000000 sec
D16 0.00020000 sec
d21 0.33375451 sec
d22 0.16399699 sec
p2 19.50 usec

======== CHANNEL f1 ========
NUC1 1H
P1 9.75 usec
p3 29.25 usec
p4 39.00 usec
p5 26.00 usec
P29 40000.00 usec
PL1 1.60 dB
SFO1 500.2226234 MHz
SP9 60.00 dB
SPNAM9 gauss1.512
SPOFF9 0.00 Hz

====== GRADIENT CHANNEL =====
GPNAM1 SMSQ10.100
GPNAM2 SMSQ10.100
GPNAM3 SMSQ10.100
GPNAM4 SMSQ10.100
GPX1 0.00 %
GPX2 0.00 %
GPX3 0.00 %
GPX4 0.00 %
GPY1 0.00 %
GPY2 0.00 %
GPY3 0.00 %
GPY4 0.00 %
GPZ1 7.00 %
GPZ2 3.00 %
GPZ3 2.30 %
GPZ4 -2.30 %
P16 1000.00 usec

F2 - Processing parameters
SI 65536
SF 500.2200000 MHz
WDW no
SSB 0
LB 0.00 Hz
GB 0
PC 1.00

1D NMR plot parameters
CX 22.80 cm
CY 50.00 cm
F1P 9.000 ppm
F1 4501.98 Hz
F2P -0.500 ppm
F2 -250.11 Hz
PPMCM 0.41667 ppm/cm
HZCM 208.42500 Hz/cm

1H spectrum

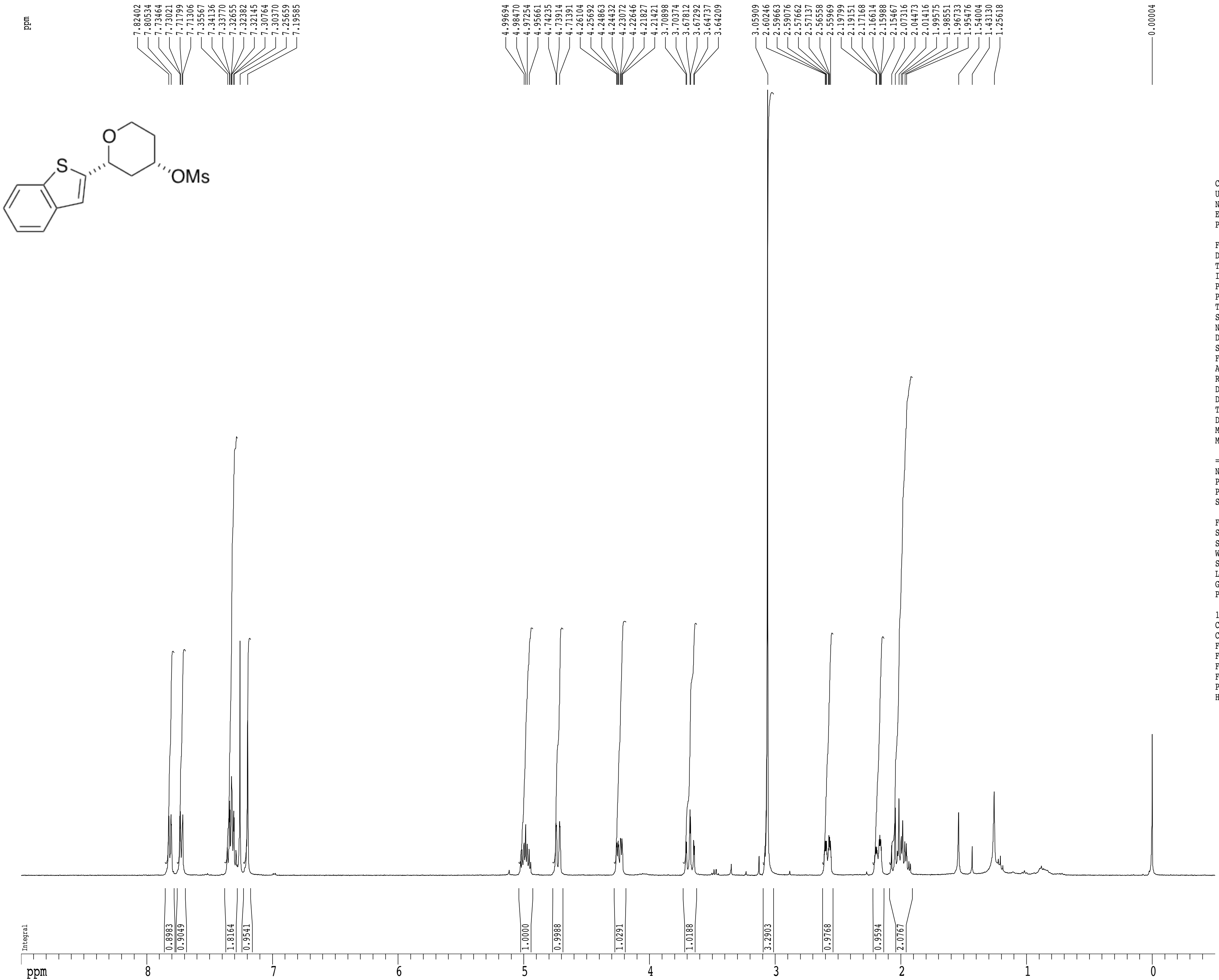

Current Data Parameters
USER linpc2
NAME pcl-3-114
EXPNO 1
PROCNO 1

F2 - Acquisition Parameters
Date_ 20220511
Time 11.13
INSTRUM drx400
PROBHD 5 mm QNP H/F/P
PULPROG zg30
TD 38460
SOLVENT CDCl3
NS 8
DS 2
SWH 6410.256 Hz
FIDRES 0.166673 Hz
AQ 2.9999299 sec
RG 362
DW 78.000 usec
DE 4.50 usec
TE 298.0 K
D1 0.10000000 sec
MCREST 0.00000000 sec
MCWRK 0.01500000 sec

======== CHANNEL f1 ========
NUC1 1H
P1 12.00 usec
PL1 -0.90 dB
SFO1 400.1328009 MHz

F2 - Processing parameters
SI 65536
SF 400.1300224 MHz
WDW EM
SSB 0
LB 0.30 Hz
GB 0
PC 2.00

1D NMR plot parameters
CX 22.80 cm
CY 15.00 cm
F1P 9.000 ppm
F1 3601.17 Hz
F2P -0.500 ppm
F2 -200.06 Hz
PPMCM 0.41667 ppm/cm
HZCM 166.72086 Hz/cm

13C spectrum with 1H decoupling

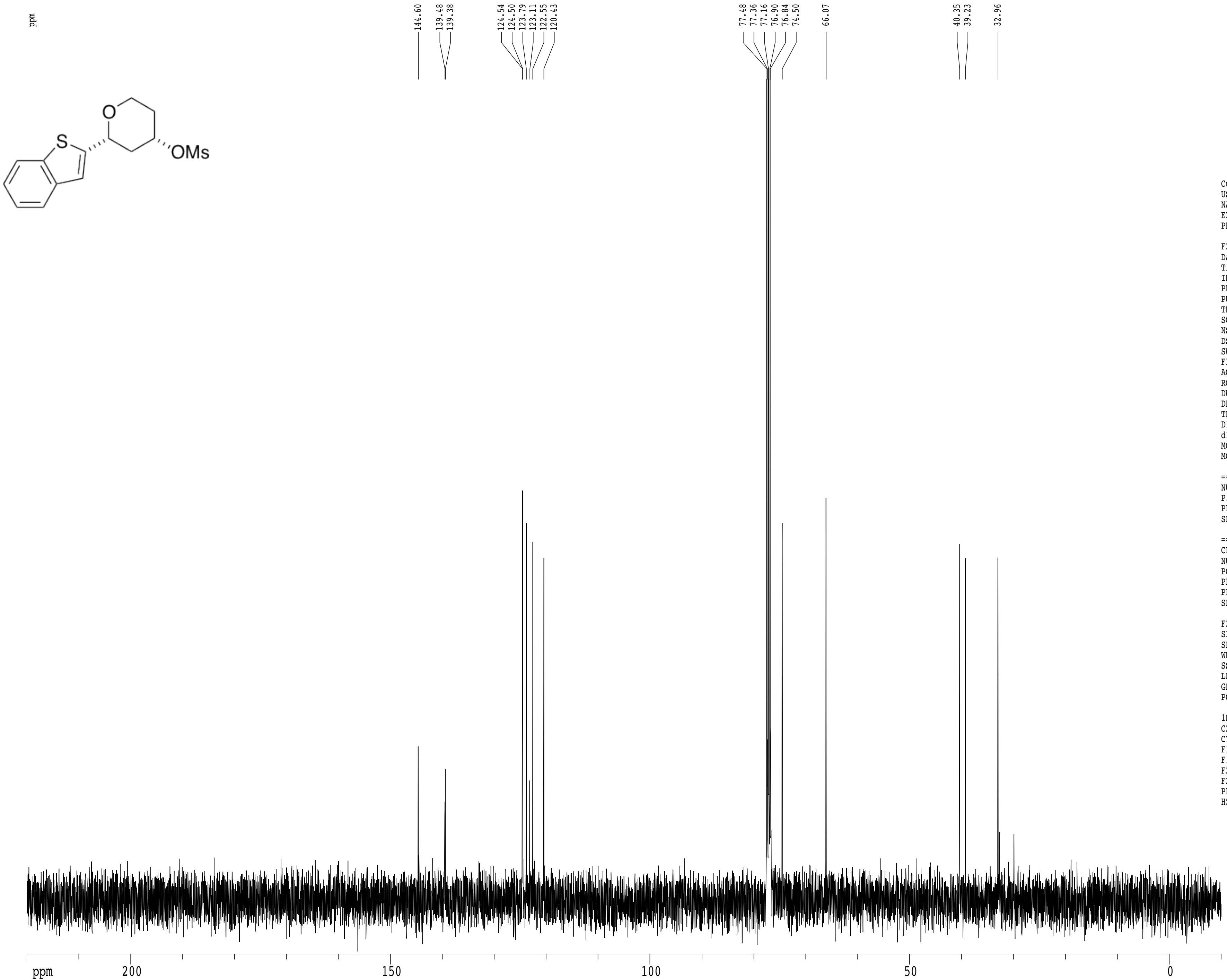

Current Data Parameters
USER linpc2
NAME pcl-3-114
EXPNO 2
PROCNO 1

F2 - Acquisition Parameters
Date_ 20220511
Time 11.15
INSTRUM drx400
PROBHD 5 mm QNP H/F/P
PULPROG zgdc30
TD 65536
SOLVENT CDCl3
NS 592
DS 4
SWH 24154.590 Hz
FIDRES 0.368570 Hz
AQ 1.3566452 sec
RG 16384
DW 20.700 usec
DE 20.39 usec
TE 298.0 K
D1 0.10000000 sec
d11 0.03000000 sec
MCREST 0.00000000 sec
MCWRK 0.01500000 sec

======== CHANNEL f1 ========
NUC1 13C
P1 7.90 usec
PL1 -3.00 dB
SFO1 100.6237964 MHz

======== CHANNEL f2 ========
CPDPRG2 waltz16
NUC2 1H
PCPD2 90.00 usec
PL2 -0.90 dB
PL12 17.00 dB
SFO2 400.1328009 MHz

F2 - Processing parameters
SI 65536
SF 100.6127576 MHz
WDW EM
SSB 0
LB 1.00 Hz
GB 0
PC 1.00

1D NMR plot parameters
CX 22.80 cm
CY 45.00 cm
F1P 220.000 ppm
F1 22134.81 Hz
F2P -10.000 ppm
F2 -1006.13 Hz
PPMCM 10.08772 ppm/cm
HZCM 1014.95325 Hz/cm

1H spectrum

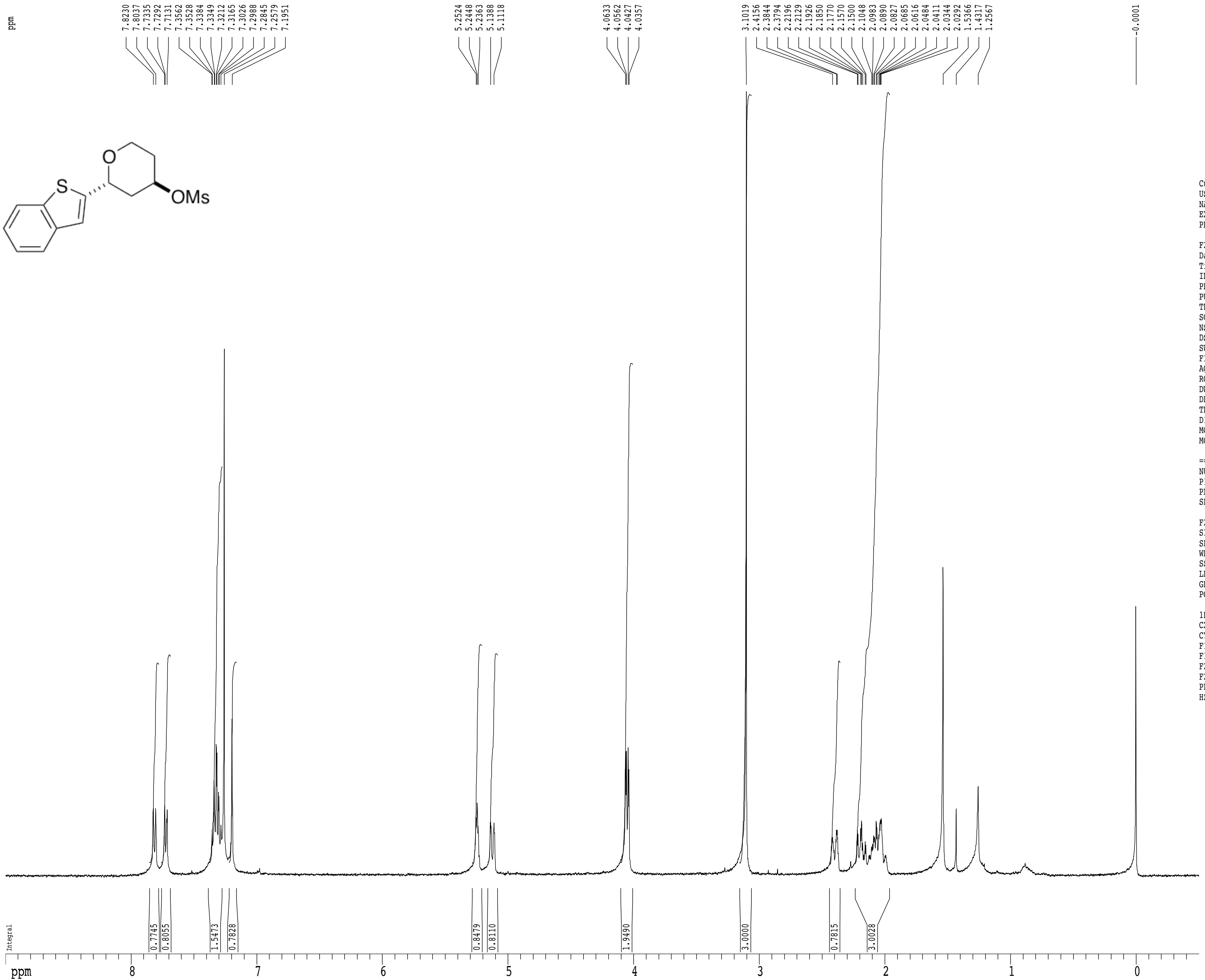

Current Data Parameters
USER linpc2
NAME pcl-3-115
EXPNO 1
PROCNO 1

F2 - Acquisition Parameters
Date_ 20220505
Time 11.09
INSTRUM drx400
PROBHD 5 mm QNP H/F/P
PULPROG zg30
TD 38460
SOLVENT CDCl3
NS 8
DS 2
SWH 6410.256 Hz
FIDRES 0.166673 Hz
AQ 2.9999299 sec
RG 912.3
DW 78.000 usec
DE 4.50 usec
TE 298.0 K
D1 0.10000000 sec
MCREST 0.00000000 sec
MCWRK 0.01500000 sec

======== CHANNEL f1 ========
NUC1 1H
P1 12.00 usec
PL1 -0.90 dB
SFO1 400.1328009 MHz

F2 - Processing parameters
SI 65536
SF 400.1300218 MHz
WDW EM
SSB 0
LB 0.30 Hz
GB 0
PC 2.00

1D NMR plot parameters
CX 22.80 cm
CY 15.00 cm
F1P 9.000 ppm
F1 3601.17 Hz
F2P -0.500 ppm
F2 -200.06 Hz
PPMCM 0.41667 ppm/cm
HZCM 166.72086 Hz/cm

1H spectrum

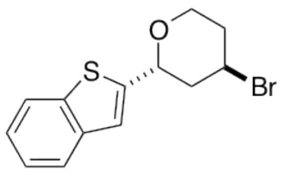

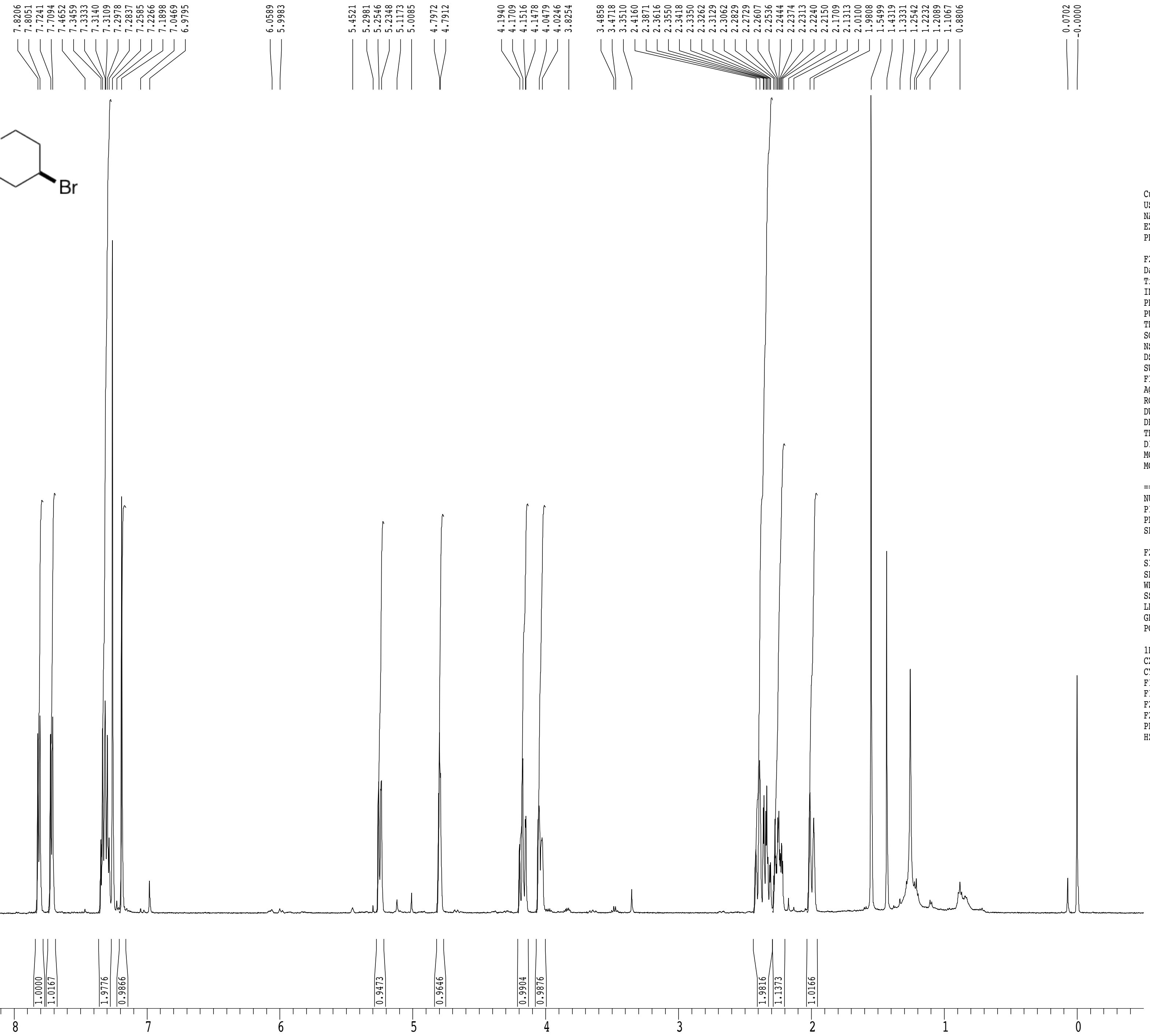

Current Data Parameters
USER linpc2
NAME pcl-3-110-cosy
EXPNO 1
PROCNO 1

F2 - Acquisition Parameters
Date_ 20220428
Time 11.10
INSTRUM cryo500
PROBHD 5 mm CPTCI 1H-
PULPROG zg30
TD 48074
SOLVENT CDCl3
NS 8
DS 2
SWH 8012.820 Hz
FIDRES 0.166677 Hz
AQ 2.9998677 sec
RG 5.7
DW 62.400 usec
DE 6.00 usec
TE 298.0 K
D1 0.10000000 sec
MCREST 0.00000000 sec
MCWRK 0.01500000 sec

======== CHANNEL f1 ========
NUC1 1H
P1 9.75 usec
PL1 1.60 dB
SFO1 500.2235015 MHz

F2 - Processing parameters
SI 65536
SF 500.2200332 MHz
WDW EM
SSB 0
LB 0.30 Hz
GB 0
PC 1.00

1D NMR plot parameters
CX 22.80 cm
CY 15.00 cm
F1P 9.000 ppm
F1 4501.98 Hz
F2P -0.500 ppm
F2 -250.11 Hz
PPMCM 0.41667 ppm/cm
HZCM 208.42502 Hz/cm

Z-restored spin-echo 13C spectrum with 1H decoupling

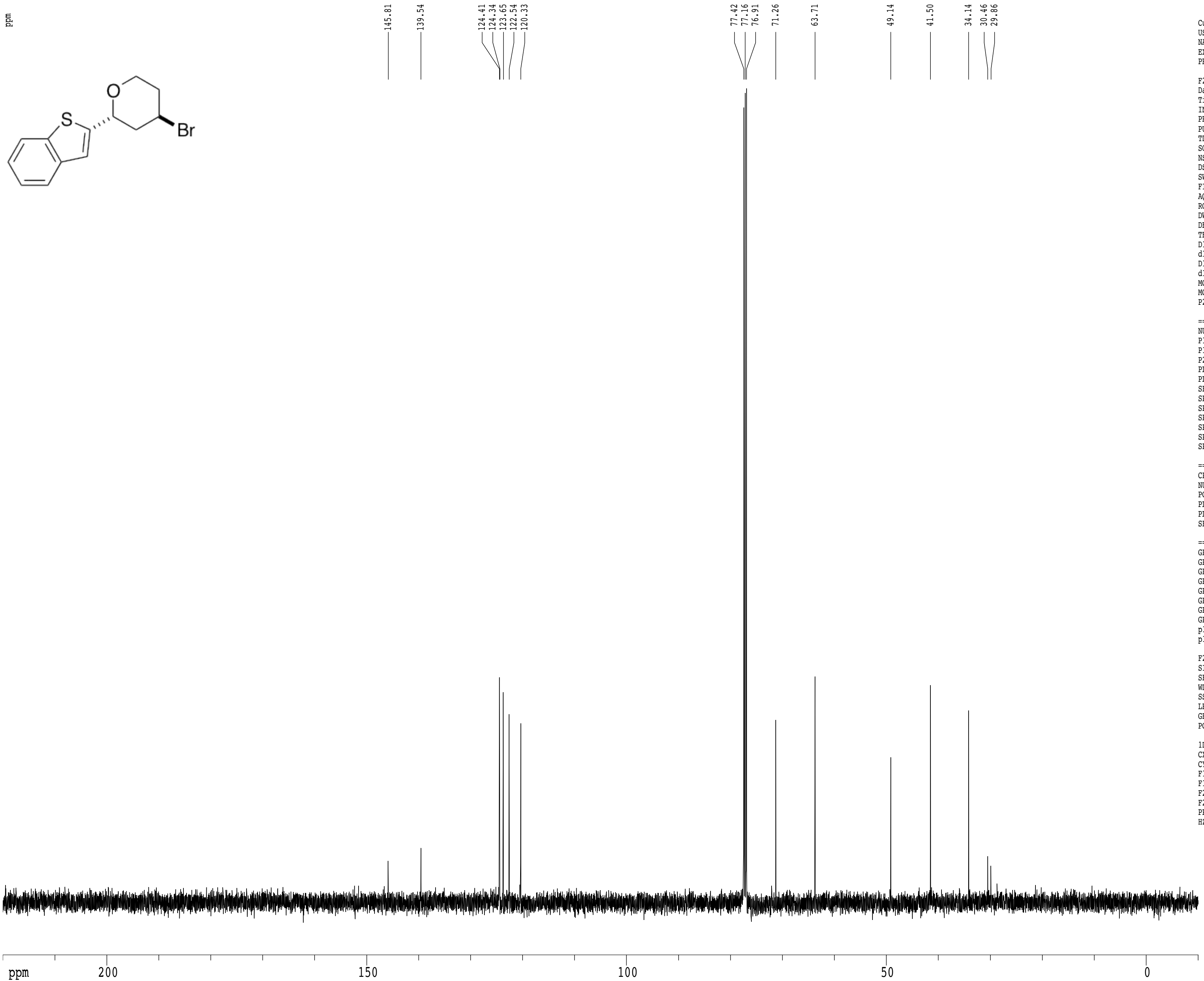

Current Data Parameters
USER linpc2
NAME pcl-3-110-cosy
EXPNO 3
PROCNO 1

F2 - Acquisition Parameters
Date_ 20220428
Time 11.28
INSTRUM cryo500
PROBHD 5 mm CPTCI 1H-
PULPROG SpinEchopg30gp2.prd
TD 65536
SOLVENT CDCl3
NS 280
DS 16
SWH 30303.031 Hz
FIDRES 0.462388 Hz
AQ 1.0813940 sec
RG 11585.2
DW 16.500 usec
DE 6.00 usec
TE 298.0 K
D1 0.25000000 sec
d11 0.03000000 sec
D16 0.00020000 sec
d17 0.00019600 sec
MCREST 0.00000000 sec
MCWRK 0.01500000 sec
P2 37.70 usec

======== CHANNEL f1 ========
NUC1 13C
P1 18.85 usec
P12 2000.00 usec
P20 500.00 usec
PL0 120.00 dB
PL1 -1.00 dB
SFO1 125.7942548 MHz
SP2 1.55 dB
SP4 1.55 dB
SPNAM2 Crp60comp.4
SPNAM4 Crp60,0.5,20.1
SPOFF2 0.00 Hz
SPOFF4 0.00 Hz

======== CHANNEL f2 ========
CPDPRG2 waltz16
NUC2 1H
PCPD2 100.00 usec
PL2 1.60 dB
PL12 22.00 dB
SFO2 500.2225011 MHz

====== GRADIENT CHANNEL =====
GPNAM1 SINE.100
GPNAM2 SINE.100
GPX1 0.00 %
GPX2 0.00 %
GPY1 0.00 %
GPY2 0.00 %
GPZ1 30.00 %
GPZ2 50.00 %
p15 500.00 usec
p16 1000.00 usec

F2 - Processing parameters
SI 65536
SF 125.7804080 MHz
WDW EM
SSB 0
LB 1.00 Hz
GB 0
PC 2.00

1D NMR plot parameters
CX 22.80 cm
CY 15.65 cm
F1P 220.000 ppm
F1 27671.69 Hz
F2P -10.000 ppm
F2 -1257.80 Hz
PPMCM 10.08772 ppm/cm
HZCM 1268.83752 Hz/cm

gcosy60

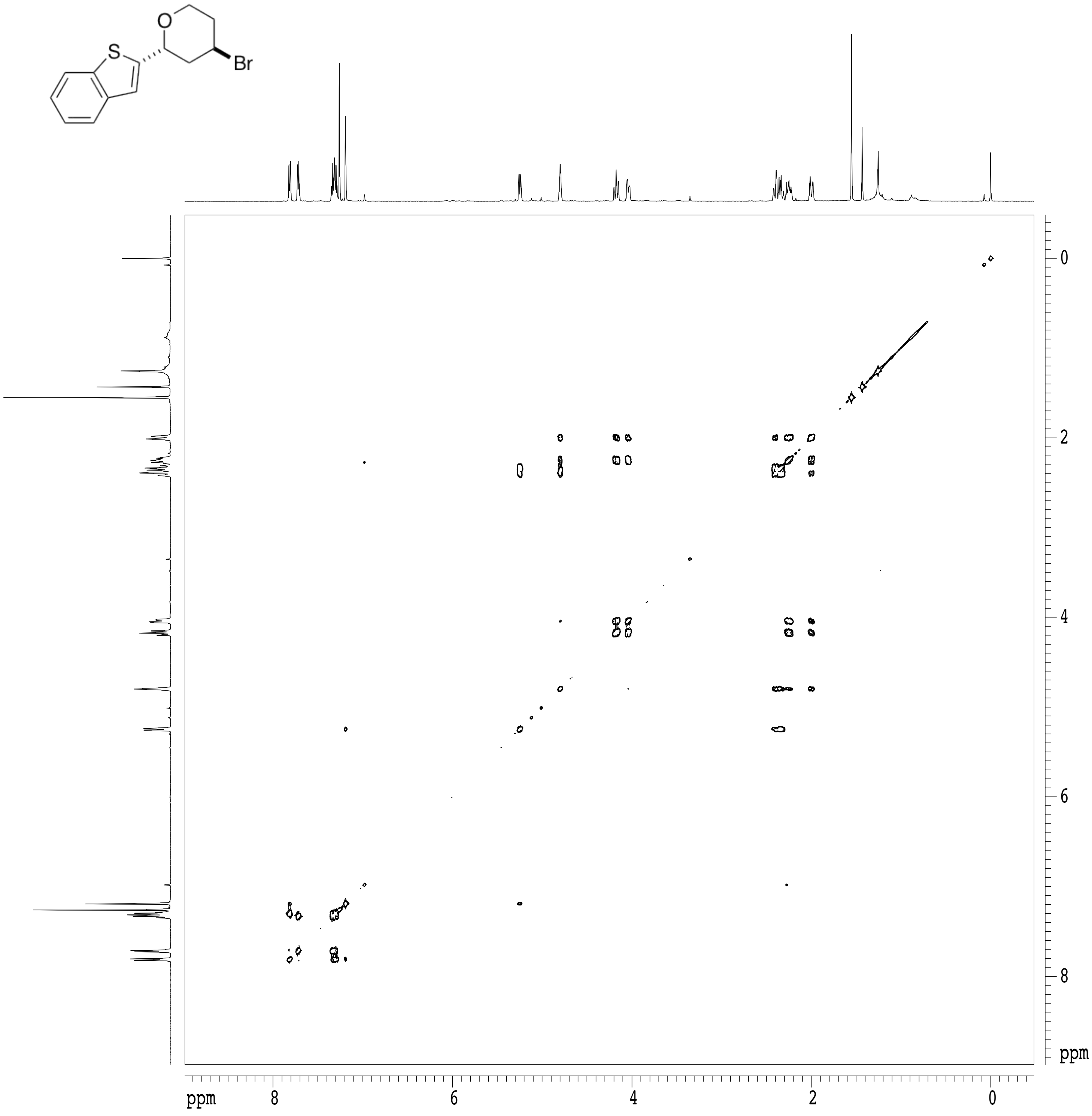

Current Data Parameters

USER	linpc2	
NAME	pcl-3-110-cosy	
EXPNO	2	
PROCNO	1	

F2 - Acquisition Parameters

Date_	20220428	
Time	11.13	
INSTRUM	cryo500	
PROBHD	5 mm CPTCI 1H-	
PULPROG	cosygp60.prd	
TD	2048	
SOLVENT	CDCl3	
NS	1	
DS	16	
SWH	4734.849	Hz
FIDRES	2.311938	Hz
AQ	0.2163188	sec
RG	512	
DW	105.600	usec
DE	6.00	usec
TE	298.0	K
d0	0.00000300	sec
D1	1.00000000	sec
d13	0.00000300	sec
D16	0.00020000	sec
IN0	0.00021120	sec

======== CHANNEL f1 ========

NUC1	1H	
P1	9.75	usec
PL1	1.60	dB
SFO1	500.2221592	MHz

====== GRADIENT CHANNEL =====

GPNAM1	SMSQ10.100	
GPNAM2	SMSQ10.100	
GPX1	0.00	%
GPX2	0.00	%
GPY1	0.00	%
GPY2	0.00	%
GPZ1	17.00	%
GPZ2	17.00	%
P16	1000.00	usec

F1 - Acquisition parameters

ND0	1	
TD	512	
SFO1	500.2222	MHz
FIDRES	9.247751	Hz
SW	9.465	ppm
FnMODE	QF	

F2 - Processing parameters

SI	1024	
SF	500.2200332	MHz
WDW	SINE	
SSB	0	
LB	0.00	Hz
GB	0	
PC	1.00	

F1 - Processing parameters

SI	1024	
MC2	QF	
SF	500.2200332	MHz
WDW	SINE	
SSB	0	
LB	0.00	Hz
GB	0	

2D NMR plot parameters

CX2	15.00	cm
CX1	15.00	cm
F2PLO	8.983	ppm
F2LO	4493.36	Hz
F2PHI	-0.483	ppm
F2HI	-241.49	Hz
F1PLO	8.983	ppm
F1LO	4493.36	Hz
F1PHI	-0.483	ppm
F1HI	-2[illegible]	[illegible]
F2PPMCM	0.63104	ppm/cm
F2HZCM	315.65659	Hz/cm
F1PPMCM	0.63104	ppm/cm
F1HZCM	315.65659	Hz/cm

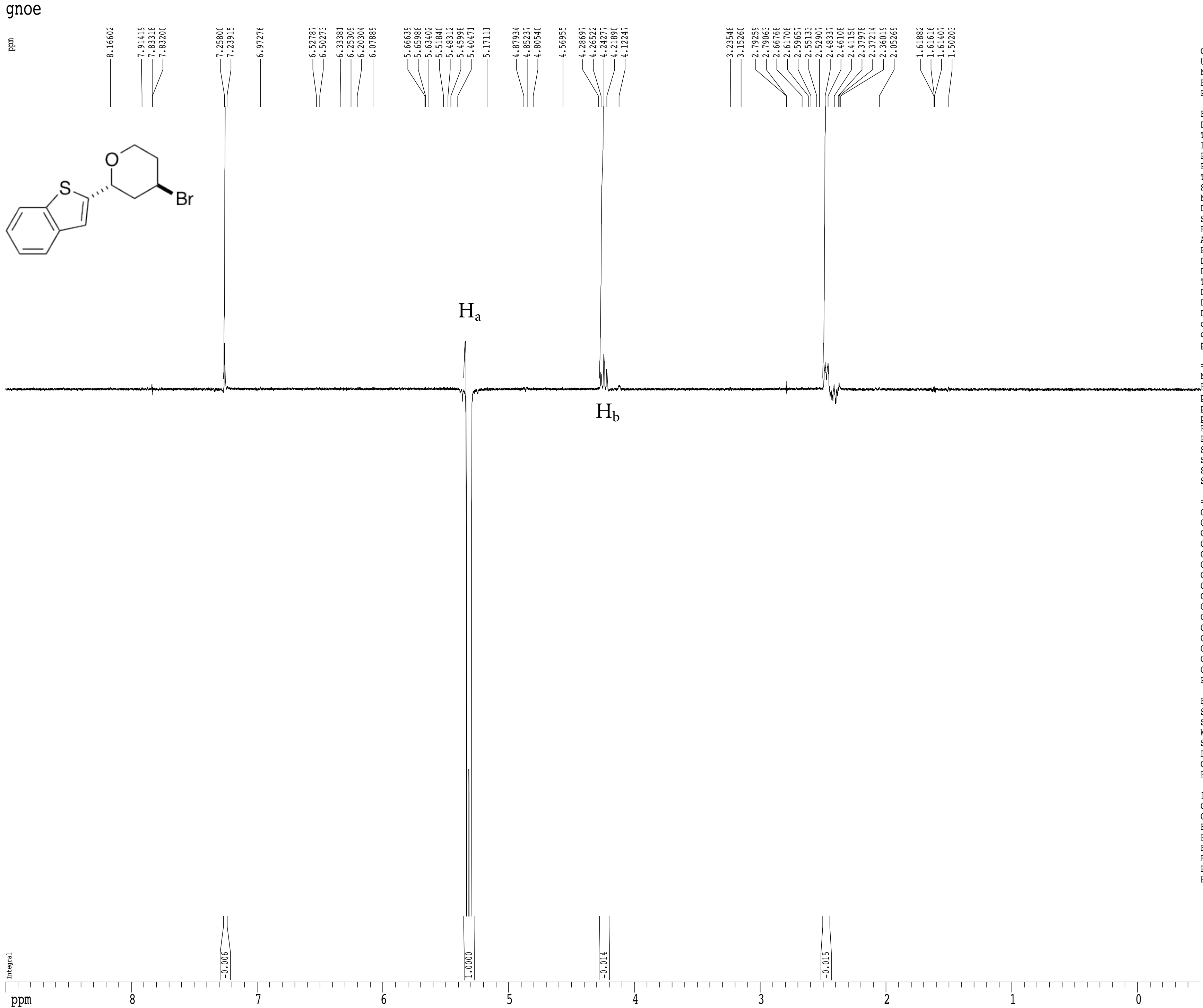

Current Data Parameters
USER linpc2
NAME pcl-3-110-noe
EXPNO 2
PROCNO 1

F2 - Acquisition Parameters
Date_ 20220428
Time 15.25
INSTRUM cryo500
PROBHD 5 mm CPTCI 1H-
PULPROG gnoe1cc22.prd
TD 65536
SOLVENT CDCl3
NS 128
DS 8
SWH 8012.820 Hz
FIDRES 0.122266 Hz
AQ 4.0894966 sec
RG 64
DW 62.400 usec
DE 6.00 usec
TE 298.0 K
D1 1.00000000 sec
D8 0.50000000 sec
D16 0.00020000 sec
d21 0.33375451 sec
d22 0.16399699 sec
p2 19.50 usec

======== CHANNEL f1 ========
NUC1 1H
P1 9.75 usec
p3 29.25 usec
p4 39.00 usec
p5 26.00 usec
P29 40000.00 usec
PL1 1.60 dB
SFO1 500.2226571 MHz
SP9 60.00 dB
SPNAM9 gauss1.512
SPOFF9 0.00 Hz

====== GRADIENT CHANNEL =====
GPNAM1 SMSQ10.100
GPNAM2 SMSQ10.100
GPNAM3 SMSQ10.100
GPNAM4 SMSQ10.100
GPX1 0.00 %
GPX2 0.00 %
GPX3 0.00 %
GPX4 0.00 %
GPY1 0.00 %
GPY2 0.00 %
GPY3 0.00 %
GPY4 0.00 %
GPZ1 7.00 %
GPZ2 3.00 %
GPZ3 2.30 %
GPZ4 -2.30 %
P16 1000.00 usec

F2 - Processing parameters
SI 65536
SF 500.2200000 MHz
WDW no
SSB 0
LB 0.00 Hz
GB 0
PC 1.00

1D NMR plot parameters
CX 22.80 cm
CY 50.00 cm
F1P 9.000 ppm
F1 4501.98 Hz
F2P -0.500 ppm
F2 -250.11 Hz
PPMCM 0.41667 ppm/cm
HZCM 208.42500 Hz/cm

1H spectrum

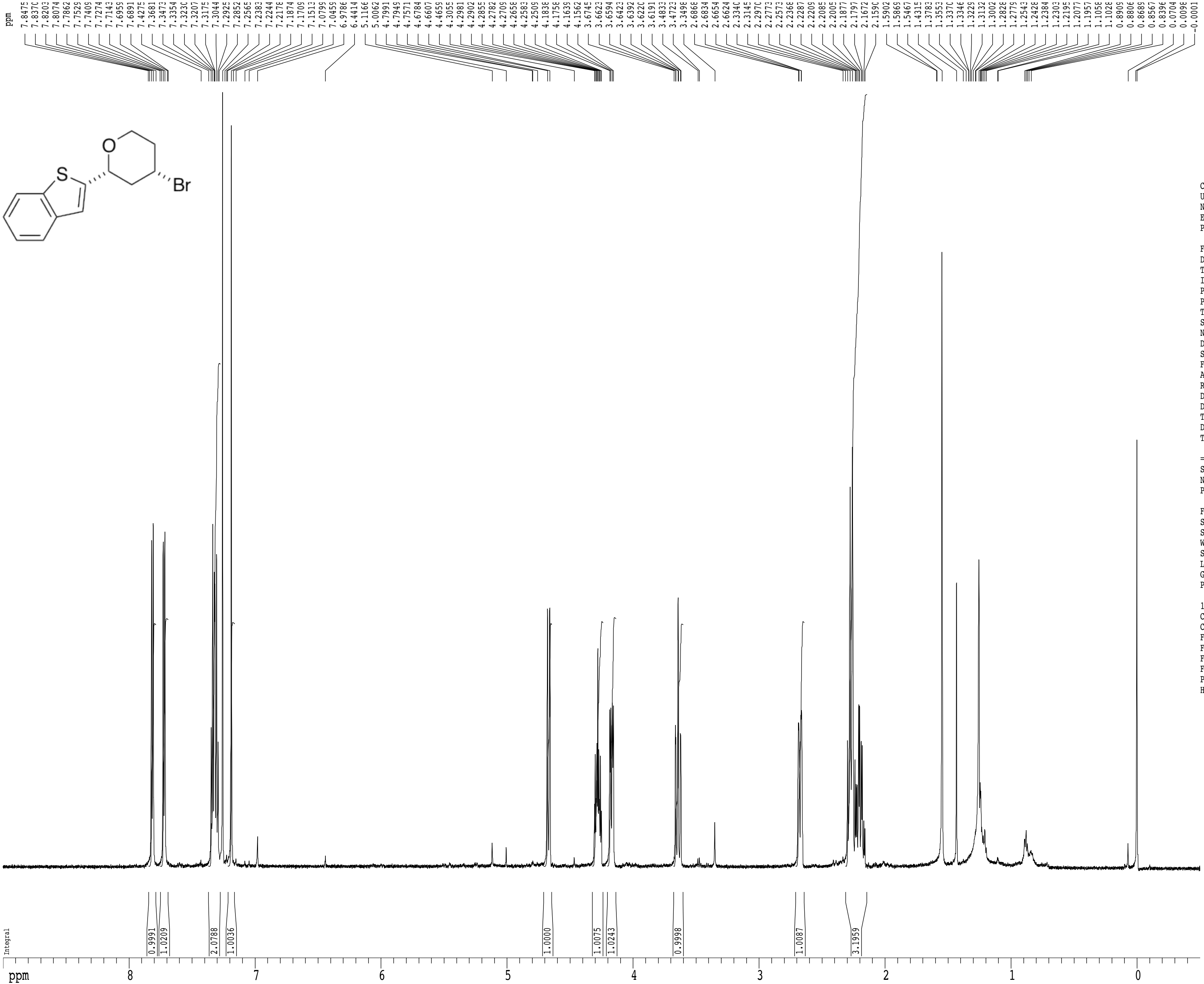

Current Data Parameters
USER linpc2
NAME pcl-3-116
EXPNO 1
PROCNO 1

F2 - Acquisition Parameters
Date_ 20220506
Time 15.22
INSTRUM av600
PROBHD 5 mm CPBBO BB-
PULPROG zg30
TD 98074
SOLVENT CDCl3T
NS 8
DS 2
SWH 9615.385 Hz
FIDRES 0.098042 Hz
AQ 5.0998979 sec
RG 10
DW 52.000 usec
DE 14.23 usec
TE 297.9 K
D1 0.10000000 sec
TD0 1

======== CHANNEL f1 ========
SFO1 600.1342009 MHz
NUC1 1H
P1 9.50 usec

F2 - Processing parameters
SI 65536
SF 600.1300375 MHz
WDW no
SSB 0
LB 0.00 Hz
GB 0
PC 1.00

1D NMR plot parameters
CX 22.80 cm
CY 15.00 cm
F1P 9.000 ppm
F1 5401.17 Hz
F2P -0.500 ppm
F2 -300.06 Hz
PPMCM 0.41667 ppm/cm
HZCM 250.05420 Hz/cm

13C spectrum

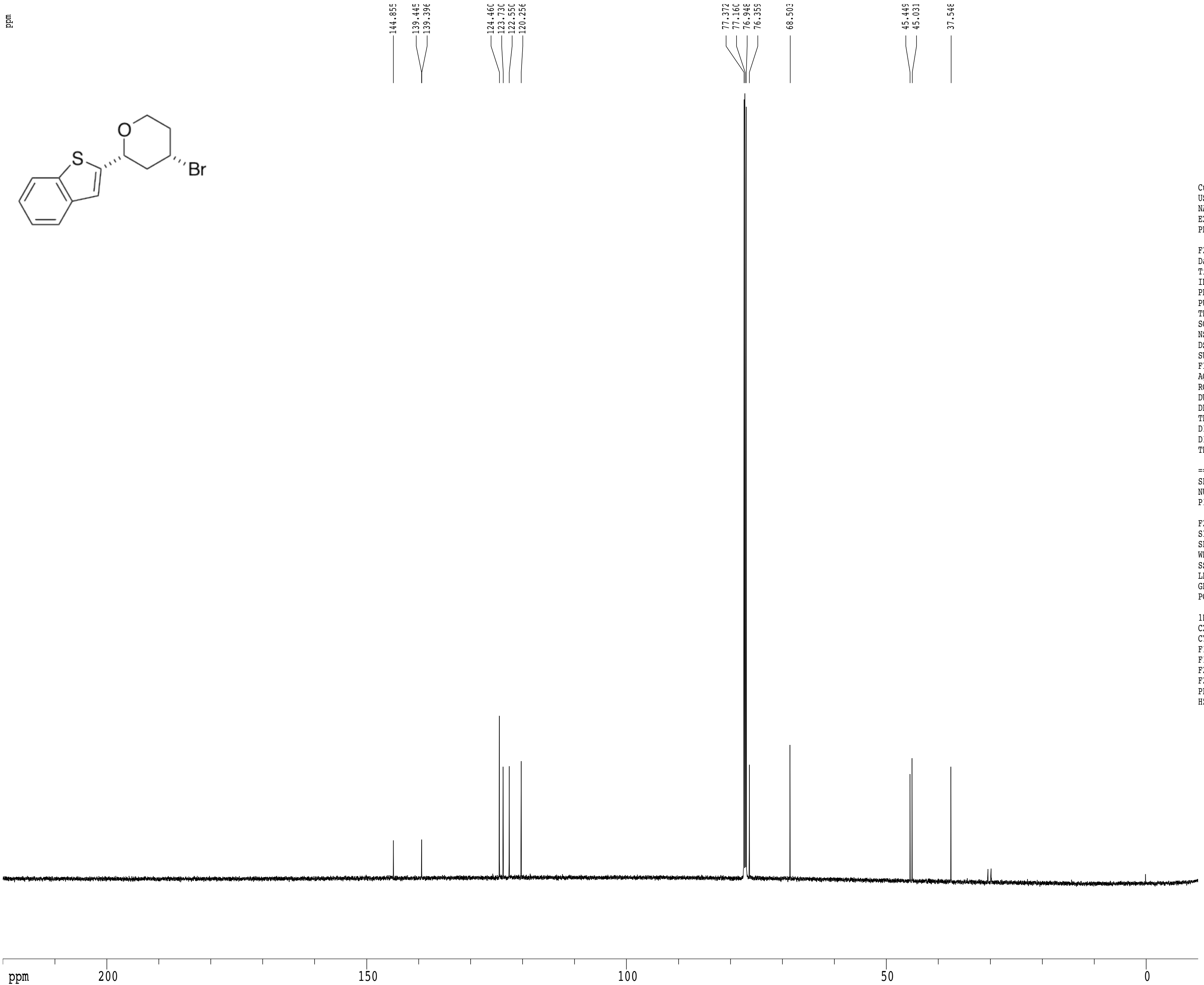

Current Data Parameters
USER linpc2
NAME pcl-3-116
EXPNO 2
PROCNO 1

F2 - Acquisition Parameters
Date_ 20220506
Time 15.28
INSTRUM av600
PROBHD 5 mm CPBBO BB-
PULPROG zgdc30
TD 65536
SOLVENT CDCl3T
NS 154
DS 4
SWH 36231.883 Hz
FIDRES 0.552855 Hz
AQ 0.9044468 sec
RG 2050
DW 13.800 usec
DE 19.63 usec
TE 298.0 K
D1 0.40000001 sec
D11 0.03000000 sec
TD0 1

======== CHANNEL f1 ========
SFO1 150.9194080 MHz
NUC1 13C
P1 10.10 usec

F2 - Processing parameters
SI 65536
SF 150.9027947 MHz
WDW EM
SSB 0
LB 1.00 Hz
GB 0
PC 1.00

1D NMR plot parameters
CX 22.80 cm
CY 15.00 cm
F1P 220.000 ppm
F1 33198.62 Hz
F2P -10.000 ppm
F2 -1509.03 Hz
PPMCM 10.08772 ppm/cm
HZCM 1522.26514 Hz/cm

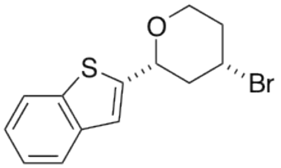

gcosy60

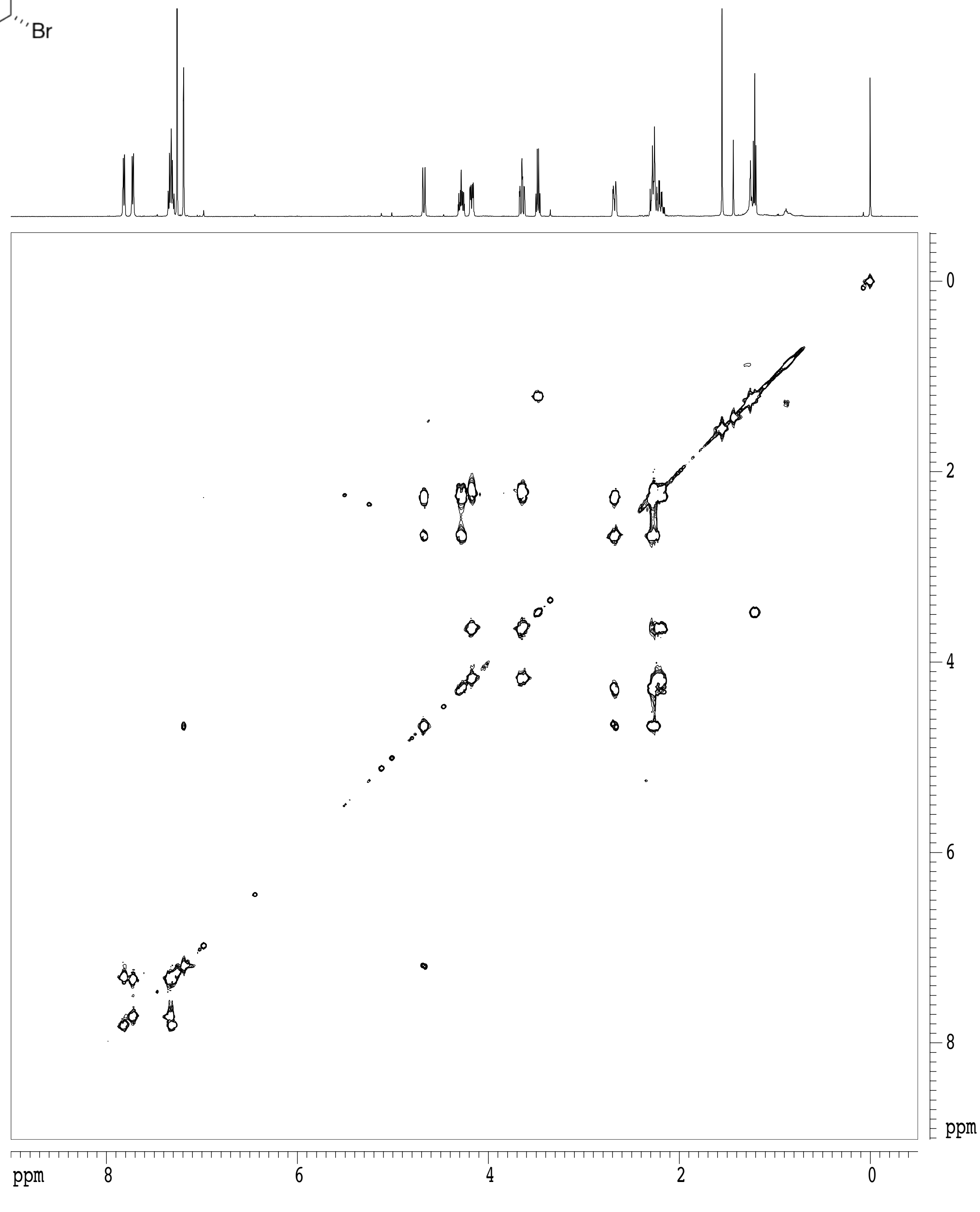

Current Data Parameters

USER	linpc2
NAME	pcl3-116-cosy
EXPNO	2
PROCNO	1

F2 - Acquisition Parameters

Date_	20220506
Time	7.26
INSTRUM	cryo500
PROBHD	5 mm CPTCI 1H-
PULPROG	cosygp60.prd
TD	2048
SOLVENT	CDCl3
NS	1
DS	16
SWH	8012.820 Hz
FIDRES	3.912510 Hz
AQ	0.1278452 sec
RG	512
DW	62.400 usec
DE	6.00 usec
TE	298.0 K
d0	0.00000300 sec
D1	1.00000000 sec
d13	0.00000300 sec
D16	0.00020000 sec
IN0	0.00012480 sec

======== CHANNEL f1 ========

NUC1	1H
P1	9.75 usec
PL1	1.60 dB
SFO1	500.2235015 MHz

====== GRADIENT CHANNEL =====

GPNAM1	SMSQ10.100
GPNAM2	SMSQ10.100
GPX1	0.00 %
GPX2	0.00 %
GPY1	0.00 %
GPY2	0.00 %
GPZ1	17.00 %
GPZ2	17.00 %
P16	1000.00 usec

F1 - Acquisition parameters

ND0	1
TD	512
SFO1	500.2235 MHz
FIDRES	15.650040 Hz
SW	16.018 ppm
FnMODE	QF

F2 - Processing parameters

SI	1024
SF	500.2200329 MHz
WDW	SINE
SSB	0
LB	0.00 Hz
GB	0
PC	1.00

F1 - Processing parameters

SI	1024
MC2	QF
SF	500.2200329 MHz
WDW	SINE
SSB	0
LB	0.00 Hz
GB	0

2D NMR plot parameters

CX2	15.00 cm
CX1	15.00 cm
F2PLO	9.000 ppm
F2LO	4501.98 Hz
F2PHI	-0.500 ppm
F2HI	-250.11 Hz
F1PLO	9.015 ppm
F1LO	4509.41 Hz
F1PHI	-0.512 ppm
F1HI	[illegible] Hz
F2PPMCM	0.63333 ppm/cm
F2HZCM	316.80603 Hz/cm
F1PPMCM	0.63511 ppm/cm
F1HZCM	317.69580 Hz/cm

gnoe

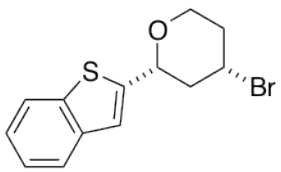

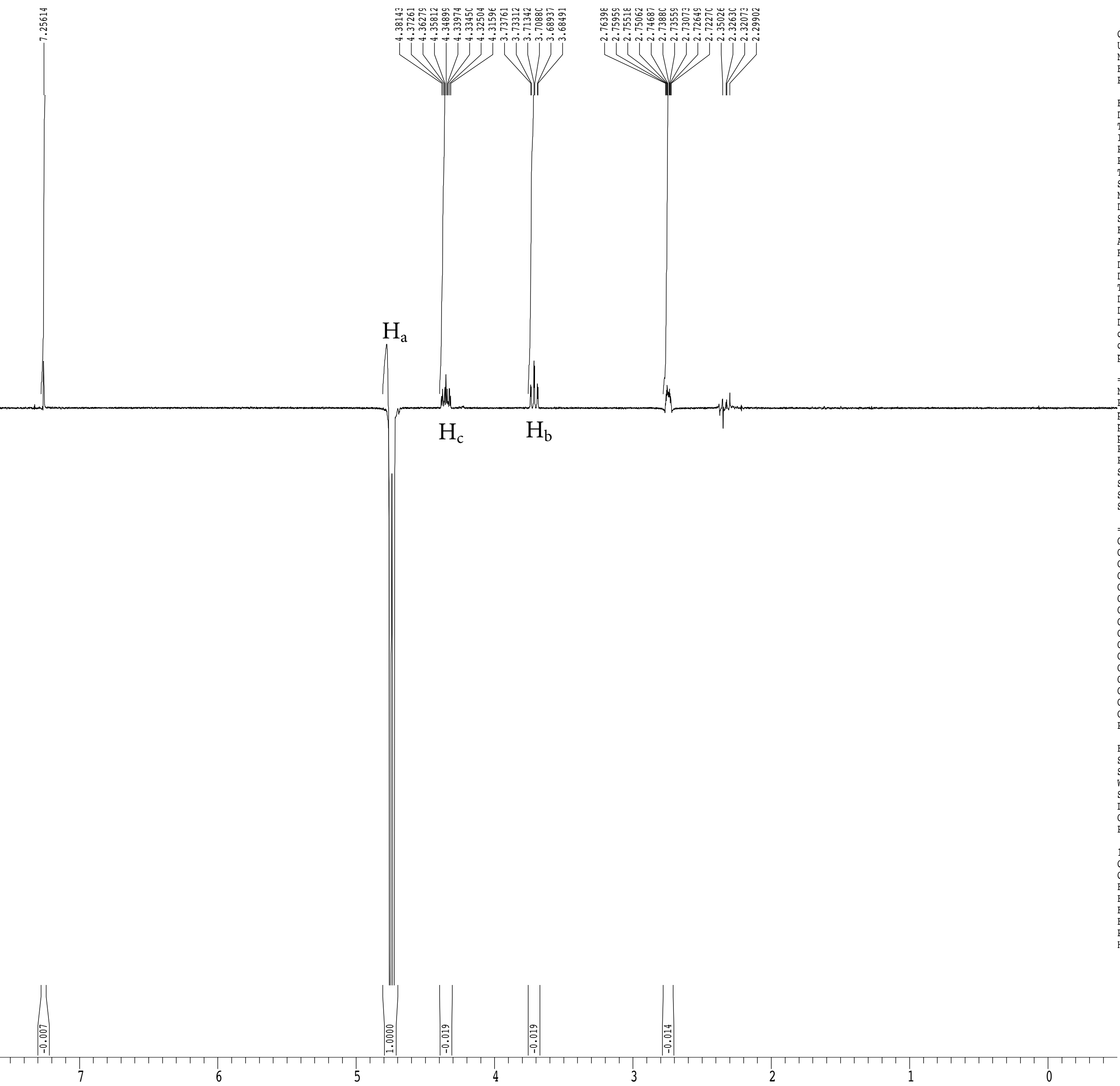

Current Data Parameters
USER linpc2
NAME pcl3-116-noe
EXPNO 2
PROCNO 1

F2 - Acquisition Parameters
Date_ 20220506
Time 7.55
INSTRUM cryo500
PROBHD 5 mm CPTCI 1H-
PULPROG gnoe1cc22.prd
TD 65536
SOLVENT CDCl3
NS 128
DS 8
SWH 8012.820 Hz
FIDRES 0.122266 Hz
AQ 4.0894966 sec
RG 80.6
DW 62.400 usec
DE 6.00 usec
TE 298.0 K
D1 1.00000000 sec
D8 0.50000000 sec
D16 0.00020000 sec
d21 0.33375451 sec
d22 0.16399699 sec
p2 19.50 usec

======== CHANNEL f1 ========
NUC1 1H
P1 9.75 usec
p3 29.25 usec
p4 39.00 usec
p5 26.00 usec
P29 40000.00 usec
PL1 1.60 dB
SFO1 500.2223698 MHz
SP9 60.00 dB
SPNAM9 gauss1.512
SPOFF9 0.00 Hz

====== GRADIENT CHANNEL =====
GPNAM1 SMSQ10.100
GPNAM2 SMSQ10.100
GPNAM3 SMSQ10.100
GPNAM4 SMSQ10.100
GPX1 0.00 %
GPX2 0.00 %
GPX3 0.00 %
GPX4 0.00 %
GPY1 0.00 %
GPY2 0.00 %
GPY3 0.00 %
GPY4 0.00 %
GPZ1 7.00 %
GPZ2 3.00 %
GPZ3 2.30 %
GPZ4 -2.30 %
P16 1000.00 usec

F2 - Processing parameters
SI 65536
SF 500.2200000 MHz
WDW no
SSB 0
LB 0.00 Hz
GB 0
PC 1.00

1D NMR plot parameters
CX 22.80 cm
CY 50.00 cm
F1P 9.000 ppm
F1 4501.98 Hz
F2P -0.500 ppm
F2 -250.11 Hz
PPMCM 0.41667 ppm/cm
HZCM 208.42500 Hz/cm

gnoe

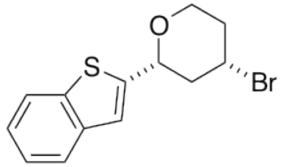

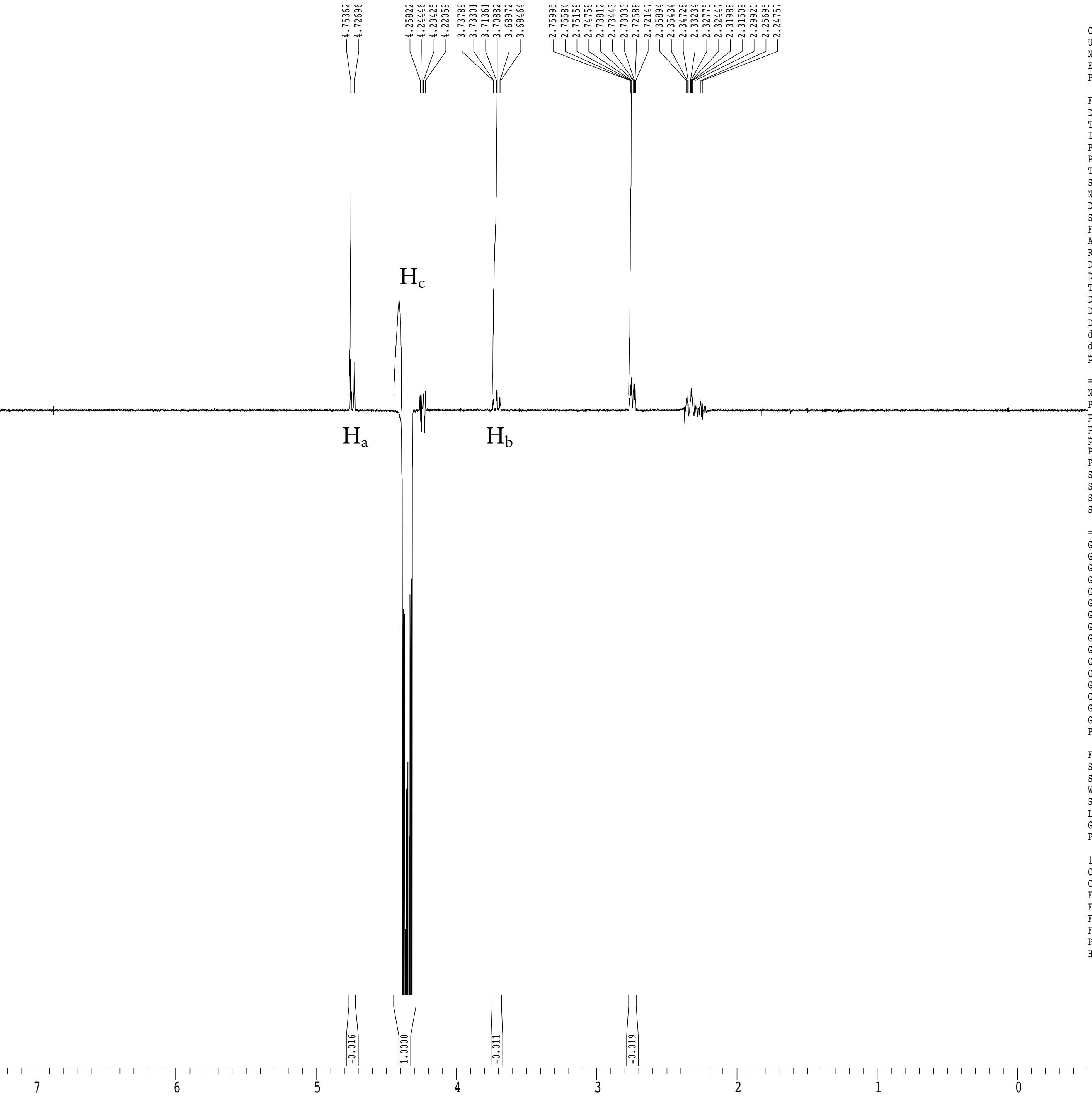

Current Data Parameters
USER linpc2
NAME pcl3-116-noe
EXPNO 3
PROCNO 1

F2 - Acquisition Parameters
Date_ 20220506
Time 8.09
INSTRUM cryo500
PROBHD 5 mm CPTCI 1H-
PULPROG gnoe1cc22.prd
TD 65536
SOLVENT CDCl3
NS 128
DS 8
SWH 8012.820 Hz
FIDRES 0.122266 Hz
AQ 4.0894966 sec
RG 101.6
DW 62.400 usec
DE 6.00 usec
TE 298.0 K
D1 1.00000000 sec
D8 0.50000000 sec
D16 0.00020000 sec
d21 0.33375451 sec
d22 0.16399699 sec
p2 19.50 usec

======== CHANNEL f1 ========
NUC1 1H
P1 9.75 usec
p3 29.25 usec
p4 39.00 usec
p5 26.00 usec
P29 40000.00 usec
PL1 1.60 dB
SFO1 500.2221742 MHz
SP9 60.00 dB
SPNAM9 gauss1.512
SPOFF9 0.00 Hz

====== GRADIENT CHANNEL =====
GPNAM1 SMSQ10.100
GPNAM2 SMSQ10.100
GPNAM3 SMSQ10.100
GPNAM4 SMSQ10.100
GPX1 0.00 %
GPX2 0.00 %
GPX3 0.00 %
GPX4 0.00 %
GPY1 0.00 %
GPY2 0.00 %
GPY3 0.00 %
GPY4 0.00 %
GPZ1 7.00 %
GPZ2 3.00 %
GPZ3 2.30 %
GPZ4 -2.30 %
P16 1000.00 usec

F2 - Processing parameters
SI 65536
SF 500.2200000 MHz
WDW no
SSB 0
LB 0.00 Hz
GB 0
PC 1.00

1D NMR plot parameters
CX 22.80 cm
CY 50.00 cm
F1P 9.000 ppm
F1 4501.98 Hz
F2P -0.500 ppm
F2 -250.11 Hz
PPMCM 0.41667 ppm/cm
HZCM 208.42500 Hz/cm
